# Supplementary material for: Identification and Validation of a Novel Ferroptotic Prognostic Genes-Based Signature of Clear Cell Renal Cell Carcinoma
Source: Cancers (Basel). 2022 Sep 27;14(19):4690. doi: 10.3390/cancers14194690 (PMC9562262; doi:10.3390/cancers14194690)
Supplement: Supplementary file 1 [file cancers-14-04690-s001.zip › Supplementary Materials Figures.pdf]

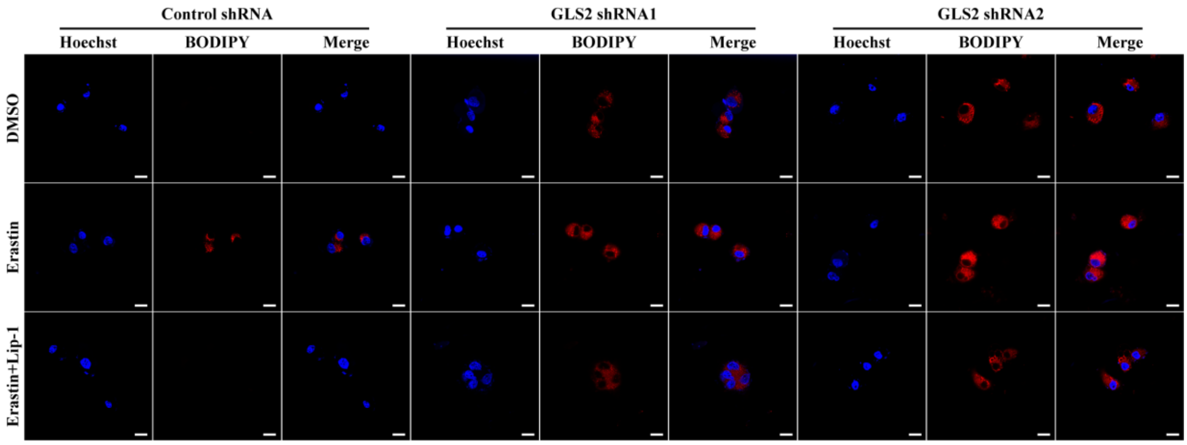

Figure S1. Lipid peroxidation of Caki-1 with various treatments (Nuclei were stained with Hoechst 33342, and lipid peroxides were stained with BODIPY 665_676). The scale bar repre-sented 20 μm.

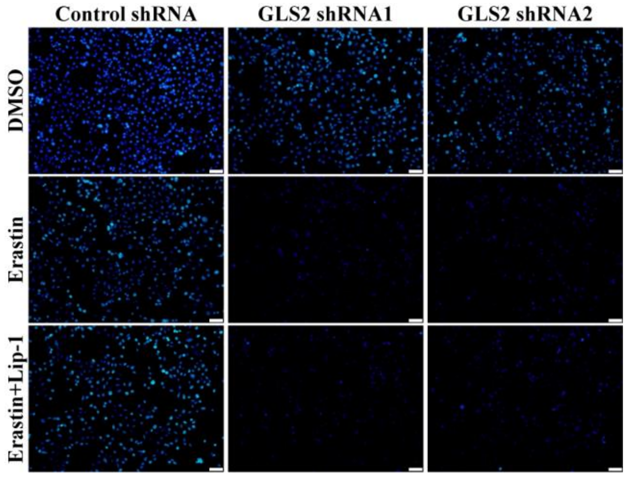

Figure S2. The status of intracellular GSH levels was assessed by MBB staining in ACHN treated with erastin (10 μM, 12 h) in the absence or presence of Lip-1 (1 μM). The scale bar represented 100 μm.

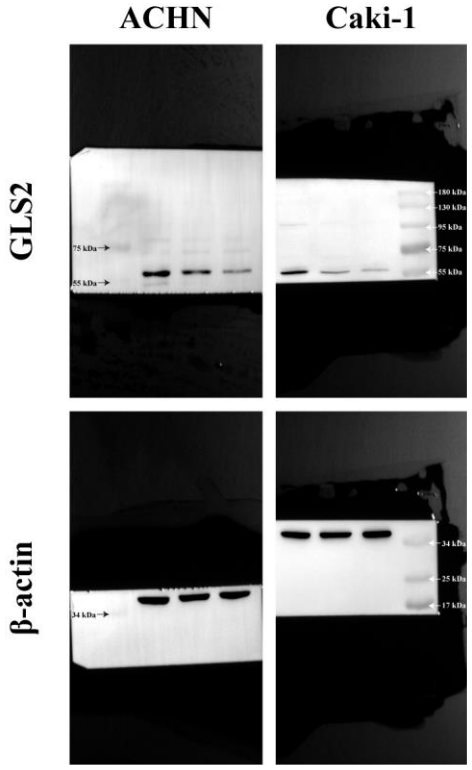

Figure S3. Original whole blot.
