# Supplementary material for: Identification and Validation of a Novel Ferroptotic Prognostic Genes-Based Signature of Clear Cell Renal Cell Carcinoma
Source: Cancers (Basel). 2022 Sep 27;14(19):4690. doi: 10.3390/cancers14194690 (PMC9562262; doi:10.3390/cancers14194690)
Supplement: Supplementary file 1 [file cancers-14-04690-s001.zip › Table S1 Primer sequences of genes.pdf]

Table S1 Primer sequences of genes

Gene		Sequence(5'-3')
β-actin	F	CGTGCGTGACATTAAGGAGAAG
	R	GGAAGGAAGGCTGGAAGAGTG
DPEP1	F	CCCAAAGGGTGACAAGC
	R	CCTGCGGGCACTCTATC
NOX4	F	GCACAGTACAGGCACAAAGG
	R	ATTTAGATACCCACCCTCCC
MT1G	F	GCTGCACTTCTCCGATGC
	R	TGCCGCTGGTGTCTCCT
GLS2	F	GAAATTCGGAACAAGACTGTG
	R	AACTTCGATGTGTCCTTCAG
GLRX5	F	GGGACCCTTGGAACACC
	R	CCTGCGGGCACTCTATC
TIMP1	F	TGTGGGACCTGTGGAAGTA
	R	TGTTGTTGCTGTGGCTGAT
CA9	F	GGAGCCCTCTTCTTCTGATTTA
	R	GCTGCTTCTGGTGCCTGTC
CDCA3	F	TCCGTGCAATACCAAGAGTA
	R	AGCCAAGAGCGTCCCA
CYBB	F	TGAGAATGGATGCGAAGG
	R	CTAAGATAGCGGTTGATGGG
