# Supplementary material for: Identification and Validation of a Novel Ferroptotic Prognostic Genes-Based Signature of Clear Cell Renal Cell Carcinoma
Source: Cancers (Basel). 2022 Sep 27;14(19):4690. doi: 10.3390/cancers14194690 (PMC9562262; doi:10.3390/cancers14194690)
Supplement: Supplementary file 1 [file cancers-14-04690-s001.zip › Table S2 List of DEGs from three GEO databases.pdf]

Table S2 List of DEGs from three GEO databases.

Gene name
KNG1
UMOD
CALB1
FABP1
SLC12A1
HPD
NPHS2
SLC22A8
DIO1
KCNJ1
CLDN8
SLC7A13
RALYL
ALB
NDUFA4L2
NPTX2
MUC15
SLC13A3
CLCNKB
SERPINA5
CTXN3
FXYD4
XPNPEP2
TMEM213
G6PC
ATP6V0A4
TFAP2B
PLG
DPEP1
HRG
ENPP6
TNFAIP6
DMRT2
HEPACAM2
CRYAA
AFM
FAM151A
PVALB
ANGPTL4
SLC22A7
SH3GL2

UPP2
SOST
TYRP1
ALDOB
RHCG
CA10
PCP4
FLJ22763
EHF
SLC26A7
GPC5
ERBB4
FGF9
SFRP1
IGSF11
LPPR1
LOC149703
DNMT3L
ATP6V1G3
TFCP2L1
FABP6
SLC22A6
IYD
IRX2
SLC47A2
RAB25
CA9
HK2
SOSTDC1
CYP4F3
HILPDA
FABP7
TUBAL3
SCN2A
EGF
NPHS1
TREM2
C5orf46
ENO2
FAM3B
MTTP
ACPP
SLC5A11

NKG7
RHBG
SLC4A1
PSAT1
CDCA2
TSPAN8
FGF1
ATP6V0D2
DDN
TREH
TCEAL2
OLFM4
ADH1C
ANGPTL3
SLC12A3
C3
PIGR
AZGP1
CNTN3
AHNAK2
MIOX
COL23A1
SLC6A3
CWH43
CLIC5
SLC34A1
PPFIA4
HAO2
CRHBP
CHL1
PROM2
APOH
CTHRC1
CCL20
PTH1R
SLC22A13
SLC28A2
NXPH2
RGS1
LOX
QRFPR
TMPRSS4
MAL

CYP27B1
PCK1
ABAT
CLSTN2
VTCN1
REEP6
APOC1
LDHD
PROZ
PPP1R1A
ACSF2
ATP6V1C2
INHBB
PIPOX
HSD11B2
DAO
APOC3
PROC
ADH6
TMEM45A
SLC5A2
DOC2A
S100A2
PRODH2
PPAPDC1A
SLC13A1
TNNC1
BHLHE41
ASPM
NR1I3
PLCXD3
WT1
C7
DIRAS2
ALDH6A1
AGPAT9
COL4A6
MT1G
C1orf168
C16orf89
ESRRG
PAPPA
CXCL13

LOC645321
DUSP9
CCL18
ST8SIA4
PIK3C2G
EGLN3
NETO2
SPAG4
CYP4A11
RDH12
SLAMF7
FMN2
DNER
FKBP10
FAM26F
STAP1
DCXR
RANBP3L
OTOGL
TRPA1
ACOT12
GABRA2
IRX1
SLC5A12
SIM1
ANGPT2
MFSD4
IGFBP3
RBP4
DNAH11
TAC1
CDH3
WNK4
NNMT
PNCK
HIGD1B
DEFB1
HPGD
C14orf37
FCAMR
CD70
DDC
TMEM61

TNFRSF4
SEMA5B
SORCS1
NR0B2
LPCAT1
ASS1
SLC16A9
LGSN
TUBB2B
CCBE1
GPD1
ADAMDEC1
CYP2J2
IDO1
LOXL2
VCAN
GPC3
SUCNR1
MYH8
SLC7A8
MRO
OGDHL
LAMA4
FOXI1
CXCR4
CD300LF
ZYG11A
PLA2G7
WIF1
FBP1
SLC26A4
TGFBI
TRIM63
SCARB1
GLDC
FREM1
C4orf6
CDH13
PLA2R1
CAV1
GRHL2
CDH9
IL17RB

ANO5
MT1H
BSND
STC2
SUSD2
RNASET2
PFKFB4
CHGB
MIR210HG
TMCC1
SUSD4
FAM169A
F11
KCNJ13
FOLR3
LYPD6B
SORD
EFHD1
LRRC19
SLC30A2
IL4I1
LOC389332
LINC00473
CYP8B1
TCF21
PHF21B
CRTAM
E2F8
CP
PHYHD1
AQP6
VAV1
PDK1
BIN2
CLDN16
RASL11B
BIRC3
CPNE6
ERP27
CXCL9
FMO5
INPP5J
DNAJC12

BIK
APOM
ERVMER34-1
ANXA9
MAN1C1
ANK2
MCOLN3
DLGAP5
PFKP
HMGCS2
ALDH4A1
PCK2
GZMH
IGSF6
CEP55
CAV2
ESRP1
PCDH9
SLC17A4
DACH1
EDDM3A
SCGN
MT1F
SHISA3
ENPP3
CES4A
TMEM72
BRCA2
SLC44A4
CNDP1
EHD2
TRIB3
HAO1
SLC27A2
STK33
TRIM9
NAT2
DCN
CORO1A
CXCL11
PEPD
MRAP2
PRR15L

HOGA1
CXCL10
ABCA12
LRRC2
BCHE
ALOX5
SLC23A1
NOX4
SLC7A9
AOX1
CTSS
PLCL1
SOX11
PHYHIP
MME
SCD
SLC2A12
CLDN19
C1orf116
SLC17A1
DHDH
GNLY
PLK2
P2RX7
GATA3
GZMK
WDR72
TEX11
AGXT2
CYP24A1
DDB2
LHX1
C1QC
C1QA
FPR3
LRP1B
PC
S1PR5
KIF20A
DTL
PRAP1
ADM
C1QB

LRRC25
IRX3
SLC16A10
ACOX2
ZNF395
DPEP2
FABP5
AP1M2
C9orf135
LILRB2
CA4
SAP30
CSPG4
RPS6KA6
PRRG2
HTRA4
ETNK2
FN1
SVOPL
APOLD1
SLFN13
CCR5
STAMBPL1
TLR7
SIRPG
KLK1
SLIT2
SEMA6D
PRIMA1
SPINK13
ARG2
RHOH
UGT3A1
ESM1
CCL5
CHP2
RGS7
RAB42
PLCD4
PNMA2
DPYS
GSTM3
ANGPTL1

KCNK3
TSPAN1
GJC1
RASSF10
SIGLEC10
C6orf123
APOL1
DDIT4
FRMD7
ASPDH
PP7080
COL8A1
ANO4
BMPR1B
PTHLH
ITGB2
SLAMF8
TLR3
FAP
CXCL5
CD1D
LYZ
C9orf84
TMPRSS2
KL
HADH
CPNE4
SPTBN2
LAD1
CORO2B
NECAB2
CD33
MPPED2
C2orf40
CLDN10
MSR1
AIF1L
CPA6
QPRT
STRA6
SFXN2
IKZF1
MT1E

CENPM
KCNJ2
GGH
MPP7
MCAM
CKMT2
EBF2
LCP2
RASSF2
EPCAM
CENPK
LOC100506125
ARSF
ISG20
HLA-DQB1
UPK1B
PDE1A
CLUL1
SUCLG1
DDX25
TNFSF13B
MS4A7
UBASH3A
CD86
NRG3
ADAMTS16
AGR3
TYROBP
ADAMTS19
GZMA
BTK
RPRM
CHST15
RRM2
EPHA3
IL3RA
PNPLA1
ANLN
TMC4
NUF2
RAC2
CD36
C8orf22

SH2D1A
SLC7A11
COL21A1
OLFML2A
SLC4A9
CDH16
CRISP2
SLC1A3
KLK7
GAS2L3
TLR8
HSPA6
NOL3
FUT3
ALLC
DEF6
CTSW
DLX5
LOC100505938
FCGR1B
MYBL1
CRYM
MOGAT1
EMX1
XCL1
PMCH
GAL3ST1
LRRN1
C10orf10
NDNF
NAPSA
PAG1
LAPTM5
VAT1L
EOMES
OLFML2B
CLEC14A
TRAT1
RBM11
SCGB2A1
CENPE
LIX1
CTH

SLC39A5
CAPN3
CD2
OXGR1
TIMP1
COL1A1
FOLR1
TPX2
PTPRC
UHRF1
SLC7A7
PSMB9
BHMT
PYCARD
PROX1
LILRB1
COLEC11
ALDH8A1
KCNJ15
ITK
ALAD
LGI4
ITGA4
CST7
SLFN11
CD247
HSPA2
GLTPD2
ACSBG2
BEX1
TLR2
ACAA1
PRDM1
GZMB
MELK
RGS5
C4orf47
SIGLEC8
KLRC3
NEK2
GABRP
IGFBP2
GJA1

GRB14
IGF2BP3
FOXM1
ALDOC
CCL4
FBLN5
PLOD2
LGALS1
PHKA2
NAP1L2
OSBPL6
NCKAP1L
HYAL1
KLRG2
ADORA3
C9orf66
CCND1
NKAIN4
AKR7A3
MCHR1
GABRD
PTGS1
BPHL
LAT2
CD163
EPHX2
SGK2
CSTA
LPAR5
ICOS
IGSF5
NPNT
APBB1IP
PTGDS
ITGA5
SCG5
CDCA7
CLEC2B
REEP2
PALM3
EVI2A
PRKCDBP
TRPV2

UCHL1
PLXDC1
KRT7
KCNMA1
PRF1
BTNL9
MAGI2-AS3
TAP1
FXYD5
PPP1R3C
EMR2
COL6A2
FLJ32255
MFSD3
LAMP3
TTK
RGN
BTN3A2
P4HA3
USP44
PAK6
CLCN5
CDCA7L
TIGIT
KIAA0101
GPR65
TST
C12orf56
TAGAP
PAQR5
C1orf210
IL20RB
KCNE4
SLC16A7
GSTO2
VDR
FAM57A
SLC15A2
GMPR
SERPINA4
ENTPD1
TMEM45B
SELENBP1

CD3D
IL10RA
CCL11
ERV3-2
HLX
AXL
IFI44L
ARHGAP9
ABCG1
MACROD1
P2RY12
PPP1R3B
CLEC4A
PBK
FCGR2B
ABCB1
SMTNL2
CCNE2
LOC154761
GPD1L
SLC15A4
HLA-DQA1
TYRO3
CAPSL
SLC25A10
DOCK10
IL2RB
TAGLN3
SERPINE1
DCLK1
TYMP
CHSY3
CD180
ACSM2B
EGFR
HCLS1
TMEM155
GDA
EYA2
ACSM2A
SAT2
RNF152
KLK6

CSF2RA
PLIN2
RERGL
HAPLN1
NOD2
APOBR
NAT8B
CD8A
ACSM3
SIRPB2
TNFRSF9
HFM1
GBP5
MT1X
FLRT3
TINAG
ANK3
MARVELD2
MAL2
VNN2
AQP1
PSMB8
LAIR1
CDC45
MNDA
P2RY8
ACAT1
SLC43A3
GGTA1P
ABCA4
GPT2
SLC38A1
UGT2B28
TNFSF9
BDH2
MND1
GCGR
PABPC4L
TACC3
HLA-DPB1
SCG2
CPE
LHFPL2

AMDHD1
VWCE
SCN4B
PLVAP
TNIP3
PECAM1
MUC13
CHODL
RAD51AP1
APOBEC3C
AQP9
IKBIP
BARX2
FERMT3
HSF4
HAVCR1
ODAM
CARD16
ADA
TMC8
HOXB8
BCL11A
MS4A4A
MARVELD3
EBI3
APOBEC3G
CX3CR1
MCF2L-AS1
CD72
PKLR
ARHGAP30
KCNMB2
IRF6
PARVG
ANO1
DHRS11
TGFA
CDO1
IFNG
RTP4
GJB1
DIRAS3
ITGAL

PRSS8
ATP6V0E2
ECHS1
LPPR5
ANXA3
HLA-DOB
CYBB
CD38
MILR1
PYGL
RUNX2
ADORA2B
SERPINC1
ALS2CL
UNC5D
ARL4D
LINGO2
SPC25
FA2H
IL7R
MECOM
FBXL16
FMO4
TGFB1
SAMHD1
SPI1
COL5A3
GPHN
IFI16
CDCA8
PNPLA3
PGBD5
MT1M
CGN
C1orf162
LONRF2
CDKN1C
OSMR
KRBA1
PCDH17
SLC26A9
PRC1
NLRC4

NLRC5
TJP3
CCRL2
DACH2
RNF150
AQP11
ATP1A1
OVOL2
PIK3CG
HJURP
EZH2
SIGIRR
SLC23A3
CASP1
LAG3
RARRES2
BBOX1
C5
TCF4
NFATC2
SPAG5
IP6K3
ADCY7
HCK
HIBCH
UCN
SELPLG
MSC
HIST1H2AM
GPNMB
CDH4
FUT11
VIM
EVI2B
CASC5
FCER1G
LY86
GBP2
LCK
HEY1
VEGFA
SAMSN1
QDPR

ATP8B3
SERPINH1
NR3C2
C8orf4
NUSAP1
LOC100507537
SNX20
SFTA2
BSPRY
HLA-F
PHYH
PPM1H
CTSH
HHLA2
C9orf24
LRRK1
TOX2
CMTM4
CHIT1
NCAPH
CRYL1
44621
GBA3
44622
C19orf33
HCP5
HIST1H2BK
NQO2
ATHL1
SOBP
ARHGDIB
NAT8L
ENPP1
CAPS
ADAM18
NDC80
CHST11
ASB9
ZNF300P1
C3AR1
ECI2
GBP1
KLHL13

AIM2
MYOF
SEL1L3
POU5F1P4
LZTS1
HGD
SLC9A2
TLCD1
CENPF
GCH1
HOMER2
MARCKS
KLRD1
FRAS1
CPA3
SLC15A3
LIPA
EMILIN2
GPR4
MEST
SSPN
HMCN1
SULT1C2
HLA-DMA
CD300C
FGFBP1
PTGFR
TMED6
ZNF503
PNP
TSPAN33
NFASC
FOXC1
CD14
EDIL3
ARRDC2
HS3ST2
TMEM92
COBLL1
GPR85
RNF43
GGT5
RAPGEF3

OXCT1
TBC1D24
CACNB4
PDGFD
APOD
TNMD
ACADM
ZAP70
HLA-DRA
SIT1
BCAT1
BDNF
CLECL1
IL13RA2
TYMS
PPARGC1A
BTN3A3
UQCRC1
ACTG2
FAM64A
LHPP
ITLN1
FLRT1
CRNDE
C11orf54
TPSAB1
NPY1R
CYFIP2
KIF4A
HLA-DMB
RNASE2
RIMKLA
FAM49A
ZC3HAV1L
PMEPA1
LRP1
TM7SF2
FLJ13744
RIPPLY1
KCNK5
PREX1
IGSF22
CYP4F12

FCGR3B
DSP
RASD1
PPP1R36
AEN
AFAP1L1
COL5A2
PROCR
DMGDH
RSPH9
PSORS1C1
NCF1
TBXAS1
ENOX1
AURKB
SLC16A5
FYB
BCL11B
MUM1L1
TRPC2
MICB
LOC400043
MS4A14
PTTG1
KSR1
IL21R
HRSP12
NCF2
CDT1
PPP1R18
PTPN22
RUNX3
KLHL6
PLA2G4F
CCL28
GPR19
TRIM22
CDC6
NLRP3
PLEK
BDKRB2
ABCB4
PANK1

GPR34
HPCAL1
CXorf36
ARHGAP25
NINL
ANGPTL2
DUSP26
LIPH
TDRD9
C2orf54
ADI1
P4HA1
SLC25A25
SACS
CDK18
HLA-G
FGFBP2
SPARC
DLL4
GDPD3
TRIM59
SLC2A1
C16orf74
LILRA2
SPATA17
MBOAT2
SYTL3
HOXA4
GAPT
ANXA4
GRAP
BUB1B
PROM1
ALDH1A2
FAM71E1
DIAPH2
CCR6
LCP1
ADAMTSL2
HLA-DPB2
SIX1
TMEM27
SPHK2

CDON
SPINK1
SEZ6L2
MYH10
NEFL
PHGDH
MYEOV
KRT222
ATP2C2
PACRG
AKR1B10
RAB37
MYO1F
COL4A1
ECHDC3
GRAMD1C
CD96
NFE2L3
CCDC109B
DPT
ARHGEF26
MYO1G
CD48
DACT2
WLS
GRAMD4
AOAH
LSP1
BICD1
GIMAP4
CSF1R
EMR1
GAL3ST4
IL7
INPP5D
SLC9A9
EMB
PLLP
HTATSF1P2
TGFBR3
MMRN1
MCTP1
RBP2

NMB
WDR63
SLC25A33
SDSL
RGS9BP
LIN52
SCUBE3
GALNT3
MAGI3
CECR1
PDLIM1
CCDC88A
OAS2
WFDC2
TPRG1
PPP1R1B
CNTD1
CLDN14
ITPR3
TMSB10
LRRIQ3
LAYN
ELF4
CD93
PDZK1
FAM184A
ETS1
SH2B3
FOXJ3
HOXB6
SRD5A2
SIRPA
SLC10A2
KCNE3
HDHD3
RAB7B
KANK4
OIP5
JAG2
HLA-DPA1
MTFP1
LOC145837
PLAT

PON3
NECAB1
CAMK1D
S100A14
LINC00472
ERO1L
FAM46B
GABARAPL1
ST8SIA1
PLXND1
LCN12
CYP39A1
C6orf223
FAM171A1
HSPB8
TCHH
ACSS3
KDELC1
44623
TNFRSF1B
C21orf62
LDLRAD3
ARHGAP11A
MLKL
DPY19L2P2
SASH3
PLXNC1
BTN3A1
PGAM2
COL9A3
ARPC1B
ELMO1
SIGLEC9
HIGD1A
WBSCR17
LY6E
DEGS1
P2RY13
CCNA2
RNF125
SLC1A4
SEMA6A
TLR1

BCKDHB
MET
OCLN
HLA-E
MST1
RNF166
FLT1
SLC39A14
PXDN
ZMYND12
RNF149
HACL1
SYT1
ABCA9
APCDD1L
AUH
GLRX5
CGREF1
IL2RG
XK
CIB4
DOK3
FANCI
FGR
LCN2
CDKN2A
RHBDF2
RASSF5
KIF18B
GXYLT2
SLC19A2
TCF19
PDP2
KCNS3
PTPRO
ATAD2
TNFAIP8L2
CD3G
MNX1
STMN3
EIF4EBP1
INSIG2
ERAP2

HINT2
ATP12A
MAP4K4
PTTG3P
HOXD8
TGM2
PPP2R2B
BEX2
C15orf59
CCL21
MARCO
NLRC3
ST3GAL6
TAPBP
IL32
C11orf92
MKI67
ST6GALNAC3
HLA-DOA
ENO1-AS1
CLEC2D
TNFRSF11A
ADAP2
TBC1D10C
CD27
SLC12A6
SAMD3
MDM2
CA2
TPD52L1
CENPH
C2orf15
KCNJ16
AASS
SP140L
KIAA1522
ASAP3
C21orf33
ASF1B
NCAPG
TAGLN2
OGN
TRIM2

ASAP1
GMIP
SLCO2B1
PRKAR2B
TRAF3IP2
MAN2B1
RAB11FIP4
PODXL
STOX1
MAGI2
DOK2
BTC
FASLG
DGKD
RBPMS2
HCG4
NOG
LOC344887
TRIM6
KCNN3
PLAG1
LEAP2
RASGEF1A
NTM
CKAP2L
C11orf85
SH3BGR
PHACTR3
MCM5
PCDHB11
HOXA7
NRL
C1QTNF7
BTBD16
LDHA
GSTZ1
PAQR4
B4GALNT1
ASPHD1
ABCC1
ERGIC1
STK39
LILRA1

EMX2
FECH
IL16
GRHPR
AICDA
CD40
KBTBD12
OMD
MDH1B
ADAMTS8
FRK
COL4A2
GCAT
DOCK4
AFMID
ALDOA
RGS20
ITGAM
SLC27A5
IL10RB
SPATA18
MAOA
KITLG
L2HGDH
CLDN2
FDXR
YEATS2
CLMN
PRSS22
NRP2
CACNA2D4
RHOBTB3
B3GNT5
LILRB5
PRR7
PTPN3
UBE2L6
JAKMIP1
CXCR6
UGT8
SLC16A2
PRR16
ERMP1

GLS2
LOC100506538
CATSPER1
ARNT2
PPP1R13L
ATP6V1A
CPAMD8
REN
MAD2L1
ARL10
PALM
GIMAP1
TMEM125
WIPF1
BAG2
PLIN4
CORO1C
CLIC3
C3orf70
VNN1
COQ10A
RIC3
LOC100131662
CYGB
DOPEY2
TBX3
ADRA2C
HLA-J
ADHFE1
NUPR1
NAP1L3
HOXB9
MFNG
NEFH
RASD2
CD163L1
LRRC4
THSD7A
LOC100289361
PTAFR
MRPS25
SLC16A1
SEMA3G

AJAP1
FAM198A
NPR3
KDM3A
REEP1
CDCA3
ADAMTSL4
C18orf54
PIP5K1B
CCDC148
MMP9
CBLN4
NR3C1
C1orf64
KIF11
AQP3
STK10
FAM83F
PLAUR
CD160
LRP4
PHLDA3
BLM
SETD7
FMNL1
GNA15
EMX2OS
SLC25A35
RAB15
MCM2
P4HB
HVCN1
CALCRL
AK3
HOXD1
PEBP4
RELT
CTGF
CDH5
BNIP3
GNA14
SLC41A2
WASF3

TMEM25
DNAJC28
PLEKHG4
UBE2C
GPR18
CRB2
MAMDC2
RNF180
PI16
FAM101B
MUC20
SLC48A1
SPAG5-AS1
ELK3
ZNF165
COL9A2
