# Supplementary material for: Identification and Validation of a Novel Ferroptotic Prognostic Genes-Based Signature of Clear Cell Renal Cell Carcinoma
Source: Cancers (Basel). 2022 Sep 27;14(19):4690. doi: 10.3390/cancers14194690 (PMC9562262; doi:10.3390/cancers14194690)
Supplement: Supplementary file 1 [file cancers-14-04690-s001.zip › Table S3 List of genes from FerrDb V2 database.pdf]

Table S3 List of genes from FerrDb V2 database.

symbol	hgncid	testin	confidence	uniprotac	pmid	latestupdate	datasource
RPL8	HGNC:10368	Human	Validated	P62917	22632970	2020/12/31	FerrDb team
IREB2	HGNC:6115	Human	Validated	P48200	22632970	2020/12/31	FerrDb team
ATP5MC3	HGNC:843	Human	Validated	P48201	22632970	2020/12/31	FerrDb team
CS	HGNC:2422	Human	Validated	O75390	22632970	2020/12/31	FerrDb team
EMC2	HGNC:28963	Human	Validated	Q15006	22632970	2020/12/31	FerrDb team
ACSF2	HGNC:26101	Human	Validated	Q96CM8	22632970	2020/12/31	FerrDb team
NOX1	HGNC:7889	Human	Deduced	Q9Y5S8	22632970	2020/12/31	FerrDb team
CYBB	HGNC:2578	Human	Deduced	P04839	22632970	2020/12/31	FerrDb team
NOX3	HGNC:7890	Human	Deduced	Q9HBY0	22632970	2020/12/31	FerrDb team
NOX4	HGNC:7891	Human	Deduced	Q9NPH5	22632970	2020/12/31	FerrDb team
NOX5	HGNC:14874	Human	Deduced	Q96PH1	22632970	2020/12/31	FerrDb team
DUOX1	HGNC:3062	Human	Deduced	Q9NRD9	22632970	2020/12/31	FerrDb team
DUOX2	HGNC:13273	Human	Deduced	Q9NRD8	22632970	2020/12/31	FerrDb team
G6PD	HGNC:4057	Human	Validated	P11413	22632970	2020/12/31	FerrDb team
PGD	HGNC:8891	Human	Validated	P52209	22632970	2020/12/31	FerrDb team
VDAC2	HGNC:12672	Human	Validated	P45880	22632970	2020/12/31	FerrDb team
PIK3CA	HGNC:8975	Mice	Validated	P42336	24739485	2020/12/31	FerrDb team
FLT3	HGNC:3765	Mice	Validated	P36888	24739485	2020/12/31	FerrDb team
SCP2	HGNC:10606	Mice	Validated	P22307	25402683	2020/12/31	FerrDb team
TP53	HGNC:11998	Human, mice	Validated	P04637	25799988	2020/12/31	FerrDb team
ACSL4	HGNC:3571	Human	Predicted	O60488	25965523	2020/12/31	FerrDb team
LPCAT3	HGNC:30244	Human	Predicted	Q6P1A2	25965523	2020/12/31	FerrDb team
NRAS	HGNC:7989	Human	Deduced	P01111	26157704	2020/12/31	FerrDb team

KRAS	HGNC:6407	Human	Deduced	P01116	26157704	2020/12/31	FerrDb team
HRAS	HGNC:5173	Human	Deduced	P01112	26157704	2020/12/31	FerrDb team
TF	HGNC:11740	Mice	Validated	P02787	26166707	2020/12/31	FerrDb team
TFRC	HGNC:11763	Mice	Validated	P02786	26166707	2020/12/31	FerrDb team
TFR2	HGNC:11762	Mice	Validated	Q9UP52	26166707	2020/12/31	FerrDb team
SLC38A1	HGNC:13447	Mice	Validated	Q9H2H9	26166707	2020/12/31	FerrDb team
SLC1A5	HGNC:10943	Mice	Validated	Q15758	26166707	2020/12/31	FerrDb team
GLS2	HGNC:29570	Mice	Validated	Q9UI32	26166707	2020/12/31	FerrDb team
GOT1	HGNC:4432	Mice	Validated	P17174	26166707	2020/12/31	FerrDb team
CARS1	HGNC:1493	Human, rat	Validated	P49589	26184909	2020/12/31	FerrDb team
ALOX5	HGNC:435	Mice	Validated	P09917	26235588	2020/12/31	FerrDb team
KEAP1	HGNC:23177	Human, mice	Validated	Q14145	26403645	2020/12/31	FerrDb team
HMOX1	HGNC:5013	Human, mice	Validated	P09601	26405158	2020/12/31	FerrDb team
ATG5	HGNC:589	Human, mice	Validated	Q9H1Y0	27245739	2020/12/31	FerrDb team
ATG7	HGNC:16935	Human, mice	Validated	O95352	27245739	2020/12/31	FerrDb team
NCOA4	HGNC:7671	Human	Validated	Q13772	27245739	2020/12/31	FerrDb team
ALOX12	HGNC:429	Human	Validated	P18054	27506793	2020/12/31	FerrDb team
ALOX12B	HGNC:430	Human	Validated	O75342	27506793	2020/12/31	FerrDb team
ALOX15	HGNC:433	Human	Validated	P16050	27506793	2020/12/31	FerrDb team
ALOX15B	HGNC:434	Human	Validated	O15296	27506793	2020/12/31	FerrDb team
ALOXE3	HGNC:13743	Human	Validated	Q9BYJ1	27506793	2020/12/31	FerrDb team
PHKG2	HGNC:8931	Human	Validated	P15735	27506793	2020/12/31	FerrDb team
ACO1	HGNC:117	Mice	Screened	P21399	27514700	2020/12/31	FerrDb team
ULK1	HGNC:12558	Mice	Validated	O75385	27514700	2020/12/31	FerrDb team
ATG3	HGNC:20962	Mice	Validated	Q9NT62	27514700	2020/12/31	FerrDb team

ATG4D	HGNC:20789	Mice	Screened	Q86TL0	27514700	2020/12/31	FerrDb team
BECN1	HGNC:1034	Mice	Screened	Q14457	27514700	2020/12/31	FerrDb team
MAP1LC3A	HGNC:6838	Mice	Screened	Q9H492	27514700	2020/12/31	FerrDb team
GABARAPL 2	HGNC:13291	Mice	Screened	P60520	27514700	2020/12/31	FerrDb team
GABARAPL 1	HGNC:4068	Mice	Screened	Q9H0R8	27514700	2020/12/31	FerrDb team
ATG16L1	HGNC:21498	Mice	Screened	Q676U5	27514700	2020/12/31	FerrDb team
WIPI1	HGNC:25471	Mice	Screened	Q5MNZ9	27514700	2020/12/31	FerrDb team
WIPI2	HGNC:32225	Mice	Screened	Q9Y4P8	27514700	2020/12/31	FerrDb team
SNX4	HGNC:11175	Mice	Screened	O95219	27514700	2020/12/31	FerrDb team
ATG13	HGNC:29091	Mice	Validated	O75143	27514700	2020/12/31	FerrDb team
ULK2	HGNC:13480	Mice	Validated	Q8IYT8	27514700	2020/12/31	FerrDb team
SAT1	HGNC:10540	Human, mice	Validated	P21673	27698118	2020/12/31	FerrDb team
EGFR	HGNC:3236	Human	Validated	P00533	28297659	2020/12/31	FerrDb team
MAPK3	HGNC:6877	Human	Validated	P27361	28297659	2020/12/31	FerrDb team
MAPK1	HGNC:6871	Human	Validated	P28482	28297659	2020/12/31	FerrDb team
BID	HGNC:1050	Mice	Validated	P55957	28384611	2020/12/31	FerrDb team
ZEB1	HGNC:11642	Human	Validated	P37275	28678785	2020/12/31	FerrDb team
DPP4	HGNC:3009	Human, mice	Validated	P27487	28813679	2020/12/31	FerrDb team
CDKN2A	HGNC:1787	Human, mice	Validated	P42771	28985506	2020/12/31	FerrDb team
PEBP1	HGNC:8630	Human, mice	Validated	P30086	29053969	2020/12/31	FerrDb team
SOCS1	HGNC:19383	Human	Validated	O15524	29081404	2020/12/31	FerrDb team
CDO1	HGNC:1795	Human	Validated	Q16878	29144989	2020/12/31	FerrDb team
MYB	HGNC:7545	Human	Validated	P10242	29144989	2020/12/31	FerrDb team

MAPK8	HGNC:6881	Rat	Validated	P45983	29330409	2020/12/31	FerrDb team
MAPK9	HGNC:6886	Rat	Validated	P45984	29330409	2020/12/31	FerrDb team
CHAC1	HGNC:28680	Human	Validated	Q9BUX1	29383104	2020/12/31	FerrDb team
MAPK14	HGNC:6876	Mice	Validated	Q16539	29436589	2020/12/31	FerrDb team
LINC00472	HGNC:21380	Human	Validated	Q9H8W2	29588351	2020/12/31	FerrDb team
PRKAA2	HGNC:9377	Human	Validated	P54646	30057310	2020/12/31	FerrDb team
PRKAA1	HGNC:9376	Human	Validated	Q13131	30057310	2020/12/31	FerrDb team
ELAVL1	HGNC:3312	Human, mice	Validated	Q15717	30081711	2020/12/31	FerrDb team
BAP1	HGNC:950	Human	Validated	Q92560	30202049	2020/12/31	FerrDb team
ABCC1	HGNC:51	Human	Validated	P33527	30726737	2020/12/31	FerrDb team
MIR6852	HGNC:49993	Human	Validated	_NA_	30787392	2020/12/31	FerrDb team
ACVR1B	HGNC:172	Human	Validated	P36896	30804470	2020/12/31	FerrDb team
TGFBR1	HGNC:11772	Human	Validated	P36897	30804470	2020/12/31	FerrDb team
EPAS1	HGNC:3374	Mice	Validated	Q99814	30962421	2020/12/31	FerrDb team
HILPDA	HGNC:28859	Mice	Validated	Q9Y5L2	30962421	2020/12/31	FerrDb team
HIF1A	HGNC:4910	Mice	Validated	Q16665	30962421	2020/12/31	FerrDb team
IFNG	HGNC:5438	Human, mice	Validated	P01579	31043744	2020/12/31	FerrDb team
ANO6	HGNC:25240	Human, mice	Validated	Q4KMQ2	31060306	2020/12/31	FerrDb team
LPIN1	HGNC:13345	Mice	Validated	Q14693	31061954	2020/12/31	FerrDb team
HMGB1	HGNC:4983	Human, mice	Validated	P09429	31105999	2020/12/31	FerrDb team
TNFAIP3	HGNC:11896	Human	Validated	P21580	31160087	2020/12/31	FerrDb team
TLR4	HGNC:11850	Rat	Validated	O00206	31196626	2020/12/31	FerrDb team
ATF3	HGNC:785	Human	Validated	P18847	31273299	2020/12/31	FerrDb team
ATM	HGNC:795	Human	Validated	Q13315	31320750	2020/12/31	FerrDb team
YY1AP1	HGNC:30935	Human, mice	Validated	Q9H869	31341276	2020/12/31	FerrDb team

EGLN2	HGNC:14660	Human, mice	Validated	Q96KS0	31355331	2020/12/31	FerrDb team
MIOX	HGNC:14522	Human, mice	Validated	Q9UGB7	31437128	2020/12/31	FerrDb team
TAZ	HGNC:11577	Human, mice	Validated	Q16635	31484063	2020/12/31	FerrDb team
MTDH	HGNC:29608	Human, mice	Validated	Q86UE4	31527591	2020/12/31	FerrDb team
IDH1	HGNC:5382	Human	Validated	O75874	31591388	2020/12/31	FerrDb team
SIRT1	HGNC:14929	Mice	Predicted	Q96EB6	31610175	2020/12/31	FerrDb team
PANX1	HGNC:8599	Human, mice	Validated	Q96RD7	31694915	2020/12/31	FerrDb team
DNAJB6	HGNC:14888	Human, mice	Predicted	O75190	31701262	2020/12/31	FerrDb team
BACH1	HGNC:935	Mice	Validated	O14867	31740582	2020/12/31	FerrDb team
LONP1	HGNC:9479	Human	Validated	P36776	31822343	2021/4/11	FerrDb team
CD82	HGNC:6210	Human	Validated		33655331	2021/4/16	FerrDb team
IL1B	HGNC:5992	Human, mice	Validated	_NA_	33376672	2021/5/17	FerrDb team
CTSB	HGNC:2527	_NA_	Validated	_NA_	33340545	2021/5/18	FerrDb team
POR	HGNC:9208	Human, mice	Validated	_NA_	33321093	2021/7/14	FerrDb team
CYB5R1	HGNC:13397	Human	Validated	_NA_	33321093	2021/7/14	FerrDb team
ELOVL5	HGNC:21308	Human	Validated	_NA_	33288688	2021/7/21	FerrDb team
FADS1	HGNC:3574	Human	Validated	_NA_	33288688	2021/7/21	FerrDb team
FBW7	HGNC:16712	Human	Validated	_NA_	33271455	2021/8/2	FerrDb team
PTEN	HGNC:9588	Human	Deduced	_NA_	33229547	2021/8/2	FerrDb team
NR1D1	HGNC:7962	Mice	Deduced	_NA_	33068011	2021/8/4	FerrDb team
NR1D2	HGNC:7963	Mice	Deduced	_NA_	33068011	2021/8/4	FerrDb team
TBK1	HGNC:11584	Mice	Validated	_NA_	33312375	2021/8/9	FerrDb team
IL6	HGNC:6018	Human, mice	Validated	_NA_	33166496	2021/8/11	FerrDb team
USP7	HGNC:12630	Rat	Validated	_NA_	33157209	2021/8/21	FerrDb team
ATF4	HGNC:786	Human, mice	Validated	_NA_	33055209	2021/8/30	FerrDb team

AQP3	HGNC:636	_NA_	Deduced	_NA_	33017631	2021/9/1	FerrDb team
AQP5	HGNC:638	_NA_	Deduced	_NA_	33017631	2021/9/1	FerrDb team
AQP8	HGNC:642	_NA_	Deduced	_NA_	33017631	2021/9/1	FerrDb team
LINC00618	HGNC:20110	Human, mice	Validated	_NA_	33002417	2021/9/8	FerrDb team
MT1DP	HGNC:7396	Human	Validated	_NA_	32929075	2021/9/15	FerrDb team
PEX10	HGNC:8851	Human, mice	Validated	_NA_	32939090	2021/9/26	FerrDb team
AGPAT3	HGNC:326	Human	Screened	_NA_	32939090	2021/9/26	FerrDb team
PEX12	HGNC:8854	Human	Validated	_NA_	32939090	2021/9/26	FerrDb team
CHP1	HGNC:17433	Human	Screened	_NA_	32939090	2021/9/26	FerrDb team
GPAT4	HGNC:20880	Human	Screened	_NA_	32939090	2021/9/26	FerrDb team
BRPF1	HGNC:14255	Human	Screened	_NA_	32939090	2021/9/26	FerrDb team
OSBPL9	HGNC:16386	Human	Screened	_NA_	32939090	2021/9/26	FerrDb team
INTS2	HGNC:29241	Human	Screened	_NA_	32939090	2021/9/26	FerrDb team
MMD	HGNC:7153	Human	Screened	_NA_	32939090	2021/9/26	FerrDb team
CYP4F8	HGNC:2648	Human	Screened	_NA_	32939090	2021/9/26	FerrDb team
MLLT1	HGNC:7134	Human	Screened	_NA_	32939090	2021/9/26	FerrDb team
TTPA	HGNC:12404	Human	Screened	_NA_	32939090	2021/9/26	FerrDb team
GRIA3	HGNC:4573	Human	Screened	_NA_	32939090	2021/9/26	FerrDb team
POM121L12	HGNC:25369	Human	Screened	_NA_	32939090	2021/9/26	FerrDb team
LIG3	HGNC:6600	Human, mice	Screened	_NA_	32939090	2021/9/26	FerrDb team
AEBP2	HGNC:24051	Human	Screened	_NA_	32939090	2021/9/26	FerrDb team
AGPS	HGNC:327	Human	Validated	_NA_	32939090	2021/9/26	FerrDb team
CDCA3	HGNC:14624	Human	Screened	_NA_	32939090	2021/9/26	FerrDb team
PEX2	HGNC:9717	Human	Validated	_NA_	32939090	2021/9/26	FerrDb team
PEX6	HGNC:8859	Human	Screened	_NA_	32939090	2021/9/26	FerrDb team

TIMM9	HGNC:11819	Human	Screened	_NA_	32939090	2021/9/26	FerrDb team
DCAF7	HGNC:30915	Human	Screened	_NA_	32939090	2021/9/26	FerrDb team
LCE2C	HGNC:29460	Human	Screened	_NA_	32939090	2021/9/26	FerrDb team
FAR1	HGNC:26222	Human	Validated	_NA_	32939090	2021/9/26	FerrDb team
PHF21A	HGNC:24156	Human	Screened	_NA_	32939090	2021/9/26	FerrDb team
SMAD7	HGNC:6773	Human	Screened	_NA_	32939090	2021/9/26	FerrDb team
LYRM1	HGNC:25074	Human	Screened	_NA_	32939090	2021/9/26	FerrDb team
AMN	HGNC:14604	Human	Screened	_NA_	32939090	2021/9/26	FerrDb team
PEX3	HGNC:8858	Human	Validated	_NA_	32939090	2021/9/26	FerrDb team
MTCH1	HGNC:17586	Human	Screened	_NA_	32939090	2021/9/26	FerrDb team
ACADSB	HGNC:91	Human	Deduced	_NA_	32776663	2021/10/11	FerrDb team
PVT1	HGNC:9709	Human, mice	Validated	_NA_	32827544	2021/10/13	FerrDb team
SLC39A14	HGNC:20858	Mice	Validated	_NA_	32374849	2021/10/17	FerrDb team
MAP3K11	HGNC:6850	Mice	Validated	Q16584	32710001	2021/11/5	FerrDb team
GSK3B	HGNC:4617	Human	Validated	P49841	32642794	2021/11/11	FerrDb team
BRD7	HGNC:14310	Human, mice	Validated	Q9NPI1	32863216	2021/11/18	FerrDb team
SLC25A28	HGNC:23472	Human, mice	Validated	Q96A46	32863216	2021/11/18	FerrDb team
MFN2	HGNC:16877	Rat	Validated	O95140	32593899	2021/11/18	FerrDb team
SLC11A2	HGNC:10908	Human	Validated	P49281	32535745	2021/11/21	FerrDb team
ZFAS1	HGNC:33101	Human, rat	Validated	_NA_	32453709	2021/12/1	FerrDb team
TSC1	HGNC:12362	Human, mice	Validated	Q92574	32404875	2021/12/2	FerrDb team
TGFB1	HGNC:11766	Human	Deduced	P01137	32471991	2021/12/3	FerrDb team
SNCA	HGNC:11138	Human	Validated	P37840	32341450	2021/12/9	FerrDb team
SIRT3	HGNC:14931	Human, porcine	Validated	Q9NTG7	32329068	2021/12/9	FerrDb team

CGAS	HGNC:21367	Human	Validated	Q8N884	32186434	2021/12/19	FerrDb team
STING1	HGNC:27962	Human	Validated	Q86WV6	32186434	2021/12/19	FerrDb team
HDDC3	HGNC:30522	Human	Validated	Q8N4P3	32462112	2021/12/22	FerrDb team
MIR761	HGNC:37305	Human	Validated	_NA_	32196629	2021/12/24	FerrDb team
MDM2	HGNC:6973	Human	Validated	Q00987	32079652	2021/12/30	FerrDb team
MDM4	HGNC:6974	Human	Validated	O15151	32079652	2021/12/30	FerrDb team
MIR214	HGNC:31591	Human	Validated	_NA_	31960438	2021/12/30	FerrDb team
DLD	HGNC:2898	Human	Validated	P09622	31931284	2021/12/30	FerrDb team
WWTR1	HGNC:24042	Human	Validated	Q9GZV5	31641008	2022/1/10	FerrDb team
PRKCA	HGNC:9393	Human	Validated	P17252	31173656	2022/1/10	FerrDb team
LGMN	HGNC:9472	Mice	Validated	Q99538	33431801	2022/1/19	FerrDb team
SMPD1	HGNC:11120	Human	Validated	P17405	33414455	2022/1/26	FerrDb team
MYCN	HGNC:7559	Human	Validated	P04198	34011924	2022/2/10	FerrDb team
IFNA1	HGNC:5417	Human, mice	Deduced	P01562	34385713	2022/2/10	FerrDb team
IFNA2	HGNC:5423	Human, mice	Deduced	P01563	34385713	2022/2/10	FerrDb team
IFNA4	HGNC:5425	Human, mice	Deduced	P05014	34385713	2022/2/10	FerrDb team
IFNA5	HGNC:5426	Human, mice	Deduced	P01569	34385713	2022/2/10	FerrDb team
IFNA6	HGNC:5427	Human, mice	Deduced	P05013	34385713	2022/2/10	FerrDb team
IFNA7	HGNC:5428	Human, mice	Deduced	P01567	34385713	2022/2/10	FerrDb team
IFNA8	HGNC:5429	Human, mice	Deduced	P32881	34385713	2022/2/10	FerrDb team
IFNA10	HGNC:5418	Human, mice	Deduced	P01566	34385713	2022/2/10	FerrDb team
IFNA13	HGNC:5419	Human, mice	Deduced	P01562	34385713	2022/2/10	FerrDb team
IFNA14	HGNC:5420	Human, mice	Deduced	P01570	34385713	2022/2/10	FerrDb team
IFNA16	HGNC:5421	Human, mice	Deduced	P05015	34385713	2022/2/10	FerrDb team
IFNA17	HGNC:5422	Human, mice	Deduced	P01571	34385713	2022/2/10	FerrDb team

IFNA21	HGNC:5424	Human, mice	Deduced	P01568	34385713	2022/2/10	FerrDb team
SMG9	HGNC:25763	Human	Validated	Q9H0W8	34146907	2022/2/23	FerrDb team
PPARG	HGNC:9236	Mice	Validated	P37231	34478917	2022/2/24	FerrDb team
miR-335	HGNC:31773	Rat	Validated	_NA_	33649797	2022/2/24	FerrDb team
SNX5	HGNC:14969	Rat	Validated	Q9Y5X3	34134000	2022/3/3	FerrDb team
PAQR3	HGNC:30130	Human	Validated	Q6TCH7	33955706	2022/3/9	FerrDb team
MICU1	HGNC:1530	Human	Validated	Q9BPX6	33822458	2022/3/9	FerrDb team
TOR2A	HGNC:11996	Human	Predicted	Q5JU69	34296310	2022/3/11	FerrDb team
MIR375	HGNC:31868	Human	Validate	_NA_	34090492	2022/3/15	FerrDb team
MAP3K14	HGNC:6853	Mice	Validate	Q99558	34558831	2022/3/16	FerrDb team
MIR324	HGNC:31767	Human	Validated	_NA_	33662669	2022/3/21	FerrDb team
QSOX1	HGNC:9756	Human	Validated	O00391	33770521	2022/3/21	FerrDb team
MIB2	HGNC:30577	Mice	Validated	Q96AX9	34121396	2022/3/21	FerrDb team
CLTRN	HGNC:29437	Human	Validated	Q9HBJ8	33508374	2022/3/21	FerrDb team
KLF2	HGNC:6347	Human	Validated	Q9Y5W3	34520818	2022/3/21	FerrDb team
MIR5096	HGNC:41611	Human	Validated	_NA_	34571083	2022/3/21	FerrDb team
HOTAIR	HGNC:33510	Rat	Validated	_NA_	33906483	2022/3/21	FerrDb team
H19	HGNC:4713	Human	Validated	_NA_	34288826	2022/3/21	FerrDb team
FOXO4	HGNC:7139	Rat	Deduced	P98177	34296293	2022/3/22	FerrDb team
YTHDC2	HGNC:24721	Human	Validated	Q9H6S0	33785413	2022/3/30	FerrDb team
DDR2	HGNC:2731	Human	Validated	Q16832	33603168	2022/3/30	FerrDb team
SLC39A7	HGNC:4927	Human	Validated	Q92504	33608508	2022/3/30	FerrDb team
TRIM46	HGNC:19019	Human	Validated	Q7Z4K8	34487731	2022/3/30	FerrDb team
ACSL1	HGNC:3569	Human	Validated	P33121	33854057	2022/3/30	FerrDb team
KDM5A	HGNC:9886	Human	Validated	P29375	33741422	2022/3/30	FerrDb team

TRIM21	HGNC:11312	Mice	Validated	P19474	34233258	2022/3/30	FerrDb team
DPEP1	HGNC:3002	Mice	Validated	P16444	34426578	2022/3/30	FerrDb team
CYGB	HGNC:16505	Human	Validated	Q8WWM9	33611811	2022/3/30	FerrDb team
IDO1	HGNC:6059	Mice	Validated	P14902	33899121	2022/3/30	FerrDb team
GSTZ1	HGNC:4643	Human	Validated	O43708	33931597	2022/3/30	FerrDb team
GJA1	HGNC:4274	Human, mice	Validated	P17302	34785303	2022/4/6	FerrDb team
SLC7A11	HGNC:11059	Human	Validated	Q9UPY5	34722314	2022/4/6	FerrDb team
PGRMC1	HGNC:16090	Mice	Validated	O00264	34749765	2022/4/7	FerrDb team
CIRBP	HGNC:1982	Human	Validated	Q14011	34114349	2022/4/7	FerrDb team
USP11	HGNC:12609	Mice	Validated	P51784	34839355	2022/4/7	FerrDb team
YAP	HGNC:16262	Human	Validated	P46937	34977009	2022/4/7	FerrDb team
MIR135B	HGNC:31760	Rat	Validated	_NA_	34485394	2022/4/8	FerrDb team
TRIM26	HGNC:12962	Human	Validated	Q12899	33869196	2022/4/8	FerrDb team
NDRG1	HGNC:7679	Human	Deduced	Q92597	34917147	2022/4/8	FerrDb team
MIR302A	HGNC:31623	Human	Validated	_NA_	34181495	2022/4/8	FerrDb team
ASMTL-AS1	HGNC:25811	Human	Validated	_NA_	34658100	2022/4/11	FerrDb team
FADS2	HGNC:3575	Human	Validated	O95864	34520742	2022/4/12	FerrDb team
PIEZO1	HGNC:28993	Human, mice	Validated	Q92508	34568428	2022/4/12	FerrDb team
LIFR	HGNC:6597	Human	Validated	P42702	34921145	2022/4/12	FerrDb team
PTPN6	HGNC:9658	Human	Validated	P29350	34921145	2022/4/12	FerrDb team
MIR15A	HGNC:31543	Mice	Validated	_NA_	34339707	2022/4/17	FerrDb team
EGR1	HGNC:3238	Mice	Validated	P18146	34339707	2022/4/17	FerrDb team
ADAM23	HGNC:202	Human	Validated	O75077	35003396	2022/4/17	FerrDb team
ARHGEF26-AS1	HGNC:41048	Human	Validated	_NA_	35003396	2022/4/17	FerrDb team

CPEB1	HGNC:21744	Human	Validated	Q9BZB8	34184391	2022/4/18	FerrDb team
COX4I2	HGNC:16232	Rat	Validated	Q96KJ9	34026834	2022/4/18	FerrDb team
TIMP1	HGNC:11820	Rat	Validated	P01033	34938604	2022/4/25	FerrDb team
KDM6B	HGNC:29012	Human, mice	Validated	O15054	34542160	2022/4/25	FerrDb team
METTL14	HGNC:29330	Human, rat	Validated	Q9HCE5	34648132	2022/5/5	FerrDb team
MIB1	HGNC:21086	Human	Validated	Q86YT6	34670864	2022/5/5	FerrDb team
KDM5C	HGNC:11114	Human, mice	Validated	P41229	34522206	2022/5/9	FerrDb team
MEG3	HGNC:14575	Mice	Validated	_NA_	34587716	2022/5/11	FerrDb team
CCDC6	HGNC:18782	Human, mice	Validated	Q16204	34841108	2022/5/11	FerrDb team
CFL1	HGNC:1874	Mice	Validated	P23528	34657120	2022/5/16	FerrDb team
PTGS2	HGNC:9605	Human	Validated	P35354	24439385	2022/3/9	FerrDb team
SLC40A1	HGNC:10909	Mice	Validated	Q9NP59	27773819	2022/3/9	FerrDb team
FTH1	HGNC:3976	Mice	Validated	P02794	27773819	2022/3/9	FerrDb team
GPX4	HGNC:4556	Mice	Validated	P36969	27773819	2022/3/9	FerrDb team
HSPB1	HGNC:5246	Mice	Validated	P04792	27773819	2022/3/9	FerrDb team
NFE2L2	HGNC:7782	Mice	Validated	Q16236	27773819	2022/3/9	FerrDb team
AKR1C1	HGNC:384	Human	Validated	Q04828	24844246	2020/12/31	FerrDb team
AKR1C2	HGNC:385	Human	Validated	P52895	24844246	2020/12/31	FerrDb team
AKR1C3	HGNC:386	Human	Validated	P42330	24844246	2020/12/31	FerrDb team
RB1	HGNC:9884	Human, mice	Validated	P06400	25444922	2020/12/31	FerrDb team
HSF1	HGNC:5224	Human, mice	Validated	Q00613	25728673	2020/12/31	FerrDb team
GCLC	HGNC:4311	Mice	Validated	P48506	26166707	2020/12/31	FerrDb team
SQSTM1	HGNC:11280	Human, mice	Validated	Q13501	26403645	2020/12/31	FerrDb team
NQO1	HGNC:2874	Human, mice	Validated	P15559	26403645	2020/12/31	FerrDb team
MUC1	HGNC:7508	Human	Validated	P15941	26930718	2020/12/31	FerrDb team

SLC3A2	HGNC:11026	Mice	Validated	P08195	26945935	2020/12/31	FerrDb team
MT1G	HGNC:7399	Human, mice	Validated	P13640	27015352	2020/12/31	FerrDb team
CISD1	HGNC:30880	Human	Validated	Q9NZ45	27510639	2020/12/31	FerrDb team
FANCD2	HGNC:3585	Mice	Validated	Q9BXW9	27773819	2020/12/31	FerrDb team
FTMT	HGNC:17345	Mice, fly	Validated	Q8N4E7	28066232	2020/12/31	FerrDb team
HSPA5	HGNC:5238	Human, mice	Validated	P11021	28130223	2020/12/31	FerrDb team
HELLS	HGNC:4861	Human, mice	Validated	Q9NRZ9	28900510	2020/12/31	FerrDb team
SCD	HGNC:10571	Human	Validated	O00767	28900510	2020/12/31	FerrDb team
SRC	HGNC:11283	Human	Validated	P12931	28972104	2020/12/31	FerrDb team
STAT3	HGNC:11364	Human	Validated	P40763	28972104	2020/12/31	FerrDb team
PML	HGNC:9113	Human	Validated	P29590	29081404	2020/12/31	FerrDb team
MTOR	HGNC:3942	Mice	Validated	P42345	29127238	2020/12/31	FerrDb team
NFS1	HGNC:15910	Human	Validated	Q9Y697	29168506	2020/12/31	FerrDb team
TP63	HGNC:15979	Human	Validated	Q9H3D4	29212036	2020/12/31	FerrDb team
CDKN1A	HGNC:1784	Human	Validated	P38936	29346757	2020/12/31	FerrDb team
MIR137	HGNC:31523	Human, mice	Validated	_NA_	29348676	2020/12/31	FerrDb team
ENPP2	HGNC:3357	Rat	Validated	Q13822	29551679	2020/12/31	FerrDb team
FH	HGNC:3700	Human	Validated	P07954	29917289	2020/12/31	FerrDb team
CISD2	HGNC:24212	Human	Validated	Q8N5K1	29928961	2020/12/31	FerrDb team
MIR9-1	HGNC:31641	Human	Validated	_NA_	30035324	2020/12/31	FerrDb team
MIR9-2	HGNC:31642	Human	Validated	_NA_	30035324	2020/12/31	FerrDb team
MIR9-3	HGNC:31646	Human	Validated	_NA_	30035324	2020/12/31	FerrDb team
CBS	HGNC:1550	Human, mice	Validated	P35520	30258181	2020/12/31	FerrDb team
ISCU	HGNC:29882	Human	Validated	Q9H1K1	30557609	2020/12/31	FerrDb team
ACSL3	HGNC:3570	Human	Validated	O95573	30686757	2020/12/31	FerrDb team

OTUB1	HGNC:23077	Human, mice	Validated	Q96FW1	30709928	2020/12/31	FerrDb team
CD44	HGNC:1681	Human	Validated	P16070	30709928	2020/12/31	FerrDb team
LINC00336	HGNC:33813	Human	Validated	Q6ZUF6	30787392	2020/12/31	FerrDb team
BRD4	HGNC:13575	Human	Validated	O60885	30988278	2020/12/31	FerrDb team
PRDX6	HGNC:16753	Human	Validated	P30041	31036877	2020/12/31	FerrDb team
MIR17	HGNC:31547	Human	Validated	_NA_	31160087	2020/12/31	FerrDb team
SESN2	HGNC:20746	Human, mice	Validated	P58004	31323261	2020/12/31	FerrDb team
NF2	HGNC:7773	Human, mice	Validated	P35240	31341276	2020/12/31	FerrDb team
ARNTL	HGNC:701	Human, mice	Validated	O00327	31355331	2020/12/31	FerrDb team
JUN	HGNC:6204	Human, mice	Deduced	P05412	31394193	2020/12/31	FerrDb team
CA9	HGNC:1383	Human	Validated	Q16790	31442913	2020/12/31	FerrDb team
TMBIM4	HGNC:24257	Human, mice	Validated	Q9HC24	31507082	2020/12/31	FerrDb team
PLIN2	HGNC:248	Human, mice	Screened	Q99541	31520166	2020/12/31	FerrDb team
MIR212	HGNC:31589	Mice	Validated	_NA_	31533781	2020/12/31	FerrDb team
AIFM2	HGNC:21411	Human, mice	Validated	Q9BRQ8	31634899	2020/12/31	FerrDb team
LAMP2	HGNC:6501	Human	Validated	P13473	31672277	2020/12/31	FerrDb team
ZFP36	HGNC:12862	Human, mice	Validated	P26651	31679460	2020/12/31	FerrDb team
PROM2	HGNC:20685	Human	Validated	Q8N271	31735663	2020/12/31	FerrDb team
CHMP5	HGNC:26942	Human, mice	Validated	Q9NZZ3	31761326	2020/12/31	FerrDb team
CHMP6	HGNC:25675	Human, mice	Validated	Q96FZ7	31761326	2020/12/31	FerrDb team
CAV1	HGNC:1527	Human, mice	Validated	Q03135	31877357	2020/12/31	FerrDb team
GCH1	HGNC:4193	Human	Validated	P30793	31989025	2021/5/15	FerrDb team
DAZAP1	HGNC:2683	_NA_	Validated	Q96EP5	33358859	2021/7/2	FerrDb team
PIR	HGNC:30048	Human, mice	Validated	O00625	33373853	2021/7/2	FerrDb team
FTL	HGNC:3999	Rat	Validated	P02792	33333054	2021/7/12	FerrDb team

HCAR1	HGNC:4532	Human, mice	Validated	Q9BXC0	33296645	2021/7/12	FerrDb team
SLC16A1	HGNC:10922	Human, mice	Validated	P53985	33296645	2021/7/16	FerrDb team
RRM2	HGNC:10452	Human	Validated	P31350	33372599	2021/7/19	FerrDb team
NR4A1	HGNC:7980	Human	Deduced	P22736	33271455	2021/8/2	FerrDb team
RPTOR	HGNC:30287	Human	Deduced	Q8N122	33229547	2021/8/2	FerrDb team
SREBF1	HGNC:11289	Human	Validated	P36956	33229547	2021/8/2	FerrDb team
SREBF2	HGNC:11290	Human	Validated	Q12772	33203734	2021/8/6	FerrDb team
FZD7	HGNC:4045	Human, mice	Validated	O75084	33172933	2021/8/9	FerrDb team
P4HB	HGNC:8548	Human	Deduced	P07237	33124817	2021/8/21	FerrDb team
NT5DC2	HGNC:25717	_NA_	Deduced	Q9H857	33124817	2021/8/21	FerrDb team
BCAT2	HGNC:977	Human	Validated	O15382	33097833	2021/8/23	FerrDb team
PLA2G6	HGNC:9039	Human, mice	Validated	O60733	33087576	2021/8/27	FerrDb team
MIR424	HGNC:31881	Human	Validated	_NA_	33038905	2021/8/30	FerrDb team
PARK7	HGNC:16369	Human, mice	Validated	Q99497	33024240	2021/9/1	FerrDb team
FXN	HGNC:3951	Human	Validated	Q16595	33017621	2021/9/3	FerrDb team
SUV39H1	HGNC:11479	Human	Validated	O43463	33643820	2021/9/3	FerrDb team
ATF2	HGNC:784	Human	Validated	P15336	33008584	2021/9/6	FerrDb team
ACOT1	HGNC:33128	Mice	Validated	Q86TX2	32934217	2021/9/15	FerrDb team
ALDH3A2	HGNC:403	Mice	Validated	P51648	32458004	2021/9/17	FerrDb team
STK11	HGNC:11389	Human	Validated	Q15831	32883948	2021/9/17	FerrDb team
FNDC5	HGNC:20240	Human, mice	Deduced	Q8NAU1	32997405	2021/9/26	FerrDb team
CDH1	HGNC:1748	Human, mice	Validated	P12830	32896720	2021/9/27	FerrDb team
NEDD4L	HGNC:7728	Human	Validated	Q96PU5	32811647	2021/10/14	FerrDb team
BRD2	HGNC:1103	Human	Deduced	P25440	32937365	2021/10/28	FerrDb team

BRD3	HGNC:1104	Human	Deduced	Q15059 聽<br><br>(BRD3_HUMA<br>N	32937365	2021/10/28	FerrDb team
BRDT	HGNC:1105	Human	Deduced	Q58F21	32937365	2021/10/28	FerrDb team
DECR1	HGNC:2753	Human	Validated	Q16698	32686647	2021/11/11	FerrDb team
GLRX5	HGNC:20134	Human, mice	Validated	Q86SX6	32685019	2021/11/20	FerrDb team
NCOA3	HGNC:7670	Human	Deduced	Q9Y6Q9	32536370	2021/11/26	FerrDb team
NR5A2	HGNC:7984	Human	Deduced	O00482	32536370	2021/11/26	FerrDb team
PANX2	HGNC:8600	Human	Validated	Q96RD6	32547072	2021/12/1	FerrDb team
RHEBP1	HGNC:10010	Human, mice	Validated	_NA_	32404875	2021/12/2	FerrDb team
TFAP2A	HGNC:11742	Human	Validated	P05549	32432738	2021/12/9	FerrDb team
CP	HGNC:2295	Human	Validated	P00450	32283255	2021/12/9	FerrDb team
ARF6	HGNC:659	Human	Validated	P62330	32368394	2021/12/16	FerrDb team
GDF15	HGNC:30142	Human	Validated	Q99988	32209255	2021/12/17	FerrDb team
ABHD12	HGNC:15868	Human	Validated	Q8N2K0	32195565	2021/12/17	FerrDb team
PPP1R13L	HGNC:18838	Mice	Validated	Q8WUF5	32203170	2021/12/19	FerrDb team
TFAM	HGNC:11741	Human	Validated	Q00059	32186434	2021/12/19	FerrDb team
KDM3B	HGNC:1337	Human	Validated	Q7LBC6	32107878	2021/12/20	FerrDb team
RNF113A	HGNC:12974	Human	Validated	O15541	32152280	2021/12/20	FerrDb team
AHCY	HGNC:343	Human	Deduced	P23526	32144268	2021/12/21	FerrDb team
circ-TTBK2	HGNC:19141	Human	Validated	Q6IQ55	32196629	2021/12/22	FerrDb team
MIR522	HGNC:32127	Human	Validated	_NA_	32106859	2021/12/22	FerrDb team
IDH2	HGNC:5383	Human, mice	Validated	P48735	32089268	2021/12/30	FerrDb team
PPARA	HGNC:9232	Human	Validated	Q07869	32079652	2021/12/30	FerrDb team

NOS2	HGNC:7873	Mice	Validated	P35228	32080625	2021/12/30	FerrDb team
SIAH2	HGNC:10858	Human	Validated	O43255	32042051	2021/12/30	FerrDb team
RELA	HGNC:9955	Mice	Validated	Q04206	32015337	2021/12/31	FerrDb team
VDR	HGNC:12679	Mice	Validated	P11473	31996668	2021/12/30	FerrDb team
NEDD4	HGNC:7727	Human	Validated	P46934	31974380	2021/12/30	FerrDb team
PRDX1	HGNC:9352	Human	Deduced	Q06830	31901729	2022/1/7	FerrDb team
AR	HGNC:644	Human	Validated	P10275	31896509	2022/1/7	FerrDb team
MTF1	HGNC:7428	Human	Validated	Q14872	31320750	2022/1/10	FerrDb team
COPZ1	HGNC:2243	Human	Validated	P61923	33420375	2022/1/19	FerrDb team
NUPR1	HGNC:29990	Human	Validated	O60356	33510144	2022/1/26	FerrDb team
USP35	HGNC:20061	Human	Validated	Q9P2H5	33931967	2022/1/26	FerrDb team
NEAT1	HGNC:30815	Human	Validated	_NA_	33730930	2022/2/24	FerrDb team
PARP1	HGNC:270	Human	Deduced	P09874	33722571	2022/3/2	FerrDb team
PARP2	HGNC:272	Human	Deduced	Q9UGN5	33722571	2022/3/2	FerrDb team
PARP3	HGNC:273	Human	Deduced	Q9Y6F1	33722571	2022/3/2	FerrDb team
PARP4	HGNC:271	Human	Deduced	Q9UKK3	33722571	2022/3/2	FerrDb team
PARP6	HGNC:26921	Human	Deduced	Q2NL67	33722571	2022/3/2	FerrDb team
PARP8	HGNC:26124	Human	Deduced	Q8N3A8	33722571	2022/3/2	FerrDb team
PARP9	HGNC:24118	Human	Deduced	Q8IXQ6	33722571	2022/3/2	FerrDb team
PARP10	HGNC:25895	Human	Deduced	Q53GL7	33722571	2022/3/2	FerrDb team
PARP11	HGNC:1186	Human	Deduced	Q9NR21	33722571	2022/3/2	FerrDb team
PARP12	HGNC:21919	Human	Deduced	Q9H0J9	33722571	2022/3/2	FerrDb team
PARP14	HGNC:29232	Human	Deduced	Q460N5	33722571	2022/3/2	FerrDb team
PARP15	HGNC:26876	Human	Deduced	Q460N3	33722571	2022/3/2	FerrDb team
PARP16	HGNC:26040	Human	Deduced	Q8N5Y8	33722571	2022/3/2	FerrDb team

PDSS2	HGNC:23041	Human	Validated	Q86YH6	33929387	2022/3/2	FerrDb team
TXN	HGNC:12435	Mice	Validated	P10599	33634378	2022/3/2	FerrDb team
SENP1	HGNC:17927	Rat	Validated	Q9P0U3	33746578	2022/3/3	FerrDb team
OIP5-AS1	HGNC:43563	Human	Validated	_NA_	34051661	2022/3/3	FerrDb team
MIR190A	HGNC:31560	Human	Validated	_NA_	34111670	2022/3/3	FerrDb team
FGF21	HGNC:3678	Mice	Validated	Q9NSA1	34530349	2022/3/7	FerrDb team
CREB1	HGNC:2345	Human	Deduced	P16220	33846793	2022/3/7	FerrDb team
CREB3	HGNC:2347	Human	Deduced	O43889	33846793	2022/3/7	FerrDb team
CREB5	HGNC:16844	Human	Deduced	Q02930	33846793	2022/3/7	FerrDb team
MIR130B	HGNC:31515	Human	Validated	_NA_	34117611	2022/3/10	FerrDb team
BEX1	HGNC:1036	Human	Validated	Q9HBH7	33745298	2022/3/10	FerrDb team
ASAH2	HGNC:18860	Mice	Validated	Q9NR71	33547170	2022/3/15	FerrDb team
FABP4	HGNC:3559	Human	Validated	P15090	34030117	2022/3/15	FerrDb team
AKT1S1	HGNC:28426	Human	Deduced	Q96B36	33707434	2022/3/15	FerrDb team
MLST8	HGNC:24825	Human	Deduced	Q9BVC4	33707434	2022/3/15	FerrDb team
TYRO3	HGNC:12446	Human, mice	Validated	Q06418	33855973	2022/3/16	FerrDb team
SIRT6	HGNC:14934	Human	Validated	Q8N6T7	34530350	2022/3/16	FerrDb team
TMSB4X	HGNC:11881	Human, rat	Deduced	P62328	34280397	2022/3/16	FerrDb team
TMSB4Y	HGNC:11882	Human, rat	Deduced	O14604	34280397	2022/3/16	FerrDb team
KIF20A	HGNC:9787	Human	Validated	O95235	33819186	2022/3/16	FerrDb team
ECH1	HGNC:3149	Human, mice	Validated	Q13011	33813878	2022/3/16	FerrDb team
ETV4	HGNC:3493	Human	Validated	P43268	34283663	2022/3/21	FerrDb team
MEG8	HGNC:14574	Human	Validated	_NA_	33839417	2022/3/21	FerrDb team
VCP	HGNC:12666	Human	Validated	P55072	34033175	2022/3/21	FerrDb team
RBMS1	HGNC:9907	Human	Validated	P29558	34609966	2022/3/30	FerrDb team

KDM4A	HGNC:22978	Human	Validated	O75164	33689883	2022/3/30	FerrDb team
MGST1	HGNC:7061	Human	Validated	P10620	33539732	2022/3/30	FerrDb team
MPC1	HGNC:21606	Human	Validated	Q9Y5U8	33741422	2022/3/30	FerrDb team
CHMP1A	HGNC:8740	Mice	Validated	Q9HD42	34426578	2022/3/30	FerrDb team
CAMKK2	HGNC:1470	Human	Validated	Q96RR4	34242660	2022/3/30	FerrDb team
SOX2	HGNC:11195	Human, mice	Validated	P48431	34385181	2022/3/31	FerrDb team
SRSF9	HGNC:10791	Human	Validated	Q13242	33609745	2022/3/31	FerrDb team
PROK2	HGNC:18455	Mice	Validated	Q9HC23	34244497	2022/3/31	FerrDb team
MIR4443	HGNC:41830	Human	Validated	_NA_	33781830	2022/4/6	FerrDb team
SIRT2	HGNC:10886	Mice	Validated	Q8IXJ6	34102645	2022/4/6	FerrDb team
MIR27A	HGNC:31613	Human	Validated	_NA_	34722314	2022/4/6	FerrDb team
MIR670	HGNC:37304	Human	Validated	_NA_	34323631	2022/4/7	FerrDb team
MEF2C	HGNC:6996	Human	Validated	Q06413	33984142	2022/4/7	FerrDb team
EZH2	HGNC:3527	Human	Validated	Q15910	34614259	2022/4/7	FerrDb team
PEDS1	HGNC:16735	Human	Validated	A5PLL7	33731874	2022/4/7	FerrDb team
ADAMTS13	HGNC:1366	Mice	Validated	Q76LX8	34666603	2022/4/8	FerrDb team
CDC25A	HGNC:1725	Human	Validated	P30304	34743185	2022/4/8	FerrDb team
PPARD	HGNC:9235	Mice	Validated	Q03181	34649350	2022/4/11	FerrDb team
ENO3	HGNC:3354	Mice	Validated	P13929	33987359	2022/4/12	FerrDb team
LCN2	HGNC:6526	Human	Validated	P80188	34921145	2022/4/12	FerrDb team
MARCHF5	HGNC:26025	Rat	Validated	Q9NX47	34390730	2022/4/17	FerrDb team
TRIB2	HGNC:30809	Human	Validated	Q92519	34315867	2022/4/17	FerrDb team
DHODH	HGNC:2867	Human	Validated	Q02127	33981038	2022/4/18	FerrDb team
MIR545	HGNC:32531	Human	Validated	_NA_	34954694	2022/4/18	FerrDb team
PDK4	HGNC:8812	Human	Validated	Q16654	33626342	2022/4/18	FerrDb team

MIR9-3HG	HGNC:27388	Mice	Validated	_NA_	34953631	2022/4/24	FerrDb team
ADIPOQ	HGNC:13633	Mice	Validated	Q15848	34859390	2022/4/25	FerrDb team
PTPN18	HGNC:9649	Human	Validated	Q99952	33642877	2022/5/5	FerrDb team
ABCC5	HGNC:56	Human	Validated	O15440	34768109	2022/5/11	FerrDb team
CISD3	HGNC:27578	Human	Validated	P0C7P0	34497268	2022/5/11	FerrDb team
MS4A15	HGNC:28573	Mice	Validated	Q8N5U1	34663908	2022/5/11	FerrDb team
FURIN	HGNC:8568	Human, mice	Deduced	P09958	33640301	2022/5/11	FerrDb team
GALNT14	HGNC:22946	Human	Validated	Q96FL9	34643088	2022/5/16	FerrDb team
KLHDC3	HGNC:20704	Human	Validated	Q9BQ90	34743205	2022/5/16	FerrDb team
