# Supplementary material for: Identification and Validation of a Novel Ferroptotic Prognostic Genes-Based Signature of Clear Cell Renal Cell Carcinoma
Source: Cancers (Basel). 2022 Sep 27;14(19):4690. doi: 10.3390/cancers14194690 (PMC9562262; doi:10.3390/cancers14194690)
Supplement: Supplementary file 1 [file cancers-14-04690-s001.zip › Table S4 List of DEGs from three TCGA database.pdf]

Table S4 List of DEGs from three TCGA database

gene_name	gene_id	gene_biotype	baseMean	log2FoldChange	lfcSE	stat	pvalue	padj
TSPAN6	ENSG00000000003	protein_coding	3475.62172	-1.2610769	0.07506917	-16.798864	2.4876E-63	9.4088E-62
CYP51A1	ENSG00000001630	protein_coding	218.048511	-1.1549351	0.12013032	-9.6140188	6.9771E-22	4.5162E-21
HECW1	ENSG00000002746	protein_coding	240.483871	-3.7260308	0.22427325	-16.613799	5.5372E-62	1.9893E-60
KLHL13	ENSG00000003096	protein_coding	963.861954	-2.1759814	0.11826271	-18.399556	1.3245E-75	7.8748E-74
CYP26B1	ENSG00000003137	protein_coding	557.075127	-1.6510987	0.14803281	-11.153599	6.8767E-29	6.4704E-28
HSPB6	ENSG00000004776	protein_coding	776.118121	-1.0334148	0.20962379	-4.9298543	8.2291E-07	1.8986E-06
SLC4A1	ENSG00000004939	protein_coding	3212.85211	-3.203467	0.40223074	-7.9642521	1.6623E-15	7.388E-15
PRSS22	ENSG00000005001	protein_coding	71.0558965	-3.2079894	0.3457506	-9.2783336	1.7215E-20	1.0327E-19
SLC25A5	ENSG00000005022	protein_coding	14443.0941	-1.1376125	0.12108368	-9.3952593	5.7077E-21	3.5092E-20
HOXA11	ENSG00000005073	protein_coding	132.757838	-1.43904	0.15954162	-9.0198409	1.8836E-19	1.0656E-18
THSD7A	ENSG00000005108	protein_coding	2576.47978	-1.6259675	0.16340926	-9.9502773	2.5148E-23	1.7525E-22
ACSM3	ENSG00000005187	protein_coding	1991.53973	-1.4444049	0.11783616	-12.257738	1.527E-34	1.8792E-33
PRKAR2B	ENSG00000005249	protein_coding	557.834414	-2.2400543	0.10544823	-21.243167	3.813E-100	5.6751E-98
PON1	ENSG00000005421	protein_coding	9.02408227	-1.5785287	0.24477575	-6.448877	1.1268E-10	3.573E-10
CROT	ENSG00000005469	protein_coding	1197.89884	-1.2617788	0.06911751	-18.25556	1.8688E-74	1.0655E-72
TAC1	ENSG00000006128	protein_coding	22.7757793	-2.913045	0.41974404	-6.9400508	3.9196E-12	1.382E-11
MAP3K9	ENSG00000006432	protein_coding	366.595079	-1.6688068	0.1402381	-11.89981	1.1862E-32	1.3324E-31
SCIN	ENSG00000006747	protein_coding	5241.54162	-1.9995735	0.21734398	-9.2000409	3.5781E-20	2.105E-19
UPP2	ENSG00000007001	protein_coding	273.254849	-4.3285609	0.21193124	-20.424364	1.0157E-92	1.1554E-90
PROM1	ENSG00000007062	protein_coding	5085.1008	-1.2032131	0.28887136	-4.1652212	3.1105E-05	6.1724E-05
SLC13A2	ENSG00000007216	protein_coding	561.57762	-4.7243168	0.36234218	-13.038275	7.4115E-39	1.1006E-37
CEACAM7	ENSG00000007306	protein_coding	4.1317355	-2.4948436	0.44968092	-5.5480308	2.889E-08	7.548E-08
CACNA2D2	ENSG00000007402	protein_coding	179.326796	-2.3019838	0.10766792	-21.380407	2.033E-101	3.237E-99

BAIAP3	ENSG00000007516	protein_coding	614.650464	-1.1287943	0.11746198	-9.6098701	7.2641E-22	4.6966E-21
SELE	ENSG00000007908	protein_coding	383.072739	-1.8406391	0.18607554	-9.8918917	4.5142E-23	3.1071E-22
DNAJC11	ENSG00000007923	protein_coding	2195.96748	-1.3323372	0.0628019	-21.214917	6.955E-100	1.0285E-97
TFAP2B	ENSG00000008196	protein_coding	310.852804	-5.757358	0.34772344	-16.557291	1.4184E-61	5.0248E-60
IYD	ENSG00000009765	protein_coding	463.059091	-4.2889404	0.23828251	-17.999393	1.9697E-72	1.0343E-70
UQCRC1	ENSG00000010256	protein_coding	8763.14891	-1.0334484	0.10204214	-10.127663	4.1648E-24	3.029E-23
SEMA3G	ENSG00000010319	protein_coding	2118.95287	-1.6345071	0.14856854	-11.001704	3.7498E-28	3.3931E-27
PRSS3	ENSG00000010438	protein_coding	39.8658314	-1.7152315	0.32163268	-5.3328896	9.6662E-08	2.4196E-07
SYT7	ENSG00000011347	protein_coding	471.433638	-3.3362493	0.24643669	-13.537957	9.3349E-42	1.5612E-40
LARS2	ENSG00000011376	protein_coding	1214.17319	-1.397772	0.0929577	-15.036646	4.2242E-51	1.0428E-49
DCN	ENSG00000011465	protein_coding	7575.80191	-2.5451807	0.24289403	-10.478564	1.0838E-25	8.6372E-25
SEMA3B	ENSG00000012171	protein_coding	1887.1814	-1.3304514	0.16822955	-7.9085471	2.6041E-15	1.1418E-14
EHD3	ENSG00000013016	protein_coding	2321.58928	-1.2868147	0.10646099	-12.087194	1.2343E-33	1.4579E-32
SLC7A14	ENSG00000013293	protein_coding	11.0057888	-2.9722083	0.30364305	-9.7884944	1.2616E-22	8.4874E-22
CLDN11	ENSG00000013297	protein_coding	215.786353	-2.4718164	0.17802415	-13.884725	7.84E-44	1.4399E-42
POLR3B	ENSG00000013503	protein_coding	644.356928	-1.2228111	0.06863321	-17.816611	5.2524E-71	2.6401E-69
GPRC5A	ENSG00000013588	protein_coding	1567.07351	-1.4640125	0.25137716	-5.8239677	5.7467E-09	1.5916E-08
ACP3	ENSG00000014257	protein_coding	431.306283	-5.5897135	0.16850153	-33.173072	2.633E-241	1.509E-237
DPEP1	ENSG00000015413	protein_coding	2295.18951	-4.3941969	0.26494619	-16.585243	8.9105E-62	3.1812E-60
CHDH	ENSG00000016391	protein_coding	3552.98452	-1.3238573	0.12608515	-10.499709	8.6647E-26	6.938E-25
IL20RA	ENSG00000016402	protein_coding	33.3155005	-1.0110084	0.29629117	-3.4122125	0.00064438	0.00111074
RALBP1	ENSG00000017797	protein_coding	4927.4964	-1.474413	0.05955992	-24.755119	2.731E-135	1.204E-132
CNTN1	ENSG00000018236	protein_coding	432.817949	-3.6791906	0.28698202	-12.820283	1.2624E-37	1.7911E-36
CYP24A1	ENSG00000019186	protein_coding	791.143313	-1.3559382	0.32466698	-4.176397	2.9616E-05	5.89E-05
PLEKHB1	ENSG00000021300	protein_coding	564.351303	-2.3112898	0.177082	-13.052088	6.183E-39	9.2349E-38

SLC7A9	ENSG00000021488	protein_coding	1175.11292	-1.4100475	0.19290362	-7.309596	2.6795E-13	1.0247E-12
NLRP2	ENSG00000022556	protein_coding	183.234044	-1.6397576	0.22340341	-7.3398952	2.1376E-13	8.2317E-13
GRAMD1B	ENSG00000023171	protein_coding	915.380974	-1.2777829	0.14090247	-9.0685629	1.206E-19	6.8957E-19
NDUFS1	ENSG00000023228	protein_coding	7366.49872	-1.1976215	0.07723309	-15.506585	3.131E-54	8.6514E-53
ALAS1	ENSG00000023330	protein_coding	2346.55881	-1.0089044	0.08880562	-11.360817	6.5529E-30	6.4656E-29
INSRR	ENSG00000027644	protein_coding	61.5782316	-3.6139611	0.28210227	-12.810819	1.4262E-37	2.0216E-36
USP2	ENSG00000036672	protein_coding	2051.29596	-1.8596071	0.17336589	-10.726488	7.6447E-27	6.4764E-26
CASR	ENSG00000036828	protein_coding	1057.67192	-5.5201586	0.28766208	-19.189733	4.5095E-82	3.3558E-80
CDH1	ENSG00000039068	protein_coding	8243.88498	-1.45857	0.14015635	-10.406735	2.3101E-25	1.8062E-24
SOX30	ENSG00000039600	protein_coding	20.417228	-1.9767144	0.14886064	-13.278959	3.0663E-40	4.8319E-39
CDH10	ENSG00000040731	protein_coding	7.29801474	-1.7643037	0.30266553	-5.8292193	5.5687E-09	1.5446E-08
TNC	ENSG00000041982	protein_coding	9331.37169	-1.054191	0.16959861	-6.2157998	5.1064E-10	1.5439E-09
ZPBP	ENSG00000042813	protein_coding	2.74446781	-2.910986	0.27400168	-10.623972	2.3054E-26	1.9025E-25
ADRB1	ENSG00000043591	protein_coding	56.4387347	-1.4778245	0.25656812	-5.7599692	8.4129E-09	2.2979E-08
GPM6B	ENSG00000046653	protein_coding	391.084925	-2.1019957	0.1503728	-13.978563	2.107E-44	3.9552E-43
ATP6V1H	ENSG00000047249	protein_coding	3319.89908	-1.1584321	0.08462436	-13.689109	1.1795E-42	2.0605E-41
XK	ENSG00000047597	protein_coding	105.054948	-1.8660872	0.21876774	-8.5299927	1.4636E-17	7.402E-17
FAM184B	ENSG00000047662	protein_coding	57.0514966	-1.3592357	0.19856577	-6.845267	7.6333E-12	2.6365E-11
ROS1	ENSG00000047936	protein_coding	53.7489479	-2.4301939	0.44956549	-5.4056505	6.4574E-08	1.641E-07
LMO3	ENSG00000048540	protein_coding	177.097736	-3.3590158	0.18441761	-18.214181	3.9833E-74	2.2405E-72
VPS13D	ENSG00000048707	protein_coding	4410.1452	-1.0184574	0.07959438	-12.795595	1.7352E-37	2.4505E-36
COL9A2	ENSG00000049089	protein_coding	506.697012	-2.2014223	0.16745759	-13.146148	1.7907E-39	2.7398E-38
KITLG	ENSG00000049130	protein_coding	3020.35382	-1.6259503	0.13153438	-12.361409	4.2268E-35	5.3348E-34
EPN3	ENSG00000049283	protein_coding	253.637545	-3.7433633	0.29999228	-12.478199	9.8183E-36	1.279E-34
NEDD4L	ENSG00000049759	protein_coding	6888.02128	-1.0218871	0.13049725	-7.8307177	4.8509E-15	2.0888E-14

NEXMIF	ENSG00000050030	protein_coding	130.649391	-2.9441894	0.25561431	-11.518093	1.0695E-30	1.0924E-29
SLC4A8	ENSG00000050438	protein_coding	213.874439	-2.7129605	0.11252076	-24.110755	1.928E-128	6.905E-126
PTGER3	ENSG00000050628	protein_coding	6564.27589	-2.1109418	0.2139579	-9.8661551	5.8359E-23	3.9863E-22
PRSS8	ENSG00000052344	protein_coding	3041.87372	-1.6923839	0.16021766	-10.56303	4.4216E-26	3.5944E-25
FSTL4	ENSG00000053108	protein_coding	119.447683	-2.5506239	0.21604508	-11.80598	3.6353E-32	4.003E-31
AP5M1	ENSG00000053770	protein_coding	2688.59979	-1.0589677	0.08151139	-12.991653	1.3645E-38	2.0054E-37
KCNQ1	ENSG00000053918	protein_coding	1904.0066	-1.8520696	0.12877123	-14.382635	6.6513E-47	1.3834E-45
FOXC1	ENSG00000054598	protein_coding	1480.36499	-2.1812896	0.10745587	-20.299399	1.3017E-91	1.3877E-89
SYNE2	ENSG00000054654	protein_coding	13452.054	-1.183013	0.08825327	-13.40475	5.6714E-41	9.216E-40
CYFIP2	ENSG00000055163	protein_coding	16179.0113	-2.2680604	0.1065759	-21.281175	1.696E-100	2.575E-98
MCOLN3	ENSG00000055732	protein_coding	217.214448	-1.4202454	0.25002882	-5.6803265	1.3444E-08	3.6145E-08
NPFFR2	ENSG00000056291	protein_coding	6.84088807	-1.0644403	0.31181976	-3.4136399	0.00064101	0.0011051
PHF21B	ENSG00000056487	protein_coding	24.4833326	-2.2629931	0.27975245	-8.08927	6.0023E-16	2.7388E-15
GYG2	ENSG00000056998	protein_coding	134.152128	-1.2392531	0.26641554	-4.6515796	3.294E-06	7.1827E-06
LAMC2	ENSG00000058085	protein_coding	1471.33584	-1.5575323	0.22564355	-6.9026227	5.1051E-12	1.7844E-11
STYK1	ENSG00000060140	protein_coding	39.1124913	-1.7365369	0.16497036	-10.526357	6.5313E-26	5.2573E-25
WNK1	ENSG00000060237	protein_coding	12306.2743	-1.5377315	0.07226572	-21.27885	1.783E-100	2.6969E-98
RIMBP2	ENSG00000060709	protein_coding	74.658845	-3.4538417	0.21024983	-16.427322	1.2193E-60	4.2246E-59
MPC1	ENSG00000060762	protein_coding	3229.08967	-1.53763	0.1200302	-12.810359	1.4347E-37	2.033E-36
ACAA1	ENSG00000060971	protein_coding	3513.19563	-1.6309236	0.07768298	-20.994606	7.3469E-98	1.0144E-95
CDH3	ENSG00000062038	protein_coding	472.289149	-2.2391142	0.25127892	-8.9108714	5.0633E-19	2.7951E-18
SPHK2	ENSG00000063176	protein_coding	788.698907	-1.2079869	0.06847813	-17.640476	1.2044E-69	5.7333E-68
DLX3	ENSG00000064195	protein_coding	21.1914308	-2.2393729	0.18110234	-12.365235	4.0303E-35	5.0897E-34
ATP2C2	ENSG00000064270	protein_coding	90.9803457	-1.9224666	0.21830693	-8.8062556	1.294E-18	6.9776E-18
EYA2	ENSG00000064655	protein_coding	255.808376	-1.304156	0.16809854	-7.7582831	8.6087E-15	3.6455E-14

BCAS1	ENSG00000064787	protein_coding	178.201318	-1.9768697	0.28497497	-6.9369942	4.0053E-12	1.4113E-11
OAT	ENSG00000065154	protein_coding	3378.17757	-1.2080854	0.07875536	-15.339724	4.1496E-53	1.1066E-51
GSTO2	ENSG00000065621	protein_coding	622.993695	-1.7853638	0.17656849	-10.111452	4.915E-24	3.5611E-23
PRKCQ	ENSG00000065675	protein_coding	622.532373	-1.6834531	0.12374964	-13.603701	3.8067E-42	6.4847E-41
ZMYND12	ENSG00000066185	protein_coding	253.309343	-1.3408039	0.11106302	-12.07246	1.4765E-33	1.736E-32
SLC9A3	ENSG00000066230	protein_coding	1810.49962	-3.5022155	0.23631797	-14.819929	1.089E-49	2.5483E-48
MPPED2	ENSG00000066382	protein_coding	436.034868	-2.449614	0.17833756	-13.735828	6.1944E-43	1.0981E-41
FGFR2	ENSG00000066468	protein_coding	2450.89037	-1.2785616	0.12184329	-10.493492	9.2544E-26	7.4011E-25
FECH	ENSG00000066926	protein_coding	2261.15922	-1.9770529	0.06895303	-28.672457	8.415E-181	1.378E-177
IDI1	ENSG00000067064	protein_coding	1987.99389	-1.025856	0.06361284	-16.126556	1.6602E-58	5.3521E-57
SYT1	ENSG00000067715	protein_coding	119.139022	-2.2378004	0.22583205	-9.9091358	3.7992E-23	2.6233E-22
ATP2B3	ENSG00000067842	protein_coding	22.5730608	-2.4556099	0.34207892	-7.1784893	7.0486E-13	2.6214E-12
ACSL4	ENSG00000068366	protein_coding	5970.79071	-1.5137833	0.0851055	-17.787137	8.8908E-71	4.4109E-69
TRPC7	ENSG00000069018	protein_coding	30.2534144	-1.6671622	0.24430661	-6.8240568	8.8505E-12	3.0441E-11
GAL	ENSG00000069482	protein_coding	6.89975607	-1.5096474	0.30846823	-4.8940126	9.8801E-07	2.2607E-06
TGFBR3	ENSG00000069702	protein_coding	3304.76421	-1.628549	0.09286167	-17.537366	7.4292E-69	3.4227E-67
PFN2	ENSG00000070087	protein_coding	5411.43022	-1.0861233	0.14348885	-7.5693916	3.7498E-14	1.5196E-13
PTPN3	ENSG00000070159	protein_coding	3787.40573	-1.2607155	0.11135912	-11.32117	1.0309E-29	1.008E-28
SPTB	ENSG00000070182	protein_coding	200.574884	-2.6604368	0.14799995	-17.975931	3.0077E-72	1.5704E-70
FGF10	ENSG00000070193	protein_coding	11.8346712	-3.4444352	0.31648638	-10.88336	1.3837E-27	1.2142E-26
ST6GALNAC2	ENSG00000070731	protein_coding	478.669045	-1.4739616	0.16741082	-8.8044582	1.3149E-18	7.0886E-18
CAMK2A	ENSG00000070808	protein_coding	35.3928092	-2.7682334	0.22135014	-12.506129	6.9111E-36	9.0621E-35
SLC12A3	ENSG00000070915	protein_coding	4149.7429	-4.7545935	0.38374672	-12.389926	2.9632E-35	3.7721E-34
TRPM5	ENSG00000070985	protein_coding	7.12240017	-2.1124266	0.22512236	-9.3834596	6.3843E-21	3.9126E-20
MYO3B	ENSG00000071909	protein_coding	438.489393	-3.7521074	0.26023026	-14.418413	3.9635E-47	8.3306E-46

CDH19	ENSG00000071991	protein_coding	24.2136567	-2.082272	0.39401085	-5.2848089	1.2584E-07	3.1186E-07
ADGRL1	ENSG00000072071	protein_coding	1308.19991	-1.0038358	0.11043679	-9.0896867	9.9325E-20	5.7043E-19
RPS6KA6	ENSG00000072133	protein_coding	564.725766	-2.9768291	0.21587497	-13.789598	2.9439E-43	5.2778E-42
LNX1	ENSG00000072201	protein_coding	987.500289	-1.5100079	0.11634474	-12.978738	1.6152E-38	2.3678E-37
TRPC5	ENSG00000072315	protein_coding	5.29596249	-1.53462	0.24750476	-6.2003655	5.6332E-10	1.6972E-09
MPP5	ENSG00000072415	protein_coding	2837.30336	-1.3864279	0.08149974	-17.011441	6.7556E-65	2.707E-63
EVC	ENSG00000072840	protein_coding	4609.71214	-1.2110279	0.11696273	-10.353964	4.0151E-25	3.0913E-24
LLGL2	ENSG00000073350	protein_coding	3021.25157	-1.2519491	0.0942174	-13.287876	2.722E-40	4.2982E-39
PTGS2	ENSG00000073756	protein_coding	891.166668	-1.1144468	0.22383923	-4.9787822	6.3986E-07	1.492E-06
IGF2BP2	ENSG00000073792	protein_coding	448.791571	-1.0062424	0.23218919	-4.3337175	1.4661E-05	3.0032E-05
ST6GAL1	ENSG00000073849	protein_coding	10235.5658	-2.1721399	0.12712112	-17.087168	1.8494E-65	7.5359E-64
FRY	ENSG00000073910	protein_coding	2879.19716	-1.1720143	0.09751606	-12.018681	2.8346E-33	3.288E-32
NTN4	ENSG00000074527	protein_coding	8511.13119	-1.6623052	0.12304776	-13.509431	1.3758E-41	2.2926E-40
HACD3	ENSG00000074696	protein_coding	6270.38885	-1.3846317	0.10500275	-13.186623	1.0477E-39	1.615E-38
IPCEF1	ENSG00000074706	protein_coding	451.492389	-1.0189304	0.11479494	-8.8760919	6.9251E-19	3.7964E-18
SLC12A1	ENSG00000074803	protein_coding	22201.1143	-8.2764291	0.33238901	-24.899828	7.472E-137	3.495E-134
WSCD2	ENSG00000075035	protein_coding	91.5201442	-2.2353638	0.25873053	-8.6397372	5.6342E-18	2.9247E-17
KCNQ2	ENSG00000075043	protein_coding	5.48824434	-2.5979027	0.30065075	-8.6409321	5.5756E-18	2.8946E-17
ACAT1	ENSG00000075239	protein_coding	10957.459	-1.2117407	0.13662721	-8.8689555	7.3835E-19	4.0404E-18
WNT8B	ENSG00000075290	protein_coding	14.3596141	-3.9453601	0.17286362	-22.823542	2.677E-115	6.392E-113
FGF4	ENSG00000075388	protein_coding	0.64665998	-2.0230848	0.59683246	-3.389703	0.00069968	0.00120088
ATP12A	ENSG00000075673	protein_coding	138.350225	-7.2362811	0.32976195	-21.943954	9.892E-107	1.897E-104
FMO4	ENSG00000076258	protein_coding	899.939802	-1.3966283	0.09511526	-14.683535	8.2191E-49	1.8569E-47
RAP1GAP	ENSG00000076864	protein_coding	4782.45904	-2.5650022	0.17474116	-14.678867	8.805E-49	1.9873E-47
CAPN6	ENSG00000077274	protein_coding	1347.70036	-1.2890442	0.27835341	-4.6309626	3.6397E-06	7.9022E-06

FBLN1	ENSG00000077942	protein_coding	2833.71881	-1.548122	0.1831219	-8.4540515	2.8137E-17	1.3991E-16
AMPH	ENSG00000078053	protein_coding	123.482612	-3.1571289	0.18502776	-17.063002	2.798E-65	1.1341E-63
MCCC1	ENSG00000078070	protein_coding	2254.74828	-1.0819938	0.07830506	-13.817674	1.9941E-43	3.6046E-42
NEBL	ENSG00000078114	protein_coding	4522.5276	-1.5045562	0.13168224	-11.425658	3.1129E-30	3.115E-29
RBFOX1	ENSG00000078328	protein_coding	13.817469	-2.3613465	0.29769286	-7.9321568	2.1537E-15	9.4995E-15
CLUL1	ENSG00000079101	protein_coding	86.5461277	-3.4591118	0.14623909	-23.653811	1.078E-123	3.363E-121
OSBPL6	ENSG00000079156	protein_coding	125.960406	-1.3975382	0.16596242	-8.4208117	3.7388E-17	1.8433E-16
RAPGEF3	ENSG00000079337	protein_coding	2001.30188	-1.7453769	0.11333821	-15.399722	1.6437E-53	4.4507E-52
CEACAM1	ENSG00000079385	protein_coding	1548.50107	-1.180805	0.12034198	-9.8120786	9.9889E-23	6.7527E-22
FDFT1	ENSG00000079459	protein_coding	3882.37744	-1.0097738	0.07045025	-14.333147	1.3582E-46	2.7908E-45
OPHN1	ENSG00000079482	protein_coding	631.890103	-1.6819626	0.08512926	-19.757749	6.8805E-87	6.1364E-85
AFM	ENSG00000079557	protein_coding	288.591623	-4.6944688	0.29967684	-15.665104	2.62E-55	7.53E-54
CARMIL1	ENSG00000079691	protein_coding	1587.56166	-1.4794801	0.06864481	-21.552687	4.996E-103	8.546E-101
MOXD1	ENSG00000079931	protein_coding	1260.26076	-2.2851855	0.20958163	-10.903558	1.1084E-27	9.7972E-27
SCTR	ENSG00000080293	protein_coding	412.759487	-1.425034	0.15062441	-9.4608438	3.0547E-21	1.9088E-20
RDH8	ENSG00000080511	protein_coding	16.5071131	-3.8384738	0.44487328	-8.6282408	6.2303E-18	3.221E-17
DNAAF6	ENSG00000080572	protein_coding	1.13918795	-1.1511879	0.21433965	-5.3708583	7.8363E-08	1.9786E-07
MAGI3	ENSG00000081026	protein_coding	936.305944	-1.7882034	0.11882069	-15.049596	3.4735E-51	8.6162E-50
AFP	ENSG00000081051	protein_coding	42.816704	-2.3377555	0.25733168	-9.0846006	1.0408E-19	5.9744E-19
COL4A4	ENSG00000081052	protein_coding	2062.27453	-2.1289719	0.13415969	-15.868938	1.0399E-56	3.1278E-55
IMPG2	ENSG00000081148	protein_coding	46.6334157	-1.3830046	0.11485349	-12.041467	2.151E-33	2.5058E-32
ARG2	ENSG00000081181	protein_coding	1819.05733	-1.9078068	0.22339008	-8.5402483	1.3393E-17	6.7861E-17
CACNA1S	ENSG00000081248	protein_coding	19.3336973	-2.0838233	0.21240948	-9.8104059	1.0156E-22	6.8636E-22
SLC13A1	ENSG00000081800	protein_coding	2492.86583	-1.3140513	0.28877352	-4.5504563	5.353E-06	1.1436E-05
CADPS2	ENSG00000081803	protein_coding	2697.11662	-1.5952717	0.0776612	-20.541424	9.1831E-94	1.0906E-91

ATP8B1	ENSG00000081923	protein_coding	1910.60758	-1.2399427	0.10292449	-12.04711	2.0087E-33	2.3454E-32
COL19A1	ENSG00000082293	protein_coding	66.0709288	-1.280356	0.22313062	-5.7381455	9.5719E-09	2.6038E-08
COBLL1	ENSG00000082438	protein_coding	6085.35642	-2.3893878	0.10908499	-21.903909	2.384E-106	4.516E-104
TRPM3	ENSG00000083067	protein_coding	1259.47585	-1.1629265	0.18797009	-6.1867635	6.1412E-10	1.8443E-09
BCKDHB	ENSG00000083123	protein_coding	1234.57254	-1.2714983	0.09991566	-12.725716	4.2556E-37	5.9115E-36
GRHL2	ENSG00000083307	protein_coding	297.506631	-3.6914079	0.34766721	-10.617647	2.467E-26	2.0314E-25
OXCT1	ENSG00000083720	protein_coding	3763.04619	-1.2094524	0.2017999	-5.9933251	2.0559E-09	5.9181E-09
SLCO1A2	ENSG00000084453	protein_coding	71.383344	-2.2332386	0.35667391	-6.2612895	3.8181E-10	1.1652E-09
CD82	ENSG00000085117	protein_coding	2727.65258	-1.3627238	0.14508216	-9.3927731	5.8441E-21	3.5916E-20
MECOM	ENSG00000085276	protein_coding	3666.9104	-2.5507379	0.1309242	-19.482555	1.5438E-84	1.2615E-82
IGSF9	ENSG00000085552	protein_coding	55.2077374	-1.5641245	0.21975272	-7.1176567	1.0978E-12	4.0291E-12
ABCB1	ENSG00000085563	protein_coding	2943.62683	-1.709443	0.1639841	-10.424444	1.9178E-25	1.5067E-24
WNT11	ENSG00000085741	protein_coding	81.5065361	-1.4182032	0.23507809	-6.0329026	1.6104E-09	4.6738E-09
AQP6	ENSG00000086159	protein_coding	1308.58088	-2.7119194	0.43544681	-6.2279006	4.7273E-10	1.4339E-09
FAT2	ENSG00000086570	protein_coding	117.715868	-1.807043	0.17609839	-10.261553	1.05E-24	7.9104E-24
NOX4	ENSG00000086991	protein_coding	2483.64325	-1.3236347	0.15292479	-8.6554623	4.9092E-18	2.5553E-17
L2HGDH	ENSG00000087299	protein_coding	844.810301	-1.4292557	0.10437176	-13.693893	1.1043E-42	1.9314E-41
TFAP2C	ENSG00000087510	protein_coding	97.8716461	-2.0712557	0.30857281	-6.7123728	1.9148E-11	6.4292E-11
SULT2B1	ENSG00000088002	protein_coding	106.363417	-3.1505056	0.22994339	-13.701223	9.9828E-43	1.752E-41
PTPN4	ENSG00000088179	protein_coding	1528.27899	-1.2607981	0.07097481	-17.764024	1.3426E-70	6.6248E-69
ASAP3	ENSG00000088280	protein_coding	1125.10543	-1.1199274	0.10877668	-10.295657	7.3715E-25	5.5817E-24
DOCK3	ENSG00000088538	protein_coding	87.2274905	-1.098658	0.21105075	-5.205658	1.9331E-07	4.7179E-07
TMEM40	ENSG00000088726	protein_coding	5.334068	-1.0789117	0.21452818	-5.0292309	4.9245E-07	1.1598E-06
DEFB127	ENSG00000088782	protein_coding	0.32157247	-2.6794357	0.57981513	-4.6211896	3.8155E-06	8.2658E-06
SLC4A11	ENSG00000088836	protein_coding	485.928166	-4.4300408	0.18236575	-24.292066	2.378E-130	9.083E-128

LZTS3	ENSG00000088899	protein_coding	1362.09927	-2.3076424	0.13093849	-17.623866	1.6158E-69	7.6517E-68
F11	ENSG00000088926	protein_coding	297.826621	-4.2354473	0.27632014	-15.328044	4.9672E-53	1.3215E-51
CFAP61	ENSG00000089101	protein_coding	29.1640153	-1.796884	0.14322835	-12.545589	4.2028E-36	5.565E-35
CHGB	ENSG00000089199	protein_coding	369.678027	-3.6243799	0.27340605	-13.2564	4.1431E-40	6.4843E-39
PEBP1	ENSG00000089220	protein_coding	34442.7092	-1.2064988	0.07913115	-15.246825	1.7284E-52	4.5248E-51
NOS1	ENSG00000089250	protein_coding	197.970195	-4.4347885	0.20777685	-21.343997	4.434E-101	6.961E-99
FETUB	ENSG00000090512	protein_coding	8.24292519	-1.6972878	0.43852484	-3.8704486	0.00010864	0.0002038
LAMB4	ENSG00000091128	protein_coding	112.519542	-1.3611739	0.22345128	-6.0915913	1.1179E-09	3.2878E-09
SLC26A4	ENSG00000091137	protein_coding	230.458697	-3.2698735	0.19693294	-16.603995	6.5203E-62	2.3333E-60
IL5RA	ENSG00000091181	protein_coding	21.6650551	-1.6529831	0.18412436	-8.9775361	2.769E-19	1.5514E-18
APOH	ENSG00000091583	protein_coding	164.793109	-2.5156108	0.30437258	-8.264906	1.398E-16	6.6474E-16
PITPNM3	ENSG00000091622	protein_coding	222.872411	-1.5106115	0.12369323	-12.212565	2.6636E-34	3.2405E-33
CPA1	ENSG00000091704	protein_coding	8.91773981	-2.8993539	0.24073852	-12.043581	2.0965E-33	2.4455E-32
JPH4	ENSG00000092051	protein_coding	140.657938	-1.6311528	0.11399818	-14.308586	1.9341E-46	3.958E-45
MYH7	ENSG00000092054	protein_coding	5.83401426	-3.128871	0.23379947	-13.382712	7.6311E-41	1.2313E-39
SLC7A8	ENSG00000092068	protein_coding	3968.04798	-3.5985589	0.18350689	-19.609939	1.2718E-85	1.0857E-83
DAZL	ENSG00000092345	protein_coding	2.60542481	-1.9798956	0.31837524	-6.2187486	5.0114E-10	1.5161E-09
TBL1Y	ENSG00000092377	protein_coding	21.1858944	-3.3659261	0.33229165	-10.129433	4.0901E-24	2.9756E-23
TYRO3	ENSG00000092445	protein_coding	712.612066	-2.2579763	0.12242151	-18.444278	5.7968E-76	3.5196E-74
PHGDH	ENSG00000092621	protein_coding	2315.90498	-1.4176971	0.16855936	-8.41067	4.0768E-17	2.005E-16
COL9A3	ENSG00000092758	protein_coding	68.5434556	-1.3426645	0.19220388	-6.9856265	2.8359E-12	1.0104E-11
XYLB	ENSG00000093217	protein_coding	491.097647	-1.0935237	0.1222922	-8.9418927	3.8256E-19	2.1252E-18
GABRP	ENSG00000094755	protein_coding	180.309455	-2.2095873	0.24817056	-8.9035026	5.4111E-19	2.9828E-18
EPB41L4B	ENSG00000095203	protein_coding	345.681355	-3.3527502	0.2735819	-12.255015	1.5792E-34	1.9424E-33
BLNK	ENSG00000095585	protein_coding	1052.0774	-1.1183076	0.11312038	-9.8859958	4.788E-23	3.2901E-22

TDRD1	ENSG00000095627	protein_coding	53.1522703	-1.9346724	0.3131618	-6.1778684	6.4973E-10	1.9472E-09
IL11	ENSG00000095752	protein_coding	43.9764985	-3.350727	0.29455545	-11.375539	5.536E-30	5.4752E-29
SMIM24	ENSG00000095932	protein_coding	7530.65699	-1.3023612	0.18252394	-7.1352899	9.6583E-13	3.5571E-12
PGC	ENSG00000096088	protein_coding	6.03553239	-1.131026	0.29862038	-3.7875044	0.00015217	0.00028092
DSP	ENSG00000096696	protein_coding	7240.88911	-1.4462097	0.1658693	-8.7189718	2.8074E-18	1.483E-17
ABLIM1	ENSG00000099204	protein_coding	10373.8305	-1.0180056	0.0760995	-13.377297	8.2078E-41	1.3216E-39
ERMP1	ENSG00000099219	protein_coding	4705.21953	-2.4618815	0.10364805	-23.752317	1.04E-124	3.357E-122
MACROH2A2	ENSG00000099284	protein_coding	669.121453	-1.2231571	0.10818593	-11.306065	1.2246E-29	1.1952E-28
CBARP	ENSG00000099625	protein_coding	48.2117071	-1.4443567	0.1562173	-9.2458176	2.3345E-20	1.3882E-19
PCDH11Y	ENSG00000099715	protein_coding	13.01595	-1.7670157	0.2999114	-5.8917925	3.8203E-09	1.0746E-08
AMELY	ENSG00000099721	protein_coding	0.70856686	-3.7639745	0.43211176	-8.7106504	3.0214E-18	1.5936E-17
IGFALS	ENSG00000099769	protein_coding	31.6073239	-1.7276484	0.16515468	-10.46079	1.3076E-25	1.0365E-24
MISP	ENSG00000099812	protein_coding	400.094941	-1.6429746	0.2638233	-6.2275569	4.7376E-10	1.4369E-09
PALM	ENSG00000099864	protein_coding	1794.29392	-1.6159313	0.11343347	-14.245631	4.7722E-46	9.5738E-45
CECR2	ENSG00000099954	protein_coding	88.3475816	-1.4808055	0.21720074	-6.8176817	9.2521E-12	3.1767E-11
SLC7A4	ENSG00000099960	protein_coding	15.1794054	-2.025946	0.2673476	-7.5779475	3.5106E-14	1.4259E-13
SUSD2	ENSG00000099994	protein_coding	2056.28661	-1.5268115	0.15334641	-9.956617	2.3596E-23	1.6463E-22
PLA2G3	ENSG00000100078	protein_coding	4.93555156	-2.5938531	0.42775031	-6.0639421	1.3283E-09	3.8787E-09
GCAT	ENSG00000100116	protein_coding	587.025865	-1.0783456	0.13448089	-8.0185788	1.0698E-15	4.8077E-15
MIOX	ENSG00000100253	protein_coding	6709.80334	-2.3357673	0.23880602	-9.7810235	1.3583E-22	9.1232E-22
NEFH	ENSG00000100285	protein_coding	65.3846396	-1.2860185	0.12741777	-10.092929	5.9371E-24	4.2793E-23
BIK	ENSG00000100290	protein_coding	48.9246298	-1.2957791	0.18932943	-6.844045	7.6988E-12	2.6585E-11
SYNGR1	ENSG00000100321	protein_coding	1332.12123	-1.349738	0.12805146	-10.54059	5.6145E-26	4.5425E-25
PNPLA3	ENSG00000100344	protein_coding	87.5147016	-1.6285312	0.18619285	-8.7464754	2.2012E-18	1.1698E-17
KCNK10	ENSG00000100433	protein_coding	72.162314	-3.3544193	0.18264304	-18.365985	2.4594E-75	1.4529E-73

COCH	ENSG00000100473	protein_coding	91.2030022	-1.177827	0.24727165	-4.7632916	1.9046E-06	4.2453E-06
CDKL1	ENSG00000100490	protein_coding	451.723205	-1.5680569	0.10509978	-14.919698	2.4538E-50	5.8986E-49
ISM2	ENSG00000100593	protein_coding	69.0242678	-1.4868485	0.28909695	-5.1430792	2.7027E-07	6.5158E-07
CHGA	ENSG00000100604	protein_coding	20.9556754	-2.6863609	0.27662447	-9.7112194	2.7009E-22	1.783E-21
SIX4	ENSG00000100625	protein_coding	108.441624	-2.2055322	0.26944514	-8.1854594	2.7127E-16	1.2649E-15
GALNT16	ENSG00000100626	protein_coding	617.495898	-1.0271492	0.15778131	-6.509955	7.5173E-11	2.4178E-10
HIF1A	ENSG00000100644	protein_coding	11524.6176	-1.1844885	0.09189348	-12.889798	5.1378E-38	7.397E-37
SERPINA4	ENSG00000100665	protein_coding	124.602392	-2.7977516	0.3269821	-8.5562837	1.1656E-17	5.924E-17
CPNE6	ENSG00000100884	protein_coding	89.8949945	-6.5806623	0.30536658	-21.550041	5.29E-103	9.015E-101
PCK2	ENSG00000100889	protein_coding	3920.77711	-2.3152718	0.11873565	-19.499382	1.1112E-84	9.1779E-83
DCAF11	ENSG00000100897	protein_coding	4463.70568	-1.1489834	0.05468221	-21.012013	5.0929E-98	7.0962E-96
REC8	ENSG00000100918	protein_coding	502.137411	-1.0319562	0.1376429	-7.4973443	6.5124E-14	2.6027E-13
NINL	ENSG00000101004	protein_coding	1166.35778	-1.2254987	0.08919895	-13.738936	5.9341E-43	1.0527E-41
SGK2	ENSG00000101049	protein_coding	1498.10087	-1.5287757	0.14741118	-10.370826	3.366E-25	2.606E-24
R3HDML	ENSG00000101074	protein_coding	1.7700135	-1.6789895	0.47020274	-3.5707778	0.00035592	0.00063091
RIMS4	ENSG00000101098	protein_coding	10.5903286	-1.593905	0.31368413	-5.0812422	3.7497E-07	8.9244E-07
BMP7	ENSG00000101144	protein_coding	234.59468	-3.1199417	0.35703806	-8.7384008	2.3643E-18	1.2543E-17
AVP	ENSG00000101200	protein_coding	2.43063412	-1.1686967	0.35326261	-3.3082943	0.00093866	0.00158914
CHRNA4	ENSG00000101204	protein_coding	48.7910887	-3.2252456	0.38084165	-8.4687313	2.4808E-17	1.2369E-16
SLC52A3	ENSG00000101276	protein_coding	358.799025	-2.9675249	0.18679976	-15.886128	7.9063E-57	2.3891E-55
PROKR2	ENSG00000101292	protein_coding	3.89517465	-1.1061895	0.34774382	-3.1810471	0.00146744	0.00243111
FERMT1	ENSG00000101311	protein_coding	244.563683	-1.3498304	0.20785166	-6.494201	8.3475E-11	2.6752E-10
HAO1	ENSG00000101323	protein_coding	9.42677306	-3.2733287	0.46673943	-7.0131823	2.3296E-12	8.3495E-12
PAK5	ENSG00000101349	protein_coding	25.6670799	-1.2353394	0.52877975	-2.3362078	0.01948041	0.02798589
BPI	ENSG00000101425	protein_coding	42.2163604	-2.4294456	0.18384953	-13.214315	7.2544E-40	1.1227E-38

WFDC2	ENSG00000101443	protein_coding	4826.52881	-1.0920147	0.23527754	-4.6413894	3.4607E-06	7.529E-06
LPIN2	ENSG00000101577	protein_coding	4466.15276	-1.042995	0.06554022	-15.913817	5.0819E-57	1.5448E-55
LIPG	ENSG00000101670	protein_coding	770.074579	-1.5333906	0.17912872	-8.5602722	1.126E-17	5.7283E-17
CHRDL1	ENSG00000101938	protein_coding	649.375258	-1.8087485	0.33353817	-5.4229131	5.8636E-08	1.4959E-07
PAGE4	ENSG00000101951	protein_coding	2.08226189	-3.5584689	0.44579634	-7.9822748	1.4366E-15	6.4143E-15
MCF2	ENSG00000101977	protein_coding	44.6177194	-1.2944336	0.165373	-7.8273576	4.9823E-15	2.1439E-14
SYP	ENSG00000102003	protein_coding	184.797805	-1.5628546	0.11406375	-13.701589	9.9327E-43	1.7439E-41
ASB9	ENSG00000102048	protein_coding	378.127589	-1.0924914	0.1055675	-10.348748	4.24E-25	3.259E-24
RS1	ENSG00000102104	protein_coding	13.1596588	-1.3015308	0.14779489	-8.8063314	1.2931E-18	6.9742E-18
PCSK1N	ENSG00000102109	protein_coding	753.68564	-1.5598932	0.2660926	-5.8622192	4.5672E-09	1.2769E-08
GPR50	ENSG00000102195	protein_coding	0.80342322	-2.4454956	0.43414826	-5.6328583	1.7725E-08	4.7174E-08
VGLL1	ENSG00000102243	protein_coding	64.7477269	-6.1655599	0.48694072	-12.661829	9.6232E-37	1.3156E-35
PCDH11X	ENSG00000102290	protein_coding	3.91643133	-1.8873051	0.31837615	-5.9279096	3.0682E-09	8.7007E-09
SYTL4	ENSG00000102362	protein_coding	819.410512	-1.1622262	0.11763587	-9.8798623	5.0903E-23	3.489E-22
ZDHHC15	ENSG00000102383	protein_coding	229.289441	-1.3021571	0.15111718	-8.6168695	6.8809E-18	3.5477E-17
CAB39L	ENSG00000102547	protein_coding	1101.58064	-1.6212112	0.09984968	-16.236518	2.7828E-59	9.2171E-58
KLF5	ENSG00000102554	protein_coding	998.344351	-1.4790941	0.15155499	-9.759455	1.6806E-22	1.121E-21
FGF9	ENSG00000102678	protein_coding	577.982615	-2.886451	0.42156815	-6.8469381	7.5447E-12	2.6073E-11
SGCG	ENSG00000102683	protein_coding	16.6829829	-1.3739459	0.17216768	-7.9802779	1.46E-15	6.5151E-15
SLC25A15	ENSG00000102743	protein_coding	254.710302	-1.094039	0.11009688	-9.9370572	2.8719E-23	1.9968E-22
VWA8	ENSG00000102763	protein_coding	2094.73173	-1.1460754	0.07909041	-14.4907	1.3872E-47	2.9674E-46
OLFM4	ENSG00000102837	protein_coding	174.977575	-4.8860359	0.36032971	-13.559903	6.9228E-42	1.165E-40
PARD6A	ENSG00000102981	protein_coding	276.723536	-1.2279987	0.1175229	-10.449016	1.4805E-25	1.1694E-24
CCDC113	ENSG00000103021	protein_coding	293.718848	-1.1921818	0.14634727	-8.1462519	3.7538E-16	1.7348E-15
ESRP2	ENSG00000103067	protein_coding	888.360017	-1.5249189	0.11342982	-13.443721	3.3515E-41	5.4899E-40

FA2H	ENSG00000103089	protein_coding	236.992128	-1.6850821	0.25338488	-6.6502866	2.9252E-11	9.6995E-11
ZP2	ENSG00000103310	protein_coding	3.15183606	-2.7974966	0.26174555	-10.687848	1.1603E-26	9.7329E-26
CRYM	ENSG00000103316	protein_coding	998.404063	-1.2224091	0.16718143	-7.3118712	2.6345E-13	1.008E-12
TOX3	ENSG00000103460	protein_coding	594.558603	-2.1886867	0.24994968	-8.756509	2.0139E-18	1.0737E-17
QPRT	ENSG00000103485	protein_coding	4398.40494	-1.1768533	0.16238457	-7.2473218	4.2509E-13	1.6017E-12
SLC6A2	ENSG00000103546	protein_coding	13.4994285	-1.9838991	0.42893148	-4.6252122	3.7422E-06	8.115E-06
IQCH	ENSG00000103599	protein_coding	161.856992	-1.1712711	0.09822276	-11.92464	8.8064E-33	9.9554E-32
CORO2B	ENSG00000103647	protein_coding	411.801903	-2.0060383	0.16911727	-11.861818	1.8687E-32	2.0813E-31
CTSH	ENSG00000103811	protein_coding	10132.0558	-1.2868493	0.09122902	-14.105702	3.5029E-45	6.7868E-44
ZDHHC2	ENSG00000104219	protein_coding	2664.92936	-1.8023343	0.12737252	-14.150103	1.8648E-45	3.6532E-44
RP1	ENSG00000104237	protein_coding	20.1915962	-1.5944338	0.18144497	-8.7874236	1.5303E-18	8.2191E-18
CA2	ENSG00000104267	protein_coding	8190.51851	-1.4838441	0.12868925	-11.530443	9.2666E-31	9.4861E-30
CALB1	ENSG00000104327	protein_coding	2845.95924	-7.5296097	0.28594438	-26.332428	8.159E-153	6.562E-150
SFRP1	ENSG00000104332	protein_coding	3899.29558	-5.2834968	0.24603185	-21.474849	2.676E-102	4.397E-100
LAPTM4B	ENSG00000104341	protein_coding	5993.82142	-1.0453553	0.09592792	-10.897299	1.1873E-27	1.0481E-26
PLAT	ENSG00000104368	protein_coding	3754.86931	-1.7852886	0.11333831	-15.751854	6.6696E-56	1.9524E-54
ESRP1	ENSG00000104413	protein_coding	474.815369	-3.0772215	0.34798944	-8.8428587	9.33E-19	5.0735E-18
SH2D4A	ENSG00000104611	protein_coding	1095.30565	-1.1437084	0.11083591	-10.318934	5.7862E-25	4.4075E-24
DHDH	ENSG00000104808	protein_coding	319.478944	-1.2396489	0.2175458	-5.6983353	1.2098E-08	3.2637E-08
CKM	ENSG00000104879	protein_coding	20.7905669	-2.8874913	0.19880781	-14.524034	8.5337E-48	1.8392E-46
KLC3	ENSG00000104892	protein_coding	78.068297	-1.9763122	0.2498682	-7.9094184	2.5859E-15	1.1343E-14
CLEC4M	ENSG00000104938	protein_coding	2.77592744	-2.1343073	0.27298281	-7.8184677	5.347E-15	2.2968E-14
CCNP	ENSG00000105219	protein_coding	65.8883527	-4.7924943	0.22352989	-21.440061	5.654E-102	9.094E-100
TJP3	ENSG00000105289	protein_coding	288.994501	-2.7559448	0.22728187	-12.125669	7.7227E-34	9.212E-33
MYH14	ENSG00000105357	protein_coding	2027.39945	-1.7647582	0.20971342	-8.4150942	3.9258E-17	1.9328E-16

CAPS	ENSG00000105519	protein_coding	776.756281	-1.7517345	0.15322171	-11.432678	2.8712E-30	2.8794E-29
FAM83E	ENSG00000105523	protein_coding	21.9909665	-2.3722628	0.30226629	-7.8482546	4.2187E-15	1.8232E-14
GCDH	ENSG00000105607	protein_coding	1099.35936	-1.0639827	0.07228905	-14.718448	4.9079E-49	1.1165E-47
UPK1A	ENSG00000105668	protein_coding	8.55095912	-1.5682536	0.23099291	-6.7891851	1.1277E-11	3.8457E-11
GRIK5	ENSG00000105737	protein_coding	260.985641	-3.0070237	0.25119426	-11.970909	5.0472E-33	5.7857E-32
CADM4	ENSG00000105767	protein_coding	916.330466	-1.6638118	0.17849074	-9.3215581	1.1464E-20	6.9442E-20
PON3	ENSG00000105852	protein_coding	74.8027846	-1.4328033	0.19903419	-7.1987797	6.0754E-13	2.2674E-12
ATP6V0A4	ENSG00000105929	protein_coding	4278.70094	-2.0166612	0.45518566	-4.4304146	9.4052E-06	1.9635E-05
HIBADH	ENSG00000106049	protein_coding	6450.48559	-1.079856	0.08354543	-12.925376	3.2371E-38	4.696E-37
CPVL	ENSG00000106066	protein_coding	5325.86329	-1.3924351	0.12869711	-10.819474	2.7837E-27	2.4123E-26
CHN2	ENSG00000106069	protein_coding	520.771864	-1.5796985	0.11663359	-13.544113	8.5844E-42	1.4388E-40
COBL	ENSG00000106078	protein_coding	1822.15512	-1.2097448	0.1303397	-9.2814759	1.6715E-20	1.0036E-19
GHRHR	ENSG00000106128	protein_coding	5.9148812	-1.6215233	0.19067783	-8.5039948	1.8318E-17	9.2062E-17
PTPRZ1	ENSG00000106278	protein_coding	24.5014345	-1.0998508	0.2768434	-3.9728267	7.1025E-05	0.00013568
AGFG2	ENSG00000106351	protein_coding	1619.39783	-1.1743412	0.08877321	-13.228555	6.0031E-40	9.3157E-39
MEST	ENSG00000106484	protein_coding	1096.91745	-1.1715361	0.17593787	-6.6588059	2.7606E-11	9.1696E-11
AGR2	ENSG00000106541	protein_coding	189.196326	-2.6522036	0.41351829	-6.4137517	1.4198E-10	4.4671E-10
TMEM245	ENSG00000106771	protein_coding	8127.08544	-1.0869943	0.08165054	-13.312764	1.9513E-40	3.1005E-39
C5	ENSG00000106804	protein_coding	370.108098	-1.266576	0.1084851	-11.675115	1.7084E-31	1.8195E-30
OGN	ENSG00000106809	protein_coding	358.397282	-1.1557454	0.28822065	-4.0099328	6.0736E-05	0.00011689
PTGR1	ENSG00000106853	protein_coding	5265.54158	-1.320858	0.09760312	-13.532948	9.9934E-42	1.6701E-40
SUSD1	ENSG00000106868	protein_coding	1122.30609	-1.4105274	0.11775368	-11.978627	4.5988E-33	5.2835E-32
AMBP	ENSG00000106927	protein_coding	195.165809	-1.8570136	0.20717604	-8.9634573	3.1465E-19	1.7573E-18
RLN1	ENSG00000107018	protein_coding	8.35254971	-1.1366017	0.1347222	-8.4366327	3.2661E-17	1.6162E-16
NCS1	ENSG00000107130	protein_coding	1643.52799	-1.2216741	0.11689492	-10.451046	1.4492E-25	1.1456E-24

TYRP1	ENSG00000107165	protein_coding	163.938667	-5.2555799	0.35949216	-14.619456	2.1108E-48	4.6971E-47
PIP5K1B	ENSG00000107242	protein_coding	375.383307	-1.6867749	0.14886185	-11.331143	9.1996E-30	9.0227E-29
BAG1	ENSG00000107262	protein_coding	4116.60361	-1.2150021	0.09992567	-12.159058	5.1347E-34	6.1699E-33
SH3GL2	ENSG00000107295	protein_coding	280.302563	-2.7097507	0.33404939	-8.1118264	4.9864E-16	2.2879E-15
PTGDS	ENSG00000107317	protein_coding	1899.07484	-1.866294	0.24486728	-7.6216554	2.5044E-14	1.0271E-13
GATA3	ENSG00000107485	protein_coding	1052.85365	-3.0897464	0.19760006	-15.636364	4.1157E-55	1.1762E-53
ATRNL1	ENSG00000107518	protein_coding	280.195819	-1.9127466	0.24719783	-7.737716	1.0122E-14	4.2666E-14
PHYH	ENSG00000107537	protein_coding	3958.1191	-1.1362961	0.11602565	-9.7934909	1.2008E-22	8.0904E-22
DNMBP	ENSG00000107554	protein_coding	1808.85721	-1.0100813	0.05973712	-16.908772	3.8771E-64	1.5049E-62
CXCL12	ENSG00000107562	protein_coding	6454.7589	-1.2572376	0.14059019	-8.94257	3.8022E-19	2.113E-18
SPOCK2	ENSG00000107742	protein_coding	8513.2019	-1.0799939	0.10510536	-10.275346	9.1017E-25	6.8714E-24
PITX3	ENSG00000107859	protein_coding	2.14166441	-1.7551329	0.28641165	-6.1280079	8.8986E-10	2.6384E-09
CPEB3	ENSG00000107864	protein_coding	693.497344	-1.3527601	0.08322885	-16.2535	2.1097E-59	7.0283E-58
SORCS1	ENSG00000108018	protein_coding	107.117056	-2.2699082	0.28302432	-8.0201877	1.0558E-15	4.7475E-15
DNAJC12	ENSG00000108176	protein_coding	282.624998	-1.3557872	0.18006452	-7.5294522	5.0954E-14	2.05E-13
PBLD	ENSG00000108187	protein_coding	5795.47016	-1.2543403	0.13333971	-9.4071018	5.1001E-21	3.1449E-20
CYP2C18	ENSG00000108242	protein_coding	10.8095285	-1.1422468	0.28815852	-3.9639529	7.3719E-05	0.00014061
KRT23	ENSG00000108244	protein_coding	38.6712375	-1.3260734	0.28470919	-4.6576415	3.1985E-06	6.9817E-06
RNF43	ENSG00000108375	protein_coding	425.324702	-2.5515081	0.14971309	-17.042652	3.9634E-65	1.6008E-63
HOXB6	ENSG00000108511	protein_coding	814.766476	-2.0839228	0.13575554	-15.350554	3.5118E-53	9.3977E-52
RASD1	ENSG00000108551	protein_coding	3315.12733	-2.5412539	0.16601663	-15.307225	6.8422E-53	1.8151E-51
SLC6A4	ENSG00000108576	protein_coding	34.3892817	-2.1461698	0.1591468	-13.485473	1.9044E-41	3.1619E-40
ASIC2	ENSG00000108684	protein_coding	54.6045014	-1.7545767	0.23638561	-7.4225191	1.1491E-13	4.5155E-13
MPP2	ENSG00000108852	protein_coding	130.504013	-1.9450346	0.17913515	-10.857917	1.8287E-27	1.5953E-26
VTN	ENSG00000109072	protein_coding	117.878645	-1.0258632	0.24473631	-4.1917084	2.7686E-05	5.5243E-05

FOXN1	ENSG00000109101	protein_coding	9.74473049	-2.5366628	0.2392127	-10.604215	2.8485E-26	2.338E-25
GABRA4	ENSG00000109158	protein_coding	9.59480696	-2.9287758	0.33988684	-8.6169144	6.8783E-18	3.5467E-17
CWH43	ENSG00000109182	protein_coding	251.486406	-2.735829	0.35448911	-7.7176671	1.1848E-14	4.9709E-14
USP46	ENSG00000109189	protein_coding	1042.94356	-1.3236798	0.06404113	-20.669213	6.5577E-95	8.1027E-93
ODAM	ENSG00000109205	protein_coding	20.6878632	-2.8662087	0.6429582	-4.4578461	8.2787E-06	1.7354E-05
CRACD	ENSG00000109265	protein_coding	103.496016	-1.2769691	0.18936161	-6.7435483	1.5456E-11	5.2256E-11
MAPK10	ENSG00000109339	protein_coding	1076.65646	-1.1011689	0.0794358	-13.862376	1.0707E-43	1.957E-42
TBC1D9	ENSG00000109436	protein_coding	3410.82524	-1.1459042	0.09333414	-12.277439	1.1973E-34	1.4822E-33
SOD3	ENSG00000109610	protein_coding	2599.66687	-1.5461394	0.12890901	-11.994036	3.8184E-33	4.4046E-32
TRIM2	ENSG00000109654	protein_coding	5498.69923	-1.7300306	0.15295845	-11.310461	1.1648E-29	1.1377E-28
GLRB	ENSG00000109738	protein_coding	263.924821	-1.2247069	0.22984574	-5.3283866	9.9089E-08	2.4781E-07
PPARGC1A	ENSG00000109819	protein_coding	3573.74093	-1.6818131	0.18115155	-9.2840117	1.6321E-20	9.8072E-20
DDX25	ENSG00000109832	protein_coding	46.675472	-2.4778958	0.20288473	-12.213318	2.639E-34	3.2115E-33
JHY	ENSG00000109944	protein_coding	402.18899	-1.0531768	0.08972019	-11.73846	8.0947E-32	8.7599E-31
VWA5A	ENSG00000110002	protein_coding	1706.17301	-1.1714446	0.09112209	-12.85577	7.9827E-38	1.1396E-36
ST3GAL4	ENSG00000110080	protein_coding	2012.97433	-1.1804274	0.14561317	-8.1065978	5.2057E-16	2.3847E-15
FOLR3	ENSG00000110203	protein_coding	68.5730933	-3.1907656	0.30423009	-10.488001	9.8081E-26	7.8303E-25
APOC3	ENSG00000110245	protein_coding	73.4631354	-1.5813762	0.43918399	-3.6007147	0.00031734	0.00056542
KIAA1549L	ENSG00000110427	protein_coding	141.856475	-2.0676446	0.2028197	-10.194496	2.0983E-24	1.5564E-23
CALCA	ENSG00000110680	protein_coding	233.847552	-3.04546	0.51321096	-5.934129	2.9541E-09	8.3913E-09
DAO	ENSG00000110887	protein_coding	638.754354	-3.2551759	0.25209538	-12.912478	3.8278E-38	5.5406E-37
SYT10	ENSG00000110975	protein_coding	69.3572888	-1.7498458	0.43517839	-4.020985	5.7955E-05	0.00011179
BCL7A	ENSG00000110987	protein_coding	1606.70614	-1.0336983	0.07794862	-13.261278	3.8822E-40	6.0885E-39
CYP27B1	ENSG00000111012	protein_coding	237.938448	-3.6154528	0.19390172	-18.6458	1.3659E-77	8.8192E-76
MYF6	ENSG00000111046	protein_coding	1.87278519	-2.1788095	0.33830811	-6.4403111	1.1923E-10	3.7738E-10

MYF5	ENSG00000111049	protein_coding	0.407156	-2.3029507	0.59892789	-3.8451218	0.00012049	0.00022497
PPM1H	ENSG00000111110	protein_coding	1403.54731	-1.8760748	0.15266923	-12.288493	1.0443E-34	1.2967E-33
SLC6A12	ENSG00000111181	protein_coding	3790.11627	-1.1524597	0.15037721	-7.6637924	1.8052E-14	7.4707E-14
PRMT8	ENSG00000111218	protein_coding	12.5887027	-1.6523516	0.26588122	-6.2146231	5.1448E-10	1.555E-09
AKAP3	ENSG00000111254	protein_coding	96.8268173	-2.3432995	0.10068502	-23.273566	8.214E-120	2.255E-117
KCNA1	ENSG00000111262	protein_coding	3.93743108	-1.9282157	0.28137428	-6.8528499	7.2393E-12	2.5069E-11
SCNN1A	ENSG00000111319	protein_coding	5659.34359	-2.8668824	0.25899973	-11.069055	1.7726E-28	1.6271E-27
RASAL1	ENSG00000111344	protein_coding	1434.64835	-1.0556856	0.15465543	-6.8260494	8.7285E-12	3.0041E-11
RERGL	ENSG00000111404	protein_coding	216.393926	-1.5682828	0.19011227	-8.2492455	1.594E-16	7.554E-16
VDR	ENSG00000111424	protein_coding	1430.76853	-1.7298928	0.1373285	-12.59675	2.2002E-36	2.9543E-35
ADGRD1	ENSG00000111452	protein_coding	160.47105	-1.3104673	0.15773982	-8.3077775	9.7511E-17	4.6846E-16
TBC1D30	ENSG00000111490	protein_coding	337.375521	-1.1001942	0.08080673	-13.615131	3.2556E-42	5.5625E-41
LDHB	ENSG00000111716	protein_coding	31456.5295	-1.5515552	0.0832814	-18.630273	1.8259E-77	1.169E-75
FRK	ENSG00000111816	protein_coding	2620.08878	-1.2863561	0.0960701	-13.389765	6.94E-41	1.1222E-39
RSPH4A	ENSG00000111834	protein_coding	44.0666391	-1.0382268	0.12413789	-8.3634969	6.0886E-17	2.961E-16
ADTRP	ENSG00000111863	protein_coding	292.203307	-1.9651298	0.20214126	-9.7215665	2.44E-22	1.614E-21
FAM184A	ENSG00000111879	protein_coding	230.065366	-1.5664554	0.12379643	-12.653478	1.0703E-36	1.4607E-35
MAN1A1	ENSG00000111885	protein_coding	7667.39904	-1.4042631	0.10566706	-13.289508	2.6633E-40	4.2084E-39
TPD52L1	ENSG00000111907	protein_coding	1248.91513	-1.7147853	0.15745935	-10.890337	1.2817E-27	1.1292E-26
NCOA7	ENSG00000111912	protein_coding	5022.4477	-1.1537502	0.10891422	-10.593201	3.2044E-26	2.6222E-25
UST	ENSG00000111962	protein_coding	429.218825	-1.2028332	0.1970656	-6.1037196	1.0363E-09	3.0579E-09
TULP1	ENSG00000112041	protein_coding	2.2935326	-1.0111432	0.20056655	-5.041435	4.6205E-07	1.0911E-06
PHACTR1	ENSG00000112137	protein_coding	1079.02234	-1.0932873	0.10406948	-10.50536	8.1611E-26	6.5404E-25
BMP5	ENSG00000112175	protein_coding	90.378906	-1.4165256	0.31027025	-4.5654575	4.9841E-06	1.0683E-05
BACH2	ENSG00000112182	protein_coding	147.320372	-1.0997223	0.12561288	-8.754853	2.0437E-18	1.0889E-17

GPR63	ENSG00000112218	protein_coding	54.8734018	-1.0231792	0.12411144	-8.244036	1.665E-16	7.8749E-16
KHDRBS2	ENSG00000112232	protein_coding	11.7095378	-2.2810703	0.20856342	-10.937058	7.6647E-28	6.8291E-27
SIM1	ENSG00000112246	protein_coding	1226.98868	-2.9955211	0.28604318	-10.472269	1.1583E-25	9.2154E-25
BVES	ENSG00000112276	protein_coding	245.237855	-1.2707072	0.12287212	-10.341704	4.5635E-25	3.5012E-24
ALDH5A1	ENSG00000112294	protein_coding	2274.03942	-1.0891206	0.09691641	-11.237731	2.6614E-29	2.5619E-28
EYA4	ENSG00000112319	protein_coding	273.288106	-3.4993637	0.17905315	-19.543715	4.666E-85	3.9103E-83
SLC16A10	ENSG00000112394	protein_coding	529.494433	-1.5127166	0.17382666	-8.7024429	3.2482E-18	1.7093E-17
EPM2A	ENSG00000112425	protein_coding	521.324026	-1.396607	0.06689144	-20.87871	8.3618E-97	1.1307E-94
UNC93A	ENSG00000112494	protein_coding	71.5217255	-1.0732	0.39341717	-2.7278932	0.00637402	0.00980052
PACRG	ENSG00000112530	protein_coding	269.310528	-1.3354758	0.25437277	-5.2500738	1.5204E-07	3.7411E-07
C6orf118	ENSG00000112539	protein_coding	3.92279973	-1.165135	0.2748159	-4.2396926	2.2383E-05	4.5051E-05
PRPH2	ENSG00000112619	protein_coding	55.7434549	-1.2619901	0.15128447	-8.3418348	7.3147E-17	3.5441E-16
GMDS	ENSG00000112699	protein_coding	879.864835	-1.0059753	0.06993348	-14.384746	6.4515E-47	1.3425E-45
CLIC5	ENSG00000112782	protein_coding	2215.05942	-3.8274958	0.18075139	-21.17547	1.608E-99	2.3471E-97
SEMA5A	ENSG00000112902	protein_coding	4249.45925	-1.0246807	0.11074373	-9.2527198	2.1885E-20	1.3034E-19
C7	ENSG00000112936	protein_coding	12503.0128	-3.310292	0.24597557	-13.457808	2.7701E-41	4.5645E-40
NNT	ENSG00000112992	protein_coding	6961.92103	-1.2375328	0.12285126	-10.073424	7.2413E-24	5.1956E-23
SLC4A9	ENSG00000113073	protein_coding	249.925492	-3.1170311	0.362009	-8.6103691	7.2826E-18	3.7502E-17
CDH9	ENSG00000113100	protein_coding	57.8027833	-2.2491823	0.34036548	-6.6081387	3.8918E-11	1.2779E-10
HMGCR	ENSG00000113161	protein_coding	2211.01129	-1.0268944	0.07543421	-13.613113	3.3468E-42	5.7139E-41
PDE8B	ENSG00000113231	protein_coding	733.722961	-1.0432809	0.11309017	-9.2252126	2.83E-20	1.6737E-19
SLC27A6	ENSG00000113396	protein_coding	1.61142044	-1.4574781	0.34293977	-4.2499534	2.1381E-05	4.3112E-05
AGXT2	ENSG00000113492	protein_coding	3899.59666	-1.242371	0.21222753	-5.8539577	4.8001E-09	1.3395E-08
PRLR	ENSG00000113494	protein_coding	1517.67404	-1.8104275	0.21804377	-8.303046	1.0148E-16	4.8694E-16
FGF1	ENSG00000113578	protein_coding	1379.72097	-4.2361482	0.17366564	-24.392552	2.052E-131	8.178E-129

CPEB4	ENSG00000113742	protein_coding	5732.63975	-1.0093469	0.1013764	-9.9564293	2.364E-23	1.6492E-22
CNTN3	ENSG00000113805	protein_coding	218.286708	-1.7033875	0.32672365	-5.2135421	1.8527E-07	4.53E-07
KNG1	ENSG00000113889	protein_coding	7700.98515	-7.5316943	0.38756913	-19.433163	4.0464E-84	3.2485E-82
HRG	ENSG00000113905	protein_coding	403.66418	-7.0539069	0.34534058	-20.425942	9.8343E-93	1.1214E-90
CLDN16	ENSG00000113946	protein_coding	846.57717	-5.8696521	0.25072073	-23.411116	3.293E-121	9.494E-119
PCCB	ENSG00000114054	protein_coding	2247.70958	-1.659324	0.10870747	-15.26412	1.326E-52	3.4895E-51
RBP2	ENSG00000114113	protein_coding	17.2626871	-3.9940526	0.17608711	-22.682255	6.706E-114	1.585E-111
BCHE	ENSG00000114200	protein_coding	155.790067	-1.013751	0.26506195	-3.8245813	0.00013099	0.0002435
WNT5A	ENSG00000114251	protein_coding	545.29264	-1.2862429	0.15641275	-8.223389	1.9783E-16	9.3004E-16
HYAL1	ENSG00000114378	protein_coding	2473.71514	-1.0733086	0.1057506	-10.149433	3.333E-24	2.4377E-23
C3orf52	ENSG00000114529	protein_coding	284.089459	-1.9258195	0.17523692	-10.989805	4.2785E-28	3.8611E-27
ROPN1B	ENSG00000114547	protein_coding	5.47853539	-1.318455	0.17859132	-7.3825252	1.5532E-13	6.0455E-13
ATP6V1A	ENSG00000114573	protein_coding	11400.9214	-1.140808	0.132898	-8.5840868	9.1561E-18	4.6829E-17
ABHD14A-ACY1	ENSG00000114786	protein_coding	21.4333441	-1.1126398	0.14020855	-7.9356059	2.0947E-15	9.2472E-15
ARHGEF26	ENSG00000114790	protein_coding	207.340193	-1.4153365	0.146271	-9.6761248	3.8088E-22	2.4971E-21
TNNC1	ENSG00000114854	protein_coding	79.5148975	-3.3460179	0.2172604	-15.400956	1.6127E-53	4.3717E-52
EPB41L5	ENSG00000115109	protein_coding	2329.55552	-1.7729105	0.08545978	-20.745554	1.3447E-95	1.7028E-93
TFCP2L1	ENSG00000115112	protein_coding	4647.77103	-3.0989203	0.30071245	-10.305261	6.671E-25	5.0647E-24
ITGB6	ENSG00000115221	protein_coding	2184.92629	-2.5974038	0.19968386	-13.007581	1.1079E-38	1.6347E-37
PDE1A	ENSG00000115252	protein_coding	2273.55375	-2.8073746	0.16810316	-16.700308	1.3039E-62	4.7894E-61
REEP6	ENSG00000115255	protein_coding	318.917364	-2.9791844	0.16876954	-17.652382	9.7553E-70	4.6631E-68
GRB14	ENSG00000115290	protein_coding	887.103001	-1.293015	0.18533431	-6.9766629	3.0227E-12	1.0746E-11
GALNT3	ENSG00000115339	protein_coding	599.841225	-2.5640536	0.17264881	-14.851267	6.8266E-50	1.6089E-48
TACR1	ENSG00000115353	protein_coding	112.547023	-1.4334072	0.17566058	-8.1600957	3.3476E-16	1.5512E-15
IGFBP2	ENSG00000115457	protein_coding	2568.07567	-1.7902585	0.19243766	-9.3030571	1.3646E-20	8.2322E-20

EFHD1	ENSG00000115468	protein_coding	4517.23586	-2.633102	0.12861939	-20.472045	3.8224E-93	4.3915E-91
KCNJ13	ENSG00000115474	protein_coding	165.510478	-2.3071583	0.33069467	-6.9767024	3.0219E-12	1.0743E-11
IL1RL1	ENSG00000115602	protein_coding	928.358425	-2.0960554	0.24281182	-8.632427	6.0064E-18	3.1101E-17
SLC9A2	ENSG00000115616	protein_coding	334.753632	-2.9896506	0.3383291	-8.8365163	9.875E-19	5.3622E-18
UXS1	ENSG00000115652	protein_coding	3533.57242	-1.4532853	0.06040396	-24.059436	6.65E-128	2.309E-125
SLC5A7	ENSG00000115665	protein_coding	11.3257857	-4.3604429	0.23224262	-18.775378	1.2009E-78	8.0249E-77
PROC	ENSG00000115718	protein_coding	277.614336	-1.9666388	0.24011085	-8.1905454	2.6005E-16	1.214E-15
PLCL1	ENSG00000115896	protein_coding	1957.9949	-2.6597293	0.13256704	-20.063278	1.5455E-89	1.5302E-87
ATP6V1B1	ENSG00000116039	protein_coding	2203.81043	-2.6769786	0.35833398	-7.4706247	7.9815E-14	3.1697E-13
PARD3B	ENSG00000116117	protein_coding	1316.70911	-1.4306295	0.07758192	-18.440242	6.2461E-76	3.7874E-74
BCL9	ENSG00000116128	protein_coding	1341.61778	-1.0700545	0.06016461	-17.785448	9.1627E-71	4.5409E-69
PAPPA2	ENSG00000116183	protein_coding	918.380594	-3.4099482	0.30552188	-11.161061	6.3233E-29	5.9618E-28
ANGPTL1	ENSG00000116194	protein_coding	401.762749	-2.4942167	0.22931073	-10.877017	1.4834E-27	1.3002E-26
NPHS2	ENSG00000116218	protein_coding	985.567934	-7.1043532	0.49738767	-14.283332	2.7799E-46	5.6361E-45
CHD5	ENSG00000116254	protein_coding	46.9932781	-1.5705305	0.2758887	-5.6926237	1.251E-08	3.3698E-08
DLGAP3	ENSG00000116544	protein_coding	41.7069325	-1.5979162	0.11177953	-14.295249	2.3427E-46	4.7708E-45
FBXO2	ENSG00000116661	protein_coding	471.551911	-2.4735951	0.20853898	-11.861548	1.8748E-32	2.0875E-31
DNAJC6	ENSG00000116675	protein_coding	663.562236	-1.4435502	0.13082398	-11.034293	2.611E-28	2.379E-27
PLA2G4A	ENSG00000116711	protein_coding	445.13434	-1.543902	0.13808895	-11.180489	5.0809E-29	4.8143E-28
WLS	ENSG00000116729	protein_coding	4983.9794	-1.4682908	0.10296226	-14.260476	3.8581E-46	7.7741E-45
CTH	ENSG00000116761	protein_coding	286.572016	-1.8779782	0.10514213	-17.861329	2.3595E-71	1.2005E-69
AGMAT	ENSG00000116771	protein_coding	2946.53625	-2.4773941	0.1837133	-13.48511	1.9138E-41	3.1763E-40
HAO2	ENSG00000116882	protein_coding	2056.57945	-2.5074333	0.24330238	-10.305831	6.6316E-25	5.0356E-24
NT5C1A	ENSG00000116981	protein_coding	5.68672855	-4.2343761	0.19970029	-21.203655	8.836E-100	1.3024E-97
HPCAL4	ENSG00000116983	protein_coding	140.851529	-3.9945921	0.26340097	-15.165442	5.9894E-52	1.5416E-50

ACADM	ENSG00000117054	protein_coding	5695.90082	-1.5380933	0.09880764	-15.566542	1.2287E-54	3.4598E-53
PADI2	ENSG00000117115	protein_coding	1342.62993	-2.4819989	0.16467266	-15.072319	2.4631E-51	6.1432E-50
RAB29	ENSG00000117280	protein_coding	3171.26727	-1.1624089	0.09742079	-11.931837	8.0771E-33	9.1536E-32
CR2	ENSG00000117322	protein_coding	136.447181	-3.8758103	0.31649626	-12.245991	1.7651E-34	2.164E-33
IPO13	ENSG00000117408	protein_coding	2474.86236	-1.0580482	0.06745994	-15.684097	1.9431E-55	5.6056E-54
TSPAN1	ENSG00000117472	protein_coding	10679.0248	-1.1288662	0.19651185	-5.7445198	9.2182E-09	2.5107E-08
CCDC181	ENSG00000117477	protein_coding	108.050101	-3.062558	0.16261511	-18.83317	4.0385E-79	2.7225E-77
SLC19A2	ENSG00000117479	protein_coding	1037.22836	-1.1035733	0.15148715	-7.2849303	3.2184E-13	1.2236E-12
ABCD3	ENSG00000117528	protein_coding	4482.81065	-1.0050852	0.07037111	-14.282639	2.8077E-46	5.6899E-45
IRF6	ENSG00000117595	protein_coding	1947.32075	-1.5686559	0.18137588	-8.6486463	5.2114E-18	2.7095E-17
SERPINC1	ENSG00000117601	protein_coding	35.1165076	-1.1596959	0.26390551	-4.3943604	1.111E-05	2.3026E-05
MAN1C1	ENSG00000117643	protein_coding	1285.8867	-2.1988089	0.16415789	-13.394476	6.5134E-41	1.0554E-39
PROX1	ENSG00000117707	protein_coding	542.844089	-4.3231644	0.21040174	-20.547189	8.155E-94	9.71E-92
TREH	ENSG00000118094	protein_coding	265.157851	-2.3397791	0.22680885	-10.316084	5.9605E-25	4.5335E-24
TNNT2	ENSG00000118194	protein_coding	147.659248	-4.8667364	0.26803907	-18.156817	1.1341E-73	6.2789E-72
ATP10B	ENSG00000118322	protein_coding	30.4079575	-2.0765417	0.22998882	-9.0288813	1.7344E-19	9.8312E-19
CCN2	ENSG00000118523	protein_coding	13062.4301	-1.2077492	0.14260013	-8.4694819	2.4649E-17	1.2294E-16
TCF21	ENSG00000118526	protein_coding	531.767858	-3.5395248	0.16613957	-21.304526	1.031E-100	1.5854E-98
SLC16A7	ENSG00000118596	protein_coding	2779.78757	-1.9089374	0.22036534	-8.6626026	4.6111E-18	2.4059E-17
OLFM3	ENSG00000118733	protein_coding	9.03708115	-5.4412361	0.37236549	-14.612622	2.3336E-48	5.1729E-47
PPL	ENSG00000118898	protein_coding	2750.80572	-1.524746	0.13084746	-11.652852	2.2191E-31	2.3515E-30
TRPM6	ENSG00000119121	protein_coding	142.624835	-3.0064505	0.16658231	-18.047837	8.2041E-73	4.3782E-71
ECRG4	ENSG00000119147	protein_coding	328.023605	-2.0591668	0.20143738	-10.222367	1.5745E-24	1.174E-23
CTNNAL1	ENSG00000119326	protein_coding	1711.69858	-1.4036625	0.09810981	-14.307055	1.9771E-46	4.0443E-45
BSPRY	ENSG00000119411	protein_coding	523.690149	-2.0683022	0.1996709	-10.358556	3.827E-25	2.9519E-24

NR4A3	ENSG00000119508	protein_coding	1372.91552	-1.2811024	0.18556676	-6.9037277	5.0655E-12	1.7711E-11
IRF2BPL	ENSG00000119669	protein_coding	2419.82111	-1.4740724	0.07620189	-19.344302	2.2766E-83	1.7771E-81
AREL1	ENSG00000119682	protein_coding	2121.47016	-1.1934116	0.06322571	-18.875417	1.8168E-79	1.2375E-77
PPP4R4	ENSG00000119698	protein_coding	22.5252457	-1.764757	0.24077081	-7.3296133	2.3082E-13	8.8662E-13
ALDH6A1	ENSG00000119711	protein_coding	6834.63126	-3.0617224	0.1359546	-22.520183	2.633E-112	5.916E-110
ESRRB	ENSG00000119715	protein_coding	461.127043	-5.9282099	0.21303321	-27.827633	2.009E-170	2.489E-167
EPCAM	ENSG00000119888	protein_coding	5117.19482	-2.8565563	0.15986313	-17.868762	2.0653E-71	1.0519E-69
ELOVL3	ENSG00000119915	protein_coding	13.4555913	-1.4511634	0.15232432	-9.5268003	1.6221E-21	1.0287E-20
GOT1	ENSG00000120053	protein_coding	6667.47212	-1.021342	0.11232897	-9.0924185	9.686E-20	5.5697E-19
C10orf95	ENSG00000120055	protein_coding	21.6089595	-1.3759054	0.11504815	-11.959387	5.7988E-33	6.6224E-32
HOXB8	ENSG00000120068	protein_coding	630.43353	-2.2292387	0.18603205	-11.98309	4.3577E-33	5.0129E-32
HOXB5	ENSG00000120075	protein_coding	297.807283	-1.9375957	0.13991984	-13.847898	1.3099E-43	2.3904E-42
HOXB1	ENSG00000120094	protein_coding	4.80370722	-2.2288207	0.38666427	-5.7642272	8.2033E-09	2.2426E-08
TEK	ENSG00000120156	protein_coding	2786.3223	-1.437027	0.12584681	-11.418859	3.3662E-30	3.3612E-29
INSL4	ENSG00000120211	protein_coding	1.10397561	-1.8477042	0.31039481	-5.9527549	2.6367E-09	7.5199E-09
GRIA2	ENSG00000120251	protein_coding	15.0093138	-1.4545475	0.33903569	-4.290249	1.7847E-05	3.6247E-05
KCNJ5	ENSG00000120457	protein_coding	937.269678	-1.3887054	0.16188117	-8.5785479	9.6078E-18	4.9095E-17
TP53AIP1	ENSG00000120471	protein_coding	17.5955399	-1.4143835	0.24212545	-5.8415315	5.1723E-09	1.4399E-08
ENOX1	ENSG00000120658	protein_coding	139.842483	-1.9335258	0.11921993	-16.218142	3.7538E-59	1.2389E-57
EPHX2	ENSG00000120915	protein_coding	4545.14573	-1.3902216	0.11413815	-12.180165	3.9648E-34	4.7866E-33
RDH10	ENSG00000121039	protein_coding	3111.972	-1.6422577	0.11144252	-14.736366	3.7651E-49	8.6168E-48
EPX	ENSG00000121053	protein_coding	17.3859804	-1.4760354	0.12260781	-12.038673	2.2251E-33	2.5901E-32
SCPEP1	ENSG00000121064	protein_coding	4186.6956	-1.1882057	0.08512722	-13.958	2.8122E-44	5.2574E-43
PDZRN3	ENSG00000121440	protein_coding	693.757422	-1.1930691	0.15104522	-7.8987544	2.817E-15	1.232E-14
GJA8	ENSG00000121634	protein_coding	3.34333975	-3.1527445	0.29027611	-10.861192	1.7643E-27	1.5405E-26

CAT	ENSG00000121691	protein_coding	11745.5161	-1.2718672	0.0975464	-13.038587	7.3812E-39	1.0964E-37
GJA3	ENSG00000121743	protein_coding	48.6956962	-2.9975782	0.22041968	-13.599413	4.0366E-42	6.8611E-41
SLITRK3	ENSG00000121871	protein_coding	2.76338451	-2.6614852	0.32714287	-8.1355442	4.1009E-16	1.8909E-15
RASL11A	ENSG00000122035	protein_coding	1146.19889	-1.0575828	0.10797658	-9.7945572	1.1882E-22	8.0086E-22
XPNPEP2	ENSG00000122121	protein_coding	1803.64912	-5.4050311	0.27951463	-19.337203	2.6127E-83	2.03E-81
TBX22	ENSG00000122145	protein_coding	0.8685127	-1.3006585	0.39838848	-3.2647996	0.00109542	0.00184133
PLG	ENSG00000122194	protein_coding	2457.99425	-3.2271526	0.38058452	-8.4794637	2.2623E-17	1.1319E-16
PTGFR	ENSG00000122420	protein_coding	309.678038	-1.6753937	0.20988695	-7.9823624	1.4356E-15	6.4104E-15
HOXA7	ENSG00000122592	protein_coding	201.934912	-1.4975557	0.14540793	-10.298996	7.12E-25	5.3993E-24
SPINK4	ENSG00000122711	protein_coding	3.04338244	-1.7829443	0.35796141	-4.9808281	6.3313E-07	1.4769E-06
ACO1	ENSG00000122729	protein_coding	6035.69883	-1.1081963	0.08903894	-12.446199	1.4667E-35	1.8982E-34
CNTFR	ENSG00000122756	protein_coding	67.976784	-1.3403974	0.27404072	-4.8912345	1.0021E-06	2.2919E-06
NUDT10	ENSG00000122824	protein_coding	48.8481769	-2.6870378	0.18067928	-14.871864	5.0196E-50	1.1904E-48
PLAU	ENSG00000122861	protein_coding	3828.18064	-2.1660428	0.14697371	-14.737621	3.6958E-49	8.4625E-48
EGR2	ENSG00000122877	protein_coding	1039.39003	-1.183477	0.18279389	-6.4743795	9.5202E-11	3.0377E-10
RASSF8	ENSG00000123094	protein_coding	2171.69026	-1.51842	0.08207568	-18.500243	2.0554E-76	1.2733E-74
ITPR2	ENSG00000123104	protein_coding	3680.68064	-1.2057118	0.12816373	-9.4075897	5.0764E-21	3.1308E-20
SARDH	ENSG00000123453	protein_coding	766.786017	-1.25313	0.14010428	-8.9442669	3.7443E-19	2.0813E-18
IL13RA2	ENSG00000123496	protein_coding	146.033816	-1.1485184	0.21272087	-5.3991807	6.6946E-08	1.6995E-07
PLP1	ENSG00000123560	protein_coding	30.6473958	-2.1127528	0.25856012	-8.1712246	3.0527E-16	1.4188E-15
NRK	ENSG00000123572	protein_coding	458.37268	-4.8094109	0.29192496	-16.474819	5.5655E-61	1.9446E-59
PFKFB2	ENSG00000123836	protein_coding	1889.53094	-1.7233482	0.17457713	-9.8715575	5.5299E-23	3.7824E-22
SLPI	ENSG00000124107	protein_coding	2263.40034	-1.5972995	0.34830042	-4.5859823	4.5186E-06	9.7297E-06
SEMG2	ENSG00000124157	protein_coding	12.6718343	-7.5971649	0.46480947	-16.344686	4.7461E-60	1.6128E-58
EDN3	ENSG00000124205	protein_coding	16.5549153	-1.3543439	0.38089619	-3.5556773	0.00037701	0.00066658

SEMG1	ENSG00000124233	protein_coding	1.47014858	-5.036829	0.39485826	-12.756043	2.8849E-37	4.0343E-36
C20orf85	ENSG00000124237	protein_coding	0.77520639	-2.6483176	0.62172705	-4.2596146	2.0478E-05	4.1367E-05
PCK1	ENSG00000124253	protein_coding	15226.0775	-2.8654675	0.25900535	-11.063353	1.889E-28	1.7322E-27
PEPD	ENSG00000124299	protein_coding	7689.67654	-1.8870736	0.10441174	-18.073385	5.1646E-73	2.7853E-71
LYPD3	ENSG00000124466	protein_coding	82.7598493	-1.3121731	0.15777571	-8.3166989	9.0447E-17	4.352E-16
CRISP2	ENSG00000124490	protein_coding	6.10406285	-4.678214	0.44767317	-10.450065	1.4642E-25	1.1573E-24
SLC17A1	ENSG00000124568	protein_coding	2176.82492	-1.1877739	0.21935826	-5.4147672	6.1368E-08	1.5626E-07
TCP11	ENSG00000124678	protein_coding	14.1170173	-1.3110809	0.16649752	-7.8744773	3.4217E-15	1.4875E-14
CRISP1	ENSG00000124812	protein_coding	0.51781216	-1.6426072	0.44736411	-3.6717457	0.0002409	0.00043521
SCGB1D2	ENSG00000124935	protein_coding	86.3006904	-1.4842717	0.22220631	-6.679701	2.3943E-11	7.9887E-11
SCGB2A1	ENSG00000124939	protein_coding	22.765887	-2.1781898	0.28882201	-7.5416337	4.6412E-14	1.872E-13
SSUH2	ENSG00000125046	protein_coding	33.1775186	-1.2363661	0.1568612	-7.8819116	3.2241E-15	1.4034E-14
MT1G	ENSG00000125144	protein_coding	3897.27361	-4.4587237	0.28743657	-15.512026	2.8766E-54	7.958E-53
GOT2	ENSG00000125166	protein_coding	6532.94338	-1.0789576	0.08068267	-13.372853	8.7133E-41	1.399E-39
DMAC2L	ENSG00000125375	protein_coding	2066.60657	-1.0270967	0.0748465	-13.722708	7.424E-43	1.3104E-41
HS3ST3B1	ENSG00000125430	protein_coding	300.355559	-1.2946864	0.10828439	-11.956353	6.0146E-33	6.8654E-32
SLC25A35	ENSG00000125434	protein_coding	320.646107	-1.5828531	0.09597033	-16.49315	4.1095E-61	1.4436E-59
FNDC11	ENSG00000125531	protein_coding	24.4399814	-2.0125009	0.17962098	-11.204153	3.8906E-29	3.7094E-28
CST11	ENSG00000125831	protein_coding	0.36184714	-2.3600329	0.50728155	-4.6523137	3.2823E-06	7.1575E-06
FLRT3	ENSG00000125848	protein_coding	2462.73744	-1.1767932	0.19625263	-5.996318	2.0184E-09	5.8156E-09
OVOL2	ENSG00000125850	protein_coding	31.0077117	-3.4667891	0.33148311	-10.458419	1.3407E-25	1.0619E-24
PCSK2	ENSG00000125851	protein_coding	10.4397272	-2.3698493	0.29108692	-8.1413801	3.908E-16	1.8037E-15
FAM83C	ENSG00000125998	protein_coding	1.63777735	-1.2703884	0.28881489	-4.398625	1.0894E-05	2.2602E-05
PROZ	ENSG00000126231	protein_coding	74.0325683	-4.1809053	0.27231859	-15.352993	3.3822E-53	9.0668E-52
KIRREL2	ENSG00000126259	protein_coding	21.7428913	-2.4504627	0.26527081	-9.2375889	2.5211E-20	1.4959E-19

PRRG2	ENSG00000126460	protein_coding	176.73384	-2.80461	0.18165477	-15.439231	8.9157E-54	2.4357E-52
FLRT1	ENSG00000126500	protein_coding	86.2967675	-4.1110914	0.21832375	-18.830253	4.2672E-79	2.8724E-77
WNK4	ENSG00000126562	protein_coding	977.085529	-4.0894162	0.17731821	-23.062584	1.1E-117	2.786E-115
DACH2	ENSG00000126733	protein_coding	21.1078558	-3.1997616	0.23558278	-13.582324	5.0983E-42	8.6272E-41
HSPA2	ENSG00000126803	protein_coding	3024.51117	-3.891516	0.14717147	-26.442054	4.503E-154	3.753E-151
SGPP1	ENSG00000126821	protein_coding	2220.67044	-1.2788588	0.08159639	-15.672981	2.3146E-55	6.6565E-54
PLEKHG3	ENSG00000126822	protein_coding	2784.08829	-1.0296021	0.08142587	-12.644657	1.1975E-36	1.6308E-35
PRDM7	ENSG00000126856	protein_coding	3.06493086	-2.2527039	0.2552929	-8.8239975	1.1044E-18	5.9796E-18
AIF1L	ENSG00000126878	protein_coding	15169.963	-3.2205474	0.1649717	-19.521818	7.1644E-85	5.9497E-83
AVPR2	ENSG00000126895	protein_coding	200.941079	-3.4188787	0.19023603	-17.971772	3.2419E-72	1.6888E-70
OMD	ENSG00000127083	protein_coding	107.086611	-1.0251923	0.2307467	-4.4429336	8.8741E-06	1.8557E-05
TSPAN8	ENSG00000127324	protein_coding	641.1903	-3.9080059	0.28797298	-13.570738	5.9718E-42	1.0083E-40
TRPV5	ENSG00000127412	protein_coding	35.4462873	-5.1317809	0.28152882	-18.228262	3.0796E-74	1.7385E-72
ECHS1	ENSG00000127884	protein_coding	10458.0231	-1.332123	0.08913093	-14.945687	1.6617E-50	4.0197E-49
FGL2	ENSG00000127951	protein_coding	6497.18074	-1.0078015	0.11576828	-8.7053338	3.1664E-18	1.6686E-17
GNAI1	ENSG00000127955	protein_coding	3046.16516	-1.2198298	0.08442284	-14.44905	2.5417E-47	5.3816E-46
RASL11B	ENSG00000128045	protein_coding	373.191232	-4.2679261	0.23117692	-18.461731	4.1968E-76	2.5685E-74
TST	ENSG00000128311	protein_coding	2533.29632	-1.0654206	0.12950105	-8.2271194	1.9177E-16	9.0265E-16
PODXL	ENSG00000128567	protein_coding	22163.3314	-1.3731886	0.12144922	-11.306689	1.2159E-29	1.1869E-28
HOXD1	ENSG00000128645	protein_coding	53.3634825	-1.2247414	0.15541481	-7.8804674	3.2616E-15	1.4194E-14
HOXD3	ENSG00000128652	protein_coding	165.228881	-1.100523	0.10163329	-10.828371	2.5261E-27	2.194E-26
HOXD10	ENSG00000128710	protein_coding	769.008837	-1.3346909	0.14235581	-9.3757389	6.8693E-21	4.2053E-20
SNRPN	ENSG00000128739	protein_coding	4147.89542	-1.2151393	0.07327171	-16.584016	9.0943E-62	3.2443E-60
TWSG1	ENSG00000128791	protein_coding	3984.76698	-1.1649251	0.08649953	-13.467416	2.4323E-41	4.0166E-40
MYO5C	ENSG00000128833	protein_coding	1155.01252	-1.2860015	0.08939517	-14.385582	6.374E-47	1.3275E-45

CGNL1	ENSG00000128849	protein_coding	8390.86402	-2.1204967	0.14667522	-14.457089	2.2616E-47	4.7998E-46
ALDH1A2	ENSG00000128918	protein_coding	1227.83963	-1.6720636	0.23935411	-6.9857319	2.8337E-12	1.0097E-11
CHAC1	ENSG00000128965	protein_coding	183.394458	-1.9178302	0.18067115	-10.615032	2.5371E-26	2.0865E-25
SHBG	ENSG00000129214	protein_coding	34.9379316	-2.3349877	0.13996323	-16.682866	1.7464E-62	6.3789E-61
AP1M2	ENSG00000129354	protein_coding	1306.85326	-2.461664	0.17701872	-13.906235	5.8056E-44	1.0731E-42
NRL	ENSG00000129535	protein_coding	104.364779	-1.166125	0.08871438	-13.144712	1.825E-39	2.7914E-38
CDO1	ENSG00000129596	protein_coding	78.1419481	-1.9283162	0.18909677	-10.19751	2.0342E-24	1.5101E-23
FOXJ1	ENSG00000129654	protein_coding	83.3164348	-3.1738768	0.27936273	-11.361132	6.5293E-30	6.4451E-29
CDKN1C	ENSG00000129757	protein_coding	1673.95101	-1.52731	0.12737379	-11.990772	3.9719E-33	4.5771E-32
LDLR	ENSG00000130164	protein_coding	1402.96952	-1.2592565	0.16885394	-7.4576675	8.8068E-14	3.4854E-13
DPP6	ENSG00000130226	protein_coding	214.089038	-3.3780445	0.28943178	-11.671298	1.7868E-31	1.9004E-30
ACSBG2	ENSG00000130377	protein_coding	23.6950203	-1.8370078	0.14886061	-12.340456	5.4844E-35	6.8918E-34
STK33	ENSG00000130413	protein_coding	285.436885	-1.6275753	0.23118318	-7.0401978	1.9197E-12	6.9264E-12
GDF15	ENSG00000130513	protein_coding	4929.84451	-1.1608018	0.15978341	-7.2648454	3.7347E-13	1.413E-12
GATA5	ENSG00000130700	protein_coding	41.7329478	-2.170178	0.22226177	-9.7640633	1.6059E-22	1.0728E-21
RBBP8NL	ENSG00000130701	protein_coding	16.802553	-3.2175924	0.48233311	-6.6708926	2.5425E-11	8.4685E-11
ASS1	ENSG00000130707	protein_coding	13863.6369	-2.7380034	0.14187808	-19.298284	5.5523E-83	4.2491E-81
DUSP9	ENSG00000130829	protein_coding	946.701601	-6.8150304	0.29477183	-23.11968	2.936E-118	7.646E-116
ZNF331	ENSG00000130844	protein_coding	1629.19099	-1.2068748	0.09687364	-12.458237	1.2613E-35	1.6375E-34
SLC7A10	ENSG00000130876	protein_coding	19.279325	-1.4924477	0.28809194	-5.1804563	2.2134E-07	5.3711E-07
CASZ1	ENSG00000130940	protein_coding	397.209011	-2.0335507	0.10822656	-18.789757	9.1599E-79	6.1299E-77
FBP2	ENSG00000130957	protein_coding	3.91083987	-1.517302	0.22506022	-6.7417601	1.5648E-11	5.2872E-11
PRRG1	ENSG00000130962	protein_coding	1329.37194	-1.2708636	0.08968355	-14.170533	1.3944E-45	2.7469E-44
RGN	ENSG00000130988	protein_coding	1092.10953	-1.4450503	0.12253816	-11.792656	4.259E-32	4.6673E-31
SYNE1	ENSG00000131018	protein_coding	8640.69568	-1.0047737	0.0897372	-11.196848	4.225E-29	4.0182E-28

EPS8L1	ENSG00000131037	protein_coding	589.854963	-1.5442073	0.21728028	-7.1069831	1.1861E-12	4.3434E-12
SLC34A1	ENSG00000131183	protein_coding	1729.18183	-4.4414015	0.33015016	-13.452671	2.9695E-41	4.8825E-40
RAB11FIP4	ENSG00000131242	protein_coding	1201.29765	-1.9599831	0.10273093	-19.078802	3.7884E-81	2.7566E-79
NAPSA	ENSG00000131400	protein_coding	1785.95713	-1.1663371	0.22822373	-5.1104987	3.2131E-07	7.6903E-07
G6PC	ENSG00000131482	protein_coding	1207.46849	-3.530568	0.30038583	-11.753444	6.7798E-32	7.3561E-31
TNS4	ENSG00000131746	protein_coding	90.7910616	-2.2646445	0.22959316	-9.8637284	5.9787E-23	4.0796E-22
PPP1R1B	ENSG00000131771	protein_coding	77.2977049	-3.7519361	0.30025933	-12.495652	7.8847E-36	1.0309E-34
FMO5	ENSG00000131781	protein_coding	719.742766	-2.4523606	0.14646075	-16.744149	6.2477E-63	2.3134E-61
FSHB	ENSG00000131808	protein_coding	0.5280803	-3.6417029	0.48244374	-7.5484508	4.4047E-14	1.7784E-13
PDHA1	ENSG00000131828	protein_coding	5167.12317	-1.1545986	0.07770034	-14.859634	6.0254E-50	1.4274E-48
ZNF132	ENSG00000131849	protein_coding	309.593692	-1.011112	0.08652942	-11.685182	1.5175E-31	1.622E-30
NR0B2	ENSG00000131910	protein_coding	219.688016	-2.7096644	0.5199838	-5.2110553	1.8777E-07	4.5885E-07
GCH1	ENSG00000131979	protein_coding	754.593904	-1.1287509	0.09448711	-11.946083	6.8058E-33	7.7434E-32
RAP1GAP2	ENSG00000132359	protein_coding	1249.71109	-1.2501969	0.11922242	-10.486257	9.9908E-26	7.9733E-25
DDC	ENSG00000132437	protein_coding	4749.3436	-1.7394757	0.21732117	-8.0041705	1.2027E-15	5.3943E-15
ENAM	ENSG00000132464	protein_coding	592.465158	-1.7377671	0.1999264	-8.6920339	3.5601E-18	1.869E-17
MATN2	ENSG00000132561	protein_coding	3027.39975	-1.1393811	0.12186796	-9.3493079	8.8223E-21	5.3694E-20
RHBG	ENSG00000132677	protein_coding	365.697123	-2.0231139	0.44835708	-4.5122826	6.4134E-06	1.3601E-05
RAB25	ENSG00000132698	protein_coding	477.6576	-3.4284688	0.34794411	-9.8535044	6.6195E-23	4.5108E-22
ALDH3B2	ENSG00000132746	protein_coding	25.9482612	-3.883524	0.31340317	-12.391463	2.9069E-35	3.7067E-34
ANGPTL3	ENSG00000132855	protein_coding	831.638175	-1.7695498	0.28199682	-6.2750699	3.4948E-10	1.0693E-09
SYT4	ENSG00000132872	protein_coding	4.21923782	-1.6015809	0.33114699	-4.836465	1.3217E-06	2.9892E-06
SLC14A2	ENSG00000132874	protein_coding	468.4272	-6.3910899	0.29730549	-21.49671	1.671E-102	2.806E-100
NMUR2	ENSG00000132911	protein_coding	9.0257992	-3.4260127	0.29332096	-11.680081	1.6114E-31	1.7187E-30
WASF3	ENSG00000132970	protein_coding	649.506761	-1.5564452	0.10647256	-14.618275	2.1477E-48	4.7747E-47

GPR12	ENSG00000132975	protein_coding	1.92282427	-3.5648433	0.30759469	-11.589418	4.6629E-31	4.858E-30
MYH10	ENSG00000133026	protein_coding	6205.69067	-1.7454643	0.11771812	-14.82749	9.7303E-50	2.2804E-48
MYBPH	ENSG00000133055	protein_coding	17.1818695	-3.2175776	0.21247621	-15.143237	8.3977E-52	2.141E-50
LGR6	ENSG00000133067	protein_coding	67.5250758	-1.5999587	0.13553899	-11.804417	3.7035E-32	4.0737E-31
TMCC2	ENSG00000133069	protein_coding	227.683121	-1.0550444	0.1004986	-10.498101	8.8136E-26	7.0547E-25
KL	ENSG00000133116	protein_coding	6612.77296	-1.6375505	0.16024958	-10.218751	1.6343E-24	1.2174E-23
IRS4	ENSG00000133124	protein_coding	1.02250291	-2.9329833	0.37322376	-7.8585118	3.8872E-15	1.6843E-14
BEX2	ENSG00000133134	protein_coding	1058.05877	-1.0383699	0.15468234	-6.7129182	1.9077E-11	6.4076E-11
EPHB2	ENSG00000133216	protein_coding	529.935712	-1.0190007	0.15772657	-6.460552	1.0432E-10	3.3182E-10
PLAAT2	ENSG00000133328	protein_coding	79.2238408	-2.0743122	0.17387673	-11.929786	8.2786E-33	9.3726E-32
FAM83F	ENSG00000133477	protein_coding	575.569249	-1.0311016	0.29287685	-3.5205978	0.00043058	0.00075658
NTS	ENSG00000133636	protein_coding	85.4461022	-1.0935475	0.23827988	-4.5893403	4.4465E-06	9.5808E-06
DUSP26	ENSG00000133878	protein_coding	46.0195011	-2.6854975	0.22593345	-11.886232	1.3956E-32	1.5635E-31
MRO	ENSG00000134042	protein_coding	524.218943	-2.9999783	0.15802635	-18.984039	2.3113E-80	1.6351E-78
CHL1	ENSG00000134121	protein_coding	895.766488	-3.3330059	0.23496741	-14.184971	1.1351E-45	2.2526E-44
REG4	ENSG00000134193	protein_coding	3.82396934	-1.0142558	0.28721446	-3.5313535	0.00041344	0.00072785
GSTM5	ENSG00000134201	protein_coding	145.864379	-1.2489315	0.18440112	-6.7729065	1.2622E-11	4.2917E-11
GSTM3	ENSG00000134202	protein_coding	3365.74282	-3.3152713	0.13300016	-24.926822	3.81E-137	1.838E-134
VAV3	ENSG00000134215	protein_coding	5215.8249	-1.3948216	0.0985534	-14.152953	1.7908E-45	3.5112E-44
CHIA	ENSG00000134216	protein_coding	6.73120493	-1.3316943	0.30870399	-4.3138227	1.6046E-05	3.2724E-05
HMGCS2	ENSG00000134240	protein_coding	4242.48383	-2.6455028	0.29408819	-8.9956104	2.3492E-19	1.3219E-18
SORT1	ENSG00000134243	protein_coding	8112.01482	-1.5291031	0.0873603	-17.503409	1.3494E-68	6.1798E-67
VTCN1	ENSG00000134258	protein_coding	589.77853	-3.4294537	0.3239037	-10.587881	3.3918E-26	2.7731E-25
GRHL1	ENSG00000134317	protein_coding	221.124367	-1.3378907	0.12669491	-10.559941	4.5695E-26	3.712E-25
MYCN	ENSG00000134323	protein_coding	72.7463387	-1.9274712	0.15413597	-12.505006	7.0094E-36	9.1884E-35

CFHR4	ENSG00000134365	protein_coding	3.10385917	-1.9112115	0.39313052	-4.8615189	1.1649E-06	2.6481E-06
KCTD1	ENSG00000134504	protein_coding	936.743918	-1.9509781	0.1311549	-14.875373	4.7632E-50	1.1308E-48
FHOD3	ENSG00000134775	protein_coding	1869.19216	-1.243738	0.11388743	-10.920766	9.1719E-28	8.1388E-27
PDGFRA	ENSG00000134853	protein_coding	1216.07925	-2.3715799	0.2296494	-10.326959	5.3221E-25	4.0648E-24
GGACT	ENSG00000134864	protein_coding	697.486506	-2.3909007	0.15463617	-15.461459	6.3152E-54	1.7294E-52
CLDN10	ENSG00000134873	protein_coding	2212.99602	-2.308263	0.14126239	-16.340251	5.1042E-60	1.7281E-58
ADAMTS8	ENSG00000134917	protein_coding	90.6933715	-1.4144351	0.148196	-9.5443539	1.3696E-21	8.7211E-21
PSAT1	ENSG00000135069	protein_coding	1664.55201	-2.5102466	0.2348146	-10.690334	1.1296E-26	9.4789E-26
FBXO21	ENSG00000135108	protein_coding	4820.1848	-1.064441	0.07995325	-13.313292	1.9375E-40	3.0797E-39
TBX3	ENSG00000135111	protein_coding	785.189122	-1.8412317	0.12984037	-14.180734	1.2058E-45	2.3856E-44
BICDL1	ENSG00000135127	protein_coding	3135.6182	-2.0285535	0.14655612	-13.841479	1.4323E-43	2.6096E-42
DTX1	ENSG00000135144	protein_coding	414.067135	-2.4539773	0.14016011	-17.508386	1.2365E-68	5.6683E-67
ADGRB3	ENSG00000135298	protein_coding	57.9861674	-1.7135159	0.18894643	-9.068792	1.2034E-19	6.8821E-19
MRAP2	ENSG00000135324	protein_coding	75.461521	-3.3518971	0.22624165	-14.815562	1.1621E-49	2.7139E-48
CGA	ENSG00000135346	protein_coding	18.9306809	-1.5229337	0.4688726	-3.2480756	0.00116188	0.00194778
EHF	ENSG00000135373	protein_coding	694.715729	-5.2194413	0.28627324	-18.232376	2.8564E-74	1.6145E-72
ELF5	ENSG00000135374	protein_coding	355.50719	-7.8309376	0.29962349	-26.135927	1.425E-150	1.053E-147
GLS2	ENSG00000135423	protein_coding	31.8315388	-1.0749091	0.12083819	-8.8954419	5.8187E-19	3.2033E-18
KRT85	ENSG00000135443	protein_coding	3.37870017	-1.9986081	0.22873057	-8.7378267	2.3764E-18	1.2602E-17
PPP1R1A	ENSG00000135447	protein_coding	2937.53656	-2.6616181	0.32150952	-8.278505	1.2473E-16	5.9536E-16
FAIM2	ENSG00000135472	protein_coding	89.2621244	-1.5795272	0.17959195	-8.795089	1.4293E-18	7.6905E-18
KRT7	ENSG00000135480	protein_coding	3363.1253	-1.6020721	0.2844993	-5.6311988	1.7896E-08	4.7613E-08
KCNH3	ENSG00000135519	protein_coding	60.6733074	-1.9732112	0.19771114	-9.9802735	1.8595E-23	1.3054E-22
NHSL1	ENSG00000135540	protein_coding	1391.10943	-1.2567697	0.08668294	-14.498467	1.2389E-47	2.6525E-46
EGR4	ENSG00000135625	protein_coding	7.44566028	-1.7379007	0.29137943	-5.9643903	2.4555E-09	7.0228E-09

EMX1	ENSG00000135638	protein_coding	1176.71955	-2.6281525	0.13213399	-19.890056	4.962E-88	4.6326E-86
RGS8	ENSG00000135824	protein_coding	6.1094758	-1.217886	0.27232981	-4.4720996	7.7455E-06	1.629E-05
ITM2C	ENSG00000135916	protein_coding	9594.0095	-1.402421	0.11103179	-12.630806	1.4281E-36	1.9357E-35
WNT10A	ENSG00000135925	protein_coding	119.133909	-1.2438267	0.19819303	-6.275835	3.4776E-10	1.0643E-09
TSGA10	ENSG00000135951	protein_coding	250.783466	-1.0568682	0.08486769	-12.453128	1.3447E-35	1.7443E-34
EDAR	ENSG00000135960	protein_coding	71.3309366	-1.199044	0.23513546	-5.0993754	3.4078E-07	8.1371E-07
USP44	ENSG00000136014	protein_coding	48.3762565	-3.0303094	0.13409779	-22.597758	4.559E-113	1.05E-110
TBC1D4	ENSG00000136111	protein_coding	3964.63624	-1.4548099	0.1039701	-13.992579	1.7302E-44	3.26E-43
SUCLA2	ENSG00000136143	protein_coding	2781.46303	-1.0418523	0.07438207	-14.006766	1.4171E-44	2.6789E-43
LMO7	ENSG00000136153	protein_coding	4489.74452	-1.2203647	0.0989809	-12.329295	6.2995E-35	7.8944E-34
SCRN1	ENSG00000136193	protein_coding	5571.50293	-1.0355311	0.12689151	-8.1607602	3.3292E-16	1.543E-15
DGKB	ENSG00000136267	protein_coding	53.3861469	-1.4756039	0.16159857	-9.1312933	6.7686E-20	3.9326E-19
ZFHX2	ENSG00000136367	protein_coding	202.46893	-1.7544537	0.09444183	-18.577084	4.9256E-77	3.123E-75
ABHD17C	ENSG00000136379	protein_coding	664.893902	-2.0426868	0.10288888	-19.85333	1.0313E-87	9.4936E-86
SCN2A	ENSG00000136531	protein_coding	161.478274	-3.0293574	0.24532213	-12.348488	4.9635E-35	6.2475E-34
SCN7A	ENSG00000136546	protein_coding	55.0382379	-4.0011095	0.28884671	-13.852017	1.2369E-43	2.2581E-42
IL36RN	ENSG00000136695	protein_coding	0.74121594	-1.0051616	0.3618652	-2.7777238	0.00547411	0.00848368
HS6ST1	ENSG00000136720	protein_coding	2761.27788	-2.1644344	0.08515051	-25.418925	1.558E-142	8.025E-140
RALGPS1	ENSG00000136828	protein_coding	572.924183	-1.913656	0.14025795	-13.643832	2.1971E-42	3.7906E-41
ALDOB	ENSG00000136872	protein_coding	37884.0885	-4.7373217	0.3200416	-14.802206	1.4176E-49	3.2936E-48
CTSV	ENSG00000136943	protein_coding	177.710715	-2.8762108	0.19128317	-15.036402	4.2397E-51	1.046E-49
LMX1B	ENSG00000136944	protein_coding	105.704585	-4.0981833	0.35430683	-11.566763	6.0731E-31	6.2829E-30
ALDH1B1	ENSG00000137124	protein_coding	3007.03524	-1.2849414	0.13341185	-9.6313893	5.8927E-22	3.8289E-21
GMPR	ENSG00000137198	protein_coding	725.259147	-2.288946	0.16832683	-13.598225	4.1027E-42	6.9708E-41
TFAP2A	ENSG00000137203	protein_coding	431.38464	-2.0376618	0.24656804	-8.2640953	1.4076E-16	6.6907E-16

SLC22A7	ENSG00000137204	protein_coding	527.859504	-3.1567429	0.3045245	-10.366138	3.5353E-25	2.731E-24
HCRTR2	ENSG00000137252	protein_coding	4.72412404	-5.867876	0.30155246	-19.45889	2.4504E-84	1.9916E-82
GCM1	ENSG00000137270	protein_coding	36.5785802	-2.0194032	0.2502594	-8.0692404	7.0737E-16	3.2144E-15
BPHL	ENSG00000137274	protein_coding	1495.02746	-1.6097503	0.09038479	-17.809968	5.9144E-71	2.9696E-69
TUBB2B	ENSG00000137285	protein_coding	226.639917	-2.6009967	0.18990172	-13.69654	1.0648E-42	1.8644E-41
C6orf52	ENSG00000137434	protein_coding	24.9053507	-1.8710185	0.10227855	-18.293363	9.3467E-75	5.3827E-73
FGFBP1	ENSG00000137440	protein_coding	14.4122801	-3.0015763	0.41600959	-7.2151613	5.387E-13	2.0162E-12
TTC29	ENSG00000137473	protein_coding	6.42587823	-2.4094049	0.22571763	-10.674421	1.3409E-26	1.1219E-25
TTPA	ENSG00000137561	protein_coding	17.7956268	-3.3120629	0.28933691	-11.447081	2.432E-30	2.4475E-29
GGH	ENSG00000137563	protein_coding	1208.03023	-1.447417	0.1258183	-11.504026	1.259E-30	1.2834E-29
SORL1	ENSG00000137642	protein_coding	11004.7508	-1.0710021	0.12572579	-8.5185551	1.6156E-17	8.1437E-17
TMPRSS4	ENSG00000137648	protein_coding	337.941068	-3.7205263	0.31377804	-11.857191	1.9749E-32	2.1979E-31
TRIM29	ENSG00000137699	protein_coding	231.075796	-1.0613536	0.26119253	-4.0634917	4.8344E-05	9.4112E-05
POU2F3	ENSG00000137709	protein_coding	47.2129404	-2.098006	0.20095062	-10.440406	1.6212E-25	1.2782E-24
FDX1	ENSG00000137714	protein_coding	1656.142	-1.1257899	0.07067577	-15.928936	3.9909E-57	1.2229E-55
TMPRSS13	ENSG00000137747	protein_coding	67.8199067	-1.7061053	0.17839984	-9.5633794	1.1397E-21	7.2909E-21
UNC13C	ENSG00000137766	protein_coding	68.7306997	-2.0233926	0.30515186	-6.6307724	3.3393E-11	1.1036E-10
PAQR5	ENSG00000137819	protein_coding	3352.33798	-1.7853243	0.1136483	-15.709204	1.3081E-55	3.8025E-54
PAK6	ENSG00000137843	protein_coding	13.6249633	-3.3567659	0.27505357	-12.204044	2.9576E-34	3.5906E-33
SLC28A2	ENSG00000137860	protein_coding	47.9616764	-3.5507179	0.18275514	-19.428826	4.4032E-84	3.5226E-82
STRA6	ENSG00000137868	protein_coding	197.092754	-4.0451813	0.24107114	-16.780032	3.4165E-63	1.2827E-61
SEMA6D	ENSG00000137872	protein_coding	1096.2359	-2.94466	0.1455861	-20.226245	5.752E-91	5.9791E-89
BCL2L10	ENSG00000137875	protein_coding	78.5319367	-1.6551183	0.22403421	-7.3877925	1.4929E-13	5.8207E-13
GCOM1	ENSG00000137878	protein_coding	32.5380339	-1.3815357	0.3151006	-4.3844272	1.1629E-05	2.405E-05
BCAR3	ENSG00000137936	protein_coding	1401.03517	-1.0168199	0.07122296	-14.276574	3.0629E-46	6.199E-45

DBT	ENSG00000137992	protein_coding	2281.5359	-1.5566167	0.06383862	-24.383622	2.552E-131	9.998E-129
CYP1B1	ENSG00000138061	protein_coding	3776.01006	-1.3057353	0.18486587	-7.0631494	1.6277E-12	5.9083E-12
SIX3	ENSG00000138083	protein_coding	5.05560665	-1.4374255	0.30309604	-4.7424752	2.1112E-06	4.6849E-06
LOXL4	ENSG00000138131	protein_coding	873.090806	-1.1514861	0.13445841	-8.5638836	1.0913E-17	5.5565E-17
CH25H	ENSG00000138135	protein_coding	125.703725	-1.3079654	0.16058143	-8.1451845	3.787E-16	1.7497E-15
AOX1	ENSG00000138356	protein_coding	5450.76363	-1.4951552	0.20477667	-7.3013939	2.848E-13	1.0865E-12
GUCA1C	ENSG00000138472	protein_coding	5.7990187	-3.1820425	0.41231997	-7.7174107	1.1872E-14	4.98E-14
MNS1	ENSG00000138587	protein_coding	464.933667	-1.054806	0.11214664	-9.4055969	5.1736E-21	3.1881E-20
CILP	ENSG00000138615	protein_coding	112.634056	-1.6569475	0.2009897	-8.2439424	1.6663E-16	7.8803E-16
HCN4	ENSG00000138622	protein_coding	24.4777706	-1.329012	0.21742436	-6.1125258	9.8066E-10	2.8981E-09
ARHGAP24	ENSG00000138639	protein_coding	4656.85153	-1.643397	0.08640599	-19.019479	1.1765E-80	8.4003E-79
GPAT3	ENSG00000138678	protein_coding	1847.26144	-2.4754976	0.19349208	-12.793793	1.7759E-37	2.5041E-36
BMPR1B	ENSG00000138696	protein_coding	829.317611	-2.0194475	0.34683731	-5.8224633	5.7987E-09	1.6057E-08
FRAS1	ENSG00000138759	protein_coding	3385.24931	-1.2853678	0.15546278	-8.2680098	1.3621E-16	6.48E-16
CDKL2	ENSG00000138769	protein_coding	397.855784	-1.5959732	0.11895975	-13.416078	4.8681E-41	7.933E-40
SHROOM3	ENSG00000138771	protein_coding	2031.15716	-1.8638719	0.1581928	-11.78228	4.8172E-32	5.269E-31
ANXA3	ENSG00000138772	protein_coding	839.803386	-2.0017527	0.20856635	-9.5976779	8.1766E-22	5.2725E-21
HADH	ENSG00000138796	protein_coding	3600.26402	-1.7855534	0.08410435	-21.230216	5.023E-100	7.452E-98
EGF	ENSG00000138798	protein_coding	3672.88485	-5.4207386	0.24386561	-22.228385	1.826E-109	3.839E-107
MTTP	ENSG00000138823	protein_coding	286.841241	-1.7305217	0.31452218	-5.5020658	3.7537E-08	9.7117E-08
B4GALNT3	ENSG00000139044	protein_coding	602.295681	-2.2089698	0.20846712	-10.59625	3.1017E-26	2.5386E-25
ERP27	ENSG00000139055	protein_coding	1047.57582	-1.4493425	0.28058959	-5.1653467	2.3999E-07	5.8062E-07
GABARAPL1	ENSG00000139112	protein_coding	8613.96909	-1.5184604	0.11998903	-12.654994	1.0498E-36	1.4332E-35
PIK3C2G	ENSG00000139144	protein_coding	176.612559	-4.3583729	0.41027007	-10.62318	2.325E-26	1.9176E-25
PLCZ1	ENSG00000139151	protein_coding	4.83908066	-2.3787918	0.23574544	-10.09051	6.0852E-24	4.3833E-23

SLC38A4	ENSG00000139209	protein_coding	360.734449	-1.7033422	0.28254293	-6.0286138	1.6537E-09	4.7965E-09
LGR5	ENSG00000139292	protein_coding	58.1402511	-2.5838014	0.31320833	-8.2494658	1.591E-16	7.5416E-16
PTPRQ	ENSG00000139304	protein_coding	24.0004621	-3.972558	0.31597904	-12.57222	3.0017E-36	4.0071E-35
LUM	ENSG00000139329	protein_coding	5926.86624	-1.2818972	0.24484556	-5.235534	1.6451E-07	4.0377E-07
FAM222A	ENSG00000139438	protein_coding	291.313373	-2.7436106	0.15957555	-17.193176	2.9871E-66	1.2505E-64
FOXN4	ENSG00000139445	protein_coding	9.57144878	-1.4908572	0.25973827	-5.739844	9.4764E-09	2.5781E-08
PDX1	ENSG00000139515	protein_coding	0.77496361	-2.7045729	0.4027603	-6.7150932	1.8795E-11	6.3146E-11
SUOX	ENSG00000139531	protein_coding	2446.4936	-1.076022	0.06293734	-17.096718	1.57E-65	6.4088E-64
GALNT6	ENSG00000139629	protein_coding	532.936122	-1.1713759	0.10164867	-11.52377	1.0013E-30	1.0241E-29
PELI2	ENSG00000139946	protein_coding	922.251565	-1.2440036	0.09482997	-13.118253	2.5883E-39	3.9341E-38
ARMH4	ENSG00000139971	protein_coding	635.878954	-3.5620502	0.15125045	-23.550675	1.236E-122	3.726E-120
RDH12	ENSG00000139988	protein_coding	192.72738	-2.0835135	0.22791275	-9.1417153	6.1469E-20	3.5786E-19
RAB15	ENSG00000139998	protein_coding	800.688722	-1.0261183	0.10933169	-9.3853698	6.2696E-21	3.8439E-20
KCNH5	ENSG00000140015	protein_coding	3.75728118	-1.1825882	0.37287862	-3.1715097	0.00151649	0.00250829
AK7	ENSG00000140057	protein_coding	311.438667	-1.5222204	0.11382129	-13.373776	8.6059E-41	1.3832E-39
FBLN5	ENSG00000140092	protein_coding	4556.05152	-1.7358089	0.16300759	-10.648639	1.7693E-26	1.472E-25
SLC25A47	ENSG00000140107	protein_coding	9.5280712	-2.4241106	0.19635362	-12.345638	5.1425E-35	6.4657E-34
SORD	ENSG00000140263	protein_coding	1610.21566	-2.195791	0.12217571	-17.972402	3.2053E-72	1.6716E-70
DUOXA2	ENSG00000140274	protein_coding	3.3897434	-1.062197	0.30241158	-3.5124217	0.00044404	0.00077921
SLC27A2	ENSG00000140284	protein_coding	2796.98705	-1.1074698	0.18651829	-5.9375936	2.8924E-09	8.2215E-09
FGF7	ENSG00000140285	protein_coding	371.102165	-1.0315278	0.26409247	-3.9059343	9.3862E-05	0.00017723
GCNT3	ENSG00000140297	protein_coding	1159.45055	-1.755613	0.18183939	-9.6547452	4.6932E-22	3.063E-21
CYP1A1	ENSG00000140465	protein_coding	42.2472724	-2.9117923	0.43229171	-6.7357117	1.6313E-11	5.5034E-11
CYP1A2	ENSG00000140505	protein_coding	9.36664694	-1.0545566	0.40201621	-2.6231694	0.00871159	0.01314683
RHCG	ENSG00000140519	protein_coding	3785.44675	-1.6810621	0.4701806	-3.575354	0.00034975	0.00062057

SH3GL3	ENSG00000140600	protein_coding	50.9245545	-5.6356635	0.27696979	-20.347575	4.8782E-92	5.3498E-90
SLC5A2	ENSG00000140675	protein_coding	337.626521	-4.7308373	0.25620204	-18.46526	3.9313E-76	2.4093E-74
MYLK3	ENSG00000140795	protein_coding	94.0909361	-4.0672487	0.14821349	-27.441826	8.697E-166	9.493E-163
NKD1	ENSG00000140807	protein_coding	115.613861	-2.9217347	0.17488583	-16.706526	1.1748E-62	4.3188E-61
MARVELD3	ENSG00000140832	protein_coding	577.437545	-1.4501305	0.14772655	-9.8163158	9.578E-23	6.4797E-22
ADAD2	ENSG00000140955	protein_coding	6.34509739	-1.3730935	0.21424472	-6.4089955	1.4648E-10	4.6049E-10
ABCA8	ENSG00000141338	protein_coding	571.388589	-1.9873291	0.17332872	-11.465665	1.9625E-30	1.9841E-29
SLC14A1	ENSG00000141469	protein_coding	1075.23753	-2.9897925	0.17341943	-17.240239	1.3249E-66	5.6079E-65
SLC13A5	ENSG00000141485	protein_coding	20.1703036	-1.7201262	0.26616206	-6.4627023	1.0285E-10	3.2743E-10
SAT2	ENSG00000141504	protein_coding	3095.05523	-1.1578435	0.07692448	-15.05169	3.3652E-51	8.3613E-50
ZNF750	ENSG00000141579	protein_coding	33.5761756	-1.2339422	0.17943956	-6.8766452	6.1278E-12	2.1316E-11
MAPK4	ENSG00000141639	protein_coding	205.370372	-4.0768585	0.34843051	-11.700636	1.265E-31	1.3568E-30
CBLN2	ENSG00000141668	protein_coding	38.7709572	-3.0873377	0.26600568	-11.606285	3.8289E-31	4.0028E-30
ERBB2	ENSG00000141736	protein_coding	9244.12095	-1.4750265	0.08704371	-16.945812	2.0666E-64	8.1108E-63
GRB7	ENSG00000141738	protein_coding	1205.15532	-1.2254268	0.10712646	-11.439068	2.6673E-30	2.6773E-29
PNMT	ENSG00000141744	protein_coding	16.3977477	-2.4606231	0.27376269	-8.9881609	2.514E-19	1.4123E-18
MISP3	ENSG00000141854	protein_coding	457.127729	-1.6817742	0.13077562	-12.859998	7.5579E-38	1.0807E-36
HUNK	ENSG00000142149	protein_coding	854.856923	-1.7287091	0.13612823	-12.699123	5.9796E-37	8.2415E-36
DNMT3L	ENSG00000142182	protein_coding	4.82016404	-3.0220212	0.28621302	-10.558643	4.6331E-26	3.7617E-25
IL19	ENSG00000142224	protein_coding	2.00095031	-2.9577755	0.249724	-11.844178	2.3067E-32	2.5622E-31
LMTK3	ENSG00000142235	protein_coding	183.35953	-1.4725977	0.20060895	-7.340638	2.1258E-13	8.1882E-13
CBLC	ENSG00000142273	protein_coding	130.311808	-1.9419956	0.22051654	-8.8065759	1.2903E-18	6.9609E-18
MMEL1	ENSG00000142606	protein_coding	32.2171642	-1.4119148	0.19616095	-7.1977362	6.122E-13	2.2844E-12
PRDM16	ENSG00000142611	protein_coding	485.363708	-3.6878225	0.18738571	-19.680383	3.1759E-86	2.789E-84
CNKSR1	ENSG00000142675	protein_coding	175.794915	-2.5637583	0.22129654	-11.585171	4.8999E-31	5.0979E-30

CYP4B1	ENSG00000142973	protein_coding	72.5853964	-1.5981116	0.18944565	-8.4357262	3.2915E-17	1.6286E-16
TMEM61	ENSG00000143001	protein_coding	124.741706	-2.8067781	0.38962615	-7.2037723	5.8569E-13	2.1878E-12
SYPL2	ENSG00000143028	protein_coding	632.708275	-1.3373612	0.12807828	-10.441749	1.5984E-25	1.2607E-24
IGSF3	ENSG00000143061	protein_coding	2985.9825	-1.1549621	0.09843972	-11.732683	8.6667E-32	9.3568E-31
ATP1B1	ENSG00000143153	protein_coding	65622.4213	-1.3667496	0.09016361	-15.158551	6.6521E-52	1.7055E-50
DPT	ENSG00000143196	protein_coding	316.748219	-2.1774185	0.2361891	-9.2189624	2.9999E-20	1.771E-19
NECTIN4	ENSG00000143217	protein_coding	150.033086	-1.9102983	0.25244121	-7.5672995	3.8106E-14	1.5437E-13
NR1I3	ENSG00000143257	protein_coding	67.7549966	-2.4647703	0.16588232	-14.858547	6.1239E-50	1.4493E-48
TUFT1	ENSG00000143367	protein_coding	1624.72426	-1.0313266	0.09862587	-10.456958	1.3616E-25	1.0778E-24
CGN	ENSG00000143375	protein_coding	1468.31526	-2.4379884	0.16459327	-14.8122	1.2217E-49	2.8516E-48
ANXA9	ENSG00000143412	protein_coding	800.419703	-1.9258921	0.09697427	-19.859826	9.0623E-88	8.3924E-86
SELENBP1	ENSG00000143416	protein_coding	2713.99432	-1.7249632	0.10161457	-16.97555	1.2458E-64	4.9317E-63
KCNH1	ENSG00000143473	protein_coding	14.8889954	-1.8402012	0.19940039	-9.2286743	2.74E-20	1.6224E-19
SUSD4	ENSG00000143502	protein_coding	518.460137	-3.6500118	0.21923399	-16.648932	3.0805E-62	1.1199E-60
SCCPDH	ENSG00000143653	protein_coding	3585.50902	-1.3337287	0.07975759	-16.72228	9.0201E-63	3.3319E-61
LEFTY2	ENSG00000143768	protein_coding	20.3908172	-1.2106232	0.25346655	-4.7762642	1.7858E-06	3.9893E-06
MBOAT2	ENSG00000143797	protein_coding	1388.52065	-1.5690146	0.09894795	-15.856968	1.2582E-56	3.7723E-55
ETNK2	ENSG00000143845	protein_coding	2166.40134	-1.730725	0.14561369	-11.885729	1.404E-32	1.5717E-31
GDF7	ENSG00000143869	protein_coding	544.400929	-1.5002116	0.14418854	-10.404513	2.3646E-25	1.8476E-24
ATP6V1C2	ENSG00000143882	protein_coding	453.511202	-2.1606503	0.2282824	-9.4648132	2.9409E-21	1.8402E-20
GALM	ENSG00000143891	protein_coding	3444.44735	-1.1935954	0.0828326	-14.40973	4.4946E-47	9.4167E-46
ABCG8	ENSG00000143921	protein_coding	12.3746145	-2.0780993	0.31637814	-6.5684036	5.0858E-11	1.6558E-10
SFXN5	ENSG00000144040	protein_coding	1108.79481	-1.263536	0.08725555	-14.480867	1.6007E-47	3.4144E-46
CNGA3	ENSG00000144191	protein_coding	4.07426094	-1.9441885	0.34746361	-5.595373	2.2015E-08	5.8086E-08
FAHD2B	ENSG00000144199	protein_coding	227.702608	-1.0545486	0.13362067	-7.8921067	2.9713E-15	1.2966E-14

NXPH2	ENSG00000144227	protein_coding	33.1198811	-3.5297633	0.63551532	-5.554175	2.7893E-08	7.2968E-08
GRIP2	ENSG00000144596	protein_coding	154.083943	-1.0213627	0.16044939	-6.3656374	1.9448E-10	6.0553E-10
GADL1	ENSG00000144644	protein_coding	32.0460705	-4.4112407	0.25402298	-17.365518	1.5053E-67	6.5972E-66
POMGNT2	ENSG00000144647	protein_coding	1002.07877	-1.1306502	0.06730978	-16.79771	2.5365E-63	9.5857E-62
ACKR2	ENSG00000144648	protein_coding	86.5838845	-2.2680821	0.15608682	-14.530901	7.7199E-48	1.666E-46
GASK1A	ENSG00000144649	protein_coding	67.5456566	-1.490135	0.14570151	-10.227314	1.4961E-24	1.117E-23
CTDSPL	ENSG00000144677	protein_coding	4156.19372	-1.2040068	0.08395952	-14.340325	1.2248E-46	2.5234E-45
CAND2	ENSG00000144712	protein_coding	214.470374	-1.8266474	0.11865472	-15.394645	1.778E-53	4.8113E-52
TAGLN3	ENSG00000144834	protein_coding	53.0780638	-3.1765165	0.30931778	-10.269428	9.6777E-25	7.2978E-24
IGSF11	ENSG00000144847	protein_coding	108.873358	-2.9059508	0.23907396	-12.155029	5.3943E-34	6.4767E-33
ALDH1L1	ENSG00000144908	protein_coding	6632.07847	-1.1991411	0.18446777	-6.5005455	8.0029E-11	2.5687E-10
SPATA16	ENSG00000144962	protein_coding	1.38098615	-3.5367312	0.30253925	-11.690156	1.4312E-31	1.5315E-30
AMT	ENSG00000145020	protein_coding	1141.84882	-1.105466	0.10335065	-10.696266	1.0596E-26	8.9078E-26
NICN1	ENSG00000145029	protein_coding	972.241798	-1.0833387	0.06342169	-17.081517	2.0375E-65	8.2803E-64
EAF2	ENSG00000145088	protein_coding	294.375069	-1.1082364	0.11885463	-9.3243011	1.1172E-20	6.7688E-20
ILDR1	ENSG00000145103	protein_coding	624.639448	-2.0018381	0.14964784	-13.376993	8.2415E-41	1.3261E-39
SLIT2	ENSG00000145147	protein_coding	1289.99727	-1.1515158	0.2163636	-5.3221325	1.0256E-07	2.5613E-07
EPHA5	ENSG00000145242	protein_coding	5.42103603	-2.0942128	0.39774643	-5.2651957	1.4004E-07	3.4551E-07
CORIN	ENSG00000145244	protein_coding	92.7836899	-1.5293538	0.12629622	-12.10926	9.4342E-34	1.1189E-32
SCD5	ENSG00000145284	protein_coding	2889.59364	-1.7436174	0.19308192	-9.0304542	1.7096E-19	9.6969E-19
ANK2	ENSG00000145362	protein_coding	5885.70057	-2.1479921	0.17833555	-12.044666	2.0691E-33	2.4141E-32
FABP2	ENSG00000145384	protein_coding	5.19213633	-1.9013715	0.26203964	-7.256045	3.9857E-13	1.5049E-12
USP53	ENSG00000145390	protein_coding	2376.66675	-1.385822	0.09139753	-15.162575	6.2567E-52	1.6077E-50
GLRA3	ENSG00000145451	protein_coding	6.08305157	-1.8509016	0.30759295	-6.0173734	1.7727E-09	5.1295E-09
CDH18	ENSG00000145526	protein_coding	12.0505489	-2.1204383	0.43803932	-4.8407488	1.2935E-06	2.9279E-06

ADAMTS16	ENSG00000145536	protein_coding	293.444945	-2.6780165	0.19462221	-13.760077	4.4306E-43	7.8844E-42
MYO10	ENSG00000145555	protein_coding	4502.56433	-1.1015648	0.11733986	-9.3878139	6.1258E-21	3.7567E-20
UGT3A1	ENSG00000145626	protein_coding	2847.06891	-1.3364779	0.27982145	-4.7761811	1.7866E-06	3.9907E-06
BHMT	ENSG00000145692	protein_coding	16819.9631	-1.3168494	0.18754583	-7.0214805	2.1953E-12	7.8836E-12
IQGAP2	ENSG00000145703	protein_coding	3433.90142	-1.1025935	0.12253009	-8.9985527	2.2871E-19	1.2879E-18
CRHBP	ENSG00000145708	protein_coding	396.391297	-4.0887634	0.20634337	-19.815337	2.1953E-87	2.0007E-85
LIX1	ENSG00000145721	protein_coding	475.199106	-1.7351378	0.30550279	-5.6796138	1.35E-08	3.629E-08
TSLP	ENSG00000145777	protein_coding	41.0687863	-1.1233186	0.18008201	-6.2378169	4.4372E-10	1.3488E-09
ADAMTS19	ENSG00000145808	protein_coding	15.1466897	-4.3253886	0.39850858	-10.853941	1.9101E-27	1.6656E-26
SLC25A48	ENSG00000145832	protein_coding	266.400977	-1.1066212	0.2128509	-5.199044	2.0032E-07	4.881E-07
GABRB2	ENSG00000145864	protein_coding	81.8516894	-1.8837329	0.22969296	-8.2010912	2.3821E-16	1.1145E-15
FAM217A	ENSG00000145975	protein_coding	9.26144091	-2.3750851	0.15860375	-14.974962	1.0703E-50	2.6097E-49
GFRA3	ENSG00000146013	protein_coding	14.0088553	-1.6265203	0.25630666	-6.3459933	2.2099E-10	6.8487E-10
KLHL3	ENSG00000146021	protein_coding	755.139746	-1.632161	0.18974024	-8.6020815	7.8283E-18	4.0217E-17
DCDC2	ENSG00000146038	protein_coding	2718.89806	-1.6610455	0.16354595	-10.156445	3.1018E-24	2.2725E-23
KAAG1	ENSG00000146049	protein_coding	33.3897646	-1.0872291	0.15466364	-7.0296362	2.0707E-12	7.4515E-12
MMUT	ENSG00000146085	protein_coding	3257.78896	-1.0813393	0.07851448	-13.772482	3.7317E-43	6.6588E-42
LGSN	ENSG00000146166	protein_coding	45.4400955	-2.0824855	0.21869277	-9.5224253	1.6918E-21	1.0725E-20
SCUBE3	ENSG00000146197	protein_coding	387.016918	-1.1374745	0.16971951	-6.7020848	2.0547E-11	6.8841E-11
CYP39A1	ENSG00000146233	protein_coding	281.941071	-1.671815	0.16786928	-9.9590291	2.303E-23	1.6086E-22
PRSS35	ENSG00000146250	protein_coding	193.921232	-2.1799492	0.19066234	-11.43356	2.8421E-30	2.8515E-29
FAXC	ENSG00000146267	protein_coding	103.073633	-1.625285	0.28429356	-5.7169251	1.0847E-08	2.9361E-08
RSPO3	ENSG00000146374	protein_coding	198.518608	-1.2689157	0.19696238	-6.4424267	1.1758E-10	3.7228E-10
SLC2A12	ENSG00000146411	protein_coding	193.326107	-3.3108551	0.22181312	-14.926327	2.2217E-50	5.3518E-49
SSC4D	ENSG00000146700	protein_coding	153.126869	-2.2403638	0.14581062	-15.364888	2.8153E-53	7.5692E-52

ASB15	ENSG00000146809	protein_coding	66.3201599	-5.2459359	0.33360293	-15.725089	1.0181E-55	2.9669E-54
EPHA1	ENSG00000146904	protein_coding	318.867043	-1.5422556	0.11825617	-13.04165	7.0906E-39	1.0539E-37
ASB10	ENSG00000146926	protein_coding	0.68787452	-3.3711711	0.33614985	-10.028775	1.1392E-23	8.0928E-23
SYTL5	ENSG00000147041	protein_coding	102.6645	-1.5556639	0.24470169	-6.3573893	2.0521E-10	6.378E-10
AKAP4	ENSG00000147081	protein_coding	1.82174506	-2.3404062	0.19436203	-12.041478	2.1507E-33	2.5058E-32
ZNF157	ENSG00000147117	protein_coding	14.9279763	-1.2688684	0.12770368	-9.9360359	2.9014E-23	2.0168E-22
NXF3	ENSG00000147206	protein_coding	15.0792592	-1.1932582	0.17068948	-6.9908124	2.733E-12	9.7481E-12
RIPPLY1	ENSG00000147223	protein_coding	26.4443788	-2.8623878	0.1860852	-15.382136	2.1571E-53	5.8168E-52
GPC3	ENSG00000147257	protein_coding	1431.72317	-3.6417277	0.21733684	-16.756145	5.1068E-63	1.9002E-61
MFHAS1	ENSG00000147324	protein_coding	1830.43284	-1.1307454	0.09569786	-11.815786	3.2351E-32	3.5692E-31
SNTG1	ENSG00000147481	protein_coding	11.5065996	-1.9487834	0.41924783	-4.648285	3.3471E-06	7.2942E-06
CRH	ENSG00000147571	protein_coding	3.14365218	-1.2823656	0.47148245	-2.7198586	0.00653098	0.01002904
ADHFE1	ENSG00000147576	protein_coding	502.789701	-1.1784428	0.11013392	-10.70009	1.0168E-26	8.5588E-26
PMP2	ENSG00000147588	protein_coding	8.57023494	-2.4433579	0.44592464	-5.4793068	4.27E-08	1.0996E-07
SLC26A7	ENSG00000147606	protein_coding	2146.48467	-1.8347962	0.37991055	-4.8295478	1.3684E-06	3.0905E-06
PSKH2	ENSG00000147613	protein_coding	2.89224346	-4.1062492	0.48280998	-8.5048971	1.8176E-17	9.1379E-17
ATP6V0D2	ENSG00000147614	protein_coding	3756.91729	-1.1971317	0.43113784	-2.7766797	0.00549173	0.00850839
DPYS	ENSG00000147647	protein_coding	3823.56489	-1.4809334	0.1947214	-7.605396	2.8403E-14	1.1603E-13
MAL2	ENSG00000147676	protein_coding	4637.10838	-2.7246176	0.19196433	-14.193353	1.0073E-45	2.0023E-44
SLC39A4	ENSG00000147804	protein_coding	929.699847	-1.544389	0.14517326	-10.638247	1.9782E-26	1.6401E-25
AK3	ENSG00000147853	protein_coding	4746.42384	-1.4684541	0.07080736	-20.738722	1.5499E-95	1.9519E-93
CER1	ENSG00000147869	protein_coding	1.56520638	-2.7660863	0.30762282	-8.9918112	2.4319E-19	1.367E-18
NTRK2	ENSG00000148053	protein_coding	2933.44687	-2.0850844	0.1900381	-10.971928	5.2148E-28	4.6836E-27
IDNK	ENSG00000148057	protein_coding	526.501837	-1.1949093	0.09547233	-12.515766	6.1215E-36	8.0521E-35
SHC3	ENSG00000148082	protein_coding	145.944186	-1.5562872	0.146826	-10.599534	2.9947E-26	2.4536E-25

AUH	ENSG00000148090	protein_coding	1640.95155	-1.1230409	0.09958272	-11.277468	1.6956E-29	1.6461E-28
PLPPR1	ENSG00000148123	protein_coding	297.401216	-4.20854	0.3444633	-12.217673	2.5014E-34	3.048E-33
CRB2	ENSG00000148204	protein_coding	309.499052	-2.9029746	0.23703561	-12.246998	1.7433E-34	2.1396E-33
ALAD	ENSG00000148218	protein_coding	3749.4771	-1.2968084	0.06699993	-19.35537	1.8367E-83	1.4417E-81
SLC25A25	ENSG00000148339	protein_coding	1265.0576	-1.8902299	0.1097235	-17.227211	1.6596E-66	7.0054E-65
LCN2	ENSG00000148346	protein_coding	320.675058	-1.5542867	0.35724162	-4.3507997	1.3564E-05	2.7874E-05
PROSER2	ENSG00000148426	protein_coding	657.076629	-1.128498	0.12490055	-9.0351726	1.6374E-19	9.306E-19
FAM171A1	ENSG00000148468	protein_coding	2436.80936	-2.1814236	0.09855633	-22.133775	1.495E-108	3.019E-106
PLEKHS1	ENSG00000148735	protein_coding	74.6652338	-1.0515039	0.30281745	-3.4724018	0.00051582	0.00089867
CYP17A1	ENSG00000148795	protein_coding	990.507574	-1.9631541	0.34441424	-5.6999796	1.1982E-08	3.2335E-08
NKX6-2	ENSG00000148826	protein_coding	1.20960039	-3.0489347	0.24289329	-12.552568	3.8482E-36	5.1073E-35
CNNM2	ENSG00000148842	protein_coding	1412.88853	-1.0560859	0.07221576	-14.624036	1.9735E-48	4.398E-47
SLC5A12	ENSG00000148942	protein_coding	7210.06765	-1.5819406	0.25945134	-6.0972534	1.0791E-09	3.1782E-09
GLYAT	ENSG00000149124	protein_coding	4393.8156	-1.5300453	0.19761533	-7.7425436	9.7447E-15	4.1126E-14
SLC43A1	ENSG00000149150	protein_coding	505.559595	-1.295697	0.14036129	-9.2311566	2.6773E-20	1.586E-19
CAPN5	ENSG00000149260	protein_coding	1949.94045	-1.0828252	0.09422464	-11.491954	1.448E-30	1.4751E-29
HTR3B	ENSG00000149305	protein_coding	6.20237205	-2.6909351	0.51909775	-5.1838697	2.1733E-07	5.2765E-07
ST14	ENSG00000149418	protein_coding	4575.40375	-1.2243685	0.17765498	-6.8918334	5.5078E-12	1.9213E-11
SLC22A8	ENSG00000149452	protein_coding	2524.68245	-6.1500703	0.35274353	-17.434963	4.4785E-68	2.0029E-66
TKFC	ENSG00000149476	protein_coding	2872.26134	-1.2491826	0.07321572	-17.061671	2.8625E-65	1.1592E-63
SCN2B	ENSG00000149575	protein_coding	53.3200017	-2.2758101	0.29355712	-7.752529	9.008E-15	3.8094E-14
TMEM25	ENSG00000149582	protein_coding	1198.38277	-1.2337031	0.10740327	-11.486644	1.5398E-30	1.5661E-29
DUSP15	ENSG00000149599	protein_coding	526.429286	-1.1867024	0.25338164	-4.6834584	2.8207E-06	6.189E-06
CNTN5	ENSG00000149972	protein_coding	26.6335943	-2.5237542	0.28693527	-8.7955524	1.4235E-18	7.6615E-18
MPP7	ENSG00000150054	protein_coding	897.756857	-2.7834337	0.15998034	-17.398599	8.4546E-68	3.7374E-66

FXYD4	ENSG00000150201	protein_coding	1746.79366	-6.1644654	0.47249132	-13.046727	6.6336E-39	9.8795E-38
PCDH15	ENSG00000150275	protein_coding	18.6337558	-3.9615092	0.40360229	-9.8153783	9.6675E-23	6.5393E-22
CTF1	ENSG00000150281	protein_coding	374.200623	-1.1767569	0.12475898	-9.4322425	4.0141E-21	2.4917E-20
KLHL1	ENSG00000150361	protein_coding	2.97719307	-3.170298	0.42677793	-7.4284488	1.0988E-13	4.3232E-13
FAM124A	ENSG00000150510	protein_coding	465.690432	-1.1407547	0.14071643	-8.1067628	5.1986E-16	2.3817E-15
LYPD6B	ENSG00000150556	protein_coding	190.453184	-2.7243119	0.34926718	-7.80008	6.1868E-15	2.6444E-14
CNDP1	ENSG00000150656	protein_coding	113.207081	-1.2318853	0.19650908	-6.2688467	3.6373E-10	1.1117E-09
DLG2	ENSG00000150672	protein_coding	253.23181	-1.1817672	0.1689994	-6.9927302	2.6959E-12	9.621E-12
PPP1R1C	ENSG00000150722	protein_coding	31.2222284	-1.643252	0.26062716	-6.3049915	2.8821E-10	8.8676E-10
C11orf53	ENSG00000150750	protein_coding	12.8116975	-1.2642691	0.57731286	-2.1899201	0.02853003	0.03999282
FREM2	ENSG00000150893	protein_coding	1874.35169	-1.6944039	0.17919549	-9.4556168	3.2112E-21	2.0044E-20
THRB	ENSG00000151090	protein_coding	1111.10791	-1.8944085	0.13673795	-13.8543	1.1982E-43	2.1883E-42
TMEM86A	ENSG00000151117	protein_coding	965.82389	-1.4099183	0.10110269	-13.945409	3.3552E-44	6.2523E-43
BTBD11	ENSG00000151136	protein_coding	453.12038	-1.4540502	0.21879516	-6.6457145	3.0175E-11	1E-10
ANK3	ENSG00000151150	protein_coding	5078.11782	-1.3396036	0.1363339	-9.825902	8.7091E-23	5.9041E-22
ALLC	ENSG00000151360	protein_coding	6.18150562	-1.3616337	0.2828927	-4.8132514	1.4849E-06	3.3437E-06
ATP6V1G3	ENSG00000151418	protein_coding	308.554904	-1.7210764	0.73342972	-2.3466139	0.01894487	0.0272541
PTPRO	ENSG00000151490	protein_coding	1144.97842	-3.2056759	0.16023242	-20.006413	4.8427E-89	4.7032E-87
ACAD8	ENSG00000151498	protein_coding	1637.66739	-1.1006642	0.06249375	-17.612387	1.9792E-69	9.2864E-68
NR3C2	ENSG00000151623	protein_coding	1444.71691	-1.9982644	0.14476287	-13.803708	2.4207E-43	4.3559E-42
AKR1C2	ENSG00000151632	protein_coding	575.552144	-1.1543974	0.20014435	-5.7678243	8.0301E-09	2.1969E-08
ITIH2	ENSG00000151655	protein_coding	51.7213295	-1.2635356	0.19769812	-6.3912374	1.6455E-10	5.1538E-10
ASAP2	ENSG00000151693	protein_coding	1876.837	-1.1771484	0.10208066	-11.531552	9.148E-31	9.3689E-30
KCNJ1	ENSG00000151704	protein_coding	3601.47828	-6.2763965	0.30641619	-20.48324	3.0376E-93	3.5342E-91
TMEM45B	ENSG00000151715	protein_coding	567.307883	-3.9092541	0.18469654	-21.165822	1.973E-99	2.8445E-97

SLC25A4	ENSG00000151729	protein_coding	4054.85525	-1.1188385	0.10544766	-10.610368	2.667E-26	2.1918E-25
ZNF385D	ENSG00000151789	protein_coding	260.113977	-1.3010028	0.15414511	-8.4401174	3.1702E-17	1.5697E-16
GABRA2	ENSG00000151834	protein_coding	233.589808	-4.8179792	0.44830155	-10.747184	6.1099E-27	5.2021E-26
TLCD4	ENSG00000152078	protein_coding	908.594979	-1.132645	0.12290398	-9.21569	3.0928E-20	1.824E-19
TMEM178A	ENSG00000152154	protein_coding	335.90666	-3.2234834	0.16433983	-19.614742	1.1572E-85	9.9156E-84
ATP5F1A	ENSG00000152234	protein_coding	30139.2157	-1.2323378	0.09315232	-13.229277	5.9457E-40	9.2298E-39
SH2D6	ENSG00000152292	protein_coding	20.9518983	-1.5015772	0.20222896	-7.4251343	1.1267E-13	4.4298E-13
KCNK13	ENSG00000152315	protein_coding	193.737745	-2.6379262	0.12925575	-20.408579	1.4031E-92	1.5881E-90
JMY	ENSG00000152409	protein_coding	1315.46905	-1.191317	0.09335036	-12.761783	2.68E-37	3.7524E-36
HOMER1	ENSG00000152413	protein_coding	504.688476	-2.198657	0.12827298	-17.140453	7.4071E-66	3.0507E-64
OLAH	ENSG00000152463	protein_coding	13.0429012	-1.2345184	0.14986371	-8.2376071	1.7569E-16	8.296E-16
IGSF10	ENSG00000152580	protein_coding	57.2211039	-2.7429601	0.18410663	-14.898758	3.3576E-50	8.0165E-49
CAPSL	ENSG00000152611	protein_coding	9.47213992	-2.8471708	0.24369859	-11.683165	1.554E-31	1.659E-30
GPD1L	ENSG00000152642	protein_coding	2313.86204	-1.4788249	0.1389419	-10.643477	1.8702E-26	1.5542E-25
CCNO	ENSG00000152669	protein_coding	71.5517435	-1.1597101	0.26673158	-4.3478546	1.3748E-05	2.8232E-05
SLC16A12	ENSG00000152779	protein_coding	6731.58147	-1.1643223	0.18989406	-6.1314307	8.7092E-10	2.5851E-09
PANK1	ENSG00000152782	protein_coding	1575.09796	-1.4779606	0.13438221	-10.998186	3.899E-28	3.526E-27
BMP3	ENSG00000152785	protein_coding	22.8025632	-1.9561304	0.21181679	-9.2350112	2.5826E-20	1.5312E-19
GRM1	ENSG00000152822	protein_coding	88.1293725	-3.36794	0.25783746	-13.06226	5.4097E-39	8.1042E-38
CNTNAP4	ENSG00000152910	protein_coding	3.36265927	-2.1496592	0.34678539	-6.1988172	5.6889E-10	1.7131E-09
LMNTD1	ENSG00000152936	protein_coding	1.43243826	-1.5622214	0.34865381	-4.4807236	7.439E-06	1.5678E-05
MARVELD2	ENSG00000152939	protein_coding	949.202164	-2.3792019	0.14260783	-16.68353	1.7271E-62	6.3134E-61
LGI2	ENSG00000153012	protein_coding	492.521567	-3.2773683	0.15258513	-21.478949	2.45E-102	4.069E-100
BMP6	ENSG00000153162	protein_coding	724.682199	-2.1244151	0.13651641	-15.56161	1.3272E-54	3.7302E-53
RGPD3	ENSG00000153165	protein_coding	13.4863681	-1.5796751	0.17643795	-8.953148	3.4549E-19	1.9263E-18

NR4A2	ENSG00000153234	protein_coding	1429.41615	-1.1810141	0.17520806	-6.740638	1.5769E-11	5.3259E-11
PLA2R1	ENSG00000153246	protein_coding	1379.16391	-3.1192461	0.13487104	-23.12762	2.442E-118	6.398E-116
SCN3A	ENSG00000153253	protein_coding	99.3540759	-1.5282992	0.27721709	-5.5130051	3.5276E-08	9.1464E-08
ADGRF1	ENSG00000153292	protein_coding	1325.03369	-3.9888372	0.37381872	-10.670512	1.3985E-26	1.1697E-25
FRMD1	ENSG00000153303	protein_coding	152.162744	-2.0192309	0.27752343	-7.2758934	3.4414E-13	1.3058E-12
PLEKHG4B	ENSG00000153404	protein_coding	185.772807	-2.1304491	0.2270453	-9.3833659	6.39E-21	3.9155E-20
C16orf89	ENSG00000153446	protein_coding	490.968108	-2.8477377	0.26870087	-10.598171	3.0387E-26	2.4879E-25
ADPRHL1	ENSG00000153531	protein_coding	50.1246733	-1.0762876	0.13068273	-8.2358821	1.7824E-16	8.4078E-16
PTPRD	ENSG00000153707	protein_coding	1748.20006	-1.1724213	0.20125684	-5.8254978	5.6943E-09	1.5777E-08
SPHKAP	ENSG00000153820	protein_coding	5.1933654	-1.8188738	0.38808021	-4.6868501	2.7744E-06	6.0911E-06
KCNJ16	ENSG00000153822	protein_coding	12771.7879	-1.5804177	0.1301113	-12.14666	5.9757E-34	7.1579E-33
MSI2	ENSG00000153944	protein_coding	6006.24654	-1.02261	0.09855558	-10.375972	3.1895E-25	2.4739E-24
AK5	ENSG00000154027	protein_coding	72.4824933	-1.2601535	0.17421313	-7.2334014	4.7104E-13	1.7711E-12
PRKCA	ENSG00000154229	protein_coding	2721.38628	-1.2217445	0.10358973	-11.79407	4.188E-32	4.5918E-31
GAL3ST2	ENSG00000154252	protein_coding	6.64938607	-1.3105987	0.20812234	-6.2972517	3.0297E-10	9.3098E-10
UCHL1	ENSG00000154277	protein_coding	1601.2348	-1.8959188	0.24579974	-7.7132661	1.2264E-14	5.135E-14
FAM167A	ENSG00000154319	protein_coding	400.113315	-3.5131304	0.19108006	-18.385646	1.7119E-75	1.0139E-73
PGM5	ENSG00000154330	protein_coding	1473.62413	-1.2387498	0.12745461	-9.7191443	2.4987E-22	1.6522E-21
TMSB4Y	ENSG00000154620	protein_coding	70.6776046	-1.5952198	0.29366037	-5.432193	5.5666E-08	1.4224E-07
CHODL	ENSG00000154645	protein_coding	115.455355	-1.6749903	0.23892272	-7.0105942	2.3731E-12	8.5001E-12
NCAM2	ENSG00000154654	protein_coding	39.4924507	-1.3917851	0.22990445	-6.0537547	1.4151E-09	4.1268E-09
WNT7A	ENSG00000154764	protein_coding	24.30334	-2.9955273	0.27943986	-10.719757	8.2219E-27	6.9513E-26
OXNAD1	ENSG00000154814	protein_coding	623.107783	-1.1143274	0.10373533	-10.742023	6.4612E-27	5.4931E-26
CA10	ENSG00000154975	protein_coding	144.040088	-4.8332042	0.42311601	-11.422882	3.214E-30	3.214E-29
PROM2	ENSG00000155066	protein_coding	1991.7022	-3.5000359	0.28780769	-12.161023	5.0127E-34	6.0264E-33

PPM1J	ENSG00000155367	protein_coding	59.0442648	-1.1589918	0.12930587	-8.9631797	3.1545E-19	1.7615E-18
SLC7A7	ENSG00000155465	protein_coding	5326.49645	-1.1862743	0.18069899	-6.5649196	5.2061E-11	1.6929E-10
XAGE2	ENSG00000155622	protein_coding	1.87658748	-3.1410227	0.40334412	-7.7874513	6.8374E-15	2.9149E-14
DEPTOR	ENSG00000155792	protein_coding	1999.14561	-1.5696128	0.08678949	-18.085287	4.162E-73	2.2499E-71
FMN2	ENSG00000155816	protein_coding	68.4661881	-3.7632725	0.28738555	-13.094856	3.5234E-39	5.3323E-38
SLC24A2	ENSG00000155886	protein_coding	41.4650194	-1.653565	0.39377402	-4.199274	2.6777E-05	5.3486E-05
RAET1L	ENSG00000155918	protein_coding	5.46174532	-2.2901599	0.26236399	-8.7289415	2.5707E-18	1.3608E-17
GRIP1	ENSG00000155974	protein_coding	236.953079	-1.2964591	0.13941512	-9.2992714	1.4141E-20	8.5273E-20
KIF5A	ENSG00000155980	protein_coding	51.1649541	-2.138896	0.19518943	-10.958053	6.0793E-28	5.443E-27
NAT2	ENSG00000156006	protein_coding	26.8082192	-1.7130476	0.2217295	-7.7258445	1.1111E-14	4.6704E-14
WIF1	ENSG00000156076	protein_coding	8.47248418	-1.9985818	0.28209777	-7.084713	1.3933E-12	5.0792E-12
ADK	ENSG00000156110	protein_coding	1129.62267	-1.157158	0.0675301	-17.135439	8.0741E-66	3.3225E-64
CLDN8	ENSG00000156284	protein_coding	1561.75058	-3.7208083	0.59562808	-6.2468651	4.1877E-10	1.2748E-09
ANKRD9	ENSG00000156381	protein_coding	995.338655	-1.1783628	0.12200464	-9.6583443	4.5313E-22	2.9611E-21
SFXN2	ENSG00000156398	protein_coding	1273.74676	-2.3847978	0.11036299	-21.608674	1.489E-103	2.575E-101
FUT6	ENSG00000156413	protein_coding	1355.50534	-1.3181618	0.21181163	-6.223274	4.8689E-10	1.475E-09
TDRD9	ENSG00000156414	protein_coding	232.697316	-1.1035427	0.24037223	-4.5909744	4.4118E-06	9.5074E-06
PCDH1	ENSG00000156453	protein_coding	3757.57773	-1.0050664	0.10424533	-9.6413563	5.3477E-22	3.4811E-21
PPP2R2B	ENSG00000156475	protein_coding	215.061616	-2.2931329	0.12104166	-18.944989	4.8569E-80	3.3992E-78
KCNS2	ENSG00000156486	protein_coding	17.817474	-1.3586393	0.22912503	-5.9296854	3.0352E-09	8.613E-09
ZIC3	ENSG00000156925	protein_coding	1.12595917	-2.231853	0.3701604	-6.0294211	1.6455E-09	4.7729E-09
SYN2	ENSG00000157152	protein_coding	68.020038	-2.3214832	0.18900034	-12.282958	1.1183E-34	1.3874E-33
DHRS4	ENSG00000157326	protein_coding	1057.40159	-1.0529263	0.09806174	-10.73738	6.7945E-27	5.7722E-26
CACNA2D3	ENSG00000157445	protein_coding	163.632884	-2.1037514	0.12194663	-17.25141	1.092E-66	4.6437E-65
FAM81A	ENSG00000157470	protein_coding	313.399769	-2.5561359	0.12916734	-19.789336	3.6786E-87	3.3195E-85

KCNJ15	ENSG00000157551	protein_coding	9532.70091	-1.3760221	0.16125987	-8.5329478	1.4267E-17	7.2208E-17
TMEM164	ENSG00000157600	protein_coding	1855.25157	-1.5029859	0.06781909	-22.161694	8.047E-109	1.662E-106
SVOPL	ENSG00000157703	protein_coding	63.2273723	-2.0916227	0.32505982	-6.434578	1.2382E-10	3.9141E-10
DRC1	ENSG00000157856	protein_coding	12.1064217	-1.1594248	0.20912157	-5.5442621	2.952E-08	7.7053E-08
PAFAH2	ENSG00000158006	protein_coding	1660.33872	-1.0625172	0.0563099	-18.869102	2.0475E-79	1.3884E-77
EXTL1	ENSG00000158008	protein_coding	22.3749776	-1.3525612	0.15770545	-8.5765029	9.7801E-18	4.9948E-17
SLC30A2	ENSG00000158014	protein_coding	360.509171	-3.3956026	0.28302289	-11.997625	3.6564E-33	4.2209E-32
NLRP14	ENSG00000158077	protein_coding	25.6793264	-1.0276556	0.14543665	-7.0660017	1.5946E-12	5.7914E-12
HPD	ENSG00000158104	protein_coding	2366.13541	-4.8800358	0.28164653	-17.326809	2.9526E-67	1.2842E-65
LRRC43	ENSG00000158113	protein_coding	90.6236938	-1.4853824	0.18193525	-8.1643464	3.2318E-16	1.4996E-15
ESYT3	ENSG00000158220	protein_coding	102.026956	-1.0248402	0.18178425	-5.6376732	1.7236E-08	4.5927E-08
TENT5B	ENSG00000158246	protein_coding	216.87624	-2.5751627	0.1804326	-14.272159	3.2632E-46	6.6014E-45
CLSTN2	ENSG00000158258	protein_coding	961.034125	-2.7142844	0.20400634	-13.304902	2.1678E-40	3.4397E-39
SLC13A3	ENSG00000158296	protein_coding	4136.43168	-4.7025669	0.31407222	-14.972884	1.1043E-50	2.6912E-49
TSPAN33	ENSG00000158457	protein_coding	5215.69617	-1.5640106	0.12291645	-12.724176	4.3403E-37	6.0237E-36
PFKFB1	ENSG00000158571	protein_coding	17.0487997	-1.3571473	0.11252967	-12.06035	1.7105E-33	2.0034E-32
ITLN2	ENSG00000158764	protein_coding	2.77022004	-2.9177127	0.28718963	-10.159533	3.0051E-24	2.2045E-23
NPM2	ENSG00000158806	protein_coding	112.670407	-1.0196466	0.17438269	-5.847178	4.9998E-09	1.3937E-08
VWA5B1	ENSG00000158816	protein_coding	85.6686445	-2.0998915	0.35358148	-5.9389182	2.8691E-09	8.1569E-09
SLC5A11	ENSG00000158865	protein_coding	73.0597085	-3.0977839	0.21287604	-14.552055	5.6675E-48	1.2336E-46
WNT9B	ENSG00000158955	protein_coding	95.5260663	-4.1171559	0.19671504	-20.929543	2.8825E-97	3.9326E-95
LAD1	ENSG00000159166	protein_coding	1210.39756	-2.6727136	0.22575252	-11.839131	2.4498E-32	2.7165E-31
TNNI1	ENSG00000159173	protein_coding	144.184384	-4.0527498	0.24004247	-16.88347	5.954E-64	2.2917E-62
GJD2	ENSG00000159248	protein_coding	9.36288284	-1.963973	0.51227	-3.833863	0.00012615	0.00023498
CLDN14	ENSG00000159261	protein_coding	137.03435	-2.950988	0.17382032	-16.977233	1.2106E-64	4.7965E-63

SIM2	ENSG00000159263	protein_coding	371.190018	-4.5057163	0.14657803	-30.739369	1.696E-207	5.554E-204
SCUBE1	ENSG00000159307	protein_coding	305.706519	-1.295436	0.18679833	-6.9349441	4.0638E-12	1.4313E-11
M1AP	ENSG00000159374	protein_coding	59.7047336	-1.7403157	0.12621021	-13.789025	2.9674E-43	5.3179E-42
BTG2	ENSG00000159388	protein_coding	9883.15934	-2.1160182	0.10805135	-19.583451	2.1402E-85	1.8068E-83
CELF3	ENSG00000159409	protein_coding	5.2256076	-1.8468558	0.20214431	-9.1363232	6.4612E-20	3.7568E-19
ALDH4A1	ENSG00000159423	protein_coding	5053.20536	-2.0822858	0.1652977	-12.597185	2.1881E-36	2.939E-35
AMFR	ENSG00000159461	protein_coding	9207.78077	-1.3978354	0.08260443	-16.922039	3.0953E-64	1.2076E-62
TGM7	ENSG00000159495	protein_coding	3.24210789	-1.4686589	0.48081388	-3.0545269	0.00225416	0.00365431
AGRP	ENSG00000159723	protein_coding	8.87339511	-1.8466071	0.16395978	-11.262561	2.0083E-29	1.9415E-28
FNDC5	ENSG00000160097	protein_coding	69.1059655	-1.2806272	0.19486418	-6.5718966	4.9678E-11	1.619E-10
CPAMD8	ENSG00000160111	protein_coding	839.944912	-2.7933157	0.13779276	-20.271862	2.2787E-91	2.3958E-89
CBS	ENSG00000160200	protein_coding	11.4873711	-1.1494016	0.23577341	-4.8750264	1.0879E-06	2.4808E-06
CRYAA	ENSG00000160202	protein_coding	3.52717431	-4.5851156	0.48378914	-9.4775082	2.6043E-21	1.6343E-20
PDXK	ENSG00000160209	protein_coding	8095.03607	-1.0650718	0.08610131	-12.369983	3.799E-35	4.8055E-34
FTCD	ENSG00000160282	protein_coding	1987.40066	-1.2752541	0.26500584	-4.8121735	1.493E-06	3.361E-06
TLCD1	ENSG00000160606	protein_coding	242.123048	-1.2511093	0.14146507	-8.8439449	9.2397E-19	5.0273E-18
PTH1R	ENSG00000160801	protein_coding	2598.89384	-3.265412	0.18147289	-17.993939	2.1735E-72	1.14E-70
MYL3	ENSG00000160808	protein_coding	355.809424	-1.2925835	0.20460686	-6.3174008	2.66E-10	8.2024E-10
AZGP1	ENSG00000160862	protein_coding	2645.31444	-1.7842572	0.2300335	-7.75651	8.7298E-15	3.6945E-14
CYP3A4	ENSG00000160868	protein_coding	44.6818469	-2.0913785	0.20657898	-10.123869	4.3296E-24	3.1469E-23
CYP3A7	ENSG00000160870	protein_coding	163.802792	-1.0556246	0.18281768	-5.774193	7.7323E-09	2.1175E-08
LY6K	ENSG00000160886	protein_coding	45.2673233	-1.9642155	0.21743575	-9.0335443	1.662E-19	9.4385E-19
PTGER1	ENSG00000160951	protein_coding	109.29899	-4.8529773	0.30362843	-15.983277	1.6712E-57	5.1975E-56
COL26A1	ENSG00000160963	protein_coding	114.210717	-4.6516148	0.28204593	-16.492402	4.1607E-61	1.4604E-59
NAPEPLD	ENSG00000161048	protein_coding	1840.27879	-1.1909657	0.07257075	-16.411099	1.593E-60	5.4946E-59

BDH1	ENSG00000161267	protein_coding	703.180082	-1.798194	0.19746002	-9.1066234	8.4986E-20	4.9047E-19
NPHS1	ENSG00000161270	protein_coding	604.597184	-5.9631731	0.29984162	-19.887743	5.1962E-88	4.8414E-86
FAM171A2	ENSG00000161682	protein_coding	108.415211	-1.0573692	0.14116453	-7.490332	6.87E-14	2.7418E-13
AQP5	ENSG00000161798	protein_coding	32.9615997	-2.5038009	0.34565084	-7.2437288	4.3651E-13	1.6438E-12
PRR35	ENSG00000161992	protein_coding	92.7091266	-6.9489294	0.43648628	-15.920155	4.5923E-57	1.4016E-55
SSTR5	ENSG00000162009	protein_coding	22.7073939	-3.7652308	0.31068664	-12.119062	8.3711E-34	9.9673E-33
HS3ST6	ENSG00000162040	protein_coding	5.817144	-3.9751246	0.37801191	-10.515871	7.3003E-26	5.8649E-25
TBC1D24	ENSG00000162065	protein_coding	1217.63208	-1.3583696	0.09657085	-14.066042	6.1412E-45	1.1754E-43
CYP4A22	ENSG00000162365	protein_coding	707.775482	-1.8774298	0.31059233	-6.0446755	1.4971E-09	4.3563E-09
ACOT11	ENSG00000162390	protein_coding	802.307167	-1.9838528	0.13871977	-14.301154	2.1521E-46	4.3905E-45
FAM151A	ENSG00000162391	protein_coding	1250.50969	-3.9802774	0.24971375	-15.93936	3.3779E-57	1.0399E-55
BSND	ENSG00000162399	protein_coding	644.556382	-3.2767894	0.58273198	-5.6231501	1.8751E-08	4.9809E-08
KLHL21	ENSG00000162413	protein_coding	3293.34273	-1.1740989	0.09000566	-13.044723	6.8104E-39	1.0136E-37
TMEM82	ENSG00000162460	protein_coding	305.668238	-1.1807169	0.25118436	-4.7005988	2.594E-06	5.7084E-06
AKR7A3	ENSG00000162482	protein_coding	1232.03599	-1.5520439	0.1805061	-8.5982905	8.0912E-18	4.1531E-17
LRRC38	ENSG00000162494	protein_coding	2.58289931	-1.5774028	0.37079396	-4.2541222	2.0987E-05	4.2352E-05
KIAA1522	ENSG00000162522	protein_coding	4268.83733	-1.4075188	0.08485268	-16.587794	8.54E-62	3.0513E-60
WNT4	ENSG00000162552	protein_coding	74.3344512	-1.8459908	0.22042706	-8.3746108	5.5407E-17	2.702E-16
DIRAS3	ENSG00000162595	protein_coding	185.901628	-1.837642	0.17246284	-10.655293	1.6473E-26	1.3717E-25
C1orf87	ENSG00000162598	protein_coding	5.68107586	-2.7702823	0.33239561	-8.3342928	7.7963E-17	3.7671E-16
LRRIQ3	ENSG00000162620	protein_coding	81.2629499	-1.2968791	0.11852676	-10.941657	7.2856E-28	6.5002E-27
B3GALT2	ENSG00000162630	protein_coding	54.6013687	-1.6128095	0.16320881	-9.8818774	4.9889E-23	3.4231E-22
NTNG1	ENSG00000162631	protein_coding	168.59463	-4.4796892	0.31469405	-14.235061	5.5514E-46	1.1113E-44
WDR63	ENSG00000162643	protein_coding	45.1536544	-1.3507157	0.14717962	-9.1773283	4.4192E-20	2.5892E-19
BRINP3	ENSG00000162670	protein_coding	31.9584671	-4.3210571	0.4639993	-9.312637	1.247E-20	7.5372E-20

KCNJ9	ENSG00000162728	protein_coding	10.0800553	-1.7443443	0.15197081	-11.478153	1.6987E-30	1.7239E-29
LRRC52	ENSG00000162763	protein_coding	11.5060758	-2.6613226	0.60773284	-4.3790995	1.1917E-05	2.4618E-05
ATF3	ENSG00000162772	protein_coding	5478.23204	-1.2223501	0.1790958	-6.825119	8.7852E-12	3.023E-11
AXDND1	ENSG00000162779	protein_coding	21.5912173	-2.1164582	0.16799843	-12.598083	2.1634E-36	2.9066E-35
TDRD5	ENSG00000162782	protein_coding	40.7252041	-2.8214447	0.28556405	-9.8802517	5.0705E-23	3.477E-22
SPATA17	ENSG00000162814	protein_coding	154.9337	-1.1767879	0.10904627	-10.791638	3.7701E-27	3.2455E-26
KIF26B	ENSG00000162849	protein_coding	553.35166	-1.2789424	0.16178285	-7.9053028	2.6728E-15	1.171E-14
PM20D1	ENSG00000162877	protein_coding	76.5586371	-2.871965	0.21092165	-13.616265	3.2055E-42	5.4829E-41
IL20	ENSG00000162891	protein_coding	2.24494594	-1.3258139	0.35657829	-3.7181566	0.00020068	0.00036583
PIGR	ENSG00000162896	protein_coding	13977.2097	-1.4703636	0.30303137	-4.8521828	1.2211E-06	2.7711E-06
FCAMR	ENSG00000162897	protein_coding	908.576717	-1.5483672	0.2507435	-6.1751038	6.612E-10	1.9808E-09
LRRTM1	ENSG00000162951	protein_coding	9.09650294	-1.2265726	0.47341222	-2.5909188	0.00957201	0.01436537
NEUROD1	ENSG00000162992	protein_coding	1.97701383	-1.2101683	0.37556793	-3.2222353	0.00127195	0.00212252
VSNL1	ENSG00000163032	protein_coding	43.9693342	-1.8382607	0.30602857	-6.0068271	1.8919E-09	5.4641E-09
CFAP221	ENSG00000163075	protein_coding	605.60936	-1.4084008	0.12521193	-11.248136	2.3654E-29	2.2799E-28
STPG2	ENSG00000163116	protein_coding	35.5451596	-1.0962434	0.14762158	-7.4260371	1.119E-13	4.4008E-13
NEURL3	ENSG00000163121	protein_coding	231.786978	-1.0084356	0.14427827	-6.9895181	2.7583E-12	9.8339E-12
BNIPL	ENSG00000163141	protein_coding	58.0992103	-1.6032221	0.15419672	-10.397252	2.5519E-25	1.9905E-24
C1QTNF7	ENSG00000163145	protein_coding	247.951292	-1.6170768	0.17037891	-9.491062	2.2869E-21	1.44E-20
SPRR2D	ENSG00000163216	protein_coding	1.39018473	-1.5910737	0.5680278	-2.801049	0.00509368	0.00791926
GABRB1	ENSG00000163288	protein_coding	17.5511907	-1.1850837	0.25623753	-4.6249419	3.747E-06	8.1249E-06
NIPAL1	ENSG00000163293	protein_coding	346.721476	-3.2091586	0.18100409	-17.729757	2.471E-70	1.2025E-68
GPR155	ENSG00000163328	protein_coding	1539.36929	-1.1287163	0.12188462	-9.2605314	2.0342E-20	1.2135E-19
TAFA4	ENSG00000163377	protein_coding	4.11538556	-4.4622009	0.25330404	-17.615988	1.8572E-69	8.7588E-68
SLC22A15	ENSG00000163393	protein_coding	325.205217	-1.3007025	0.14333153	-9.0747827	1.139E-19	6.5244E-19

ATP1A1	ENSG00000163399	protein_coding	71053.5275	-2.7720038	0.09701323	-28.573463	1.436E-179	2.194E-176
SLC15A2	ENSG00000163406	protein_coding	493.506827	-3.073409	0.16160023	-19.018594	1.1965E-80	8.5302E-79
TEX55	ENSG00000163424	protein_coding	4.15494286	-1.1441168	0.50368992	-2.2714705	0.02311851	0.03286424
ELF3	ENSG00000163435	protein_coding	4688.66776	-1.1127462	0.14048831	-7.9205608	2.3644E-15	1.0398E-14
ADORA1	ENSG00000163485	protein_coding	448.379758	-1.1334803	0.19773531	-5.7323112	9.9071E-09	2.69E-08
HDAC11	ENSG00000163517	protein_coding	1512.87168	-1.0455042	0.09430838	-11.086016	1.4668E-28	1.3526E-27
DPPA2	ENSG00000163530	protein_coding	3.60325025	-1.880201	0.399999	-4.7005142	2.5951E-06	5.7105E-06
NFASC	ENSG00000163531	protein_coding	2835.38279	-1.7245553	0.14678331	-11.748988	7.147E-32	7.7416E-31
SUCLG1	ENSG00000163541	protein_coding	6295.90756	-1.7617537	0.08972737	-19.634519	7.842E-86	6.7572E-84
NUAK2	ENSG00000163545	protein_coding	1843.58568	-2.1239765	0.12925612	-16.432309	1.123E-60	3.8941E-59
FABP1	ENSG00000163586	protein_coding	651.608048	-4.4161359	0.3339867	-13.22249	6.5073E-40	1.0088E-38
CDS1	ENSG00000163624	protein_coding	1748.14058	-1.3916233	0.12288061	-11.325003	9.8675E-30	9.6591E-29
PTPN13	ENSG00000163629	protein_coding	3353.43775	-1.8551012	0.091185	-20.344367	5.208E-92	5.6842E-90
SYNPR	ENSG00000163630	protein_coding	1.24697421	-3.2015614	0.4361717	-7.3401403	2.1337E-13	8.2173E-13
ALB	ENSG00000163631	protein_coding	1687.51982	-5.0971487	0.27346789	-18.63893	1.5532E-77	9.9579E-76
PPM1K	ENSG00000163644	protein_coding	1538.65053	-1.5333259	0.08660688	-17.704436	3.8754E-70	1.878E-68
PTX3	ENSG00000163661	protein_coding	125.359395	-1.0001406	0.20301715	-4.9263849	8.3765E-07	1.9317E-06
DNASE1L3	ENSG00000163687	protein_coding	595.688725	-2.1852365	0.1641314	-13.313945	1.9207E-40	3.0539E-39
RBM47	ENSG00000163694	protein_coding	8602.71281	-1.0950282	0.08368031	-13.085853	3.9668E-39	5.9915E-38
IL17RE	ENSG00000163701	protein_coding	309.827365	-1.4439351	0.13448483	-10.736788	6.8382E-27	5.8061E-26
PRRT3	ENSG00000163704	protein_coding	150.191869	-1.2650644	0.10122557	-12.497479	7.7056E-36	1.0084E-34
LRRC2	ENSG00000163827	protein_coding	233.021741	-3.424578	0.23075346	-14.840852	7.9736E-50	1.8725E-48
LIPH	ENSG00000163898	protein_coding	443.655802	-3.5719978	0.2538625	-14.0706	5.7578E-45	1.1039E-43
ARHGEF3	ENSG00000163947	protein_coding	2300.55239	-1.0861035	0.0689476	-15.752594	6.5921E-56	1.9309E-54
MELTF	ENSG00000163975	protein_coding	925.409047	-2.4944337	0.24373119	-10.234364	1.3911E-24	1.0408E-23

CLDN19	ENSG00000164007	protein_coding	885.389427	-5.6948846	0.27806761	-20.480215	3.2323E-93	3.7323E-91
EMCN	ENSG00000164035	protein_coding	4937.13342	-1.1210508	0.13851391	-8.0934163	5.8014E-16	2.6499E-15
PLXNB1	ENSG00000164050	protein_coding	4850.20943	-1.4254212	0.08879542	-16.052868	5.4582E-58	1.7328E-56
APEH	ENSG00000164062	protein_coding	4584.38068	-1.225512	0.08556487	-14.322608	1.5808E-46	3.2423E-45
ETNPPL	ENSG00000164089	protein_coding	63.7176154	-2.4462788	0.33842811	-7.2283559	4.8888E-13	1.8359E-12
NDST3	ENSG00000164100	protein_coding	25.4765934	-3.0645254	0.21427814	-14.301624	2.1376E-46	4.3648E-45
MAP9	ENSG00000164114	protein_coding	1021.21017	-1.2785772	0.1228662	-10.406257	2.3217E-25	1.8147E-24
HPGD	ENSG00000164120	protein_coding	1291.94672	-1.9060381	0.22619852	-8.4263949	3.5648E-17	1.7598E-16
NPY1R	ENSG00000164128	protein_coding	1821.0973	-1.1962495	0.14223179	-8.4105636	4.0805E-17	2.0064E-16
NPY5R	ENSG00000164129	protein_coding	79.7464763	-2.4265756	0.17228873	-14.084354	4.7397E-45	9.1176E-44
RANBP3L	ENSG00000164188	protein_coding	522.438003	-5.2040735	0.23965821	-21.714564	1.495E-104	2.697E-102
ADGRV1	ENSG00000164199	protein_coding	284.816832	-2.7343284	0.17539543	-15.589507	8.5793E-55	2.4247E-53
CDC20B	ENSG00000164287	protein_coding	36.0034853	-1.2393818	0.2436282	-5.0871854	3.6342E-07	8.6605E-07
RHOBTB3	ENSG00000164292	protein_coding	3067.29855	-1.5621102	0.15268393	-10.231006	1.4402E-24	1.0764E-23
ENPP6	ENSG00000164303	protein_coding	283.319145	-5.0634414	0.19643986	-25.776039	1.646E-146	1.048E-143
TMEM174	ENSG00000164325	protein_coding	1384.94981	-2.2761793	0.28465082	-7.9963911	1.2812E-15	5.736E-15
FOXQ1	ENSG00000164379	protein_coding	460.399827	-1.2115878	0.15662563	-7.7355649	1.0295E-14	4.337E-14
ACSL6	ENSG00000164398	protein_coding	214.633541	-2.1592247	0.1776368	-12.155278	5.3779E-34	6.4586E-33
GRIK2	ENSG00000164418	protein_coding	37.8996888	-2.8177453	0.18614091	-15.1377	9.1353E-52	2.3213E-50
CALHM4	ENSG00000164451	protein_coding	4.49641562	-1.2631491	0.19322686	-6.5371301	6.271E-11	2.0273E-10
DACT2	ENSG00000164488	protein_coding	169.199377	-1.3078286	0.28780255	-4.5441868	5.5148E-06	1.1768E-05
RAET1E	ENSG00000164520	protein_coding	57.2820181	-1.8953085	0.11754324	-16.124351	1.7206E-58	5.5427E-57
PI16	ENSG00000164530	protein_coding	108.586625	-2.2914672	0.29842078	-7.6786451	1.6078E-14	6.6784E-14
KIF6	ENSG00000164627	protein_coding	89.2950272	-1.5428719	0.12958997	-11.905798	1.104E-32	1.2416E-31
SLC30A8	ENSG00000164756	protein_coding	57.4044738	-3.3382376	0.31392963	-10.633713	2.0768E-26	1.7187E-25

TNFRSF11B	ENSG00000164761	protein_coding	2549.74077	-1.1449585	0.14997277	-7.6344428	2.268E-14	9.3269E-14
DEFB1	ENSG00000164825	protein_coding	9328.19491	-1.7000491	0.26336417	-6.4551268	1.0813E-10	3.4331E-10
UNCX	ENSG00000164853	protein_coding	10.7647437	-7.3183028	0.32967123	-22.198791	3.528E-109	7.318E-107
SLC7A13	ENSG00000164893	protein_coding	155.751759	-5.0054533	0.46390766	-10.789762	3.8478E-27	3.31E-26
FREM1	ENSG00000164946	protein_coding	616.926906	-3.3165178	0.24664991	-13.446256	3.2386E-41	5.3097E-40
C9orf24	ENSG00000164972	protein_coding	60.4297839	-1.0338506	0.19112829	-5.4091973	6.3308E-08	1.6101E-07
MYORG	ENSG00000164976	protein_coding	1463.79219	-1.5357356	0.10197132	-15.060466	2.947E-51	7.3262E-50
MAMDC2	ENSG00000165072	protein_coding	204.152426	-1.9785964	0.16460212	-12.02048	2.7736E-33	3.2196E-32
TMC1	ENSG00000165091	protein_coding	26.960656	-2.1333083	0.15170646	-14.06208	6.4949E-45	1.2426E-43
SVEP1	ENSG00000165124	protein_coding	1244.66375	-1.5752199	0.17244833	-9.1344454	6.5743E-20	3.8216E-19
TRPV6	ENSG00000165125	protein_coding	133.368423	-4.8469477	0.20568434	-23.564982	8.815E-123	2.676E-120
LLCFC1	ENSG00000165131	protein_coding	1.69675579	-1.0319865	0.1906379	-5.4133333	6.1862E-08	1.5746E-07
FBP1	ENSG00000165140	protein_coding	3649.18269	-2.361061	0.15494231	-15.238323	1.9686E-52	5.1332E-51
SHOC1	ENSG00000165181	protein_coding	111.217537	-1.0710773	0.27282213	-3.9259179	8.64E-05	0.00016374
CXorf58	ENSG00000165182	protein_coding	5.84182239	-1.9042431	0.11440311	-16.64503	3.288E-62	1.1943E-60
PTCHD1	ENSG00000165186	protein_coding	26.1243571	-1.1915686	0.28771305	-4.1415175	3.4502E-05	6.8195E-05
VEGFD	ENSG00000165197	protein_coding	61.4935513	-1.0210442	0.18371404	-5.5577905	2.7321E-08	7.1522E-08
AQP7	ENSG00000165269	protein_coding	734.81892	-1.0621512	0.17073061	-6.2212109	4.9333E-10	1.4937E-09
AQP3	ENSG00000165272	protein_coding	9395.65241	-2.2091725	0.14339627	-15.406067	1.4901E-53	4.0419E-52
SLC7A3	ENSG00000165349	protein_coding	2.9912876	-2.463206	0.34195656	-7.2032718	5.8785E-13	2.1957E-12
SLC16A9	ENSG00000165449	protein_coding	5817.97669	-1.0990075	0.20997939	-5.2338827	1.6599E-07	4.0727E-07
CRYL1	ENSG00000165475	protein_coding	9455.54073	-1.084869	0.10443476	-10.388007	2.8117E-25	2.1872E-24
HEPACAM	ENSG00000165478	protein_coding	5.28347647	-1.1538846	0.25392558	-4.5441842	5.5148E-06	1.1768E-05
PKNOX2	ENSG00000165495	protein_coding	123.327261	-1.580086	0.16312272	-9.6864863	3.4417E-22	2.2609E-21
EML5	ENSG00000165521	protein_coding	45.9066975	-1.351056	0.17521877	-7.7106808	1.2515E-14	5.2373E-14

AMER2	ENSG00000165566	protein_coding	5.27884394	-2.4760934	0.32279542	-7.6707822	1.7095E-14	7.0881E-14
OXGR1	ENSG00000165621	protein_coding	110.80715	-2.5983449	0.34028776	-7.6357282	2.2455E-14	9.2401E-14
ZNF503	ENSG00000165655	protein_coding	1280.85352	-1.9294889	0.09895972	-19.497719	1.1479E-84	9.4641E-83
TMEM52B	ENSG00000165685	protein_coding	1984.80823	-5.0317032	0.26612273	-18.907454	9.9024E-80	6.8674E-78
FRMD7	ENSG00000165694	protein_coding	51.4523885	-3.656101	0.48951133	-7.4688792	8.0881E-14	3.2109E-13
SPACA9	ENSG00000165698	protein_coding	497.177679	-1.1442736	0.07939658	-14.412127	4.3413E-47	9.108E-46
STOX1	ENSG00000165730	protein_coding	151.32675	-1.705222	0.18410407	-9.2622718	2.0013E-20	1.1945E-19
NDRG2	ENSG00000165795	protein_coding	6957.72955	-1.323083	0.11211784	-11.800825	3.8651E-32	4.2428E-31
PPP1R36	ENSG00000165807	protein_coding	73.3241899	-2.411102	0.20272679	-11.893357	1.2815E-32	1.4379E-31
CCDC186	ENSG00000165813	protein_coding	1855.19932	-1.2950968	0.07449916	-17.384045	1.0899E-67	4.7901E-66
VWA2	ENSG00000165816	protein_coding	49.3101469	-2.9085363	0.21819908	-13.329737	1.5544E-40	2.482E-39
PRAP1	ENSG00000165828	protein_coding	1901.33037	-1.1877564	0.26043377	-4.5606853	5.0987E-06	1.0914E-05
C10orf82	ENSG00000165863	protein_coding	19.9535937	-2.7993428	0.22519939	-12.430508	1.785E-35	2.303E-34
ANKRD2	ENSG00000165887	protein_coding	186.898741	-3.2128354	0.27570286	-11.653254	2.2086E-31	2.3415E-30
OTOGL	ENSG00000165899	protein_coding	181.99538	-2.5153414	0.18004608	-13.970543	2.3582E-44	4.4196E-43
LARGE2	ENSG00000165905	protein_coding	697.892597	-1.4110855	0.17379823	-8.119102	4.6965E-16	2.1583E-15
CLMN	ENSG00000165959	protein_coding	4029.63539	-1.3939122	0.10203291	-13.661399	1.7264E-42	2.9931E-41
PDZRN4	ENSG00000165966	protein_coding	74.9001625	-1.330136	0.25975589	-5.1207156	3.0438E-07	7.3014E-07
NELL1	ENSG00000165973	protein_coding	364.974666	-5.7951581	0.41126932	-14.090908	4.3197E-45	8.3272E-44
HACD1	ENSG00000165996	protein_coding	119.370041	-1.0724625	0.15578665	-6.884175	5.8123E-12	2.0248E-11
ABTB2	ENSG00000166016	protein_coding	1085.83984	-1.6147679	0.14538436	-11.106889	1.1614E-28	1.0778E-27
ADAMTS15	ENSG00000166106	protein_coding	696.727417	-1.9579693	0.15150206	-12.923714	3.3078E-38	4.797E-37
GPT2	ENSG00000166123	protein_coding	1503.49699	-1.1225155	0.13995633	-8.0204697	1.0534E-15	4.7375E-15
MOGAT2	ENSG00000166391	protein_coding	23.7117366	-4.2618781	0.32095332	-13.27881	3.0724E-40	4.8399E-39
SERPINB7	ENSG00000166396	protein_coding	15.0852999	-1.3419094	0.287235	-4.6718171	2.9855E-06	6.5357E-06

RIC3	ENSG00000166405	protein_coding	296.673807	-1.069484	0.24658205	-4.3372338	1.4429E-05	2.9581E-05
WDR72	ENSG00000166415	protein_coding	4774.11235	-1.8892481	0.16813736	-11.236337	2.7037E-29	2.6016E-28
CRABP1	ENSG00000166426	protein_coding	80.2790031	-2.0255646	0.50482534	-4.0124067	6.0103E-05	0.00011572
PRTG	ENSG00000166450	protein_coding	168.843544	-2.0014313	0.16055054	-12.466051	1.1435E-35	1.4867E-34
CLEC3A	ENSG00000166509	protein_coding	3.72057711	-2.4811368	0.37653812	-6.5893377	4.4179E-11	1.4451E-10
CDH16	ENSG00000166589	protein_coding	21125.2389	-2.3690328	0.14371922	-16.483757	4.8006E-61	1.6825E-59
BMERB1	ENSG00000166780	protein_coding	883.593395	-1.0709582	0.08754216	-12.233627	2.0555E-34	2.5107E-33
LDHC	ENSG00000166796	protein_coding	31.3921776	-1.4814675	0.250078	-5.9240218	3.1416E-09	8.9035E-09
LDHD	ENSG00000166816	protein_coding	1187.6047	-2.268937	0.18202658	-12.464866	1.1607E-35	1.5081E-34
SCNN1G	ENSG00000166828	protein_coding	1930.31872	-4.7349233	0.38794729	-12.205068	2.9206E-34	3.5466E-33
NAV2	ENSG00000166833	protein_coding	2123.72068	-1.6208687	0.08827002	-18.36262	2.6167E-75	1.5418E-73
GLYATL1	ENSG00000166840	protein_coding	4232.4788	-1.4515498	0.19428965	-7.4710608	7.9551E-14	3.1598E-13
GPR182	ENSG00000166856	protein_coding	45.9720754	-2.4393996	0.16910135	-14.425666	3.5681E-47	7.5134E-46
CHP2	ENSG00000166869	protein_coding	19.505395	-5.3097327	0.2709754	-19.594888	1.7096E-85	1.4513E-83
PIP4K2C	ENSG00000166908	protein_coding	2834.18321	-1.4163859	0.06483616	-21.845617	8.556E-106	1.594E-103
MTMR10	ENSG00000166912	protein_coding	3928.40514	-1.1584126	0.06468241	-17.909236	9.9897E-72	5.1166E-70
GREM1	ENSG00000166923	protein_coding	464.831544	-2.423351	0.28952345	-8.3701369	5.7551E-17	2.8033E-16
NYAP1	ENSG00000166924	protein_coding	30.9988735	-2.614785	0.15250293	-17.145803	6.756E-66	2.7901E-64
CCDC178	ENSG00000166960	protein_coding	129.529909	-1.4906594	0.15223001	-9.7921519	1.2168E-22	8.1931E-22
TCP10L2	ENSG00000166984	protein_coding	3.47279414	-1.6517034	0.26362411	-6.2653729	3.7193E-10	1.1358E-09
B4GALNT2	ENSG00000167080	protein_coding	573.825061	-3.6076997	0.29162751	-12.370917	3.7551E-35	4.7512E-34
FAM102A	ENSG00000167106	protein_coding	3971.86286	-1.0673279	0.13076913	-8.1619257	3.2973E-16	1.5289E-15
ACSF2	ENSG00000167107	protein_coding	1908.02673	-3.2985896	0.14067447	-23.448388	1.373E-121	4.034E-119
SLC27A4	ENSG00000167114	protein_coding	1594.16377	-1.004449	0.08970109	-11.197735	4.1829E-29	3.979E-28
PRR15L	ENSG00000167183	protein_coding	617.783871	-2.0888963	0.2780531	-7.5125806	5.7973E-14	2.3236E-13

GPRC5B	ENSG00000167191	protein_coding	4634.22258	-1.2095462	0.10622104	-11.387068	4.8504E-30	4.8085E-29
RBFOX3	ENSG00000167281	protein_coding	13.7273266	-1.1981083	0.21630429	-5.5389947	3.0421E-08	7.933E-08
CA4	ENSG00000167434	protein_coding	666.552977	-1.4900749	0.21714031	-6.8622674	6.7776E-12	2.3509E-11
AQP2	ENSG00000167580	protein_coding	23239.9844	-8.9127805	0.49104155	-18.150767	1.2662E-73	6.9849E-72
GPD1	ENSG00000167588	protein_coding	4154.34525	-1.6825087	0.21358049	-7.8776328	3.3364E-15	1.4511E-14
CYP2S1	ENSG00000167600	protein_coding	218.665599	-1.0532191	0.13936042	-7.5575198	4.1083E-14	1.6616E-13
TMC4	ENSG00000167608	protein_coding	1562.6543	-2.0689725	0.20318288	-10.182809	2.3663E-24	1.7487E-23
PSCA	ENSG00000167653	protein_coding	60.3515374	-2.955279	0.31159487	-9.4843636	2.4387E-21	1.5326E-20
MFSD3	ENSG00000167700	protein_coding	702.93198	-1.0620844	0.12168229	-8.7283405	2.5844E-18	1.3677E-17
SLC43A2	ENSG00000167703	protein_coding	6203.70456	-1.0842496	0.09899126	-10.952983	6.4295E-28	5.7498E-27
GGT6	ENSG00000167741	protein_coding	827.611618	-4.7096393	0.32947771	-14.294258	2.3762E-46	4.8349E-45
KLK1	ENSG00000167748	protein_coding	1077.96216	-1.2758971	0.47121988	-2.7076469	0.00677621	0.0103823
KLK5	ENSG00000167754	protein_coding	10.8611448	-3.536942	0.41795464	-8.4625021	2.617E-17	1.3034E-16
KLK6	ENSG00000167755	protein_coding	114.478811	-5.4261187	0.418582	-12.963096	1.9809E-38	2.8919E-37
KLK11	ENSG00000167757	protein_coding	6.66558452	-1.3706133	0.43409746	-3.1573862	0.0015919	0.00262668
EVPL	ENSG00000167880	protein_coding	1125.67704	-1.5563114	0.11539728	-13.486552	1.8767E-41	3.1171E-40
KRT24	ENSG00000167916	protein_coding	2.53320958	-2.2403038	0.30941844	-7.2403693	4.4746E-13	1.6844E-12
SOST	ENSG00000167941	protein_coding	146.232612	-6.8430868	0.33541293	-20.401977	1.6059E-92	1.8088E-90
ENTPD3	ENSG00000168032	protein_coding	43.1819469	-3.2400043	0.20697725	-15.653915	3.1239E-55	8.9503E-54
SCARA3	ENSG00000168077	protein_coding	1625.1653	-1.1820853	0.18424571	-6.4158091	1.4008E-10	4.4093E-10
SCARA5	ENSG00000168079	protein_coding	79.7544649	-1.9289158	0.29715678	-6.4912396	8.5133E-11	2.7258E-10
FAM83B	ENSG00000168143	protein_coding	110.621169	-3.4344925	0.40140273	-8.556226	1.1662E-17	5.9263E-17
GLYCTK	ENSG00000168237	protein_coding	788.006837	-1.0750268	0.11418844	-9.4144975	4.7535E-21	2.9373E-20
PDHB	ENSG00000168291	protein_coding	3089.66759	-1.1617235	0.06585699	-17.640093	1.2126E-69	5.7663E-68
ACOX2	ENSG00000168306	protein_coding	936.649276	-1.9143946	0.12791222	-14.966472	1.216E-50	2.9588E-49

FAM107A	ENSG00000168309	protein_coding	2293.90666	-1.5439686	0.12735889	-12.122975	7.9809E-34	9.5101E-33
DEGS2	ENSG00000168350	protein_coding	387.922347	-2.9854587	0.17247597	-17.309419	3.9943E-67	1.7306E-65
BDKRB2	ENSG00000168398	protein_coding	1666.82545	-1.1587115	0.19944802	-5.8095916	6.2625E-09	1.7304E-08
MTNR1A	ENSG00000168412	protein_coding	12.029794	-3.0176277	0.29202906	-10.333313	4.981E-25	3.8138E-24
SCNN1B	ENSG00000168447	protein_coding	1110.04733	-3.9727714	0.30342588	-13.093054	3.6081E-39	5.4586E-38
TNXB	ENSG00000168477	protein_coding	1055.42659	-1.1358166	0.15491204	-7.3320101	2.2673E-13	8.7141E-13
PHYHIP	ENSG00000168490	protein_coding	101.884006	-1.8393384	0.15180976	-12.116075	8.6819E-34	1.0316E-32
CHRM1	ENSG00000168539	protein_coding	10.8616125	-1.8240075	0.2705706	-6.7413367	1.5694E-11	5.3019E-11
SLC20A2	ENSG00000168575	protein_coding	2115.49455	-1.392815	0.06200526	-22.462853	9.583E-112	2.133E-109
GDNF	ENSG00000168621	protein_coding	42.4353704	-1.8544523	0.2859547	-6.485126	8.8658E-11	2.8353E-10
AXIN2	ENSG00000168646	protein_coding	166.961639	-1.1255883	0.10999803	-10.232804	1.4137E-24	1.057E-23
VWA3B	ENSG00000168658	protein_coding	12.7839984	-2.2599544	0.1838261	-12.29398	9.7581E-35	1.2136E-33
UGT3A2	ENSG00000168671	protein_coding	65.3392996	-1.8044821	0.29578268	-6.1007026	1.056E-09	3.1138E-09
LRP1B	ENSG00000168702	protein_coding	61.4291548	-2.5595026	0.26164645	-9.7822943	1.3413E-22	9.012E-22
WFDC12	ENSG00000168703	protein_coding	7.54705838	-1.174899	0.41652143	-2.8207407	0.00479129	0.00747575
NPNT	ENSG00000168743	protein_coding	4437.67211	-2.0218277	0.14923587	-13.547867	8.1566E-42	1.3696E-40
NSG1	ENSG00000168824	protein_coding	128.358372	-1.2542864	0.23547273	-5.3266737	1.0003E-07	2.4999E-07
PLA2G4F	ENSG00000168907	protein_coding	665.358904	-2.2385013	0.47883645	-4.6748766	2.9413E-06	6.4421E-06
ENHO	ENSG00000168913	protein_coding	18.5145041	-1.5456893	0.14908938	-10.367535	3.484E-25	2.6919E-24
UQCRFS1	ENSG00000169021	protein_coding	3758.92691	-1.2242964	0.09856309	-12.421449	1.9991E-35	2.572E-34
COL4A3	ENSG00000169031	protein_coding	1699.40218	-1.9798595	0.14918098	-13.271528	3.386E-40	5.3249E-39
KLK7	ENSG00000169035	protein_coding	78.4848935	-5.8163653	0.41106132	-14.149629	1.8774E-45	3.6748E-44
IRS1	ENSG00000169047	protein_coding	1608.90031	-1.0383114	0.13707075	-7.5750033	3.5912E-14	1.4579E-13
PARM1	ENSG00000169116	protein_coding	4906.27145	-1.1437457	0.1144084	-9.997043	1.5702E-23	1.1068E-22
AFAP1L2	ENSG00000169129	protein_coding	2052.33931	-1.8670803	0.09998499	-18.673606	8.1182E-78	5.2787E-76

PCSK9	ENSG00000169174	protein_coding	16.2477701	-1.3297118	0.31807666	-4.1804759	2.909E-05	5.7903E-05
GSG1L	ENSG00000169181	protein_coding	5.03133103	-1.7490245	0.27943612	-6.259121	3.8715E-10	1.181E-09
RAB3B	ENSG00000169213	protein_coding	151.943098	-2.1115852	0.27806735	-7.5937904	3.1068E-14	1.2661E-13
RSPO1	ENSG00000169218	protein_coding	9.31597373	-2.9943625	0.33445463	-8.9529706	3.4604E-19	1.9291E-18
HSPB3	ENSG00000169271	protein_coding	5.52065092	-1.1007246	0.35579767	-3.0936812	0.0019769	0.00322513
STK32A	ENSG00000169302	protein_coding	321.760319	-1.3364237	0.24937401	-5.3591138	8.3631E-08	2.1062E-07
PDILT	ENSG00000169340	protein_coding	4.69494658	-3.0827238	0.19369477	-15.915369	4.9574E-57	1.51E-55
UMOD	ENSG00000169344	protein_coding	65470.7177	-8.4940238	0.47727911	-17.796764	7.4872E-71	3.7226E-69
GP2	ENSG00000169347	protein_coding	88.9504375	-6.8794391	0.35903795	-19.160758	7.8719E-82	5.8109E-80
SPRR1A	ENSG00000169474	protein_coding	1.76505541	-1.1996785	0.5386307	-2.2272747	0.02592892	0.03659507
CCDC8	ENSG00000169515	protein_coding	1151.5641	-1.312739	0.1899433	-6.9112151	4.8052E-12	1.6834E-11
MUC15	ENSG00000169550	protein_coding	661.594303	-7.2307325	0.42100228	-17.175044	4.0835E-66	1.7017E-64
GJB1	ENSG00000169562	protein_coding	1188.84104	-1.4893003	0.16588102	-8.9781238	2.7542E-19	1.5433E-18
DCXR	ENSG00000169738	protein_coding	2439.39349	-2.0826292	0.13689271	-15.213587	2.8736E-52	7.4717E-51
TACR3	ENSG00000169836	protein_coding	9.19463573	-3.9670396	0.35799008	-11.081423	1.544E-28	1.4215E-27
PCDH7	ENSG00000169851	protein_coding	405.88961	-1.387154	0.18981749	-7.3078305	2.7149E-13	1.0376E-12
CHD3	ENSG00000170004	protein_coding	6354.21798	-1.0223367	0.08293153	-12.327479	6.4431E-35	8.0699E-34
RNF150	ENSG00000170153	protein_coding	1197.12167	-3.1037394	0.16415952	-18.90685	1.0016E-79	6.936E-78
GYPA	ENSG00000170180	protein_coding	39.9822952	-1.2218029	0.26339593	-4.6386553	3.5068E-06	7.626E-06
SLC16A5	ENSG00000170190	protein_coding	870.23758	-3.0210841	0.15017563	-20.117006	5.2377E-90	5.2556E-88
CST5	ENSG00000170367	protein_coding	10.1468346	-1.4166803	0.39237413	-3.6105343	0.00030557	0.00054548
LRRN2	ENSG00000170382	protein_coding	623.805521	-2.3104993	0.23006096	-10.042987	9.8641E-24	7.0301E-23
ZNF804A	ENSG00000170396	protein_coding	48.0606039	-1.2549441	0.15790345	-7.9475404	1.9025E-15	8.4264E-15
SLC23A1	ENSG00000170482	protein_coding	939.959575	-1.6100208	0.19626733	-8.2032031	2.3407E-16	1.0959E-15
LONRF2	ENSG00000170500	protein_coding	419.027802	-1.8684446	0.21941567	-8.5155476	1.658E-17	8.3542E-17

PFKFB3	ENSG00000170525	protein_coding	15146.888	-1.261146	0.11403593	-11.059199	1.9786E-28	1.8133E-27
IRX1	ENSG00000170549	protein_coding	154.180749	-4.5755294	0.2423514	-18.879732	1.6743E-79	1.1456E-77
IRX2	ENSG00000170561	protein_coding	241.714034	-5.0276362	0.19483245	-25.804922	7.808E-147	5.041E-144
SIX2	ENSG00000170577	protein_coding	49.1348877	-1.0311617	0.21806524	-4.7286846	2.2598E-06	5.001E-06
DLGAP1	ENSG00000170579	protein_coding	118.41854	-1.7400605	0.22212769	-7.8336048	4.7408E-15	2.0433E-14
FOXA3	ENSG00000170608	protein_coding	52.3740228	-2.8215942	0.29694382	-9.5021148	2.0567E-21	1.2977E-20
OR10A3	ENSG00000170683	protein_coding	0.26950593	-1.7702888	0.51849922	-3.4142556	0.00063957	0.0011029
HOXB9	ENSG00000170689	protein_coding	742.990607	-3.2719674	0.23279342	-14.05524	7.1539E-45	1.3659E-43
CEL	ENSG00000170835	protein_coding	117.807	-3.7332615	0.22254667	-16.775185	3.707E-63	1.3861E-61
GPR27	ENSG00000170837	protein_coding	185.916153	-1.8389836	0.18143143	-10.13597	3.8256E-24	2.7889E-23
RIOX2	ENSG00000170854	protein_coding	799.932388	-1.2156792	0.09675109	-12.565018	3.288E-36	4.3777E-35
PAQR8	ENSG00000170915	protein_coding	1040.02691	-1.3397745	0.09196515	-14.568285	4.4697E-48	9.7942E-47
PKHD1	ENSG00000170927	protein_coding	4489.61478	-1.2621636	0.17379533	-7.2623559	3.8041E-13	1.4386E-12
HAS2	ENSG00000170961	protein_coding	108.569313	-1.0301854	0.20172406	-5.1069043	3.2748E-07	7.833E-07
HS6ST2	ENSG00000171004	protein_coding	397.519403	-6.2620559	0.26817939	-23.350251	1.37E-120	3.83E-118
PYGO1	ENSG00000171016	protein_coding	206.625329	-1.6250497	0.17009292	-9.5538938	1.2491E-21	7.9717E-21
PATE1	ENSG00000171053	protein_coding	0.83165028	-2.9287362	0.46894685	-6.2453478	4.2286E-10	1.2871E-09
FUT3	ENSG00000171124	protein_coding	163.333726	-2.3221591	0.22208346	-10.456245	1.3718E-25	1.0856E-24
KCNG3	ENSG00000171126	protein_coding	11.2989055	-1.6222746	0.39731524	-4.0830917	4.444E-05	8.6874E-05
ATP6V0E2	ENSG00000171130	protein_coding	2010.5506	-1.2892761	0.11974699	-10.766668	4.9458E-27	4.2314E-26
UGT2B7	ENSG00000171234	protein_coding	15711.7727	-1.3226943	0.16250438	-8.1394377	3.9712E-16	1.8323E-15
SOSTDC1	ENSG00000171243	protein_coding	531.867659	-2.9162394	0.28855899	-10.106216	5.1849E-24	3.753E-23
NPTX1	ENSG00000171246	protein_coding	42.6304392	-1.0757611	0.2317477	-4.6419492	3.4514E-06	7.5101E-06
LURAP1	ENSG00000171357	protein_coding	149.353502	-1.7775097	0.12812689	-13.873042	9.2279E-44	1.6921E-42
CLCN5	ENSG00000171365	protein_coding	5827.49943	-1.4334185	0.1200511	-11.94007	7.3162E-33	8.3159E-32

XAGE3	ENSG00000171402	protein_coding	1.88083744	-1.1164308	0.23712112	-4.7082723	2.4983E-06	5.5056E-06
GLOD5	ENSG00000171433	protein_coding	59.7205395	-2.4118635	0.19529154	-12.350066	4.8671E-35	6.1295E-34
KSR2	ENSG00000171435	protein_coding	201.447714	-3.230189	0.21174072	-15.255399	1.5157E-52	3.9794E-51
PTGER4	ENSG00000171522	protein_coding	772.566346	-1.0179497	0.10225067	-9.9554332	2.3878E-23	1.665E-22
MAP6	ENSG00000171533	protein_coding	354.818098	-2.4045776	0.13611449	-17.665845	7.6853E-70	3.6775E-68
SLC25A33	ENSG00000171612	protein_coding	386.638094	-1.7345975	0.09370438	-18.511381	1.6716E-76	1.0369E-74
ANO5	ENSG00000171714	protein_coding	398.162356	-1.9596226	0.26604333	-7.3658026	1.7608E-13	6.8325E-13
SPATA46	ENSG00000171722	protein_coding	4.88379409	-1.6371639	0.2046362	-8.000363	1.2405E-15	5.5583E-15
GPHN	ENSG00000171723	protein_coding	1403.92679	-1.1566808	0.11908658	-9.7129402	2.6556E-22	1.7536E-21
VAT1L	ENSG00000171724	protein_coding	426.926846	-2.4050914	0.27420149	-8.7712558	1.7668E-18	9.452E-18
PAH	ENSG00000171759	protein_coding	3935.1818	-2.9020716	0.3233492	-8.9750388	2.8325E-19	1.5862E-18
GATM	ENSG00000171766	protein_coding	26660.6938	-2.2086006	0.16075057	-13.739302	5.9042E-43	1.0478E-41
CFAP46	ENSG00000171811	protein_coding	136.580569	-1.0670786	0.1715414	-6.2205311	4.9547E-10	1.4998E-09
PCDHB1	ENSG00000171815	protein_coding	29.3667973	-2.8530575	0.2597144	-10.985365	4.4942E-28	4.0499E-27
ANGPTL7	ENSG00000171819	protein_coding	30.1613362	-1.1464004	0.34164604	-3.3555208	0.00079216	0.0013519
ADRA1D	ENSG00000171873	protein_coding	14.5341112	-1.0594755	0.19870064	-5.3320185	9.7127E-08	2.431E-07
AQP4	ENSG00000171885	protein_coding	277.613536	-1.3430536	0.20515354	-6.5465779	5.887E-11	1.9067E-10
TLN2	ENSG00000171914	protein_coding	5850.95945	-1.1530854	0.1028779	-11.208291	3.7129E-29	3.5452E-28
ZNF556	ENSG00000172000	protein_coding	41.537763	-1.1867927	0.19177924	-6.1883272	6.0806E-10	1.8273E-09
MAL	ENSG00000172005	protein_coding	5765.92541	-3.2746424	0.25813427	-12.685811	7.0877E-37	9.7335E-36
CCL11	ENSG00000172156	protein_coding	40.1022345	-2.4240026	0.28839233	-8.4052257	4.2704E-17	2.0982E-16
PRL	ENSG00000172179	protein_coding	1.88985091	-2.1386228	0.25324464	-8.4448888	3.0434E-17	1.5094E-16
GPR22	ENSG00000172209	protein_coding	15.1427593	-1.8398991	0.34421448	-5.3452111	9.0312E-08	2.2662E-07
SUCLG2	ENSG00000172340	protein_coding	4671.25878	-1.2854121	0.08072856	-15.922644	4.4133E-57	1.3487E-55
CSDC2	ENSG00000172346	protein_coding	819.221594	-1.3896603	0.27079286	-5.1318202	2.8695E-07	6.8993E-07

ARNT2	ENSG00000172379	protein_coding	2721.85301	-1.7745108	0.14837347	-11.959758	5.773E-33	6.5978E-32
MYOZ2	ENSG00000172399	protein_coding	14.577639	-3.1386038	0.16759181	-18.727669	2.9453E-78	1.9427E-76
TTC36	ENSG00000172425	protein_coding	47.7907913	-2.7737131	0.19079949	-14.53732	7.0291E-48	1.5214E-46
FUT9	ENSG00000172461	protein_coding	49.4810752	-1.3269476	0.40352229	-3.2884121	0.00100754	0.00170023
MAB21L4	ENSG00000172478	protein_coding	161.020415	-3.8297517	0.32723293	-11.703442	1.2239E-31	1.3142E-30
AGXT	ENSG00000172482	protein_coding	191.653616	-3.6381937	0.20903664	-17.404574	7.617E-68	3.3802E-66
ACOT12	ENSG00000172497	protein_coding	24.1074794	-4.2927547	0.2970077	-14.453345	2.388E-47	5.0633E-46
MS4A10	ENSG00000172689	protein_coding	12.3709234	-1.3334876	0.40520515	-3.290895	0.00099869	0.00168629
COL6A5	ENSG00000172752	protein_coding	6.17090727	-1.6967666	0.31122171	-5.4519544	4.9819E-08	1.2771E-07
FADS6	ENSG00000172782	protein_coding	30.3876494	-1.0879025	0.35536807	-3.0613399	0.00220349	0.00357608
OVOL1	ENSG00000172818	protein_coding	232.587416	-2.3319198	0.19127552	-12.191418	3.4536E-34	4.1827E-33
PDP2	ENSG00000172840	protein_coding	893.899017	-1.5122346	0.09498855	-15.920178	4.5907E-57	1.4016E-55
MRGPRF	ENSG00000172935	protein_coding	279.965171	-2.2137793	0.18917375	-11.70236	1.2396E-31	1.3304E-30
SLC22A13	ENSG00000172940	protein_coding	160.255322	-2.9186751	0.26599244	-10.972775	5.1662E-28	4.6427E-27
ADH6	ENSG00000172955	protein_coding	675.527368	-2.6355682	0.20302099	-12.981752	1.5529E-38	2.2794E-37
FRG2C	ENSG00000172969	protein_coding	40.6857244	-4.09976	0.33637083	-12.188215	3.592E-34	4.3458E-33
HPSE2	ENSG00000172987	protein_coding	64.351201	-2.3844299	0.21818206	-10.928625	8.4113E-28	7.4783E-27
RXFP4	ENSG00000173080	protein_coding	4.1697381	-2.4192301	0.18455889	-13.108175	2.9563E-39	4.4844E-38
DMRT2	ENSG00000173253	protein_coding	803.811672	-1.6398925	0.49359865	-3.3223197	0.00089272	0.00151523
TRIB1	ENSG00000173334	protein_coding	3373.04981	-1.0018126	0.11744263	-8.5302296	1.4606E-17	7.3876E-17
CST9	ENSG00000173335	protein_coding	2.82099792	-5.4990693	0.33038261	-16.644549	3.3146E-62	1.203E-60
NDNF	ENSG00000173376	protein_coding	1390.34297	-2.4405751	0.33802229	-7.2201602	5.1926E-13	1.9454E-12
DAG1	ENSG00000173402	protein_coding	6919.04807	-1.1535924	0.06617567	-17.432272	4.6943E-68	2.0953E-66
AGR3	ENSG00000173467	protein_coding	30.3515974	-1.9981377	0.44220095	-4.5186192	6.2244E-06	1.322E-05
MOB1B	ENSG00000173542	protein_coding	3895.59557	-1.0277807	0.14071143	-7.3041732	2.7898E-13	1.0649E-12

ADGRF3	ENSG00000173567	protein_coding	97.5885024	-3.5465226	0.12603776	-28.138572	3.307E-174	4.458E-171
CCR9	ENSG00000173585	protein_coding	13.2093518	-1.4970169	0.18961246	-7.8951396	2.8999E-15	1.267E-14
NUDT4	ENSG00000173598	protein_coding	3134.85771	-1.8025859	0.08718879	-20.674514	5.8756E-95	7.2795E-93
PC	ENSG00000173599	protein_coding	2125.96084	-1.4307721	0.13676236	-10.461739	1.2946E-25	1.0266E-24
UGT2A1	ENSG00000173610	protein_coding	51.7636279	-4.6970073	0.49816138	-9.4286861	4.1526E-21	2.5759E-20
HSPB7	ENSG00000173641	protein_coding	680.916418	-3.2424904	0.23037504	-14.074834	5.4232E-45	1.0415E-43
TAS1R1	ENSG00000173662	protein_coding	9.25513759	-1.0911884	0.17677236	-6.1728452	6.7072E-10	2.0083E-09
MUC13	ENSG00000173702	protein_coding	436.718663	-2.5354112	0.28681625	-8.8398452	9.5851E-19	5.2073E-18
WFIKKN2	ENSG00000173714	protein_coding	10.0616087	-1.9045843	0.30296015	-6.2865837	3.2453E-10	9.951E-10
CNP	ENSG00000173786	protein_coding	5613.13412	-1.3129979	0.06046096	-21.71646	1.434E-104	2.609E-102
JUP	ENSG00000173801	protein_coding	14715.1681	-1.0378473	0.08425217	-12.318345	7.216E-35	9.0182E-34
HAP1	ENSG00000173805	protein_coding	37.1521016	-1.5953636	0.23352228	-6.8317406	8.389E-12	2.8908E-11
KCNH6	ENSG00000173826	protein_coding	203.55301	-2.0169313	0.22937925	-8.7929979	1.4562E-18	7.8322E-18
MARCHF10	ENSG00000173838	protein_coding	56.0558267	-1.8721898	0.30589125	-6.1204426	9.3316E-10	2.7623E-09
SPTBN2	ENSG00000173898	protein_coding	2777.53013	-4.3120272	0.27076298	-15.925468	4.2185E-57	1.2918E-55
CBY2	ENSG00000174015	protein_coding	5.42733258	-1.9459956	0.31856204	-6.1086864	1.0045E-09	2.966E-09
TENT5D	ENSG00000174016	protein_coding	1.6019662	-3.1309952	0.28247737	-11.084056	1.4992E-28	1.3823E-27
SLC25A30	ENSG00000174032	protein_coding	3114.63145	-1.0219878	0.08251917	-12.384853	3.1566E-35	4.0118E-34
SELP	ENSG00000174175	protein_coding	754.363106	-1.1240707	0.13700182	-8.2047871	2.31E-16	1.0819E-15
ZHX3	ENSG00000174306	protein_coding	4577.31916	-1.3059205	0.07241131	-18.034758	1.0395E-72	5.5152E-71
SLC6A19	ENSG00000174358	protein_coding	4411.85815	-2.2141235	0.33454634	-6.6182863	3.6339E-11	1.1958E-10
LINGO2	ENSG00000174482	protein_coding	18.5101861	-2.4416096	0.2869458	-8.5089576	1.755E-17	8.8332E-17
IGDCC3	ENSG00000174498	protein_coding	22.6536989	-1.7211603	0.2182256	-7.8870687	3.0937E-15	1.3487E-14
MFSD4A	ENSG00000174514	protein_coding	3784.53336	-5.2474125	0.12572422	-41.737485	0	0
AKIRIN1	ENSG00000174574	protein_coding	2774.00288	-1.0981817	0.06743047	-16.286134	1.2381E-59	4.1459E-58

TRAM1L1	ENSG00000174599	protein_coding	346.250649	-1.5100703	0.10013533	-15.080294	2.1829E-51	5.4533E-50
UGT8	ENSG00000174607	protein_coding	2593.5182	-1.7084058	0.15077861	-11.330558	9.2612E-30	9.0792E-29
SLC29A2	ENSG00000174669	protein_coding	392.714737	-1.8793701	0.17193214	-10.930883	8.2046E-28	7.3016E-27
WDR49	ENSG00000174776	protein_coding	7.93174359	-2.0572701	0.20297492	-10.135588	3.8406E-24	2.799E-23
BTC	ENSG00000174808	protein_coding	247.262695	-1.7931647	0.14263281	-12.571895	3.0141E-36	4.0201E-35
ZG16	ENSG00000174992	protein_coding	1.89232957	-2.423815	0.32867145	-7.3745832	1.6486E-13	6.4056E-13
SLC22A1	ENSG00000175003	protein_coding	37.1265841	-1.0769735	0.14538901	-7.4075305	1.2867E-13	5.038E-13
DES	ENSG00000175084	protein_coding	1150.82575	-1.7050587	0.29826108	-5.7166651	1.0863E-08	2.9403E-08
RAG2	ENSG00000175097	protein_coding	4.44535327	-3.8780006	0.23826966	-16.275679	1.4688E-59	4.9111E-58
CADM2	ENSG00000175161	protein_coding	14.2438129	-2.0623415	0.29709733	-6.941636	3.8758E-12	1.367E-11
PPM1E	ENSG00000175175	protein_coding	160.832176	-1.4160899	0.25166719	-5.6268357	1.8355E-08	4.8799E-08
PCCA	ENSG00000175198	protein_coding	3144.27314	-1.4788622	0.09883714	-14.962616	1.2886E-50	3.132E-49
GAL3ST3	ENSG00000175229	protein_coding	72.9655802	-4.3526804	0.30853969	-14.10736	3.4215E-45	6.6347E-44
PHYHD1	ENSG00000175287	protein_coding	699.790075	-2.2371269	0.18946425	-11.807646	3.564E-32	3.9264E-31
CST6	ENSG00000175315	protein_coding	29.9734347	-1.8214073	0.23687939	-7.6891758	1.4809E-14	6.1645E-14
ISX	ENSG00000175329	protein_coding	3.16204511	-2.6315112	0.4594768	-5.7271906	1.0211E-08	2.7696E-08
APOF	ENSG00000175336	protein_coding	2.02989054	-1.5358909	0.2308566	-6.6530085	2.8716E-11	9.5279E-11
LPL	ENSG00000175445	protein_coding	2278.427	-1.2337327	0.13874889	-8.8918382	6.0106E-19	3.3069E-18
DPP10	ENSG00000175497	protein_coding	35.0084814	-1.4320998	0.43687893	-3.2780244	0.00104536	0.00176133
P2RY2	ENSG00000175591	protein_coding	83.4834935	-1.4162003	0.13515878	-10.47805	1.0897E-25	8.6828E-25
TOM1L2	ENSG00000175662	protein_coding	4135.4469	-1.067858	0.06331433	-16.865975	8.0069E-64	3.0638E-62
KDF1	ENSG00000175707	protein_coding	296.159085	-2.0371676	0.16574219	-12.291183	1.0102E-34	1.256E-33
MSRA	ENSG00000175806	protein_coding	2336.63179	-1.2995441	0.12069226	-10.767418	4.9056E-27	4.1979E-26
ETV4	ENSG00000175832	protein_coding	105.121871	-1.3021142	0.22230127	-5.8574305	4.7008E-09	1.3127E-08
CALCB	ENSG00000175868	protein_coding	12.0953045	-1.3681673	0.21354079	-6.4070535	1.4836E-10	4.662E-10

HOXD8	ENSG00000175879	protein_coding	2307.00115	-1.7771414	0.09048475	-19.640231	7.0078E-86	6.0498E-84
ARL4D	ENSG00000175906	protein_coding	783.097008	-2.687385	0.18630558	-14.424608	3.6232E-47	7.6259E-46
DOK7	ENSG00000175920	protein_coding	84.2638934	-2.3663212	0.207349	-11.412263	3.6316E-30	3.6198E-29
LRRN1	ENSG00000175928	protein_coding	60.229631	-1.7544299	0.25090333	-6.9924538	2.7012E-12	9.6392E-12
PLEKHD1	ENSG00000175985	protein_coding	63.3289588	-2.4743407	0.23771748	-10.408745	2.2618E-25	1.7691E-24
ASCL3	ENSG00000176009	protein_coding	1.87157974	-1.4486654	0.24697143	-5.8657204	4.4719E-09	1.2513E-08
C11orf16	ENSG00000176029	protein_coding	12.0460849	-2.0392274	0.1669124	-12.217351	2.5113E-34	3.0593E-33
GPX2	ENSG00000176153	protein_coding	75.5683015	-1.4543986	0.23680627	-6.1417234	8.1631E-10	2.428E-09
ACBD7	ENSG00000176244	protein_coding	12.3263293	-1.6523763	0.13720981	-12.042698	2.1191E-33	2.4699E-32
PRR18	ENSG00000176381	protein_coding	8.1340583	-1.6788103	0.29045255	-5.7799813	7.4709E-09	2.0482E-08
HSD11B2	ENSG00000176387	protein_coding	7979.53855	-2.7792375	0.22124752	-12.561666	3.4303E-36	4.5619E-35
DIRAS1	ENSG00000176490	protein_coding	171.183786	-2.2071521	0.28545901	-7.7319408	1.0592E-14	4.4594E-14
PRR15	ENSG00000176532	protein_coding	289.317042	-3.9267427	0.19734073	-19.898288	4.2106E-88	3.9472E-86
GNG7	ENSG00000176533	protein_coding	971.721607	-1.4078687	0.09941963	-14.160873	1.6E-45	3.1451E-44
RNF152	ENSG00000176641	protein_coding	6790.06875	-1.1956293	0.15317824	-7.8054774	5.9277E-15	2.5386E-14
TCIM	ENSG00000176907	protein_coding	4001.13985	-2.1765326	0.1510098	-14.413188	4.2751E-47	8.9733E-46
FUT2	ENSG00000176920	protein_coding	64.3016082	-1.8721642	0.15886033	-11.78497	4.6659E-32	5.1047E-31
GCNT4	ENSG00000176928	protein_coding	449.376358	-1.6119253	0.15758636	-10.228838	1.4728E-24	1.1005E-23
SHMT1	ENSG00000176974	protein_coding	10161.1514	-1.0607017	0.1016547	-10.43436	1.7277E-25	1.3611E-24
B3GNT8	ENSG00000177191	protein_coding	185.576329	-1.2591719	0.15745837	-7.9968559	1.2764E-15	5.715E-15
C10orf71	ENSG00000177354	protein_coding	16.5544734	-1.4462622	0.59447927	-2.432822	0.01498166	0.02186901
UMODL1	ENSG00000177398	protein_coding	16.8095431	-2.1962383	0.3139619	-6.9952381	2.6481E-12	9.4585E-12
UBE2U	ENSG00000177414	protein_coding	4.03707173	-1.6518983	0.3178377	-5.1973015	2.022E-07	4.9246E-07
RPRM	ENSG00000177519	protein_coding	24.9395151	-3.2928942	0.34830591	-9.4540291	3.2603E-21	2.034E-20
SAMD12	ENSG00000177570	protein_coding	720.705408	-1.8332588	0.15512053	-11.818286	3.1402E-32	3.4671E-31

DNAJC28	ENSG00000177692	protein_coding	116.669888	-1.1544595	0.08879334	-13.001646	1.1974E-38	1.7627E-37
NAALADL2	ENSG00000177694	protein_coding	525.310171	-1.8801889	0.11094829	-16.946533	2.0414E-64	8.0189E-63
MYOZ1	ENSG00000177791	protein_coding	99.9409174	-1.4496969	0.13459526	-10.770787	4.7294E-27	4.0501E-26
KCNJ10	ENSG00000177807	protein_coding	1060.25566	-6.1859803	0.23137748	-26.735447	1.823E-157	1.577E-154
CAPZA3	ENSG00000177938	protein_coding	1.92938909	-2.5094175	0.49250636	-5.0951982	3.4838E-07	8.3133E-07
ALS2CL	ENSG00000178038	protein_coding	986.793894	-1.7156792	0.17062055	-10.055525	8.6857E-24	6.2135E-23
GRAMD1C	ENSG00000178075	protein_coding	1307.99847	-1.3947812	0.11607214	-12.016502	2.9103E-33	3.3715E-32
HTR3C	ENSG00000178084	protein_coding	1.29372846	-1.1941767	0.34796367	-3.4319005	0.00059937	0.00103646
PPP1R42	ENSG00000178125	protein_coding	12.3359508	-1.6734078	0.20663595	-8.0983383	5.5715E-16	2.5477E-15
SLITRK1	ENSG00000178235	protein_coding	2.92474113	-1.0889016	0.33515864	-3.2489141	0.00115846	0.00194233
KCNG2	ENSG00000178342	protein_coding	5.49525519	-1.5106011	0.15813218	-9.5527748	1.2627E-21	8.056E-21
SHISA3	ENSG00000178343	protein_coding	1034.57105	-2.1066615	0.24209842	-8.7016737	3.2702E-18	1.7207E-17
CALML3	ENSG00000178363	protein_coding	79.7630627	-5.6789816	0.32358855	-17.550008	5.9472E-69	2.7482E-67
HTR1A	ENSG00000178394	protein_coding	0.50927789	-1.5181624	0.41716736	-3.6392166	0.00027347	0.00049098
CCDC185	ENSG00000178395	protein_coding	3.28430615	-2.7863979	0.41460583	-6.7205952	1.8098E-11	6.0856E-11
GLDC	ENSG00000178445	protein_coding	1358.39243	-2.3596344	0.17753802	-13.290868	2.6153E-40	4.1354E-39
TUBAL3	ENSG00000178462	protein_coding	47.6075896	-3.8198945	0.40250648	-9.4902683	2.3044E-21	1.45E-20
CA8	ENSG00000178538	protein_coding	345.493987	-3.2546649	0.20409526	-15.946793	2.9991E-57	9.2579E-56
ERBB4	ENSG00000178568	protein_coding	691.363168	-3.861119	0.28740972	-13.434198	3.8118E-41	6.2294E-40
DEFB125	ENSG00000178591	protein_coding	0.84460498	-3.0695288	0.60594379	-5.065699	4.069E-07	9.6552E-07
CSRNP3	ENSG00000178662	protein_coding	379.356462	-1.6131679	0.11521048	-14.00192	1.5172E-44	2.8609E-43
STX19	ENSG00000178750	protein_coding	9.91580302	-1.6905721	0.18882226	-8.9532458	3.4518E-19	1.925E-18
ERFE	ENSG00000178752	protein_coding	98.3689936	-1.1404045	0.20958277	-5.4413085	5.2891E-08	1.3536E-07
CPN2	ENSG00000178772	protein_coding	614.585697	-1.1900027	0.40738584	-2.9210704	0.00348831	0.00554138
FOXE1	ENSG00000178919	protein_coding	15.3329232	-1.0868179	0.39203116	-2.7722744	0.00556661	0.00861887

KLHDC7A	ENSG00000179023	protein_coding	3269.38925	-1.0072537	0.13401551	-7.5159488	5.65E-14	2.2668E-13
ZFP42	ENSG00000179059	protein_coding	3.03357355	-1.4854972	0.37939559	-3.9154307	9.0243E-05	0.00017069
TCAIM	ENSG00000179152	protein_coding	1816.95651	-1.0648236	0.06085948	-17.496429	1.5254E-68	6.9577E-67
SMCO3	ENSG00000179256	protein_coding	97.5613519	-1.1760496	0.2241951	-5.2456528	1.5573E-07	3.8292E-07
PCARE	ENSG00000179270	protein_coding	13.4757557	-5.8134452	0.34955936	-16.630781	4.1712E-62	1.5056E-60
GATA2	ENSG00000179348	protein_coding	1003.24099	-1.5230913	0.1289287	-11.813438	3.3267E-32	3.6694E-31
EGR3	ENSG00000179388	protein_coding	928.187149	-1.1468704	0.197433	-5.8089092	6.2881E-09	1.7371E-08
GPC5	ENSG00000179399	protein_coding	115.415694	-5.8462631	0.21536209	-27.146203	2.807E-162	2.681E-159
LSMEM2	ENSG00000179564	protein_coding	5.9540648	-1.3129976	0.16246086	-8.0819318	6.3749E-16	2.9031E-15
TPPP2	ENSG00000179636	protein_coding	12.0009942	-4.1265235	0.21178212	-19.484759	1.4787E-84	1.2105E-82
PIPOX	ENSG00000179761	protein_coding	1555.19255	-2.9888691	0.20253947	-14.756971	2.7747E-49	6.3852E-48
FAM216B	ENSG00000179813	protein_coding	1.36070046	-2.1385981	0.42238081	-5.063199	4.1228E-07	9.7772E-07
AKAP5	ENSG00000179841	protein_coding	280.315994	-1.3277021	0.12960829	-10.24396	1.2597E-24	9.4543E-24
ABCA13	ENSG00000179869	protein_coding	48.7609571	-3.4736999	0.26737799	-12.99172	1.3633E-38	2.0043E-37
ITLN1	ENSG00000179914	protein_coding	40.2292251	-3.6595671	0.24110272	-15.178457	4.912E-52	1.2693E-50
NRXN1	ENSG00000179915	protein_coding	27.8636521	-2.0140921	0.28815023	-6.9897294	2.7542E-12	9.8199E-12
GPBAR1	ENSG00000179921	protein_coding	112.47298	-1.6915292	0.13272387	-12.744725	3.3357E-37	4.6491E-36
LYNX1	ENSG00000180155	protein_coding	831.602029	-1.1849525	0.13117669	-9.0332554	1.6664E-19	9.4623E-19
TH	ENSG00000180176	protein_coding	3.34460327	-2.0426195	0.35068687	-5.8246251	5.7241E-09	1.5859E-08
SLC9A4	ENSG00000180251	protein_coding	431.013813	-6.1086497	0.41953296	-14.560595	5.002E-48	1.0919E-46
PLD5	ENSG00000180287	protein_coding	34.2607505	-1.7328721	0.24019897	-7.2143192	5.4204E-13	2.0281E-12
PNPLA1	ENSG00000180316	protein_coding	75.68883	-2.4846293	0.19883563	-12.495896	7.8606E-36	1.0281E-34
ALX1	ENSG00000180318	protein_coding	29.6577886	-3.0819709	0.27348936	-11.26907	1.8653E-29	1.8062E-28
MEIOC	ENSG00000180336	protein_coding	38.6512681	-1.6789955	0.11447818	-14.666512	1.0564E-48	2.375E-47
MTURN	ENSG00000180354	protein_coding	3712.62392	-3.0530952	0.1158558	-26.352546	4.799E-153	3.928E-150

CYP8B1	ENSG00000180432	protein_coding	661.126873	-1.4760698	0.23769809	-6.2098515	5.3035E-10	1.6012E-09
SERTM1	ENSG00000180440	protein_coding	9.28491833	-1.2444255	0.32669789	-3.8091016	0.00013947	0.00025855
GAS1	ENSG00000180447	protein_coding	347.697432	-2.1022985	0.16919124	-12.425576	1.8985E-35	2.4481E-34
KCNE1	ENSG00000180509	protein_coding	28.0007979	-1.8231221	0.1751085	-10.411385	2.2E-25	1.7227E-24
RNF182	ENSG00000180537	protein_coding	150.404155	-1.2707078	0.20101613	-6.321422	2.5917E-10	7.9982E-10
TSPYL5	ENSG00000180543	protein_coding	1313.55283	-1.6164891	0.11776682	-13.726184	7.0764E-43	1.251E-41
SKIDA1	ENSG00000180592	protein_coding	119.937823	-1.2728303	0.14333923	-8.879846	6.6953E-19	3.6748E-18
SLC47A2	ENSG00000180638	protein_coding	563.116117	-3.9368432	0.22417459	-17.561505	4.857E-69	2.2513E-67
SHISA2	ENSG00000180730	protein_coding	172.899105	-2.6414194	0.21073444	-12.53435	4.8431E-36	6.3981E-35
AGTR2	ENSG00000180772	protein_coding	7.43161741	-2.6304632	0.4988334	-5.27323	1.3404E-07	3.3127E-07
MAP3K15	ENSG00000180815	protein_coding	113.157004	-2.8573892	0.22952005	-12.44941	1.4088E-35	1.8249E-34
GREM2	ENSG00000180875	protein_coding	52.4092195	-1.9716695	0.31065384	-6.3468376	2.1979E-10	6.8122E-10
GPR62	ENSG00000180929	protein_coding	13.287408	-1.5860224	0.18150026	-8.7384028	2.3643E-18	1.2543E-17
HIGD1A	ENSG00000181061	protein_coding	2556.58045	-1.3324083	0.12989085	-10.257907	1.0904E-24	8.2053E-24
TMEM132C	ENSG00000181234	protein_coding	31.7642749	-2.0383791	0.28028978	-7.2723991	3.5316E-13	1.3387E-12
SYNE4	ENSG00000181392	protein_coding	111.493726	-2.1899691	0.21944574	-9.9795469	1.8732E-23	1.3144E-22
UTS2R	ENSG00000181408	protein_coding	6.93164654	-4.0886708	0.3900251	-10.483097	1.033E-25	8.2387E-25
DDN	ENSG00000181418	protein_coding	250.052901	-6.3697633	0.24364211	-26.143934	1.155E-150	8.681E-148
EDDM3A	ENSG00000181562	protein_coding	6.75536942	-4.9282837	0.27502859	-17.919169	8.3567E-72	4.285E-70
TNFSF15	ENSG00000181634	protein_coding	394.455097	-1.2535588	0.12551558	-9.9872762	1.7328E-23	1.2183E-22
PLAG1	ENSG00000181690	protein_coding	142.937158	-1.6154437	0.18576692	-8.6960785	3.4355E-18	1.8056E-17
CLDN7	ENSG00000181885	protein_coding	2555.81388	-1.1565444	0.17071648	-6.7746502	1.2471E-11	4.2425E-11
PNMA8A	ENSG00000182013	protein_coding	1016.47345	-1.8678003	0.1709027	-10.929027	8.3741E-28	7.4467E-27
ADIG	ENSG00000182035	protein_coding	1.5118407	-1.6187739	0.33175107	-4.8794835	1.0636E-06	2.4266E-06
MGAT4C	ENSG00000182050	protein_coding	16.9086318	-2.0007463	0.25105103	-7.9694806	1.5934E-15	7.0944E-15

IDH2	ENSG00000182054	protein_coding	11648.0095	-1.0892262	0.09513689	-11.449042	2.3776E-30	2.3946E-29
FAM181B	ENSG00000182103	protein_coding	31.8568121	-2.2332505	0.18038322	-12.380589	3.3289E-35	4.2213E-34
TMEM30B	ENSG00000182107	protein_coding	1054.73221	-2.8217386	0.23466607	-12.024485	2.6423E-33	3.0696E-32
KCNIP1	ENSG00000182132	protein_coding	95.863953	-1.3505663	0.28621075	-4.7187825	2.3726E-06	5.2378E-06
KCNA4	ENSG00000182255	protein_coding	7.58324785	-2.4922274	0.34553564	-7.2126493	5.4874E-13	2.0524E-12
GABRG3	ENSG00000182256	protein_coding	11.4294039	-2.6746991	0.27801698	-9.6206323	6.5428E-22	4.2429E-21
NLRP10	ENSG00000182261	protein_coding	1.18684671	-1.7283187	0.34987863	-4.9397665	7.8216E-07	1.8085E-06
TMIGD1	ENSG00000182271	protein_coding	34.331398	-1.3930478	0.20273344	-6.8713271	6.3607E-12	2.2109E-11
GLTPD2	ENSG00000182327	protein_coding	224.097997	-1.1173674	0.17014472	-6.5671588	5.1284E-11	1.6691E-10
ZNF804B	ENSG00000182348	protein_coding	21.876423	-2.864943	0.40943262	-6.997349	2.6085E-12	9.3244E-12
CACNB4	ENSG00000182389	protein_coding	156.173274	-1.6056275	0.18847206	-8.5191804	1.6069E-17	8.1017E-17
GLRX5	ENSG00000182512	protein_coding	1943.38364	-1.0912144	0.08179671	-13.340567	1.3443E-40	2.1495E-39
CAV3	ENSG00000182533	protein_coding	1.87693986	-1.9719703	0.28664692	-6.8794402	6.0088E-12	2.0915E-11
EPHB3	ENSG00000182580	protein_coding	392.088712	-1.1054934	0.14436686	-7.6575292	1.8954E-14	7.837E-14
EPGN	ENSG00000182585	protein_coding	6.73652237	-4.0283873	0.22885668	-17.602227	2.3682E-69	1.1089E-67
KCNB2	ENSG00000182674	protein_coding	2.73970497	-2.5395093	0.32353557	-7.849243	4.1856E-15	1.8094E-14
RGS6	ENSG00000182732	protein_coding	54.3446911	-2.3114974	0.16777162	-13.777642	3.4744E-43	6.2068E-42
PAQR7	ENSG00000182749	protein_coding	1904.04901	-1.6133284	0.07639341	-21.118687	5.356E-99	7.6491E-97
PAPPA	ENSG00000182752	protein_coding	667.652841	-3.1396507	0.17907529	-17.532574	8.0827E-69	3.7163E-67
MAFA	ENSG00000182759	protein_coding	10.2313111	-1.8559916	0.29100544	-6.3778589	1.7958E-10	5.607E-10
C1orf116	ENSG00000182795	protein_coding	495.408359	-2.8690766	0.26658369	-10.762386	5.1811E-27	4.4278E-26
PLCXD3	ENSG00000182836	protein_coding	455.89549	-1.84512	0.22018272	-8.3799492	5.2951E-17	2.5861E-16
C11orf54	ENSG00000182919	protein_coding	11356.4623	-1.0951187	0.12056431	-9.0832744	1.0536E-19	6.0446E-19
CADM1	ENSG00000182985	protein_coding	3000.32888	-1.3565805	0.12171451	-11.145594	7.524E-29	7.0649E-28
SLC8A1	ENSG00000183023	protein_coding	2550.33189	-1.3681822	0.12858917	-10.639949	1.9424E-26	1.6119E-25

PCP4	ENSG00000183036	protein_coding	334.638778	-2.3593108	0.41034536	-5.7495735	8.9469E-09	2.4394E-08
ABAT	ENSG00000183044	protein_coding	2705.74196	-3.0671661	0.14775562	-20.758372	1.03E-95	1.3152E-93
SLC25A10	ENSG00000183048	protein_coding	823.480735	-1.0488048	0.13356228	-7.8525528	4.0765E-15	1.7636E-14
IGSF5	ENSG00000183067	protein_coding	13.3274708	-2.251036	0.22536971	-9.9881921	1.7168E-23	1.2075E-22
AFMID	ENSG00000183077	protein_coding	2020.09505	-1.0917405	0.08914895	-12.246252	1.7594E-34	2.1582E-33
FREM3	ENSG00000183090	protein_coding	11.2766493	-1.5016929	0.21394224	-7.0191512	2.2322E-12	8.0086E-12
FAM43B	ENSG00000183114	protein_coding	69.5655249	-1.5991219	0.18277963	-8.7489065	2.1543E-18	1.1458E-17
CSMD1	ENSG00000183117	protein_coding	149.630326	-1.3293926	0.21636204	-6.1442967	8.0319E-10	2.3905E-09
RIPPLY3	ENSG00000183145	protein_coding	18.1522685	-1.339994	0.18748379	-7.1472528	8.8532E-13	3.2705E-12
CHST6	ENSG00000183196	protein_coding	45.2954969	-2.6688817	0.24025194	-11.108679	1.1383E-28	1.0572E-27
PRR36	ENSG00000183248	protein_coding	132.030595	-1.3965942	0.22195868	-6.292136	3.1313E-10	9.6129E-10
CCBE1	ENSG00000183287	protein_coding	260.895235	-1.7165525	0.3007942	-5.7067342	1.1516E-08	3.1109E-08
OVCH2	ENSG00000183378	protein_coding	77.9478926	-4.4193822	0.30616658	-14.434567	3.1361E-47	6.6158E-46
LRIT3	ENSG00000183423	protein_coding	44.510616	-2.0926973	0.16992418	-12.315477	7.4772E-35	9.337E-34
URAD	ENSG00000183463	protein_coding	0.54309184	-1.1525793	0.35487189	-3.2478743	0.00116271	0.00194901
IZUMO1R	ENSG00000183560	protein_coding	2.64667628	-1.0815473	0.19017126	-5.6872278	1.2912E-08	3.4746E-08
PGPEP1L	ENSG00000183571	protein_coding	10.7769884	-1.1163424	0.27709299	-4.0287645	5.6071E-05	0.00010833
ZNRF3	ENSG00000183579	protein_coding	24.6845307	-1.3009847	0.13348659	-9.7461827	1.9154E-22	1.2725E-21
TAFA1	ENSG00000183662	protein_coding	10.9148558	-1.3614174	0.1288531	-10.565655	4.2996E-26	3.4959E-25
CMTM4	ENSG00000183723	protein_coding	4721.02388	-1.8695445	0.12542962	-14.905127	3.0523E-50	7.3028E-49
KCTD8	ENSG00000183783	protein_coding	14.0804821	-4.079302	0.25633113	-15.914189	5.0518E-57	1.5367E-55
EMILIN3	ENSG00000183798	protein_coding	17.4338704	-1.2473512	0.15369519	-8.1157459	4.8281E-16	2.2168E-15
ANKRD45	ENSG00000183831	protein_coding	94.5200096	-1.2912911	0.16090625	-8.0251148	1.0143E-15	4.5666E-15
FAM3B	ENSG00000183844	protein_coding	254.506146	-3.8745542	0.26440141	-14.65406	1.269E-48	2.8418E-47
SRARP	ENSG00000183888	protein_coding	26.8604471	-2.9518909	0.36566132	-8.0727458	6.8735E-16	3.1252E-15

LRRC55	ENSG00000183908	protein_coding	52.5199997	-1.2577988	0.16132653	-7.7966023	6.3596E-15	2.7162E-14
PRKX	ENSG00000183943	protein_coding	2363.99426	-1.00632	0.09406811	-10.697781	1.0425E-26	8.7666E-26
ST6GALNAC3	ENSG00000184005	protein_coding	765.201533	-1.2239374	0.12223231	-10.013207	1.3336E-23	9.4398E-23
TMPRSS2	ENSG00000184012	protein_coding	1474.20088	-3.4843064	0.34674426	-10.048635	9.3149E-24	6.6491E-23
ADRA2C	ENSG00000184160	protein_coding	455.868029	-1.4393223	0.16445988	-8.7518145	2.0995E-18	1.117E-17
KCNJ12	ENSG00000184185	protein_coding	186.22632	-2.3330499	0.1487475	-15.684633	1.9267E-55	5.5619E-54
PCDH9	ENSG00000184226	protein_coding	321.530759	-3.2604207	0.18955183	-17.200682	2.6242E-66	1.0996E-64
ALDH1A3	ENSG00000184254	protein_coding	1022.09609	-1.3987567	0.18246887	-7.6657278	1.7782E-14	7.3635E-14
TACSTD2	ENSG00000184292	protein_coding	4646.35693	-3.4790618	0.25480733	-13.653696	1.9189E-42	3.317E-41
PRKD1	ENSG00000184304	protein_coding	1381.53063	-1.0750808	0.08266327	-13.005544	1.1379E-38	1.6772E-37
CCSER1	ENSG00000184305	protein_coding	195.163416	-2.4846755	0.15626929	-15.89996	6.3405E-57	1.9211E-55
GDF3	ENSG00000184344	protein_coding	47.6513605	-2.1493739	0.16558197	-12.980724	1.5739E-38	2.3094E-37
ACTRT3	ENSG00000184378	protein_coding	98.9807394	-1.7752282	0.11473155	-15.472886	5.2882E-54	1.4533E-52
LRRC19	ENSG00000184434	protein_coding	1271.29421	-2.0135578	0.19386783	-10.38624	2.8642E-25	2.2265E-24
NCMAP	ENSG00000184454	protein_coding	100.613363	-1.995881	0.23124302	-8.6310972	6.0766E-18	3.1451E-17
KCNH7	ENSG00000184611	protein_coding	6.809466	-1.239542	0.16912902	-7.3289728	2.3192E-13	8.9057E-13
RALYL	ENSG00000184672	protein_coding	133.611025	-5.7437468	0.41314501	-13.902496	6.117E-44	1.128E-42
H2BC21	ENSG00000184678	protein_coding	953.415136	-1.199952	0.12760999	-9.4032761	5.289E-21	3.2562E-20
CLDN6	ENSG00000184697	protein_coding	7.86425415	-1.7230103	0.20252856	-8.5074926	1.7774E-17	8.9416E-17
SATL1	ENSG00000184788	protein_coding	1.97258341	-1.9339257	0.22136966	-8.7361825	2.4112E-18	1.2781E-17
APOO	ENSG00000184831	protein_coding	755.676796	-1.1685711	0.10721527	-10.899297	1.1615E-27	1.0259E-26
DRD1	ENSG00000184845	protein_coding	40.8668454	-1.0346616	0.1988762	-5.2025409	1.9658E-07	4.7944E-07
SDR42E1	ENSG00000184860	protein_coding	464.366897	-1.2761598	0.15242543	-8.3723547	5.6478E-17	2.7531E-16
SRY	ENSG00000184895	protein_coding	0.39895365	-1.6238684	0.42168249	-3.8509267	0.00011767	0.00021992
TCEAL2	ENSG00000184905	protein_coding	142.741027	-3.1345311	0.33173811	-9.4488121	3.427E-21	2.1345E-20

CLCNKB	ENSG00000184908	protein_coding	3852.23261	-3.1543177	0.39095297	-8.0682791	7.1296E-16	3.2391E-15
DMRTC1B	ENSG00000184911	protein_coding	0.54025912	-1.7896657	0.25854806	-6.9219846	4.4536E-12	1.5639E-11
WT1	ENSG00000184937	protein_coding	381.426392	-2.5077002	0.28017253	-8.9505569	3.5369E-19	1.9703E-18
MUC6	ENSG00000184956	protein_coding	79.3853601	-2.6644675	0.2263745	-11.770176	5.5609E-32	6.0665E-31
SORCS2	ENSG00000184985	protein_coding	1220.11648	-1.314815	0.23461222	-5.604205	2.0921E-08	5.5321E-08
ROBO2	ENSG00000185008	protein_coding	220.791185	-1.046942	0.17663526	-5.9271404	3.0826E-09	8.7394E-09
SGCZ	ENSG00000185053	protein_coding	5.0043331	-4.4624647	0.4450212	-10.027533	1.1536E-23	8.1939E-23
C5orf47	ENSG00000185056	protein_coding	7.50762047	-1.8397979	0.1833075	-10.036675	1.0516E-23	7.4842E-23
FLRT2	ENSG00000185070	protein_coding	407.427953	-1.1463653	0.16829416	-6.8116763	9.6468E-12	3.3058E-11
INPP5J	ENSG00000185133	protein_coding	1367.54875	-1.7148037	0.32559785	-5.266631	1.3895E-07	3.4297E-07
NPY2R	ENSG00000185149	protein_coding	1.03663773	-3.7254071	0.37381111	-9.9660147	2.1467E-23	1.5029E-22
MFSD6L	ENSG00000185156	protein_coding	65.2589288	-3.3898524	0.27058604	-12.527817	5.2591E-36	6.9376E-35
MC2R	ENSG00000185231	protein_coding	0.60648139	-2.9757295	0.59089032	-5.0360098	4.7534E-07	1.121E-06
PPIL6	ENSG00000185250	protein_coding	274.549275	-1.0342939	0.08661512	-11.941262	7.212E-33	8.1996E-32
NOTUM	ENSG00000185269	protein_coding	16.019272	-2.2069018	0.28715359	-7.6854404	1.5247E-14	6.3408E-14
KLHL33	ENSG00000185271	protein_coding	10.6118488	-1.532298	0.1555707	-9.8495279	6.8867E-23	4.6908E-22
RBM11	ENSG00000185272	protein_coding	38.4949663	-2.5089277	0.24863095	-10.090971	6.0567E-24	4.3635E-23
NUPR2	ENSG00000185290	protein_coding	60.9261244	-2.9317446	0.45224053	-6.482711	9.0089E-11	2.8795E-10
HS6ST3	ENSG00000185352	protein_coding	140.226556	-1.4221294	0.29056427	-4.8943712	9.8621E-07	2.2568E-06
SH3BGR	ENSG00000185437	protein_coding	276.030548	-1.1620524	0.08092968	-14.358793	9.3848E-47	1.944E-45
MUC1	ENSG00000185499	protein_coding	7196.5414	-1.5069772	0.1802817	-8.359014	6.3245E-17	3.0726E-16
BRCC3	ENSG00000185515	protein_coding	922.733108	-1.0734555	0.0693832	-15.471404	5.4114E-54	1.4863E-52
PDE6G	ENSG00000185527	protein_coding	88.1720907	-1.0698879	0.14566672	-7.3447654	2.0612E-13	7.9475E-13
DLK1	ENSG00000185559	protein_coding	19.9139195	-3.0251514	0.38679722	-7.8210267	5.2394E-15	2.2525E-14
LSAMP	ENSG00000185565	protein_coding	236.559148	-2.1021891	0.33800805	-6.2193463	4.9923E-10	1.5107E-09

DBX2	ENSG00000185610	protein_coding	1.87873657	-2.5543559	0.25151023	-10.156072	3.1137E-24	2.2808E-23
PBX1	ENSG00000185630	protein_coding	3910.96293	-1.4195054	0.12222022	-11.614325	3.4853E-31	3.6536E-30
SYN3	ENSG00000185666	protein_coding	45.7969459	-1.110741	0.12559355	-8.8439337	9.2406E-19	5.0273E-18
MORN5	ENSG00000185681	protein_coding	22.7247726	-1.0266904	0.19710663	-5.2088071	1.9006E-07	4.6427E-07
SRL	ENSG00000185739	protein_coding	167.259057	-1.8979668	0.1204543	-15.756738	6.1739E-56	1.8096E-54
PCYT2	ENSG00000185813	protein_coding	1798.41631	-1.3070374	0.11077919	-11.798583	3.9694E-32	4.3532E-31
NAT8L	ENSG00000185818	protein_coding	573.401152	-4.5772284	0.27802259	-16.463513	6.7092E-61	2.3353E-59
NPAP1	ENSG00000185823	protein_coding	10.2300817	-2.6121043	0.28180239	-9.2692768	1.8741E-20	1.1211E-19
NKAIN3	ENSG00000185942	protein_coding	2.50079055	-1.4471028	0.43166316	-3.3523891	0.00080117	0.00136642
GRK1	ENSG00000185974	protein_coding	3.01013745	-1.4801937	0.22871785	-6.4717015	9.6905E-11	3.0898E-10
ATP4B	ENSG00000186009	protein_coding	10.2042587	-2.7569232	0.27124242	-10.164056	2.8689E-24	2.1069E-23
HTR3D	ENSG00000186090	protein_coding	1.0803902	-2.6808862	0.32970416	-8.131187	4.2511E-16	1.9585E-15
AGBL4	ENSG00000186094	protein_coding	59.3583724	-2.4781614	0.12614926	-19.644676	6.4205E-86	5.5638E-84
CYP4F2	ENSG00000186115	protein_coding	363.502427	-4.9255293	0.35717457	-13.790258	2.9172E-43	5.2318E-42
SLC51B	ENSG00000186198	protein_coding	196.95456	-1.5456344	0.1751708	-8.8235847	1.1085E-18	5.9995E-18
SOWAHB	ENSG00000186212	protein_coding	609.363328	-1.3239525	0.14854165	-8.913005	4.9668E-19	2.7435E-18
NAP1L3	ENSG00000186310	protein_coding	197.235806	-1.3122481	0.14720134	-8.9146473	4.8937E-19	2.7054E-18
RGS9BP	ENSG00000186326	protein_coding	14.0877605	-1.1345077	0.17693302	-6.4120741	1.4355E-10	4.5159E-10
SLC36A2	ENSG00000186335	protein_coding	688.385837	-4.6917538	0.36669049	-12.794861	1.7517E-37	2.4715E-36
TRDN	ENSG00000186439	protein_coding	10.3645676	-1.9161888	0.31646613	-6.0549571	1.4046E-09	4.0968E-09
TMPRSS12	ENSG00000186452	protein_coding	3.56848102	-1.9913362	0.26151162	-7.6147142	2.6428E-14	1.082E-13
DEFB132	ENSG00000186458	protein_coding	4.70558818	-3.7653803	0.66835156	-5.6338319	1.7625E-08	4.693E-08
NAP1L2	ENSG00000186462	protein_coding	351.918934	-2.2408251	0.189685	-11.813402	3.3282E-32	3.6701E-31
C5orf38	ENSG00000186493	protein_coding	90.0730659	-2.5635396	0.26008697	-9.8564706	6.4269E-23	4.3809E-22
CLCNKA	ENSG00000186510	protein_coding	2070.37279	-4.9303223	0.33485216	-14.723878	4.5292E-49	1.0324E-47

CYP4F3	ENSG00000186529	protein_coding	647.479727	-2.1745359	0.28178229	-7.7170778	1.1903E-14	4.9902E-14
HPDL	ENSG00000186603	protein_coding	20.1308939	-1.0045544	0.13002444	-7.7258888	1.1108E-14	4.6692E-14
MAGEE2	ENSG00000186675	protein_coding	5.22961729	-2.7390926	0.19544035	-14.01498	1.2623E-44	2.3912E-43
CFAP73	ENSG00000186710	protein_coding	20.4833685	-1.4578449	0.15220737	-9.5780176	9.8925E-22	6.3522E-21
FOXE3	ENSG00000186790	protein_coding	2.36031568	-2.105352	0.21100384	-9.9777902	1.9067E-23	1.3373E-22
KRTAP17-1	ENSG00000186860	protein_coding	1.16270897	-2.1922273	0.31380975	-6.9858483	2.8314E-12	1.009E-11
PPARA	ENSG00000186951	protein_coding	3654.75288	-1.0664247	0.07669817	-13.904174	5.9753E-44	1.1032E-42
CYP4A11	ENSG00000187048	protein_coding	5798.68362	-1.6739943	0.3024391	-5.5349796	3.1126E-08	8.1119E-08
CCK	ENSG00000187094	protein_coding	1.57327514	-2.7184624	0.2930072	-9.2778006	1.7301E-20	1.0377E-19
LYPD6	ENSG00000187123	protein_coding	88.9675804	-2.654599	0.23305251	-11.390562	4.6598E-30	4.6216E-29
AKR1C1	ENSG00000187134	protein_coding	2295.55053	-1.0433275	0.18384524	-5.6750312	1.3866E-08	3.7233E-08
FOXD3	ENSG00000187140	protein_coding	1.43815887	-2.2291537	0.39231013	-5.6821211	1.3303E-08	3.5774E-08
TSPYL4	ENSG00000187189	protein_coding	2123.11044	-1.0661039	0.06714008	-15.8788	8.8862E-57	2.6817E-55
LCE2D	ENSG00000187223	protein_coding	0.37783789	-3.5512945	0.62851269	-5.6503146	1.6015E-08	4.2793E-08
BCAM	ENSG00000187244	protein_coding	17145.7138	-1.6860502	0.1090171	-15.465924	5.8921E-54	1.6174E-52
MAGI2	ENSG00000187391	protein_coding	700.325405	-1.6081911	0.09130865	-17.612691	1.9686E-69	9.2461E-68
LHFPL3	ENSG00000187416	protein_coding	36.3854386	-1.4469421	0.19379927	-7.46619	8.255E-14	3.2744E-13
PLEKHG7	ENSG00000187510	protein_coding	20.140485	-1.4626693	0.15680948	-9.3276841	1.0821E-20	6.5588E-20
ATP13A5	ENSG00000187527	protein_coding	10.5764244	-1.4675252	0.2600643	-5.6429321	1.6718E-08	4.4616E-08
TLR5	ENSG00000187554	protein_coding	577.560666	-1.1832024	0.10063137	-11.757789	6.4399E-32	6.9972E-31
MYMK	ENSG00000187616	protein_coding	5.73576197	-3.2333568	0.28044583	-11.529345	9.3855E-31	9.6057E-30
C5orf52	ENSG00000187658	protein_coding	0.49448144	-1.2800155	0.27988345	-4.5733875	4.799E-06	1.0304E-05
ERC2	ENSG00000187672	protein_coding	36.6952694	-1.6346659	0.19710959	-8.2931829	1.1026E-16	5.2809E-16
THSD4	ENSG00000187720	protein_coding	1665.13657	-1.9320129	0.15194984	-12.714807	4.8932E-37	6.7667E-36
ADH1A	ENSG00000187758	protein_coding	4.12112061	-1.7680131	0.22285425	-7.9334951	2.1306E-15	9.4004E-15

TMEM72	ENSG00000187783	protein_coding	2572.60481	-3.0253744	0.20302347	-14.9016	3.2178E-50	7.6867E-49
HELT	ENSG00000187821	protein_coding	4.70327779	-5.5370022	0.34608318	-15.99905	1.2974E-57	4.0513E-56
RTL4	ENSG00000187823	protein_coding	16.481771	-2.4801163	0.22773922	-10.890159	1.2842E-27	1.1312E-26
P2RX2	ENSG00000187848	protein_coding	3.36668805	-3.5825501	0.25858423	-13.85448	1.1952E-43	2.1837E-42
PALM3	ENSG00000187867	protein_coding	817.254975	-1.853272	0.16918146	-10.954345	6.3335E-28	5.6662E-27
FYB2	ENSG00000187889	protein_coding	211.272165	-2.0843251	0.26011263	-8.0131637	1.1179E-15	5.0189E-15
COL14A1	ENSG00000187955	protein_coding	3929.55616	-1.2222634	0.17177645	-7.1154307	1.1156E-12	4.093E-12
DNER	ENSG00000187957	protein_coding	597.773596	-2.513503	0.31018581	-8.1032174	5.3524E-16	2.4499E-15
CLCN1	ENSG00000188037	protein_coding	11.1286025	-1.8273962	0.16298985	-11.211718	3.5719E-29	3.4155E-28
WNT7B	ENSG00000188064	protein_coding	198.689538	-3.8363704	0.32453409	-11.821163	3.0345E-32	3.3544E-31
SCGB1C1	ENSG00000188076	protein_coding	0.72793619	-1.7942151	0.37942976	-4.7287146	2.2595E-06	5.0005E-06
C6orf132	ENSG00000188112	protein_coding	518.009888	-1.4498026	0.1526426	-9.4980213	2.1392E-21	1.3487E-20
COL4A5	ENSG00000188153	protein_coding	924.691774	-2.3153828	0.15955218	-14.511759	1.0207E-47	2.1946E-46
TMPPE	ENSG00000188167	protein_coding	128.268905	-1.0028691	0.12062421	-8.3139951	9.2533E-17	4.4501E-16
HEPACAM2	ENSG00000188175	protein_coding	823.590907	-1.166621	0.51221019	-2.2776216	0.02274913	0.03237738
SMTNL2	ENSG00000188176	protein_coding	1140.3486	-1.2302302	0.15736626	-7.8176235	5.383E-15	2.3116E-14
HYKK	ENSG00000188266	protein_coding	208.587217	-2.0223661	0.08035391	-25.168235	8.926E-140	4.353E-137
SBK1	ENSG00000188322	protein_coding	182.00888	-1.4543861	0.13145486	-11.063768	1.8803E-28	1.7246E-27
SLC38A3	ENSG00000188338	protein_coding	81.3472161	-2.9480437	0.29046407	-10.149426	3.3332E-24	2.4377E-23
INSC	ENSG00000188487	protein_coding	15.1738855	-1.8060931	0.18576957	-9.7222228	2.4243E-22	1.6041E-21
SERPINA5	ENSG00000188488	protein_coding	3070.931	-4.8851226	0.27857802	-17.535922	7.6203E-69	3.5073E-67
NCCRP1	ENSG00000188505	protein_coding	47.4308575	-2.956962	0.20235843	-14.612498	2.3379E-48	5.1798E-47
CFAP77	ENSG00000188523	protein_coding	4.40064676	-1.7003093	0.18625464	-9.1289501	6.9167E-20	4.0171E-19
NKAIN2	ENSG00000188580	protein_coding	13.2685723	-1.9539697	0.22888304	-8.5369791	1.3778E-17	6.9773E-17
NANOS1	ENSG00000188613	protein_coding	133.051595	-1.6042026	0.12383239	-12.954628	2.212E-38	3.2201E-37

HMX3	ENSG00000188620	protein_coding	0.85634669	-2.5323676	0.39167275	-6.4655191	1.0095E-10	3.2159E-10
SAXO2	ENSG00000188659	protein_coding	54.8091533	-1.0381167	0.11850449	-8.7601469	1.95E-18	1.0407E-17
BCL2L15	ENSG00000188761	protein_coding	86.1521192	-1.9148849	0.19326751	-9.9079505	3.8445E-23	2.6534E-22
PRELP	ENSG00000188783	protein_coding	3068.85517	-1.0030933	0.20495348	-4.8942487	9.8682E-07	2.2581E-06
SHISA6	ENSG00000188803	protein_coding	159.939543	-1.6766984	0.20045215	-8.3645819	6.0328E-17	2.9345E-16
HMX2	ENSG00000188816	protein_coding	9.58876226	-5.4378559	0.59864996	-9.0835317	1.0511E-19	6.0311E-19
SNTN	ENSG00000188817	protein_coding	0.76916675	-1.920302	0.44373863	-4.327552	1.5078E-05	3.0845E-05
ENTPD8	ENSG00000188833	protein_coding	91.0299872	-1.7294475	0.18555814	-9.3202456	1.1607E-20	7.026E-20
KLRG2	ENSG00000188883	protein_coding	101.682293	-5.0658912	0.30459885	-16.631354	4.1315E-62	1.4925E-60
GJB3	ENSG00000188910	protein_coding	38.2002502	-1.0394835	0.28854738	-3.6024709	0.00031521	0.00056182
C9orf152	ENSG00000188959	protein_coding	12.1431347	-1.7903884	0.21268503	-8.4180275	3.8288E-17	1.8866E-16
NDUFA4	ENSG00000189043	protein_coding	7657.23727	-1.0117454	0.10652679	-9.4975681	2.1485E-21	1.3544E-20
RELN	ENSG00000189056	protein_coding	430.579011	-1.0949359	0.25856181	-4.2347162	2.2884E-05	4.6024E-05
APOD	ENSG00000189058	protein_coding	608.781128	-1.446765	0.1939772	-7.458428	8.7561E-14	3.4659E-13
H1-0	ENSG00000189060	protein_coding	8612.20238	-1.9029603	0.10928897	-17.412191	6.6683E-68	2.9678E-66
ANKRD34B	ENSG00000189127	protein_coding	19.6098171	-3.9297781	0.2800471	-14.032561	9.8528E-45	1.871E-43
NKAPL	ENSG00000189134	protein_coding	48.9580727	-1.0842032	0.11352558	-9.5502988	1.2932E-21	8.2429E-21
CLDN4	ENSG00000189143	protein_coding	6828.31206	-1.1968122	0.13670697	-8.7545804	2.0486E-18	1.0911E-17
NUGGC	ENSG00000189233	protein_coding	231.794589	-1.3368001	0.20950726	-6.3806862	1.763E-10	5.5092E-10
TSPYL1	ENSG00000189241	protein_coding	7981.10319	-1.021979	0.07681443	-13.304519	2.1789E-40	3.4562E-39
FAM180A	ENSG00000189320	protein_coding	84.8234654	-2.3251165	0.21860251	-10.636275	2.0205E-26	1.6736E-25
S100A14	ENSG00000189334	protein_coding	307.164881	-2.0814193	0.18686751	-11.138476	8.1502E-29	7.645E-28
KAZN	ENSG00000189337	protein_coding	1904.9294	-1.0987138	0.08120934	-13.529403	1.0487E-41	1.752E-40
ALG1L	ENSG00000189366	protein_coding	21.55608	-1.0189337	0.18243967	-5.5850445	2.3364E-08	6.1511E-08
RASSF10	ENSG00000189431	protein_coding	115.558228	-3.1028722	0.21331166	-14.546191	6.1747E-48	1.3421E-46

PTPRT	ENSG00000196090	protein_coding	28.0889587	-1.5752921	0.24943305	-6.3154907	2.6931E-10	8.301E-10
SPOCK3	ENSG00000196104	protein_coding	8.67933643	-1.8740834	0.28515772	-6.5720943	4.9612E-11	1.6171E-10
ZNF676	ENSG00000196109	protein_coding	68.5198288	-1.1407216	0.28457714	-4.0084793	6.1111E-05	0.00011757
VN1R2	ENSG00000196131	protein_coding	0.5008242	-1.3046341	0.26502743	-4.9226381	8.5385E-07	1.9673E-06
ACADSB	ENSG00000196177	protein_coding	3967.64123	-2.0274276	0.10234591	-19.809562	2.4622E-87	2.235E-85
SEMA4A	ENSG00000196189	protein_coding	1431.4674	-1.5700236	0.12577062	-12.483229	9.2171E-36	1.2021E-34
SRGAP3	ENSG00000196220	protein_coding	487.975104	-2.4579039	0.11613303	-21.164554	2.027E-99	2.9124E-97
OR2C3	ENSG00000196242	protein_coding	1.33504107	-1.7347177	0.28168958	-6.1582602	7.3548E-10	2.1966E-09
SFTA2	ENSG00000196260	protein_coding	31.3689817	-2.4544472	0.3259324	-7.5305406	5.0531E-14	2.0333E-13
NLGN3	ENSG00000196338	protein_coding	70.1639979	-1.2862808	0.11648887	-11.042091	2.394E-28	2.1865E-27
ZNF729	ENSG00000196350	protein_coding	2.3382863	-1.4966581	0.30973375	-4.8320796	1.3511E-06	3.0532E-06
CPNE4	ENSG00000196353	protein_coding	30.8003454	-2.6211039	0.27512216	-9.527055	1.6181E-21	1.0264E-20
S100A5	ENSG00000196420	protein_coding	16.4392691	-1.7533173	0.18504466	-9.4751036	2.665E-21	1.6714E-20
ESRRG	ENSG00000196482	protein_coding	1417.0584	-3.0698674	0.20046377	-15.313826	6.1818E-53	1.6409E-51
SPTSSB	ENSG00000196542	protein_coding	34.9409106	-3.496117	0.23692503	-14.756216	2.8059E-49	6.4538E-48
MME	ENSG00000196549	protein_coding	9813.98951	-1.7848158	0.18195807	-9.80894	1.0305E-22	6.962E-22
LAMA2	ENSG00000196569	protein_coding	727.613405	-1.5260406	0.17709575	-8.6170372	6.8709E-18	3.5433E-17
PFN3	ENSG00000196570	protein_coding	1.60944501	-2.3182303	0.47004763	-4.931905	8.1432E-07	1.8797E-06
AJAP1	ENSG00000196581	protein_coding	734.498989	-1.188137	0.16993788	-6.991596	2.7178E-12	9.6953E-12
MYO6	ENSG00000196586	protein_coding	6840.46682	-1.2472857	0.07496156	-16.639004	3.6362E-62	1.3177E-60
ADH1B	ENSG00000196616	protein_coding	2002.58699	-2.5969406	0.26293335	-9.8768019	5.2481E-23	3.5939E-22
WNK3	ENSG00000196632	protein_coding	212.690366	-1.536934	0.17891949	-8.5900872	8.6903E-18	4.4521E-17
S100A2	ENSG00000196754	protein_coding	809.053185	-3.4670836	0.21153353	-16.390232	2.2459E-60	7.7176E-59
POU3F4	ENSG00000196767	protein_coding	54.3449723	-2.2741714	0.65654961	-3.4638227	0.00053256	0.00092631
HCAR1	ENSG00000196917	protein_coding	154.652118	-1.5225823	0.23246265	-6.5497931	5.7617E-11	1.8671E-10

SMIM10L2B	ENSG00000196972	protein_coding	208.025763	-1.4770983	0.14774532	-9.997598	1.5614E-23	1.1012E-22
LCE1C	ENSG00000197084	protein_coding	2.07090759	-1.9644268	0.47155986	-4.1658058	3.1025E-05	6.1579E-05
SLC6A17	ENSG00000197106	protein_coding	129.18909	-2.7525238	0.19820248	-13.887434	7.5492E-44	1.3876E-42
ADGRA1	ENSG00000197177	protein_coding	12.3349069	-2.4497449	0.41541999	-5.8970318	3.701E-09	1.0424E-08
ZNF165	ENSG00000197279	protein_coding	151.271012	-1.0155391	0.10268942	-9.8894221	4.6269E-23	3.1819E-22
ZNF98	ENSG00000197360	protein_coding	28.4624397	-2.1228127	0.24325382	-8.7267395	2.6212E-18	1.3869E-17
CYP2B6	ENSG00000197408	protein_coding	160.193073	-4.4968571	0.35337095	-12.725599	4.2619E-37	5.9167E-36
VEPH1	ENSG00000197415	protein_coding	1831.23614	-1.4836708	0.11922376	-12.444422	1.4997E-35	1.9403E-34
OGDHL	ENSG00000197444	protein_coding	7714.48586	-1.9204782	0.21057699	-9.1200765	7.5071E-20	4.3512E-19
COL4A6	ENSG00000197565	protein_coding	218.537308	-4.3747594	0.24231122	-18.0543	7.2982E-73	3.9084E-71
KCNMB2	ENSG00000197584	protein_coding	77.0719683	-2.0849597	0.23766968	-8.7725104	1.7473E-18	9.3527E-18
ENPP1	ENSG00000197594	protein_coding	744.216081	-1.0828839	0.14767512	-7.3328801	2.2526E-13	8.6599E-13
MYH6	ENSG00000197616	protein_coding	1.55915804	-1.3028423	0.28710452	-4.5378675	5.6826E-06	1.2112E-05
DNAH10	ENSG00000197653	protein_coding	105.021491	-1.0379209	0.11060937	-9.383662	6.372E-21	3.9056E-20
KLHL14	ENSG00000197705	protein_coding	260.625962	-3.8339317	0.17327572	-22.126191	1.769E-108	3.541E-106
OCLN	ENSG00000197822	protein_coding	645.01613	-2.0682075	0.17360595	-11.913229	1.0099E-32	1.1386E-31
ZNF44	ENSG00000197857	protein_coding	1114.07808	-1.3298115	0.07414735	-17.934713	6.3189E-72	3.2657E-70
ADAMTSL2	ENSG00000197859	protein_coding	1253.11973	-1.3215822	0.13148583	-10.051138	9.0812E-24	6.4914E-23
MEIG1	ENSG00000197889	protein_coding	5.90369653	-1.2771691	0.12092164	-10.561957	4.4724E-26	3.6345E-25
SLC22A12	ENSG00000197891	protein_coding	4223.52156	-2.0573421	0.30046128	-6.8472786	7.5268E-12	2.6015E-11
KIF13B	ENSG00000197892	protein_coding	4638.35727	-1.3194365	0.11410477	-11.563377	6.3174E-31	6.5231E-30
SLC22A6	ENSG00000197901	protein_coding	3170.63286	-2.9242387	0.32700273	-8.9425514	3.8029E-19	2.1131E-18
DNM3	ENSG00000197959	protein_coding	468.664495	-1.212682	0.09745506	-12.4435	1.5171E-35	1.9623E-34
DLGAP2	ENSG00000198010	protein_coding	34.9778022	-2.6546303	0.21882895	-12.131074	7.2295E-34	8.6349E-33
HIBCH	ENSG00000198130	protein_coding	2659.88369	-1.2748679	0.0932092	-13.677489	1.3839E-42	2.4112E-41

HMGN5	ENSG00000198157	protein_coding	433.890721	-1.0979037	0.07937658	-13.831583	1.6436E-43	2.9864E-42
TMEM116	ENSG00000198270	protein_coding	1060.74957	-1.1290474	0.13188923	-8.5605733	1.1231E-17	5.7146E-17
PEG3	ENSG00000198300	protein_coding	446.552869	-2.3604833	0.13643864	-17.300696	4.6474E-67	2.0061E-65
TMEM207	ENSG00000198398	protein_coding	108.653007	-8.2058902	0.55377627	-14.81806	1.1197E-49	2.6175E-48
NTRK1	ENSG00000198400	protein_coding	34.168945	-1.2989724	0.17147892	-7.5751141	3.5881E-14	1.4568E-13
MT1F	ENSG00000198417	protein_coding	1732.75739	-2.1674474	0.18685306	-11.599743	4.1332E-31	4.314E-30
CNGA1	ENSG00000198515	protein_coding	174.694923	-2.496254	0.18610191	-13.413371	5.0491E-41	8.2222E-40
SLC34A3	ENSG00000198569	protein_coding	217.543616	-3.4278474	0.24157888	-14.18935	1.0664E-45	2.1181E-44
ARC	ENSG00000198576	protein_coding	222.096565	-2.1044482	0.17985572	-11.700757	1.2632E-31	1.3552E-30
LRBA	ENSG00000198589	protein_coding	5849.01258	-1.1924697	0.081657	-14.603399	2.6719E-48	5.9027E-47
LPA	ENSG00000198670	protein_coding	20.5313903	-2.1835753	0.28751485	-7.5946522	3.0862E-14	1.258E-13
ABCA4	ENSG00000198691	protein_coding	398.148889	-4.6274351	0.23603862	-19.604568	1.4135E-85	1.2021E-83
ECI2	ENSG00000198721	protein_coding	4413.34881	-1.0757327	0.07204919	-14.930532	2.086E-50	5.0301E-49
SLC5A3	ENSG00000198743	protein_coding	8889.96886	-1.9073782	0.15000157	-12.715722	4.8362E-37	6.6917E-36
RASSF9	ENSG00000198774	protein_coding	339.648177	-1.440068	0.14724866	-9.7798371	1.3743E-22	9.2267E-22
FAM169A	ENSG00000198780	protein_coding	225.825163	-3.0829865	0.2342025	-13.163764	1.4184E-39	2.1812E-38
MTOR	ENSG00000198793	protein_coding	3354.89795	-1.1799852	0.05348672	-22.061272	7.446E-108	1.465E-105
PNP	ENSG00000198805	protein_coding	4583.49358	-1.4841508	0.07474523	-19.856127	9.755E-88	9.0157E-86
GK	ENSG00000198814	protein_coding	1393.40224	-1.5132601	0.12357156	-12.246022	1.7644E-34	2.1638E-33
SUCNR1	ENSG00000198829	protein_coding	1351.34994	-2.1600483	0.17593751	-12.277361	1.1984E-34	1.4832E-33
TOX	ENSG00000198846	protein_coding	536.285973	-1.23684	0.16705344	-7.4038582	1.3228E-13	5.1736E-13
PRMT6	ENSG00000198890	protein_coding	1035.4483	-1.2942837	0.06244314	-20.727395	1.9612E-95	2.4564E-93
BHLHB9	ENSG00000198908	protein_coding	410.313367	-1.0826844	0.07385303	-14.659987	1.1629E-48	2.6133E-47
L1CAM	ENSG00000198910	protein_coding	1995.3107	-3.1520122	0.29733169	-10.600996	2.9482E-26	2.4168E-25
SREBF2	ENSG00000198911	protein_coding	6083.49937	-1.2565977	0.07145112	-17.586816	3.1086E-69	1.4497E-67

NOS1AP	ENSG00000198929	protein_coding	84.2036283	-2.7880034	0.15496286	-17.991429	2.2742E-72	1.1901E-70
SOWAHA	ENSG00000198944	protein_coding	222.620543	-3.0746431	0.24609334	-12.493809	8.0696E-36	1.0542E-34
MFAP3L	ENSG00000198948	protein_coding	3308.25975	-1.7111578	0.12110652	-14.129361	2.5041E-45	4.8805E-44
RORB	ENSG00000198963	protein_coding	46.4164495	-2.2060821	0.27146105	-8.1266984	4.4114E-16	2.031E-15
STUM	ENSG00000203685	protein_coding	777.57709	-1.1079721	0.22775175	-4.8648237	1.1456E-06	2.6056E-06
CAPN8	ENSG00000203697	protein_coding	69.3390817	-1.810255	0.29022464	-6.2374272	4.4483E-10	1.3519E-09
CR1	ENSG00000203710	protein_coding	257.809415	-2.0005431	0.1913415	-10.455354	1.3848E-25	1.0954E-24
OR6K3	ENSG00000203757	protein_coding	0.2561116	-1.2851054	0.50671323	-2.5361592	0.01120758	0.0166564
PLPP4	ENSG00000203805	protein_coding	112.842532	-2.4801265	0.25338498	-9.7879775	1.2681E-22	8.5284E-22
SAMD13	ENSG00000203943	protein_coding	49.352886	-1.4790336	0.11949369	-12.377504	3.4594E-35	4.3831E-34
CCDC160	ENSG00000203952	protein_coding	143.891732	-2.3891231	0.15937847	-14.99025	8.5032E-51	2.08E-49
C1orf141	ENSG00000203963	protein_coding	1.22011314	-1.8786153	0.26178741	-7.1761101	7.1723E-13	2.6657E-12
LDLRAD1	ENSG00000203985	protein_coding	1.69821791	-1.8139206	0.38598285	-4.6994849	2.6082E-06	5.7385E-06
ZYG11A	ENSG00000203995	protein_coding	83.0259098	-1.3106741	0.21311316	-6.150132	7.7418E-10	2.3077E-09
TRPC5OS	ENSG00000204025	protein_coding	11.8683772	-1.3228705	0.19065357	-6.9386088	3.9598E-12	1.3961E-11
TCEAL5	ENSG00000204065	protein_coding	6.17495258	-1.1751257	0.37758148	-3.112244	0.00185671	0.00303987
TCEAL6	ENSG00000204071	protein_coding	3.99100155	-3.1974713	0.28982698	-11.032345	2.6681E-28	2.4307E-27
NEU4	ENSG00000204099	protein_coding	61.8208666	-1.7627842	0.2468856	-7.140085	9.3273E-13	3.4404E-12
SMIM5	ENSG00000204323	protein_coding	509.956647	-3.1309933	0.24978486	-12.53476	4.8182E-36	6.3688E-35
SLC44A4	ENSG00000204385	protein_coding	2048.23882	-1.9261337	0.20193573	-9.5383502	1.4512E-21	9.232E-21
VWA7	ENSG00000204396	protein_coding	419.678132	-1.5919477	0.11613254	-13.708025	9.0899E-43	1.599E-41
APOM	ENSG00000204444	protein_coding	1914.05389	-1.2900879	0.18479933	-6.9810206	2.9304E-12	1.0429E-11
MCCD1	ENSG00000204511	protein_coding	204.567184	-4.2662644	0.37656164	-11.329525	9.3712E-30	9.185E-29
AADACL4	ENSG00000204518	protein_coding	2.1744302	-2.7119426	0.27395585	-9.8991955	4.1964E-23	2.891E-22
TRIM40	ENSG00000204614	protein_coding	2.1382346	-2.0414642	0.27819395	-7.3382769	2.1636E-13	8.329E-13

ASPDH	ENSG00000204653	protein_coding	319.736703	-2.7555694	0.21181824	-13.009122	1.0858E-38	1.6025E-37
SPDYC	ENSG00000204710	protein_coding	2.26715866	-2.1077054	0.26462786	-7.9647903	1.655E-15	7.3573E-15
C9orf135	ENSG00000204711	protein_coding	14.5760115	-3.4947523	0.26739043	-13.069848	4.8963E-39	7.3591E-38
MALRD1	ENSG00000204740	protein_coding	39.1683061	-1.8799914	0.23050113	-8.1561048	3.46E-16	1.6023E-15
KRT40	ENSG00000204889	protein_coding	4.0910011	-3.2913537	0.30831225	-10.675391	1.327E-26	1.1108E-25
ZNF425	ENSG00000204947	protein_coding	165.476142	-1.2318592	0.06266076	-19.65918	4.8246E-86	4.2127E-84
ERICH4	ENSG00000204978	protein_coding	58.2296104	-3.6290895	0.19583201	-18.531647	1.1472E-76	7.1648E-75
PKHD1L1	ENSG00000205038	protein_coding	30.7760284	-2.4705416	0.22088506	-11.184738	4.8433E-29	4.5929E-28
CCNI2	ENSG00000205089	protein_coding	67.7135319	-2.9393716	0.14454241	-20.335704	6.2142E-92	6.7664E-90
FRG2	ENSG00000205097	protein_coding	1.6507654	-4.0107521	0.53385024	-7.5128787	5.7841E-14	2.3188E-13
TMEM88B	ENSG00000205116	protein_coding	1.06579863	-1.3655742	0.2671154	-5.1123003	3.1826E-07	7.6204E-07
VIT	ENSG00000205221	protein_coding	9.85736963	-1.2513795	0.26915115	-4.6493561	3.3297E-06	7.2578E-06
CTXN3	ENSG00000205279	protein_coding	322.454353	-3.4102038	0.39374495	-8.6609462	4.6786E-18	2.4397E-17
MT1H	ENSG00000205358	protein_coding	671.429458	-4.3660752	0.34371074	-12.702761	5.708E-37	7.8719E-36
INSYN1	ENSG00000205363	protein_coding	428.907744	-1.0717824	0.27675505	-3.8726751	0.00010765	0.00020205
LIN52	ENSG00000205659	protein_coding	627.217237	-1.1183448	0.06225115	-17.965047	3.6597E-72	1.9042E-70
ARSH	ENSG00000205667	protein_coding	1.00803603	-2.9854297	0.33691159	-8.8611667	7.9181E-19	4.3263E-18
ACOT6	ENSG00000205669	protein_coding	12.4122881	-2.2298554	0.18585237	-11.997993	3.6402E-33	4.2033E-32
CYS1	ENSG00000205795	protein_coding	6863.75055	-2.097973	0.1559669	-13.451399	3.021E-41	4.9654E-40
GMNC	ENSG00000205835	protein_coding	67.7300912	-3.5591986	0.32071517	-11.097693	1.2872E-28	1.1916E-27
C21orf62	ENSG00000205929	protein_coding	413.874195	-2.2341456	0.21330674	-10.473863	1.139E-25	9.0662E-25
SMIM21	ENSG00000206026	protein_coding	1.00962436	-1.2275201	0.33406454	-3.6745	0.00023832	0.00043068
VGLL3	ENSG00000206538	protein_coding	531.42628	-1.3169708	0.1903565	-6.9184443	4.5663E-12	1.6014E-11
XKR4	ENSG00000206579	protein_coding	41.0893938	-1.5849515	0.27321679	-5.8010766	6.589E-09	1.8166E-08
GPX3	ENSG00000211445	protein_coding	182621.631	-1.6257425	0.16178904	-10.048533	9.3245E-24	6.6539E-23

DIO1	ENSG00000211452	protein_coding	572.4254	-4.8069653	0.27939758	-17.204749	2.4463E-66	1.0269E-64
SLC48A1	ENSG00000211584	protein_coding	2322.20195	-1.4374866	0.07460891	-19.266958	1.0174E-82	7.7605E-81
SYT3	ENSG00000213023	protein_coding	29.1258905	-1.2713551	0.21957822	-5.7899874	7.0392E-09	1.9344E-08
DENND1B	ENSG00000213047	protein_coding	1229.00778	-1.0565664	0.09580619	-11.028164	2.7951E-28	2.5433E-27
TCL1B	ENSG00000213231	protein_coding	0.60512855	-1.273493	0.36356914	-3.5027532	0.00046048	0.00080669
SYNJ2BP	ENSG00000213463	protein_coding	3348.63556	-1.2368678	0.08188024	-15.105815	1.4826E-51	3.7261E-50
ATP6V1G2	ENSG00000213760	protein_coding	64.2607957	-1.1387831	0.10803967	-10.540416	5.6249E-26	4.5501E-25
DNASE1	ENSG00000213918	protein_coding	1720.62169	-2.163509	0.19556296	-11.062979	1.8969E-28	1.7391E-27
CLDN9	ENSG00000213937	protein_coding	39.9330316	-1.471622	0.19430566	-7.5737473	3.6261E-14	1.4711E-13
ZNF726	ENSG00000213967	protein_coding	78.5412205	-1.4445355	0.13275628	-10.881109	1.4183E-27	1.2441E-26
ZNF99	ENSG00000213973	protein_coding	21.1450761	-1.2507847	0.21446809	-5.8320319	5.4756E-09	1.5201E-08
WEE2	ENSG00000214102	protein_coding	11.4629597	-1.7685558	0.13922206	-12.703129	5.6812E-37	7.8396E-36
TMEM213	ENSG00000214128	protein_coding	4673.62955	-2.8479796	0.45715761	-6.2297542	4.6717E-10	1.4175E-09
PLIN5	ENSG00000214456	protein_coding	177.590311	-1.4477019	0.20351685	-7.1134254	1.132E-12	4.1513E-12
B3GNT10	ENSG00000214654	protein_coding	92.8140734	-1.2828889	0.10417979	-12.314183	7.5981E-35	9.4854E-34
IQCF6	ENSG00000214686	protein_coding	1.98038441	-1.8482367	0.2952432	-6.2600483	3.8486E-10	1.1742E-09
FER1L6	ENSG00000214814	protein_coding	84.8859283	-3.3589199	0.28106029	-11.950888	6.4236E-33	7.3195E-32
PNMA6E	ENSG00000214897	protein_coding	0.45736174	-1.2726441	0.49038683	-2.595184	0.00945403	0.0142018
RNF212B	ENSG00000215277	protein_coding	49.5987246	-4.1372982	0.201741	-20.50797	1.8277E-93	2.1428E-91
SIAH3	ENSG00000215475	protein_coding	70.7990596	-5.204702	0.2475327	-21.026321	3.7676E-98	5.2979E-96
GCGR	ENSG00000215644	protein_coding	310.305118	-2.247988	0.48634207	-4.6222362	3.7963E-06	8.2265E-06
PLXNA4	ENSG00000221866	protein_coding	266.825413	-2.4296537	0.21932531	-11.077854	1.6068E-28	1.4784E-27
ZNF844	ENSG00000223547	protein_coding	1343.03557	-1.0088061	0.1056594	-9.5477175	1.3259E-21	8.4473E-21
TINCR	ENSG00000223573	protein_coding	37.2374422	-2.334591	0.17101673	-13.651243	1.9846E-42	3.4279E-41
MKRN2OS	ENSG00000225526	protein_coding	21.5263409	-1.1430886	0.16985859	-6.7296485	1.7007E-11	5.728E-11

FRG2B	ENSG00000225899	protein_coding	4.45851758	-4.1495838	0.55783404	-7.4387424	1.0165E-13	4.0087E-13
ELFN1	ENSG00000225968	protein_coding	433.583323	-1.0154416	0.12721946	-7.9818101	1.442E-15	6.4372E-15
C13orf42	ENSG00000226792	protein_coding	4.5278247	-5.393785	0.25477204	-21.171024	1.767E-99	2.563E-97
ERVMER34-1	ENSG00000226887	protein_coding	161.27407	-4.6819289	0.23406034	-20.003085	5.1769E-89	5.0172E-87
C14orf132	ENSG00000227051	protein_coding	783.439734	-1.4263285	0.18746826	-7.6083731	2.7757E-14	1.1342E-13
MRLN	ENSG00000227877	protein_coding	18.4325462	-4.4998609	0.28896502	-15.572338	1.1223E-54	3.164E-53
IFNA14	ENSG00000228083	protein_coding	0.66648599	-4.4233199	0.5062114	-8.7380882	2.3709E-18	1.2575E-17
ANKRD63	ENSG00000230778	protein_coding	1.22502308	-2.3514343	0.27803616	-8.4572965	2.7365E-17	1.3614E-16
TMEM229A	ENSG00000234224	protein_coding	1.15834932	-1.9945069	0.35529799	-5.6136171	1.9814E-08	5.25E-08
PINLYP	ENSG00000234465	protein_coding	192.133573	-1.0174543	0.15365144	-6.6218342	3.5477E-11	1.1687E-10
MCIDAS	ENSG00000234602	protein_coding	6.46908159	-1.7491753	0.34905297	-5.0112031	5.4091E-07	1.2698E-06
SHISA8	ENSG00000234965	protein_coding	2.88348794	-1.5763311	0.28607788	-5.5101468	3.5853E-08	9.2904E-08
ANKRD34C	ENSG00000235711	protein_coding	4.11893599	-1.0367127	0.28194177	-3.6770455	0.00023595	0.00042664
ARHGEF38	ENSG00000236699	protein_coding	219.778057	-1.4269465	0.19889368	-7.1744183	7.2615E-13	2.698E-12
CKMT1B	ENSG00000237289	protein_coding	59.2532928	-1.0578111	0.37708946	-2.8051995	0.00502854	0.00782171
RNF223	ENSG00000237330	protein_coding	10.2848314	-2.7754583	0.31761441	-8.7384523	2.3633E-18	1.254E-17
C1orf226	ENSG00000239887	protein_coding	362.961938	-2.7751911	0.15271293	-18.1726	8.5069E-74	4.7326E-72
STRIT1	ENSG00000240045	protein_coding	18.3590862	-2.2141005	0.29459366	-7.5157779	5.6574E-14	2.2695E-13
KRBOX1	ENSG00000240747	protein_coding	23.1066356	-1.2076762	0.19595306	-6.1630893	7.1339E-10	2.1323E-09
PLCXD2	ENSG00000240891	protein_coding	208.678171	-1.5696277	0.17955723	-8.741657	2.2972E-18	1.2196E-17
TDGF1	ENSG00000241186	protein_coding	296.326211	-3.217344	0.36414867	-8.8352487	9.9876E-19	5.4227E-18
C3orf85	ENSG00000241224	protein_coding	54.8879444	-3.3361489	0.30490013	-10.941776	7.2761E-28	6.4929E-27
SPRR2A	ENSG00000241794	protein_coding	3.59626573	-2.1518537	0.46827629	-4.5952651	4.322E-06	9.3213E-06
HOGA1	ENSG00000241935	protein_coding	1849.16739	-1.919885	0.1603416	-11.973718	4.8792E-33	5.5973E-32
MRPL33	ENSG00000243147	protein_coding	1655.22262	-1.0816346	0.05951908	-18.172905	8.4598E-74	4.7121E-72

VSIG8	ENSG00000243284	protein_coding	27.441939	-2.8762172	0.16738035	-17.18372	3.5162E-66	1.4693E-64
MRPS6	ENSG00000243927	protein_coding	3439.15803	-1.5861902	0.11870964	-13.361932	1.0091E-40	1.6186E-39
ACY1	ENSG00000243989	protein_coding	540.089187	-2.2037769	0.11577121	-19.035621	8.6462E-81	6.193E-79
MT1HL1	ENSG00000244020	protein_coding	1.38006523	-2.6055064	0.24293772	-10.724997	7.7689E-27	6.5805E-26
CFHR1	ENSG00000244414	protein_coding	23.0292924	-1.3671065	0.38713755	-3.5313198	0.00041349	0.00072791
ERVFRD-1	ENSG00000244476	protein_coding	29.8086626	-1.9299696	0.18275071	-10.560668	4.5342E-26	3.684E-25
CCDC13	ENSG00000244607	protein_coding	72.5397313	-1.4129253	0.139149	-10.154045	3.1791E-24	2.3276E-23
ADH1C	ENSG00000248144	protein_coding	265.920545	-4.3513578	0.29298956	-14.85158	6.7948E-50	1.6023E-48
APELA	ENSG00000248329	protein_coding	28.6569514	-3.2414876	0.37624273	-8.6154159	6.9688E-18	3.5918E-17
PCP4L1	ENSG00000248485	protein_coding	12.8956819	-1.1875348	0.15123726	-7.8521311	4.0903E-15	1.7691E-14
ABHD14A	ENSG00000248487	protein_coding	747.448987	-1.0118787	0.09476879	-10.677341	1.2994E-26	1.0884E-25
C4orf54	ENSG00000248713	protein_coding	72.3306057	-6.7607351	0.39567543	-17.086568	1.8685E-65	7.607E-64
SMIM31	ENSG00000248771	protein_coding	3.53839005	-3.3211938	0.42613281	-7.7937999	6.5024E-15	2.7748E-14
PCDHA11	ENSG00000249158	protein_coding	68.9355884	-1.0988175	0.24038061	-4.5711569	4.8504E-06	1.0406E-05
AP000311.1	ENSG00000249209	protein_coding	0.3373482	-1.0923471	0.33661322	-3.2451103	0.00117405	0.00196717
CLRN2	ENSG00000249581	protein_coding	0.6134255	-1.665452	0.31222273	-5.3341792	9.5978E-08	2.4041E-07
HS3ST5	ENSG00000249853	protein_coding	10.7264972	-3.2106097	0.32073316	-10.010221	1.3745E-23	9.7262E-23
KIAA1210	ENSG00000250423	protein_coding	15.0752078	-2.1650815	0.20912783	-10.352909	4.0596E-25	3.124E-24
CCDC169-SOHLH2	ENSG00000250709	protein_coding	2.48827033	-1.1487446	0.2804302	-4.0963655	4.1969E-05	8.2246E-05
PRODH2	ENSG00000250799	protein_coding	1792.80906	-2.2834993	0.22703305	-10.058004	8.4698E-24	6.0619E-23
PCDHA12	ENSG00000251664	protein_coding	48.6983733	-2.0476374	0.24433975	-8.3802875	5.2799E-17	2.5792E-16
C1orf210	ENSG00000253313	protein_coding	711.743118	-1.4309104	0.18068866	-7.9192041	2.3904E-15	1.051E-14
TRNP1	ENSG00000253368	protein_coding	418.004132	-1.8869401	0.22272623	-8.4720154	2.4118E-17	1.204E-16
SMIM18	ENSG00000253457	protein_coding	4.23170839	-1.3134932	0.24473382	-5.3670275	8.0045E-08	2.0189E-07

AL512785.2	ENSG00000254706	protein_coding	2.81102773	-2.8701553	0.20882025	-13.744622	5.4859E-43	9.7511E-42
SMIM35	ENSG00000255274	protein_coding	1.06820103	-1.4249918	0.21544238	-6.6142598	3.7342E-11	1.2277E-10
AL590560.2	ENSG00000256029	protein_coding	3.69863598	-3.9741754	0.25311454	-15.701095	1.4865E-55	4.3102E-54
ASIC5	ENSG00000256394	protein_coding	1.56461909	-1.7486294	0.4571505	-3.8250629	0.00013074	0.00024305
SALL3	ENSG00000256463	protein_coding	84.8495616	-2.3324142	0.53161246	-4.3874332	1.147E-05	2.3737E-05
NHLRC4	ENSG00000257108	protein_coding	170.007518	-3.2095059	0.14780913	-21.713854	1.518E-104	2.729E-102
AC004080.3	ENSG00000257184	protein_coding	0.43878029	-1.6144392	0.31653579	-5.100337	3.3905E-07	8.0979E-07
AC008770.1	ENSG00000257355	protein_coding	0.96140549	-1.4184485	0.20600801	-6.8854048	5.7623E-12	2.008E-11
ZNF878	ENSG00000257446	protein_coding	15.9729413	-1.2400332	0.1204196	-10.297603	7.2239E-25	5.4744E-24
AL355102.2	ENSG00000258691	protein_coding	4.01885635	-1.1358345	0.30380482	-3.738698	0.00018498	0.00033856
BLOC1S5-TXNDC5	ENSG00000259040	protein_coding	50.1398485	-1.8226131	0.14723986	-12.378531	3.4154E-35	4.3286E-34
BUB1B-PAK6	ENSG00000259288	protein_coding	0.42032702	-1.8877946	0.47291399	-3.991835	6.5564E-05	0.00012571
CTXND1	ENSG00000259417	protein_coding	245.425164	-2.1324791	0.23191524	-9.1950797	3.7472E-20	2.2019E-19
LYPD8	ENSG00000259823	protein_coding	69.0471083	-1.4333191	0.26121708	-5.4870805	4.0863E-08	1.0541E-07
AL845331.1	ENSG00000259916	protein_coding	10.6022137	-1.8167049	0.17160757	-10.586391	3.4462E-26	2.8155E-25
TGFBR3L	ENSG00000260001	protein_coding	17.7103395	-1.9516277	0.17894992	-10.906	1.079E-27	9.5488E-27
CCDC187	ENSG00000260220	protein_coding	8.33544724	-1.6936102	0.37570715	-4.5077933	6.5505E-06	1.3879E-05
FRRS1L	ENSG00000260230	protein_coding	8.31797062	-1.7363052	0.27664134	-6.2763766	3.4655E-10	1.0607E-09
LY6L	ENSG00000261667	protein_coding	4.08248361	-4.1708561	0.61477565	-6.7843547	1.1661E-11	3.9725E-11
TCF24	ENSG00000261787	protein_coding	10.7520514	-5.0734983	0.27749193	-18.283408	1.1219E-74	6.4529E-73
MYMX	ENSG00000262179	protein_coding	9.53144426	-1.4936513	0.21791329	-6.8543378	7.1644E-12	2.4822E-11
MYZAP	ENSG00000263155	protein_coding	159.30423	-1.1884284	0.27217239	-4.3664549	1.2628E-05	2.6017E-05
TMEM238L	ENSG00000263429	protein_coding	74.616564	-7.1332945	0.20055506	-35.567762	4.416E-277	6.748E-273
AC135178.3	ENSG00000263809	protein_coding	8.3110833	-1.2500843	0.14035731	-8.9064424	5.2696E-19	2.9069E-18

DYNLL2	ENSG00000264364	protein_coding	8025.04598	-1.0149705	0.06446319	-15.744963	7.4374E-56	2.1757E-54
AC113554.1	ENSG00000264813	protein_coding	1.6685241	-1.8797732	0.24760839	-7.5917186	3.1569E-14	1.2857E-13
RBP3	ENSG00000265203	protein_coding	1.03675418	-2.6622515	0.31292106	-8.5077418	1.7735E-17	8.9234E-17
ZNF488	ENSG00000265763	protein_coding	33.5165401	-3.5317077	0.21630704	-16.32729	6.3127E-60	2.1341E-58
KLF14	ENSG00000266265	protein_coding	1.94130765	-1.0802882	0.31502172	-3.4292499	0.00060525	0.00104601
AC011195.2	ENSG00000266826	protein_coding	0.90380305	-1.0338815	0.28301664	-3.6530768	0.00025912	0.00046647
AC091551.1	ENSG00000267699	protein_coding	4.20071698	-1.0963004	0.13361678	-8.204811	2.3095E-16	1.0818E-15
AC011479.1	ENSG00000267748	protein_coding	9.53524259	-1.0213525	0.19006643	-5.3736606	7.7154E-08	1.9497E-07
SMIM22	ENSG00000267795	protein_coding	110.727039	-2.1674289	0.29277443	-7.4030677	1.3307E-13	5.2041E-13
ARL14EPL	ENSG00000268223	protein_coding	2.51826891	-2.5002021	0.37325319	-6.6984077	2.107E-11	7.0554E-11
SCGB1C2	ENSG00000268320	protein_coding	0.90667844	-2.2108467	0.51878112	-4.2616176	2.0295E-05	4.1008E-05
ZNF728	ENSG00000269067	protein_coding	18.3909942	-2.4197194	0.30600167	-7.9075368	2.6253E-15	1.1505E-14
ZIM2	ENSG00000269699	protein_coding	3.46295995	-1.6301361	0.19998829	-8.1511579	3.6046E-16	1.668E-15
RNF225	ENSG00000269855	protein_coding	0.6661046	-1.6652478	0.2771105	-6.0093276	1.8629E-09	5.3825E-09
C17orf50	ENSG00000270806	protein_coding	2.95365098	-1.0369105	0.21967937	-4.7201089	2.3572E-06	5.2057E-06
SMIM32	ENSG00000271824	protein_coding	728.657734	-1.2895428	0.26852086	-4.8023934	1.5678E-06	3.522E-06
FAM47E-STBD1	ENSG00000272414	protein_coding	5.24767132	-1.0457043	0.15364882	-6.8058075	1.0048E-11	3.4378E-11
DOC2B	ENSG00000272636	protein_coding	928.817798	-1.2957108	0.12356018	-10.486475	9.9678E-26	7.9564E-25
C2orf15	ENSG00000273045	protein_coding	130.556814	-1.8561129	0.10538297	-17.613025	1.957E-69	9.2011E-68
AL359736.1	ENSG00000273167	protein_coding	1.51444358	-3.0382908	0.17166778	-17.698666	4.2935E-70	2.074E-68
AL049839.2	ENSG00000273259	protein_coding	12.1881517	-1.947991	0.30288921	-6.4313647	1.2646E-10	3.9942E-10
ZBTB8B	ENSG00000273274	protein_coding	6.91738499	-1.620617	0.18712276	-8.6607158	4.6881E-18	2.4441E-17
LHX1	ENSG00000273706	protein_coding	173.506137	-3.4063842	0.38169169	-8.924439	4.4796E-19	2.4792E-18
CBSL	ENSG00000274276	protein_coding	4.51240009	-1.3814224	0.30344988	-4.5523907	5.304E-06	1.1334E-05
CCL23	ENSG00000274736	protein_coding	21.8674582	-1.4910024	0.13013129	-11.457677	2.1521E-30	2.1711E-29

AC117457.1	ENSG00000275163	protein_coding	1.76875621	-2.3958193	0.39814308	-6.0174832	1.7715E-09	5.1267E-09
FCGBP	ENSG00000275395	protein_coding	2386.78779	-1.1383288	0.20143066	-5.651219	1.5931E-08	4.2576E-08
AL627230.1	ENSG00000275493	protein_coding	2.65311406	-1.3872927	0.21698337	-6.3935436	1.6208E-10	5.0804E-10
CRYAA2	ENSG00000276076	protein_coding	6.05971249	-3.9054677	0.70974602	-5.5026272	3.7417E-08	9.6841E-08
KCNE1B	ENSG00000276289	protein_coding	9.78325103	-5.1687588	0.27283196	-18.944844	4.8703E-80	3.4034E-78
DACH1	ENSG00000276644	protein_coding	596.437405	-2.3835038	0.14907668	-15.988442	1.5383E-57	4.7971E-56
PNMA8C	ENSG00000277531	protein_coding	1.99836396	-1.0848617	0.22101745	-4.9084888	9.1781E-07	2.108E-06
OR13C5	ENSG00000277556	protein_coding	0.36591739	-1.5058325	0.30502514	-4.9367489	7.9436E-07	1.8358E-06
AC007906.2	ENSG00000277639	protein_coding	285.48434	-3.9931773	0.19016348	-20.998655	6.7468E-98	9.3439E-96
SRD5A2	ENSG00000277893	protein_coding	12.5027885	-2.2498825	0.22113536	-10.174232	2.5843E-24	1.9043E-23
SSTR3	ENSG00000278195	protein_coding	9.72753176	-1.0351789	0.21871974	-4.7329012	2.2133E-06	4.9015E-06
DHRS11	ENSG00000278535	protein_coding	502.502479	-1.4389093	0.09186105	-15.663975	2.6669E-55	7.6553E-54
PCDH20	ENSG00000280165	protein_coding	4.17638379	-1.3460831	0.29377632	-4.5820002	4.6055E-06	9.9067E-06
AC006059.2	ENSG00000280571	protein_coding	1.07156003	-1.0625097	0.20368444	-5.2164503	1.8238E-07	4.4612E-07
BLACAT1	ENSG00000281406	protein_coding	27.4059526	-1.4705494	0.22885999	-6.4255418	1.314E-10	4.1457E-10
FGR	ENSG00000000938	protein_coding	1056.34837	1.50711095	0.09995442	15.0779817	2.2607E-51	5.6446E-50
SEMA3F	ENSG00000001617	protein_coding	6403.27312	1.13742379	0.08976549	12.671059	8.5552E-37	1.1721E-35
CD99	ENSG00000002586	protein_coding	9710.44075	1.26431236	0.08537479	14.8089662	1.282E-49	2.9891E-48
TMEM176A	ENSG00000002933	protein_coding	29342.985	1.27274993	0.12599214	10.1018203	5.4226E-24	3.9208E-23
TFPI	ENSG00000003436	protein_coding	12877.7018	1.06102541	0.14387908	7.37442431	1.6506E-13	6.4127E-13
PLXND1	ENSG00000004399	protein_coding	14457.9086	1.59208185	0.09135616	17.4271982	5.1299E-68	2.2853E-66
CD38	ENSG00000004468	protein_coding	489.708311	1.85108593	0.16191888	11.432181	2.8876E-30	2.8946E-29
CAMKK1	ENSG00000004660	protein_coding	890.25019	1.45535411	0.08297678	17.5392946	7.1813E-69	3.3119E-67
ARHGAP33	ENSG00000004777	protein_coding	638.301678	2.09301764	0.14362768	14.5725231	4.2008E-48	9.2095E-47
SLC22A16	ENSG00000004809	protein_coding	16.4815187	2.10049003	0.218326	9.62088815	6.5265E-22	4.2329E-21

ABCB5	ENSG00000004846	protein_coding	21.1718343	2.04426612	0.30653706	6.66890374	2.5772E-11	8.5809E-11
ARX	ENSG00000004848	protein_coding	48.9530364	3.54513545	0.40306681	8.79540394	1.4253E-18	7.6707E-18
CALCR	ENSG00000004948	protein_coding	382.254578	1.17517084	0.23274558	5.04916503	4.4375E-07	1.0495E-06
MCUB	ENSG00000005059	protein_coding	638.822374	1.63366744	0.11125226	14.6843526	8.1206E-49	1.8356E-47
WDR54	ENSG00000005448	protein_coding	1728.1218	1.14841891	0.09522678	12.0598319	1.7213E-33	2.0145E-32
ABCB4	ENSG00000005471	protein_coding	337.57066	2.1030136	0.16736505	12.5654284	3.271E-36	4.3589E-35
ITGAL	ENSG00000005844	protein_coding	2376.72049	2.50740986	0.13231635	18.950114	4.4063E-80	3.098E-78
YBX2	ENSG00000006047	protein_coding	179.918131	1.34553515	0.19979025	6.73473871	1.6422E-11	5.5383E-11
KRT33A	ENSG00000006059	protein_coding	2.37247729	2.89853668	0.42625055	6.80007723	1.0456E-11	3.5733E-11
MAP3K14	ENSG00000006062	protein_coding	2430.2516	1.36323192	0.09850039	13.8398635	1.4648E-43	2.6679E-42
TMEM132A	ENSG00000006118	protein_coding	2039.13719	1.20605431	0.13196581	9.13914194	6.295E-20	3.6625E-19
DLX6	ENSG00000006377	protein_coding	26.5359064	2.58675268	0.16813814	15.3846869	2.0738E-53	5.5953E-52
LGALS14	ENSG00000006659	protein_coding	0.86981621	2.08314255	0.48154881	4.32592188	1.519E-05	3.1054E-05
MYH13	ENSG00000006788	protein_coding	25.5107963	4.70486741	0.40499197	11.6171869	3.3705E-31	3.5373E-30
PRSS21	ENSG00000007038	protein_coding	12.6793997	2.26818711	0.25083763	9.04245137	1.532E-19	8.7206E-19
CEACAM21	ENSG00000007129	protein_coding	122.882104	1.85253404	0.12109209	15.2985556	7.8172E-53	2.0714E-51
MATK	ENSG00000007264	protein_coding	316.79853	2.24558281	0.12791547	17.5552089	5.4267E-69	2.5102E-67
SCN4A	ENSG00000007314	protein_coding	398.009715	1.63478412	0.17759552	9.20509773	3.4136E-20	2.0095E-19
LUC7L	ENSG00000007392	protein_coding	1852.29869	1.03069197	0.10693286	9.63868361	5.4887E-22	3.5715E-21
E2F2	ENSG00000007968	protein_coding	115.513363	2.34677931	0.13012353	18.0350106	1.0348E-72	5.4964E-71
SYN1	ENSG00000008056	protein_coding	134.059637	1.1862748	0.16924318	7.00929141	2.3953E-12	8.5756E-12
TFAP2D	ENSG00000008197	protein_coding	0.94298707	2.51030852	0.5232717	4.79733284	1.6079E-06	3.6073E-06
CELSR3	ENSG00000008300	protein_coding	149.725924	1.47578007	0.1542499	9.56746187	1.0956E-21	7.0195E-21
PGLYRP1	ENSG00000008438	protein_coding	7.0970313	1.23502626	0.21067066	5.86235535	4.5635E-09	1.276E-08
MMP25	ENSG00000008516	protein_coding	149.876536	2.08303087	0.11685698	17.8254731	4.4828E-71	2.2582E-69

IL32	ENSG00000008517	protein_coding	14531.8976	1.96517301	0.13419565	14.6440893	1.4696E-48	3.2862E-47
RPS20	ENSG00000008988	protein_coding	45728.5265	1.0599054	0.09312425	11.3816259	5.1628E-30	5.1116E-29
TENM1	ENSG00000009694	protein_coding	1366.07542	2.4452697	0.15717553	15.5575722	1.4136E-54	3.9609E-53
PAX7	ENSG00000009709	protein_coding	1.05982318	1.34788077	0.46447668	2.90193422	0.00370866	0.00587088
TRAF3IP3	ENSG00000009790	protein_coding	537.720628	1.84218407	0.13041247	14.1258271	2.6329E-45	5.1251E-44
ETV7	ENSG00000010030	protein_coding	519.660096	2.68774046	0.13257256	20.2737319	2.1937E-91	2.3118E-89
HHATL	ENSG00000010282	protein_coding	142.592129	4.60448965	0.46385003	9.92667746	3.187E-23	2.2105E-22
IFFO1	ENSG00000010295	protein_coding	1377.12627	1.75919374	0.09382041	18.7506501	1.9124E-78	1.2742E-76
STAB1	ENSG00000010327	protein_coding	8251.63299	1.68569066	0.10754397	15.6744315	2.2624E-55	6.5145E-54
SLC6A13	ENSG00000010379	protein_coding	9143.43665	1.03338784	0.19802986	5.21834344	1.8053E-07	4.4177E-07
CD4	ENSG00000010610	protein_coding	5580.93079	1.77837053	0.106207	16.7443813	6.2234E-63	2.3063E-61
BTK	ENSG00000010671	protein_coding	596.104184	2.17328735	0.11311321	19.2133823	2.8601E-82	2.1458E-80
NME1-NME2	ENSG00000011052	protein_coding	27.9745446	1.04430831	0.11888318	8.78432315	1.5731E-18	8.445E-18
SLC6A7	ENSG00000011083	protein_coding	4.91642474	1.98080189	0.24523807	8.07705718	6.6348E-16	3.0182E-15
ANOS1	ENSG00000011201	protein_coding	549.507213	1.00145457	0.12266548	8.16411066	3.2381E-16	1.5024E-15
DPF1	ENSG00000011332	protein_coding	17.6495131	1.1676141	0.17482238	6.67885924	2.4081E-11	8.0342E-11
PLAUR	ENSG00000011422	protein_coding	1310.11121	1.54510985	0.14414096	10.7194366	8.2504E-27	6.9742E-26
ANLN	ENSG00000011426	protein_coding	664.554173	2.3051503	0.15898184	14.4994564	1.2211E-47	2.6159E-46
ZBTB32	ENSG00000011590	protein_coding	38.5568458	1.79698842	0.14044714	12.794767	1.7538E-37	2.4737E-36
TYROBP	ENSG00000011600	protein_coding	4611.63749	2.65058809	0.12228064	21.6762697	3.436E-104	6.106E-102
CD22	ENSG00000012124	protein_coding	270.976467	1.24313338	0.15392825	8.07605744	6.6894E-16	3.0428E-15
ALOX5	ENSG00000012779	protein_coding	2466.55232	2.0804627	0.1504414	13.829057	1.7024E-43	3.0906E-42
CLK1	ENSG00000013441	protein_coding	5184.56302	1.14931445	0.1005843	11.4263803	3.0871E-30	3.0899E-29
DDX11	ENSG00000013573	protein_coding	979.988919	1.26295417	0.10727813	11.7727084	5.3964E-32	5.8941E-31
CD6	ENSG00000013725	protein_coding	613.103314	2.3539462	0.13500259	17.4363038	4.3747E-68	1.9584E-66

TACC3	ENSG00000013810	protein_coding	1010.20016	1.45543656	0.09803557	14.8460043	7.3841E-50	1.7394E-48
MTMR11	ENSG00000014914	protein_coding	3111.5826	1.58564994	0.11211	14.1436983	2.0426E-45	3.9896E-44
WAS	ENSG00000015285	protein_coding	1007.18579	2.16625347	0.11824933	18.3193717	5.7978E-75	3.3643E-73
BID	ENSG00000015475	protein_coding	1438.04388	1.09680589	0.0656298	16.7120091	1.0716E-62	3.9521E-61
CLCA1	ENSG00000016490	protein_coding	0.60503899	1.16107388	0.52386408	2.21636473	0.02666653	0.0375667
SLC38A5	ENSG00000017483	protein_coding	732.717521	1.71952225	0.26427416	6.50658493	7.6878E-11	2.4707E-10
SLC11A1	ENSG00000018280	protein_coding	726.17681	2.37978064	0.14196463	16.7631942	4.5359E-63	1.6905E-61
MARCO	ENSG00000019169	protein_coding	287.420255	2.37453526	0.23683454	10.0261359	1.1701E-23	8.3081E-23
CD74	ENSG00000019582	protein_coding	239481.823	1.74305166	0.11148168	15.6353194	4.1837E-55	1.1949E-53
ADGRA2	ENSG00000020181	protein_coding	3973.87366	1.32745771	0.11504973	11.5381208	8.4757E-31	8.6959E-30
RUNX3	ENSG00000020633	protein_coding	1378.11567	2.66924409	0.13152381	20.2947588	1.4305E-91	1.5215E-89
CYP3A43	ENSG00000021461	protein_coding	9.66779966	1.78570979	0.20367571	8.76741658	1.8281E-18	9.7718E-18
NRXN3	ENSG00000021645	protein_coding	702.781609	1.37754327	0.16849136	8.17575041	2.9403E-16	1.368E-15
FHL1	ENSG00000022267	protein_coding	18572.2211	1.70179169	0.16099697	10.5703335	4.0904E-26	3.3287E-25
BIRC3	ENSG00000023445	protein_coding	10071.2487	2.31413611	0.14189471	16.3088253	8.542E-60	2.8792E-58
DEF6	ENSG00000023892	protein_coding	767.6802	2.26194384	0.12063636	18.7501006	1.9323E-78	1.2856E-76
PLEKHO1	ENSG00000023902	protein_coding	2913.83642	1.77819381	0.10362981	17.1590955	5.3744E-66	2.2316E-64
EHD2	ENSG00000024422	protein_coding	18657.3155	2.23520712	0.10456664	21.3759098	2.239E-101	3.552E-99
DEPDC1	ENSG00000024526	protein_coding	150.180293	2.38028215	0.15682867	15.1775954	4.977E-52	1.2853E-50
NR1H3	ENSG00000025434	protein_coding	2169.35124	1.0227372	0.0761032	13.4388205	3.581E-41	5.8606E-40
TYMP	ENSG00000025708	protein_coding	5774.87734	2.93744104	0.12401461	23.6862508	4.997E-124	1.58E-121
VIM	ENSG00000026025	protein_coding	166640.828	2.5599207	0.09459181	27.0628147	2.699E-161	2.475E-158
RTEL1-TNFRSF6B	ENSG00000026036	protein_coding	197.760279	2.46614551	0.14768406	16.6987925	1.3374E-62	4.9087E-61
FAS	ENSG00000026103	protein_coding	1985.33023	1.19284739	0.08335751	14.3100166	1.8947E-46	3.8791E-45

RNASET2	ENSG00000026297	protein_coding	13576.2515	3.34853997	0.15719255	21.3021538	1.084E-100	1.6622E-98
CD44	ENSG00000026508	protein_coding	10520.496	1.40020237	0.14319926	9.77799996	1.3995E-22	9.3902E-22
SLAMF7	ENSG00000026751	protein_coding	1168.21018	2.80827451	0.1716285	16.3625189	3.5417E-60	1.2089E-58
BTN3A1	ENSG00000026950	protein_coding	4511.41836	1.86557106	0.08979652	20.7755389	7.2051E-96	9.2778E-94
SH2D2A	ENSG00000027869	protein_coding	340.112355	2.72531249	0.15952088	17.0843623	1.9405E-65	7.8932E-64
TNFRSF1B	ENSG00000028137	protein_coding	4455.96589	1.41505369	0.0889372	15.9107062	5.3408E-57	1.6224E-55
POU2F2	ENSG00000028277	protein_coding	668.023035	1.9407829	0.13575941	14.2957522	2.3258E-46	4.7385E-45
ANK1	ENSG00000029534	protein_coding	649.292698	2.21122609	0.19149129	11.5473975	7.609E-31	7.8277E-30
IBSP	ENSG00000029559	protein_coding	60.5087507	5.13101889	0.32845371	15.6217412	5.1772E-55	1.475E-53
LRRC7	ENSG00000033122	protein_coding	190.234881	1.46899756	0.17061424	8.61005239	7.3027E-18	3.7597E-17
APBA2	ENSG00000034053	protein_coding	208.224017	1.24993379	0.17613816	7.09632596	1.2812E-12	4.6816E-12
TMSB10	ENSG00000034510	protein_coding	68952.6592	2.12835549	0.10822741	19.6655858	4.2523E-86	3.72E-84
SLC18A1	ENSG00000036565	protein_coding	6.21915507	1.15840664	0.19720398	5.87415437	4.2501E-09	1.1912E-08
FLT4	ENSG00000037280	protein_coding	4613.87226	1.12311768	0.11334593	9.90876033	3.8135E-23	2.6327E-22
TLL1	ENSG00000038295	protein_coding	974.297083	1.52909179	0.14777738	10.3472654	4.3061E-25	3.3065E-24
VCAN	ENSG00000038427	protein_coding	19810.9706	1.87844208	0.17311975	10.8505357	1.9826E-27	1.7282E-26
MSR1	ENSG00000038945	protein_coding	3436.17122	2.06575296	0.13645649	15.1385468	9.0185E-52	2.2929E-50
C6	ENSG00000039537	protein_coding	714.964318	1.53266997	0.24988061	6.13360904	8.5907E-10	2.5506E-09
BEST2	ENSG00000039987	protein_coding	2.2204805	2.18866744	0.34438459	6.35530012	2.0802E-10	6.4615E-10
RTN4R	ENSG00000040608	protein_coding	181.39355	1.09671736	0.13100562	8.37152934	5.6875E-17	2.7713E-16
C2orf83	ENSG00000042304	protein_coding	1.45711397	2.82433639	0.47291707	5.97215996	2.3413E-09	6.7122E-09
ADAM28	ENSG00000042980	protein_coding	1183.51761	1.28087754	0.11541071	11.0984286	1.2767E-28	1.1821E-27
BARX2	ENSG00000043039	protein_coding	2554.49532	2.6288891	0.13677335	19.2207699	2.4806E-82	1.8695E-80
ZIC2	ENSG00000043355	protein_coding	14.2841749	3.04613688	0.39456094	7.72032041	1.1604E-14	4.872E-14
LCP2	ENSG00000043462	protein_coding	2848.49075	2.20569397	0.09774272	22.5663259	9.285E-113	2.118E-110

GUCA2B	ENSG00000044012	protein_coding	99.0836812	5.77097026	0.33577956	17.1867827	3.3353E-66	1.395E-64
PHKA2	ENSG00000044446	protein_coding	7807.53033	2.17015142	0.09119493	23.7968429	3.601E-125	1.171E-122
EPHA3	ENSG00000044524	protein_coding	1015.34529	1.74767726	0.14269944	12.2472607	1.7377E-34	2.1333E-33
MAGEC2	ENSG00000046774	protein_coding	3.04132358	2.78826852	0.85445872	3.26319861	0.00110162	0.00185115
PREX2	ENSG00000046889	protein_coding	3308.77373	2.05495677	0.16323815	12.5887044	2.4364E-36	3.2686E-35
CP	ENSG00000047457	protein_coding	25699.9141	4.5845425	0.23686879	19.3547766	1.858E-83	1.4559E-81
NOP16	ENSG00000048162	protein_coding	992.790096	1.11543142	0.07700185	14.4857734	1.4904E-47	3.1836E-46
TNFRSF17	ENSG00000048462	protein_coding	40.9928792	1.57605484	0.25532491	6.17274222	6.7116E-10	2.0094E-09
GUCA1A	ENSG00000048545	protein_coding	1.65688014	1.08472891	0.36713578	2.9545715	0.00313104	0.00500086
UTS2	ENSG00000049247	protein_coding	19.3246657	2.76201253	0.24594856	11.2300416	2.9034E-29	2.7908E-28
TNFRSF9	ENSG00000049249	protein_coding	514.184837	4.06039562	0.20894705	19.4326532	4.0868E-84	3.2752E-82
RFC2	ENSG00000049541	protein_coding	1537.69566	1.09869271	0.05683532	19.331162	2.9373E-83	2.2745E-81
FOXP3	ENSG00000049768	protein_coding	165.949276	1.6826817	0.16564273	10.1585004	3.0371E-24	2.2273E-23
NFE2L3	ENSG00000050344	protein_coding	2189.23601	1.36892376	0.11324473	12.0881895	1.2194E-33	1.4407E-32
TNIP3	ENSG00000050730	protein_coding	86.2313594	3.75376113	0.22325714	16.8136222	1.9395E-63	7.3661E-62
COL23A1	ENSG00000050767	protein_coding	11256.1869	5.17685488	0.17038312	30.3836137	9.043E-203	2.591E-199
RAD51	ENSG00000051180	protein_coding	134.413033	1.5927322	0.10594766	15.0331988	4.4499E-51	1.0961E-49
POLQ	ENSG00000051341	protein_coding	104.545882	2.14251467	0.14326372	14.9550397	1.444E-50	3.506E-49
CYBA	ENSG00000051523	protein_coding	7571.56089	1.22368575	0.11240072	10.8868145	1.3322E-27	1.1706E-26
ENTPD2	ENSG00000054179	protein_coding	741.346631	1.22596845	0.15514027	7.90232276	2.7375E-15	1.1988E-14
PTPRN	ENSG00000054356	protein_coding	340.374773	5.33740294	0.25829898	20.6636623	7.3567E-95	9.0656E-93
CBLN4	ENSG00000054803	protein_coding	259.369755	2.06515321	0.20796956	9.93007436	3.0803E-23	2.1381E-22
RELT	ENSG00000054967	protein_coding	465.914658	2.0776956	0.09376155	22.1593574	8.475E-109	1.742E-106
ITIH4	ENSG00000055955	protein_coding	51.7411034	2.38044796	0.22405815	10.624242	2.2987E-26	1.8973E-25
ITIH1	ENSG00000055957	protein_coding	63.387947	4.66034761	0.34268388	13.599553	4.0289E-42	6.8504E-41

TRAF3IP2	ENSG00000056972	protein_coding	2451.25366	1.05434442	0.07156687	14.7322985	3.9987E-49	9.1333E-48
MSH4	ENSG00000057468	protein_coding	28.0416861	2.93231123	0.20321585	14.4295402	3.3732E-47	7.1095E-46
PRDM1	ENSG00000057657	protein_coding	2038.78026	2.26236893	0.09688079	23.352091	1.312E-120	3.691E-118
TBXAS1	ENSG00000059377	protein_coding	1669.54521	1.79176609	0.08737138	20.5074727	1.8464E-93	2.1592E-91
SLC2A3	ENSG00000059804	protein_coding	7391.57933	2.01500605	0.13906468	14.4897035	1.4075E-47	3.0094E-46
YBX3	ENSG00000060138	protein_coding	16304.4278	1.50740828	0.07802395	19.319815	3.6596E-83	2.8148E-81
OGFR	ENSG00000060491	protein_coding	3206.04062	1.04444165	0.06900722	15.1352514	9.4818E-52	2.4041E-50
GNA15	ENSG00000060558	protein_coding	403.823228	1.73557852	0.11494575	15.0991098	1.6413E-51	4.116E-50
CREB3L3	ENSG00000060566	protein_coding	1166.08457	2.66797304	0.25289861	10.5495757	5.1027E-26	4.1349E-25
COL11A1	ENSG00000060718	protein_coding	307.552106	2.70374024	0.27948921	9.67386262	3.894E-22	2.5511E-21
BCAT1	ENSG00000060982	protein_coding	1510.75111	1.42557216	0.14348312	9.93546947	2.918E-23	2.0273E-22
LZTS1	ENSG00000061337	protein_coding	2441.48694	2.37662985	0.1188446	19.997794	5.7563E-89	5.567E-87
WNT8A	ENSG00000061492	protein_coding	1.11465874	2.13920796	0.37320436	5.73200155	9.9252E-09	2.6948E-08
SPAG4	ENSG00000061656	protein_coding	2538.8419	4.09652261	0.11860413	34.5394589	2.052E-261	1.881E-257
GUCY1B1	ENSG00000061918	protein_coding	3898.01995	1.18162428	0.08283698	14.2644547	3.6443E-46	7.3594E-45
TNK2	ENSG00000061938	protein_coding	2150.86486	1.37559893	0.0912035	15.0827429	2.1035E-51	5.2662E-50
VMP1	ENSG00000062716	protein_coding	11882.6207	1.27696302	0.10441417	12.2297868	2.155E-34	2.6288E-33
SEZ6	ENSG00000063015	protein_coding	8.99021137	1.0027053	0.20880697	4.8020682	1.5704E-06	3.5274E-06
RPL18	ENSG00000063177	protein_coding	34909.9704	1.15104939	0.09307814	12.3664842	3.9681E-35	5.0125E-34
AHRR	ENSG00000063438	protein_coding	232.474231	1.46970864	0.16252675	9.04287243	1.5261E-19	8.6892E-19
TSPAN32	ENSG00000064201	protein_coding	123.839567	2.00550995	0.12383526	16.1949834	5.4713E-59	1.7966E-57
CCN5	ENSG00000064205	protein_coding	362.998324	3.33482262	0.28426303	11.7314681	8.792E-32	9.4877E-31
DMRT3	ENSG00000064218	protein_coding	8.34497022	4.30815822	0.48125317	8.95195802	3.4923E-19	1.9464E-18
NGFR	ENSG00000064300	protein_coding	1847.40023	1.84317615	0.15799698	11.6658949	1.9039E-31	2.0231E-30
CDON	ENSG00000064309	protein_coding	1630.50344	2.22656344	0.12989429	17.1413495	7.2938E-66	3.0068E-64

CHI3L2	ENSG00000064886	protein_coding	377.703286	2.30361892	0.216092	10.6603618	1.5599E-26	1.3008E-25
MCM10	ENSG00000065328	protein_coding	113.020178	2.50640912	0.13351317	18.7727479	1.2619E-78	8.42E-77
DGKA	ENSG00000065357	protein_coding	973.288706	1.12060171	0.10030638	11.1717887	5.6041E-29	5.2968E-28
PDIA5	ENSG00000065485	protein_coding	4019.85485	1.74326515	0.10526436	16.56083	1.3374E-61	4.7451E-60
COL17A1	ENSG00000065618	protein_coding	97.692522	1.08291128	0.23533774	4.60151982	4.1942E-06	9.0585E-06
ASPM	ENSG00000066279	protein_coding	366.889263	2.62854867	0.15542832	16.911646	3.6925E-64	1.4369E-62
CD84	ENSG00000066294	protein_coding	1799.12987	2.7723769	0.1490797	18.5966093	3.4228E-77	2.1762E-75
SPI1	ENSG00000066336	protein_coding	1807.87913	2.34882363	0.11468502	20.4806494	3.2036E-93	3.7085E-91
STAG3	ENSG00000066923	protein_coding	288.595678	1.28119333	0.11383447	11.2548805	2.1912E-29	2.1143E-28
PFKP	ENSG00000067057	protein_coding	27918.2438	1.95940735	0.09409146	20.8244964	2.5964E-96	3.4399E-94
CACNB1	ENSG00000067191	protein_coding	263.3341	1.30038294	0.12035399	10.8046513	3.272E-27	2.8252E-26
PRR11	ENSG00000068489	protein_coding	344.727992	1.88608038	0.13195689	14.2931555	2.4142E-46	4.9099E-45
ATP11A	ENSG00000068650	protein_coding	16463.0183	1.47662854	0.11382424	12.972883	1.7435E-38	2.551E-37
RASGRP2	ENSG00000068831	protein_coding	621.920181	1.05205731	0.10651247	9.87731608	5.2213E-23	3.5761E-22
PAGE1	ENSG00000068985	protein_coding	0.94644784	1.50790701	0.6885023	2.19012633	0.02851508	0.03997552
PITX1	ENSG00000069011	protein_coding	83.1800861	3.64993537	0.36501073	9.9995289	1.5312E-23	1.0802E-22
CLEC2D	ENSG00000069493	protein_coding	864.811759	2.55023099	0.12172516	20.9507301	1.8478E-97	2.5285E-95
DRD4	ENSG00000069696	protein_coding	50.5756172	1.97842589	0.1514936	13.059468	5.6119E-39	8.3987E-38
PLA2G10	ENSG00000069764	protein_coding	7.41950392	1.14362075	0.15192212	7.5276778	5.1651E-14	2.0771E-13
FSTL3	ENSG00000070404	protein_coding	5924.68359	1.74829479	0.15035214	11.6280005	2.9697E-31	3.1267E-30
CHAT	ENSG00000070748	protein_coding	7.48540539	5.20587829	0.61069689	8.52448799	1.5349E-17	7.7489E-17
MAP4K4	ENSG00000071054	protein_coding	8594.14362	1.19394204	0.07030011	16.9835016	1.088E-64	4.3218E-63
MS4A12	ENSG00000071203	protein_coding	0.72096237	1.77517902	0.55406779	3.20390222	0.00135579	0.00225551
VASH1	ENSG00000071246	protein_coding	3057.58712	2.16271092	0.09770914	22.1341715	1.482E-108	3.006E-106
TRIP13	ENSG00000071539	protein_coding	297.730262	1.76847931	0.11662301	15.1640681	6.1161E-52	1.5733E-50

SPP2	ENSG00000072080	protein_coding	0.44382352	1.29942662	0.47593314	2.73027134	0.00632822	0.00973332
ASIC4	ENSG00000072182	protein_coding	8.45623853	2.16924972	0.23957723	9.05449054	1.3721E-19	7.8298E-19
HMMR	ENSG00000072571	protein_coding	225.339629	1.36104934	0.1425015	9.55112269	1.283E-21	8.181E-21
CHFR	ENSG00000072609	protein_coding	1320.43756	1.11223766	0.06348578	17.5194764	1.0176E-68	4.6693E-67
P4HA2	ENSG00000072682	protein_coding	6535.09149	1.26236149	0.10186606	12.3923654	2.8744E-35	3.6662E-34
FCGR2B	ENSG00000072694	protein_coding	579.433609	1.97988601	0.15351962	12.8966318	4.702E-38	6.7867E-37
STK10	ENSG00000072786	protein_coding	3197.08277	1.18016187	0.06481195	18.2090172	4.3773E-74	2.4531E-72
ACAP1	ENSG00000072818	protein_coding	916.462084	1.84074704	0.11566344	15.9146841	5.0119E-57	1.5256E-55
SCARB1	ENSG00000073060	protein_coding	13776.3526	4.06829782	0.13288483	30.615216	7.679E-206	2.347E-202
MOV10L1	ENSG00000073146	protein_coding	43.3773939	1.4206652	0.15457553	9.19075073	3.9011E-20	2.29E-19
GSDMB	ENSG00000073605	protein_coding	410.154027	1.87212816	0.16170996	11.5770739	5.3853E-31	5.5916E-30
ADAM11	ENSG00000073670	protein_coding	58.7221708	2.08352999	0.1509622	13.8016668	2.4903E-43	4.475E-42
ABCB11	ENSG00000073734	protein_coding	2.14901647	1.32124526	0.30396204	4.34674422	1.3817E-05	2.8369E-05
DHRS9	ENSG00000073737	protein_coding	107.570406	2.398538	0.16767535	14.3046546	2.0465E-46	4.1807E-45
CD5L	ENSG00000073754	protein_coding	146.881437	4.88517012	0.32408671	15.0736516	2.4139E-51	6.0238E-50
TBX21	ENSG00000073861	protein_coding	137.399553	2.45642947	0.1253748	19.5926897	1.7851E-85	1.5126E-83
NOTCH3	ENSG00000074181	protein_coding	18488.1816	1.32108671	0.11907651	11.0944355	1.335E-28	1.2348E-27
PPP2R2C	ENSG00000074211	protein_coding	178.387724	2.22418972	0.2879318	7.72471013	1.1211E-14	4.7118E-14
SNCB	ENSG00000074317	protein_coding	6.28114746	3.26437572	0.38279505	8.5277375	1.4924E-17	7.5435E-17
SCARF1	ENSG00000074660	protein_coding	1817.18053	1.5216515	0.09155021	16.6209509	4.9147E-62	1.7684E-60
MYDGF	ENSG00000074842	protein_coding	4940.40802	1.23959581	0.07776107	15.9410843	3.286E-57	1.0123E-55
GTSE1	ENSG00000075218	protein_coding	197.21647	2.32876106	0.13927129	16.7210414	9.2096E-63	3.3992E-61
GRAMD4	ENSG00000075240	protein_coding	4863.89424	1.75627301	0.1152685	15.2363653	2.0285E-52	5.2863E-51
ADD2	ENSG00000075340	protein_coding	229.751652	2.70511274	0.20767732	13.0255567	8.756E-39	1.2965E-37
FSCN1	ENSG00000075618	protein_coding	5363.61505	1.54864127	0.10964088	14.1246707	2.6765E-45	5.2055E-44

WDR62	ENSG00000075702	protein_coding	162.281208	1.50400965	0.12325421	12.2025008	3.0142E-34	3.6583E-33
SEC31B	ENSG00000075826	protein_coding	439.226853	1.63170361	0.15972199	10.2158985	1.6831E-24	1.2531E-23
ARHGAP15	ENSG00000075884	protein_coding	867.777489	1.60840921	0.09843123	16.3404353	5.0888E-60	1.7241E-58
TUBA3D	ENSG00000075886	protein_coding	665.385234	6.49483248	0.31505881	20.6146669	2.0271E-94	2.4649E-92
PAG1	ENSG00000076641	protein_coding	2800.77901	1.723139	0.08869984	19.4266311	4.5955E-84	3.6701E-82
ICAM3	ENSG00000076662	protein_coding	155.413263	1.96569747	0.12974692	15.1502435	7.5486E-52	1.9299E-50
MCAM	ENSG00000076706	protein_coding	24428.636	2.0580988	0.10845235	18.9769862	2.6433E-80	1.8642E-78
ARHGEF1	ENSG00000076928	protein_coding	4881.34731	1.16633215	0.07755992	15.0378206	4.1499E-51	1.025E-49
NMRK2	ENSG00000077009	protein_coding	86.0917318	1.64320621	0.45901966	3.57981664	0.00034384	0.00061045
DGKD	ENSG00000077044	protein_coding	2703.85083	2.0977915	0.09124259	22.9913636	5.687E-117	1.417E-114
ACTL6B	ENSG00000077080	protein_coding	2.33158063	3.14204295	0.42186926	7.44790694	9.4833E-14	3.7463E-13
NFKB2	ENSG00000077150	protein_coding	4295.38599	1.21016561	0.08965437	13.4981212	1.6042E-41	2.6692E-40
IL4R	ENSG00000077238	protein_coding	4244.60925	1.45577389	0.0714913	20.3629523	3.5644E-92	3.9564E-90
EXOSC5	ENSG00000077348	protein_coding	1166.63432	1.31165911	0.09011193	14.5558881	5.3585E-48	1.1675E-46
APBB1IP	ENSG00000077420	protein_coding	3178.2392	2.41073486	0.13137433	18.3501216	3.2937E-75	1.9259E-73
TYR	ENSG00000077498	protein_coding	5.42674507	1.90404119	0.25955573	7.33577012	2.2045E-13	8.48E-13
ACTN2	ENSG00000077522	protein_coding	137.251349	1.51242779	0.2354432	6.42374809	1.3296E-10	4.1927E-10
FKBP6	ENSG00000077800	protein_coding	2.18290502	1.68479325	0.30669078	5.49345904	3.9414E-08	1.0184E-07
SMC1B	ENSG00000077935	protein_coding	20.4410083	2.29880826	0.18913706	12.1541928	5.4497E-34	6.5398E-33
CST7	ENSG00000077984	protein_coding	897.787572	3.11106799	0.16367026	19.008145	1.4603E-80	1.0378E-78
LAMP3	ENSG00000078081	protein_coding	347.462586	1.17091407	0.12372322	9.46398026	2.9644E-21	1.8544E-20
FAP	ENSG00000078098	protein_coding	377.038657	1.18472426	0.17845361	6.63883597	3.1617E-11	1.0461E-10
ADCY2	ENSG00000078295	protein_coding	313.013745	2.56789818	0.2405653	10.6744328	1.3407E-26	1.1219E-25
EDN1	ENSG00000078401	protein_coding	3577.02694	1.75901874	0.14705793	11.9614004	5.6599E-33	6.4767E-32
ADCYAP1R1	ENSG00000078549	protein_coding	30.9959891	1.80119109	0.22376624	8.04943189	8.3179E-16	3.7619E-15

FGF20	ENSG00000078579	protein_coding	12.067395	2.10064684	0.19414878	10.8197788	2.7745E-27	2.4051E-26
P2RY10	ENSG00000078589	protein_coding	151.333325	1.92958462	0.17198346	11.2195938	3.2677E-29	3.1318E-28
MYH7B	ENSG00000078814	protein_coding	166.041432	1.1582057	0.14583819	7.94171739	1.994E-15	8.8171E-15
BPIFB2	ENSG00000078898	protein_coding	1.02914008	2.42274894	0.62630359	3.86832997	0.00010958	0.00020548
TP73	ENSG00000078900	protein_coding	85.7009171	3.05124552	0.17807733	17.1343847	8.2218E-66	3.3802E-64
SLC1A3	ENSG00000079215	protein_coding	1259.23793	2.71718809	0.1457736	18.6397823	1.5286E-77	9.8142E-76
SP140	ENSG00000079263	protein_coding	251.54574	1.78535462	0.14527056	12.2898582	1.0269E-34	1.2757E-33
DUSP13	ENSG00000079393	protein_coding	3.29432322	1.0175409	0.36671568	2.77474065	0.00552457	0.00855781
SCGN	ENSG00000079689	protein_coding	2194.83973	4.6572675	0.25938829	17.95481	4.4008E-72	2.2795E-70
PTPRH	ENSG00000080031	protein_coding	173.435452	2.5422016	0.29534726	8.60750028	7.4671E-18	3.8422E-17
EPHA6	ENSG00000080224	protein_coding	117.954101	1.38168064	0.23266183	5.93857883	2.875E-09	8.1728E-09
COL5A3	ENSG00000080573	protein_coding	2175.61239	3.53285032	0.13053133	27.0651516	2.533E-161	2.37E-158
CPB2	ENSG00000080618	protein_coding	5.92328159	1.41543517	0.26857195	5.27022704	1.3626E-07	3.3653E-07
NDC80	ENSG00000080986	protein_coding	244.823977	2.03474554	0.11468361	17.7422523	1.9784E-70	9.6381E-69
CXCL2	ENSG00000081041	protein_coding	623.401515	1.62521337	0.20381096	7.9741217	1.5347E-15	6.8395E-15
CDH7	ENSG00000081138	protein_coding	13.1646785	1.46658648	0.380557	3.85378928	0.0001163	0.0002175
MEF2C	ENSG00000081189	protein_coding	5230.59968	1.28101498	0.09381368	13.6548848	1.8879E-42	3.2657E-41
PTPRC	ENSG00000081237	protein_coding	4718.4012	1.82679983	0.13293882	13.7416584	5.7152E-43	1.0151E-41
PKP1	ENSG00000081277	protein_coding	562.830313	2.09642037	0.26461526	7.92252272	2.3274E-15	1.0242E-14
IL12RB2	ENSG00000081985	protein_coding	245.382046	2.61056215	0.26368621	9.90026044	4.1519E-23	2.8612E-22
FYB1	ENSG00000082074	protein_coding	2743.44354	1.67795292	0.13356095	12.5631997	3.3645E-36	4.4769E-35
MPP4	ENSG00000082126	protein_coding	11.3418393	1.16471866	0.18482647	6.30168763	2.9442E-10	9.0545E-10
SEMA5B	ENSG00000082684	protein_coding	8738.45466	3.88108476	0.13774937	28.1749727	1.185E-174	1.646E-171
PLOD1	ENSG00000083444	protein_coding	16363.0827	1.32109516	0.10006142	13.2028426	8.4486E-40	1.3031E-38
P2RX5	ENSG00000083454	protein_coding	43.5454069	1.89618471	0.19307648	9.82089953	9.1523E-23	6.1954E-22

EPYC	ENSG00000083782	protein_coding	7.97990857	3.65750846	0.34811082	10.506736	8.0429E-26	6.4491E-25
HAL	ENSG00000084110	protein_coding	32.6691983	1.51614535	0.15456831	9.80890144	1.0308E-22	6.9636E-22
NKAIN1	ENSG00000084628	protein_coding	142.499759	4.01190582	0.21764253	18.4334646	7.08E-76	4.2593E-74
APOB	ENSG00000084674	protein_coding	1521.67247	4.18798297	0.2903834	14.4222534	3.749E-47	7.887E-46
GCKR	ENSG00000084734	protein_coding	21.6096042	1.69011548	0.29878678	5.65659386	1.5441E-08	4.1323E-08
FCN1	ENSG00000085265	protein_coding	681.337066	1.05678832	0.14158219	7.46413324	8.385E-14	3.3239E-13
PILRA	ENSG00000085514	protein_coding	676.962884	1.63615391	0.10343322	15.8184564	2.3211E-56	6.8914E-55
TTC39A	ENSG00000085831	protein_coding	2705.0351	1.1816565	0.1263615	9.35139671	8.6498E-21	5.2665E-20
RAD54L	ENSG00000085999	protein_coding	163.235887	2.48943647	0.15451242	16.1115618	2.1161E-58	6.7978E-57
NME8	ENSG00000086288	protein_coding	36.8618986	2.79591905	0.15038562	18.5916648	3.7534E-77	2.3831E-75
SNX10	ENSG00000086300	protein_coding	6376.34407	1.24278861	0.11008546	11.2893076	1.482E-29	1.4421E-28
PPEF1	ENSG00000086717	protein_coding	48.5734846	2.52399049	0.17833507	14.1530798	1.7875E-45	3.5063E-44
LAT2	ENSG00000086730	protein_coding	927.060849	2.14632695	0.10304624	20.8287748	2.3746E-96	3.1643E-94
ACHE	ENSG00000087085	protein_coding	317.604674	1.36620268	0.19327502	7.06869786	1.5639E-12	5.6831E-12
BAX	ENSG00000087088	protein_coding	2830.80104	1.22620226	0.06699574	18.3026899	7.8762E-75	4.5473E-73
ADAMTS2	ENSG00000087116	protein_coding	1705.52804	1.91079285	0.13665528	13.9825758	1.9915E-44	3.7461E-43
CETP	ENSG00000087237	protein_coding	618.891435	1.23429534	0.12388689	9.9630826	2.211E-23	1.5457E-22
MT3	ENSG00000087250	protein_coding	898.511365	3.33572355	0.29300885	11.3843781	5.0024E-30	4.9539E-29
SH3BP2	ENSG00000087266	protein_coding	9739.31224	1.66548101	0.08369187	19.9001533	4.0569E-88	3.8109E-86
PTHLH	ENSG00000087494	protein_coding	2540.06488	6.38818352	0.27720906	23.0446423	1.665E-117	4.193E-115
CASS4	ENSG00000087589	protein_coding	329.071571	1.22398216	0.1255257	9.75084947	1.8293E-22	1.2173E-21
RFX2	ENSG00000087903	protein_coding	789.88242	1.47358887	0.10613946	13.8835164	7.9734E-44	1.4638E-42
GP6	ENSG00000088053	protein_coding	44.8214816	2.24798778	0.18180418	12.3648847	4.0479E-35	5.1105E-34
TPX2	ENSG00000088325	protein_coding	820.722175	2.49348231	0.14510833	17.1835915	3.524E-66	1.4713E-64
SMOX	ENSG00000088826	protein_coding	1143.69561	1.11253356	0.10529268	10.5661056	4.279E-26	3.4797E-25

SIGLEC1	ENSG00000088827	protein_coding	1336.77929	2.52900831	0.14641241	17.2731825	7.4896E-67	3.2057E-65
SIRPG	ENSG00000089012	protein_coding	249.965253	3.36337185	0.19320524	17.4082852	7.139E-68	3.1711E-66
P2RX7	ENSG00000089041	protein_coding	1052.17821	2.65953784	0.10456261	25.4348829	1.038E-142	5.406E-140
OAS1	ENSG00000089127	protein_coding	3545.52492	1.42908451	0.09449464	15.1234457	1.1345E-51	2.8685E-50
RPLP0	ENSG00000089157	protein_coding	73993.0042	1.04957549	0.08534922	12.2974233	9.351E-35	1.1633E-33
RPH3A	ENSG00000089169	protein_coding	22.9171079	1.39737577	0.17997453	7.76429738	8.2099E-15	3.4802E-14
TBX5	ENSG00000089225	protein_coding	22.3759449	6.18824303	0.44965	13.7623551	4.2931E-43	7.6457E-42
FXYD5	ENSG00000089327	protein_coding	4752.89619	1.76379692	0.10655671	16.5526593	1.5318E-61	5.4225E-60
GRAMD1A	ENSG00000089351	protein_coding	3931.2165	1.30247015	0.06929521	18.7959623	8.1489E-79	5.4693E-77
KCNH4	ENSG00000089558	protein_coding	25.9342393	1.57371934	0.14606437	10.7741495	4.5598E-27	3.9099E-26
GMIP	ENSG00000089639	protein_coding	1126.42986	1.59386633	0.08788101	18.1366418	1.6374E-73	8.9998E-72
BIRC5	ENSG00000089685	protein_coding	438.479813	2.47046358	0.1575633	15.6791818	2.0994E-55	6.0528E-54
LAG3	ENSG00000089692	protein_coding	587.934556	3.55128614	0.20705776	17.1511856	6.1583E-66	2.5525E-64
ARHGAP4	ENSG00000089820	protein_coding	2357.71321	1.10250928	0.10075305	10.9426888	7.2031E-28	6.4304E-27
RGS1	ENSG00000090104	protein_coding	6840.23492	3.09383414	0.15230647	20.3132158	9.8256E-92	1.0573E-89
ICAM1	ENSG00000090339	protein_coding	5922.16112	1.3961255	0.11102538	12.5748316	2.9042E-36	3.8802E-35
LYZ	ENSG00000090382	protein_coding	10375.6396	2.43047554	0.16759444	14.5021253	1.1746E-47	2.5219E-46
SI	ENSG00000090402	protein_coding	0.90620832	2.24416057	0.48334303	4.64299767	3.4339E-06	7.4724E-06
KIF4A	ENSG00000090889	protein_coding	289.730145	2.2091063	0.13387812	16.5008762	3.6161E-61	1.2712E-59
PLEKHG2	ENSG00000090924	protein_coding	2991.83343	1.73699969	0.096033	18.0875299	3.996E-73	2.1627E-71
DLL3	ENSG00000090932	protein_coding	5.41750528	1.6259284	0.30280111	5.36962505	7.8901E-08	1.9918E-07
DTX2	ENSG00000091073	protein_coding	780.069475	1.30269095	0.07461376	17.4591238	2.934E-68	1.3225E-66
NLRC4	ENSG00000091106	protein_coding	254.118927	1.77716966	0.10460841	16.9887833	9.943E-65	3.96E-63
SEL1L3	ENSG00000091490	protein_coding	8784.60812	1.65574074	0.10708695	15.4616488	6.2966E-54	1.7253E-52
TF	ENSG00000091513	protein_coding	721.891184	3.98495218	0.3138594	12.6966156	6.1743E-37	8.5021E-36

NLRP1	ENSG00000091592	protein_coding	1793.29236	1.18183939	0.10144068	11.6505473	2.2799E-31	2.4137E-30
ORC6	ENSG00000091651	protein_coding	120.866795	1.34511638	0.12453659	10.8009732	3.4057E-27	2.9368E-26
RGS17	ENSG00000091844	protein_coding	105.180787	1.53498942	0.17713638	8.66557978	4.4922E-18	2.3451E-17
ANGPT2	ENSG00000091879	protein_coding	6720.30508	3.10452268	0.13972383	22.2189922	2.251E-109	4.69E-107
CD200	ENSG00000091972	protein_coding	2276.02657	1.65916714	0.09641175	17.2091789	2.2662E-66	9.5308E-65
CEBPE	ENSG00000092067	protein_coding	7.07986216	1.51003252	0.15212981	9.92594734	3.2104E-23	2.2255E-22
RPGRIP1	ENSG00000092200	protein_coding	49.1629187	1.02841505	0.09283654	11.0776971	1.6096E-28	1.4807E-27
SEMA6A	ENSG00000092421	protein_coding	6218.36419	1.897041	0.11564431	16.4041016	1.7875E-60	6.1564E-59
TBX15	ENSG00000092607	protein_coding	360.694589	3.11618489	0.19016746	16.3865304	2.3869E-60	8.1898E-59
CLSPN	ENSG00000092853	protein_coding	125.423988	1.61503791	0.12282655	13.1489316	1.726E-39	2.6426E-38
UNC13D	ENSG00000092929	protein_coding	827.840359	2.16472628	0.11940365	18.1294823	1.8651E-73	1.0215E-71
CDC45	ENSG00000093009	protein_coding	144.951435	2.71458257	0.1323068	20.5173322	1.5076E-93	1.7766E-91
VNN3	ENSG00000093134	protein_coding	19.2323196	2.45143554	0.21872606	11.2077891	3.734E-29	3.5646E-28
CDC6	ENSG00000094804	protein_coding	289.048925	1.98866151	0.1252681	15.8752425	9.4047E-57	2.8344E-55
SH2D3C	ENSG00000095370	protein_coding	2670.14029	1.0861156	0.09972253	10.8913764	1.2671E-27	1.1166E-26
PDE6C	ENSG00000095464	protein_coding	29.6449487	1.72045057	0.14769445	11.6487151	2.3294E-31	2.4656E-30
CYP26A1	ENSG00000095596	protein_coding	13.710049	1.87944555	0.26864725	6.99596058	2.6345E-12	9.4121E-12
MYO3A	ENSG00000095777	protein_coding	610.680487	3.29627707	0.15297313	21.5480784	5.519E-103	9.37E-101
TPSD1	ENSG00000095917	protein_coding	51.7927683	1.2631356	0.25692948	4.91627346	8.8207E-07	2.0295E-06
TREM2	ENSG00000095970	protein_coding	1345.39075	4.24795857	0.14733807	28.8313715	8.677E-183	1.53E-179
KCNK16	ENSG00000095981	protein_coding	0.76046077	1.98295848	0.44406856	4.46543319	7.9907E-06	1.6784E-05
NCR2	ENSG00000096264	protein_coding	0.95394652	1.96988026	0.40599008	4.85204036	1.222E-06	2.773E-06
MLN	ENSG00000096395	protein_coding	1.07327285	2.28942057	0.34553628	6.6257024	3.456E-11	1.1399E-10
IL12RB1	ENSG00000096996	protein_coding	383.070042	2.58226326	0.1252849	20.6111294	2.1808E-94	2.6388E-92
SCD	ENSG00000099194	protein_coding	25302.8604	2.99635648	0.13617567	22.0036113	2.659E-107	5.188E-105

NRP1	ENSG00000099250	protein_coding	25386.5737	1.05812535	0.08874524	11.9231781	8.9623E-33	1.0129E-31
MYO9B	ENSG00000099331	protein_coding	5017.09506	1.15867663	0.06314405	18.349736	3.3172E-75	1.9371E-73
STX1B	ENSG00000099365	protein_coding	198.6268	1.72232568	0.13904634	12.3867029	3.0847E-35	3.9224E-34
HSD3B7	ENSG00000099377	protein_coding	4249.08206	2.22275476	0.13794803	16.1129862	2.0679E-58	6.6476E-57
MAGEB2	ENSG00000099399	protein_coding	1.75994832	3.10889221	0.76257409	4.07683956	4.5652E-05	8.9128E-05
EFNA2	ENSG00000099617	protein_coding	7.77924202	1.72202723	0.3247079	5.30331171	1.1372E-07	2.8283E-07
PRKY	ENSG00000099725	protein_coding	322.864657	1.40171845	0.3260795	4.29870158	1.718E-05	3.4948E-05
CDHR5	ENSG00000099834	protein_coding	9793.39983	1.02302097	0.2067064	4.94914995	7.4538E-07	1.7268E-06
SERPIND1	ENSG00000099937	protein_coding	28.6802338	1.5741033	0.26703807	5.89467746	3.7541E-09	1.0568E-08
MMP11	ENSG00000099953	protein_coding	1010.16694	2.01859894	0.13005254	15.5214117	2.4852E-54	6.892E-53
DERL3	ENSG00000099958	protein_coding	601.728683	2.16068633	0.18503663	11.6770737	1.6694E-31	1.7793E-30
OSM	ENSG00000099985	protein_coding	216.723985	1.87765398	0.1789249	10.4940899	9.196E-26	7.3556E-25
SEC14L3	ENSG00000100012	protein_coding	4.91166069	3.83501204	0.34738118	11.0397806	2.4563E-28	2.2417E-27
PPM1F	ENSG00000100034	protein_coding	3936.34206	1.02447942	0.07337696	13.9618672	2.6636E-44	4.9818E-43
CYTH4	ENSG00000100055	protein_coding	1424.91813	1.94935775	0.10970345	17.7693392	1.2212E-70	6.0325E-69
MFNG	ENSG00000100060	protein_coding	1376.78036	1.19788429	0.08626873	13.8854977	7.756E-44	1.425E-42
SH3BP1	ENSG00000100092	protein_coding	687.006709	1.64465368	0.10257142	16.0342294	7.3692E-58	2.3297E-56
SEZ6L	ENSG00000100095	protein_coding	28.2292941	2.0093329	0.23837539	8.42928005	3.478E-17	1.7182E-16
LGALS1	ENSG00000100097	protein_coding	15118.3378	2.14282482	0.12400127	17.280668	6.5782E-67	2.8235E-65
CRYBB1	ENSG00000100122	protein_coding	41.8826468	1.46742661	0.12263529	11.9657775	5.3692E-33	6.1517E-32
POLR2F	ENSG00000100142	protein_coding	17.7347064	1.6727557	0.13749434	12.1659965	4.7165E-34	5.6778E-33
SLC16A8	ENSG00000100156	protein_coding	47.9468066	2.05725961	0.15613223	13.1763931	1.1999E-39	1.8471E-38
CENPM	ENSG00000100162	protein_coding	169.605168	2.20436678	0.13193158	16.7084088	1.1383E-62	4.188E-61
SLC5A1	ENSG00000100170	protein_coding	3024.174	2.09931179	0.25659244	8.18150276	2.8033E-16	1.3061E-15
SLC5A4	ENSG00000100191	protein_coding	109.20017	1.77111109	0.14536819	12.1836221	3.8002E-34	4.594E-33

CYP2D6	ENSG00000100197	protein_coding	58.5985983	1.23633554	0.16181459	7.64044518	2.1647E-14	8.9182E-14
RASL10A	ENSG00000100276	protein_coding	44.1003517	2.03734134	0.14748456	13.8139299	2.1005E-43	3.7924E-42
CHKB	ENSG00000100288	protein_coding	297.838255	1.7096941	0.13566958	12.6018971	2.0613E-36	2.7734E-35
HMOX1	ENSG00000100292	protein_coding	16370.0316	2.31566541	0.13315393	17.3908909	9.6719E-68	4.2632E-66
MCM5	ENSG00000100297	protein_coding	2796.52422	1.05529782	0.06276273	16.8140838	1.9244E-63	7.315E-62
APOBEC3H	ENSG00000100298	protein_coding	103.842056	2.9020562	0.14359044	20.2106509	7.8901E-91	8.1461E-89
RASD2	ENSG00000100302	protein_coding	809.552792	2.87757691	0.13657378	21.0697615	1.5069E-98	2.1386E-96
ACR	ENSG00000100312	protein_coding	33.7840208	1.2800546	0.14310125	8.94509699	3.7162E-19	2.0667E-18
CABP7	ENSG00000100314	protein_coding	15.302264	1.09352456	0.13963603	7.83124945	4.8305E-15	2.0804E-14
PNPLA5	ENSG00000100341	protein_coding	2.11594653	2.82430401	0.50090887	5.63835893	1.7168E-08	4.5753E-08
APOL1	ENSG00000100342	protein_coding	20238.6373	2.05353305	0.17675567	11.6179192	3.3418E-31	3.5079E-30
CACNA1I	ENSG00000100346	protein_coding	21.3041723	1.80017815	0.20430561	8.81120282	1.2381E-18	6.6827E-18
GRAP2	ENSG00000100351	protein_coding	218.068848	1.67587036	0.11907595	14.0739618	5.4905E-45	1.054E-43
NCF4	ENSG00000100365	protein_coding	654.311607	2.00858042	0.11482642	17.4923191	1.6395E-68	7.4559E-67
CSF2RB	ENSG00000100368	protein_coding	931.015761	1.33822118	0.10353389	12.9254412	3.2344E-38	4.6935E-37
FAM118A	ENSG00000100376	protein_coding	911.049392	1.07796357	0.10424278	10.3408945	4.6022E-25	3.5297E-24
IL2RB	ENSG00000100385	protein_coding	1506.39772	2.99256481	0.13302544	22.4961847	4.523E-112	1.011E-109
MLC1	ENSG00000100427	protein_coding	55.9550272	2.19388294	0.13584729	16.1496263	1.1425E-58	3.704E-57
HDAC10	ENSG00000100429	protein_coding	467.110183	1.50850621	0.11076857	13.6185399	3.1072E-42	5.3168E-41
GZMH	ENSG00000100450	protein_coding	390.269142	2.97757097	0.15036587	19.8021729	2.8513E-87	2.5831E-85
GZMB	ENSG00000100453	protein_coding	335.366222	2.55670925	0.14725186	17.362832	1.5775E-67	6.9067E-66
POLE2	ENSG00000100479	protein_coding	118.814919	1.33952873	0.09421077	14.2184248	7.042E-46	1.4029E-44
PYGL	ENSG00000100504	protein_coding	4051.47157	1.99938417	0.0979789	20.4062735	1.4708E-92	1.6607E-90
TRIM9	ENSG00000100505	protein_coding	921.541685	2.83164201	0.14006725	20.216303	7.0363E-91	7.281E-89
CDKN3	ENSG00000100526	protein_coding	148.332618	1.89026017	0.13711171	13.7862778	3.0826E-43	5.5199E-42

LRRC74A	ENSG00000100565	protein_coding	4.32474312	1.29217225	0.20116954	6.4232998	1.3335E-10	4.2037E-10
RIN3	ENSG00000100599	protein_coding	2175.78573	1.40556909	0.08090607	17.3728507	1.3248E-67	5.8114E-66
ASB2	ENSG00000100628	protein_coding	225.740832	1.43341849	0.15733124	9.11083224	8.1752E-20	4.7235E-19
SLC10A1	ENSG00000100652	protein_coding	7.79858541	1.80771621	0.25145845	7.18892612	6.5303E-13	2.433E-12
MMP9	ENSG00000100985	protein_coding	1368.06001	3.75915505	0.23700824	15.8608624	1.1826E-56	3.5478E-55
VSX1	ENSG00000100987	protein_coding	28.2994706	3.20587328	0.27921467	11.481751	1.6295E-30	1.6544E-29
PROCR	ENSG00000101000	protein_coding	2086.87579	1.58535062	0.10541152	15.0396336	4.0378E-51	9.9836E-50
CD40	ENSG00000101017	protein_coding	2993.72492	1.82077802	0.07704963	23.6312356	1.841E-123	5.701E-121
MYBL2	ENSG00000101057	protein_coding	378.690385	3.4014482	0.18144409	18.7465358	2.0662E-78	1.3687E-76
SLA2	ENSG00000101082	protein_coding	301.577281	2.90305261	0.14865981	19.5281607	6.3278E-85	5.274E-83
NFATC2	ENSG00000101096	protein_coding	1543.73255	1.16193927	0.09334131	12.4482849	1.4288E-35	1.8503E-34
PABPC1L	ENSG00000101104	protein_coding	1113.2969	2.21273393	0.17277117	12.80731	1.4922E-37	2.1112E-36
SALL4	ENSG00000101115	protein_coding	39.0490131	1.85163298	0.2421217	7.64752997	2.0488E-14	8.4519E-14
CTSZ	ENSG00000101160	protein_coding	18562.6782	1.26793506	0.09195897	13.7880519	3.0077E-43	5.3879E-42
SLC17A9	ENSG00000101194	protein_coding	596.625168	3.34533845	0.18446806	18.1350556	1.6853E-73	9.2521E-72
BIRC7	ENSG00000101197	protein_coding	749.862373	7.04918428	0.30556787	23.0691283	9.455E-118	2.421E-115
EEF1A2	ENSG00000101210	protein_coding	469.834303	1.30437488	0.30952702	4.21409051	2.5079E-05	5.0266E-05
PTK6	ENSG00000101213	protein_coding	150.656368	1.02621257	0.20203225	5.07944936	3.7853E-07	9.0048E-07
TRIB3	ENSG00000101255	protein_coding	3341.18699	3.12372442	0.16546006	18.8790238	1.6969E-79	1.1593E-77
RASSF2	ENSG00000101265	protein_coding	3013.17069	2.1809585	0.10774069	20.2426629	4.1228E-91	4.3051E-89
RSPO4	ENSG00000101282	protein_coding	37.8135079	2.19581061	0.25319932	8.67226109	4.2362E-18	2.2153E-17
SIRPB1	ENSG00000101307	protein_coding	351.277841	2.65832876	0.1669276	15.9250402	4.2474E-57	1.2989E-55
PLCB4	ENSG00000101333	protein_coding	1625.16745	1.0855442	0.15517967	6.99540195	2.645E-12	9.4482E-12
HCK	ENSG00000101336	protein_coding	1385.97274	2.03496074	0.10854487	18.7476463	2.0235E-78	1.3443E-76
SAMHD1	ENSG00000101347	protein_coding	6778.51896	1.40993019	0.09891078	14.2545655	4.1991E-46	8.4425E-45

E2F1	ENSG00000101412	protein_coding	360.204677	2.18185297	0.1119208	19.4946156	1.2197E-84	1.0038E-82
ASIP	ENSG00000101440	protein_coding	23.9532363	1.56487995	0.22310012	7.01424974	2.3119E-12	8.2873E-12
CST4	ENSG00000101441	protein_coding	1.07664769	1.62198693	0.60777929	2.66871041	0.00761431	0.01158395
CELF4	ENSG00000101489	protein_coding	86.6975569	1.03945022	0.16971855	6.12455265	9.0939E-10	2.6947E-09
H2BW2	ENSG00000101812	protein_coding	0.45461328	1.14023697	0.40430246	2.8202573	0.00479852	0.00748575
VSIG1	ENSG00000101842	protein_coding	221.42653	3.94731155	0.18396011	21.4574319	3.892E-102	6.327E-100
RHOXF1	ENSG00000101883	protein_coding	17.9776885	1.64593858	0.18930888	8.69446027	3.4848E-18	1.8308E-17
GUCY2F	ENSG00000101890	protein_coding	1.11402749	1.92727746	0.37563048	5.13078036	2.8854E-07	6.9361E-07
TLR8	ENSG00000101916	protein_coding	399.10755	2.11530875	0.15955285	13.2577307	4.0703E-40	6.3724E-39
ABCD1	ENSG00000101986	protein_coding	1547.89481	1.03045028	0.0746629	13.80137	2.5005E-43	4.4917E-42
CACNA1F	ENSG00000102001	protein_coding	107.782903	2.26506751	0.17966703	12.6070294	1.9314E-36	2.5994E-35
PLP2	ENSG00000102007	protein_coding	4968.39791	1.14182794	0.12469987	9.15660893	5.3554E-20	3.1277E-19
BMX	ENSG00000102010	protein_coding	161.223404	1.50983422	0.13899511	10.8624987	1.7392E-27	1.5189E-26
PIM2	ENSG00000102096	protein_coding	1688.69957	1.2602791	0.13647838	9.23427659	2.6004E-20	1.5415E-19
TAZ	ENSG00000102125	protein_coding	1381.53879	1.09844672	0.08187654	13.4158912	4.8803E-41	7.9502E-40
RAB40AL	ENSG00000102128	protein_coding	4.18858899	1.47673931	0.21532854	6.85807528	6.9794E-12	2.4196E-11
GATA1	ENSG00000102145	protein_coding	7.99661077	1.61940593	0.19798724	8.17934489	2.8539E-16	1.3291E-15
PHEX	ENSG00000102174	protein_coding	133.987067	1.2862222	0.15904662	8.08707661	6.1114E-16	2.7856E-15
BRS3	ENSG00000102239	protein_coding	11.9245676	1.94287032	0.40253444	4.82659396	1.3889E-06	3.1353E-06
CD40LG	ENSG00000102245	protein_coding	102.750731	1.47877624	0.14650858	10.0934446	5.906E-24	4.2582E-23
TIMP1	ENSG00000102265	protein_coding	24668.6812	2.05932215	0.14786584	13.9269632	4.3444E-44	8.0661E-43
GABRE	ENSG00000102287	protein_coding	1476.74698	2.92693265	0.18027594	16.2358475	2.8134E-59	9.3117E-58
KLF8	ENSG00000102349	protein_coding	1206.84426	1.19166123	0.10382062	11.4780788	1.7002E-30	1.725E-29
CENPI	ENSG00000102384	protein_coding	86.8669694	1.59767899	0.12249142	13.043191	6.9487E-39	1.0335E-37
RUBCNL	ENSG00000102445	protein_coding	237.769501	1.4886674	0.12097633	12.3054433	8.467E-35	1.055E-33

FGF14	ENSG00000102466	protein_coding	238.434151	1.13954752	0.18639558	6.11359725	9.741E-10	2.8796E-09
TNFSF13B	ENSG00000102524	protein_coding	917.266012	2.50593289	0.14550366	17.2224736	1.8012E-66	7.5961E-65
FLT1	ENSG00000102755	protein_coding	29372.9655	1.86902196	0.12402825	15.0693243	2.5774E-51	6.4211E-50
RGCC	ENSG00000102760	protein_coding	4686.05345	1.58924118	0.11373813	13.9728094	2.2843E-44	4.2846E-43
MSLN	ENSG00000102854	protein_coding	998.905368	1.30013716	0.32367601	4.01678572	5.8997E-05	0.00011369
TRADD	ENSG00000102871	protein_coding	2243.14501	1.44155991	0.06933218	20.7920761	5.1054E-96	6.6299E-94
HSF4	ENSG00000102878	protein_coding	4187.55865	5.69423433	0.18961103	30.0311338	3.851E-198	8.405E-195
CORO1A	ENSG00000102879	protein_coding	3728.55399	2.41871089	0.12669788	19.0903819	3.0354E-81	2.2122E-79
CENPT	ENSG00000102901	protein_coding	1529.62939	1.16374467	0.0937429	12.4142172	2.1882E-35	2.8105E-34
CBLN1	ENSG00000102924	protein_coding	58.8607334	2.08794348	0.24978365	8.35900776	6.3248E-17	3.0726E-16
CCL22	ENSG00000102962	protein_coding	107.855496	1.0811095	0.17245003	6.26911753	3.631E-10	1.1099E-09
NME3	ENSG00000103024	protein_coding	2190.25518	1.03477836	0.10857704	9.53036095	1.5674E-21	9.9474E-21
FOXF1	ENSG00000103241	protein_coding	442.282181	1.36215368	0.12840567	10.6082053	2.7294E-26	2.2419E-25
HAGHL	ENSG00000103253	protein_coding	385.812995	1.54076287	0.21740726	7.08698919	1.3706E-12	5.0004E-12
MEFV	ENSG00000103313	protein_coding	113.631613	2.24519635	0.14002686	16.0340405	7.3916E-58	2.3352E-56
CAPN15	ENSG00000103326	protein_coding	2914.06153	1.29668699	0.0793081	16.3499942	4.3502E-60	1.4805E-58
AQP8	ENSG00000103375	protein_coding	5.01262504	2.40908476	0.25157255	9.57610349	1.0078E-21	6.4674E-21
CORO7-PAM16	ENSG00000103426	protein_coding	2.76945713	1.25875677	0.18556147	6.7835028	1.173E-11	3.9945E-11
PYCARD	ENSG00000103490	protein_coding	900.036475	2.06483459	0.13266789	15.5639368	1.2798E-54	3.6014E-53
STX4	ENSG00000103496	protein_coding	3255.04833	1.16302893	0.06371071	18.2548419	1.8936E-74	1.0783E-72
IL21R	ENSG00000103522	protein_coding	317.556667	2.60132284	0.14564789	17.8603535	2.4011E-71	1.2189E-69
AQP9	ENSG00000103569	protein_coding	751.36226	3.37447643	0.24996379	13.4998609	1.5667E-41	2.6088E-40
RASL12	ENSG00000103710	protein_coding	1039.33618	1.23492713	0.11438946	10.7958118	3.6026E-27	3.1031E-26
ACSBG1	ENSG00000103740	protein_coding	40.7254459	1.57523404	0.2024401	7.78123513	7.182E-15	3.0558E-14
IGDCC4	ENSG00000103742	protein_coding	382.868078	1.41338796	0.25075036	5.63663383	1.7341E-08	4.6192E-08

CD276	ENSG00000103855	protein_coding	4166.01737	1.19856975	0.07216234	16.609352	5.9634E-62	2.1374E-60
TGM5	ENSG00000104055	protein_coding	16.185162	2.46978455	0.30668049	8.05328228	8.0603E-16	3.6493E-15
SCG3	ENSG00000104112	protein_coding	36.5766243	1.65629726	0.2702816	6.12804295	8.8967E-10	2.638E-09
OIP5	ENSG00000104147	protein_coding	56.3365854	1.5647204	0.10442228	14.984545	9.2658E-51	2.2617E-49
PDGFRL	ENSG00000104213	protein_coding	226.814945	1.12916809	0.1968206	5.73704221	9.6344E-09	2.62E-08
EYA1	ENSG00000104313	protein_coding	99.296055	1.5004779	0.28193478	5.32207457	1.0259E-07	2.562E-07
TRPA1	ENSG00000104321	protein_coding	377.849983	3.7401191	0.18538959	20.1743744	1.6443E-90	1.675E-88
NDRG1	ENSG00000104419	protein_coding	125433.602	1.32788318	0.09748434	13.6215026	2.9836E-42	5.1092E-41
IL7	ENSG00000104432	protein_coding	292.773269	1.16914191	0.0910174	12.8452572	9.1447E-38	1.3035E-36
STMN2	ENSG00000104435	protein_coding	36.6449665	3.77934456	0.36272056	10.4194385	2.0214E-25	1.5854E-24
ANXA13	ENSG00000104537	protein_coding	1215.43443	1.80506472	0.2323053	7.77022625	7.8346E-15	3.3242E-14
SLC39A14	ENSG00000104635	protein_coding	18981.0341	1.69648522	0.13897984	12.2066999	2.8627E-34	3.479E-33
KCNN4	ENSG00000104783	protein_coding	163.225535	1.55475553	0.15976704	9.73139124	2.2154E-22	1.4672E-21
MAP4K1	ENSG00000104814	protein_coding	561.383486	2.38326857	0.13697136	17.3997584	8.2852E-68	3.666E-66
TUBB4A	ENSG00000104833	protein_coding	615.978859	1.35454957	0.25012526	5.41548481	6.1123E-08	1.5568E-07
KCNA7	ENSG00000104848	protein_coding	3.46021855	1.98273528	0.28112209	7.05293292	1.7519E-12	6.3383E-12
RELB	ENSG00000104856	protein_coding	1463.33395	1.10759081	0.07683486	14.415213	4.1516E-47	8.7219E-46
CLASRP	ENSG00000104859	protein_coding	1561.86278	1.2013333	0.09630601	12.4741255	1.0333E-35	1.3446E-34
PPP1R13L	ENSG00000104881	protein_coding	2792.80522	1.59279935	0.09997619	15.9317876	3.813E-57	1.17E-55
CD37	ENSG00000104894	protein_coding	1586.54395	2.13005311	0.11447605	18.6069753	2.821E-77	1.7986E-75
AMH	ENSG00000104899	protein_coding	52.4904523	2.68516763	0.23163035	11.5924688	4.4997E-31	4.6933E-30
LYL1	ENSG00000104903	protein_coding	410.40352	1.08897925	0.10121469	10.7591025	5.369E-27	4.5841E-26
TRMT1	ENSG00000104907	protein_coding	1644.28488	1.16276159	0.07861464	14.7906499	1.6832E-49	3.8998E-48
RETN	ENSG00000104918	protein_coding	14.1276477	2.66319772	0.23721457	11.2269566	3.0066E-29	2.8864E-28
RSPH6A	ENSG00000104941	protein_coding	3.69963003	1.09114556	0.3430233	3.18096634	0.00146785	0.00243161

IL4I1	ENSG00000104951	protein_coding	907.230209	2.11173988	0.14455819	14.6082337	2.4889E-48	5.5064E-47
NOVA2	ENSG00000104967	protein_coding	915.860244	1.14919976	0.11012113	10.4357791	1.7021E-25	1.3411E-24
LILRB1	ENSG00000104972	protein_coding	847.061731	2.91089444	0.10962918	26.5521856	2.423E-155	2.057E-152
LILRA1	ENSG00000104974	protein_coding	175.771909	1.51195325	0.1094838	13.8098353	2.2234E-43	4.008E-42
ASF1B	ENSG00000105011	protein_coding	384.053516	2.15281262	0.1201151	17.9229138	7.8127E-72	4.015E-70
TNNT1	ENSG00000105048	protein_coding	85.0436298	4.72543186	0.32196643	14.6767844	9.0795E-49	2.0463E-47
RASAL3	ENSG00000105122	protein_coding	963.891651	2.66029099	0.11046249	24.083206	3.749E-128	1.332E-125
SLC1A6	ENSG00000105143	protein_coding	15.6166748	2.42900868	0.3704939	6.55613679	5.522E-11	1.7925E-10
EBI3	ENSG00000105246	protein_coding	330.169036	2.29344197	0.13136349	17.4587471	2.9535E-68	1.33E-66
OVOL3	ENSG00000105261	protein_coding	3.09932864	2.01573254	0.28086951	7.17675812	7.1384E-13	2.6533E-12
BBC3	ENSG00000105327	protein_coding	587.519549	1.70228175	0.09438333	18.0358311	1.0195E-72	5.4217E-71
TGFB1	ENSG00000105329	protein_coding	7569.05878	1.56719957	0.09450952	16.5824512	9.3343E-62	3.3273E-60
CEACAM4	ENSG00000105352	protein_coding	38.8443611	1.83151317	0.1631184	11.2281211	2.9672E-29	2.8492E-28
SIGLEC8	ENSG00000105366	protein_coding	457.480415	5.06469793	0.17655579	28.6861054	5.687E-181	9.655E-178
CD79A	ENSG00000105369	protein_coding	283.923747	1.40150495	0.23409642	5.98687045	2.1392E-09	6.15E-09
LIM2	ENSG00000105370	protein_coding	3.28501449	3.1880221	0.29611934	10.7660043	4.9815E-27	4.2612E-26
RPS19	ENSG00000105372	protein_coding	41314.5833	1.47621121	0.10056988	14.6784625	8.8576E-49	1.9982E-47
NKG7	ENSG00000105374	protein_coding	1628.58922	3.48008011	0.16607643	20.9546901	1.7003E-97	2.3337E-95
CD33	ENSG00000105383	protein_coding	345.076536	1.93196968	0.11147058	17.3316548	2.7141E-67	1.1838E-65
CRX	ENSG00000105392	protein_coding	5.76552535	2.90169289	0.344991	8.41092335	4.068E-17	2.0008E-16
SULT2A1	ENSG00000105398	protein_coding	3.69360105	3.01934878	0.46060004	6.55525075	5.5548E-11	1.8026E-10
ATP1A3	ENSG00000105409	protein_coding	116.568319	2.03961091	0.20331267	10.0318927	1.1038E-23	7.8473E-23
MEIS3	ENSG00000105419	protein_coding	412.414168	1.444658	0.13373913	10.8020593	3.3657E-27	2.9029E-26
ZNRF4	ENSG00000105428	protein_coding	0.65760545	1.5547887	0.66797657	2.32760963	0.01993284	0.02859731
GRIN2D	ENSG00000105464	protein_coding	106.446859	1.96932482	0.15420542	12.7707888	2.3872E-37	3.3481E-36

SYNGR4	ENSG00000105467	protein_coding	8.03255289	2.00046603	0.21943099	9.11660657	7.7513E-20	4.4887E-19
LIG1	ENSG00000105486	protein_coding	1542.01261	1.02915508	0.06298016	16.3409398	5.0469E-60	1.7112E-58
SIGLEC6	ENSG00000105492	protein_coding	20.5614162	1.06138202	0.17887976	5.93349423	2.9655E-09	8.4217E-09
PLA2G4C	ENSG00000105499	protein_coding	1270.20386	1.01465334	0.09222173	11.0023238	3.7241E-28	3.3712E-27
CABP5	ENSG00000105507	protein_coding	1.64698509	2.29475391	0.42765027	5.36595925	8.052E-08	2.0308E-07
HAS1	ENSG00000105509	protein_coding	13.9574252	1.17626544	0.25547147	4.60429274	4.1387E-06	8.9437E-06
THEG	ENSG00000105549	protein_coding	2.48816035	2.98196318	0.41010536	7.27121248	3.5628E-13	1.3498E-12
FGF21	ENSG00000105550	protein_coding	1.48798961	2.85300056	0.4813298	5.92733003	3.079E-09	8.7299E-09
LILRB5	ENSG00000105609	protein_coding	394.951801	1.70657585	0.14897277	11.4556224	2.2037E-30	2.2227E-29
KLF1	ENSG00000105610	protein_coding	4.97245222	2.20319046	0.27035632	8.14921003	3.6631E-16	1.6944E-15
MAST1	ENSG00000105613	protein_coding	34.5353958	1.37291839	0.20838012	6.58852877	4.4421E-11	1.4526E-10
JAK3	ENSG00000105639	protein_coding	1771.53792	2.42729775	0.13004351	18.6652738	9.4888E-78	6.1611E-76
RPL18A	ENSG00000105640	protein_coding	15721.7274	1.12252262	0.09083471	12.3578594	4.4176E-35	5.568E-34
SLC5A5	ENSG00000105641	protein_coding	9.0180057	2.42351444	0.23317694	10.3934566	2.6555E-25	2.0699E-24
KCNN1	ENSG00000105642	protein_coding	194.169606	4.28688163	0.2229818	19.2252533	2.2753E-82	1.7211E-80
ARRDC2	ENSG00000105643	protein_coding	4821.58078	1.96056397	0.0994537	19.7133339	1.6568E-86	1.4691E-84
ATP4A	ENSG00000105675	protein_coding	3.39371087	2.67416629	0.39837593	6.71267028	1.9109E-11	6.4176E-11
GAPDHS	ENSG00000105679	protein_coding	1.22226712	2.15240837	0.38493435	5.59162463	2.2495E-08	5.9306E-08
HAMP	ENSG00000105697	protein_coding	90.3523433	3.77763812	0.22893321	16.5010491	3.6057E-61	1.2685E-59
SCN1B	ENSG00000105711	protein_coding	1056.26091	1.9890675	0.10231227	19.4411439	3.4636E-84	2.7953E-82
PBX4	ENSG00000105717	protein_coding	131.63391	1.48535987	0.14606021	10.169504	2.7129E-24	1.9962E-23
PIK3CG	ENSG00000105851	protein_coding	570.225718	1.22329989	0.13038318	9.38234433	6.4522E-21	3.9526E-20
DNAH11	ENSG00000105877	protein_coding	2194.50266	4.61392318	0.20891546	22.0851212	4.394E-108	8.681E-106
DLX5	ENSG00000105880	protein_coding	144.067881	3.41990461	0.15568004	21.967521	5.89E-107	1.134E-104
STEAP1B	ENSG00000105889	protein_coding	28.7401751	1.92595984	0.17337346	11.1087353	1.1376E-28	1.0567E-27

CAV2	ENSG00000105971	protein_coding	9017.14161	1.99417841	0.08903765	22.397025	4.208E-111	9.318E-109
CAV1	ENSG00000105974	protein_coding	14643.4096	2.22662137	0.10818965	20.5807242	4.0853E-94	4.8897E-92
MET	ENSG00000105976	protein_coding	17805.8509	1.17391307	0.10029744	11.7043171	1.2113E-31	1.301E-30
HOXA13	ENSG00000106031	protein_coding	98.3176558	2.76011902	0.24567287	11.2349361	2.7469E-29	2.6421E-28
GRB10	ENSG00000106070	protein_coding	9893.6113	1.43234543	0.09526527	15.0353374	4.3085E-51	1.0619E-49
CRHR2	ENSG00000106113	protein_coding	47.6257135	1.23574763	0.18977627	6.51160244	7.4353E-11	2.3917E-10
NPTX2	ENSG00000106236	protein_coding	10590.4821	6.97640638	0.25802096	27.0381382	5.266E-161	4.734E-158
CYP3A5	ENSG00000106258	protein_coding	1911.73224	2.0812182	0.17514813	11.8826174	1.4573E-32	1.6306E-31
NUDT1	ENSG00000106268	protein_coding	517.621216	1.2402824	0.08985925	13.8025018	2.4616E-43	4.4269E-42
TFR2	ENSG00000106327	protein_coding	171.905187	4.03826161	0.23744025	17.0074854	7.2274E-65	2.8885E-63
FSCN3	ENSG00000106328	protein_coding	8.15112688	1.63242691	0.17354808	9.40619376	5.1443E-21	3.1705E-20
PAX4	ENSG00000106331	protein_coding	0.44963958	1.47425318	0.53405778	2.76047505	0.00577174	0.0089166
PPP1R17	ENSG00000106341	protein_coding	4.17861622	1.34590721	0.24269693	5.54562937	2.929E-08	7.6471E-08
SERPINE1	ENSG00000106366	protein_coding	21070.4924	2.24354176	0.20940299	10.7139912	8.7506E-27	7.386E-26
MOGAT3	ENSG00000106384	protein_coding	231.508061	2.2341169	0.23861298	9.36293099	7.7555E-21	4.7339E-20
PLOD3	ENSG00000106397	protein_coding	6892.45552	1.37956545	0.07748901	17.8033683	6.6543E-71	3.3302E-69
NOBOX	ENSG00000106410	protein_coding	0.76905727	1.16219455	0.3737982	3.10914968	0.00187627	0.00306926
MYL10	ENSG00000106436	protein_coding	1.63169291	1.63753475	0.34793232	4.70647494	2.5204E-06	5.5514E-06
EZH2	ENSG00000106462	protein_coding	433.850465	1.8516976	0.09100009	20.3483054	4.806E-92	5.2833E-90
MEOX2	ENSG00000106511	protein_coding	159.719535	1.22853284	0.21321011	5.76207598	8.3086E-09	2.2706E-08
RARRES2	ENSG00000106538	protein_coding	13580.6636	1.63850898	0.14443852	11.343989	7.9439E-30	7.8195E-29
GIMAP2	ENSG00000106560	protein_coding	883.472245	1.26919361	0.09463238	13.4118318	5.155E-41	8.3887E-40
GALNTL5	ENSG00000106648	protein_coding	1.52457749	3.00152571	0.43197636	6.94835646	3.6957E-12	1.3053E-11
LHX2	ENSG00000106689	protein_coding	11.7752905	3.35088974	0.30220975	11.0879604	1.4352E-28	1.3249E-27
PRUNE2	ENSG00000106772	protein_coding	13635.809	1.28093742	0.14764659	8.67569917	4.1102E-18	2.1506E-17

TRIM14	ENSG00000106785	protein_coding	3092.53039	1.09388065	0.10069688	10.8631042	1.7277E-27	1.5092E-26
AKNA	ENSG00000106948	protein_coding	2628.16536	1.45515626	0.09337647	15.5837571	9.3871E-55	2.6481E-53
TNFSF8	ENSG00000106952	protein_coding	262.486563	1.82520758	0.12631794	14.4493138	2.532E-47	5.366E-46
DNM1	ENSG00000106976	protein_coding	1714.06323	1.42402098	0.15876043	8.96962163	2.9753E-19	1.6641E-18
DOCK8	ENSG00000107099	protein_coding	5562.9905	1.17760449	0.08795724	13.388375	7.0711E-41	1.143E-39
KCNT1	ENSG00000107147	protein_coding	19.8328541	2.37179572	0.18823727	12.6000328	2.1106E-36	2.8389E-35
CA9	ENSG00000107159	protein_coding	11697.2025	6.04133579	0.20027662	30.1649583	6.828E-200	1.647E-196
LHX3	ENSG00000107187	protein_coding	0.73066467	1.81650677	0.53858918	3.37271306	0.00074431	0.00127361
NPDC1	ENSG00000107281	protein_coding	2899.22497	1.38329606	0.10953082	12.6292858	1.4559E-36	1.9723E-35
PDLIM1	ENSG00000107438	protein_coding	10568.7289	1.66222638	0.08423739	19.7326434	1.131E-86	1.0048E-84
DNTT	ENSG00000107447	protein_coding	6.10007171	2.0490471	0.45512665	4.50214699	6.727E-06	1.4235E-05
RASSF4	ENSG00000107551	protein_coding	10741.744	1.67401976	0.10459263	16.0051409	1.1765E-57	3.6813E-56
PKD2L1	ENSG00000107593	protein_coding	56.1684331	3.2185004	0.21758792	14.7917236	1.6566E-49	3.8411E-48
PALD1	ENSG00000107719	protein_coding	1933.67003	1.94915127	0.09068854	21.4928076	1.818E-102	3.041E-100
UNC5B	ENSG00000107731	protein_coding	6334.3209	1.74513389	0.1128522	15.4638889	6.0814E-54	1.6673E-52
CDH23	ENSG00000107736	protein_coding	523.166634	2.34802888	0.17512843	13.407468	5.4674E-41	8.8907E-40
VSIR	ENSG00000107738	protein_coding	4674.71686	1.18254828	0.07742295	15.273873	1.1419E-52	3.0117E-51
LIPA	ENSG00000107798	protein_coding	11850.0297	1.16845699	0.09822064	11.8962471	1.2379E-32	1.3902E-31
TLX1	ENSG00000107807	protein_coding	2.57443004	2.57014222	0.43298288	5.93589804	2.9224E-09	8.3039E-09
SFXN3	ENSG00000107819	protein_coding	4831.92065	1.21242718	0.06697423	18.1028913	3.0237E-73	1.6423E-71
EBF3	ENSG00000108001	protein_coding	274.456547	1.65780919	0.1557399	10.6447303	1.8452E-26	1.5337E-25
RPL28	ENSG00000108107	protein_coding	30286.8601	1.10610101	0.09660549	11.4496699	2.3604E-30	2.379E-29
CRYBA1	ENSG00000108255	protein_coding	4.03214452	1.69908122	0.23270966	7.3012923	2.8502E-13	1.0872E-12
RAPGEFL1	ENSG00000108352	protein_coding	340.735624	1.7716152	0.11848362	14.9524057	1.5022E-50	3.6436E-49
SEPTIN4	ENSG00000108387	protein_coding	2104.38962	1.1299436	0.10871897	10.3932511	2.6612E-25	2.074E-24

CCL1	ENSG00000108702	protein_coding	0.70414779	1.55250268	0.40549896	3.82862313	0.00012886	0.00023975
KRT32	ENSG00000108759	protein_coding	7.09691867	3.76596158	0.31998038	11.7693514	5.6155E-32	6.1232E-31
KAT2A	ENSG00000108773	protein_coding	2390.18483	1.11924703	0.104723	10.6876902	1.1623E-26	9.7459E-26
CNTNAP1	ENSG00000108797	protein_coding	766.84991	1.62669792	0.11849068	13.728488	6.8549E-43	1.2123E-41
ABI3	ENSG00000108798	protein_coding	1532.52015	1.06186698	0.08074845	13.1503081	1.6948E-39	2.5984E-38
DLX4	ENSG00000108813	protein_coding	57.1891517	2.66749884	0.19287725	13.8300332	1.6794E-43	3.0502E-42
COL1A1	ENSG00000108821	protein_coding	59064.7644	2.79236266	0.20603428	13.5529034	7.6157E-42	1.2802E-40
ALOX12	ENSG00000108839	protein_coding	139.449712	1.20622101	0.09123633	13.2208417	6.6515E-40	1.0305E-38
ABCC3	ENSG00000108846	protein_coding	10323.8217	2.30367086	0.13296185	17.3258038	3.0047E-67	1.3056E-65
CACNG1	ENSG00000108878	protein_coding	1.56095697	1.7795774	0.3463066	5.13873372	2.766E-07	6.6615E-07
SLC16A6	ENSG00000108932	protein_coding	371.02157	1.65098041	0.15085719	10.9439952	7.1001E-28	6.342E-27
WSB1	ENSG00000109046	protein_coding	7760.10468	1.00464313	0.11061511	9.08233139	1.0627E-19	6.0965E-19
RCVRN	ENSG00000109047	protein_coding	2.66385028	1.49386109	0.23671179	6.3108858	2.7744E-10	8.5466E-10
MYH1	ENSG00000109061	protein_coding	10.5030321	1.15555329	0.36837631	3.1368827	0.00170754	0.00280769
MYH3	ENSG00000109063	protein_coding	162.810723	1.42638357	0.14332089	9.95237708	2.4623E-23	1.7165E-22
ALDOC	ENSG00000109107	protein_coding	5011.05027	2.2811126	0.14658319	15.5618976	1.3212E-54	3.7157E-53
PHOX2B	ENSG00000109132	protein_coding	0.93096351	1.8367298	0.517315	3.55050562	0.00038449	0.00067929
NMU	ENSG00000109255	protein_coding	49.2040369	2.86449338	0.28150983	10.1754649	2.5518E-24	1.8816E-23
PF4V1	ENSG00000109272	protein_coding	87.0574973	2.12137598	0.23309761	9.10080523	8.9665E-20	5.165E-19
AREG	ENSG00000109321	protein_coding	311.333393	1.09871514	0.18828898	5.83526005	5.3707E-09	1.4925E-08
IL2	ENSG00000109471	protein_coding	3.61641947	2.57219449	0.27344078	9.40676994	5.1162E-21	3.154E-20
CPE	ENSG00000109472	protein_coding	16471.3908	1.0001251	0.17083459	5.85434767	4.7889E-09	1.3366E-08
ANXA10	ENSG00000109511	protein_coding	5.04883218	1.90823121	0.25130259	7.59336084	3.1171E-14	1.2702E-13
NEIL3	ENSG00000109674	protein_coding	63.6647554	3.07236552	0.17260891	17.7995763	7.1206E-71	3.5518E-69
NCAPG	ENSG00000109805	protein_coding	238.640288	2.36517916	0.13674614	17.2961308	5.0306E-67	2.1674E-65

CRYAB	ENSG00000109846	protein_coding	56295.9881	1.02969751	0.13913364	7.40078053	1.3539E-13	5.2923E-13
DBX1	ENSG00000109851	protein_coding	0.52931568	1.47887095	0.5216149	2.83517771	0.00458002	0.00716856
HTATIP2	ENSG00000109854	protein_coding	2756.59929	1.20319125	0.08711208	13.8119902	2.1579E-43	3.8929E-42
CRTAM	ENSG00000109943	protein_coding	162.308072	3.03213792	0.16910608	17.9303895	6.83E-72	3.5179E-70
P2RX3	ENSG00000109991	protein_coding	11.9962457	2.90180827	0.23093957	12.5652275	3.2793E-36	4.3674E-35
LPXN	ENSG00000110031	protein_coding	1305.34672	1.31164749	0.0810266	16.1878628	6.1425E-59	2.0113E-57
NRXN2	ENSG00000110076	protein_coding	1878.94747	1.49019896	0.16848564	8.84466469	9.1804E-19	4.9969E-18
MS4A6A	ENSG00000110077	protein_coding	4459.02145	2.23425574	0.1218125	18.341761	3.8415E-75	2.2376E-73
MS4A4A	ENSG00000110079	protein_coding	1324.83534	1.90825559	0.12993812	14.6858791	7.9398E-49	1.7956E-47
CCND1	ENSG00000110092	protein_coding	45193.3711	2.28721355	0.11097657	20.6098776	2.238E-94	2.6998E-92
HPX	ENSG00000110169	protein_coding	49.5644848	2.41719275	0.17490223	13.820251	1.924E-43	3.4805E-42
APOA5	ENSG00000110243	protein_coding	2.07908777	1.38964937	0.42947685	3.23567937	0.00121354	0.00202969
APOA4	ENSG00000110244	protein_coding	2.8762989	3.47034317	0.71372367	4.86230641	1.1603E-06	2.6379E-06
IL10RA	ENSG00000110324	protein_coding	2871.25678	2.2202479	0.12327453	18.0105965	1.6089E-72	8.4869E-71
UPK2	ENSG00000110375	protein_coding	12.8008187	1.06778179	0.16730203	6.38236013	1.7438E-10	5.4509E-10
SLC15A3	ENSG00000110446	protein_coding	2467.45402	1.6491702	0.09910179	16.6411747	3.5067E-62	1.2718E-60
CD5	ENSG00000110448	protein_coding	457.524537	2.09718337	0.14307114	14.658326	1.1917E-48	2.6727E-47
ACCS	ENSG00000110455	protein_coding	1146.93687	1.25744529	0.10675427	11.7788757	5.0158E-32	5.4836E-31
MDK	ENSG00000110492	protein_coding	2857.49158	1.72642316	0.18478571	9.34283916	9.3785E-21	5.7003E-20
C11orf21	ENSG00000110665	protein_coding	125.94407	2.62899253	0.14492431	18.1404522	1.5277E-73	8.4172E-72
TCIRG1	ENSG00000110719	protein_coding	3112.58519	1.48554637	0.09860865	15.0650719	2.7487E-51	6.8368E-50
POU2AF1	ENSG00000110777	protein_coding	281.956343	1.36870688	0.23996691	5.70373163	1.1721E-08	3.1655E-08
VWF	ENSG00000110799	protein_coding	61569.1747	3.00160491	0.14129097	21.2441384	3.735E-100	5.5769E-98
CD69	ENSG00000110848	protein_coding	495.220799	1.20668919	0.12984784	9.29310166	1.4986E-20	9.0282E-20
CLEC2B	ENSG00000110852	protein_coding	1065.61481	2.30073917	0.12487674	18.4240804	8.421E-76	5.0461E-74

SELPLG	ENSG00000110876	protein_coding	1720.51512	2.04069066	0.10987576	18.5727096	5.3437E-77	3.3834E-75
CORO1C	ENSG00000110880	protein_coding	9543.81373	1.0510561	0.06945943	15.1319422	9.9709E-52	2.5267E-50
BIN2	ENSG00000110934	protein_coding	942.7901	2.00266234	0.10103251	19.8219596	1.9247E-87	1.761E-85
FOXM1	ENSG00000111206	protein_coding	598.797345	2.42292025	0.149656	16.1899301	5.9396E-59	1.9476E-57
PRR4	ENSG00000111215	protein_coding	66.6362065	1.03506099	0.11732941	8.82183784	1.126E-18	6.0917E-18
MYL2	ENSG00000111245	protein_coding	1.85168822	1.61031725	0.31050207	5.1861724	2.1466E-07	5.215E-07
RAD51AP1	ENSG00000111247	protein_coding	207.625042	1.30338375	0.10381522	12.5548421	3.7393E-36	4.9641E-35
SH2B3	ENSG00000111252	protein_coding	5295.10297	1.27995006	0.07901319	16.1991945	5.1092E-59	1.6801E-57
OAS2	ENSG00000111335	protein_coding	3190.72265	1.31361353	0.09989308	13.1501955	1.6974E-39	2.6014E-38
ARHGDIB	ENSG00000111348	protein_coding	14292.4248	1.65034506	0.07981065	20.6782573	5.437E-95	6.7544E-93
IL26	ENSG00000111536	protein_coding	2.45615863	2.6974792	0.29885112	9.02616391	1.778E-19	1.0071E-18
IFNG	ENSG00000111537	protein_coding	46.6580957	4.47676961	0.25678694	17.4337902	4.5713E-68	2.0424E-66
GAPDH	ENSG00000111640	protein_coding	233555.095	1.45971327	0.08508102	17.1567436	5.5965E-66	2.3217E-64
ACRBP	ENSG00000111644	protein_coding	245.785319	1.25129452	0.1027221	12.1813569	3.9073E-34	4.7197E-33
GNB3	ENSG00000111664	protein_coding	43.1783736	1.41984599	0.21361926	6.64661978	2.999E-11	9.9405E-11
CDCA3	ENSG00000111665	protein_coding	222.410614	1.68404424	0.13662053	12.3264365	6.527E-35	8.1727E-34
ENO2	ENSG00000111674	protein_coding	10587.2278	3.19343955	0.1287284	24.8075765	7.427E-136	3.305E-133
NT5DC3	ENSG00000111696	protein_coding	2437.48539	2.2564405	0.13485531	16.7323074	7.6227E-63	2.8203E-61
SLCO1B3	ENSG00000111700	protein_coding	0.78952459	1.46871022	0.57934579	2.5351185	0.01124093	0.0167038
APOBEC1	ENSG00000111701	protein_coding	0.65093611	1.50350559	0.52956039	2.83915793	0.00452328	0.00708458
NANOG	ENSG00000111704	protein_coding	3.19821165	1.56529236	0.26539749	5.89791697	3.6812E-09	1.0372E-08
CLEC4A	ENSG00000111729	protein_coding	225.308002	1.67968748	0.09931949	16.9119632	3.6727E-64	1.4304E-62
AICDA	ENSG00000111732	protein_coding	47.0597175	4.5177752	0.23965303	18.8513169	2.8662E-79	1.935E-77
KLRB1	ENSG00000111796	protein_coding	340.417928	1.5506035	0.13583287	11.4155251	3.4979E-30	3.4911E-29
BTN3A3	ENSG00000111801	protein_coding	3067.13337	1.44514431	0.08774785	16.4692844	6.0988E-61	2.1276E-59

GABRR2	ENSG00000111886	protein_coding	42.0902608	1.29948618	0.15645007	8.30607599	9.8919E-17	4.7497E-16
OPRM1	ENSG00000112038	protein_coding	0.92380753	1.85018217	0.43556863	4.24773967	2.1594E-05	4.3529E-05
RHAG	ENSG00000112077	protein_coding	5.35405704	2.56753734	0.40531126	6.33472984	2.3776E-10	7.3543E-10
SOD2	ENSG00000112096	protein_coding	93317.3001	1.20448499	0.12327167	9.77097958	1.4999E-22	1.0039E-21
GLP1R	ENSG00000112164	protein_coding	32.4881452	1.25023412	0.24480488	5.10706364	3.272E-07	7.8272E-07
VNN2	ENSG00000112303	protein_coding	536.531179	1.79380074	0.16146866	11.1092811	1.1307E-28	1.0505E-27
B3GAT2	ENSG00000112309	protein_coding	44.94583	1.11413566	0.13012279	8.56218718	1.1075E-17	5.6364E-17
NR2E1	ENSG00000112333	protein_coding	105.255127	3.89625621	0.33173213	11.745188	7.4756E-32	8.0918E-31
SLC17A2	ENSG00000112337	protein_coding	238.730967	3.27489511	0.23015479	14.2290979	6.0457E-46	1.2092E-44
ADGRG6	ENSG00000112414	protein_coding	3825.94058	1.05958906	0.14386713	7.36505327	1.7708E-13	6.8692E-13
CCR6	ENSG00000112486	protein_coding	26.8086174	2.56918857	0.21332902	12.0433152	2.1033E-33	2.4527E-32
GUCA1B	ENSG00000112599	protein_coding	32.6198768	1.11699825	0.13661571	8.17620679	2.9292E-16	1.3629E-15
VEGFA	ENSG00000112715	protein_coding	90545.8928	3.45474801	0.12071844	28.6182299	3.986E-180	6.3E-177
TTK	ENSG00000112742	protein_coding	144.662756	1.83182948	0.1284402	14.2621194	3.7683E-46	7.6032E-45
LAMA4	ENSG00000112769	protein_coding	9781.69837	2.64727462	0.11230502	23.5721842	7.436E-123	2.273E-120
LY86	ENSG00000112799	protein_coding	702.54636	2.47267041	0.12205809	20.2581436	3.011E-91	3.1513E-89
MEP1A	ENSG00000112818	protein_coding	7.98183449	4.06270392	0.31441642	12.9214114	3.4083E-38	4.9381E-37
PCDHB2	ENSG00000112852	protein_coding	281.433375	1.13742363	0.18273312	6.2245072	4.8307E-10	1.4639E-09
KIF20A	ENSG00000112984	protein_coding	406.429962	2.34876296	0.15707143	14.9534703	1.4784E-50	3.5877E-49
LOX	ENSG00000113083	protein_coding	18587.7974	4.03807225	0.20197033	19.9933933	6.2871E-89	6.0676E-87
GZMK	ENSG00000113088	protein_coding	932.526402	3.32690229	0.20064628	16.5809318	9.5733E-62	3.4099E-60
APBB3	ENSG00000113108	protein_coding	1114.26171	1.46697795	0.11110698	13.2032921	8.3983E-40	1.2963E-38
SPARC	ENSG00000113140	protein_coding	144572.414	1.81111039	0.09947793	18.2061525	4.6124E-74	2.5817E-72
PCDHB6	ENSG00000113211	protein_coding	322.875901	1.80169314	0.18055603	9.97858176	1.8915E-23	1.3268E-22
CLK4	ENSG00000113240	protein_coding	2182.44579	1.4879926	0.09162801	16.2394954	2.651E-59	8.7869E-58

HAVCR1	ENSG00000113249	protein_coding	3030.55415	2.17419503	0.21026133	10.3404418	4.624E-25	3.5458E-24
ITK	ENSG00000113263	protein_coding	511.994188	2.08953169	0.14425071	14.485417	1.4981E-47	3.1971E-46
THBS4	ENSG00000113296	protein_coding	428.540425	1.70646265	0.20798043	8.20491937	2.3075E-16	1.0809E-15
IL12B	ENSG00000113302	protein_coding	23.381125	1.82015677	0.24630208	7.38993661	1.469E-13	5.7301E-13
BTNL8	ENSG00000113303	protein_coding	22.5832717	1.0800726	0.15725782	6.86816462	6.5033E-12	2.2585E-11
GABRG2	ENSG00000113327	protein_coding	1.24838917	2.29337648	0.57130519	4.01427558	5.9629E-05	0.00011486
CDH6	ENSG00000113361	protein_coding	18047.6787	1.78438012	0.15298704	11.6636034	1.9559E-31	2.0769E-30
LMNB1	ENSG00000113368	protein_coding	947.275778	1.33908305	0.10222256	13.0996825	3.3064E-39	5.0105E-38
IRX4	ENSG00000113430	protein_coding	2.4789437	1.87650698	0.47866652	3.92028037	8.8446E-05	0.00016742
IL4	ENSG00000113520	protein_coding	2.80254073	1.52475224	0.238584	6.39084039	1.6498E-10	5.1663E-10
IL5	ENSG00000113525	protein_coding	4.39943982	1.30988775	0.22296224	5.87493089	4.2302E-09	1.1858E-08
ST8SIA4	ENSG00000113532	protein_coding	3294.56021	3.54390824	0.1288263	27.5091988	1.363E-166	1.562E-163
PCDH12	ENSG00000113555	protein_coding	4252.13025	1.15708593	0.11645492	9.93591309	2.905E-23	2.0189E-22
DPYSL3	ENSG00000113657	protein_coding	5183.24116	1.12224872	0.14897653	7.53305728	4.9566E-14	1.9952E-13
STC2	ENSG00000113739	protein_coding	7364.5469	4.24178781	0.14124574	30.0312624	3.836E-198	8.405E-195
HRH2	ENSG00000113749	protein_coding	904.901131	3.43002183	0.19316691	17.7567778	1.5276E-70	7.5054E-69
UNC5A	ENSG00000113763	protein_coding	136.318472	3.41830377	0.21881349	15.6219972	5.1565E-55	1.47E-53
NPHP3	ENSG00000113971	protein_coding	639.250551	1.14912993	0.09292177	12.3666388	3.9605E-35	5.0056E-34
CD86	ENSG00000114013	protein_coding	974.971074	2.12822927	0.12475543	17.0592121	2.9856E-65	1.2079E-63
GRK7	ENSG00000114124	protein_coding	5.89177879	1.34760098	0.17495701	7.70246914	1.3346E-14	5.572E-14
LRRC31	ENSG00000114248	protein_coding	57.5062583	1.15590439	0.25878947	4.46658209	7.9479E-06	1.6698E-05
PFKFB4	ENSG00000114268	protein_coding	1618.95357	2.41548038	0.11128181	21.7059771	1.802E-104	3.226E-102
HHLA2	ENSG00000114455	protein_coding	3662.51206	3.10893655	0.21381894	14.5400426	6.7551E-48	1.4634E-46
AADAC	ENSG00000114771	protein_coding	4.42539558	2.03612422	0.38374701	5.30590257	1.1212E-07	2.7897E-07
DNAH1	ENSG00000114841	protein_coding	805.382679	1.36110021	0.12480831	10.9055254	1.0846E-27	9.595E-27

CCL20	ENSG00000115009	protein_coding	1269.79282	3.62834657	0.25430788	14.2675349	3.4869E-46	7.0477E-45
ZAP70	ENSG00000115085	protein_coding	479.921649	2.88014804	0.14908361	19.3190117	3.717E-83	2.8541E-81
STEAP3	ENSG00000115107	protein_coding	2557.04877	1.40564586	0.19979816	7.03532943	1.9879E-12	7.1613E-12
OTOF	ENSG00000115155	protein_coding	20.7330735	2.59722948	0.18114197	14.3380881	1.2649E-46	2.6037E-45
CENPA	ENSG00000115163	protein_coding	73.3189761	2.44585171	0.16803642	14.5554859	5.3902E-48	1.1738E-46
CYTIP	ENSG00000115165	protein_coding	919.170864	1.23074857	0.12840538	9.584868	9.2576E-22	5.9545E-21
SLC30A3	ENSG00000115194	protein_coding	22.2837241	1.1794076	0.20814486	5.66628252	1.4593E-08	3.9136E-08
ITGA4	ENSG00000115232	protein_coding	1797.76251	1.7377256	0.11935145	14.5597355	5.0653E-48	1.1052E-46
RPS15	ENSG00000115268	protein_coding	14933.3283	1.00097772	0.0968967	10.3303591	5.1368E-25	3.9305E-24
INO80B	ENSG00000115274	protein_coding	199.027052	1.0816236	0.09880113	10.9474824	6.832E-28	6.1038E-27
PCGF1	ENSG00000115289	protein_coding	847.569985	1.02651018	0.05780691	17.7575699	1.5062E-70	7.4081E-69
LOXL3	ENSG00000115318	protein_coding	435.796775	1.44939896	0.09952601	14.5630173	4.8279E-48	1.0544E-46
DOK1	ENSG00000115325	protein_coding	814.967124	1.24195026	0.07368413	16.8550587	9.6312E-64	3.6792E-62
REG1A	ENSG00000115386	protein_coding	3132.19762	2.64436633	0.32415308	8.15777028	3.4127E-16	1.5808E-15
FN1	ENSG00000115414	protein_coding	104396.99	1.80172197	0.14894953	12.0961909	1.1063E-33	1.3087E-32
OTX1	ENSG00000115507	protein_coding	8.72781658	1.26681034	0.29265365	4.32870161	1.4999E-05	3.0688E-05
GNLY	ENSG00000115523	protein_coding	683.065932	3.10991135	0.14920255	20.8435539	1.744E-96	2.3308E-94
PRKAG3	ENSG00000115592	protein_coding	2.95390227	1.81813275	0.2816074	6.45626767	1.0732E-10	3.4083E-10
WNT6	ENSG00000115596	protein_coding	45.8396926	1.15407955	0.18691629	6.17431217	6.6452E-10	1.9905E-09
IL18R1	ENSG00000115604	protein_coding	603.23172	1.25667827	0.11663847	10.7741323	4.5606E-27	3.9099E-26
IL18RAP	ENSG00000115607	protein_coding	109.129991	1.74824174	0.13577209	12.8762967	6.1203E-38	8.7812E-37
ABCB6	ENSG00000115657	protein_coding	608.475636	1.73998419	0.10458636	16.636817	3.7714E-62	1.3645E-60
TPO	ENSG00000115705	protein_coding	6.54290984	1.90121729	0.21142696	8.99231266	2.4208E-19	1.3611E-18
HPCAL1	ENSG00000115756	protein_coding	12407.328	1.82877626	0.10377446	17.6226041	1.6522E-69	7.8162E-68
DLX2	ENSG00000115844	protein_coding	12.0388163	3.13815719	0.21596477	14.5308754	7.7227E-48	1.666E-46

LCT	ENSG00000115850	protein_coding	3.21402792	1.71136354	0.26529251	6.45085503	1.1122E-10	3.5281E-10
DARS1	ENSG00000115866	protein_coding	11045.7006	1.04971791	0.06255931	16.7795625	3.4436E-63	1.2918E-61
SLC1A4	ENSG00000115902	protein_coding	2025.79875	1.93056378	0.07507943	25.7136183	8.232E-146	4.965E-143
WIPF1	ENSG00000115935	protein_coding	5792.02343	1.39192014	0.07267355	19.1530508	9.1279E-82	6.7272E-80
PLEK	ENSG00000115956	protein_coding	2077.71744	2.24101629	0.13256349	16.9052304	4.1171E-64	1.5967E-62
KISS1R	ENSG00000116014	protein_coding	522.125176	6.39337237	0.26324965	24.2863468	2.733E-130	1.027E-127
CD207	ENSG00000116031	protein_coding	72.5632108	1.20336594	0.19368496	6.21300653	5.198E-10	1.5706E-09
VAX2	ENSG00000116035	protein_coding	20.9867259	1.23962145	0.18485622	6.70586822	2.0021E-11	6.7129E-11
TNR	ENSG00000116147	protein_coding	30.3342555	1.09704712	0.23954259	4.57975813	4.6551E-06	1.0011E-05
TPSG1	ENSG00000116176	protein_coding	198.125536	2.47591056	0.28673215	8.63492468	5.8766E-18	3.0453E-17
ELAPOR1	ENSG00000116299	protein_coding	201.279614	1.68729568	0.16872973	9.99999045	1.5241E-23	1.0754E-22
OPRD1	ENSG00000116329	protein_coding	33.6582222	1.78072484	0.14406204	12.360819	4.258E-35	5.3727E-34
NCF2	ENSG00000116701	protein_coding	1078.17505	1.73523467	0.11361562	15.2728528	1.1599E-52	3.0575E-51
PDC	ENSG00000116703	protein_coding	4.79996967	1.83714071	0.22511317	8.16096506	3.3236E-16	1.5407E-15
RPE65	ENSG00000116745	protein_coding	5.60126038	2.74321597	0.24832032	11.0470863	2.2645E-28	2.0695E-27
CD2	ENSG00000116824	protein_coding	1295.28771	3.0058471	0.16234875	18.5147535	1.5701E-76	9.7529E-75
NR5A2	ENSG00000116833	protein_coding	487.866427	1.27352704	0.11270414	11.2997365	1.3161E-29	1.2826E-28
KIF21B	ENSG00000116852	protein_coding	514.941834	2.11875956	0.1146502	18.4802086	2.9803E-76	1.8313E-74
NID1	ENSG00000116962	protein_coding	13500.3125	1.35439235	0.10705935	12.6508549	1.1066E-36	1.5098E-35
BMP8B	ENSG00000116985	protein_coding	401.445833	1.19504764	0.14880469	8.03098122	9.6696E-16	4.3573E-15
SIPA1L2	ENSG00000116991	protein_coding	2932.16191	1.13881075	0.08593235	13.2524095	4.3695E-40	6.8245E-39
SLAMF1	ENSG00000117090	protein_coding	163.13718	1.49045007	0.14716137	10.1279979	4.1506E-24	3.0192E-23
CD48	ENSG00000117091	protein_coding	1300.83634	1.93546225	0.13447602	14.3926199	5.7573E-47	1.2013E-45
ACTL8	ENSG00000117148	protein_coding	4.57411478	4.28637769	0.58446083	7.33390066	2.2355E-13	8.5971E-13
IGSF21	ENSG00000117154	protein_coding	169.295628	1.54038748	0.13651729	11.2834607	1.5839E-29	1.5393E-28

PLA2G2D	ENSG00000117215	protein_coding	148.136753	3.28288946	0.26763511	12.2662884	1.3741E-34	1.6965E-33
GBP1	ENSG00000117228	protein_coding	5624.30812	1.8149794	0.13050094	13.9077881	5.6809E-44	1.0518E-42
CDK18	ENSG00000117266	protein_coding	17148.0801	2.35194125	0.11386574	20.6553905	8.7312E-95	1.0702E-92
CD160	ENSG00000117281	protein_coding	75.2030904	1.85376566	0.1157146	16.0201529	9.2423E-58	2.9099E-56
P3H1	ENSG00000117385	protein_coding	2355.38404	1.31908114	0.08707509	15.1487766	7.719E-52	1.9713E-50
SLC2A1	ENSG00000117394	protein_coding	16502.9858	1.99380523	0.12246682	16.2803707	1.3604E-59	4.5521E-58
CDC20	ENSG00000117399	protein_coding	341.698719	1.69345597	0.15229866	11.11931	1.0105E-28	9.4247E-28
PTCH2	ENSG00000117425	protein_coding	137.551414	1.46120906	0.12877533	11.3469644	7.6782E-30	7.5613E-29
MROH9	ENSG00000117501	protein_coding	2.74404975	2.87466316	0.33889075	8.48256606	2.2028E-17	1.1025E-16
FASLG	ENSG00000117560	protein_coding	155.000431	3.48046509	0.17513595	19.8729336	6.9802E-88	6.4905E-86
PLPPR5	ENSG00000117598	protein_coding	386.879562	6.37725596	0.2960849	21.538606	6.771E-103	1.145E-100
RSRP1	ENSG00000117616	protein_coding	2417.99358	1.03097947	0.12808143	8.04940653	8.3196E-16	3.7623E-15
NEK2	ENSG00000117650	protein_coding	137.87928	2.0523158	0.15408546	13.3193351	1.7869E-40	2.8482E-39
CENPF	ENSG00000117724	protein_coding	764.629942	1.80501055	0.14184047	12.7256387	4.2598E-37	5.9155E-36
MUC5B	ENSG00000117983	protein_coding	27.6149635	2.88531592	0.24645444	11.7072995	1.1695E-31	1.2569E-30
A4GNT	ENSG00000118017	protein_coding	52.1841005	1.38553432	0.18851443	7.34975197	1.9857E-13	7.6695E-13
KIF14	ENSG00000118193	protein_coding	116.5783	2.71942633	0.14903399	18.2470215	2.185E-74	1.2412E-72
NRP2	ENSG00000118257	protein_coding	4526.80032	1.69097443	0.11154625	15.1594019	6.5665E-52	1.6845E-50
C1orf54	ENSG00000118292	protein_coding	775.447164	1.06536428	0.09247137	11.5210175	1.0338E-30	1.0567E-29
IRAG2	ENSG00000118308	protein_coding	1340.81498	1.21398943	0.11858002	10.2377233	1.3436E-24	1.0062E-23
FILIP1	ENSG00000118407	protein_coding	1225.31181	1.38967151	0.11950731	11.6283394	2.958E-31	3.115E-30
CNR1	ENSG00000118432	protein_coding	131.93988	1.67940767	0.19441866	8.63809941	5.7156E-18	2.9646E-17
PLAGL1	ENSG00000118495	protein_coding	2029.63623	1.2230301	0.1118971	10.9299539	8.2891E-28	7.3739E-27
TNFAIP3	ENSG00000118503	protein_coding	4094.98146	1.27319611	0.09285414	13.7117866	8.6306E-43	1.5193E-41
MYB	ENSG00000118513	protein_coding	53.3565847	2.51176288	0.19246278	13.0506419	6.3014E-39	9.4E-38

ARG1	ENSG00000118520	protein_coding	13.5697154	1.13858997	0.15260836	7.46086258	8.5958E-14	3.4042E-13
PMFBP1	ENSG00000118557	protein_coding	55.8424452	2.08956479	0.15214196	13.7343096	6.3256E-43	1.1209E-41
GHRH	ENSG00000118702	protein_coding	0.46195062	1.11737742	0.4907533	2.27686175	0.02279448	0.03243537
PCDH17	ENSG00000118946	protein_coding	2774.39694	1.98551209	0.13016918	15.2533194	1.5647E-52	4.1035E-51
CCND2	ENSG00000118971	protein_coding	4173.24473	1.18947918	0.10555581	11.2687228	1.8727E-29	1.813E-28
FGF23	ENSG00000118972	protein_coding	2.28702006	1.21730375	0.40300917	3.02053614	0.00252328	0.00406943
NEK6	ENSG00000119408	protein_coding	7048.60552	1.96280741	0.10862572	18.0694535	5.546E-73	2.984E-71
SLC46A2	ENSG00000119457	protein_coding	14.4283593	1.20734908	0.1484037	8.13557277	4.1E-16	1.8906E-15
CSF3R	ENSG00000119535	protein_coding	1079.61384	2.7352057	0.13291374	20.5788026	4.2505E-94	5.0742E-92
PROX2	ENSG00000119608	protein_coding	22.8054271	1.2815288	0.15236296	8.4110256	4.0644E-17	1.9993E-16
PGF	ENSG00000119630	protein_coding	9161.45201	4.66716876	0.20531252	22.7320223	2.161E-114	5.134E-112
BCL11A	ENSG00000119866	protein_coding	198.563072	1.53236037	0.14188668	10.799889	3.4462E-27	2.9701E-26
NKX2-3	ENSG00000119919	protein_coding	13.4034631	3.13231886	0.42517843	7.36706914	1.7442E-13	6.7696E-13
PPP1R3C	ENSG00000119938	protein_coding	4006.19145	1.95662075	0.11480727	17.0426559	3.9632E-65	1.6008E-63
PRLHR	ENSG00000119973	protein_coding	1.67986959	2.76175049	0.37933622	7.28048191	3.3263E-13	1.2638E-12
KCNIP2	ENSG00000120049	protein_coding	104.317156	1.70647451	0.15334638	11.1282351	9.1428E-29	8.5472E-28
CXorf21	ENSG00000120280	protein_coding	207.532616	1.62708848	0.12690615	12.8211944	1.2476E-37	1.7707E-36
PCDHB8	ENSG00000120322	protein_coding	103.500638	1.06959261	0.17569157	6.08789938	1.144E-09	3.3617E-09
PCDHB10	ENSG00000120324	protein_coding	358.455019	1.7026157	0.10947043	15.5532023	1.5135E-54	4.233E-53
PCDHB14	ENSG00000120327	protein_coding	791.765839	1.38076304	0.1124514	12.2787537	1.178E-34	1.4591E-33
SLC25A2	ENSG00000120329	protein_coding	3.88327466	1.69302017	0.25071719	6.75270877	1.4511E-11	4.9154E-11
SEC16B	ENSG00000120341	protein_coding	43.745845	1.71260276	0.1283291	13.3453965	1.26E-40	2.0174E-39
GPR31	ENSG00000120436	protein_coding	7.33270171	2.26394602	0.24441985	9.26252925	1.9965E-20	1.1918E-19
TEX11	ENSG00000120498	protein_coding	332.920859	4.22072864	0.24534794	17.2030329	2.5199E-66	1.0569E-64
LYZL1	ENSG00000120563	protein_coding	7.11431441	4.27308952	0.36501173	11.7067184	1.1775E-31	1.2653E-30

IQSEC3	ENSG00000120645	protein_coding	900.400344	1.19994675	0.23739295	5.05468577	4.311E-07	1.0204E-06
TNFSF11	ENSG00000120659	protein_coding	34.9876931	2.1255455	0.24754792	8.58640033	8.9737E-18	4.5906E-17
TGFBI	ENSG00000120708	protein_coding	91296.36	4.12603907	0.24295989	16.9823879	1.1088E-64	4.4008E-63
MYOT	ENSG00000120729	protein_coding	99.7594977	1.41555827	0.18024835	7.85337733	4.0498E-15	1.7522E-14
DUSP4	ENSG00000120875	protein_coding	1012.63112	1.91178826	0.12660219	15.1007523	1.601E-51	4.017E-50
TNFRSF10B	ENSG00000120889	protein_coding	5715.72009	1.49752639	0.07025312	21.3161555	8.04E-101	1.2409E-98
TBX4	ENSG00000121075	protein_coding	9.19198498	1.807237	0.21039626	8.58968214	8.721E-18	4.4673E-17
NCAPH	ENSG00000121152	protein_coding	170.447528	1.92568112	0.12675996	15.1915567	4.0225E-52	1.0418E-50
LRAT	ENSG00000121207	protein_coding	263.134056	2.27928098	0.17792942	12.8100284	1.4408E-37	2.0411E-36
TMEM131L	ENSG00000121210	protein_coding	1243.10207	1.10198664	0.06369014	17.3023126	4.5188E-67	1.9524E-65
MND1	ENSG00000121211	protein_coding	58.897833	1.53935808	0.11657621	13.2047366	8.2387E-40	1.2729E-38
ADCY7	ENSG00000121281	protein_coding	545.522761	1.9087064	0.13624317	14.0095562	1.3625E-44	2.5767E-43
TAS2R10	ENSG00000121318	protein_coding	7.3802303	1.44363384	0.17250902	8.36845409	5.8379E-17	2.8421E-16
A1BG	ENSG00000121410	protein_coding	15.5482231	1.04280865	0.1433777	7.27315781	3.5118E-13	1.3315E-12
RGSL1	ENSG00000121446	protein_coding	3.6911368	3.24363786	0.31736455	10.2205424	1.6044E-24	1.1955E-23
CSTA	ENSG00000121552	protein_coding	207.621215	2.32670802	0.13595017	17.1144174	1.1587E-65	4.7425E-64
DPPA4	ENSG00000121570	protein_coding	5.63955633	1.21529003	0.22562274	5.38638105	7.189E-08	1.8213E-07
POPDC2	ENSG00000121577	protein_coding	538.943018	1.57011968	0.10545028	14.8896684	3.8467E-50	9.1604E-49
CD80	ENSG00000121594	protein_coding	55.154158	2.46230727	0.15641451	15.7421918	7.7705E-56	2.2717E-54
KIF18A	ENSG00000121621	protein_coding	148.438516	1.54123631	0.11945972	12.9017237	4.4014E-38	6.3588E-37
PILRB	ENSG00000121716	protein_coding	526.492002	2.41598769	0.19452219	12.4201138	2.0327E-35	2.6131E-34
GJB6	ENSG00000121742	protein_coding	15.1103804	1.0470379	0.34338178	3.04919467	0.00229456	0.00371665
CCRL2	ENSG00000121797	protein_coding	388.34223	1.43122597	0.11607474	12.3302104	6.2284E-35	7.8095E-34
CCR2	ENSG00000121807	protein_coding	529.129149	1.324722	0.15021352	8.8189266	1.1556E-18	6.2478E-18
TMEM156	ENSG00000121895	protein_coding	115.952511	1.42334884	0.13101128	10.8643224	1.7048E-27	1.4897E-26

CSMD2	ENSG00000121904	protein_coding	152.954511	1.00396443	0.14331201	7.00544531	2.462E-12	8.8097E-12
HPCA	ENSG00000121905	protein_coding	88.5476707	2.78786347	0.16075842	17.341944	2.2693E-67	9.9074E-66
TMIGD3	ENSG00000121933	protein_coding	895.931034	2.90972008	0.12913536	22.532326	2.002E-112	4.52E-110
CXCR4	ENSG00000121966	protein_coding	10970.205	2.76691091	0.10747453	25.744807	3.685E-146	2.314E-143
SV2C	ENSG00000122012	protein_coding	30.0564388	1.0563712	0.18554907	5.6932174	1.2467E-08	3.3591E-08
SASH3	ENSG00000122122	protein_coding	1404.81712	2.4667579	0.11975453	20.598452	2.8336E-94	3.4004E-92
PAEP	ENSG00000122133	protein_coding	128.850742	7.35970145	0.50521373	14.5675008	4.5213E-48	9.9026E-47
OBP2A	ENSG00000122136	protein_coding	4.13383074	3.79516711	0.34722297	10.9300577	8.2796E-28	7.3669E-27
LAX1	ENSG00000122188	protein_coding	153.551111	2.02155914	0.16683978	12.1167694	8.6086E-34	1.0239E-32
CD244	ENSG00000122223	protein_coding	158.685974	2.29959791	0.12752911	18.0319449	1.0938E-72	5.7964E-71
LY9	ENSG00000122224	protein_coding	180.830106	2.08852843	0.148612	14.0535648	7.3252E-45	1.398E-43
HS3ST2	ENSG00000122254	protein_coding	351.265691	4.89217506	0.22913906	21.3502447	3.879E-101	6.111E-99
OPN4	ENSG00000122375	protein_coding	18.8162953	5.43369728	0.33603128	16.1702128	8.1815E-59	2.6618E-57
ZNF205	ENSG00000122386	protein_coding	973.920009	1.03722107	0.07648911	13.5603752	6.8783E-42	1.1579E-40
LRRC39	ENSG00000122477	protein_coding	67.8710906	1.65413549	0.13873605	11.9228958	8.9928E-33	1.0161E-31
CCDC18	ENSG00000122483	protein_coding	187.009017	1.25422615	0.10752643	11.6643524	1.9387E-31	2.0592E-30
OCM	ENSG00000122543	protein_coding	5.28661625	1.48807326	0.19401681	7.66981625	1.7224E-14	7.1397E-14
ARL4A	ENSG00000122644	protein_coding	1037.32818	1.11871276	0.08469896	13.2081056	7.8782E-40	1.218E-38
TWIST1	ENSG00000122691	protein_coding	65.5908198	1.06222991	0.15761445	6.73941955	1.5902E-11	5.3687E-11
GLIPR2	ENSG00000122694	protein_coding	1253.24296	1.03256533	0.09156435	11.2769361	1.7058E-29	1.6553E-28
AKR1D1	ENSG00000122787	protein_coding	8.45746606	2.38098175	0.3215485	7.40473609	1.3141E-13	5.1413E-13
SFTPA1	ENSG00000122852	protein_coding	2.14121988	1.1065947	0.40352877	2.74229442	0.00610116	0.00940207
NEUROG3	ENSG00000122859	protein_coding	2.45268214	1.81272497	0.31828912	5.69521501	1.2322E-08	3.3212E-08
P4HA1	ENSG00000122884	protein_coding	8762.6426	1.68994956	0.08474007	19.9427449	1.7329E-88	1.6379E-86
HVCN1	ENSG00000122986	protein_coding	600.012806	1.38756283	0.07771891	17.8536063	2.7096E-71	1.371E-69

CDKN2C	ENSG00000123080	protein_coding	1379.02412	1.53522533	0.09140725	16.7954441	2.6352E-63	9.9426E-62
BHLHE41	ENSG00000123095	protein_coding	7752.45951	2.74347267	0.11635957	23.5775428	6.552E-123	2.016E-120
SSPN	ENSG00000123096	protein_coding	2704.97615	1.17062171	0.10309468	11.3548218	7.0183E-30	6.9173E-29
PRDX4	ENSG00000123131	protein_coding	5434.74222	1.4183511	0.08133429	17.4385376	4.207E-68	1.8852E-66
DDX39A	ENSG00000123136	protein_coding	2497.18534	1.01095361	0.0757681	13.3427337	1.3058E-40	2.0886E-39
ADGRE5	ENSG00000123146	protein_coding	5306.55438	1.10749236	0.08612765	12.858732	7.6827E-38	1.0978E-36
CCDC70	ENSG00000123171	protein_coding	0.64937411	1.03820991	0.343924	3.01871898	0.00253846	0.00409262
CENPK	ENSG00000123219	protein_coding	157.069052	1.92948255	0.11343963	17.0088926	7.0559E-65	2.8224E-63
NEUROD4	ENSG00000123307	protein_coding	3.46452038	2.58587064	0.6164506	4.19477351	2.7314E-05	5.453E-05
ARHGAP9	ENSG00000123329	protein_coding	786.842912	2.54480536	0.12685671	20.060471	1.6353E-89	1.6156E-87
NCKAP1L	ENSG00000123338	protein_coding	2909.50561	2.30023615	0.12670301	18.1545499	1.1819E-73	6.5357E-72
PDE1B	ENSG00000123360	protein_coding	980.616388	1.64609244	0.10627243	15.4893642	4.0932E-54	1.129E-52
HOXC13	ENSG00000123364	protein_coding	16.8386636	1.90462482	0.21175544	8.99445528	2.3741E-19	1.335E-18
HOXC12	ENSG00000123407	protein_coding	10.3304665	2.25866868	0.23701841	9.52950734	1.5803E-21	1.0028E-20
TUBA1B	ENSG00000123416	protein_coding	11469.9828	1.07663879	0.0839673	12.8221194	1.2328E-37	1.7508E-36
HJURP	ENSG00000123485	protein_coding	206.375842	2.88255862	0.16100273	17.9037874	1.1017E-71	5.6239E-70
H2BW1	ENSG00000123569	protein_coding	0.52844939	1.3405807	0.46362452	2.89152247	0.0038338	0.00605789
NMI	ENSG00000123609	protein_coding	1430.48486	1.01434441	0.07000657	14.4892742	1.4163E-47	3.0268E-46
TNFAIP6	ENSG00000123610	protein_coding	2797.51529	5.65781903	0.20413433	27.7161564	4.46E-169	5.242E-166
BATF3	ENSG00000123685	protein_coding	214.208184	2.12111115	0.12704419	16.6958526	1.405E-62	5.1524E-61
KCNJ2	ENSG00000123700	protein_coding	1154.90061	1.8568814	0.1326101	14.0025639	1.5035E-44	2.8362E-43
C4BPA	ENSG00000123838	protein_coding	40.6056113	1.36033155	0.3233921	4.20644644	2.5942E-05	5.1903E-05
INHA	ENSG00000123999	protein_coding	89.805753	1.08994238	0.24527304	4.44379209	8.8387E-06	1.8486E-05
GCNT7	ENSG00000124091	protein_coding	15.0521452	1.70535521	0.18422857	9.25673586	2.1078E-20	1.2563E-19
PI3	ENSG00000124102	protein_coding	77.0201307	2.69097819	0.32338431	8.32130102	8.7003E-17	4.1929E-16

FAM209A	ENSG00000124103	protein_coding	5.67586338	1.99086474	0.20123507	9.89322966	4.4542E-23	3.0663E-22
WFDC3	ENSG00000124116	protein_coding	54.4208633	1.05869055	0.18889221	5.60473377	2.0858E-08	5.5172E-08
PREX1	ENSG00000124126	protein_coding	5446.28269	1.34368081	0.0803299	16.7270312	8.3288E-63	3.079E-61
SLC12A5	ENSG00000124140	protein_coding	71.3579695	2.41628491	0.14767873	16.3617663	3.5857E-60	1.223E-58
TOX2	ENSG00000124191	protein_coding	529.630228	1.70296945	0.11984129	14.2102057	7.9193E-46	1.5763E-44
GDAP1L1	ENSG00000124194	protein_coding	12.1439904	1.67846493	0.23610085	7.10910168	1.168E-12	4.28E-12
GTSF1L	ENSG00000124196	protein_coding	5.22874396	3.77232778	0.36121581	10.4434183	1.5705E-25	1.2394E-24
ZNF831	ENSG00000124203	protein_coding	172.368016	2.24216869	0.15626337	14.3486522	1.0863E-46	2.241E-45
CDH26	ENSG00000124215	protein_coding	55.0476603	1.62227978	0.10878171	14.9131671	2.7061E-50	6.4947E-49
PMEPA1	ENSG00000124225	protein_coding	7714.88086	1.59127453	0.12176857	13.0680233	5.0152E-39	7.5279E-38
ANKRD60	ENSG00000124227	protein_coding	0.62728267	1.94284084	0.72253674	2.68891633	0.00716844	0.01094632
RBPJL	ENSG00000124232	protein_coding	3.67014008	1.83367408	0.30636928	5.98517612	2.1616E-09	6.212E-09
ZBP1	ENSG00000124256	protein_coding	144.812392	2.85624177	0.17043513	16.7585271	4.9063E-63	1.827E-61
MAGEA10	ENSG00000124260	protein_coding	2.75208598	3.64431884	0.41015556	8.88521138	6.3799E-19	3.5051E-18
IL9R	ENSG00000124334	protein_coding	19.8398255	2.11794484	0.17236938	12.2872453	1.0606E-34	1.3165E-33
IRGC	ENSG00000124449	protein_coding	1.00125564	2.17892253	0.41273562	5.27922091	1.2973E-07	3.2105E-07
PSG8	ENSG00000124467	protein_coding	1.69601015	2.5183723	0.57291382	4.39572623	1.104E-05	2.2887E-05
CEACAM8	ENSG00000124469	protein_coding	2.59297483	1.20570788	0.30828226	3.9110517	9.1895E-05	0.00017366
GRM4	ENSG00000124493	protein_coding	12.7133415	1.99878158	0.24214233	8.25457306	1.5245E-16	7.2343E-16
BTN2A2	ENSG00000124508	protein_coding	1560.40781	1.17229518	0.07273047	16.1183505	1.896E-58	6.0994E-57
BTN1A1	ENSG00000124557	protein_coding	7.13303182	1.11267941	0.16079405	6.91990423	4.5195E-12	1.5858E-11
SLC17A3	ENSG00000124564	protein_coding	12486.1476	1.60646386	0.24426765	6.57665415	4.8115E-11	1.5697E-10
H2BC11	ENSG00000124635	protein_coding	38.390984	1.16232066	0.17588986	6.60822989	3.8894E-11	1.2772E-10
OR2B6	ENSG00000124657	protein_coding	3.09424833	2.08978144	0.27968954	7.47178968	7.9111E-14	3.1431E-13
SPDEF	ENSG00000124664	protein_coding	27.7343208	2.97084257	0.27271099	10.8937398	1.2346E-27	1.0886E-26

TREM1	ENSG00000124731	protein_coding	228.777894	2.10980503	0.20541763	10.2708078	9.5402E-25	7.1989E-24
KLHL31	ENSG00000124743	protein_coding	97.7567188	1.66484179	0.13089811	12.7186086	4.6609E-37	6.453E-36
COL21A1	ENSG00000124749	protein_coding	744.542293	2.20753475	0.1710988	12.9021056	4.3796E-38	6.3294E-37
CPNE5	ENSG00000124772	protein_coding	545.500143	1.75326698	0.13526988	12.9612518	2.0291E-38	2.9604E-37
KCNK17	ENSG00000124780	protein_coding	40.4217701	3.50715808	0.27628874	12.6938149	6.3991E-37	8.8064E-36
NRN1	ENSG00000124785	protein_coding	1259.24591	1.12271937	0.18032966	6.22592731	4.7872E-10	1.4512E-09
RUNX2	ENSG00000124813	protein_coding	599.624548	1.45095103	0.12034045	12.0570515	1.7804E-33	2.0821E-32
OPN5	ENSG00000124818	protein_coding	2.3497122	1.10634521	0.2740198	4.03746452	5.4032E-05	0.00010463
GCM2	ENSG00000124827	protein_coding	1.66401277	1.24116624	0.3213682	3.86213143	0.0001124	0.00021053
WNT1	ENSG00000125084	protein_coding	4.24685385	2.5241345	0.24853819	10.1559222	3.1185E-24	2.284E-23
GPR18	ENSG00000125245	protein_coding	66.7996273	2.17032381	0.16368368	13.2592564	3.9883E-40	6.2484E-39
SOX21	ENSG00000125285	protein_coding	4.2399285	2.35738738	0.31384588	7.51128982	5.8548E-14	2.346E-13
HROB	ENSG00000125319	protein_coding	120.776153	1.53766635	0.09716589	15.8251654	2.0865E-56	6.2068E-55
KIF25	ENSG00000125337	protein_coding	22.9123187	1.14752614	0.16364672	7.01221608	2.3457E-12	8.4061E-12
IRF1	ENSG00000125347	protein_coding	4972.03979	1.10611818	0.10560945	10.4736668	1.1413E-25	9.0834E-25
TMEM255A	ENSG00000125355	protein_coding	570.360633	1.258907	0.24052745	5.23394325	1.6593E-07	4.0715E-07
AMELX	ENSG00000125363	protein_coding	1.52108438	2.26722187	0.44407406	5.10550394	3.2991E-07	7.8888E-07
MYH2	ENSG00000125414	protein_coding	4.70430157	1.22274135	0.38782486	3.15281835	0.00161702	0.00266592
C1orf61	ENSG00000125462	protein_coding	10.4396448	2.68398067	0.22359252	12.0038929	3.3897E-33	3.92E-32
KIR2DL1	ENSG00000125498	protein_coding	11.2525019	2.44309755	0.18405229	13.2739316	3.2791E-40	5.1603E-39
BHLHE23	ENSG00000125533	protein_coding	0.56670526	1.50997339	0.40632837	3.71614066	0.00020229	0.00036862
PLGLB2	ENSG00000125551	protein_coding	3.74175556	1.75456005	0.2205124	7.95674109	1.7663E-15	7.8359E-15
IL37	ENSG00000125571	protein_coding	1.31179064	2.57580591	0.33928432	7.59188017	3.153E-14	1.2842E-13
TNFSF9	ENSG00000125657	protein_coding	527.941279	3.45990391	0.16332285	21.184445	1.329E-99	1.9462E-97
CD70	ENSG00000125726	protein_coding	2928.12793	6.09412712	0.23412911	26.0289164	2.331E-149	1.696E-146

C3	ENSG00000125730	protein_coding	102650.517	3.73173755	0.18332815	20.3555072	4.1493E-92	4.5724E-90
TNFSF14	ENSG00000125735	protein_coding	392.095424	3.77583674	0.20364897	18.5409075	9.6578E-77	6.0481E-75
TGM3	ENSG00000125780	protein_coding	13.0082396	1.80210583	0.21354437	8.43902307	3.2E-17	1.584E-16
CD93	ENSG00000125810	protein_coding	16106.755	1.04721632	0.1144977	9.14617753	5.8984E-20	3.437E-19
NKX2-2	ENSG00000125820	protein_coding	3.43316795	1.89288357	0.40930323	4.62464848	3.7523E-06	8.1348E-06
CSTL1	ENSG00000125823	protein_coding	1.09371402	1.84728587	0.55789434	3.31117512	0.00092905	0.00157328
RBCK1	ENSG00000125826	protein_coding	7167.08201	1.19351836	0.09464801	12.6100738	1.8582E-36	2.5067E-35
TMEM74B	ENSG00000125895	protein_coding	336.850487	3.71798459	0.14467828	25.6982913	1.221E-145	7.272E-143
FAM110A	ENSG00000125898	protein_coding	648.179332	1.12242278	0.07754722	14.4740564	1.7673E-47	3.7665E-46
SIRPD	ENSG00000125900	protein_coding	8.18593868	3.14341591	0.22391341	14.0385338	9.0567E-45	1.7213E-43
S1PR4	ENSG00000125910	protein_coding	229.671618	1.8118296	0.11775712	15.3861572	2.0272E-53	5.4728E-52
C20orf173	ENSG00000125975	protein_coding	1.17674065	2.40877773	0.38437745	6.26669883	3.6878E-10	1.1265E-09
BPIFB1	ENSG00000125999	protein_coding	10.3799563	2.69002023	0.37107765	7.24921121	4.1921E-13	1.5805E-12
GRPR	ENSG00000126010	protein_coding	27.9719763	1.95138471	0.23190574	8.41455966	3.9437E-17	1.9414E-16
XRCC3	ENSG00000126215	protein_coding	396.698791	1.44695988	0.10481758	13.8045539	2.3925E-43	4.3077E-42
MCF2L	ENSG00000126217	protein_coding	4021.61567	1.14114866	0.10069194	11.3330683	8.9996E-30	8.8303E-29
F10	ENSG00000126218	protein_coding	627.441812	1.19775053	0.2272523	5.27057599	1.36E-07	3.3591E-07
SLURP1	ENSG00000126233	protein_coding	0.36071098	1.02917669	0.37469772	2.74668524	0.00602009	0.00928306
IGFLR1	ENSG00000126246	protein_coding	135.292204	1.64885825	0.13352653	12.3485446	4.9601E-35	6.2448E-34
GPR42	ENSG00000126251	protein_coding	1.52163643	2.7453724	0.39754689	6.90578261	4.9927E-12	1.747E-11
FFAR2	ENSG00000126262	protein_coding	29.9286743	1.76209167	0.16064074	10.9691458	5.3778E-28	4.8281E-27
HCST	ENSG00000126264	protein_coding	329.056429	2.01007687	0.14255875	14.0999896	3.7983E-45	7.3436E-44
KRT36	ENSG00000126337	protein_coding	7.10214256	3.72976869	0.2663261	14.0045184	1.4627E-44	2.7616E-43
CCR7	ENSG00000126353	protein_coding	214.981496	1.61851604	0.1575337	10.2740938	9.2207E-25	6.9601E-24
TSKS	ENSG00000126467	protein_coding	12.3679297	1.41939873	0.1749359	8.1138218	4.9052E-16	2.2513E-15

PRKCG	ENSG00000126583	protein_coding	5.9589842	2.42811138	0.31463502	7.71723175	1.1888E-14	4.9851E-14
SSX1	ENSG00000126752	protein_coding	2.45048376	2.26275161	0.59337847	3.8133362	0.0001371	0.00025438
CFP	ENSG00000126759	protein_coding	213.339498	1.30001123	0.11664499	11.1450243	7.5724E-29	7.1088E-28
SIX1	ENSG00000126778	protein_coding	131.815125	2.46034791	0.1632407	15.0719028	2.4787E-51	6.1787E-50
DLGAP5	ENSG00000126787	protein_coding	188.016849	2.71960498	0.15165816	17.9324676	6.5794E-72	3.3926E-70
PZP	ENSG00000126838	protein_coding	44.2597361	1.56184224	0.16096337	9.70309115	2.925E-22	1.9271E-21
EVI2A	ENSG00000126860	protein_coding	810.851811	1.86469805	0.12342986	15.1073499	1.4485E-51	3.6424E-50
OMG	ENSG00000126861	protein_coding	38.0514938	2.3174895	0.18807423	12.3222065	6.8786E-35	8.6107E-34
FAM78A	ENSG00000126882	protein_coding	855.939642	2.3586546	0.09876663	23.8810872	4.816E-126	1.611E-123
NXF5	ENSG00000126952	protein_coding	9.12280677	2.02200604	0.21325376	9.48168988	2.502E-21	1.5714E-20
RGS13	ENSG00000127074	protein_coding	25.2864384	1.83108529	0.16490794	11.1036816	1.2038E-28	1.1162E-27
BCL11B	ENSG00000127152	protein_coding	425.09361	1.5928001	0.13493917	11.803838	3.7291E-32	4.1004E-31
TRAF2	ENSG00000127191	protein_coding	1183.14352	1.03123587	0.07024926	14.6796686	8.7015E-49	1.9649E-47
MASP1	ENSG00000127241	protein_coding	2754.03175	1.97329835	0.16539878	11.9305493	8.203E-33	9.294E-32
IL22	ENSG00000127318	protein_coding	0.90508931	1.80461004	0.39697826	4.54586619	5.471E-06	1.1678E-05
TAS2R3	ENSG00000127362	protein_coding	3.38174107	1.24469106	0.22313885	5.57810103	2.4316E-08	6.3907E-08
TAS2R4	ENSG00000127364	protein_coding	37.5840048	1.45083742	0.15135117	9.58590186	9.1653E-22	5.896E-21
TAS2R5	ENSG00000127366	protein_coding	39.2360718	1.9219853	0.14016422	13.712382	8.5601E-43	1.5075E-41
IDUA	ENSG00000127415	protein_coding	777.855681	1.23464741	0.10341492	11.9387748	7.431E-33	8.4401E-32
PLA2G5	ENSG00000127472	protein_coding	47.1825572	2.7861614	0.2003331	13.9076435	5.6924E-44	1.0535E-42
ADGRE2	ENSG00000127507	protein_coding	676.588227	1.81159576	0.13191067	13.7335047	6.3963E-43	1.133E-41
F2RL3	ENSG00000127533	protein_coding	1748.04759	2.33283815	0.17191264	13.5699049	6.04E-42	1.0194E-40
PKMYT1	ENSG00000127564	protein_coding	137.876676	2.93399333	0.14126822	20.7689553	8.2636E-96	1.0581E-93
WFIKKN1	ENSG00000127578	protein_coding	205.43964	1.28096484	0.18083465	7.08362486	1.4043E-12	5.1185E-12
FBXL16	ENSG00000127585	protein_coding	2759.5793	1.5756379	0.15938846	9.88552044	4.8108E-23	3.3048E-22

CHTF18	ENSG00000127586	protein_coding	436.815305	1.53632767	0.11823155	12.9942271	1.3193E-38	1.9409E-37
GNG13	ENSG00000127588	protein_coding	0.61320378	1.31040347	0.33807202	3.87610746	0.00010614	0.00019932
GNGT1	ENSG00000127928	protein_coding	77.0402097	2.23277172	0.2521898	8.85353704	8.4789E-19	4.626E-18
STEAP4	ENSG00000127954	protein_coding	3005.95208	1.27141682	0.16127177	7.88369103	3.1785E-15	1.3841E-14
LRFN1	ENSG00000128011	protein_coding	111.080711	1.19048953	0.11317982	10.5185669	7.0944E-26	5.7036E-25
SPINK2	ENSG00000128040	protein_coding	14.8478352	1.43951859	0.29717825	4.84395672	1.2728E-06	2.8836E-06
KDR	ENSG00000128052	protein_coding	14102.297	1.12458962	0.13452435	8.35974768	6.2853E-17	3.0547E-16
GAL3ST1	ENSG00000128242	protein_coding	8473.30961	2.92327548	0.15293529	19.1144598	1.9139E-81	1.4015E-79
RFPL2	ENSG00000128253	protein_coding	11.2712325	1.47003492	0.20843674	7.05266687	1.7552E-12	6.35E-12
MGAT3	ENSG00000128268	protein_coding	1657.10768	1.27459714	0.16611822	7.67283155	1.6824E-14	6.9807E-14
ADORA2A	ENSG00000128271	protein_coding	62.7812213	1.38716749	0.11479895	12.0834508	1.2918E-33	1.5235E-32
RFPL3	ENSG00000128276	protein_coding	3.56690106	1.44913125	0.18690567	7.75327592	8.9552E-15	3.7884E-14
MCHR1	ENSG00000128285	protein_coding	693.243243	5.71412831	0.28596865	19.9816599	7.9535E-89	7.6275E-87
BAIAP2L2	ENSG00000128298	protein_coding	1436.66361	1.15682327	0.16769605	6.89833329	5.2616E-12	1.8378E-11
GALR3	ENSG00000128310	protein_coding	1.28860344	1.37159158	0.35796168	3.83167153	0.00012728	0.00023695
APOL5	ENSG00000128313	protein_coding	12.5904072	3.98044768	0.24577134	16.195736	5.4047E-59	1.776E-57
IGLL1	ENSG00000128322	protein_coding	4.0021331	1.53533268	0.28956948	5.30212189	1.1446E-07	2.846E-07
APOL2	ENSG00000128335	protein_coding	5737.02139	1.36865	0.11156038	12.2682443	1.3413E-34	1.6569E-33
RAC2	ENSG00000128340	protein_coding	2302.38811	2.07446636	0.13287876	15.6117227	6.0579E-55	1.7184E-53
APOBEC3A	ENSG00000128383	protein_coding	56.6977688	1.17430157	0.15481825	7.58503326	3.324E-14	1.3521E-13
APOBEC3F	ENSG00000128394	protein_coding	460.672208	1.09727625	0.06886976	15.9326268	3.7621E-57	1.1551E-55
KRT17	ENSG00000128422	protein_coding	341.64303	1.01261906	0.26157693	3.87120941	0.0001083	0.00020319
CPA4	ENSG00000128510	protein_coding	202.412516	2.64119713	0.30907417	8.54551231	1.2797E-17	6.4886E-17
CDHR3	ENSG00000128536	protein_coding	493.296067	1.40234528	0.10999393	12.7492965	3.1457E-37	4.3911E-36
LRRC17	ENSG00000128606	protein_coding	498.596638	1.61324248	0.13519125	11.9330395	7.9613E-33	9.0268E-32

HOXD13	ENSG00000128714	protein_coding	26.7702657	3.17098458	0.225996	14.0311533	1.005E-44	1.907E-43
ARHGAP22	ENSG00000128805	protein_coding	432.62949	2.7477062	0.10676099	25.7369862	4.509E-146	2.793E-143
WDFY4	ENSG00000128815	protein_coding	929.336476	1.7375292	0.13585386	12.7896933	1.8721E-37	2.6382E-36
DLL4	ENSG00000128917	protein_coding	5096.34512	2.41609707	0.12502763	19.3245045	3.3418E-83	2.579E-81
MYOD1	ENSG00000129152	protein_coding	2.20239066	3.6070511	0.43669735	8.25984199	1.4587E-16	6.9284E-16
KCNC1	ENSG00000129159	protein_coding	8.3056034	1.20267223	0.17266798	6.96523033	3.2787E-12	1.1622E-11
CSRP3	ENSG00000129170	protein_coding	1.44744025	2.70839092	0.37276842	7.26561265	3.7135E-13	1.4052E-12
E2F8	ENSG00000129173	protein_coding	114.517112	2.64281416	0.16431412	16.0839136	3.308E-58	1.056E-56
PIMREG	ENSG00000129195	protein_coding	115.41763	2.29712451	0.14901873	15.4150058	1.2976E-53	3.5281E-52
AIPL1	ENSG00000129221	protein_coding	1.86666523	2.49948114	0.38211075	6.54124786	6.1008E-11	1.9739E-10
CD68	ENSG00000129226	protein_coding	228.756162	3.15859394	0.14845111	21.276998	1.854E-100	2.7963E-98
KLK14	ENSG00000129437	protein_coding	11.0199285	1.97409153	0.21138947	9.33864653	9.7573E-21	5.9235E-20
SIGLEC9	ENSG00000129450	protein_coding	283.161836	2.30052256	0.11635325	19.7718807	5.2001E-87	4.6649E-85
EGLN3	ENSG00000129521	protein_coding	29590.0663	4.24159398	0.1313917	32.2820542	1.249E-228	5.207E-225
RHBDF2	ENSG00000129667	protein_coding	2956.9943	1.9445136	0.07996756	24.3162806	1.319E-130	5.08E-128
AANAT	ENSG00000129673	protein_coding	19.1420926	2.62286876	0.17333412	15.1318666	9.9823E-52	2.5282E-50
ARHGEF6	ENSG00000129675	protein_coding	2729.04659	1.15382256	0.07474092	15.4376276	9.1401E-54	2.4955E-52
SGO1	ENSG00000129810	protein_coding	56.0676402	1.037296	0.1327003	7.81683218	5.4169E-15	2.3257E-14
CDH15	ENSG00000129910	protein_coding	29.8551793	1.27126196	0.26154883	4.86051478	1.1708E-06	2.6608E-06
SHC2	ENSG00000129946	protein_coding	1284.83255	1.31101247	0.12631849	10.378627	3.102E-25	2.4069E-24
PLPPR3	ENSG00000129951	protein_coding	8.4473961	1.8647468	0.26550938	7.02328029	2.1672E-12	7.7864E-12
LBP	ENSG00000129988	protein_coding	1409.49944	3.39956878	0.37359503	9.09960934	9.0657E-20	5.2183E-19
SYT5	ENSG00000129990	protein_coding	12.5321051	1.66806666	0.22235156	7.50193367	6.2883E-14	2.5156E-13
TNNI3	ENSG00000129991	protein_coding	11.4719646	2.50455047	0.41378739	6.05274717	1.424E-09	4.1516E-09
CBFA2T3	ENSG00000129993	protein_coding	229.028121	1.033501	0.10776763	9.59008772	8.801E-22	5.6632E-21

CRACR2A	ENSG00000130038	protein_coding	257.667848	2.15312438	0.11610135	18.5452137	8.9146E-77	5.6134E-75
NXNL2	ENSG00000130045	protein_coding	356.300123	1.08468263	0.14086438	7.70019081	1.3586E-14	5.6691E-14
DOCK6	ENSG00000130158	protein_coding	5430.35088	1.17016159	0.0859015	13.6221321	2.958E-42	5.0691E-41
TSPAN16	ENSG00000130167	protein_coding	5.25731462	2.60704487	0.26500206	9.8378288	7.7362E-23	5.2539E-22
ANGPTL8	ENSG00000130173	protein_coding	66.976791	3.24439166	0.28706335	11.3020058	1.2825E-29	1.2507E-28
ZSCAN10	ENSG00000130182	protein_coding	1.93243929	2.11084884	0.36035699	5.85766025	4.6943E-09	1.311E-08
APOC1	ENSG00000130208	protein_coding	5901.84452	5.20854927	0.19078208	27.3010406	4.123E-164	4.021E-161
RPL36	ENSG00000130255	protein_coding	20501.129	1.25886369	0.104405	12.0575041	1.7707E-33	2.0717E-32
ATP8B3	ENSG00000130270	protein_coding	633.031713	4.22179252	0.20804492	20.2926971	1.4918E-91	1.583E-89
NCAN	ENSG00000130287	protein_coding	5.30279452	1.83120239	0.25624153	7.14639198	8.9088E-13	3.29E-12
PLVAP	ENSG00000130300	protein_coding	43062.4696	1.93339662	0.12593988	15.351742	3.448E-53	9.2325E-52
BST2	ENSG00000130303	protein_coding	8303.23617	1.32301038	0.11541408	11.463163	2.02E-30	2.041E-29
COLGALT1	ENSG00000130309	protein_coding	7579.7691	1.71378977	0.0728318	23.5307887	1.975E-122	5.917E-120
EPO	ENSG00000130427	protein_coding	542.7597	4.91086011	0.35877657	13.6877948	1.201E-42	2.0965E-41
ARPC1B	ENSG00000130429	protein_coding	7446.65678	1.02518834	0.09368925	10.9424332	7.2235E-28	6.4473E-27
FCHO1	ENSG00000130475	protein_coding	337.184107	2.62522033	0.14946654	17.5639332	4.6536E-69	2.1614E-67
PXDN	ENSG00000130508	protein_coding	7802.22704	1.66556974	0.12453614	13.374188	8.5583E-41	1.3761E-39
SULT4A1	ENSG00000130540	protein_coding	47.2020937	2.52231257	0.31975189	7.88834299	3.0623E-15	1.3356E-14
SAG	ENSG00000130561	protein_coding	5.20666502	2.3336874	0.28169712	8.28438494	1.1872E-16	5.672E-16
LSP1	ENSG00000130592	protein_coding	2891.01955	2.24044766	0.12622445	17.7497119	1.7324E-70	8.4936E-69
TNNT3	ENSG00000130595	protein_coding	56.2991084	2.34107517	0.26520729	8.8273408	1.0719E-18	5.8111E-18
TNNI2	ENSG00000130598	protein_coding	40.9496423	2.4407336	0.15572758	15.673098	2.3104E-55	6.6485E-54
COL5A1	ENSG00000130635	protein_coding	7192.07039	2.02519097	0.1811324	11.1807217	5.0676E-29	4.8027E-28
CALY	ENSG00000130643	protein_coding	7.53612497	2.17030498	0.27525131	7.88481249	3.1501E-15	1.3725E-14
PNPLA7	ENSG00000130653	protein_coding	895.287523	1.53663891	0.14139245	10.8678989	1.6393E-27	1.4336E-26

MNX1	ENSG00000130675	protein_coding	32.2746801	2.49887028	0.27100558	9.22073378	2.9507E-20	1.7427E-19
ZNF337	ENSG00000130684	protein_coding	220.590713	1.28332811	0.09688964	13.2452562	4.8064E-40	7.4891E-39
PRDM12	ENSG00000130711	protein_coding	3.14935569	1.14299241	0.22668174	5.04227824	4.6002E-07	1.0866E-06
FIBCD1	ENSG00000130720	protein_coding	184.833137	4.38573807	0.29659069	14.7871739	1.7724E-49	4.0993E-48
METTL26	ENSG00000130731	protein_coding	4022.32938	1.57691025	0.11346213	13.8981196	6.5027E-44	1.1967E-42
GMFG	ENSG00000130755	protein_coding	1950.753	1.83884291	0.0936111	19.6434285	6.5802E-86	5.6914E-84
THEMIS2	ENSG00000130775	protein_coding	2133.75237	2.05356493	0.10876062	18.8815116	1.6189E-79	1.1109E-77
SLC6A8	ENSG00000130821	protein_coding	24195.9369	1.62986106	0.13358792	12.2006619	3.0831E-34	3.7409E-33
PNCK	ENSG00000130822	protein_coding	6535.51027	6.61560797	0.25446614	25.9979896	5.218E-149	3.624E-146
PLXNA3	ENSG00000130827	protein_coding	2304.91084	1.34365122	0.09488518	14.1608125	1.6013E-45	3.1464E-44
HSD17B3	ENSG00000130948	protein_coding	78.0891808	3.03844193	0.20564027	14.7755197	2.1073E-49	4.8664E-48
ULBP2	ENSG00000131015	protein_coding	84.5348406	1.03455935	0.11943037	8.66244774	4.6174E-18	2.4089E-17
LILRB2	ENSG00000131042	protein_coding	821.892842	2.27349193	0.10945682	20.7706745	7.9731E-96	1.0238E-93
COX4I2	ENSG00000131055	protein_coding	711.025739	2.86641841	0.17343583	16.5272566	2.3353E-61	8.2413E-60
DEFB118	ENSG00000131068	protein_coding	1.16831399	2.71382329	0.49099232	5.52722144	3.2534E-08	8.4628E-08
EDA2R	ENSG00000131080	protein_coding	1000.28343	2.2815041	0.09166781	24.8888243	9.831E-137	4.552E-134
C1QL1	ENSG00000131094	protein_coding	1883.2321	3.67994185	0.26860205	13.7003491	1.0104E-42	1.7712E-41
GFAP	ENSG00000131095	protein_coding	125.828819	1.39385968	0.19146094	7.2801257	3.3351E-13	1.2669E-12
HIGD1B	ENSG00000131097	protein_coding	297.771728	1.68025297	0.14442626	11.6339853	2.7686E-31	2.9223E-30
TEX101	ENSG00000131126	protein_coding	4.47735215	1.99680302	0.38273176	5.21723889	1.8161E-07	4.4434E-07
CCL25	ENSG00000131142	protein_coding	4.99648315	3.12925005	0.33406068	9.36731043	7.4404E-21	4.5434E-20
GINS2	ENSG00000131153	protein_coding	339.218807	1.90850471	0.108223	17.6349265	1.3287E-69	6.2987E-68
F12	ENSG00000131187	protein_coding	301.793312	1.5708119	0.20095251	7.81683137	5.417E-15	2.3257E-14
PRR7	ENSG00000131188	protein_coding	166.525298	2.37948583	0.15178755	15.6764231	2.1926E-55	6.3174E-54
IDO1	ENSG00000131203	protein_coding	2056.15	3.94755403	0.16530491	23.8804404	4.891E-126	1.625E-123

ADGRE3	ENSG00000131355	protein_coding	28.9657116	1.19165903	0.16320247	7.30172179	2.8411E-13	1.084E-12
LRRC4B	ENSG00000131409	protein_coding	180.052629	1.01250391	0.12770243	7.92861879	2.216E-15	9.7675E-15
ACLY	ENSG00000131473	protein_coding	24331.3506	1.34145495	0.0825603	16.248184	2.3008E-59	7.6483E-58
ANKHD1	ENSG00000131503	protein_coding	443.421095	1.00589472	0.07828527	12.8490939	8.7024E-38	1.2416E-36
ANO1	ENSG00000131620	protein_coding	2927.41814	1.82393426	0.12464791	14.6326905	1.7378E-48	3.8765E-47
KREMEN2	ENSG00000131650	protein_coding	21.7721352	2.79684759	0.21893882	12.7745621	2.2742E-37	3.195E-36
THOC6	ENSG00000131652	protein_coding	1472.48065	1.09471381	0.0791932	13.8233315	1.8434E-43	3.3374E-42
BARX1	ENSG00000131668	protein_coding	9.3598769	3.60486825	0.38231724	9.42899746	4.1403E-21	2.5686E-20
CA6	ENSG00000131686	protein_coding	2.3923445	2.87909752	0.29999239	9.59723527	8.2118E-22	5.2944E-21
RHOXF2	ENSG00000131721	protein_coding	0.7463691	2.01403709	0.67125519	3.0004045	0.00269621	0.00433628
KRT34	ENSG00000131737	protein_coding	2.97513187	1.84896085	0.43096081	4.29032247	1.7841E-05	3.6237E-05
KRT33B	ENSG00000131738	protein_coding	3.87299215	2.38620488	0.35394468	6.74174534	1.565E-11	5.2874E-11
TOP2A	ENSG00000131747	protein_coding	1287.06184	2.31186248	0.14391866	16.0636743	4.5856E-58	1.4608E-56
RARA	ENSG00000131759	protein_coding	3885.73943	1.03674514	0.08402162	12.339028	5.5826E-35	7.0074E-34
USP29	ENSG00000131864	protein_coding	0.85801814	1.95170636	0.41785373	4.6707884	3.0005E-06	6.5672E-06
LIN28A	ENSG00000131914	protein_coding	2.32791143	1.69453145	0.37406426	4.53005443	5.8968E-06	1.255E-05
LGALS3	ENSG00000131981	protein_coding	13287.9405	1.34269568	0.14103896	9.52003374	1.7312E-21	1.0969E-20
PODNL1	ENSG00000132000	protein_coding	170.100857	2.56431045	0.23507248	10.908595	1.0486E-27	9.2855E-27
RTBDN	ENSG00000132026	protein_coding	4.28814191	1.9150221	0.35690168	5.36568536	8.0642E-08	2.0336E-07
LRRC41	ENSG00000132128	protein_coding	9584.00319	1.10570242	0.10986755	10.0639585	7.9727E-24	5.7123E-23
HSD17B7	ENSG00000132196	protein_coding	762.990507	1.6362874	0.11166206	14.6539248	1.2715E-48	2.8461E-47
EMILIN2	ENSG00000132205	protein_coding	1252.3364	1.6716713	0.09900892	16.8840467	5.8961E-64	2.2713E-62
SLX1A	ENSG00000132207	protein_coding	1.95247302	2.07195261	0.31148808	6.65178781	2.8955E-11	9.6031E-11
TRIM22	ENSG00000132274	protein_coding	5673.60923	1.36480865	0.08846355	15.4279203	1.0624E-53	2.8954E-52
HHLA1	ENSG00000132297	protein_coding	0.63568678	1.35422949	0.4003007	3.38303048	0.00071691	0.00122896

PER2	ENSG00000132326	protein_coding	2591.23282	1.13130713	0.09224625	12.2639903	1.4136E-34	1.7439E-33
PTPRE	ENSG00000132334	protein_coding	2217.02184	1.658037	0.07434644	22.301497	3.573E-110	7.584E-108
SERPINF1	ENSG00000132386	protein_coding	3310.54172	1.05721328	0.18623348	5.67681655	1.3722E-08	3.6856E-08
SEC61G	ENSG00000132432	protein_coding	3088.95106	1.11762734	0.07728904	14.4603592	2.1567E-47	4.5792E-46
JCHAIN	ENSG00000132465	protein_coding	7559.49539	1.26262555	0.25357582	4.97928216	6.3821E-07	1.4885E-06
GUCY2D	ENSG00000132518	protein_coding	32.0116645	1.51945404	0.2216461	6.85531588	7.1155E-12	2.4657E-11
GPS2	ENSG00000132522	protein_coding	300.833519	1.46641548	0.12475742	11.7541342	6.7246E-32	7.2997E-31
XAF1	ENSG00000132530	protein_coding	832.180208	1.96666484	0.1359868	14.4621746	2.1005E-47	4.4621E-46
SNAP25	ENSG00000132639	protein_coding	456.92366	1.99374345	0.16471854	12.1039407	1.0066E-33	1.192E-32
BCAN	ENSG00000132692	protein_coding	123.36591	1.76738311	0.13801562	12.8056748	1.524E-37	2.1555E-36
CRP	ENSG00000132693	protein_coding	89.5872442	4.39478961	0.37281339	11.7881754	4.4917E-32	4.9188E-31
HAPLN2	ENSG00000132702	protein_coding	36.0118708	1.15434729	0.16643748	6.93562097	4.0444E-12	1.4248E-11
FCRL2	ENSG00000132704	protein_coding	21.1748008	1.3939593	0.25903249	5.38140708	7.3906E-08	1.8705E-07
LPIN3	ENSG00000132793	protein_coding	2394.79453	1.54245366	0.11791559	13.0809984	4.2285E-39	6.3784E-38
PDE6A	ENSG00000132915	protein_coding	9.84818324	1.59182471	0.19092183	8.33757315	7.5831E-17	3.6692E-16
TPTE2	ENSG00000132958	protein_coding	8.59261362	1.26350531	0.2376616	5.31640494	1.0584E-07	2.6391E-07
ALOX5AP	ENSG00000132965	protein_coding	810.41641	1.18622167	0.12775333	9.2852506	1.6133E-20	9.6976E-20
CHIT1	ENSG00000133063	protein_coding	877.968513	5.05285757	0.29441719	17.1622371	5.0914E-66	2.116E-64
DCLK1	ENSG00000133083	protein_coding	1670.87778	2.1391867	0.15735014	13.5950732	4.2833E-42	7.2723E-41
EPSTI1	ENSG00000133106	protein_coding	1641.64632	1.0921644	0.10814153	10.099398	5.5582E-24	4.0169E-23
POSTN	ENSG00000133110	protein_coding	6928.77767	1.54417938	0.19460541	7.93492537	2.1062E-15	9.2963E-15
STOML3	ENSG00000133115	protein_coding	8.27557343	1.68649184	0.28139452	5.99333577	2.0558E-09	5.9181E-09
PRAM1	ENSG00000133246	protein_coding	250.599503	2.44663257	0.1247762	19.6081666	1.3169E-85	1.1221E-83
KMT5C	ENSG00000133247	protein_coding	385.817838	1.26568778	0.10443655	12.119203	8.3568E-34	9.9528E-33
LGALS12	ENSG00000133317	protein_coding	284.234275	4.1549205	0.26180261	15.870432	1.0154E-56	3.0563E-55

PLAAT4	ENSG00000133321	protein_coding	5954.14398	1.28362634	0.13583096	9.45017483	3.3827E-21	2.1071E-20
C1QTNF6	ENSG00000133466	protein_coding	1256.24371	2.27339374	0.11099089	20.4827049	3.0712E-93	3.5642E-91
GGT2	ENSG00000133475	protein_coding	52.6361037	1.53373617	0.23776966	6.45051262	1.1147E-10	3.5354E-10
GIMAP4	ENSG00000133574	protein_coding	4744.38771	1.29639154	0.08616706	15.0450949	3.718E-51	9.2078E-50
KRBA1	ENSG00000133619	protein_coding	3489.17889	2.7839468	0.1138639	24.4497748	5.06E-132	2.04E-129
SPINK5	ENSG00000133710	protein_coding	216.779602	2.12157262	0.21400105	9.91384221	3.6243E-23	2.504E-22
CA1	ENSG00000133742	protein_coding	33.9390283	3.18515741	0.29462089	10.8110373	3.052E-27	2.6397E-26
TEX15	ENSG00000133863	protein_coding	350.183433	5.75577558	0.31809972	18.094249	3.5373E-73	1.9167E-71
GSC	ENSG00000133937	protein_coding	14.5939968	1.89002952	0.17764139	10.6395785	1.9501E-26	1.618E-25
LOXL2	ENSG00000134013	protein_coding	6883.87706	2.67701216	0.15474504	17.2995019	4.7447E-67	2.0461E-65
ADAMDEC1	ENSG00000134028	protein_coding	335.824541	4.27394791	0.24213598	17.6510234	9.9929E-70	4.7717E-68
CD180	ENSG00000134061	protein_coding	423.603552	2.11872169	0.14112065	15.0135483	5.9858E-51	1.4705E-49
PTPN22	ENSG00000134242	protein_coding	324.812256	2.05770212	0.13235214	15.5471768	1.6628E-54	4.6449E-53
WNT2B	ENSG00000134245	protein_coding	591.532269	1.20326979	0.18546807	6.4877465	8.713E-11	2.7888E-10
CD101	ENSG00000134256	protein_coding	133.695484	1.4560607	0.11641442	12.5075633	6.7875E-36	8.9051E-35
NGF	ENSG00000134259	protein_coding	415.840107	2.11914188	0.14530014	14.5845829	3.5207E-48	7.7332E-47
FKBP11	ENSG00000134285	protein_coding	1398.29195	1.95205205	0.12066316	16.1776964	7.2455E-59	2.3657E-57
LDHA	ENSG00000134333	protein_coding	94559.938	1.6734097	0.08123237	20.6002809	2.7286E-94	3.2829E-92
SAA2	ENSG00000134339	protein_coding	392.477911	3.97800022	0.37302408	10.6641914	1.497E-26	1.2499E-25
CFHR5	ENSG00000134389	protein_coding	0.64943386	1.7006982	0.54667832	3.11096697	0.00186476	0.00305196
ERN2	ENSG00000134398	protein_coding	8.22234947	1.37794172	0.29251701	4.71063793	2.4694E-06	5.4444E-06
IL2RA	ENSG00000134460	protein_coding	266.35222	2.20553121	0.18981519	11.6193608	3.2859E-31	3.4516E-30
IL15RA	ENSG00000134470	protein_coding	1133.25341	1.46583899	0.09501363	15.4276699	1.0665E-53	2.9049E-52
HRH4	ENSG00000134489	protein_coding	6.97273198	1.19659709	0.1503312	7.95973876	1.724E-15	7.6544E-15
DOCK2	ENSG00000134516	protein_coding	2058.15691	2.24088202	0.13367695	16.7634134	4.5192E-63	1.6856E-61

KLRD1	ENSG00000134539	protein_coding	393.224901	2.3197934	0.11322453	20.4884349	2.7303E-93	3.1847E-91
KLRC1	ENSG00000134545	protein_coding	33.2851807	1.91183343	0.16695122	11.4514494	2.3125E-30	2.3313E-29
MYBPC3	ENSG00000134571	protein_coding	14.3817828	1.54101398	0.19620615	7.8540556	4.028E-15	1.7433E-14
DDB2	ENSG00000134574	protein_coding	2853.17359	2.01740903	0.06686513	30.1713178	5.635E-200	1.435E-196
RAB33A	ENSG00000134594	protein_coding	103.459385	2.13938796	0.14979653	14.281959	2.8352E-46	5.7432E-45
SOX3	ENSG00000134595	protein_coding	2.13130605	1.25840019	0.34665579	3.63011442	0.0002833	0.00050764
PIWIL4	ENSG00000134627	protein_coding	211.808689	1.29898555	0.10176131	12.7650242	2.5707E-37	3.6027E-36
SPOCD1	ENSG00000134668	protein_coding	71.0594401	1.74775178	0.19969921	8.75192121	2.0975E-18	1.1161E-17
CDCA8	ENSG00000134690	protein_coding	240.638188	1.46922086	0.12519867	11.7351152	8.4212E-32	9.1024E-31
CYP2J2	ENSG00000134716	protein_coding	7926.80966	5.11050885	0.20790362	24.5811442	2.01E-133	8.531E-131
DSG3	ENSG00000134757	protein_coding	5.3050641	2.34579986	0.40176444	5.8387444	5.2596E-09	1.4631E-08
DSG1	ENSG00000134760	protein_coding	4.6739927	1.13048241	0.26390666	4.28364484	1.8386E-05	3.7308E-05
SLC43A3	ENSG00000134802	protein_coding	1917.29545	1.60267493	0.1085967	14.7580441	2.7309E-49	6.2876E-48
CBLIF	ENSG00000134812	protein_coding	2.77743605	1.29833417	0.33921614	3.82745402	0.00012948	0.0002408
APLNR	ENSG00000134817	protein_coding	3995.8971	1.32808381	0.16042273	8.27865105	1.2458E-16	5.9475E-16
TCN1	ENSG00000134827	protein_coding	22.8556933	2.55761653	0.33608528	7.61002252	2.7405E-14	1.1205E-13
COL4A2	ENSG00000134871	protein_coding	66995.886	1.52014223	0.10756495	14.1323199	2.401E-45	4.6836E-44
POGLUT2	ENSG00000134901	protein_coding	938.196662	1.21690217	0.09437504	12.8943224	4.845E-38	6.9887E-37
ACRV1	ENSG00000134940	protein_coding	7.94764536	1.90771392	0.16885124	11.2981931	1.3395E-29	1.3048E-28
ETS1	ENSG00000134954	protein_coding	17261.3821	1.13586003	0.09505678	11.9492801	6.549E-33	7.4569E-32
SLC37A2	ENSG00000134955	protein_coding	1077.54645	2.72548597	0.12704693	21.452592	4.319E-102	6.996E-100
ANXA1	ENSG00000135046	protein_coding	10491.6537	1.32370365	0.09890294	13.3838649	7.5136E-41	1.2132E-39
CEMIP2	ENSG00000135048	protein_coding	7215.352	1.22881776	0.07867475	15.61896	5.4081E-55	1.5389E-53
ADAM19	ENSG00000135074	protein_coding	1002.81183	1.57708132	0.12126279	13.0054845	1.1387E-38	1.678E-37
HAVCR2	ENSG00000135077	protein_coding	4695.94009	1.81712641	0.16852891	10.7822831	4.174E-27	3.5845E-26

SDS	ENSG00000135094	protein_coding	768.19253	4.24293413	0.17213497	24.6488788	3.783E-134	1.621E-131
OASL	ENSG00000135114	protein_coding	597.270534	1.57047042	0.14201791	11.058256	1.9995E-28	1.8317E-27
OCM2	ENSG00000135175	protein_coding	0.49108357	1.58518735	0.49604119	3.19567688	0.00139503	0.00231685
CCDC146	ENSG00000135205	protein_coding	4315.00927	1.96122254	0.22529299	8.70520904	3.1699E-18	1.6702E-17
CD36	ENSG00000135218	protein_coding	8515.4542	2.16728677	0.15541867	13.9448292	3.3826E-44	6.3007E-43
HILPDA	ENSG00000135245	protein_coding	11830.3921	4.7365178	0.14861323	31.8714418	6.643E-223	2.538E-219
PRPH	ENSG00000135406	protein_coding	82.0957755	1.93678471	0.25849148	7.49264431	6.75E-14	2.6953E-13
TESPA1	ENSG00000135426	protein_coding	285.244897	2.44173836	0.15106317	16.1636902	9.095E-59	2.9548E-57
RDH5	ENSG00000135437	protein_coding	339.264892	1.21929667	0.13211216	9.22925407	2.7252E-20	1.6138E-19
AGAP2	ENSG00000135439	protein_coding	395.179047	2.68973731	0.10539037	25.52166	1.133E-143	6.042E-141
TROAP	ENSG00000135451	protein_coding	175.217028	3.23883772	0.16448675	19.6905697	2.5975E-86	2.2855E-84
B4GALNT1	ENSG00000135454	protein_coding	860.731324	3.98023464	0.24496567	16.2481326	2.3027E-59	7.6492E-58
SLC26A10	ENSG00000135502	protein_coding	29.5706425	2.76149537	0.21792232	12.6719256	8.4612E-37	1.1595E-35
MIP	ENSG00000135517	protein_coding	6.39325233	1.13293922	0.17219895	6.57924583	4.7284E-11	1.5436E-10
HEY2	ENSG00000135547	protein_coding	746.369063	1.28239417	0.11698791	10.9617671	5.8349E-28	5.2272E-27
PKIB	ENSG00000135549	protein_coding	289.448549	1.05706013	0.1482326	7.13109061	9.9577E-13	3.6632E-12
TAAR5	ENSG00000135569	protein_coding	0.61699219	1.97853008	0.53144077	3.722955	0.0001969	0.00035928
MICAL1	ENSG00000135596	protein_coding	2422.78155	1.46092757	0.09406176	15.5315774	2.121E-54	5.8962E-53
DYSF	ENSG00000135636	protein_coding	8530.72531	1.4663792	0.09952863	14.7332405	3.9433E-49	9.0113E-48
CCDC142	ENSG00000135637	protein_coding	411.682229	1.07043806	0.07504434	14.2640744	3.6642E-46	7.3964E-45
BCO1	ENSG00000135697	protein_coding	561.108892	1.53980314	0.15067372	10.2194542	1.6225E-24	1.2088E-23
FBXL8	ENSG00000135722	protein_coding	443.559404	1.90264676	0.09836454	19.3428113	2.3434E-83	1.8239E-81
FHOD1	ENSG00000135723	protein_coding	2051.78596	1.25264078	0.07533286	16.6280804	4.3635E-62	1.5725E-60
SLC9A5	ENSG00000135740	protein_coding	111.934855	1.7583388	0.12065676	14.5730654	4.1676E-48	9.1411E-47
AGT	ENSG00000135744	protein_coding	6442.33851	1.06822947	0.17788518	6.00516284	1.9114E-09	5.5163E-09

GPR55	ENSG00000135898	protein_coding	45.0123883	1.82016512	0.14870572	12.2400477	1.8992E-34	2.3223E-33
CHRND	ENSG00000135902	protein_coding	8.80414096	3.44490486	0.29018945	11.8712271	1.67E-32	1.8631E-31
DOCK10	ENSG00000135905	protein_coding	2017.71255	1.41043743	0.09823059	14.3584332	9.4336E-47	1.9532E-45
SERPINE2	ENSG00000135919	protein_coding	8335.76247	1.2951721	0.16099467	8.04481362	8.6377E-16	3.9034E-15
GPR45	ENSG00000135973	protein_coding	6.78436036	1.48584959	0.22301093	6.66267602	2.6889E-11	8.9384E-11
ANKRD36	ENSG00000135976	protein_coding	245.438808	1.71583461	0.1474565	11.6362091	2.6974E-31	2.8485E-30
ALDH1L2	ENSG00000136010	protein_coding	397.192878	1.30890168	0.15487894	8.45112782	2.885E-17	1.4333E-16
STAB2	ENSG00000136011	protein_coding	59.3668501	1.35762242	0.19183739	7.07694381	1.4737E-12	5.3641E-12
PLXNC1	ENSG00000136040	protein_coding	1817.42123	1.48842559	0.11672951	12.7510647	3.0752E-37	4.2952E-36
SLC41A2	ENSG00000136052	protein_coding	2288.16254	1.21515821	0.08259998	14.7113615	5.45E-49	1.2374E-47
PCDH8	ENSG00000136099	protein_coding	1.30738941	1.54004812	0.47147743	3.26643021	0.00108913	0.00183177
CNMD	ENSG00000136110	protein_coding	8.74622243	1.96426021	0.33244662	5.90849801	3.4524E-09	9.747E-09
LCP1	ENSG00000136167	protein_coding	8883.88317	1.80971481	0.12277141	14.7405228	3.5403E-49	8.1146E-48
SPDYE1	ENSG00000136206	protein_coding	18.8795545	1.31254262	0.15562274	8.43413138	3.3367E-17	1.6504E-16
IGF2BP3	ENSG00000136231	protein_coding	147.180304	3.72548635	0.29205043	12.7563118	2.8749E-37	4.0217E-36
GPNMB	ENSG00000136235	protein_coding	18301.8912	1.75645568	0.19254866	9.12213917	7.3656E-20	4.2713E-19
AOAH	ENSG00000136250	protein_coding	1223.42104	2.16556417	0.12788385	16.9338369	2.5332E-64	9.8996E-63
CCM2	ENSG00000136280	protein_coding	2938.26172	1.11798077	0.07092754	15.7622943	5.6543E-56	1.6605E-54
MYO1G	ENSG00000136286	protein_coding	983.624406	2.63247143	0.13614444	19.3358714	2.681E-83	2.0795E-81
TTYH3	ENSG00000136295	protein_coding	14860.7719	1.91684825	0.11176429	17.1508109	6.1981E-66	2.5667E-64
NKX2-8	ENSG00000136327	protein_coding	2.25486601	2.28362223	0.57392047	3.97898723	6.9209E-05	0.00013235
NKX2-1	ENSG00000136352	protein_coding	0.87577968	1.48173995	0.56557991	2.6198596	0.0087966	0.01326943
ADAMTS7	ENSG00000136378	protein_coding	749.398203	2.43106	0.11397218	21.3302937	5.943E-101	9.267E-99
TM6SF1	ENSG00000136404	protein_coding	295.573597	1.00085551	0.11433753	8.75351719	2.068E-18	1.1009E-17
LIMD2	ENSG00000136490	protein_coding	1267.07353	1.99471333	0.12430253	16.0472462	5.9757E-58	1.8931E-56

BRIP1	ENSG00000136492	protein_coding	180.094363	1.80359247	0.12211527	14.7695904	2.3011E-49	5.3087E-48
RTP4	ENSG00000136514	protein_coding	917.478673	1.73960485	0.11565018	15.0419557	3.8986E-51	9.6447E-50
TBR1	ENSG00000136535	protein_coding	2.00456201	1.54369784	0.30043987	5.13812584	2.7749E-07	6.6823E-07
ERMN	ENSG00000136541	protein_coding	34.0413582	2.04666257	0.17634645	11.605919	3.8453E-31	4.019E-30
GALNT5	ENSG00000136542	protein_coding	267.254126	1.78099017	0.29211849	6.09680737	1.0821E-09	3.1869E-09
GATA4	ENSG00000136574	protein_coding	7.80242506	3.35268601	0.43699114	7.67220598	1.6906E-14	7.0123E-14
HLX	ENSG00000136630	protein_coding	1292.62482	2.53276931	0.12393255	20.4366753	7.8937E-93	9.0238E-91
IL10	ENSG00000136634	protein_coding	52.9204937	1.31060734	0.16141458	8.11951047	4.6807E-16	2.1513E-15
IL36B	ENSG00000136696	protein_coding	1.12571642	1.80808678	0.50830473	3.55709219	0.00037498	0.00066313
GYPC	ENSG00000136732	protein_coding	3543.94351	1.02811683	0.08352756	12.3087135	8.1309E-35	1.014E-33
GAD2	ENSG00000136750	protein_coding	3.57271628	2.83187724	0.32526367	8.7064051	3.1366E-18	1.6535E-17
OR1J1	ENSG00000136834	protein_coding	1.08096147	1.32325188	0.39910379	3.31555827	0.0009146	0.00154984
ANGPTL2	ENSG00000136859	protein_coding	5374.71035	1.41697825	0.12977808	10.9184714	9.4066E-28	8.3405E-27
BAAT	ENSG00000136881	protein_coding	108.971406	3.20267291	0.26718614	11.9866732	4.1733E-33	4.8031E-32
WDR38	ENSG00000136918	protein_coding	11.296242	1.09185789	0.17987692	6.07002775	1.2789E-09	3.7408E-09
GABBR2	ENSG00000136928	protein_coding	33.4147979	1.82874188	0.22774263	8.02986192	9.7582E-16	4.3959E-15
HEMGN	ENSG00000136929	protein_coding	9.52801195	1.39879233	0.26784862	5.2223241	1.7669E-07	4.3272E-07
NR5A1	ENSG00000136931	protein_coding	2.7468242	2.32953607	0.39208501	5.94140564	2.8259E-09	8.0391E-09
RPL35	ENSG00000136942	protein_coding	21329.6137	1.12381197	0.10829336	10.3774784	3.1396E-25	2.4356E-24
ENPP2	ENSG00000136960	protein_coding	24733.157	1.55731791	0.16835223	9.25035498	2.2375E-20	1.3319E-19
MYC	ENSG00000136997	protein_coding	5620.03674	1.50994941	0.12082136	12.4973712	7.7161E-36	1.0095E-34
SIT1	ENSG00000137078	protein_coding	242.935025	2.69529991	0.17166232	15.7011737	1.4847E-55	4.3076E-54
DMRT1	ENSG00000137090	protein_coding	2.34502474	3.14181579	0.41705987	7.5332489	4.9493E-14	1.9925E-13
CD72	ENSG00000137101	protein_coding	419.476057	2.71063029	0.13851518	19.5691929	2.8313E-85	2.3815E-83
ARHGEF39	ENSG00000137135	protein_coding	335.657211	2.70878238	0.09921591	27.3018956	4.027E-164	4.013E-161

CAPN11	ENSG00000137225	protein_coding	120.120308	3.33088847	0.17963328	18.5427141	9.3388E-77	5.8644E-75
KIAA0319	ENSG00000137261	protein_coding	138.624666	2.84451623	0.19316671	14.7257065	4.4084E-49	1.0054E-47
IRF4	ENSG00000137265	protein_coding	236.55286	1.97107133	0.18972198	10.3892617	2.7749E-25	2.1601E-24
FOXF2	ENSG00000137273	protein_coding	43.3702048	1.68580654	0.17577616	9.59064404	8.7537E-22	5.6336E-21
TCF19	ENSG00000137310	protein_coding	1570.74699	1.31068827	0.11626465	11.2733175	1.7774E-29	1.7233E-28
NRM	ENSG00000137404	protein_coding	1128.51077	1.07979932	0.08825293	12.2352801	2.0141E-34	2.4614E-33
FGFBP2	ENSG00000137441	protein_coding	137.219991	1.86731128	0.1505575	12.4026452	2.5284E-35	3.2384E-34
TLR2	ENSG00000137462	protein_coding	1631.28527	1.59035126	0.11120392	14.3012164	2.1502E-46	4.3885E-45
MGARP	ENSG00000137463	protein_coding	419.909839	2.96481732	0.25223839	11.7540291	6.733E-32	7.307E-31
SLCO2B1	ENSG00000137491	protein_coding	6289.82223	1.55669647	0.12635607	12.3199187	7.0766E-35	8.8488E-34
IL18BP	ENSG00000137496	protein_coding	1922.56595	1.51185704	0.09469223	15.9660095	2.2045E-57	6.8373E-56
SLCO5A1	ENSG00000137571	protein_coding	39.7565463	1.21442528	0.17680188	6.86884814	6.4722E-12	2.2484E-11
SULF1	ENSG00000137573	protein_coding	6736.31397	1.08087017	0.14484548	7.46222913	8.5071E-14	3.3697E-13
BTG4	ENSG00000137707	protein_coding	1.24501021	1.71988978	0.37719755	4.55965257	5.1238E-06	1.0966E-05
MMP13	ENSG00000137745	protein_coding	27.2684483	4.56669013	0.45845235	9.96110095	2.2555E-23	1.5759E-22
CASP1	ENSG00000137752	protein_coding	1640.67611	1.72961424	0.08661989	19.9678647	1.0484E-88	1.0033E-86
CASP5	ENSG00000137757	protein_coding	24.4624706	2.9480568	0.17562884	16.7857218	3.1043E-63	1.1693E-61
SQOR	ENSG00000137767	protein_coding	3237.31327	1.2639576	0.06952893	18.1788733	7.5877E-74	4.2315E-72
NUSAP1	ENSG00000137804	protein_coding	1078.01719	2.12681865	0.09986309	21.2973449	1.201E-100	1.8297E-98
ITPKA	ENSG00000137825	protein_coding	140.105632	3.72221698	0.24039015	15.4840663	4.4447E-54	1.2245E-52
PLCB2	ENSG00000137841	protein_coding	1360.02689	2.11780065	0.12125629	17.4654914	2.6243E-68	1.1852E-66
SPTBN5	ENSG00000137877	protein_coding	295.233563	1.24849648	0.17278058	7.22590763	4.9777E-13	1.8676E-12
BRDT	ENSG00000137948	protein_coding	2.31366808	2.29591001	0.31939115	7.18839585	6.5557E-13	2.4423E-12
IFI44L	ENSG00000137959	protein_coding	2973.64488	1.41514	0.13506611	10.4773875	1.0973E-25	8.7422E-25
IFI44	ENSG00000137965	protein_coding	1715.0024	1.55328719	0.10391168	14.9481478	1.6014E-50	3.8821E-49

DNASE2B	ENSG00000137976	protein_coding	22.050949	2.93803527	0.22607489	12.9958493	1.2917E-38	1.9008E-37
CGREF1	ENSG00000138028	protein_coding	1630.94789	3.02821183	0.16411473	18.4517974	5.0439E-76	3.0666E-74
LHCGR	ENSG00000138039	protein_coding	22.0438659	1.47164535	0.44304531	3.32165877	0.00089484	0.00151832
TRIM54	ENSG00000138100	protein_coding	318.76427	1.96282352	0.29253003	6.70981901	1.9487E-11	6.5389E-11
CYP2C9	ENSG00000138109	protein_coding	93.6158741	1.20934356	0.27181249	4.44918317	8.6197E-06	1.8045E-05
CYP2C8	ENSG00000138115	protein_coding	212.435165	1.84463043	0.21479927	8.58769406	8.8732E-18	4.5417E-17
STAMBPL1	ENSG00000138134	protein_coding	832.935627	2.46057771	0.09875182	24.9167834	4.895E-137	2.337E-134
LBX1	ENSG00000138136	protein_coding	0.62773299	1.05146197	0.48141673	2.18409935	0.02895495	0.04054754
BTBD16	ENSG00000138152	protein_coding	193.528243	3.56192248	0.17854412	19.9498165	1.5044E-88	1.4249E-86
CUZD1	ENSG00000138161	protein_coding	78.6870282	1.32892594	0.12825089	10.361924	3.6945E-25	2.8507E-24
CEP55	ENSG00000138180	protein_coding	298.825324	2.60235202	0.14639432	17.776318	1.0783E-70	5.3382E-69
ZNF365	ENSG00000138311	protein_coding	166.094901	1.38527864	0.22891823	6.0514125	1.4358E-09	4.1856E-09
ADAMTS14	ENSG00000138316	protein_coding	188.035124	3.38957001	0.20592392	16.4603028	7.0746E-61	2.4587E-59
DNA2	ENSG00000138346	protein_coding	212.860872	1.46239588	0.12307108	11.8825303	1.4588E-32	1.6319E-31
MYPN	ENSG00000138347	protein_coding	7.82774028	1.83904904	0.24778766	7.42187499	1.1547E-13	4.5371E-13
STAT4	ENSG00000138378	protein_coding	322.629002	1.87250095	0.10336314	18.1157509	2.3938E-73	1.3033E-71
MSTN	ENSG00000138379	protein_coding	45.4239682	1.37518542	0.1760237	7.81250145	5.6064E-15	2.4048E-14
HECW2	ENSG00000138411	protein_coding	2611.10851	1.41653073	0.13325318	10.6303709	2.1525E-26	1.7792E-25
CHRNA1	ENSG00000138435	protein_coding	57.2323204	5.32839047	0.29086518	18.3191075	5.826E-75	3.3764E-73
PCDH10	ENSG00000138650	protein_coding	1444.50422	1.63372988	0.23719441	6.88772504	5.6692E-12	1.9766E-11
ZGRF1	ENSG00000138658	protein_coding	189.528716	1.26802092	0.09748779	13.0069711	1.1168E-38	1.6472E-37
IL21	ENSG00000138684	protein_coding	0.72545789	1.83777897	0.43093069	4.26467417	2.0019E-05	4.0476E-05
CXCL9	ENSG00000138755	protein_coding	4529.71124	3.57911147	0.19388919	18.4595718	4.3679E-76	2.6662E-74
CENPE	ENSG00000138778	protein_coding	215.78596	1.61427471	0.13736675	11.7515684	6.932E-32	7.5159E-31
LEF1	ENSG00000138795	protein_coding	890.427628	1.15790336	0.18105926	6.39516238	1.6038E-10	5.0296E-10

C4orf17	ENSG00000138813	protein_coding	0.51434057	1.08148362	0.44709812	2.41889547	0.01556771	0.02267034
MAPK8IP3	ENSG00000138834	protein_coding	3836.82871	2.08497001	0.1322019	15.7711044	4.9182E-56	1.4462E-54
TTLL8	ENSG00000138892	protein_coding	0.89951669	1.45339456	0.44506171	3.2656023	0.00109232	0.00183652
SHISAL1	ENSG00000138944	protein_coding	184.574634	1.42985676	0.24308181	5.88220378	4.0484E-09	1.1362E-08
PARVG	ENSG00000138964	protein_coding	1124.26845	2.86158303	0.11923811	23.9988969	2.856E-127	9.696E-125
SLCO1C1	ENSG00000139155	protein_coding	101.266953	2.49724458	0.13744965	18.1684323	9.1783E-74	5.0999E-72
CD27	ENSG00000139193	protein_coding	532.838879	3.29343851	0.19372696	17.0004142	8.1542E-65	3.2532E-63
INHBE	ENSG00000139269	protein_coding	182.696089	3.33641699	0.24810063	13.4478379	3.1701E-41	5.1992E-40
GLIPR1	ENSG00000139278	protein_coding	1803.43047	1.50465152	0.12023838	12.5139039	6.2667E-36	8.2407E-35
KERA	ENSG00000139330	protein_coding	3.45717478	3.42694007	0.42878846	7.9921463	1.3261E-15	5.9336E-15
ASCL1	ENSG00000139352	protein_coding	4.46438036	2.68699905	0.40547269	6.6268312	3.4297E-11	1.1319E-10
GAS2L3	ENSG00000139354	protein_coding	2287.10768	3.09084868	0.13324341	23.1970096	4.881E-119	1.293E-116
SLC15A4	ENSG00000139370	protein_coding	5116.77848	1.8403783	0.07495462	24.5532347	3.994E-133	1.68E-130
GIT2	ENSG00000139436	protein_coding	3626.52033	1.28962656	0.078699	16.3868229	2.3754E-60	8.1566E-59
RDH16	ENSG00000139547	protein_coding	23.6897904	1.00930585	0.13914807	7.2534664	4.0624E-13	1.5325E-12
GPR84	ENSG00000139572	protein_coding	68.2100293	2.10775379	0.17054843	12.3586822	4.3727E-35	5.5128E-34
N4BP2L1	ENSG00000139597	protein_coding	1318.43096	1.33558178	0.06682081	19.9875133	7.0734E-89	6.7977E-87
CELA1	ENSG00000139610	protein_coding	2.66389927	2.23833119	0.28419894	7.87593089	3.3822E-15	1.4707E-14
BRCA2	ENSG00000139618	protein_coding	275.350681	1.70890695	0.10286144	16.6136791	5.5483E-62	1.9917E-60
MAP3K12	ENSG00000139625	protein_coding	953.15544	1.33136715	0.09241236	14.4068087	4.6888E-47	9.8146E-46
CSAD	ENSG00000139631	protein_coding	1097.29005	1.53490456	0.13576025	11.3059937	1.2256E-29	1.1959E-28
LMBR1L	ENSG00000139636	protein_coding	1772.73598	1.38474847	0.07915838	17.4933904	1.6089E-68	7.3243E-67
LPAR6	ENSG00000139679	protein_coding	2889.39607	1.19736807	0.09751385	12.2789537	1.1751E-34	1.4559E-33
RNF113B	ENSG00000139797	protein_coding	6.10183694	2.3447123	0.31903407	7.3494104	1.9908E-13	7.6865E-13
ZIC5	ENSG00000139800	protein_coding	8.45994634	3.66207302	0.46039459	7.95420523	1.8029E-15	7.995E-15

CDH24	ENSG00000139880	protein_coding	440.975463	1.09117147	0.08996052	12.1294487	7.3744E-34	8.8034E-33
CBLN3	ENSG00000139899	protein_coding	486.169873	1.63143909	0.10914681	14.9471995	1.6244E-50	3.9336E-49
GPR65	ENSG00000140030	protein_coding	556.92923	1.89607666	0.12150595	15.6048056	6.7515E-55	1.914E-53
SLC24A4	ENSG00000140090	protein_coding	87.1087444	1.72545505	0.10171525	16.9635827	1.5274E-64	6.0256E-63
HDC	ENSG00000140287	protein_coding	69.8545191	1.80528256	0.17477043	10.3294506	5.1857E-25	3.9652E-24
DISP2	ENSG00000140323	protein_coding	94.2733279	1.28024508	0.16315343	7.84687819	4.2652E-15	1.8425E-14
PSTPIP1	ENSG00000140368	protein_coding	430.863929	2.77526778	0.14768702	18.7915486	8.8557E-79	5.935E-77
BCL2A1	ENSG00000140379	protein_coding	248.042817	2.56240574	0.16494632	15.5347858	2.0175E-54	5.6152E-53
PIF1	ENSG00000140451	protein_coding	78.1067959	2.25423914	0.15641955	14.4114927	4.3814E-47	9.1879E-46
PML	ENSG00000140464	protein_coding	6508.22058	1.20792182	0.05663336	21.3288055	6.135E-101	9.534E-99
PCSK6	ENSG00000140479	protein_coding	5272.15305	2.96411077	0.1743214	17.0037118	7.7081E-65	3.0779E-63
CELF6	ENSG00000140488	protein_coding	3.73595065	1.57318034	0.23989147	6.5578836	5.4577E-11	1.7726E-10
LMAN1L	ENSG00000140506	protein_coding	0.96594369	2.39118529	0.47750019	5.00771592	5.508E-07	1.2914E-06
HAPLN3	ENSG00000140511	protein_coding	578.784323	2.38422086	0.13395376	17.7988348	7.2155E-71	3.5953E-69
RLBP1	ENSG00000140522	protein_coding	1.79996161	2.68173117	0.38861249	6.90078478	5.1716E-12	1.8069E-11
FANCI	ENSG00000140525	protein_coding	744.95721	1.2791867	0.0820495	15.5904272	8.4566E-55	2.3915E-53
TICRR	ENSG00000140534	protein_coding	108.227728	2.2869168	0.14022093	16.309383	8.4643E-60	2.8551E-58
MCTP2	ENSG00000140563	protein_coding	1460.34311	1.29634402	0.10352572	12.5219509	5.6626E-36	7.457E-35
SEPTIN12	ENSG00000140623	protein_coding	2.90199665	2.11089983	0.36926131	5.71654758	1.0871E-08	2.9421E-08
ITGAX	ENSG00000140678	protein_coding	1901.16928	3.27288584	0.13591255	24.0808211	3.971E-128	1.4E-125
IGSF6	ENSG00000140749	protein_coding	1153.49805	2.67652123	0.12149014	22.03077	1.461E-107	2.862E-105
ABCC12	ENSG00000140798	protein_coding	1.00778697	1.74826738	0.42772565	4.08735685	4.3632E-05	8.5362E-05
CLEC18B	ENSG00000140839	protein_coding	1366.49225	1.65710973	0.19537698	8.48160173	2.2211E-17	1.1116E-16
NLRC5	ENSG00000140853	protein_coding	3114.04847	2.23969835	0.10256424	21.8370299	1.033E-105	1.916E-103
ADAMTS18	ENSG00000140873	protein_coding	135.571497	1.5010138	0.16581126	9.05254435	1.3968E-19	7.9667E-19

CMTM3	ENSG00000140931	protein_coding	2573.59054	1.50833014	0.10276784	14.6770631	9.0423E-49	2.0389E-47
CMTM2	ENSG00000140932	protein_coding	21.1233879	1.02034676	0.13843885	7.37037851	1.7014E-13	6.607E-13
NOL3	ENSG00000140939	protein_coding	6002.5955	3.40890342	0.10825864	31.488512	1.248E-217	4.4E-214
CDH13	ENSG00000140945	protein_coding	5449.83908	1.57983193	0.12284331	12.8605451	7.5046E-38	1.0734E-36
IRF8	ENSG00000140968	protein_coding	1557.99262	1.04636275	0.12031452	8.69689508	3.4109E-18	1.7929E-17
RPS2	ENSG00000140988	protein_coding	41872.418	1.46643248	0.09165619	15.9992743	1.2927E-57	4.0395E-56
KSR1	ENSG00000141068	protein_coding	6270.51642	1.89848634	0.11200032	16.9507225	1.901E-64	7.4867E-63
CTRL	ENSG00000141086	protein_coding	4.21424635	1.4893403	0.20917564	7.12004647	1.0789E-12	3.9611E-12
DPEP3	ENSG00000141096	protein_coding	11.1222651	2.27418381	0.20017701	11.3608639	6.5494E-30	6.4635E-29
SPACA3	ENSG00000141316	protein_coding	2.4846898	1.71988233	0.37113528	4.63411168	3.5847E-06	7.7862E-06
C17orf64	ENSG00000141371	protein_coding	8.95170746	1.32571202	0.19456577	6.81369596	9.5123E-12	3.2617E-11
PRELID3A	ENSG00000141391	protein_coding	103.57107	1.60742193	0.11163518	14.398883	5.2587E-47	1.0992E-45
MEP1B	ENSG00000141434	protein_coding	5.36364745	1.42037848	0.20837691	6.8163908	9.3356E-12	3.2042E-11
SLC25A52	ENSG00000141437	protein_coding	4.73196031	1.17086236	0.22681139	5.1622733	2.4397E-07	5.9002E-07
ARRB2	ENSG00000141480	protein_coding	3361.50496	1.49491576	0.06893993	21.6843243	2.885E-104	5.145E-102
ZMYND15	ENSG00000141497	protein_coding	324.876138	1.71882617	0.0973834	17.6500949	1.0159E-69	4.8458E-68
ASGR1	ENSG00000141505	protein_coding	91.3187949	1.60688933	0.13879964	11.5770425	5.3873E-31	5.5923E-30
PIK3R5	ENSG00000141506	protein_coding	988.428966	2.78873938	0.11619496	24.0005198	2.746E-127	9.395E-125
SLC16A3	ENSG00000141526	protein_coding	14898.7489	3.03352625	0.11094594	27.3423812	1.33E-164	1.418E-161
CARD14	ENSG00000141527	protein_coding	185.272373	2.63455488	0.20627386	12.7721219	2.3467E-37	3.2928E-36
TTYH2	ENSG00000141540	protein_coding	587.501303	1.09333384	0.10106892	10.8177058	2.8379E-27	2.4588E-26
RNF165	ENSG00000141622	protein_coding	402.133152	1.38631189	0.15028361	9.22463787	2.8452E-20	1.6818E-19
ARL5C	ENSG00000141748	protein_coding	2.45006874	1.14129522	0.27266356	4.18572692	2.8425E-05	5.665E-05
FKBP10	ENSG00000141756	protein_coding	11690.9102	2.7863393	0.14636849	19.0364694	8.5073E-81	6.103E-79
CACNA1A	ENSG00000141837	protein_coding	105.207599	1.17650729	0.14505882	8.11055332	5.039E-16	2.3106E-15

VAV1	ENSG00000141968	protein_coding	794.978452	2.29970016	0.11870124	19.3738519	1.2829E-83	1.0105E-81
CIB3	ENSG00000141977	protein_coding	1.73615174	2.11100624	0.38394546	5.49819301	3.837E-08	9.9207E-08
SH3GL1	ENSG00000141985	protein_coding	5189.50278	1.03526696	0.07097653	14.5860462	3.446E-48	7.58E-47
DPP9	ENSG00000142002	protein_coding	4865.76837	1.4163819	0.07716866	18.354366	3.0462E-75	1.788E-73
DMRTC2	ENSG00000142025	protein_coding	2.48457506	3.16745125	0.53446095	5.92644088	3.0957E-09	8.7761E-09
TMEM91	ENSG00000142046	protein_coding	2536.61492	3.7344458	0.15252729	24.4837874	2.199E-132	9.08E-130
PGGHG	ENSG00000142102	protein_coding	6466.47233	3.56438884	0.19964723	17.8534351	2.7179E-71	1.3737E-69
COL6A1	ENSG00000142156	protein_coding	17272.0486	1.09521849	0.11746303	9.32394176	1.121E-20	6.7908E-20
COL6A2	ENSG00000142173	protein_coding	26003.2785	1.9617351	0.13373723	14.6685793	1.0247E-48	2.3048E-47
TRPM2	ENSG00000142185	protein_coding	833.758023	2.7523539	0.12305466	22.3669217	8.265E-111	1.813E-108
EMP3	ENSG00000142227	protein_coding	3542.90515	1.69749027	0.10271893	16.5255838	2.401E-61	8.4666E-60
NTN5	ENSG00000142233	protein_coding	26.6115561	1.88407575	0.16364032	11.5135176	1.1278E-30	1.1509E-29
ADAMTS10	ENSG00000142303	protein_coding	972.129525	2.15799051	0.13991969	15.4230648	1.1454E-53	3.116E-52
SLC6A3	ENSG00000142319	protein_coding	9391.54633	6.42371158	0.25892762	24.8089086	7.185E-136	3.229E-133
MYO1F	ENSG00000142347	protein_coding	2066.26645	2.49311643	0.11139468	22.3809299	6.037E-111	1.331E-108
NLRP12	ENSG00000142405	protein_coding	51.6676749	1.68323511	0.13243668	12.7097348	5.2211E-37	7.2134E-36
CACNG8	ENSG00000142408	protein_coding	52.3007754	2.19595519	0.16511903	13.2992251	2.3388E-40	3.7047E-39
GPR32	ENSG00000142511	protein_coding	0.76034106	2.12089752	0.530012	4.00160279	6.2915E-05	0.00012088
SIGLEC10	ENSG00000142512	protein_coding	888.203177	2.96229162	0.13803493	21.4604495	3.648E-102	5.951E-100
ACP4	ENSG00000142513	protein_coding	4.87484641	1.51379652	0.19310011	7.83943876	4.5256E-15	1.9529E-14
IGLON5	ENSG00000142549	protein_coding	356.651347	6.17649345	0.34064174	18.1319338	1.7837E-73	9.7809E-72
RCN3	ENSG00000142552	protein_coding	1409.62019	1.49649699	0.1418237	10.5518115	4.9827E-26	4.0413E-25
SLC2A5	ENSG00000142583	protein_coding	6563.3602	1.71198863	0.18820582	9.09636409	9.3406E-20	5.3738E-19
CFAP74	ENSG00000142609	protein_coding	61.7648276	4.00964758	0.15842111	25.3100593	2.475E-141	1.247E-138
CELA2A	ENSG00000142615	protein_coding	2.46355746	1.98451722	0.29429536	6.74328407	1.5485E-11	5.2343E-11

PADI3	ENSG00000142619	protein_coding	59.6429362	3.64286878	0.38450999	9.47405489	2.6919E-21	1.6878E-20
PADI1	ENSG00000142623	protein_coding	507.978294	5.72807334	0.28514181	20.0885073	9.3017E-90	9.2695E-88
EFHD2	ENSG00000142634	protein_coding	3999.26809	1.43231089	0.08487472	16.8755896	6.8042E-64	2.6145E-62
SH3BGRL3	ENSG00000142669	protein_coding	9120.68335	1.11966823	0.10830058	10.3385249	4.7174E-25	3.615E-24
IL22RA1	ENSG00000142677	protein_coding	393.742046	1.55453836	0.1748576	8.89031054	6.0938E-19	3.3519E-18
SYTL1	ENSG00000142765	protein_coding	324.666134	1.53215442	0.16458748	9.30905809	1.2897E-20	7.7904E-20
RPS8	ENSG00000142937	protein_coding	55477.7079	1.02515547	0.08276534	12.3862893	3.1006E-35	3.9416E-34
KIF2C	ENSG00000142945	protein_coding	284.276345	1.47220464	0.1306261	11.2703709	1.8379E-29	1.7801E-28
BEST4	ENSG00000142959	protein_coding	145.814122	2.67842812	0.21511551	12.4511157	1.379E-35	1.7873E-34
BARHL2	ENSG00000143032	protein_coding	0.95362342	2.05130311	0.46754577	4.38738462	1.1472E-05	2.3741E-05
FNDC7	ENSG00000143107	protein_coding	1.85057184	1.68769022	0.29870914	5.64994497	1.605E-08	4.2883E-08
C1orf162	ENSG00000143110	protein_coding	1722.33736	2.33528447	0.11548701	20.2211879	6.373E-91	6.6096E-89
CD53	ENSG00000143119	protein_coding	3588.76136	1.79964693	0.10965233	16.4123003	1.5617E-60	5.391E-59
ITGA10	ENSG00000143127	protein_coding	379.803564	1.15054322	0.15370316	7.48548844	7.1282E-14	2.8421E-13
GPA33	ENSG00000143167	protein_coding	18.4401798	1.69402582	0.16631491	10.1856522	2.2981E-24	1.6997E-23
TBX19	ENSG00000143178	protein_coding	284.416988	1.5680599	0.09855748	15.9101056	5.3922E-57	1.637E-55
XCL1	ENSG00000143184	protein_coding	72.5634799	2.37001871	0.1734736	13.6621291	1.7091E-42	2.9655E-41
XCL2	ENSG00000143185	protein_coding	58.5634505	2.56433618	0.17018017	15.0683609	2.6152E-51	6.5119E-50
ADCY10	ENSG00000143199	protein_coding	45.4507566	1.87508558	0.16213117	11.5652382	6.1819E-31	6.3912E-30
FCGR2A	ENSG00000143226	protein_coding	3048.97413	1.86239122	0.10988072	16.9492085	1.9506E-64	7.6688E-63
NUF2	ENSG00000143228	protein_coding	131.508945	1.96827818	0.14449011	13.6222346	2.9539E-42	5.0639E-41
RGS5	ENSG00000143248	protein_coding	124571.475	2.58501293	0.14797013	17.4698297	2.4322E-68	1.1006E-66
F13B	ENSG00000143278	protein_coding	1.51778067	2.62305423	0.39719236	6.60398964	4.0024E-11	1.3127E-10
FCRL5	ENSG00000143297	protein_coding	152.089878	2.02339696	0.27055155	7.47878546	7.5013E-14	2.9875E-13
FAM163A	ENSG00000143340	protein_coding	83.8512982	3.21618024	0.15937968	20.1793616	1.4865E-90	1.5177E-88

HMCN1	ENSG00000143341	protein_coding	2000.87558	1.22327491	0.12913438	9.47288337	2.7222E-21	1.7064E-20
LHX9	ENSG00000143355	protein_coding	22.3521548	2.71037875	0.43065397	6.29363463	3.1012E-10	9.5225E-10
ADAMTSL4	ENSG00000143382	protein_coding	1097.766	2.10052448	0.13691429	15.3418935	4.0132E-53	1.0708E-51
HORMAD1	ENSG00000143452	protein_coding	8.9574734	3.24067232	0.25943293	12.4913685	8.321E-36	1.0867E-34
DTL	ENSG00000143476	protein_coding	348.552288	2.12820046	0.12061385	17.6447441	1.1168E-69	5.3217E-68
DUSP10	ENSG00000143507	protein_coding	515.332892	1.38411634	0.10042697	13.7823174	3.2565E-43	5.8222E-42
HHIPL2	ENSG00000143512	protein_coding	8.83790701	2.46257304	0.27218419	9.04745086	1.4634E-19	8.3409E-19
FLG2	ENSG00000143520	protein_coding	7.2603154	1.386496	0.23300253	5.95056208	2.6722E-09	7.6171E-09
CRNN	ENSG00000143536	protein_coding	2.7672244	2.16597399	0.37980804	5.70281238	1.1785E-08	3.1821E-08
S100A8	ENSG00000143546	protein_coding	581.558518	1.51800712	0.16550148	9.17216663	4.6361E-20	2.7142E-19
SLC27A3	ENSG00000143554	protein_coding	2015.73079	1.48362956	0.09147919	16.2182196	3.7491E-59	1.2382E-57
S100A7	ENSG00000143556	protein_coding	3.06694805	1.48326676	0.42733738	3.47095017	0.00051862	0.00090319
EFNA3	ENSG00000143590	protein_coding	261.610339	2.39917197	0.12677258	18.9250072	7.098E-80	4.9375E-78
AQP10	ENSG00000143595	protein_coding	2.17256294	1.17889892	0.25930181	4.54643537	5.4562E-06	1.1649E-05
RIT1	ENSG00000143622	protein_coding	4454.35291	1.08704007	0.0755084	14.3962802	5.4604E-47	1.1404E-45
DEGS1	ENSG00000143753	protein_coding	8008.04745	1.26064683	0.0935423	13.4767568	2.1432E-41	3.5507E-40
PPFIA4	ENSG00000143847	protein_coding	1697.78196	3.14025759	0.17565028	17.8778966	1.7533E-71	8.9402E-70
PTPN7	ENSG00000143851	protein_coding	711.597052	1.73366198	0.14996752	11.5602498	6.5518E-31	6.7568E-30
DQX1	ENSG00000144045	protein_coding	9.10683276	2.93721383	0.2989972	9.8235497	8.9148E-23	6.0391E-22
MALL	ENSG00000144063	protein_coding	260.473687	2.05679478	0.14799371	13.8978522	6.5271E-44	1.2007E-42
NT5DC4	ENSG00000144130	protein_coding	10.101047	1.01837208	0.23776772	4.28305434	1.8435E-05	3.7402E-05
FBLN7	ENSG00000144152	protein_coding	519.438186	2.18967613	0.16460467	13.3026367	2.2345E-40	3.5431E-39
AFF3	ENSG00000144218	protein_coding	1173.22196	1.44622594	0.1362015	10.6182824	2.4503E-26	2.0184E-25
SCN1A	ENSG00000144285	protein_coding	37.7247134	2.09874243	0.23637157	8.87899691	6.7466E-19	3.7012E-18
CDCA7	ENSG00000144354	protein_coding	218.328028	2.00721627	0.15475682	12.9701311	1.8072E-38	2.6426E-37

DLX1	ENSG00000144355	protein_coding	49.9376284	3.18397046	0.17630517	18.0594272	6.6509E-73	3.5659E-71
CCDC150	ENSG00000144395	protein_coding	390.725666	1.10311895	0.16477969	6.69450787	2.164E-11	7.2419E-11
ABCA12	ENSG00000144452	protein_coding	478.788184	2.86292864	0.1834733	15.6040611	6.8307E-55	1.9353E-53
NYAP2	ENSG00000144460	protein_coding	2.72309471	1.06858765	0.28267236	3.78030462	0.00015664	0.00028879
ACKR3	ENSG00000144476	protein_coding	5015.95313	2.27634575	0.12563744	18.1183717	2.2825E-73	1.2441E-71
TRPM8	ENSG00000144481	protein_coding	39.30365	1.72785777	0.23384845	7.38879278	1.4817E-13	5.7786E-13
MARCHF4	ENSG00000144583	protein_coding	83.6447352	2.77070742	0.16778336	16.5136012	2.9287E-61	1.0312E-59
LRTM1	ENSG00000144771	protein_coding	1.46497676	1.64193433	0.42646668	3.85008817	0.00011808	0.00022063
COL8A1	ENSG00000144810	protein_coding	6169.50108	1.96879902	0.14362328	13.7080773	9.0833E-43	1.5984E-41
ADGRG7	ENSG00000144820	protein_coding	4.47625073	2.05123148	0.34283928	5.9830702	2.1897E-09	6.2898E-09
MYH15	ENSG00000144821	protein_coding	74.7478831	2.38140957	0.15976864	14.9053625	3.0416E-50	7.2809E-49
PLA1A	ENSG00000144837	protein_coding	2756.03695	1.63022764	0.1386595	11.7570569	6.4959E-32	7.0564E-31
NR1I2	ENSG00000144852	protein_coding	28.3957393	1.39502663	0.17157142	8.13087985	4.2619E-16	1.9633E-15
TMEM44	ENSG00000145014	protein_coding	1259.59947	1.79993035	0.11189975	16.0852045	3.2398E-58	1.0357E-56
STXBP5L	ENSG00000145087	protein_coding	15.3840029	1.40227327	0.38150466	3.67563864	0.00023726	0.00042883
TM4SF19	ENSG00000145107	protein_coding	20.9016065	2.87755217	0.2311888	12.4467631	1.4563E-35	1.8853E-34
SLC10A4	ENSG00000145248	protein_coding	7.11837805	1.04113396	0.18304005	5.68801187	1.2853E-08	3.4591E-08
SLC10A6	ENSG00000145283	protein_coding	68.1563486	2.59906026	0.15553951	16.7099687	1.1089E-62	4.0831E-61
PLAC8	ENSG00000145287	protein_coding	228.389433	1.8601948	0.15901854	11.6979747	1.3053E-31	1.399E-30
CCNA2	ENSG00000145386	protein_coding	349.858253	2.23656821	0.12130141	18.4381052	6.4979E-76	3.9245E-74
MARCHF1	ENSG00000145416	protein_coding	1202.75338	1.61320593	0.13376405	12.0600861	1.716E-33	2.0088E-32
SFRP2	ENSG00000145423	protein_coding	2777.41304	1.92627538	0.28827759	6.68201567	2.3568E-11	7.8681E-11
RNF175	ENSG00000145428	protein_coding	102.401772	2.41012102	0.14903081	16.171965	7.9521E-59	2.589E-57
ROPN1L	ENSG00000145491	protein_coding	64.4156074	1.24416424	0.12611346	9.86543584	5.8779E-23	4.0132E-22
NKD2	ENSG00000145506	protein_coding	516.696235	1.82174922	0.23649566	7.70309802	1.3281E-14	5.5461E-14

RPL37	ENSG00000145592	protein_coding	34577.6068	1.03317312	0.08396668	12.3045609	8.5601E-35	1.0663E-33
OSMR	ENSG00000145623	protein_coding	10184.4707	1.50822798	0.106636	14.1437038	2.0425E-45	3.9896E-44
PLK2	ENSG00000145632	protein_coding	6225.27973	2.21036121	0.09947916	22.2193401	2.233E-109	4.675E-107
GZMA	ENSG00000145649	protein_coding	1006.51915	3.12148022	0.16013785	19.4924575	1.2722E-84	1.0452E-82
HAPLN1	ENSG00000145681	protein_coding	1002.03643	3.53792692	0.20220814	17.4964614	1.5245E-68	6.9577E-67
LHFPL2	ENSG00000145685	protein_coding	3338.29858	2.00550903	0.11573584	17.3283322	2.8755E-67	1.2518E-65
ARHGAP26	ENSG00000145819	protein_coding	3250.88741	1.02774195	0.08286737	12.4022519	2.5408E-35	3.2534E-34
LECT2	ENSG00000145826	protein_coding	19.4004825	1.72563872	0.265614	6.49679128	8.2051E-11	2.631E-10
TIMD4	ENSG00000145850	protein_coding	420.940877	1.78568505	0.1711423	10.4339201	1.7358E-25	1.3672E-24
SPINK7	ENSG00000145879	protein_coding	13.6038056	1.20454425	0.29029692	4.14935254	3.3342E-05	6.6019E-05
GLRA1	ENSG00000145888	protein_coding	1.88377166	2.03567627	0.33610313	6.05670128	1.3894E-09	4.054E-09
TNIP1	ENSG00000145901	protein_coding	23722.2159	1.03576138	0.07049716	14.6922416	7.2282E-49	1.6355E-47
CPLX2	ENSG00000145920	protein_coding	4.84539276	3.59832494	0.43096474	8.34946484	6.8573E-17	3.3264E-16
KCNMB1	ENSG00000145936	protein_coding	532.427634	1.16930075	0.12947755	9.03091532	1.7024E-19	9.6585E-19
GFOD1	ENSG00000145990	protein_coding	1067.94623	1.0472323	0.11353885	9.22355882	2.874E-20	1.6986E-19
PSD2	ENSG00000146005	protein_coding	65.9629331	2.40476343	0.18290958	13.1472799	1.7641E-39	2.7001E-38
SLC17A4	ENSG00000146039	protein_coding	2850.41354	3.58433967	0.23718105	15.1122514	1.3447E-51	3.3887E-50
H2BC1	ENSG00000146047	protein_coding	1.2931215	1.15607523	0.35408312	3.26498259	0.00109471	0.00184028
TRIM7	ENSG00000146054	protein_coding	250.218987	1.44810046	0.1744103	8.3028379	1.0165E-16	4.8769E-16
FAM193B	ENSG00000146067	protein_coding	2581.0855	2.09199445	0.12978063	16.1194663	1.8621E-58	5.9944E-57
PLA2G7	ENSG00000146070	protein_coding	495.970557	3.65076507	0.17546026	20.8067916	3.7566E-96	4.9343E-94
RASGEF1C	ENSG00000146090	protein_coding	45.3131146	1.71139127	0.25564686	6.6943567	2.1662E-11	7.2488E-11
DOK3	ENSG00000146094	protein_coding	780.165793	2.62221051	0.10806228	24.2657329	4.511E-130	1.681E-127
PPP1R18	ENSG00000146112	protein_coding	4672.97523	1.64893823	0.0830477	19.8553146	9.914E-88	9.1442E-86
MLIP	ENSG00000146147	protein_coding	121.413153	2.14639266	0.16346356	13.1307108	2.1959E-39	3.3475E-38

FGD2	ENSG00000146192	protein_coding	816.219284	2.4939043	0.12528972	19.9050992	3.6756E-88	3.467E-86
ANO7	ENSG00000146205	protein_coding	206.645579	1.14150468	0.09019786	12.6555628	1.0423E-36	1.4232E-35
CRIP3	ENSG00000146215	protein_coding	100.424073	1.04319288	0.17610898	5.92356437	3.1504E-09	8.9278E-09
TCTE1	ENSG00000146221	protein_coding	10.7450818	1.48705633	0.17787721	8.36001625	6.271E-17	3.0484E-16
NFKBIE	ENSG00000146232	protein_coding	2140.41029	1.20909648	0.09124904	13.250512	4.4814E-40	6.9945E-39
GABRR1	ENSG00000146276	protein_coding	18.1493165	1.69830461	0.19027375	8.92558533	4.4335E-19	2.4557E-18
SCML4	ENSG00000146285	protein_coding	100.235845	1.32781989	0.13673584	9.7108404	2.7109E-22	1.7891E-21
CLVS2	ENSG00000146352	protein_coding	40.1150171	4.07922979	0.29592958	13.7844613	3.1612E-43	5.6562E-42
MTFR2	ENSG00000146410	protein_coding	64.9987497	1.48783444	0.11694108	12.7229407	4.4095E-37	6.1105E-36
VIP	ENSG00000146469	protein_coding	35.8972478	2.08792975	0.19103159	10.9297616	8.3066E-28	7.3881E-27
CREB5	ENSG00000146592	protein_coding	2391.10771	1.47803685	0.11098993	13.3168548	1.8473E-40	2.9393E-39
EGFR	ENSG00000146648	protein_coding	19274.2463	1.45312397	0.11101665	13.0892439	3.7937E-39	5.7319E-38
CDCA5	ENSG00000146670	protein_coding	293.053695	1.57967231	0.12379574	12.7603123	2.731E-37	3.8215E-36
IGFBP3	ENSG00000146674	protein_coding	134297.507	3.48589828	0.14426471	24.1632085	5.424E-129	1.973E-126
IGFBP1	ENSG00000146678	protein_coding	950.999823	3.62141538	0.34065723	10.6306723	2.1456E-26	1.7744E-25
ZAN	ENSG00000146839	protein_coding	14.5555576	4.8635686	0.40660719	11.9613445	5.6637E-33	6.4784E-32
AGBL3	ENSG00000146856	protein_coding	607.622218	1.52820181	0.11055343	13.8231962	1.8468E-43	3.3423E-42
STRA8	ENSG00000146857	protein_coding	146.982548	3.32195728	0.18154973	18.2977818	8.6187E-75	4.9697E-73
ZC3HAV1L	ENSG00000146858	protein_coding	434.478187	1.17594813	0.10147989	11.5879917	4.7412E-31	4.9369E-30
TMEM140	ENSG00000146859	protein_coding	6565.18436	1.41083997	0.07581894	18.6080156	2.7668E-77	1.7665E-75
CNPY1	ENSG00000146910	protein_coding	4.88966188	4.28193568	0.39314618	10.8914595	1.2659E-27	1.1158E-26
DIPK2B	ENSG00000147113	protein_coding	3785.44312	1.87743297	0.1151251	16.3077639	8.6917E-60	2.9275E-58
GPR174	ENSG00000147138	protein_coding	84.8564829	2.44760772	0.18985759	12.8918087	5.0056E-38	7.2112E-37
LPAR4	ENSG00000147145	protein_coding	22.0370786	1.19599111	0.16602273	7.20377918	5.8566E-13	2.1878E-12
IL2RG	ENSG00000147168	protein_coding	2029.67776	1.87422608	0.14439647	12.9797224	1.5946E-38	2.3391E-37

GCNA	ENSG00000147174	protein_coding	74.8670903	1.53928943	0.13487122	11.4130309	3.5996E-30	3.5888E-29
DIAPH2	ENSG00000147202	protein_coding	2629.17752	1.11833773	0.08056282	13.8815615	8.1939E-44	1.5031E-42
FRMPD3	ENSG00000147234	protein_coding	26.1606295	1.46959989	0.19064679	7.70849543	1.2731E-14	5.3238E-14
HTR2C	ENSG00000147246	protein_coding	1.00553891	1.55915745	0.47818693	3.26056056	0.00111192	0.00186771
ARHGAP36	ENSG00000147256	protein_coding	3.84738722	1.48392298	0.41186749	3.60291358	0.00031467	0.00056093
FATE1	ENSG00000147378	protein_coding	106.486669	3.52082763	0.1536705	22.9115386	3.565E-116	8.647E-114
RPL10	ENSG00000147403	protein_coding	75208.6562	1.03911955	0.0757386	13.7198148	7.7262E-43	1.3628E-41
CHRNA6	ENSG00000147434	protein_coding	32.9549193	2.98564022	0.21842685	13.6688336	1.5587E-42	2.7117E-41
GNRH1	ENSG00000147437	protein_coding	114.901987	2.80886527	0.15625554	17.9760999	2.9986E-72	1.5674E-70
DOK2	ENSG00000147443	protein_coding	569.142039	1.87164881	0.11488038	16.292154	1.1221E-59	3.7682E-58
SLC25A37	ENSG00000147454	protein_coding	3019.22153	1.00038986	0.09883505	10.1218122	4.4215E-24	3.2122E-23
STAR	ENSG00000147465	protein_coding	10.8381601	1.05416131	0.20966567	5.02782032	4.9609E-07	1.168E-06
RGS20	ENSG00000147509	protein_coding	43.8170603	2.85741593	0.22699986	12.5877434	2.4662E-36	3.3076E-35
DNAJC5B	ENSG00000147570	protein_coding	78.8981994	3.74622317	0.18902721	19.8184333	2.0644E-87	1.8851E-85
TRIM55	ENSG00000147573	protein_coding	1246.11903	1.02057741	0.15222121	6.70456762	2.02E-11	6.772E-11
GSDMC	ENSG00000147697	protein_coding	14.1839166	3.35034218	0.30994957	10.8093138	3.1099E-27	2.6883E-26
PLIN2	ENSG00000147872	protein_coding	54329.0724	2.89848083	0.14887736	19.4689159	2.0149E-84	1.6435E-82
IFNA5	ENSG00000147873	protein_coding	1.03044378	1.2630114	0.30578409	4.13040264	3.6213E-05	7.1448E-05
CDKN2B	ENSG00000147883	protein_coding	741.949177	1.68519891	0.10671902	15.7909899	3.589E-56	1.0587E-54
CDKN2A	ENSG00000147889	protein_coding	436.34325	4.9269717	0.16701592	29.5000119	2.878E-191	5.736E-188
IFNK	ENSG00000147896	protein_coding	2.79245521	2.83808971	0.38064292	7.4560423	8.916E-14	3.5277E-13
ACTL7B	ENSG00000148156	protein_coding	0.78129323	1.17460291	0.34431713	3.41139838	0.00064631	0.00111385
OR5C1	ENSG00000148215	protein_coding	1.06023819	2.41244742	0.45064566	5.35331333	8.6358E-08	2.1711E-07
IDI2	ENSG00000148377	protein_coding	0.82333299	1.24231839	0.33732545	3.68284805	0.00023064	0.00041746
CACNA1B	ENSG00000148408	protein_coding	9.84061429	1.11722685	0.27008415	4.13658796	3.5251E-05	6.9619E-05

SLC39A12	ENSG00000148482	protein_coding	2.64006877	1.83394687	0.29853277	6.14320118	8.0875E-10	2.4061E-09
CDHR1	ENSG00000148600	protein_coding	1558.09907	4.19468479	0.25993804	16.1372487	1.3963E-58	4.5044E-57
HTR7	ENSG00000148680	protein_coding	64.3682659	1.74547481	0.15160818	11.513065	1.1338E-30	1.1567E-29
HABP2	ENSG00000148702	protein_coding	4021.29559	1.71126168	0.31860927	5.37103535	7.8286E-08	1.9769E-07
VAX1	ENSG00000148704	protein_coding	1.45384257	1.01858443	0.4261172	2.3903856	0.01683069	0.02439716
NPFFR1	ENSG00000148734	protein_coding	41.8431877	2.50275068	0.22890101	10.9337685	7.9478E-28	7.0786E-27
MKI67	ENSG00000148773	protein_coding	1248.87435	2.20729522	0.14966362	14.7483752	3.1516E-49	7.2345E-48
ADAM12	ENSG00000148848	protein_coding	899.009674	1.8874407	0.2157391	8.74871855	2.1579E-18	1.1473E-17
RGS10	ENSG00000148908	protein_coding	1243.21727	1.60457023	0.10431447	15.3820485	2.16E-53	5.8212E-52
ADM	ENSG00000148926	protein_coding	11445.1335	2.69966711	0.11942553	22.6054431	3.831E-113	8.87E-111
SAA4	ENSG00000148965	protein_coding	15.3710037	3.74138945	0.43321652	8.63630382	5.8061E-18	3.01E-17
SCGB1A1	ENSG00000149021	protein_coding	1.29452759	1.62641528	0.34130496	4.7652846	1.8859E-06	4.2048E-06
SYT8	ENSG00000149043	protein_coding	42.9377839	2.60430029	0.3503782	7.43282629	1.063E-13	4.1864E-13
DGKZ	ENSG00000149091	protein_coding	4418.02865	1.27172463	0.06971232	18.2424661	2.375E-74	1.3458E-72
CCDC81	ENSG00000149201	protein_coding	54.3580365	1.34392026	0.13175493	10.2001515	1.9796E-24	1.4703E-23
SERPINH1	ENSG00000149257	protein_coding	13431.8539	1.54816332	0.09526696	16.2507894	2.2051E-59	7.3354E-58
NCAM1	ENSG00000149294	protein_coding	1277.81016	1.34158764	0.26143275	5.13167393	2.8718E-07	6.9043E-07
P4HA3	ENSG00000149380	protein_coding	447.380633	2.83785562	0.18655123	15.212205	2.9349E-52	7.6269E-51
GRIK4	ENSG00000149403	protein_coding	117.772079	1.69210033	0.22792521	7.42392789	1.137E-13	4.4688E-13
TMC2	ENSG00000149488	protein_coding	5.5815261	1.3336286	0.20366612	6.54811224	5.8269E-11	1.8873E-10
ZP1	ENSG00000149506	protein_coding	41.2041926	4.67678523	0.31341239	14.9221452	2.3654E-50	5.6921E-49
MS4A3	ENSG00000149516	protein_coding	2.08734308	1.36091438	0.37580182	3.62136187	0.00029306	0.00052423
MS4A2	ENSG00000149534	protein_coding	129.364421	1.2730065	0.17587954	7.23794528	4.5553E-13	1.714E-12
JPH2	ENSG00000149596	protein_coding	975.250828	1.65621585	0.15557666	10.6456576	1.8269E-26	1.5194E-25
SPINT4	ENSG00000149651	protein_coding	0.52450886	1.34022518	0.61527577	2.17825119	0.02938734	0.04112039

FERMT3	ENSG00000149781	protein_coding	1698.82479	2.09719125	0.10942569	19.1654373	7.195E-82	5.3197E-80
ALDOA	ENSG00000149925	protein_coding	113385.469	1.0987817	0.07425194	14.7980198	1.5086E-49	3.4998E-48
TLCD3B	ENSG00000149926	protein_coding	21.3094171	3.67589362	0.25685137	14.3113646	1.8583E-46	3.8064E-45
DOC2A	ENSG00000149927	protein_coding	1757.40751	6.11523753	0.21485465	28.4622065	3.442E-178	4.93E-175
HMGA2	ENSG00000149948	protein_coding	45.2062565	1.66562626	0.31287994	5.32353165	1.0177E-07	2.5421E-07
CLEC1A	ENSG00000150048	protein_coding	519.274906	1.24637186	0.10610617	11.7464596	7.364E-32	7.9729E-31
FCGR1A	ENSG00000150337	protein_coding	477.98914	3.08954932	0.14814236	20.8552724	1.3652E-96	1.8299E-94
CDH8	ENSG00000150394	protein_coding	122.10753	3.46557235	0.18510253	18.7224476	3.2487E-78	2.1306E-76
VEGFC	ENSG00000150630	protein_coding	912.294563	1.12036523	0.10876923	10.3003872	7.0178E-25	5.3262E-24
CCDC102B	ENSG00000150636	protein_coding	779.368819	1.43776773	0.10416748	13.8024622	2.4629E-43	4.4276E-42
RGS18	ENSG00000150681	protein_coding	318.503963	1.60184721	0.13130201	12.1997162	3.1191E-34	3.7836E-33
PRSS53	ENSG00000151006	protein_coding	180.31069	3.50846785	0.18260335	19.2136002	2.8481E-82	2.1403E-80
SLC7A11	ENSG00000151012	protein_coding	198.508595	1.77096794	0.15826351	11.1899954	4.5646E-29	4.334E-28
ENKUR	ENSG00000151023	protein_coding	120.973966	1.53292316	0.15222234	10.0702902	7.4757E-24	5.3613E-23
CACNA2D4	ENSG00000151062	protein_coding	403.008463	2.07533246	0.14414189	14.3978438	5.3383E-47	1.1154E-45
ADAMTS12	ENSG00000151388	protein_coding	462.734776	1.35306666	0.1651412	8.19339232	2.5396E-16	1.1864E-15
SLC25A31	ENSG00000151475	protein_coding	1.28324734	1.43013253	0.35475322	4.0313447	5.5459E-05	0.00010719
ANO4	ENSG00000151572	protein_coding	521.753157	3.72821888	0.21096402	17.6722969	6.8548E-70	3.2938E-68
DRD3	ENSG00000151577	protein_coding	0.69345663	1.58164144	0.34398783	4.59795756	4.2665E-06	9.2065E-06
EDNRA	ENSG00000151617	protein_coding	2013.44085	1.2306362	0.11817299	10.4138538	2.1436E-25	1.6792E-24
DPYSL4	ENSG00000151640	protein_coding	127.327502	2.29567898	0.26808201	8.56334579	1.0964E-17	5.5819E-17
VENTX	ENSG00000151650	protein_coding	100.935966	1.90987167	0.13800605	13.8390426	1.4817E-43	2.6964E-42
ADAM8	ENSG00000151651	protein_coding	647.136716	2.1267318	0.1508672	14.0967143	3.9787E-45	7.6794E-44
ANKAR	ENSG00000151687	protein_coding	217.348296	1.04077433	0.08887793	11.7101552	1.1307E-31	1.2168E-30
FLI1	ENSG00000151702	protein_coding	2604.20745	1.19818481	0.08283254	14.4651461	2.0118E-47	4.2774E-46

CENPU	ENSG00000151725	protein_coding	358.466813	1.82963414	0.10465703	17.482191	1.9583E-68	8.8881E-67
TDO2	ENSG00000151790	protein_coding	151.480112	1.85141841	0.21251547	8.71192296	2.9876E-18	1.5764E-17
SACS	ENSG00000151835	protein_coding	1623.52629	1.29258062	0.09889773	13.069872	4.8948E-39	7.3591E-38
CCL28	ENSG00000151882	protein_coding	1722.03672	1.52407703	0.15446906	9.86655234	5.8128E-23	3.9712E-22
TMEM132D	ENSG00000151952	protein_coding	60.6616168	1.17548744	0.25179133	4.66849847	3.0341E-06	6.637E-06
RBM46	ENSG00000151962	protein_coding	24.8509095	3.63869399	0.24128225	15.0806533	2.1711E-51	5.4267E-50
MCHR2	ENSG00000152034	protein_coding	0.7075113	1.30603092	0.4968786	2.62847088	0.00857697	0.01295604
KCNE4	ENSG00000152049	protein_coding	2034.65064	2.03870432	0.12173226	16.7474455	5.9111E-63	2.1923E-61
CCDC74B	ENSG00000152076	protein_coding	307.137493	1.25423487	0.15556229	8.06258948	7.4695E-16	3.3878E-15
MZT2B	ENSG00000152082	protein_coding	3337.01083	1.16170026	0.11771262	9.86895222	5.6754E-23	3.8796E-22
TUBA3E	ENSG00000152086	protein_coding	52.1578864	6.24992032	0.40243271	15.5303488	2.162E-54	6.0066E-53
HSPB8	ENSG00000152137	protein_coding	13153.2035	2.14910139	0.11799969	18.2127721	4.0872E-74	2.2933E-72
POU4F1	ENSG00000152192	protein_coding	17.9486605	3.59152503	0.37678929	9.53191914	1.544E-21	9.8046E-21
CYSLTR2	ENSG00000152207	protein_coding	1541.31495	1.5889379	0.2714124	5.85433057	4.7894E-09	1.3367E-08
ARL11	ENSG00000152213	protein_coding	173.293203	1.93110865	0.10213855	18.9067555	1.0034E-79	6.9379E-78
PSTPIP2	ENSG00000152229	protein_coding	530.564059	1.09820716	0.098777	11.1180448	1.0249E-28	9.5574E-28
SPC25	ENSG00000152253	protein_coding	117.571817	1.8378177	0.12767017	14.3950441	5.5589E-47	1.1604E-45
G6PC2	ENSG00000152254	protein_coding	10.3204372	1.27480848	0.24739417	5.15294461	2.5643E-07	6.1927E-07
PDK1	ENSG00000152256	protein_coding	3892.9941	1.90757375	0.0886	21.5301786	8.122E-103	1.369E-100
PTH	ENSG00000152266	protein_coding	1.09363341	2.46130661	0.55403877	4.44248083	8.8928E-06	1.8595E-05
TRIM36	ENSG00000152503	protein_coding	130.302749	1.26286072	0.13031541	9.69080135	3.2993E-22	2.169E-21
GRIA4	ENSG00000152578	protein_coding	904.628395	3.63701112	0.29922173	12.1549031	5.4026E-34	6.4849E-33
SPARCL1	ENSG00000152583	protein_coding	31117.7285	1.60501841	0.13825213	11.6093579	3.6938E-31	3.8641E-30
DSPP	ENSG00000152591	protein_coding	1.16752801	2.3571484	0.38378356	6.14186916	8.1556E-10	2.4259E-09
DMP1	ENSG00000152592	protein_coding	8.92216102	3.57264119	0.28251069	12.6460389	1.1766E-36	1.6029E-35

GJA1	ENSG00000152661	protein_coding	9579.95158	1.75590349	0.12015619	14.6135086	2.3034E-48	5.1085E-47
CLEC4F	ENSG00000152672	protein_coding	46.6341425	1.519192	0.18540377	8.19396487	2.5276E-16	1.1811E-15
RASGRP3	ENSG00000152689	protein_coding	2675.15277	1.03856229	0.07599134	13.6668511	1.6018E-42	2.7834E-41
CATSPER3	ENSG00000152705	protein_coding	66.8648108	1.03325285	0.1081069	9.55769582	1.2041E-21	7.6885E-21
RAB3C	ENSG00000152932	protein_coding	137.304962	1.4088279	0.15900556	8.86024325	7.984E-19	4.3607E-18
PLOD2	ENSG00000152952	protein_coding	18532.8022	1.77832522	0.13203376	13.4687155	2.3899E-41	3.9493E-40
JAKMIP1	ENSG00000152969	protein_coding	106.270635	3.56564059	0.18445346	19.3308418	2.9556E-83	2.2848E-81
ZIC1	ENSG00000152977	protein_coding	7.42929855	1.59624364	0.37639053	4.24092406	2.226E-05	4.4813E-05
CPB1	ENSG00000153002	protein_coding	36.3392168	3.15146969	0.27658426	11.3942479	4.4667E-30	4.4339E-29
CENPH	ENSG00000153044	protein_coding	252.793159	1.36957512	0.08715937	15.7134586	1.2232E-55	3.5579E-54
TEKT5	ENSG00000153060	protein_coding	4.03515909	1.49751918	0.22417186	6.68022834	2.3857E-11	7.9612E-11
CLGN	ENSG00000153132	protein_coding	171.1806	1.76788319	0.26235356	6.73855235	1.5997E-11	5.3997E-11
PTPRR	ENSG00000153233	protein_coding	106.959325	1.52728317	0.1579577	9.66893794	4.086E-22	2.675E-21
CD96	ENSG00000153283	protein_coding	663.240591	2.46110316	0.14526033	16.9427071	2.1786E-64	8.536E-63
SLC25A27	ENSG00000153291	protein_coding	405.043472	1.05206513	0.16244623	6.47638991	9.3943E-11	2.9985E-10
ADGRF4	ENSG00000153294	protein_coding	51.1976376	2.02517814	0.33753634	5.99988171	1.9746E-09	5.693E-09
ASAP1	ENSG00000153317	protein_coding	3835.17362	1.03219403	0.07277927	14.1825271	1.1754E-45	2.3284E-44
LPCAT1	ENSG00000153395	protein_coding	14378.2726	2.53754625	0.11388943	22.2807876	5.675E-110	1.199E-107
TEX29	ENSG00000153495	protein_coding	15.8037252	1.67206073	0.18039751	9.26875736	1.8833E-20	1.1262E-19
CD8A	ENSG00000153563	protein_coding	2073.72911	3.58105072	0.19036458	18.811539	6.0748E-79	4.0832E-77
GOLGA8F	ENSG00000153684	protein_coding	0.78237309	1.36285559	0.33678994	4.04660425	5.1966E-05	0.00010079
LURAP1L	ENSG00000153714	protein_coding	916.622261	1.0147432	0.10888865	9.31909027	1.1734E-20	7.1011E-20
CIBAR2	ENSG00000153789	protein_coding	8.31630758	1.78506745	0.29664563	6.01750796	1.7712E-09	5.1263E-09
TMPRSS11D	ENSG00000153802	protein_coding	5.97741939	1.87271384	0.25583032	7.3201402	2.4771E-13	9.4968E-13
LGI4	ENSG00000153902	protein_coding	5143.25489	4.57692725	0.23543628	19.4401953	3.5282E-84	2.8425E-82

GRAP	ENSG00000154016	protein_coding	174.882544	1.06242333	0.10304175	10.3106107	6.3099E-25	4.7929E-24
CABYR	ENSG00000154040	protein_coding	87.2429206	1.48445986	0.12284408	12.084098	1.2817E-33	1.5127E-32
ANKRD29	ENSG00000154065	protein_coding	883.794288	1.1028728	0.1215933	9.07017717	1.1882E-19	6.8011E-19
CHST9	ENSG00000154080	protein_coding	1205.74518	1.19013752	0.20621199	5.77142724	7.8603E-09	2.152E-08
C16orf74	ENSG00000154102	protein_coding	488.927654	3.06971536	0.20975321	14.6348907	1.6825E-48	3.7549E-47
PANX3	ENSG00000154143	protein_coding	1.31792052	2.42224885	0.41919827	5.77828927	7.5464E-09	2.068E-08
GPR15	ENSG00000154165	protein_coding	25.0122468	2.12146188	0.28297289	7.49704998	6.527E-14	2.6084E-13
LRRK1	ENSG00000154237	protein_coding	1969.12221	1.63172345	0.09940009	16.415714	1.4763E-60	5.1E-59
ENPP3	ENSG00000154269	protein_coding	15335.284	4.70596303	0.18162478	25.9103583	5.09E-148	3.432E-145
ASZ1	ENSG00000154438	protein_coding	1.06708828	1.06152271	0.44087188	2.40778048	0.01604983	0.02332203
GBP5	ENSG00000154451	protein_coding	1938.26631	3.50230669	0.18042414	19.4115196	6.1676E-84	4.883E-82
GPR26	ENSG00000154478	protein_coding	4.00879899	1.08629566	0.27055959	4.01499597	5.9447E-05	0.00011453
LY96	ENSG00000154589	protein_coding	437.066401	1.69725722	0.13307647	12.7539997	2.9615E-37	4.1377E-36
PSMA8	ENSG00000154611	protein_coding	2.13699141	1.37817747	0.24647613	5.59152508	2.2508E-08	5.9337E-08
SLFN13	ENSG00000154760	protein_coding	2425.28602	2.15992311	0.09673668	22.3278602	1.982E-110	4.267E-108
SKA1	ENSG00000154839	protein_coding	89.637352	2.12540173	0.14709624	14.4490552	2.5415E-47	5.3816E-46
PIEZO2	ENSG00000154864	protein_coding	1771.89417	2.13971731	0.1322842	16.1751534	7.5509E-59	2.4619E-57
EME1	ENSG00000154920	protein_coding	86.1793871	2.08480723	0.12989612	16.0498037	5.7345E-58	1.818E-56
SEPTIN14	ENSG00000154997	protein_coding	1.20866606	2.07630293	0.38670176	5.36926167	7.906E-08	1.9956E-07
CNTNAP5	ENSG00000155052	protein_coding	113.243762	1.37647977	0.31621812	4.35294395	1.3432E-05	2.7612E-05
MARVELD1	ENSG00000155254	protein_coding	2396.01435	1.09779102	0.09694892	11.3233964	1.005E-29	9.8337E-29
GOLGA7B	ENSG00000155265	protein_coding	276.5308	3.84814842	0.188627	20.4008352	1.6438E-92	1.8469E-90
SLC16A1	ENSG00000155380	protein_coding	5105.42833	2.02947523	0.10391964	19.529276	6.1911E-85	5.1695E-83
TRIM74	ENSG00000155428	protein_coding	7.49138393	2.18930995	0.22318468	9.80940983	1.0257E-22	6.9307E-22
GRIA1	ENSG00000155511	protein_coding	46.1225386	1.70314918	0.26789233	6.35758836	2.0495E-10	6.3703E-10

TTN	ENSG00000155657	protein_coding	768.056996	1.47407319	0.134901	10.9270735	8.5564E-28	7.6014E-27
VSIG4	ENSG00000155659	protein_coding	2106.54469	1.5901159	0.15365508	10.3486064	4.2462E-25	3.2632E-24
OTOA	ENSG00000155719	protein_coding	46.9345104	2.92261966	0.1510782	19.3450785	2.2426E-83	1.7543E-81
CYLC2	ENSG00000155833	protein_coding	0.77731768	2.22889437	0.47044608	4.73783177	2.1602E-06	4.7898E-06
ELMO1	ENSG00000155849	protein_coding	2558.86585	1.78671489	0.08777202	20.3563141	4.0816E-92	4.5085E-90
TRIM42	ENSG00000155890	protein_coding	1.8561535	1.08555233	0.3431888	3.16313451	0.0015608	0.00257787
ADCY8	ENSG00000155897	protein_coding	309.665542	3.2191266	0.33989594	9.47091808	2.7739E-21	1.7381E-20
SLA	ENSG00000155926	protein_coding	2151.8181	1.7692566	0.1195579	14.7983243	1.5018E-49	3.4875E-48
AFF2	ENSG00000155966	protein_coding	185.002681	1.18278691	0.1459121	8.10616071	5.2244E-16	2.393E-15
MAGEA8	ENSG00000156009	protein_coding	2.74189892	1.10210922	0.21018244	5.2435836	1.5749E-07	3.8706E-07
MMP16	ENSG00000156103	protein_coding	328.025605	2.71854031	0.17645551	15.4063781	1.4829E-53	4.0248E-52
KCNMA1	ENSG00000156113	protein_coding	3473.61718	2.27606865	0.16005017	14.2209702	6.7905E-46	1.354E-44
BATF	ENSG00000156127	protein_coding	197.508151	3.01622857	0.16281115	18.5259338	1.2757E-76	7.9456E-75
PPEF2	ENSG00000156194	protein_coding	5.80353868	2.04543693	0.24494577	8.3505704	6.7934E-17	3.2958E-16
SLC28A1	ENSG00000156222	protein_coding	7920.84192	1.87832287	0.1676482	11.2039547	3.8993E-29	3.7162E-28
CXCL13	ENSG00000156234	protein_coding	368.774028	4.18825991	0.28018939	14.9479604	1.6059E-50	3.891E-49
MAP3K7CL	ENSG00000156265	protein_coding	1793.10712	2.10952676	0.13704187	15.3933011	1.8153E-53	4.9094E-52
SORCS3	ENSG00000156395	protein_coding	985.990436	3.18060928	0.31427529	10.1204561	4.4832E-24	3.2554E-23
SH3RF2	ENSG00000156463	protein_coding	370.475223	1.29926703	0.2076719	6.2563448	3.941E-10	1.2017E-09
GDF6	ENSG00000156466	protein_coding	847.970957	3.49755597	0.19629075	17.8182411	5.1015E-71	2.5671E-69
FBXO43	ENSG00000156509	protein_coding	30.0477651	1.74448411	0.14928192	11.6858369	1.5059E-31	1.6099E-30
CD109	ENSG00000156535	protein_coding	5211.2372	1.0463364	0.1249177	8.37620613	5.4661E-17	2.6668E-16
NODAL	ENSG00000156574	protein_coding	11.4746848	1.58183266	0.14957477	10.5755311	3.8698E-26	3.1514E-25
PRG3	ENSG00000156575	protein_coding	0.84544268	2.27370878	0.52711659	4.31348359	1.607E-05	3.2772E-05
UBE2L6	ENSG00000156587	protein_coding	6395.76383	1.10482738	0.07395109	14.9399743	1.8105E-50	4.3727E-49

GLYATL2	ENSG00000156689	protein_coding	79.9511941	1.36377675	0.15629483	8.72566775	2.6461E-18	1.3996E-17
ITGAD	ENSG00000156886	protein_coding	132.971232	4.77729991	0.20549502	23.2477645	1.499E-119	4.089E-117
ADGRG4	ENSG00000156920	protein_coding	7.68251923	1.13025616	0.35587211	3.17601776	0.00149312	0.00247142
BUB1B	ENSG00000156970	protein_coding	225.382035	1.3019898	0.1290915	10.0857905	6.3849E-24	4.5977E-23
SST	ENSG00000157005	protein_coding	779.890349	3.52740042	0.36853066	9.57152492	1.0534E-21	6.7547E-21
SHCBP1L	ENSG00000157060	protein_coding	0.93846864	1.40770504	0.37837973	3.72035003	0.00019895	0.00036284
NMNAT2	ENSG00000157064	protein_coding	248.06393	1.35607415	0.20610802	6.57943434	4.7224E-11	1.5418E-10
ATP2B2	ENSG00000157087	protein_coding	891.342703	3.43425873	0.20170157	17.0264352	5.2294E-65	2.0991E-63
SLC6A1	ENSG00000157103	protein_coding	461.186108	2.28055006	0.1429338	15.9552883	2.6176E-57	8.1077E-56
C8A	ENSG00000157131	protein_coding	3.34545428	1.07013111	0.4880587	2.19262786	0.0283342	0.0397454
CDCP2	ENSG00000157211	protein_coding	4.57521675	1.04370152	0.19022831	5.4865731	4.0981E-08	1.0569E-07
MMP14	ENSG00000157227	protein_coding	14990.6041	1.44402508	0.10212936	14.1391763	2.1782E-45	4.2526E-44
FZD1	ENSG00000157240	protein_coding	8927.95866	1.25510923	0.09713149	12.9217543	3.3932E-38	4.9177E-37
CLEC18A	ENSG00000157322	protein_coding	741.874439	2.04632141	0.21705062	9.4278532	4.1857E-21	2.5957E-20
CLEC18C	ENSG00000157335	protein_coding	608.816105	1.69232189	0.24549268	6.89357375	5.4408E-12	1.8985E-11
ARMC12	ENSG00000157343	protein_coding	27.8202777	2.01902265	0.17361751	11.6291416	2.9303E-31	3.088E-30
IL34	ENSG00000157368	protein_coding	546.440857	1.27115273	0.1440626	8.82361349	1.1082E-18	5.9987E-18
CCNB2	ENSG00000157456	protein_coding	282.748163	1.73568394	0.12905213	13.4494794	3.1005E-41	5.0906E-40
AFAP1L1	ENSG00000157510	protein_coding	2449.54843	1.47897705	0.09676452	15.28429	9.7318E-53	2.5727E-51
TSC22D3	ENSG00000157514	protein_coding	19992.5305	1.10950016	0.12424846	8.92968953	4.2721E-19	2.3677E-18
TSPAN18	ENSG00000157570	protein_coding	6594.07353	1.3078107	0.11642278	11.2332889	2.7986E-29	2.6913E-28
SNX22	ENSG00000157734	protein_coding	228.406368	1.24162161	0.1195274	10.3877573	2.819E-25	2.1922E-24
ACAN	ENSG00000157766	protein_coding	2504.83277	2.59813052	0.18125055	14.3344696	1.3326E-46	2.7394E-45
GAREM2	ENSG00000157833	protein_coding	540.256984	1.23819153	0.10799341	11.4654362	1.9677E-30	1.989E-29
DPYSL5	ENSG00000157851	protein_coding	13.6557736	1.02998504	0.33563787	3.06873903	0.00214964	0.00349278

TNFRSF14	ENSG00000157873	protein_coding	7533.2132	2.12640277	0.09835883	21.6188303	1.195E-103	2.074E-101
CIB4	ENSG00000157884	protein_coding	62.6554463	2.52460813	0.24360062	10.363718	3.6259E-25	2.7992E-24
MEGF11	ENSG00000157890	protein_coding	464.00749	1.10267353	0.23153179	4.76251466	1.912E-06	4.2609E-06
ANKRD61	ENSG00000157999	protein_coding	24.5773895	1.21060473	0.13395146	9.03763771	1.6009E-19	9.1053E-19
UBXN11	ENSG00000158062	protein_coding	1815.76954	1.31544214	0.10844545	12.1299891	7.3259E-34	8.7478E-33
GALNT14	ENSG00000158089	protein_coding	12646.0708	1.10143599	0.11727545	9.39187159	5.8943E-21	3.621E-20
XDH	ENSG00000158125	protein_coding	52.6403458	1.21805557	0.30994482	3.9299111	8.4977E-05	0.00016119
RNF207	ENSG00000158286	protein_coding	666.620386	1.13711987	0.13749882	8.2700334	1.3392E-16	6.3756E-16
CDC25C	ENSG00000158402	protein_coding	73.0020383	2.71852674	0.16343092	16.6341032	3.9462E-62	1.4266E-60
H4C8	ENSG00000158406	protein_coding	153.496237	1.12224634	0.15811754	7.09754512	1.2699E-12	4.6408E-12
TMSB15B	ENSG00000158427	protein_coding	15.8577117	1.5379084	0.12207296	12.5982726	2.1582E-36	2.9004E-35
KCNB1	ENSG00000158445	protein_coding	357.903647	1.26142476	0.18216765	6.92452663	4.3744E-12	1.5372E-11
CD1D	ENSG00000158473	protein_coding	487.779358	2.26091985	0.09646432	23.4378877	1.757E-121	5.13E-119
CD1A	ENSG00000158477	protein_coding	24.0141785	2.44803437	0.26049026	9.39779624	5.5718E-21	3.4279E-20
DNAH3	ENSG00000158486	protein_coding	26.0243439	1.11905822	0.17692019	6.32521478	2.5288E-10	7.811E-10
HMHB1	ENSG00000158497	protein_coding	4.11596287	2.76920142	0.30973036	8.94068452	3.8677E-19	2.1472E-18
NCF1	ENSG00000158517	protein_coding	226.08405	2.28084385	0.13281446	17.1731587	4.2183E-66	1.7563E-64
GDPD5	ENSG00000158555	protein_coding	700.308113	1.38537333	0.10264998	13.4960902	1.649E-41	2.7428E-40
ALAS2	ENSG00000158578	protein_coding	47.6873364	1.07586639	0.22658651	4.7481485	2.0529E-06	4.5607E-06
PKD1L1	ENSG00000158683	protein_coding	85.9017407	1.83464269	0.13150615	13.9510026	3.1021E-44	5.7854E-43
SLAMF8	ENSG00000158714	protein_coding	1114.17431	2.69588259	0.15632143	17.2457651	1.2041E-66	5.1013E-65
DUSP23	ENSG00000158716	protein_coding	3258.67221	1.02751569	0.11946356	8.6010803	7.8969E-18	4.0561E-17
RNF166	ENSG00000158717	protein_coding	1179.95884	1.28210819	0.07241913	17.7039988	3.9056E-70	1.8906E-68
NBL1	ENSG00000158747	protein_coding	2352.66758	1.18178799	0.16853938	7.01193976	2.3504E-12	8.422E-12
HTR6	ENSG00000158748	protein_coding	106.278352	6.44573772	0.31733237	20.3122605	1.0019E-91	1.073E-89

ZNF276	ENSG00000158805	protein_coding	993.81955	1.28952297	0.09576694	13.46522	2.5057E-41	4.1333E-40
FGF17	ENSG00000158815	protein_coding	14.0391975	1.33861	0.21026475	6.36630727	1.9363E-10	6.0297E-10
ADAMTS4	ENSG00000158859	protein_coding	4850.36281	1.98256731	0.16375674	12.1067828	9.7234E-34	1.1523E-32
FCER1G	ENSG00000158869	protein_coding	2686.06965	2.52756426	0.12088941	20.9080709	4.5215E-97	6.1504E-95
IFNAR2	ENSG00000159110	protein_coding	1420.34772	1.09958955	0.06522679	16.8579438	9.1725E-64	3.5069E-62
IFNGR2	ENSG00000159128	protein_coding	6379.30632	1.10240513	0.06138278	17.9595175	4.0431E-72	2.1013E-70
HOXB13	ENSG00000159184	protein_coding	50.8391668	3.71535139	0.41150831	9.02861804	1.7385E-19	9.8536E-19
C1QC	ENSG00000159189	protein_coding	12029.172	2.74300255	0.13658819	20.0822818	1.0544E-89	1.0485E-87
CIART	ENSG00000159208	protein_coding	569.186616	1.33710007	0.11066068	12.0828834	1.3008E-33	1.5337E-32
RUNX1	ENSG00000159216	protein_coding	3698.26095	1.76667454	0.12820052	13.7805569	3.3369E-43	5.9636E-42
GIP	ENSG00000159224	protein_coding	1.67232361	2.86698206	0.51020826	5.61923887	1.918E-08	5.0879E-08
CBR3	ENSG00000159231	protein_coding	199.569243	1.24191298	0.12588648	9.86534063	5.8834E-23	4.0158E-22
GOLGA6A	ENSG00000159289	protein_coding	0.96796501	1.85676219	0.32429612	5.72551469	1.0312E-08	2.7968E-08
PLA2G4D	ENSG00000159337	protein_coding	31.479595	4.4776261	0.32628538	13.7230364	7.3904E-43	1.305E-41
PADI4	ENSG00000159339	protein_coding	21.7350367	1.10391789	0.19212301	5.74589109	9.1438E-09	2.4913E-08
HK2	ENSG00000159399	protein_coding	5338.1895	3.4234343	0.13364681	25.6155339	1.024E-144	5.726E-142
C1R	ENSG00000159403	protein_coding	18338.7426	1.10200114	0.17280123	6.37727591	1.8027E-10	5.6272E-10
STARD9	ENSG00000159433	protein_coding	1171.47795	1.50454855	0.10981481	13.7007794	1.0044E-42	1.7614E-41
RGL4	ENSG00000159496	protein_coding	92.4074968	1.91859081	0.12331242	15.5587797	1.3872E-54	3.8893E-53
ISL2	ENSG00000159556	protein_coding	7.16896782	2.54955138	0.3020528	8.44074738	3.1531E-17	1.562E-16
CCDC17	ENSG00000159588	protein_coding	68.1748649	1.20826904	0.151695	7.96512113	1.6506E-15	7.3391E-15
ACE	ENSG00000159640	protein_coding	2427.3248	1.07980004	0.13409737	8.05235805	8.1214E-16	3.6766E-15
UROC1	ENSG00000159650	protein_coding	8.1265021	2.03000794	0.23810054	8.52584338	1.517E-17	7.6621E-17
SPON2	ENSG00000159674	protein_coding	4871.43239	1.77081895	0.18378214	9.63542441	5.6657E-22	3.6845E-21
LRRC36	ENSG00000159708	protein_coding	131.558463	1.81500522	0.1321643	13.7329463	6.4458E-43	1.1409E-41

CARMIL2	ENSG00000159753	protein_coding	203.446548	2.49987709	0.1666719	14.9987912	7.4769E-51	1.8309E-49
PIP	ENSG00000159763	protein_coding	18.1613015	2.9133247	0.45677066	6.37809064	1.7931E-10	5.5989E-10
TNFRSF13C	ENSG00000159958	protein_coding	68.6768189	1.17686184	0.18093142	6.50446375	7.7971E-11	2.5047E-10
ATAD3B	ENSG00000160072	protein_coding	602.795698	1.41901384	0.12798917	11.086984	1.451E-28	1.3391E-27
ANKLE1	ENSG00000160117	protein_coding	32.3887542	2.04436956	0.15321726	13.3429459	1.3021E-40	2.0834E-39
CILP2	ENSG00000160161	protein_coding	44.8058252	1.9274612	0.24265509	7.94321349	1.9701E-15	8.7156E-15
ABCG1	ENSG00000160179	protein_coding	3993.7749	1.28561918	0.08333824	15.426522	1.0857E-53	2.9553E-52
TFF2	ENSG00000160181	protein_coding	16.9508277	1.5564241	0.36116074	4.30950521	1.6362E-05	3.3343E-05
UBASH3A	ENSG00000160185	protein_coding	173.856319	2.78296105	0.16129759	17.2535812	1.0517E-66	4.4849E-65
U2AF1	ENSG00000160201	protein_coding	61.6590676	1.59066635	0.17838324	8.91712884	4.7853E-19	2.6468E-18
GAB3	ENSG00000160219	protein_coding	486.633348	1.60775549	0.08504379	18.9050304	1.0368E-79	7.1578E-78
ITGB2	ENSG00000160255	protein_coding	8539.44885	2.37875111	0.11976913	19.8611371	8.8289E-88	8.1928E-86
RALGDS	ENSG00000160271	protein_coding	3442.6058	1.11438961	0.06921244	16.1010005	2.5101E-58	8.0466E-57
C21orf58	ENSG00000160298	protein_coding	147.91933	1.26424643	0.10909902	11.5880645	4.7372E-31	4.9342E-30
LCN1	ENSG00000160349	protein_coding	4.22919671	4.17270037	0.34843335	11.9756055	4.7694E-33	5.4755E-32
NLRP4	ENSG00000160505	protein_coding	3.58673588	1.65152796	0.26623806	6.20320005	5.5326E-10	1.6679E-09
JAML	ENSG00000160593	protein_coding	1029.8667	1.66237954	0.11788221	14.1020386	3.6896E-45	7.1364E-44
CD3G	ENSG00000160654	protein_coding	418.55306	2.53965329	0.16064577	15.8090267	2.696E-56	7.9888E-55
SHC1	ENSG00000160691	protein_coding	8723.79833	1.06687095	0.07036065	15.162893	6.2265E-52	1.6008E-50
PAQR6	ENSG00000160781	protein_coding	183.48298	2.59043843	0.17473285	14.8251371	1.0077E-49	2.3605E-48
CCR5	ENSG00000160791	protein_coding	1108.05769	2.88118068	0.15625993	18.4383848	6.4644E-76	3.9094E-74
PPP1R35	ENSG00000160813	protein_coding	668.448155	1.02884428	0.10058532	10.2285733	1.4768E-24	1.1031E-23
LRRC71	ENSG00000160838	protein_coding	24.8014111	2.99721574	0.20126931	14.8915689	3.7389E-50	8.9083E-49
FCRL3	ENSG00000160856	protein_coding	181.704295	2.80527262	0.19785784	14.1782233	1.2497E-45	2.4714E-44
CYP11B1	ENSG00000160882	protein_coding	0.49763879	1.56279718	0.46686236	3.34744734	0.00081559	0.00139019

HK3	ENSG00000160883	protein_coding	312.182794	2.58034957	0.13444872	19.1920719	4.311E-82	3.2134E-80
LY6E	ENSG00000160932	protein_coding	15584.2578	1.68039625	0.14497152	11.5912161	4.566E-31	4.7596E-30
FOXH1	ENSG00000160973	protein_coding	7.57705873	2.00134555	0.21360855	9.36922006	7.307E-21	4.4644E-20
CCDC105	ENSG00000160994	protein_coding	0.87220181	2.10287112	0.52579758	3.99939295	6.3505E-05	0.00012193
SH2B2	ENSG00000160999	protein_coding	362.026007	1.28885959	0.09658725	13.3439928	1.2839E-40	2.0551E-39
MRNIP	ENSG00000161010	protein_coding	1454.17299	1.1896572	0.10367529	11.4748383	1.7651E-30	1.7889E-29
PGLYRP2	ENSG00000161031	protein_coding	20.8857937	3.4166266	0.31081481	10.9924834	4.1534E-28	3.7509E-27
SCGB3A1	ENSG00000161055	protein_coding	41.3586827	1.69438545	0.18060235	9.38185702	6.4821E-21	3.9704E-20
CELF5	ENSG00000161082	protein_coding	80.1491316	1.88522388	0.27667186	6.81393419	9.4965E-12	3.2568E-11
CCDC116	ENSG00000161180	protein_coding	15.2529906	1.67823089	0.15441203	10.8685246	1.6281E-27	1.4246E-26
U2AF1L4	ENSG00000161265	protein_coding	450.702635	1.1326908	0.10906083	10.385863	2.8756E-25	2.235E-24
PLXDC1	ENSG00000161381	protein_coding	2784.53182	2.86418495	0.12401474	23.0955197	5.136E-118	1.33E-115
IKZF3	ENSG00000161405	protein_coding	1027.26086	2.55713067	0.16106635	15.8762563	9.2539E-57	2.7909E-55
FDXR	ENSG00000161513	protein_coding	1519.44995	1.39669846	0.09227757	15.1358399	9.3973E-52	2.384E-50
CYGB	ENSG00000161544	protein_coding	2505.91579	2.01142909	0.12078252	16.6533124	2.8631E-62	1.0416E-60
KASH5	ENSG00000161609	protein_coding	4.19220435	1.75012798	0.29458161	5.9410633	2.8318E-09	8.0549E-09
ITGA5	ENSG00000161638	protein_coding	11768.8377	1.95451302	0.11012353	17.748368	1.7744E-70	8.6807E-69
FMNL3	ENSG00000161791	protein_coding	5350.76383	1.26528382	0.07429879	17.02967	4.9482E-65	1.9898E-63
KRT84	ENSG00000161849	protein_coding	1.25921499	2.86254483	0.35847863	7.98525924	1.4023E-15	6.2665E-15
KRT82	ENSG00000161850	protein_coding	1.11866012	2.17329523	0.45079889	4.8209862	1.4285E-06	3.2212E-06
SYCE2	ENSG00000161860	protein_coding	37.1735821	1.01168437	0.12588631	8.03649241	9.2447E-16	4.1699E-15
SPC24	ENSG00000161888	protein_coding	216.422368	2.31309663	0.14288239	16.1888154	6.0482E-59	1.9818E-57
TREML1	ENSG00000161911	protein_coding	35.472368	2.94139297	0.15970729	18.4173998	9.5271E-76	5.6866E-74
SCIMP	ENSG00000161929	protein_coding	421.552282	1.94736296	0.12413683	15.6872299	1.8495E-55	5.3458E-54
BCL6B	ENSG00000161940	protein_coding	2786.16596	1.32180239	0.11823979	11.1789981	5.167E-29	4.8928E-28

FGF11	ENSG00000161958	protein_coding	144.382543	1.66585465	0.22089654	7.54133428	4.6519E-14	1.8762E-13
EIF4A1	ENSG00000161960	protein_coding	519.675702	2.1190538	0.13835298	15.3162856	5.9523E-53	1.5809E-51
WDR90	ENSG00000161996	protein_coding	1619.37114	1.32390782	0.09646532	13.7241849	7.2743E-43	1.285E-41
CCDC78	ENSG00000162004	protein_coding	121.237689	2.84325958	0.2266713	12.5435358	4.3131E-36	5.7094E-35
MSLNL	ENSG00000162006	protein_coding	5.77632992	2.81075618	0.44656462	6.29417569	3.0904E-10	9.4899E-10
MEIOB	ENSG00000162039	protein_coding	3.03079569	1.33091604	0.28442715	4.67928622	2.8788E-06	6.312E-06
TEDC2	ENSG00000162062	protein_coding	67.9038281	1.01275734	0.13164436	7.69313122	1.4358E-14	5.9823E-14
PAQR4	ENSG00000162073	protein_coding	385.406749	1.73022626	0.11281567	15.3367548	4.3437E-53	1.1577E-51
LBHD1	ENSG00000162194	protein_coding	180.111929	1.0304838	0.08907083	11.5692623	5.8988E-31	6.1081E-30
ITIH3	ENSG00000162267	protein_coding	264.465041	1.31734258	0.21007456	6.27083344	3.5912E-10	1.0983E-09
FGF19	ENSG00000162344	protein_coding	1.01585996	1.18107574	0.45817924	2.57775918	0.00994433	0.01488807
ELAVL4	ENSG00000162374	protein_coding	15.2864495	1.58881006	0.14389978	11.0410874	2.4209E-28	2.2102E-27
SLC1A7	ENSG00000162383	protein_coding	121.662985	2.14401909	0.24733573	8.66845679	4.3802E-18	2.2882E-17
CTRC	ENSG00000162438	protein_coding	6.02809063	2.92640729	0.27344341	10.7020581	9.9542E-27	8.3804E-26
MATN1	ENSG00000162510	protein_coding	19.1588696	1.65608694	0.13547228	12.2245443	2.2987E-34	2.8017E-33
LAPTM5	ENSG00000162511	protein_coding	17445.5479	2.52783432	0.11540944	21.9031855	2.422E-106	4.57E-104
SDC3	ENSG00000162512	protein_coding	6642.52648	1.06290116	0.08659632	12.2742076	1.2461E-34	1.5409E-33
SCNN1D	ENSG00000162572	protein_coding	230.89541	2.38044159	0.17071059	13.944311	3.4072E-44	6.3441E-43
MXRA8	ENSG00000162576	protein_coding	4236.45484	1.24720469	0.17312805	7.2039437	5.8496E-13	2.1854E-12
MEGF6	ENSG00000162591	protein_coding	1274.82717	1.30640731	0.1389605	9.40128537	5.3901E-21	3.3175E-20
ADGRL4	ENSG00000162618	protein_coding	5849.90991	1.00773282	0.11396908	8.84215982	9.3886E-19	5.1041E-18
LRRC53	ENSG00000162621	protein_coding	1.27955113	1.08125417	0.31082447	3.47866487	0.00050392	0.00087917
LHX8	ENSG00000162624	protein_coding	28.134376	5.50356416	0.43364216	12.6914877	6.5922E-37	9.0694E-36
GBP2	ENSG00000162645	protein_coding	8129.12384	1.98234436	0.10414567	19.0343429	8.8597E-81	6.336E-79
GBP4	ENSG00000162654	protein_coding	7857.80515	1.22531887	0.12158803	10.0776274	6.9381E-24	4.9828E-23

GFI1	ENSG00000162676	protein_coding	158.698218	2.46875632	0.1487142	16.6006761	6.891E-62	2.4641E-60
VCAM1	ENSG00000162692	protein_coding	32624.2059	1.79136445	0.14753056	12.1423279	6.3008E-34	7.5394E-33
CADM3	ENSG00000162706	protein_coding	956.524349	2.99975814	0.27426565	10.9374183	7.6343E-28	6.8033E-27
NLRP3	ENSG00000162711	protein_coding	370.765197	1.34159148	0.11675937	11.4902259	1.4773E-30	1.5042E-29
SLAMF9	ENSG00000162723	protein_coding	5.53570538	1.98832148	0.32326328	6.15078053	7.7103E-10	2.299E-09
SLAMF6	ENSG00000162739	protein_coding	521.213491	2.51246845	0.15617309	16.087717	3.111E-58	9.9519E-57
OLFML2B	ENSG00000162745	protein_coding	3652.26875	2.67436633	0.13609254	19.6510866	5.6588E-86	4.9223E-84
FCGR3B	ENSG00000162747	protein_coding	143.502466	1.27327967	0.19830752	6.42073305	1.3562E-10	4.274E-10
SLC9C2	ENSG00000162753	protein_coding	21.5657459	1.21422025	0.27073109	4.48496793	7.2925E-06	1.5381E-05
FAM71A	ENSG00000162771	protein_coding	0.73768553	1.64927885	0.43264266	3.81210411	0.00013779	0.00025558
KLHDC8A	ENSG00000162873	protein_coding	161.932067	1.70064782	0.17883512	9.50958505	1.9142E-21	1.2099E-20
IL24	ENSG00000162892	protein_coding	92.8765043	1.12549865	0.11452615	9.82743799	8.5773E-23	5.8173E-22
FCMR	ENSG00000162894	protein_coding	501.457217	1.46691201	0.11727944	12.5078356	6.7642E-36	8.8822E-35
DISC1	ENSG00000162946	protein_coding	700.496628	1.64279481	0.08136658	20.1900443	1.1976E-90	1.2281E-88
KCNF1	ENSG00000162975	protein_coding	133.813536	1.50931712	0.20137281	7.49513847	6.6228E-14	2.6455E-13
FRZB	ENSG00000162998	protein_coding	7568.18359	2.02271201	0.19498774	10.3735345	3.2719E-25	2.5349E-24
FBXO41	ENSG00000163013	protein_coding	498.565183	1.973552	0.16210363	12.1746314	4.2431E-34	5.1159E-33
ACTG2	ENSG00000163017	protein_coding	1698.28844	1.64401291	0.19985353	8.22608889	1.9343E-16	9.0998E-16
CCDC74A	ENSG00000163040	protein_coding	657.669348	1.50328469	0.12826886	11.7197946	1.0092E-31	1.0867E-30
ANKRD30BL	ENSG00000163046	protein_coding	1.40896762	2.25054613	0.45576449	4.93795846	7.8945E-07	1.8247E-06
TEKT4	ENSG00000163060	protein_coding	5.79559353	1.11578409	0.24877079	4.48518936	7.2849E-06	1.5366E-05
EN1	ENSG00000163064	protein_coding	24.6115948	3.58875076	0.26018367	13.7931435	2.8028E-43	5.0286E-42
SPATA18	ENSG00000163071	protein_coding	2630.36437	1.67038153	0.11413362	14.6353155	1.672E-48	3.7333E-47
INHBB	ENSG00000163083	protein_coding	4146.03017	3.80876196	0.15654667	24.3298823	9.468E-131	3.678E-128
ANKRD23	ENSG00000163126	protein_coding	50.8695587	1.59531443	0.13374783	11.927778	8.4807E-33	9.5944E-32

CTSS	ENSG00000163131	protein_coding	9207.44593	1.92542853	0.12023916	16.0133231	1.0315E-57	3.2409E-56
MSX1	ENSG00000163132	protein_coding	255.198606	1.36675847	0.11076506	12.3392562	5.5668E-35	6.9895E-34
TNFAIP8L2	ENSG00000163154	protein_coding	396.819671	2.25147531	0.11769735	19.1293621	1.4382E-81	1.0583E-79
RNF149	ENSG00000163162	protein_coding	5302.25493	1.31606924	0.06327287	20.7998984	4.3373E-96	5.6645E-94
SPRR3	ENSG00000163209	protein_coding	3.78351201	3.12128385	0.55518825	5.62202788	1.8873E-08	5.0113E-08
BMP10	ENSG00000163217	protein_coding	0.35248195	1.16994482	0.36325701	3.22070815	0.00127874	0.00213322
PGLYRP4	ENSG00000163218	protein_coding	1.50997787	1.87472966	0.36312854	5.16271636	2.4339E-07	5.8868E-07
ARHGAP25	ENSG00000163219	protein_coding	1794.02789	1.74912296	0.0808	21.6475624	6.408E-104	1.121E-101
S100A9	ENSG00000163220	protein_coding	1239.97741	1.05980921	0.15778496	6.71679473	1.8577E-11	6.2418E-11
S100A12	ENSG00000163221	protein_coding	46.1926941	1.28973836	0.19095017	6.75431882	1.4351E-11	4.8626E-11
TGFA	ENSG00000163235	protein_coding	8604.99826	1.66120887	0.13335391	12.4571438	1.2787E-35	1.6596E-34
TDRD10	ENSG00000163239	protein_coding	113.426861	1.15559021	0.13634416	8.47553875	2.3399E-17	1.1694E-16
NPPC	ENSG00000163273	protein_coding	27.2828434	1.12079038	0.3176182	3.52873481	0.00041755	0.00073467
ALPI	ENSG00000163295	protein_coding	615.895822	2.4319531	0.34385438	7.0726251	1.5203E-12	5.5294E-12
LENEP	ENSG00000163352	protein_coding	2.64186392	1.05237371	0.23086284	4.55843695	5.1536E-06	1.1028E-05
DCST2	ENSG00000163354	protein_coding	41.7839495	1.9933723	0.15948256	12.498999	7.5597E-36	9.8956E-35
DCST1	ENSG00000163357	protein_coding	17.1952609	2.48464051	0.20311968	12.2323966	2.0869E-34	2.547E-33
COL6A3	ENSG00000163359	protein_coding	12713.8184	1.40349573	0.18370613	7.63989614	2.174E-14	8.9547E-14
IGFN1	ENSG00000163395	protein_coding	195.173794	4.12283407	0.37728132	10.9277451	8.4933E-28	7.5483E-27
FSTL1	ENSG00000163430	protein_coding	29195.5946	1.35653146	0.11429108	11.8690929	1.7132E-32	1.9108E-31
PDCL2	ENSG00000163440	protein_coding	1.35576256	2.80078037	0.50105221	5.58979749	2.2733E-08	5.9906E-08
TRIM46	ENSG00000163462	protein_coding	120.319313	1.92697729	0.17478753	11.0246843	2.9054E-28	2.6426E-27
CCDC141	ENSG00000163492	protein_coding	119.240187	2.11772333	0.15034802	14.0854752	4.6651E-45	8.9779E-44
CRYBA2	ENSG00000163499	protein_coding	0.70118471	1.86736159	0.54494587	3.42669188	0.00061098	0.0010555
EOMES	ENSG00000163508	protein_coding	388.444508	3.50248299	0.18174104	19.2718328	9.2598E-83	7.0746E-81

RETNLB	ENSG00000163515	protein_coding	0.76383373	1.90768373	0.54813876	3.48029343	0.00050086	0.00087414
ANKZF1	ENSG00000163516	protein_coding	2584.55114	1.44418997	0.09738781	14.8292691	9.4759E-50	2.2219E-48
FCRL4	ENSG00000163518	protein_coding	1.73196907	2.69920739	0.43381297	6.22205319	4.9069E-10	1.4861E-09
TRAT1	ENSG00000163519	protein_coding	147.290642	2.34704665	0.17251513	13.6048745	3.7461E-42	6.3838E-41
GLB1L	ENSG00000163521	protein_coding	2932.64641	1.12974069	0.11044766	10.2287429	1.4742E-24	1.1014E-23
SPTA1	ENSG00000163554	protein_coding	27.999516	2.67294759	0.20128957	13.279116	3.0598E-40	4.8235E-39
MNDA	ENSG00000163563	protein_coding	919.412819	1.7839807	0.12356342	14.4377741	2.9935E-47	6.3238E-46
PYHIN1	ENSG00000163564	protein_coding	247.097615	2.95879361	0.15816904	18.7065288	4.3799E-78	2.8601E-76
IFI16	ENSG00000163565	protein_coding	5999.56423	1.60403374	0.08711636	18.4125429	1.0421E-75	6.2042E-74
AIM2	ENSG00000163568	protein_coding	135.309728	3.04706217	0.19581735	15.5607366	1.3454E-54	3.7791E-53
RPL22L1	ENSG00000163584	protein_coding	1181.61505	1.39833843	0.11291604	12.3838778	3.1952E-35	4.0575E-34
CTLA4	ENSG00000163599	protein_coding	97.2207344	2.94430362	0.1893004	15.5536048	1.504E-54	4.209E-53
ICOS	ENSG00000163600	protein_coding	96.3059547	3.35315383	0.18892704	17.7484065	1.7731E-70	8.6807E-69
CD200R1	ENSG00000163606	protein_coding	225.718111	2.24957466	0.16629305	13.5277728	1.0722E-41	1.79E-40
CLRN1	ENSG00000163646	protein_coding	1.07819255	2.13113476	0.38691588	5.50800534	3.6292E-08	9.403E-08
DCLK3	ENSG00000163673	protein_coding	55.1306251	3.29772376	0.17786387	18.5407176	9.692E-77	6.0613E-75
CFAP20DC	ENSG00000163689	protein_coding	273.099115	1.65460764	0.11949038	13.8472039	1.3226E-43	2.4126E-42
FANCD2OS	ENSG00000163705	protein_coding	1.87556275	1.75201242	0.34346763	5.10095359	3.3795E-07	8.0729E-07
CXCL5	ENSG00000163735	protein_coding	602.113684	4.99310319	0.32763325	15.239916	1.9212E-52	5.0124E-51
PF4	ENSG00000163737	protein_coding	13.6755411	1.01070121	0.20199561	5.00358002	5.6275E-07	1.3183E-06
CXCL1	ENSG00000163739	protein_coding	527.323188	1.37502075	0.2448298	5.61623108	1.9517E-08	5.173E-08
CPA3	ENSG00000163751	protein_coding	628.385229	1.30339434	0.17558393	7.42319843	1.1433E-13	4.4927E-13
DNAJC5G	ENSG00000163793	protein_coding	1.61391524	1.60027424	0.32447026	4.93195969	8.1409E-07	1.8793E-06
UCN	ENSG00000163794	protein_coding	55.0766206	2.58554758	0.15268402	16.933976	2.5272E-64	9.8847E-63
PLB1	ENSG00000163803	protein_coding	279.762012	1.31357424	0.10371579	12.6651328	9.2264E-37	1.2625E-35

SPDYA	ENSG00000163806	protein_coding	44.7043942	1.66671215	0.15006126	11.1068783	1.1615E-28	1.0778E-27
KIF15	ENSG00000163808	protein_coding	115.165321	1.20751189	0.1297386	9.30726807	1.3116E-20	7.9197E-20
CCR1	ENSG00000163823	protein_coding	971.151892	1.11622777	0.13965267	7.99288518	1.3182E-15	5.8993E-15
YEATS2	ENSG00000163872	protein_coding	3255.86699	1.08002257	0.06349649	17.0091707	7.0225E-65	2.8115E-63
GRIK3	ENSG00000163873	protein_coding	1575.06531	4.99828464	0.18533039	26.9695907	3.361E-160	2.963E-157
CAMK2N2	ENSG00000163888	protein_coding	27.9315753	1.90926732	0.19495349	9.79345058	1.2012E-22	8.0921E-22
HEYL	ENSG00000163909	protein_coding	4250.13715	1.61045588	0.13075754	12.3163524	7.3965E-35	9.2387E-34
RHO	ENSG00000163914	protein_coding	2.98725156	1.34001191	0.25279499	5.30078502	1.1531E-07	2.8661E-07
ZDHHC19	ENSG00000163958	protein_coding	10.9907777	1.75959476	0.17065098	10.3110734	6.2796E-25	4.7707E-24
SLC9B2	ENSG00000164038	protein_coding	376.707266	1.08979535	0.10106096	10.7835438	4.1171E-27	3.5363E-26
CAMP	ENSG00000164047	protein_coding	7.50953236	1.39753151	0.23942227	5.83709919	5.3117E-09	1.4773E-08
SPRY1	ENSG00000164056	protein_coding	6748.37552	1.32484988	0.11613434	11.4079081	3.818E-30	3.7974E-29
CAMKV	ENSG00000164076	protein_coding	3.89561037	1.26689274	0.38677122	3.27556105	0.00105452	0.00177598
GRM2	ENSG00000164082	protein_coding	27.3170236	1.29976251	0.14724937	8.82694796	1.0757E-18	5.8309E-18
PITX2	ENSG00000164093	protein_coding	111.180444	1.49301774	0.3290356	4.53755686	5.691E-06	1.2129E-05
SAP30	ENSG00000164105	protein_coding	1006.19051	2.46961986	0.08663017	28.5076202	9.425E-179	1.394E-175
TMEM155	ENSG00000164112	protein_coding	97.5278434	5.12386849	0.21233103	24.1315102	1.168E-128	4.215E-126
ADAD1	ENSG00000164113	protein_coding	0.95659018	1.95648693	0.51851129	3.77327742	0.00016112	0.00029661
C4orf45	ENSG00000164123	protein_coding	4.14411007	1.08163247	0.20817699	5.19573501	2.0391E-07	4.9642E-07
GASK1B	ENSG00000164125	protein_coding	5729.10276	1.20403785	0.12473292	9.65292789	4.7772E-22	3.116E-21
IL15	ENSG00000164136	protein_coding	554.517986	1.01726785	0.08590457	11.8418364	2.372E-32	2.6322E-31
SLC45A2	ENSG00000164175	protein_coding	34.0866773	1.7198456	0.19487779	8.82525219	1.0921E-18	5.9178E-18
EDIL3	ENSG00000164176	protein_coding	7362.42026	1.14866536	0.15318372	7.49861275	6.4497E-14	2.5784E-13
ZNF474	ENSG00000164185	protein_coding	17.7362187	1.28928866	0.16231882	7.94294014	1.9744E-15	8.734E-15
SCGB3A2	ENSG00000164265	protein_coding	38.2438098	6.13059645	0.36440965	16.823365	1.6454E-63	6.2752E-62

ESM1	ENSG00000164283	protein_coding	14157.6658	3.55911712	0.14284248	24.9163778	4.945E-137	2.337E-134
ERAP2	ENSG00000164308	protein_coding	5132.18383	1.06074378	0.18890093	5.6153444	1.9617E-08	5.1987E-08
EGFLAM	ENSG00000164318	protein_coding	801.57157	1.46308873	0.13926551	10.5057504	8.1274E-26	6.5146E-25
FAM170A	ENSG00000164334	protein_coding	1.83074538	1.90049277	0.31068197	6.11716472	9.5255E-10	2.8181E-09
TLR3	ENSG00000164342	protein_coding	5633.44541	1.50335251	0.11067321	13.5837078	5.0028E-42	8.4719E-41
KLKB1	ENSG00000164344	protein_coding	285.1239	1.43321953	0.17854172	8.02736485	9.9589E-16	4.4845E-15
TERT	ENSG00000164362	protein_coding	15.7756361	6.01161945	0.41998345	14.3139437	1.7906E-46	3.6694E-45
CCDC127	ENSG00000164366	protein_coding	2434.47396	1.04307083	0.06332285	16.4722655	5.8055E-61	2.0269E-59
IL3	ENSG00000164399	protein_coding	0.47799765	1.58997293	0.44332239	3.58649363	0.00033515	0.00059559
CSF2	ENSG00000164400	protein_coding	2.59691085	1.50505717	0.3038966	4.95253046	7.3255E-07	1.6979E-06
SHROOM1	ENSG00000164403	protein_coding	1038.54087	1.12739635	0.08982395	12.5511776	3.9164E-36	5.1963E-35
CGAS	ENSG00000164430	protein_coding	337.064832	1.38730083	0.08788887	15.7847167	3.9643E-56	1.1679E-54
FABP7	ENSG00000164434	protein_coding	11698.8842	7.44033575	0.32416086	22.9526036	1.388E-116	3.42E-114
TXLNB	ENSG00000164440	protein_coding	95.4872386	1.84986848	0.14672288	12.6079071	1.91E-36	2.5721E-35
SAMD3	ENSG00000164483	protein_coding	151.642709	2.32332188	0.12904272	18.0042851	1.8031E-72	9.4899E-71
TMEM200A	ENSG00000164484	protein_coding	2761.44367	1.29025049	0.12966292	9.95080579	2.5015E-23	1.7435E-22
IL22RA2	ENSG00000164485	protein_coding	2.23525425	1.52055561	0.42587903	3.57039323	0.00035645	0.00063171
H2AC1	ENSG00000164508	protein_coding	1.81669209	1.47818427	0.3162064	4.6747449	2.9432E-06	6.4459E-06
IL31RA	ENSG00000164509	protein_coding	17.3086	1.36754341	0.21557833	6.34360337	2.2445E-10	6.9521E-10
TBX20	ENSG00000164532	protein_coding	1.37065099	2.46966366	0.4194213	5.88826477	3.9027E-09	1.0968E-08
RPS14	ENSG00000164587	protein_coding	46884.6583	1.07671582	0.0888408	12.1196103	8.3154E-34	9.906E-33
GPR85	ENSG00000164604	protein_coding	222.329145	1.49648508	0.13895821	10.7693177	4.8055E-27	4.1137E-26
PTTG1	ENSG00000164611	protein_coding	403.96782	2.36765899	0.1444087	16.3955427	2.0579E-60	7.0825E-59
BMPER	ENSG00000164619	protein_coding	96.2387806	1.02365952	0.18834297	5.43508206	5.4771E-08	1.4001E-07
SLC29A4	ENSG00000164638	protein_coding	2589.14979	2.18878747	0.13877636	15.7720481	4.8453E-56	1.4256E-54

CDCA7L	ENSG00000164649	protein_coding	1135.31295	1.76621444	0.11184553	15.791552	3.5572E-56	1.0507E-54
SP8	ENSG00000164651	protein_coding	7.02737659	2.80820453	0.57348479	4.89673761	9.7441E-07	2.2314E-06
SYTL3	ENSG00000164674	protein_coding	777.749354	1.1931649	0.1079534	11.0525919	2.1298E-28	1.9491E-27
HEY1	ENSG00000164683	protein_coding	1140.95574	1.58094297	0.10937115	14.454844	2.3366E-47	4.9566E-46
FABP5	ENSG00000164687	protein_coding	569.664699	1.92008627	0.12935681	14.8433337	7.6841E-50	1.8064E-48
TAGAP	ENSG00000164691	protein_coding	684.060347	1.96858086	0.12420669	15.8492336	1.4231E-56	4.2581E-55
COL1A2	ENSG00000164692	protein_coding	49430.8232	1.73374601	0.17142143	10.1139398	4.7918E-24	3.4745E-23
SLC13A4	ENSG00000164707	protein_coding	98.3363583	1.2881933	0.15070223	8.54793809	1.2531E-17	6.3584E-17
SLC35G3	ENSG00000164729	protein_coding	3.67603396	3.68252603	0.43613859	8.44347672	3.0804E-17	1.5274E-16
PHKG1	ENSG00000164776	protein_coding	66.8173756	1.84736782	0.12319409	14.9955877	7.8465E-51	1.9204E-49
KCNV1	ENSG00000164794	protein_coding	85.4030525	1.67135911	0.25726713	6.49659012	8.2161E-11	2.6344E-10
DEFA4	ENSG00000164821	protein_coding	2.19759346	1.08172784	0.39806555	2.71746161	0.00657848	0.01009724
MICALL2	ENSG00000164877	protein_coding	1533.28898	2.04930689	0.12130289	16.8941308	4.9698E-64	1.9193E-62
GBX1	ENSG00000164900	protein_coding	0.70842092	1.20524134	0.33018971	3.65014811	0.00026209	0.00047149
CTHRC1	ENSG00000164932	protein_coding	1557.9007	3.1432709	0.20862891	15.0663244	2.6971E-51	6.712E-50
DCSTAMP	ENSG00000164935	protein_coding	35.2196122	3.0029549	0.20110968	14.9319263	2.0428E-50	4.9286E-49
FAM219A	ENSG00000164970	protein_coding	2025.01747	1.05838205	0.05395952	19.6143704	1.1657E-85	9.9697E-84
DIRAS2	ENSG00000165023	protein_coding	1627.89876	2.9845051	0.16146193	18.4842648	2.7644E-76	1.7033E-74
ABCA1	ENSG00000165029	protein_coding	6707.06812	1.55542838	0.10010678	15.5376926	1.928E-54	5.3695E-53
ZMAT4	ENSG00000165061	protein_coding	285.930145	3.47138843	0.31693144	10.953121	6.4197E-28	5.7421E-27
NKX6-3	ENSG00000165066	protein_coding	1.78630223	2.79812397	0.41721313	6.70670153	1.9907E-11	6.6762E-11
PRSS37	ENSG00000165076	protein_coding	8.78344381	2.3369864	0.21924685	10.6591562	1.5802E-26	1.3171E-25
CPA6	ENSG00000165078	protein_coding	55.5440381	2.51877061	0.16021032	15.7216503	1.0749E-55	3.1304E-54
CFAP47	ENSG00000165164	protein_coding	73.3694839	1.21621795	0.29334214	4.14607307	3.3823E-05	6.6908E-05
CYBB	ENSG00000165168	protein_coding	4890.47635	1.79780763	0.14856833	12.1008805	1.0448E-33	1.2364E-32

METTL27	ENSG00000165171	protein_coding	496.847901	1.06016596	0.13987898	7.57916591	3.4778E-14	1.4129E-13
OR1Q1	ENSG00000165202	protein_coding	1.00300367	1.58855269	0.44623142	3.55993013	0.00037095	0.00065648
OR1K1	ENSG00000165204	protein_coding	1.15828985	1.96726518	0.38938519	5.05223415	4.3667E-07	1.0333E-06
SLITRK5	ENSG00000165300	protein_coding	394.747726	3.40236248	0.24645156	13.8054005	2.3645E-43	4.2591E-42
MELK	ENSG00000165304	protein_coding	212.514168	2.37125497	0.14543574	16.304486	9.1707E-60	3.0866E-58
INTS6L	ENSG00000165359	protein_coding	706.739765	1.75774039	0.09737554	18.0511494	7.7266E-73	4.1281E-71
TSHR	ENSG00000165409	protein_coding	69.3919781	2.37673957	0.16558065	14.3539693	1.0061E-46	2.0784E-45
FOLR2	ENSG00000165457	protein_coding	1316.39118	1.38423327	0.13864646	9.98390627	1.7927E-23	1.2596E-22
PHOX2A	ENSG00000165462	protein_coding	1.33682868	1.2190853	0.26359426	4.6248553	3.7486E-06	8.1279E-06
MBL2	ENSG00000165471	protein_coding	5.15790945	3.75726474	0.51122878	7.34947811	1.9898E-13	7.6839E-13
GJB2	ENSG00000165474	protein_coding	1772.98254	1.30284098	0.20081752	6.48768597	8.7165E-11	2.7895E-10
SKA3	ENSG00000165480	protein_coding	128.477804	2.07635329	0.12498877	16.6123192	5.6756E-62	2.0358E-60
RPL10L	ENSG00000165496	protein_coding	1.30273773	1.5394278	0.30028372	5.12657755	2.9506E-07	7.0863E-07
DEPP1	ENSG00000165507	protein_coding	20270.5745	2.24774533	0.11769082	19.0987313	2.587E-81	1.8914E-79
MAGEC3	ENSG00000165509	protein_coding	6.37827744	2.65350336	0.36849278	7.20096422	5.9788E-13	2.2321E-12
CDX2	ENSG00000165556	protein_coding	1.0700643	1.52026092	0.42822402	3.55015328	0.00038501	0.00068012
SSX5	ENSG00000165583	protein_coding	0.82980298	1.67223723	0.44608267	3.74871601	0.00017774	0.00032586
SSX3	ENSG00000165584	protein_coding	1.08720418	1.92475079	0.58248617	3.30437165	0.0009519	0.00161052
OTX2	ENSG00000165588	protein_coding	0.87019895	1.48935074	0.49272465	3.02268365	0.00250544	0.00404251
SOHLH1	ENSG00000165643	protein_coding	0.9165333	2.2956812	0.44782511	5.12628956	2.9551E-07	7.0961E-07
SLC18A2	ENSG00000165646	protein_coding	69.2607785	1.03782277	0.1515037	6.85014781	7.3774E-12	2.5531E-11
CLEC1B	ENSG00000165682	protein_coding	11.7003883	2.80482229	0.19592025	14.3161432	1.7349E-46	3.5567E-45
OR4K2	ENSG00000165762	protein_coding	0.71254873	2.07652949	0.55527911	3.73961395	0.0001843	0.00033741
C12orf50	ENSG00000165805	protein_coding	3.63974553	1.44797336	0.23576971	6.14147315	8.176E-10	2.4317E-09
BTNL9	ENSG00000165810	protein_coding	3338.23235	2.15816159	0.15829325	13.6339457	2.516E-42	4.3294E-41

ERICH6B	ENSG00000165837	protein_coding	15.8112312	1.69897282	0.15645416	10.8592371	1.8025E-27	1.573E-26
E2F7	ENSG00000165891	protein_coding	109.01547	2.41928402	0.16524056	14.6409818	1.5383E-48	3.4365E-47
ARHGAP42	ENSG00000165895	protein_coding	2551.91735	1.25029793	0.09988451	12.5174351	5.9941E-36	7.8868E-35
AGBL2	ENSG00000165923	protein_coding	154.464357	1.48047882	0.12726364	11.6331639	2.7954E-31	2.9499E-30
SMCO2	ENSG00000165935	protein_coding	10.1785122	1.4611147	0.17772913	8.22101968	2.0178E-16	9.4811E-16
IFI27	ENSG00000165949	protein_coding	7703.12838	1.47195281	0.13433655	10.9572026	6.1367E-28	5.4933E-27
SERPINA12	ENSG00000165953	protein_coding	9.73696874	4.06504815	0.43843533	9.27171674	1.8317E-20	1.0965E-19
SLC6A5	ENSG00000165970	protein_coding	1.64561315	1.425181	0.46059831	3.09419503	0.00197348	0.00321978
TAF1D	ENSG00000166012	protein_coding	3427.537	1.49208615	0.07774367	19.1923821	4.2854E-82	3.1995E-80
PASD1	ENSG00000166049	protein_coding	1.21175923	2.51100876	0.78854017	3.18437647	0.00145066	0.00240436
TMCO5A	ENSG00000166069	protein_coding	0.55253016	1.25788335	0.46901502	2.68196817	0.00731904	0.01116105
GPR176	ENSG00000166073	protein_coding	1814.09978	1.42014402	0.09894412	14.3529901	1.0204E-46	2.1071E-45
GLB1L3	ENSG00000166105	protein_coding	104.467466	1.30702076	0.32430201	4.03025791	5.5716E-05	0.00010766
IKBIP	ENSG00000166130	protein_coding	3083.68324	1.81279981	0.08917793	20.3278978	7.2858E-92	7.8832E-90
PPP1R14D	ENSG00000166143	protein_coding	381.407659	2.6506408	0.27041695	9.80205111	1.1032E-22	7.4427E-22
C16orf78	ENSG00000166152	protein_coding	1.79872322	1.68044765	0.29377269	5.72023099	1.0638E-08	2.8819E-08
SPIC	ENSG00000166211	protein_coding	9.69594399	3.82701128	0.31107905	12.3023756	8.7949E-35	1.0947E-33
MYRFL	ENSG00000166268	protein_coding	159.3067	1.93753905	0.19810011	9.78060569	1.3639E-22	9.1583E-22
C2	ENSG00000166278	protein_coding	2687.84038	2.17245636	0.19713451	11.0201726	3.0547E-28	2.7757E-27
PLEKHF1	ENSG00000166289	protein_coding	420.713785	1.01209435	0.11069206	9.14333265	6.0557E-20	3.5264E-19
TMEM100	ENSG00000166292	protein_coding	332.754576	1.25305089	0.20530863	6.10325498	1.0393E-09	3.0664E-09
CCDC182	ENSG00000166329	protein_coding	0.56630345	1.00524183	0.3558416	2.82496998	0.0047285	0.00738457
NETO1	ENSG00000166342	protein_coding	40.6719796	2.52831684	0.22591454	11.1914746	4.4891E-29	4.2632E-28
MSS51	ENSG00000166343	protein_coding	78.0480047	1.4570509	0.1326932	10.9805997	4.7377E-28	4.2626E-27
PLD4	ENSG00000166428	protein_coding	666.75062	2.25063798	0.1476471	15.2433601	1.8225E-52	4.7659E-51

ZMAT1	ENSG00000166432	protein_coding	1647.16673	1.13550242	0.12418699	9.1434891	6.0469E-20	3.5217E-19
TMEM130	ENSG00000166448	protein_coding	2354.57627	1.32681396	0.26170023	5.06997627	3.9787E-07	9.4481E-07
CCDC68	ENSG00000166510	protein_coding	502.550219	1.01112821	0.14965015	6.75661342	1.4125E-11	4.7876E-11
CLEC4E	ENSG00000166523	protein_coding	288.730118	1.24979426	0.15587035	8.0181652	1.0734E-15	4.823E-15
A2ML1	ENSG00000166535	protein_coding	10.8317268	1.19541077	0.19447857	6.1467481	7.9088E-10	2.3557E-09
SLC38A8	ENSG00000166558	protein_coding	4.88769456	1.45151684	0.46012744	3.15459744	0.0016072	0.00265087
GALR1	ENSG00000166573	protein_coding	69.2426644	1.10792539	0.21623331	5.12374975	2.9952E-07	7.1889E-07
RRAD	ENSG00000166592	protein_coding	3928.45177	1.85872832	0.1668378	11.1409307	7.9286E-29	7.4403E-28
TVP23A	ENSG00000166676	protein_coding	107.846388	1.32570157	0.11906207	11.1345416	8.5182E-29	7.9788E-28
TMPRSS5	ENSG00000166682	protein_coding	29.8994043	1.82993906	0.15275229	11.9797816	4.5352E-33	5.2131E-32
B2M	ENSG00000166710	protein_coding	221039.862	1.32839165	0.07766326	17.104505	1.3736E-65	5.6122E-64
HTR3A	ENSG00000166736	protein_coding	11.6997968	1.27003127	0.31043108	4.09118601	4.2917E-05	8.4017E-05
NNMT	ENSG00000166741	protein_coding	41585.0759	3.90624263	0.16634215	23.4831805	6.059E-122	1.792E-119
CATSPER2	ENSG00000166762	protein_coding	200.048566	1.17498652	0.13583094	8.6503599	5.1337E-18	2.6694E-17
YPEL4	ENSG00000166793	protein_coding	138.841759	2.65096483	0.1328422	19.9557426	1.3362E-88	1.2682E-86
PCLAF	ENSG00000166803	protein_coding	304.354733	2.21498757	0.12535205	17.6701349	7.1226E-70	3.4154E-68
MESP1	ENSG00000166823	protein_coding	111.754601	1.2681933	0.12897783	9.83264531	8.1451E-23	5.5282E-22
PLK1	ENSG00000166851	protein_coding	310.212993	2.37603348	0.14520637	16.3631491	3.5052E-60	1.1973E-58
CACNG2	ENSG00000166862	protein_coding	1.88584953	3.03790536	0.4712435	6.4465724	1.1441E-10	3.6255E-10
TAC3	ENSG00000166863	protein_coding	3.98521426	1.42827645	0.30722504	4.6489584	3.3362E-06	7.2711E-06
MYO1A	ENSG00000166866	protein_coding	30.7116366	2.71619548	0.2074583	13.0927301	3.6235E-39	5.4801E-38
C15orf48	ENSG00000166920	protein_coding	314.59717	1.5334113	0.16134503	9.50392674	2.0212E-21	1.2759E-20
SCG5	ENSG00000166922	protein_coding	240.552182	1.61521446	0.21994509	7.34371684	2.0774E-13	8.0073E-13
MS4A6E	ENSG00000166926	protein_coding	3.27015044	2.5514107	0.30611139	8.33490946	7.7558E-17	3.7487E-16
MS4A7	ENSG00000166927	protein_coding	3454.38048	2.4108785	0.12060904	19.9892017	6.8381E-89	6.5854E-87

MS4A14	ENSG00000166928	protein_coding	228.014153	3.19372644	0.15368826	20.7805488	6.4912E-96	8.3821E-94
EPB42	ENSG00000166947	protein_coding	11.9635289	1.7025828	0.20413607	8.34043085	7.402E-17	3.5853E-16
TGM6	ENSG00000166948	protein_coding	0.71029717	2.0236613	0.53160419	3.80670683	0.00014083	0.00026093
MS4A15	ENSG00000166961	protein_coding	3.58263714	3.36014587	0.36460055	9.21596489	3.0849E-20	1.8195E-19
MAPRE2	ENSG00000166974	protein_coding	7077.95506	1.00152467	0.07762912	12.9014046	4.4197E-38	6.3832E-37
EVA1C	ENSG00000166979	protein_coding	1291.63036	1.14495542	0.10236212	11.1853434	4.8104E-29	4.5636E-28
MEI1	ENSG00000167077	protein_coding	158.09275	1.37277765	0.16100696	8.52620074	1.5123E-17	7.641E-17
TTC16	ENSG00000167094	protein_coding	63.3040294	1.64278389	0.15376935	10.683429	1.2169E-26	1.0202E-25
SUN5	ENSG00000167098	protein_coding	0.48537894	1.54451626	0.5382553	2.86948639	0.00411139	0.00647086
SAMD14	ENSG00000167100	protein_coding	467.994709	1.6045791	0.10036772	15.9870041	1.5742E-57	4.9058E-56
TMEM92	ENSG00000167105	protein_coding	472.124048	2.76977741	0.24621591	11.2493842	2.3321E-29	2.2483E-28
ANKRD40CL	ENSG00000167117	protein_coding	5.64703755	1.30167032	0.26460836	4.91923354	8.6884E-07	2.0002E-06
TBC1D21	ENSG00000167139	protein_coding	0.80238767	1.5354253	0.40490556	3.79205784	0.0001494	0.0002761
PRRX2	ENSG00000167157	protein_coding	74.0017034	1.20382636	0.18952887	6.35167814	2.1298E-10	6.6083E-10
UGT1A6	ENSG00000167165	protein_coding	2160.92034	1.07144077	0.21880865	4.89670213	9.7458E-07	2.2317E-06
C16orf92	ENSG00000167194	protein_coding	0.64678832	2.00686106	0.46040006	4.35895044	1.3069E-05	2.6893E-05
NOD2	ENSG00000167207	protein_coding	272.277158	2.44460529	0.14145408	17.2819713	6.4312E-67	2.763E-65
SNX20	ENSG00000167208	protein_coding	564.702156	2.44163153	0.13793054	17.7018917	4.0546E-70	1.9606E-68
DPEP2	ENSG00000167261	protein_coding	325.8847	2.2665896	0.10502443	21.5815454	2.678E-103	4.597E-101
ENGASE	ENSG00000167280	protein_coding	1250.07621	1.04095567	0.10177127	10.2283848	1.4797E-24	1.1051E-23
CD3D	ENSG00000167286	protein_coding	583.070384	2.87454143	0.16994887	16.9141539	3.5386E-64	1.3794E-62
TEPSIN	ENSG00000167302	protein_coding	544.17019	1.13392588	0.09316297	12.1714228	4.4132E-34	5.3169E-33
OR51E2	ENSG00000167332	protein_coding	181.526089	2.19876203	0.16597611	13.2474612	4.6673E-40	7.2798E-39
MMP26	ENSG00000167346	protein_coding	2.5833436	1.86259035	0.53541763	3.47876173	0.00050374	0.00087888
OR51I1	ENSG00000167359	protein_coding	0.48988618	1.30713939	0.43302826	3.01860069	0.00253945	0.00409407

OR51Q1	ENSG00000167360	protein_coding	0.48251496	1.26358681	0.52200822	2.42062629	0.0154938	0.02257345
PRRT2	ENSG00000167371	protein_coding	213.310286	2.4117288	0.15786328	15.2773263	1.0829E-52	2.858E-51
VKORC1	ENSG00000167397	protein_coding	3575.45178	1.15555342	0.0997593	11.5834153	5.0013E-31	5.1987E-30
JSRP1	ENSG00000167476	protein_coding	72.1829715	2.30410962	0.22477316	10.2508217	1.1734E-24	8.8225E-24
CDT1	ENSG00000167513	protein_coding	321.661348	2.27020742	0.12566639	18.0653507	5.9741E-73	3.2105E-71
RPL13	ENSG00000167526	protein_coding	56727.9402	1.13793425	0.09867386	11.532277	9.0712E-31	9.2965E-30
CORO6	ENSG00000167549	protein_coding	93.5475598	2.14481627	0.21049073	10.1895998	2.2067E-24	1.6339E-23
RHEBL1	ENSG00000167550	protein_coding	55.863262	1.90595068	0.10183655	18.7157816	3.6818E-78	2.4077E-76
TUBA1A	ENSG00000167552	protein_coding	9245.80805	1.015896	0.08853122	11.4750035	1.7617E-30	1.7859E-29
AXL	ENSG00000167601	protein_coding	6793.39721	1.58145116	0.10639997	14.8632669	5.7073E-50	1.3528E-48
NFKBID	ENSG00000167604	protein_coding	454.696429	2.16619778	0.14557785	14.8799962	4.4452E-50	1.0575E-48
ANKRD33	ENSG00000167612	protein_coding	9.54783443	3.20248355	0.29474749	10.8651767	1.689E-27	1.4761E-26
LAIR1	ENSG00000167613	protein_coding	2356.66228	2.63163836	0.12146169	21.6664072	4.257E-104	7.535E-102
TTYH1	ENSG00000167614	protein_coding	63.9488212	1.42178877	0.18449694	7.7062999	1.2952E-14	5.4137E-14
LENG8	ENSG00000167615	protein_coding	8187.17982	1.55495048	0.12968632	11.9900886	4.0048E-33	4.6138E-32
LAIR2	ENSG00000167618	protein_coding	30.1848859	3.48481895	0.23560982	14.7906354	1.6836E-49	3.8998E-48
TMEM145	ENSG00000167619	protein_coding	87.6190383	3.86458256	0.24416496	15.8277522	2.0025E-56	5.9608E-55
KIR3DL1	ENSG00000167633	protein_coding	16.1604984	2.19518062	0.17737536	12.3759051	3.5289E-35	4.47E-34
NLRP7	ENSG00000167634	protein_coding	11.5303701	2.13798585	0.24571489	8.70108394	3.2873E-18	1.7293E-17
C19orf33	ENSG00000167644	protein_coding	1667.38312	2.52935483	0.19727023	12.8217764	1.2383E-37	1.758E-36
LY6D	ENSG00000167656	protein_coding	5.01415531	1.50953548	0.41300913	3.6549688	0.00025721	0.00046312
TMIGD2	ENSG00000167664	protein_coding	30.3805884	2.17029737	0.1574359	13.7852759	3.1257E-43	5.5949E-42
SEMA6B	ENSG00000167680	protein_coding	1978.36782	1.62275589	0.11505953	14.10362	3.6078E-45	6.9848E-44
TLCD3A	ENSG00000167695	protein_coding	1602.49841	1.58301497	0.0988635	16.0121268	1.0515E-57	3.2993E-56
KIFC2	ENSG00000167702	protein_coding	639.141527	1.21174589	0.1309558	9.25309072	2.1809E-20	1.2991E-19

SERPINF2	ENSG00000167711	protein_coding	12640.8462	1.00968154	0.17992082	5.61181033	2.0022E-08	5.3032E-08
TRPV3	ENSG00000167723	protein_coding	54.6756392	1.25654133	0.18783478	6.68960967	2.2377E-11	7.4835E-11
KLK4	ENSG00000167749	protein_coding	82.7714648	3.97225015	0.49363489	8.04693964	8.489E-16	3.837E-15
KLK2	ENSG00000167751	protein_coding	3.31516109	2.26486345	0.42536897	5.32446798	1.0125E-07	2.5293E-07
KLK13	ENSG00000167759	protein_coding	8.47134157	2.29603642	0.33993901	6.75425985	1.4357E-11	4.8642E-11
ZNF83	ENSG00000167766	protein_coding	4297.14046	1.46935523	0.10548788	13.9291383	4.2141E-44	7.8305E-43
ANGPTL4	ENSG00000167772	protein_coding	44219.1493	4.93447731	0.16903232	29.1925082	2.414E-187	4.611E-184
TBX10	ENSG00000167800	protein_coding	4.37012668	1.02750383	0.22394119	4.58827522	4.4692E-06	9.6275E-06
CD300C	ENSG00000167850	protein_coding	173.732138	2.28126592	0.13076876	17.4450369	3.7548E-68	1.6858E-66
CD300A	ENSG00000167851	protein_coding	1234.87955	2.97114479	0.12521022	23.7292513	1.8E-124	5.769E-122
TMC8	ENSG00000167895	protein_coding	1067.68596	2.51096368	0.12664094	19.8274253	1.7265E-87	1.5829E-85
GSDMA	ENSG00000167914	protein_coding	56.8898543	1.41067048	0.1954953	7.2158793	5.3587E-13	2.0061E-12
NLRC3	ENSG00000167984	protein_coding	617.581296	1.305935	0.08875503	14.7139264	5.2472E-49	1.1926E-47
VWCE	ENSG00000167992	protein_coding	824.50341	2.75465317	0.20165526	13.6602101	1.7548E-42	3.0401E-41
BEST1	ENSG00000167995	protein_coding	426.968125	1.88551601	0.11236167	16.7807757	3.374E-63	1.2678E-61
ATG16L2	ENSG00000168010	protein_coding	1512.53423	2.30737153	0.13000573	17.7482293	1.7787E-70	8.6929E-69
CCDC88B	ENSG00000168071	protein_coding	990.467387	2.57286631	0.12656848	20.3278598	7.2914E-92	7.8832E-90
PBK	ENSG00000168078	protein_coding	149.251596	2.09597767	0.14426966	14.5281941	8.031E-48	1.7316E-46
OR1F1	ENSG00000168124	protein_coding	0.67256075	1.35478781	0.57298638	2.36443285	0.0180577	0.02606206
KCNJ4	ENSG00000168135	protein_coding	43.6690876	1.69167326	0.21681657	7.8023248	6.0777E-15	2.5999E-14
H3-4	ENSG00000168148	protein_coding	1.15258741	2.0153664	0.30692706	6.56627149	5.1591E-11	1.6787E-10
DDIT4	ENSG00000168209	protein_coding	15612.5539	2.15873495	0.11831152	18.2461946	2.2183E-74	1.2585E-72
PTGDR	ENSG00000168229	protein_coding	92.8509535	2.20058664	0.1325132	16.6065466	6.2489E-62	2.2379E-60
KCNV2	ENSG00000168263	protein_coding	8.73237131	2.40040809	0.20312853	11.8171886	3.1815E-32	3.5118E-31
PTF1A	ENSG00000168267	protein_coding	0.96542752	2.16301602	0.31740949	6.81459142	9.4532E-12	3.2426E-11

CX3CR1	ENSG00000168329	protein_coding	1263.42137	1.64980129	0.16481621	10.0099457	1.3783E-23	9.7488E-23
PPDPFL	ENSG00000168333	protein_coding	479.591404	8.02292934	0.43041912	18.6398071	1.5279E-77	9.8142E-76
XIRP1	ENSG00000168334	protein_coding	23.5448127	1.46759088	0.21826785	6.72380679	1.7704E-11	5.9542E-11
INSM2	ENSG00000168348	protein_coding	1.14884334	2.22709339	0.35955606	6.19400869	5.8653E-10	1.7647E-09
TAP1	ENSG00000168394	protein_coding	9013.59643	1.9201321	0.08668038	22.151865	1.001E-108	2.044E-106
MLKL	ENSG00000168404	protein_coding	1355.65695	1.91649597	0.0751714	25.4950133	2.239E-143	1.18E-140
KCNG4	ENSG00000168418	protein_coding	1.46244503	1.42864999	0.38706449	3.69098697	0.00022339	0.00040506
RHOH	ENSG00000168421	protein_coding	379.712818	2.21044296	0.14241378	15.521272	2.4906E-54	6.9029E-53
REEP4	ENSG00000168476	protein_coding	830.75063	1.13940048	0.08084751	14.0932036	4.1815E-45	8.0676E-44
BMP1	ENSG00000168487	protein_coding	3094.23166	1.85385505	0.10203891	18.1681193	9.2308E-74	5.1229E-72
MTCL1	ENSG00000168502	protein_coding	2075.41418	2.23586468	0.12240326	18.2663825	1.5328E-74	8.7613E-73
GBX2	ENSG00000168505	protein_coding	6.5184358	3.33272418	0.28813445	11.5665592	6.0875E-31	6.2964E-30
MYL1	ENSG00000168530	protein_coding	0.5183328	1.17307909	0.44458093	2.63861764	0.00832448	0.01260496
COL3A1	ENSG00000168542	protein_coding	41217.1953	1.30899328	0.1663478	7.86901463	3.5745E-15	1.552E-14
CRYGA	ENSG00000168582	protein_coding	0.47665311	1.50369105	0.6485574	2.31851654	0.02042127	0.02925593
ADAM29	ENSG00000168594	protein_coding	4.84112668	2.34932826	0.28228754	8.32246542	8.6152E-17	4.1532E-16
ADAM18	ENSG00000168619	protein_coding	109.605142	8.20773328	0.39565664	20.7445863	1.372E-95	1.7326E-93
WFDC13	ENSG00000168634	protein_coding	3.70391627	2.62562847	0.3459656	7.58927603	3.217E-14	1.3096E-13
KCTD19	ENSG00000168676	protein_coding	31.4499467	2.4392551	0.18443361	13.2256541	6.2392E-40	9.6756E-39
IL7R	ENSG00000168685	protein_coding	1674.74269	1.14496593	0.16373022	6.99300311	2.6906E-12	9.6045E-12
FAM178B	ENSG00000168754	protein_coding	12.7570351	1.0044041	0.17758956	5.65576091	1.5516E-08	4.1509E-08
SHOX2	ENSG00000168779	protein_coding	23.3939944	2.36215356	0.22189526	10.645354	1.8329E-26	1.524E-25
OR13J1	ENSG00000168828	protein_coding	2.00740588	1.52199631	0.28318105	5.37464036	7.6736E-08	1.9394E-07
VAMP5	ENSG00000168899	protein_coding	3024.39698	1.20408705	0.09506356	12.6661259	9.1104E-37	1.247E-35
BTNL3	ENSG00000168903	protein_coding	7.97359129	1.39451177	0.22252574	6.26674369	3.6868E-10	1.1263E-09

SLC35G2	ENSG00000168917	protein_coding	619.435319	1.40939517	0.08809332	15.9988882	1.3008E-57	4.0591E-56
INPP5D	ENSG00000168918	protein_coding	2919.82773	1.67916109	0.08807573	19.0649699	4.9356E-81	3.5687E-79
LGALS9	ENSG00000168961	protein_coding	2880.61776	2.17753355	0.10167356	21.4169099	9.295E-102	1.49E-99
JMJD7-PLA2G4B	ENSG00000168970	protein_coding	212.689784	1.5304464	0.14070385	10.8770753	1.4824E-27	1.2996E-26
CPLX1	ENSG00000168993	protein_coding	222.026713	1.01371598	0.14576006	6.95468985	3.5334E-12	1.2495E-11
PXDC1	ENSG00000168994	protein_coding	3587.29612	1.1609111	0.07983092	14.5421241	6.5527E-48	1.4209E-46
SIGLEC7	ENSG00000168995	protein_coding	183.67627	2.11125206	0.13774559	15.3271841	5.0334E-53	1.3384E-51
NTSR2	ENSG00000169006	protein_coding	1.02741865	2.20458716	0.50170955	4.39415024	1.1121E-05	2.3046E-05
VCX3A	ENSG00000169059	protein_coding	0.98925217	1.3691795	0.40123213	3.41243731	0.00064385	0.00110986
VXN	ENSG00000169085	protein_coding	301.267827	1.55107739	0.16155964	9.60064872	7.9443E-22	5.1263E-21
IL13	ENSG00000169194	protein_coding	3.49077041	1.81380651	0.27264461	6.65264025	2.8788E-11	9.5497E-11
NPIPB12	ENSG00000169203	protein_coding	20.5267062	2.11784186	0.18068567	11.7211389	9.9328E-32	1.0701E-30
RGS14	ENSG00000169220	protein_coding	2455.35217	1.35186102	0.10695272	12.6398007	1.2738E-36	1.7301E-35
RAB24	ENSG00000169228	protein_coding	792.542131	1.64853791	0.10252705	16.0790528	3.5781E-58	1.1414E-56
THBS3	ENSG00000169231	protein_coding	1655.87288	1.22710087	0.09349255	13.1251193	2.3641E-39	3.5992E-38
EFNA1	ENSG00000169242	protein_coding	6887.2879	1.21433479	0.09798328	12.3932859	2.8416E-35	3.6274E-34
CXCL10	ENSG00000169245	protein_coding	2100.5488	3.39225445	0.18315671	18.5210491	1.3969E-76	8.6885E-75
NPIPB3	ENSG00000169246	protein_coding	109.907421	2.14756376	0.15200076	14.1286387	2.5299E-45	4.9287E-44
CXCL11	ENSG00000169248	protein_coding	490.95264	3.50377719	0.19536375	17.9346335	6.328E-72	3.2667E-70
GPRIN1	ENSG00000169258	protein_coding	305.294969	2.17066532	0.12947049	16.7657155	4.3475E-63	1.6242E-61
KCNAB1	ENSG00000169282	protein_coding	872.204122	1.44670904	0.12884279	11.228483	2.9551E-29	2.8381E-28
NR0B1	ENSG00000169297	protein_coding	8.2454059	2.27398681	0.57169013	3.97765622	6.9598E-05	0.00013306
P2RY12	ENSG00000169313	protein_coding	156.642292	1.70668443	0.16380776	10.4188253	2.0345E-25	1.5948E-24
RNASE2	ENSG00000169385	protein_coding	80.7027662	2.43055374	0.1763436	13.7830559	3.2233E-43	5.7652E-42
RNASE3	ENSG00000169397	protein_coding	7.1290716	2.51162737	0.22724401	11.052557	2.1306E-28	1.9494E-27

PTAFR	ENSG00000169403	protein_coding	1337.12609	1.41607041	0.12664813	11.1811394	5.0438E-29	4.7811E-28
RNASE6	ENSG00000169413	protein_coding	1139.87141	1.52179594	0.11525021	13.2042789	8.289E-40	1.2802E-38
KCNK9	ENSG00000169427	protein_coding	250.430655	4.55094984	0.22408863	20.3087049	1.0771E-91	1.1509E-89
SCN9A	ENSG00000169432	protein_coding	937.788823	1.32293002	0.17835395	7.41744184	1.194E-13	4.6875E-13
RASSF6	ENSG00000169435	protein_coding	2748.42502	1.23756716	0.11901961	10.398011	2.5316E-25	1.9754E-24
CD52	ENSG00000169442	protein_coding	1308.67504	2.09564527	0.13749615	15.2414838	1.8756E-52	4.8992E-51
SPRR1B	ENSG00000169469	protein_coding	1.75575343	1.95353941	0.64078651	3.04865877	0.00229865	0.00372289
HTRA4	ENSG00000169495	protein_coding	83.025609	4.838843	0.21653432	22.3467721	1.298E-110	2.833E-108
GPR183	ENSG00000169508	protein_coding	864.34685	1.45070122	0.13532318	10.7202716	8.1763E-27	6.9153E-26
ZNF280A	ENSG00000169548	protein_coding	1.75730869	2.5723312	0.52021649	4.94473215	7.6249E-07	1.7646E-06
CT55	ENSG00000169551	protein_coding	1.74393825	1.56687535	0.26393412	5.93661533	2.9097E-09	8.2682E-09
CLIC3	ENSG00000169583	protein_coding	105.099599	1.62107373	0.16365778	9.90526543	3.9492E-23	2.7232E-22
INO80E	ENSG00000169592	protein_coding	2613.38294	1.21271968	0.07116291	17.0414573	4.0452E-65	1.6295E-63
GKN1	ENSG00000169605	protein_coding	0.99808416	1.97990766	0.47374786	4.17924352	2.9248E-05	5.8205E-05
CKAP2L	ENSG00000169607	protein_coding	167.950609	2.24524936	0.14263153	15.7416055	7.8428E-56	2.2914E-54
PROKR1	ENSG00000169618	protein_coding	4.66460281	1.06069202	0.19411662	5.46420004	4.65E-08	1.1947E-07
BUB1	ENSG00000169679	protein_coding	337.527436	2.39789535	0.14602216	16.4214482	1.3432E-60	4.6507E-59
CHRNA5	ENSG00000169684	protein_coding	36.105232	1.12112557	0.15769484	7.10946275	1.165E-12	4.2695E-12
GP9	ENSG00000169704	protein_coding	7.37589696	2.31827372	0.22226629	10.4301633	1.8058E-25	1.4201E-24
NLGN1	ENSG00000169760	protein_coding	1899.86342	2.01060012	0.13634986	14.7458908	3.2698E-49	7.502E-48
LINGO1	ENSG00000169783	protein_coding	1043.66485	1.944217	0.13368658	14.5430975	6.4602E-48	1.4022E-46
P2RY1	ENSG00000169860	protein_coding	845.958762	2.41913039	0.11793816	20.5118552	1.6873E-93	1.9833E-91
MUC17	ENSG00000169876	protein_coding	24.8678348	6.61952695	0.46900647	14.1139353	3.1169E-45	6.0517E-44
AHSP	ENSG00000169877	protein_coding	9.01682214	2.08944793	0.3215703	6.49763974	8.159E-11	2.6166E-10
WNT10B	ENSG00000169884	protein_coding	22.4251164	2.6212014	0.21631229	12.1176718	8.5144E-34	1.013E-32

CALML6	ENSG00000169885	protein_coding	8.42457136	2.11484589	0.24899697	8.49346018	2.0057E-17	1.0057E-16
MUC3A	ENSG00000169894	protein_coding	1250.64362	3.41486166	0.18092377	18.8745879	1.8456E-79	1.2552E-77
ITGAM	ENSG00000169896	protein_coding	1438.61667	1.99039765	0.12546047	15.8647387	1.1118E-56	3.3398E-55
PYDC1	ENSG00000169900	protein_coding	5.88481687	1.86145319	0.34775673	5.35274527	8.663E-08	2.1777E-07
S100G	ENSG00000169906	protein_coding	8.46374011	5.54997017	0.61465101	9.02946556	1.7251E-19	9.7825E-19
KCNAB3	ENSG00000170049	protein_coding	90.07288	1.39207744	0.15664902	8.88660181	6.3006E-19	3.4627E-18
SERPINA9	ENSG00000170054	protein_coding	4.40330008	3.51956546	0.41529355	8.47488583	2.3531E-17	1.1757E-16
FAM153A	ENSG00000170074	protein_coding	263.097152	3.24336365	0.25183829	12.8787548	5.9284E-38	8.5113E-37
SPDYE5	ENSG00000170092	protein_coding	35.3293186	1.63506599	0.15226932	10.737987	6.75E-27	5.7365E-26
FOXD4	ENSG00000170122	protein_coding	40.7154418	1.11783785	0.13981283	7.99524535	1.2932E-15	5.789E-15
GPR25	ENSG00000170128	protein_coding	4.52268009	2.15156904	0.23877732	9.01077653	2.046E-19	1.1554E-18
CCDC144A	ENSG00000170160	protein_coding	63.5264531	1.79077349	0.28110715	6.37043017	1.885E-10	5.875E-10
HOXD12	ENSG00000170178	protein_coding	1.03780615	1.70141281	0.36607254	4.64774777	3.3558E-06	7.3121E-06
ANKK1	ENSG00000170209	protein_coding	39.1900981	2.18302082	0.16211751	13.4656698	2.4905E-41	4.1097E-40
ADRA1B	ENSG00000170214	protein_coding	535.130808	1.39822856	0.12496447	11.1890089	4.6156E-29	4.3816E-28
FABP6	ENSG00000170231	protein_coding	1240.27604	6.42376638	0.22043418	29.1414268	1.073E-186	1.967E-183
HSPB2	ENSG00000170276	protein_coding	317.423467	1.27239552	0.13341407	9.53719108	1.4675E-21	9.3331E-21
C7orf33	ENSG00000170279	protein_coding	1.1457065	2.79173417	0.44280937	6.30459604	2.8895E-10	8.8885E-10
SLN	ENSG00000170290	protein_coding	50.1954092	3.11388706	0.37829244	8.23142829	1.8499E-16	8.7174E-16
LGALS9B	ENSG00000170298	protein_coding	3.14593369	2.08093502	0.23286749	8.93613381	4.0302E-19	2.2356E-18
CST2	ENSG00000170369	protein_coding	13.4581774	2.43396804	0.30750749	7.91515051	2.4695E-15	1.0845E-14
CST1	ENSG00000170373	protein_coding	8.42654011	2.55831888	0.38285782	6.68216434	2.3544E-11	7.8611E-11
SP7	ENSG00000170374	protein_coding	2.84638669	2.04560276	0.31489196	6.49620513	8.2371E-11	2.6409E-10
TCAF2	ENSG00000170379	protein_coding	678.065447	1.66334505	0.09169438	18.1400977	1.5376E-73	8.4615E-72
KRT78	ENSG00000170423	protein_coding	3.58629678	3.19882475	0.41170726	7.76965836	7.8698E-15	3.3388E-14

SDR9C7	ENSG00000170426	protein_coding	1.70470885	1.89754401	0.32518806	5.8352204	5.372E-09	1.4927E-08
KRT86	ENSG00000170442	protein_coding	88.5620453	1.5161163	0.15903487	9.5332318	1.5246E-21	9.6881E-21
KRT75	ENSG00000170454	protein_coding	1.67340052	2.83260438	0.55233442	5.12842271	2.9218E-07	7.0202E-07
CD14	ENSG00000170458	protein_coding	6377.40845	1.74406345	0.1223919	14.2498276	4.4939E-46	9.0234E-45
KRT6C	ENSG00000170465	protein_coding	1.88889497	1.9082844	0.64047534	2.97948145	0.00288737	0.00462975
MZB1	ENSG00000170476	protein_coding	426.997078	1.58334066	0.25760218	6.14645677	7.9233E-10	2.3599E-09
KRT4	ENSG00000170477	protein_coding	8.02836464	2.37046483	0.40543983	5.84665018	5.0157E-09	1.3979E-08
KRT74	ENSG00000170484	protein_coding	0.49074689	1.28473595	0.51173063	2.51057074	0.01205362	0.01782764
KRT72	ENSG00000170486	protein_coding	5.4235044	3.87157403	0.33311328	11.6223948	3.1712E-31	3.3334E-30
KISS1	ENSG00000170498	protein_coding	16.2260525	1.21882561	0.25447549	4.78955989	1.6715E-06	3.7431E-06
HSD17B13	ENSG00000170509	protein_coding	58.7335627	1.30747513	0.21652229	6.03852442	1.5553E-09	4.5199E-09
SERPINB9	ENSG00000170542	protein_coding	1954.27165	1.34651939	0.08458224	15.9196467	4.6298E-57	1.4121E-55
CDH2	ENSG00000170558	protein_coding	5615.10887	1.20492434	0.1272031	9.47244514	2.7337E-21	1.7134E-20
EMB	ENSG00000170571	protein_coding	1339.49994	1.55533496	0.12030279	12.9285028	3.1081E-38	4.5131E-37
GTSF1	ENSG00000170627	protein_coding	28.6044807	1.18527658	0.18284272	6.48249272	9.0219E-11	2.8833E-10
CAVIN4	ENSG00000170681	protein_coding	25.4492297	1.05051454	0.13915638	7.54916537	4.3806E-14	1.7691E-13
ZNF296	ENSG00000170684	protein_coding	89.8567145	1.56727645	0.13283072	11.799051	3.9474E-32	4.3311E-31
TTLL6	ENSG00000170703	protein_coding	287.712072	1.00479182	0.20664139	4.86249066	1.1592E-06	2.6355E-06
GPR37	ENSG00000170775	protein_coding	273.868728	1.70889014	0.23908046	7.14776156	8.8204E-13	3.2589E-12
OR10A4	ENSG00000170782	protein_coding	0.48950937	1.20662653	0.53143458	2.27050812	0.02317677	0.03293999
OR10A2	ENSG00000170790	protein_coding	1.05060609	2.06500831	0.43737681	4.72134837	2.3429E-06	5.1751E-06
HTRA3	ENSG00000170801	protein_coding	581.178473	1.07936479	0.15934829	6.77362	1.256E-11	4.2715E-11
LMOD2	ENSG00000170807	protein_coding	2.33655992	1.30180798	0.24985515	5.21025063	1.8859E-07	4.6077E-07
PSG6	ENSG00000170848	protein_coding	1.47140752	3.09449432	0.52585226	5.88472187	3.9872E-09	1.1196E-08
PLA2G1B	ENSG00000170890	protein_coding	22.9567424	1.15126842	0.19351157	5.94935179	2.6921E-09	7.6703E-09

CYTL1	ENSG00000170891	protein_coding	103.820618	1.6290709	0.15831891	10.2898061	7.8334E-25	5.9275E-24
OSCAR	ENSG00000170909	protein_coding	278.239036	2.57112399	0.12429068	20.6863784	4.5945E-95	5.7233E-93
TEX13B	ENSG00000170925	protein_coding	1.13743986	2.61221548	0.39381908	6.63303432	3.2886E-11	1.0872E-10
MBD3L1	ENSG00000170948	protein_coding	0.52102336	1.39180084	0.4838982	2.87622654	0.00402461	0.0063432
PGK2	ENSG00000170950	protein_coding	0.59605149	1.03860009	0.36461558	2.84847973	0.00439287	0.00688998
CAVIN3	ENSG00000170955	protein_coding	2837.47906	2.29532277	0.12409787	18.4960694	2.2209E-76	1.3721E-74
CEACAM3	ENSG00000170956	protein_coding	13.9122726	1.64940774	0.17033177	9.68350048	3.5437E-22	2.3273E-21
PDGFD	ENSG00000170962	protein_coding	5274.16896	1.83247575	0.12605239	14.5374143	7.0194E-48	1.52E-46
PLAC1	ENSG00000170965	protein_coding	3.77470618	1.3695466	0.25316713	5.40965416	6.3147E-08	1.6061E-07
DDI1	ENSG00000170967	protein_coding	1.53321541	2.34204247	0.34897838	6.71113918	1.9311E-11	6.4819E-11
FPR2	ENSG00000171049	protein_coding	74.546852	1.17614251	0.16811363	6.99611631	2.6316E-12	9.4031E-12
FPR1	ENSG00000171051	protein_coding	755.020642	1.55010576	0.15450179	10.0329307	1.0923E-23	7.7664E-23
C8orf74	ENSG00000171060	protein_coding	2.03011793	2.91205848	0.42043202	6.92634799	4.3184E-12	1.518E-11
ALK	ENSG00000171094	protein_coding	23.2652358	2.26088853	0.19381115	11.6654202	1.9146E-31	2.034E-30
OBP2B	ENSG00000171102	protein_coding	2.73908429	3.36698463	0.30463438	11.0525432	2.1309E-28	1.9494E-27
INSR	ENSG00000171105	protein_coding	21020.9174	1.25228538	0.09310188	13.4506996	3.0497E-41	5.0091E-40
RLN3	ENSG00000171136	protein_coding	1.09618	1.29257034	0.36548738	3.53656623	0.00040536	0.00071449
ZNF692	ENSG00000171163	protein_coding	1108.12332	1.81148685	0.12489935	14.5035734	1.15E-47	2.4704E-46
GRIK1	ENSG00000171189	protein_coding	25.5940566	1.15236481	0.18025245	6.39306055	1.626E-10	5.0955E-10
NETO2	ENSG00000171208	protein_coding	3886.80023	3.29044621	0.11733379	28.0434669	4.799E-173	6.285E-170
CSN3	ENSG00000171209	protein_coding	0.66173568	1.87258429	0.65029594	2.87958786	0.00398195	0.00627878
LRG1	ENSG00000171236	protein_coding	969.190761	1.21256744	0.21353099	5.6786486	1.3576E-08	3.6482E-08
KCNK3	ENSG00000171303	protein_coding	4940.27846	2.15498022	0.20087428	10.7280045	7.5203E-27	6.3722E-26
CHST11	ENSG00000171310	protein_coding	2469.85325	1.77284321	0.09954355	17.809725	5.9401E-71	2.9792E-69
ESCO2	ENSG00000171320	protein_coding	88.6569622	1.88660807	0.12858499	14.6720702	9.733E-49	2.1903E-47

KRT15	ENSG00000171346	protein_coding	53.7323921	1.95333905	0.30082407	6.49329363	8.398E-11	2.6906E-10
APLN	ENSG00000171388	protein_coding	5653.5507	2.44741373	0.13279142	18.4305115	7.4773E-76	4.4923E-74
KRT9	ENSG00000171403	protein_coding	0.65322842	1.41080456	0.46746565	3.01798549	0.00254461	0.00410225
ZNF581	ENSG00000171425	protein_coding	1133.26203	1.06665889	0.08456212	12.6139094	1.7699E-36	2.3919E-35
KRT20	ENSG00000171431	protein_coding	10.3959917	2.68146368	0.39309566	6.82140241	9.0156E-12	3.0988E-11
CDK5R2	ENSG00000171450	protein_coding	9.99187048	2.46526741	0.33010121	7.46821681	8.1289E-14	3.2263E-13
DLK2	ENSG00000171462	protein_coding	118.307892	2.33216179	0.1601961	14.5581681	5.1828E-48	1.1303E-46
NLRP5	ENSG00000171487	protein_coding	1.80547345	2.45778092	0.46922151	5.23799711	1.6233E-07	3.9859E-07
SPACA5	ENSG00000171489	protein_coding	0.57164044	1.0483467	0.30710186	3.41367746	0.00064092	0.00110499
OR1N2	ENSG00000171501	protein_coding	0.85570811	1.08123053	0.50739093	2.1309615	0.03309231	0.04595949
OR1N1	ENSG00000171505	protein_coding	1.43109745	1.84386744	0.4688015	3.93315179	8.3839E-05	0.00015913
NEUROD2	ENSG00000171532	protein_coding	4.9801448	1.51334067	0.2471968	6.12200749	9.2404E-10	2.7362E-09
OTP	ENSG00000171540	protein_coding	2.90933196	2.30432993	0.35072383	6.57021199	5.0244E-11	1.6369E-10
FGG	ENSG00000171557	protein_coding	3893.87347	6.19622009	0.42132486	14.7065142	5.8546E-49	1.328E-47
FGA	ENSG00000171560	protein_coding	1965.1206	3.30579436	0.36974195	8.94081507	3.8631E-19	2.1452E-18
OR2AT4	ENSG00000171561	protein_coding	1.66611693	3.15784283	0.50522615	6.25035509	4.0952E-10	1.2473E-09
FGB	ENSG00000171564	protein_coding	7863.56776	3.05770486	0.40155458	7.61466811	2.6437E-14	1.0822E-13
DSCAM	ENSG00000171587	protein_coding	23.8615277	1.90805461	0.25943678	7.35460346	1.9149E-13	7.4047E-13
DNAI2	ENSG00000171595	protein_coding	8.36664456	1.34603294	0.17441775	7.71729331	1.1883E-14	4.9832E-14
NMUR1	ENSG00000171596	protein_coding	214.720711	1.04564022	0.12257984	8.53027918	1.4599E-17	7.3853E-17
PTCRA	ENSG00000171611	protein_coding	14.1372404	2.93661047	0.17886524	16.4180052	1.4216E-60	4.9147E-59
SPSB1	ENSG00000171621	protein_coding	2684.08847	1.29080963	0.13456348	9.59257022	8.5918E-22	5.5317E-21
S100Z	ENSG00000171643	protein_coding	18.7544438	2.26057039	0.14575209	15.5096944	2.983E-54	8.2474E-53
GPR82	ENSG00000171657	protein_coding	90.3860323	2.04633889	0.15561667	13.1498693	1.7047E-39	2.6118E-38
GPR34	ENSG00000171659	protein_coding	915.353941	1.61312236	0.14179314	11.3765899	5.4697E-30	5.4108E-29

LKAAEAR1	ENSG00000171695	protein_coding	2.60027244	1.61567126	0.30675142	5.26703755	1.3864E-07	3.4227E-07
RGS19	ENSG00000171700	protein_coding	868.308903	1.71498896	0.08247787	20.7933224	4.9745E-96	6.4783E-94
LGALS4	ENSG00000171747	protein_coding	2113.56609	3.4385097	0.33001436	10.419273	2.025E-25	1.5876E-24
RASGRP4	ENSG00000171777	protein_coding	200.783368	1.72841351	0.09853651	17.5408428	6.9883E-69	3.2261E-67
NHLH1	ENSG00000171786	protein_coding	13.589344	2.0658053	0.15646321	13.2031375	8.4156E-40	1.2985E-38
SLFNL1	ENSG00000171790	protein_coding	42.0256893	2.19682558	0.15283049	14.3742627	7.5065E-47	1.5585E-45
UTF1	ENSG00000171794	protein_coding	1.18817225	1.44534666	0.29704964	4.86567378	1.1407E-06	2.5953E-06
KNDC1	ENSG00000171798	protein_coding	470.732969	1.65283858	0.21528962	7.67727936	1.625E-14	6.7482E-14
NINJ2	ENSG00000171840	protein_coding	136.304692	1.64738043	0.12166773	13.5399947	9.0795E-42	1.5196E-40
RRM2	ENSG00000171848	protein_coding	757.907554	2.15401792	0.15321156	14.0591086	6.7734E-45	1.2948E-43
IFNB1	ENSG00000171855	protein_coding	0.71682947	1.49117133	0.34634364	4.30546764	1.6663E-05	3.3933E-05
C3AR1	ENSG00000171860	protein_coding	1408.21918	1.8738176	0.12200931	15.3579882	3.1314E-53	8.4143E-52
PRND	ENSG00000171864	protein_coding	338.902251	3.26610966	0.26462314	12.3424944	5.3473E-35	6.7213E-34
KLF17	ENSG00000171872	protein_coding	2.88844658	1.78724946	0.39477083	4.52730886	5.974E-06	1.2708E-05
LGALS9C	ENSG00000171916	protein_coding	5.35511771	1.76916607	0.18181909	9.73036489	2.2379E-22	1.4818E-21
SCG2	ENSG00000171951	protein_coding	433.871663	3.89482667	0.23357024	16.6751836	1.986E-62	7.2427E-61
CYP4F22	ENSG00000171954	protein_coding	19.104482	1.50940504	0.15369056	9.82106561	9.1373E-23	6.1861E-22
FOXB1	ENSG00000171956	protein_coding	5.47086347	2.20156771	0.33645625	6.54339972	6.0136E-11	1.9464E-10
SYNPO	ENSG00000171992	protein_coding	26629.1883	1.10911928	0.11549214	9.60341822	7.7336E-22	4.9946E-21
ANKRD20A4P	ENSG00000172014	protein_coding	7.15244492	1.18650702	0.23728853	5.0002712	5.725E-07	1.3401E-06
REG3A	ENSG00000172016	protein_coding	2.39083833	3.88300128	0.5195349	7.47399507	7.7796E-14	3.0925E-13
GAP43	ENSG00000172020	protein_coding	27.885309	1.44185619	0.22961832	6.27936039	3.3997E-10	1.0413E-09
REG1B	ENSG00000172023	protein_coding	60.7086751	4.08073173	0.39441096	10.3463953	4.3454E-25	3.3361E-24
LRRC15	ENSG00000172061	protein_coding	108.504231	1.25279942	0.28449485	4.40359257	1.0647E-05	2.2109E-05
TEX37	ENSG00000172073	protein_coding	0.62585848	1.89030469	0.58928365	3.20780099	0.00133754	0.00222693

MOB3A	ENSG00000172081	protein_coding	4415.20723	1.07798691	0.06383244	16.8877608	5.5365E-64	2.1363E-62
CD8B	ENSG00000172116	protein_coding	401.315688	3.39853474	0.19672985	17.2751354	7.2403E-67	3.1048E-65
CALB2	ENSG00000172137	protein_coding	31.4554804	2.29995741	0.21380178	10.7574287	5.4674E-27	4.6664E-26
ISG20	ENSG00000172183	protein_coding	828.634756	2.35202066	0.11649322	20.1901943	1.1939E-90	1.2272E-88
CXCR6	ENSG00000172215	protein_coding	399.686369	2.69809348	0.15255344	17.6862191	5.355E-70	2.584E-68
CEBPB	ENSG00000172216	protein_coding	2626.42464	1.39156786	0.13344102	10.4283364	1.8408E-25	1.4467E-24
AZU1	ENSG00000172232	protein_coding	8.44798138	2.38378805	0.23560574	10.1176993	4.6113E-24	3.3468E-23
TPSAB1	ENSG00000172236	protein_coding	409.076145	1.65385597	0.17730589	9.32769894	1.0819E-20	6.5588E-20
CLEC7A	ENSG00000172243	protein_coding	829.423737	2.33370488	0.12769873	18.275083	1.3069E-74	7.4888E-73
OR10V1	ENSG00000172289	protein_coding	0.42458953	1.02781216	0.48497489	2.11931006	0.03406427	0.04721924
B3GALT1	ENSG00000172318	protein_coding	23.8100863	1.28974736	0.18663421	6.91056262	4.8274E-12	1.6907E-11
OR5A1	ENSG00000172320	protein_coding	0.68138635	2.00309048	0.55499182	3.60922523	0.00030711	0.00054818
CLEC12A	ENSG00000172322	protein_coding	234.527629	2.79336948	0.15413761	18.1225687	2.1149E-73	1.1569E-71
IL16	ENSG00000172349	protein_coding	1382.29443	1.84782345	0.10260539	18.0090296	1.655E-72	8.7206E-71
OR9I1	ENSG00000172377	protein_coding	0.68257441	1.78519466	0.33788419	5.28345125	1.2677E-07	3.1406E-07
PRSS27	ENSG00000172382	protein_coding	89.394581	1.16494433	0.11934548	9.76110989	1.6534E-22	1.1034E-21
INSL5	ENSG00000172410	protein_coding	1.90902344	1.52574948	0.3059489	4.98694228	6.1342E-07	1.4318E-06
EFCAB3	ENSG00000172421	protein_coding	4.40673244	3.6514844	0.31107167	11.7384022	8.1002E-32	8.7638E-31
RSPH9	ENSG00000172426	protein_coding	141.773708	1.15456768	0.10670838	10.8198408	2.7726E-27	2.404E-26
CTSW	ENSG00000172543	protein_coding	803.306152	3.0156394	0.15811999	19.0718417	4.3279E-81	3.1442E-79
NIPAL4	ENSG00000172548	protein_coding	27.6582977	2.19991382	0.27789461	7.91636023	2.4456E-15	1.0743E-14
KLHL6	ENSG00000172578	protein_coding	746.498341	2.59874541	0.12561773	20.6877285	4.4677E-95	5.5805E-93
SMPDL3A	ENSG00000172594	protein_coding	5778.59733	1.79107447	0.11242953	15.9306402	3.8836E-57	1.1908E-55
AGAP5	ENSG00000172650	protein_coding	17.6828367	1.72956926	0.14963697	11.5584352	6.6917E-31	6.8996E-30
THEMIS	ENSG00000172673	protein_coding	256.117785	2.20954868	0.16798884	13.1529494	1.6366E-39	2.5126E-38

SLFN11	ENSG00000172716	protein_coding	3174.03462	1.68887438	0.09747786	17.325722	3.009E-67	1.3062E-65
TMCC1	ENSG00000172765	protein_coding	5625.85293	1.77652638	0.08384767	21.1875474	1.244E-99	1.8279E-97
OR10W1	ENSG00000172772	protein_coding	0.67733423	2.02560504	0.51507772	3.93262019	8.4025E-05	0.00015947
RAB37	ENSG00000172794	protein_coding	731.986582	1.88860252	0.10490092	18.0036797	1.8229E-72	9.5832E-71
RPL38	ENSG00000172809	protein_coding	17933.0249	1.01510102	0.08361903	12.139594	6.5148E-34	7.7915E-33
CES4A	ENSG00000172824	protein_coding	1766.0721	4.13631056	0.19145145	21.6050109	1.612E-103	2.777E-101
CES3	ENSG00000172828	protein_coding	3677.73077	1.72464768	0.20246204	8.51837533	1.6181E-17	8.1555E-17
EGFL7	ENSG00000172889	protein_coding	3881.82333	1.15102894	0.12685187	9.07380383	1.1493E-19	6.5825E-19
NADSYN1	ENSG00000172890	protein_coding	3728.94126	1.05296433	0.07213019	14.5981078	2.8875E-48	6.3698E-47
MYEOV	ENSG00000172927	protein_coding	1438.58477	7.09691264	0.27644445	25.6721114	2.395E-145	1.39E-142
XKR3	ENSG00000172967	protein_coding	5.11454401	2.08033538	0.2998757	6.93732558	3.9959E-12	1.4082E-11
SH3RF3	ENSG00000172985	protein_coding	968.903905	1.14436349	0.10042262	11.395475	4.4042E-30	4.3738E-29
GXYLT2	ENSG00000172986	protein_coding	502.204718	1.95090913	0.19493076	10.0082159	1.4026E-23	9.9131E-23
ARPP21	ENSG00000172995	protein_coding	92.4005917	1.19131425	0.29929883	3.98035048	6.8814E-05	0.00013162
HSPA6	ENSG00000173110	protein_coding	307.752825	1.90368184	0.12164449	15.649552	3.3457E-55	9.5736E-54
ADAMTS20	ENSG00000173157	protein_coding	15.8432832	5.46881207	0.38247919	14.2983257	2.2414E-46	4.5706E-45
CYSLTR1	ENSG00000173198	protein_coding	320.935557	1.12766615	0.10906295	10.3395897	4.6653E-25	3.5763E-24
PARP15	ENSG00000173200	protein_coding	175.159804	2.66412853	0.1525137	17.4681263	2.5059E-68	1.1329E-66
ABLIM3	ENSG00000173210	protein_coding	6849.21198	1.95845819	0.13641464	14.356657	9.6784E-47	2.0003E-45
SYT12	ENSG00000173227	protein_coding	359.429325	1.97477445	0.22025858	8.96570947	3.0829E-19	1.7226E-18
C11orf86	ENSG00000173237	protein_coding	65.5435721	2.48161489	0.3012834	8.23681242	1.7686E-16	8.3461E-16
GPR151	ENSG00000173250	protein_coding	1.12088806	1.50017543	0.40657042	3.68982923	0.0002244	0.00040681
PLAC8L1	ENSG00000173261	protein_coding	18.4470829	2.63858922	0.17491694	15.0848125	2.0385E-51	5.1065E-50
SLC2A14	ENSG00000173262	protein_coding	44.4720182	2.38147867	0.20863501	11.4145685	3.5365E-30	3.5282E-29
MZT2A	ENSG00000173272	protein_coding	2285.58251	1.60430426	0.12608868	12.7236184	4.3714E-37	6.0595E-36

PPP1R3B	ENSG00000173281	protein_coding	6836.5904	1.31112492	0.09569421	13.7011935	9.9869E-43	1.752E-41
KCNK7	ENSG00000173338	protein_coding	23.366718	1.68316414	0.13570834	12.4028054	2.5233E-35	3.2329E-34
C1QB	ENSG00000173369	protein_coding	14861.518	2.92212192	0.14093284	20.7341443	1.7046E-95	2.1408E-93
C1QA	ENSG00000173372	protein_coding	12613.6101	2.60715327	0.13809144	18.8799054	1.6688E-79	1.1435E-77
IHO1	ENSG00000173421	protein_coding	34.4503659	1.33434873	0.14713504	9.06887126	1.2026E-19	6.8779E-19
SAA1	ENSG00000173432	protein_coding	1673.04256	4.30165489	0.37461477	11.4828759	1.6084E-30	1.6337E-29
EHBP1L1	ENSG00000173442	protein_coding	3439.61413	1.42606624	0.07817836	18.2411879	2.4312E-74	1.3759E-72
TMEM196	ENSG00000173452	protein_coding	15.8179297	1.90854381	0.38159632	5.00147334	5.6894E-07	1.332E-06
CSPG4	ENSG00000173546	protein_coding	8298.98323	2.71959893	0.12553927	21.6633331	4.551E-104	8.024E-102
SNX33	ENSG00000173548	protein_coding	5954.09356	1.304942	0.06795612	19.2027142	3.5125E-82	2.631E-80
NABP1	ENSG00000173559	protein_coding	1314.11187	1.14854816	0.11827552	9.71078507	2.7124E-22	1.7898E-21
NLRP13	ENSG00000173572	protein_coding	1.08597971	2.70662734	0.48304818	5.60322433	2.104E-08	5.5616E-08
XCR1	ENSG00000173578	protein_coding	154.382575	1.30353989	0.20007178	6.51536105	7.2515E-11	2.3347E-10
SPDYE2B	ENSG00000173678	protein_coding	2.07761635	1.89759056	0.34277202	5.53601353	3.0943E-08	8.0664E-08
ADGRG2	ENSG00000173698	protein_coding	482.373659	2.53593196	0.23547397	10.7694788	4.7971E-27	4.1073E-26
SPATA3	ENSG00000173699	protein_coding	1.08624791	2.12486852	0.47735669	4.45132239	8.5343E-06	1.7875E-05
CD7	ENSG00000173762	protein_coding	458.256905	2.73309085	0.16149481	16.9237066	3.0088E-64	1.1749E-62
C1QTNF1	ENSG00000173918	protein_coding	3566.09651	1.17768088	0.1492606	7.89009854	3.0195E-15	1.3172E-14
MARCHF3	ENSG00000173926	protein_coding	235.703889	1.43353573	0.09262247	15.4771916	4.946E-54	1.3609E-52
C9orf131	ENSG00000174038	protein_coding	5.99472197	1.79910876	0.22433886	8.0196038	1.0609E-15	4.7692E-15
TLR10	ENSG00000174123	protein_coding	114.2977	1.81825014	0.16487047	11.0283556	2.7892E-28	2.5389E-27
TLR6	ENSG00000174130	protein_coding	237.596934	1.72391709	0.11875837	14.5161734	9.5706E-48	2.0597E-46
NWD2	ENSG00000174145	protein_coding	2.15740661	1.37781382	0.307837	4.47579022	7.6129E-06	1.6027E-05
ZNF80	ENSG00000174255	protein_coding	12.4542182	2.59629507	0.21484179	12.0846839	1.2726E-33	1.5024E-32
EVX2	ENSG00000174279	protein_coding	2.10088953	1.23248209	0.28117744	4.38328936	1.169E-05	2.4169E-05

PHLDA3	ENSG00000174307	protein_coding	3204.40781	1.64910063	0.11674546	14.1256086	2.6411E-45	5.1388E-44
GLIS1	ENSG00000174332	protein_coding	708.999361	1.85685039	0.158482	11.716475	1.0495E-31	1.1299E-30
CHRNA9	ENSG00000174343	protein_coding	1.64891858	1.59726021	0.36967527	4.32071157	1.5553E-05	3.1757E-05
EXO1	ENSG00000174371	protein_coding	113.81895	1.68029395	0.13246816	12.6845121	7.2061E-37	9.8844E-36
TRHR	ENSG00000174417	protein_coding	1.12822317	1.62144384	0.35602494	4.55429846	5.2561E-06	1.1238E-05
GOLGA6L2	ENSG00000174450	protein_coding	19.3042939	6.33191806	0.52419685	12.0792752	1.3591E-33	1.6012E-32
VWC2L	ENSG00000174453	protein_coding	9.54416282	1.21757382	0.18788441	6.48044079	9.1455E-11	2.9213E-10
ANKRD36C	ENSG00000174501	protein_coding	148.043121	1.69185466	0.16048596	10.5420727	5.5267E-26	4.473E-25
MYO1H	ENSG00000174527	protein_coding	33.9717168	1.434923	0.17336354	8.27695961	1.2636E-16	6.0288E-16
IL20RB	ENSG00000174564	protein_coding	1387.42894	6.58176797	0.29476122	22.3291517	1.925E-110	4.183E-108
BRSK2	ENSG00000174672	protein_coding	57.0966895	1.23575154	0.18644815	6.62785613	3.406E-11	1.1246E-10
SH3PXD2B	ENSG00000174705	protein_coding	1994.09487	1.5267387	0.12136677	12.579545	2.736E-36	3.6609E-35
RIN1	ENSG00000174791	protein_coding	556.300333	2.04186332	0.13189841	15.4805758	4.6925E-54	1.292E-52
ODAPH	ENSG00000174792	protein_coding	8.90552019	2.08747344	0.26436207	7.8962668	2.8738E-15	1.2561E-14
CD248	ENSG00000174807	protein_coding	4080.55952	1.77082167	0.13667149	12.9567744	2.151E-38	3.1333E-37
ADGRE1	ENSG00000174837	protein_coding	220.116027	2.72750296	0.16075611	16.9667138	1.4481E-64	5.7227E-63
CNIH2	ENSG00000174871	protein_coding	31.1308534	1.95228907	0.14243556	13.706472	9.2865E-43	1.6329E-41
NLRP6	ENSG00000174885	protein_coding	287.345313	1.36558823	0.18078373	7.55371206	4.2302E-14	1.7097E-13
CATSPERD	ENSG00000174898	protein_coding	3.63178547	3.35228009	0.40447659	8.28794593	1.1522E-16	5.5117E-16
SEZ6L2	ENSG00000174938	protein_coding	4449.09767	2.27545999	0.15052233	15.1170925	1.2494E-51	3.1538E-50
ASPHD1	ENSG00000174939	protein_coding	696.564954	2.64852564	0.15625776	16.9497228	1.9336E-64	7.6086E-63
KCTD13	ENSG00000174943	protein_coding	788.599079	1.0227839	0.07278248	14.0526115	7.4245E-45	1.4157E-43
P2RY14	ENSG00000174944	protein_coding	312.670256	1.09513317	0.13186538	8.30493323	9.9876E-17	4.7936E-16
AMZ1	ENSG00000174945	protein_coding	122.180023	3.78731739	0.2142055	17.6807663	5.8989E-70	2.8375E-68
GPR171	ENSG00000174946	protein_coding	127.721118	1.67318283	0.15050661	11.1170057	1.0369E-28	9.6673E-28

ZIC4	ENSG00000174963	protein_coding	6.31945006	2.19399908	0.37534744	5.84524859	5.0581E-09	1.409E-08
UBE2C	ENSG00000175063	protein_coding	410.439934	2.9372885	0.18897843	15.5429832	1.7752E-54	4.9531E-53
RTP1	ENSG00000175077	protein_coding	1.09330399	1.08058489	0.35292914	3.06176161	0.00220039	0.0035713
NPPA	ENSG00000175206	protein_coding	8.67311926	2.01861906	0.19890469	10.1486753	3.3589E-24	2.4558E-23
C1orf127	ENSG00000175262	protein_coding	29.5466434	3.14004741	0.15575276	20.1604609	2.1784E-90	2.2093E-88
CHST1	ENSG00000175264	protein_coding	740.621386	1.45557021	0.17866278	8.14702522	3.7299E-16	1.7239E-15
GOLGA8A	ENSG00000175265	protein_coding	1307.91917	2.83232477	0.20348855	13.9188408	4.8673E-44	9.0224E-43
VWA3A	ENSG00000175267	protein_coding	29.9187159	1.07813742	0.22018601	4.89648469	9.7566E-07	2.234E-06
TP53I11	ENSG00000175274	protein_coding	7677.20908	1.07881153	0.10194169	10.5826335	3.5873E-26	2.9261E-25
CATSPER1	ENSG00000175294	protein_coding	35.7360509	2.00014913	0.14041941	14.2441076	4.8774E-46	9.7806E-45
CCNE2	ENSG00000175305	protein_coding	101.061539	1.55288803	0.08922578	17.4040283	7.69E-68	3.4093E-66
PHYKPL	ENSG00000175309	protein_coding	4172.8045	1.6190327	0.08572635	18.8860568	1.4854E-79	1.0208E-77
PROP1	ENSG00000175325	protein_coding	1.73764912	2.84059839	0.45071658	6.30240497	2.9306E-10	9.0139E-10
ARL10	ENSG00000175414	protein_coding	1343.29471	1.31056329	0.10792156	12.143665	6.1986E-34	7.421E-33
CCDC14	ENSG00000175455	protein_coding	1932.22717	1.13567982	0.09663487	11.7522782	6.874E-32	7.4547E-31
TBC1D10C	ENSG00000175463	protein_coding	608.676293	2.53041431	0.13764461	18.383679	1.7751E-75	1.05E-73
LRRC25	ENSG00000175489	protein_coding	1033.38163	2.29112977	0.14361002	15.9538292	2.6795E-57	8.2938E-56
TSGA10IP	ENSG00000175513	protein_coding	6.39328272	2.13063218	0.21870798	9.74190424	1.9978E-22	1.3259E-21
KCNE3	ENSG00000175538	protein_coding	4791.64925	2.11388797	0.11117054	19.0148217	1.2857E-80	9.1521E-79
CCDC85B	ENSG00000175602	protein_coding	1094.76048	1.31701881	0.13075503	10.0724142	7.316E-24	5.2476E-23
CCDC197	ENSG00000175699	protein_coding	1.74817156	2.70603441	0.46613171	5.8053	6.4251E-09	1.7733E-08
B3GNTL1	ENSG00000175711	protein_coding	270.644784	1.33364592	0.09017873	14.7889185	1.7271E-49	3.9985E-48
PRIMA1	ENSG00000175785	protein_coding	2172.8866	3.05403205	0.27252047	11.2066152	3.7839E-29	3.6107E-28
CCDC168	ENSG00000175820	protein_coding	17.6460768	1.6546565	0.17137156	9.65537385	4.6645E-22	3.0447E-21
GAPT	ENSG00000175857	protein_coding	350.030537	2.07529569	0.14738482	14.0807966	4.9845E-45	9.5844E-44

CREG2	ENSG00000175874	protein_coding	179.53104	2.01258555	0.19445717	10.3497624	4.1953E-25	3.2251E-24
ORAI3	ENSG00000175938	protein_coding	1897.45884	1.28286615	0.09155009	14.0127246	1.3031E-44	2.4663E-43
TUBB6	ENSG00000176014	protein_coding	3821.63905	1.15881508	0.08907225	13.0098325	1.0758E-38	1.5882E-37
AMIGO3	ENSG00000176020	protein_coding	1.69171952	1.43413754	0.2589433	5.53842301	3.0521E-08	7.958E-08
NUPR1	ENSG00000176046	protein_coding	9307.93638	1.4621217	0.15860928	9.21838701	3.016E-20	1.7801E-19
ZNF683	ENSG00000176083	protein_coding	149.814708	3.37360419	0.18267213	18.4680838	3.7309E-76	2.2896E-74
CCDC57	ENSG00000176155	protein_coding	1297.48119	1.02837296	0.11067676	9.29167913	1.5187E-20	9.1473E-20
HSF5	ENSG00000176160	protein_coding	6.34840671	1.1186055	0.18574619	6.02222587	1.7203E-09	4.9853E-09
FOXG1	ENSG00000176165	protein_coding	5.4247943	1.74534168	0.29675803	5.88136288	4.069E-09	1.1418E-08
SPHK1	ENSG00000176170	protein_coding	452.249911	1.66683722	0.15781402	10.5620349	4.4687E-26	3.6321E-25
BNIP3	ENSG00000176171	protein_coding	14001.8491	1.52656538	0.09632477	15.8481076	1.4488E-56	4.3323E-55
ENTHD1	ENSG00000176177	protein_coding	5.7798028	2.18996693	0.2736901	8.00162998	1.2278E-15	5.5041E-15
OR11H4	ENSG00000176198	protein_coding	0.42547838	1.36781037	0.4054736	3.37336478	0.00074256	0.00127094
LRRTM4	ENSG00000176204	protein_coding	38.491401	2.44731963	0.29148492	8.39604204	4.6178E-17	2.264E-16
ATAD5	ENSG00000176208	protein_coding	230.044176	1.25438815	0.09721076	12.9037992	4.2844E-38	6.1976E-37
OR4N2	ENSG00000176294	protein_coding	0.75189612	1.8758539	0.5694103	3.29437999	0.00098639	0.00166656
FOXR1	ENSG00000176302	protein_coding	0.94273092	2.0980917	0.36981762	5.67331461	1.4006E-08	3.7602E-08
TAC4	ENSG00000176358	protein_coding	13.415207	1.2715087	0.15359022	8.2785786	1.2465E-16	5.9505E-16
B3GNT4	ENSG00000176383	protein_coding	420.150968	1.75842957	0.1727117	10.1812994	2.4033E-24	1.7749E-23
CLEC14A	ENSG00000176435	protein_coding	4727.56079	1.33521208	0.12033576	11.0957215	1.3159E-28	1.2174E-27
CLK2	ENSG00000176444	protein_coding	2069.07422	1.03634877	0.08632397	12.0053415	3.3309E-33	3.8529E-32
PLAAT3	ENSG00000176485	protein_coding	7185.5911	1.04260874	0.07861545	13.262135	3.8381E-40	6.0213E-39
CNBD1	ENSG00000176571	protein_coding	0.7436933	1.96973148	0.43025922	4.57801105	4.6942E-06	1.009E-05
FOXL1	ENSG00000176678	protein_coding	270.184416	1.06407975	0.14408015	7.38533197	1.5207E-13	5.9249E-13
FOXC2	ENSG00000176692	protein_coding	784.188092	1.23561199	0.14790272	8.35422082	6.5866E-17	3.1981E-16

BDNF	ENSG00000176697	protein_coding	249.195725	2.12100883	0.17918025	11.8372913	2.5041E-32	2.7754E-31
CDK5R1	ENSG00000176749	protein_coding	212.883263	1.06898741	0.09150659	11.6820817	1.5739E-31	1.6799E-30
SOX11	ENSG00000176887	protein_coding	328.218151	5.3936529	0.21869799	24.6625628	2.698E-134	1.167E-131
TYMS	ENSG00000176890	protein_coding	1575.00548	2.04392061	0.09046224	22.5941863	4.943E-113	1.133E-110
C8G	ENSG00000176919	protein_coding	57.6723304	1.5613889	0.19855154	7.86389722	3.7236E-15	1.6144E-14
LY6H	ENSG00000176956	protein_coding	124.606422	3.48137001	0.24397194	14.2695511	3.3875E-46	6.8499E-45
FMR1NB	ENSG00000176988	protein_coding	1.45440698	1.86384432	0.35064709	5.31544219	1.064E-07	2.6525E-07
C19orf18	ENSG00000177025	protein_coding	35.1743166	1.0068661	0.13784491	7.30434012	2.7863E-13	1.0637E-12
SCN4B	ENSG00000177098	protein_coding	2027.9885	1.94749853	0.14462284	13.4660507	2.4777E-41	4.09E-40
DSCAML1	ENSG00000177103	protein_coding	922.11788	1.30286789	0.12714753	10.2468991	1.222E-24	9.1803E-24
RHOG	ENSG00000177105	protein_coding	3038.22828	1.00591703	0.06983023	14.405179	4.8007E-47	1.0044E-45
ZDHHC22	ENSG00000177108	protein_coding	2.54308889	1.77393466	0.25897531	6.84982145	7.3942E-12	2.5584E-11
FAM9B	ENSG00000177138	protein_coding	8.78549604	2.46393136	0.29134664	8.45704399	2.7424E-17	1.3642E-16
OR2T35	ENSG00000177151	protein_coding	0.88239503	1.4499178	0.40705307	3.56198717	0.00036806	0.00065149
RIMKLA	ENSG00000177181	protein_coding	1743.60309	2.32707062	0.13693438	16.9940568	9.088E-65	3.6227E-63
CLVS1	ENSG00000177182	protein_coding	11.0347728	1.19297989	0.16518307	7.22216791	5.1165E-13	1.9181E-12
KCNA3	ENSG00000177272	protein_coding	70.7517822	1.350121	0.15416959	8.75737562	1.9985E-18	1.0658E-17
FBXO39	ENSG00000177294	protein_coding	21.2168306	2.30152957	0.16904546	13.614856	3.2679E-42	5.5814E-41
KCNA2	ENSG00000177301	protein_coding	17.4817518	1.00646305	0.24222536	4.15506889	3.2519E-05	6.4454E-05
BEND2	ENSG00000177324	protein_coding	2.00346738	1.42615237	0.32728143	4.35757194	1.3151E-05	2.7055E-05
LRRN4CL	ENSG00000177363	protein_coding	186.26547	1.60465274	0.25536527	6.28375477	3.3049E-10	1.0127E-09
NIM1K	ENSG00000177453	protein_coding	93.7832734	1.55435617	0.16075722	9.66896626	4.0848E-22	2.6747E-21
GPR4	ENSG00000177464	protein_coding	3086.05193	1.92833059	0.11413806	16.8947209	4.9204E-64	1.9018E-62
RBM44	ENSG00000177483	protein_coding	34.5820293	1.41863201	0.1436188	9.87776008	5.1982E-23	3.5613E-22
IRX3	ENSG00000177508	protein_coding	3792.9034	1.49931316	0.13310393	11.2642295	1.9707E-29	1.9067E-28

ST8SIA3	ENSG00000177511	protein_coding	2.86550195	2.42336135	0.45379973	5.34015598	9.2867E-08	2.3284E-07
OR2B11	ENSG00000177535	protein_coding	0.98347473	1.26779916	0.3337532	3.79861276	0.00014551	0.00026924
CD163	ENSG00000177575	protein_coding	5380.86328	1.8524187	0.16547249	11.1947233	4.3275E-29	4.1132E-28
PIDD1	ENSG00000177595	protein_coding	744.024185	1.38520898	0.10993929	12.599763	2.1178E-36	2.8478E-35
HASPIN	ENSG00000177602	protein_coding	41.3863999	1.71781059	0.13133915	13.0791966	4.3299E-39	6.5228E-38
PGBD5	ENSG00000177614	protein_coding	1350.79772	1.76644531	0.15277228	11.5626038	6.3746E-31	6.5796E-30
MBOAT4	ENSG00000177669	protein_coding	25.8193847	2.36838905	0.12696471	18.6539162	1.1736E-77	7.5986E-76
CD163L1	ENSG00000177675	protein_coding	400.653025	1.50860269	0.15978846	9.44124923	3.6836E-21	2.2903E-20
SRRM3	ENSG00000177679	protein_coding	207.804288	1.1908241	0.21068406	5.6521793	1.5843E-08	4.2356E-08
CRACR2B	ENSG00000177685	protein_coding	854.369757	1.2063681	0.13491558	8.94165171	3.834E-19	2.1293E-18
SUMO4	ENSG00000177688	protein_coding	8.67130944	1.55319168	0.19569348	7.93685979	2.0736E-15	9.1569E-15
MAGEB10	ENSG00000177689	protein_coding	0.77502451	1.59541527	0.72164418	2.21080598	0.02704928	0.03805794
SLC35G5	ENSG00000177710	protein_coding	5.97008712	1.27417935	0.22280206	5.71888507	1.0723E-08	2.9038E-08
ANXA2R	ENSG00000177721	protein_coding	242.920996	2.02944906	0.11883717	17.0775617	2.1804E-65	8.8532E-64
PCDHB9	ENSG00000177839	protein_coding	263.433006	1.58548279	0.12762757	12.4227296	1.9673E-35	2.5333E-34
AP3S1	ENSG00000177879	protein_coding	3045.964	1.02530112	0.06759959	15.1672681	5.8251E-52	1.501E-50
MAMDC4	ENSG00000177943	protein_coding	853.450039	1.77233107	0.148495	11.9352916	7.7487E-33	8.7923E-32
ODF3	ENSG00000177947	protein_coding	1.54984352	2.7254359	0.34879079	7.81395608	5.542E-15	2.3785E-14
LCN15	ENSG00000177984	protein_coding	2.73086009	1.58396429	0.3533086	4.48323164	7.3521E-06	1.5503E-05
ODF3B	ENSG00000177989	protein_coding	1406.734	3.52787591	0.13821247	25.5250191	1.04E-143	5.61E-141
SPATA31E1	ENSG00000177992	protein_coding	3.41456606	1.84435679	0.29348422	6.28434737	3.2923E-10	1.0091E-09
GPR150	ENSG00000178015	protein_coding	17.7276071	2.22847496	0.20012673	11.1353191	8.4442E-29	7.9143E-28
TSPYL6	ENSG00000178021	protein_coding	2.17374153	1.59884793	0.28049878	5.70001738	1.198E-08	3.233E-08
LRRC75B	ENSG00000178026	protein_coding	2099.22598	1.43450367	0.12652645	11.3375798	8.5476E-30	8.3993E-29
ZNF114	ENSG00000178150	protein_coding	175.520806	2.47573708	0.25921004	9.55108498	1.2835E-21	8.1828E-21

SPINK6	ENSG00000178172	protein_coding	1.0294081	1.59980875	0.40397446	3.96017298	7.4895E-05	0.00014275
ZC3H12D	ENSG00000178199	protein_coding	186.086356	1.52179672	0.12499696	12.1746701	4.2411E-34	5.1148E-33
PRSS36	ENSG00000178226	protein_coding	114.982843	1.07341678	0.10009009	10.7245065	7.8103E-27	6.6143E-26
SPAG11A	ENSG00000178287	protein_coding	0.49437359	1.52893658	0.60909543	2.51017575	0.01206711	0.01784702
TMPRSS9	ENSG00000178297	protein_coding	34.228461	2.71921319	0.18637632	14.5899073	3.2564E-48	7.1699E-47
ZNF354B	ENSG00000178338	protein_coding	576.104659	1.10772016	0.08768092	12.6335372	1.3794E-36	1.8713E-35
CALML5	ENSG00000178372	protein_coding	1.14979496	2.48355935	0.6632701	3.74441625	0.00018081	0.00033128
DNAJC22	ENSG00000178401	protein_coding	2921.68594	1.08746884	0.10960946	9.92130416	3.3633E-23	2.3283E-22
NEUROG2	ENSG00000178403	protein_coding	0.5657939	1.04363264	0.42423837	2.46001473	0.01389313	0.02037153
TIMM23B-AGAP6	ENSG00000178440	protein_coding	12.1284906	1.25624096	0.16331918	7.69193792	1.4492E-14	6.0367E-14
CD28	ENSG00000178562	protein_coding	283.55971	1.4145924	0.14154018	9.99428149	1.6145E-23	1.1369E-22
PSAPL1	ENSG00000178597	protein_coding	0.71208138	1.57622868	0.64635315	2.43864934	0.01474227	0.02153533
OTOS	ENSG00000178602	protein_coding	3.60558176	1.57064162	0.45304773	3.46683474	0.00052663	0.00091645
GPR35	ENSG00000178623	protein_coding	438.189165	2.41635193	0.20171167	11.9792373	4.565E-33	5.2461E-32
CPNE7	ENSG00000178773	protein_coding	181.857279	2.22987184	0.23472584	9.49989923	2.1009E-21	1.3251E-20
C5orf46	ENSG00000178776	protein_coding	885.95415	7.70552816	0.32554824	23.6693894	7.454E-124	2.341E-121
CD300LB	ENSG00000178789	protein_coding	115.432148	1.71696242	0.13403793	12.8095264	1.4502E-37	2.0534E-36
GDPD4	ENSG00000178795	protein_coding	15.3903958	1.0726847	0.18120649	5.91968136	3.2257E-09	9.1321E-09
H1-8	ENSG00000178804	protein_coding	0.44757414	1.1244395	0.34179963	3.28976216	0.00100272	0.00169253
TRIM73	ENSG00000178809	protein_coding	14.237732	2.03047584	0.1853468	10.9550086	6.2872E-28	5.6259E-27
MSC	ENSG00000178860	protein_coding	2213.35467	3.07472195	0.17477265	17.5926948	2.8023E-69	1.3081E-67
APOLD1	ENSG00000178878	protein_coding	14232.2403	1.94864387	0.15125363	12.883287	5.5903E-38	8.0334E-37
RFLNA	ENSG00000178882	protein_coding	188.978621	1.40921188	0.25245489	5.58203441	2.3772E-08	6.2542E-08
TPRX1	ENSG00000178928	protein_coding	0.48068053	1.20459096	0.53012712	2.27226812	0.02307032	0.03279785
ERICH3	ENSG00000178965	protein_coding	31.6212739	1.23227569	0.21223565	5.8061673	6.3919E-09	1.7645E-08

AURKB	ENSG00000178999	protein_coding	173.057646	3.02822055	0.16332573	18.5409885	9.6433E-77	6.0473E-75
EXOC3L1	ENSG00000179044	protein_coding	920.333957	1.50882304	0.10324538	14.6139526	2.2885E-48	5.0802E-47
TRIML2	ENSG00000179046	protein_coding	4.63659492	3.65244449	0.37966986	9.62005389	6.5797E-22	4.2656E-21
C9orf50	ENSG00000179058	protein_coding	40.6451019	1.07703919	0.18030358	5.9734765	2.3225E-09	6.6599E-09
HTR1F	ENSG00000179097	protein_coding	108.68804	1.62401936	0.17147676	9.47078378	2.7775E-21	1.7401E-20
HES7	ENSG00000179111	protein_coding	8.13991896	1.04007023	0.23218474	4.47949443	7.482E-06	1.5764E-05
GIMAP7	ENSG00000179144	protein_coding	2139.43375	1.17538111	0.10334595	11.3732676	5.682E-30	5.6172E-29
SIGLECL1	ENSG00000179213	protein_coding	1.42407035	2.42327045	0.41312277	5.86573924	4.4714E-09	1.2513E-08
GVQW3	ENSG00000179240	protein_coding	251.061623	1.01317284	0.11753724	8.62001552	6.6945E-18	3.4551E-17
LDLRAD3	ENSG00000179241	protein_coding	1205.58773	1.46768528	0.08679784	16.9092382	3.8465E-64	1.4943E-62
CDH4	ENSG00000179242	protein_coding	1603.97656	4.06449706	0.23504602	17.292346	5.3721E-67	2.3123E-65
DAND5	ENSG00000179284	protein_coding	16.227654	2.73021617	0.21792861	12.5280298	5.2449E-36	6.9209E-35
RTL3	ENSG00000179300	protein_coding	4.18208742	1.37607544	0.29524409	4.66080611	3.1497E-06	6.8788E-06
FAM156B	ENSG00000179304	protein_coding	1.05473091	1.4014931	0.28539625	4.91069205	9.0756E-07	2.0854E-06
RAB39A	ENSG00000179331	protein_coding	56.3892158	1.39884744	0.16811888	8.32058531	8.753E-17	4.2174E-16
HLA-DQB1	ENSG00000179344	protein_coding	18266.3418	2.28654465	0.13521774	16.9100937	3.791E-64	1.474E-62
VWA1	ENSG00000179403	protein_coding	9353.20028	1.30152269	0.11025736	11.804406	3.704E-32	4.0737E-31
DNAJB8	ENSG00000179407	protein_coding	0.4854304	1.16290792	0.32559006	3.57169353	0.00035468	0.0006289
FJX1	ENSG00000179431	protein_coding	851.083187	1.50715018	0.10014138	15.0502241	3.4407E-51	8.5394E-50
ALOX12B	ENSG00000179477	protein_coding	50.0600988	1.93212204	0.17518416	11.0290913	2.7665E-28	2.5187E-27
LBX2	ENSG00000179528	protein_coding	63.2398131	2.04001231	0.12979456	15.7172403	1.1523E-55	3.354E-54
DNHD1	ENSG00000179532	protein_coding	673.57092	1.56308607	0.12821709	12.1909339	3.4742E-34	4.2065E-33
RNF151	ENSG00000179580	protein_coding	1.12000005	1.65030123	0.30852673	5.34897318	8.8455E-08	2.2215E-07
CIITA	ENSG00000179583	protein_coding	2533.84791	1.44004076	0.11695998	12.3122526	7.782E-35	9.7124E-34
ALOX15B	ENSG00000179593	protein_coding	238.14792	4.27786973	0.21885237	19.5468281	4.3898E-85	3.6856E-83

GRM8	ENSG00000179603	protein_coding	494.2295	2.02348335	0.18588865	10.8854594	1.3522E-27	1.1874E-26
RPRML	ENSG00000179673	protein_coding	1.21564129	1.294649	0.34181997	3.78751717	0.00015216	0.00028092
WDR97	ENSG00000179698	protein_coding	89.3581579	2.42241838	0.1823285	13.2860106	2.7907E-40	4.4052E-39
NLRP8	ENSG00000179709	protein_coding	1.32981498	2.14952256	0.46566065	4.61607087	3.9107E-06	8.4662E-06
PCED1B	ENSG00000179715	protein_coding	529.531733	1.68728945	0.10511645	16.0516209	5.569E-58	1.7667E-56
FOXS1	ENSG00000179772	protein_coding	406.997683	1.57981541	0.15056854	10.4923343	9.3685E-26	7.4897E-25
CDH5	ENSG00000179776	protein_coding	9285.69007	1.231252	0.11040675	11.1519628	7.0044E-29	6.5864E-28
LRRC3B	ENSG00000179796	protein_coding	14.2651157	1.59855229	0.24739927	6.46142688	1.0372E-10	3.3009E-10
MRGPRX3	ENSG00000179826	protein_coding	0.75970389	1.32680285	0.43784437	3.03030698	0.00244305	0.00394685
NKPD1	ENSG00000179846	protein_coding	13.4844734	2.67319982	0.18799708	14.219369	6.9477E-46	1.3847E-44
GIPC3	ENSG00000179855	protein_coding	1130.75382	1.25493151	0.11203673	11.2010718	4.0283E-29	3.8335E-28
CITED4	ENSG00000179862	protein_coding	1187.69142	1.02930008	0.1351316	7.61702	2.596E-14	1.0635E-13
NLRP11	ENSG00000179873	protein_coding	75.4581857	1.03403234	0.17484382	5.91403426	3.3383E-09	9.4358E-09
CCR8	ENSG00000179934	protein_coding	17.278102	2.78919977	0.22755656	12.2571717	1.5377E-34	1.8919E-33
GOLGA8J	ENSG00000179938	protein_coding	0.62209525	1.05128198	0.39034903	2.69318453	0.00707731	0.01081472
WFDC11	ENSG00000180083	protein_coding	0.46353128	1.42821817	0.5499637	2.5969317	0.00940606	0.01413438
TMEM86B	ENSG00000180089	protein_coding	101.803282	1.26084611	0.12727736	9.9062873	3.909E-23	2.6963E-22
SEPTIN1	ENSG00000180096	protein_coding	735.655587	1.82808942	0.10958492	16.6819437	1.7735E-62	6.473E-61
TDRD6	ENSG00000180113	protein_coding	140.752015	1.86752793	0.13911781	13.4240751	4.3701E-41	7.1266E-40
C12orf40	ENSG00000180116	protein_coding	2.76472745	2.91765575	0.39268086	7.4300941	1.0852E-13	4.2716E-13
F2	ENSG00000180210	protein_coding	249.94931	5.41794488	0.35791898	15.13735	9.1841E-52	2.3324E-50
FAM71C	ENSG00000180219	protein_coding	1.47046483	1.99529308	0.33831732	5.89769702	3.6861E-09	1.0385E-08
ADGRD2	ENSG00000180264	protein_coding	1.85920304	1.44070264	0.33537069	4.29585135	1.7402E-05	3.5381E-05
KCTD4	ENSG00000180332	protein_coding	39.8917031	1.1523928	0.23025273	5.00490392	5.589E-07	1.3098E-06
ITPRID1	ENSG00000180347	protein_coding	4.2562166	1.03522611	0.25582974	4.04654329	5.198E-05	0.00010082

HCLS1	ENSG00000180353	protein_coding	4429.30871	1.89481418	0.09325077	20.3195549	8.6356E-92	9.3144E-90
DEFB124	ENSG00000180383	protein_coding	2.56412242	1.21578227	0.25324265	4.80085899	1.5799E-06	3.5473E-06
ARHGAP45	ENSG00000180448	protein_coding	2541.67295	1.48657007	0.09126308	16.2888438	1.1845E-59	3.9749E-58
OR10Q1	ENSG00000180475	protein_coding	4.94049368	4.73660114	0.34755318	13.6284214	2.7139E-42	4.6647E-41
ZSCAN4	ENSG00000180532	protein_coding	10.3255979	1.64752032	0.19200137	8.58077367	9.4237E-18	4.8181E-17
BHLHA15	ENSG00000180535	protein_coding	50.358212	2.42020884	0.19147475	12.6398327	1.2732E-36	1.7299E-35
FUT7	ENSG00000180549	protein_coding	26.634404	1.80676032	0.15559401	11.6120174	3.5807E-31	3.7475E-30
H2BC4	ENSG00000180596	protein_coding	255.148335	1.06510252	0.15723218	6.77407472	1.252E-11	4.2584E-11
GSX2	ENSG00000180613	protein_coding	1.85030706	1.38377861	0.43380997	3.18982665	0.00142358	0.00236161
PRF1	ENSG00000180644	protein_coding	1398.55213	2.62478836	0.13080492	20.0664338	1.4505E-89	1.4392E-87
OR2A4	ENSG00000180658	protein_coding	65.5615048	6.92103855	0.31243735	22.1517641	1.003E-108	2.044E-106
C3orf22	ENSG00000180697	protein_coding	4.40708561	3.08194483	0.26917894	11.4494277	2.367E-30	2.3848E-29
OR10K2	ENSG00000180708	protein_coding	1.07179852	1.33048254	0.41214996	3.22815157	0.00124593	0.00208167
S1PR5	ENSG00000180739	protein_coding	232.89009	2.38912873	0.12958887	18.4362181	6.7286E-76	4.0532E-74
CLRN3	ENSG00000180745	protein_coding	2271.89497	1.4569609	0.18655033	7.81001528	5.7181E-15	2.4514E-14
CHST13	ENSG00000180767	protein_coding	602.071902	1.98697739	0.17463986	11.3775706	5.4085E-30	5.3538E-29
OR51E1	ENSG00000180785	protein_coding	465.618188	2.48466926	0.14387567	17.2695578	7.9751E-67	3.4103E-65
MAP6D1	ENSG00000180834	protein_coding	114.614268	1.09955093	0.11270413	9.75608378	1.7373E-22	1.1578E-21
LSMEM1	ENSG00000181016	protein_coding	121.159484	1.85369022	0.14011852	13.2294447	5.9324E-40	9.2123E-39
OR56B2P	ENSG00000181017	protein_coding	0.33486675	1.02510921	0.43023704	2.38266144	0.01718799	0.02488362
OR56B1	ENSG00000181023	protein_coding	1.30700945	2.74828468	0.35096176	7.83072395	4.8507E-15	2.0888E-14
AEN	ENSG00000181026	protein_coding	2074.77007	1.39055838	0.07044151	19.7406087	9.6607E-87	8.5992E-85
FCRL6	ENSG00000181036	protein_coding	154.709302	2.48826166	0.13808237	18.0201255	1.3544E-72	7.1609E-71
OR52N4	ENSG00000181074	protein_coding	21.9089754	2.5330378	0.19057067	13.2918554	2.581E-40	4.0826E-39
MAPK15	ENSG00000181085	protein_coding	627.091123	1.92435974	0.17911888	10.7434778	6.3602E-27	5.4093E-26

MUC16	ENSG00000181143	protein_coding	39.6548125	3.13654575	0.24301502	12.9067981	4.1208E-38	5.9628E-37
C4orf50	ENSG00000181215	protein_coding	9.58920961	2.48579485	0.21641649	11.4861618	1.5484E-30	1.5745E-29
H2AW	ENSG00000181218	protein_coding	163.187321	1.49439716	0.19573287	7.6348808	2.2603E-14	9.2986E-14
SLC25A41	ENSG00000181240	protein_coding	14.6395563	1.05370161	0.16898669	6.23541169	4.5059E-10	1.369E-09
OR5AK2	ENSG00000181273	protein_coding	0.52827616	1.05056793	0.48595422	2.16186603	0.0306285	0.04273838
SPEM1	ENSG00000181323	protein_coding	0.37692755	1.23606501	0.37962391	3.25602521	0.00112984	0.00189579
AATK	ENSG00000181409	protein_coding	305.754296	1.37342748	0.10807071	12.708601	5.2974E-37	7.3144E-36
SAGE1	ENSG00000181433	protein_coding	3.82042858	3.62204124	0.38726824	9.35279708	8.536E-21	5.2014E-20
TMEM45A	ENSG00000181458	protein_coding	2555.23628	3.48682645	0.1888928	18.4592877	4.3909E-76	2.6767E-74
RAP2B	ENSG00000181467	protein_coding	2695.33568	1.17713945	0.07766109	15.15739	6.7707E-52	1.7349E-50
C6orf223	ENSG00000181577	protein_coding	2939.26385	3.4949202	0.17641406	19.8108943	2.3979E-87	2.181E-85
OR52D1	ENSG00000181609	protein_coding	0.71641427	1.51830203	0.45998986	3.30072934	0.00096434	0.00163069
OR52H1	ENSG00000181616	protein_coding	0.89231121	2.07200604	0.44874276	4.61735816	3.8866E-06	8.4155E-06
FDCSP	ENSG00000181617	protein_coding	44.2889492	5.1530507	0.56626344	9.10009422	9.0254E-20	5.1957E-19
SLX1B	ENSG00000181625	protein_coding	2.44936326	2.4400519	0.30446624	8.01419521	1.1086E-15	4.9779E-15
ANKRD62	ENSG00000181626	protein_coding	2.06840014	2.23406347	0.3475904	6.42728758	1.299E-10	4.1E-10
P2RY13	ENSG00000181631	protein_coding	499.37396	1.67723944	0.13140433	12.7639588	2.6061E-37	3.6512E-36
ATG9B	ENSG00000181652	protein_coding	282.84206	2.92493162	0.19689727	14.855115	6.4457E-50	1.5239E-48
ODF3L2	ENSG00000181781	protein_coding	16.6943741	1.17294798	0.14240014	8.2369861	1.766E-16	8.3357E-16
OR6S1	ENSG00000181803	protein_coding	0.68667697	2.06462051	0.55165837	3.74257083	0.00018215	0.00033358
SLC9A9	ENSG00000181804	protein_coding	1383.19045	1.79617999	0.12450939	14.4260604	3.5478E-47	7.4739E-46
TIGIT	ENSG00000181847	protein_coding	381.544233	2.87866961	0.17675451	16.2862584	1.2356E-59	4.1405E-58
OR4C6	ENSG00000181903	protein_coding	1.08439261	1.55715851	0.66142268	2.3542563	0.01855981	0.02673455
CHST15	ENSG00000182022	protein_coding	5530.62482	1.67103489	0.12285799	13.6013532	3.9309E-42	6.6913E-41
ZNF716	ENSG00000182111	protein_coding	1.31647599	2.35964188	0.41830811	5.64091828	1.6915E-08	4.5112E-08

P2RY8	ENSG00000182162	protein_coding	1760.101	1.8299709	0.114215	16.0221597	8.9487E-58	2.8213E-56
SHISAL2A	ENSG00000182183	protein_coding	57.9422747	1.7956996	0.1354243	13.2598034	3.9593E-40	6.2051E-39
ARL6IP4	ENSG00000182196	protein_coding	129.474132	1.64959671	0.13367559	12.3402987	5.4952E-35	6.9034E-34
SHMT2	ENSG00000182199	protein_coding	11866.6969	1.92962612	0.08643548	22.324467	2.138E-110	4.58E-108
SYNM	ENSG00000182253	protein_coding	2495.58027	1.05681685	0.08500392	12.4325663	1.7396E-35	2.2457E-34
IZUMO1	ENSG00000182264	protein_coding	18.6046175	1.59141073	0.18810853	8.46006703	2.6722E-17	1.3302E-16
B4GALNT4	ENSG00000182272	protein_coding	172.060104	3.18355058	0.28649326	11.1121306	1.0951E-28	1.0191E-27
DCAF4L1	ENSG00000182308	protein_coding	20.691486	1.14552132	0.15479213	7.40038476	1.3579E-13	5.3076E-13
KCNJ14	ENSG00000182324	protein_coding	96.8778031	1.31418289	0.11500525	11.4271558	3.0597E-30	3.0631E-29
FBXL6	ENSG00000182325	protein_coding	661.330937	1.11467103	0.09830851	11.3384998	8.4582E-30	8.3133E-29
C1S	ENSG00000182326	protein_coding	21749.8275	1.3362689	0.17850101	7.4860578	7.0973E-14	2.8306E-13
KIAA2012	ENSG00000182329	protein_coding	5.77162293	1.30900237	0.21598361	6.06065602	1.3557E-09	3.9576E-09
DAOA	ENSG00000182346	protein_coding	0.46010002	1.45100595	0.51618777	2.81100413	0.00493872	0.00769111
NXPH4	ENSG00000182379	protein_coding	1309.05862	4.53115265	0.18456856	24.5499706	4.328E-133	1.804E-130
IFNL1	ENSG00000182393	protein_coding	2.70292035	2.59602257	0.32499275	7.98793987	1.3721E-15	6.1365E-15
KCNK4	ENSG00000182450	protein_coding	0.6852168	1.44954376	0.39067697	3.71033842	0.00020698	0.00037664
TEX19	ENSG00000182459	protein_coding	3.46546806	2.16252244	0.38852222	5.56602	2.6062E-08	6.8325E-08
CAPN12	ENSG00000182472	protein_coding	1580.31436	3.13063386	0.16287818	19.2207073	2.4836E-82	1.8695E-80
BGN	ENSG00000182492	protein_coding	57134.5622	1.07531236	0.14046097	7.65559528	1.9242E-14	7.9501E-14
TBPL2	ENSG00000182521	protein_coding	1.49159092	2.28886865	0.35134134	6.51465791	7.2856E-11	2.345E-10
RNASE10	ENSG00000182545	protein_coding	8.9891586	2.33081056	0.1869043	12.4706095	1.08E-35	1.4044E-34
CSF1R	ENSG00000182578	protein_coding	6263.63778	1.59712046	0.11885484	13.437572	3.6419E-41	5.9582E-40
VCX	ENSG00000182583	protein_coding	1.94805949	1.91233655	0.31684544	6.03555021	1.5842E-09	4.6004E-09
KRTAP11-1	ENSG00000182591	protein_coding	0.3987279	1.26264754	0.50971474	2.47716505	0.01324306	0.01947002
SNORC	ENSG00000182600	protein_coding	140.1679	1.72064083	0.16913912	10.1729325	2.619E-24	1.9293E-23

NTM	ENSG00000182667	protein_coding	814.610652	1.74756575	0.19365092	9.02430915	1.8083E-19	1.0238E-18
PPP1R27	ENSG00000182676	protein_coding	3.70326142	1.26531838	0.27849227	4.54345965	5.5338E-06	1.1808E-05
BRICD5	ENSG00000182685	protein_coding	124.704469	1.09144097	0.1549174	7.04530902	1.8505E-12	6.6831E-12
GRID1	ENSG00000182771	protein_coding	215.030594	1.47664739	0.16306247	9.05571593	1.3567E-19	7.7434E-19
MAGEB17	ENSG00000182798	protein_coding	5.99063313	1.68449679	0.21949207	7.67452226	1.6604E-14	6.8918E-14
LCK	ENSG00000182866	protein_coding	875.391362	1.28879554	0.1429253	9.0172666	1.9284E-19	1.0901E-18
ADGRG3	ENSG00000182885	protein_coding	61.3145098	1.56264137	0.17553767	8.90202867	5.4835E-19	3.022E-18
WFDC10B	ENSG00000182931	protein_coding	7.76787381	3.68801728	0.35740193	10.318963	5.7845E-25	4.4069E-24
ODF3L1	ENSG00000182950	protein_coding	32.0723276	1.01257271	0.14785385	6.84847035	7.4644E-12	2.5813E-11
GJC1	ENSG00000182963	protein_coding	2185.49158	2.73478396	0.11849341	23.0796283	7.417E-118	1.91E-115
SOX1	ENSG00000182968	protein_coding	4.81180105	4.17301166	0.53701264	7.77078846	7.7999E-15	3.3101E-14
ZNF320	ENSG00000182986	protein_coding	3571.49079	1.12647973	0.09763508	11.537654	8.5218E-31	8.7413E-30
PYCR1	ENSG00000183010	protein_coding	537.254765	1.70968766	0.18054605	9.4695378	2.8108E-21	1.7603E-20
MCEMP1	ENSG00000183019	protein_coding	30.7084986	2.03272729	0.20078916	10.1236905	4.3375E-24	3.1521E-23
OR1G1	ENSG00000183024	protein_coding	0.83528767	2.30311305	0.35388508	6.50808176	7.6116E-11	2.4474E-10
OTOP2	ENSG00000183034	protein_coding	1.61822713	1.37608	0.30251149	4.54885195	5.3939E-06	1.152E-05
CAMK1D	ENSG00000183049	protein_coding	1686.85078	1.2739117	0.07858176	16.2112899	4.1967E-59	1.382E-57
NKX2-5	ENSG00000183072	protein_coding	6.03472225	4.37441218	0.4786537	9.13899163	6.3037E-20	3.6671E-19
NEB	ENSG00000183091	protein_coding	1056.16217	1.58296599	0.25616083	6.17957871	6.4273E-10	1.9272E-09
GPR19	ENSG00000183150	protein_coding	31.2434327	2.2134159	0.13601676	16.2731117	1.5317E-59	5.1178E-58
C2CD4C	ENSG00000183186	protein_coding	123.805978	1.42107626	0.15044391	9.44588747	3.5241E-21	2.1928E-20
POTEC	ENSG00000183206	protein_coding	0.6074518	1.61417807	0.46591447	3.46453735	0.00053114	0.00092392
RIMBP3C	ENSG00000183246	protein_coding	2.78098962	2.6124171	0.27493224	9.50203974	2.0582E-21	1.2985E-20
DDX41	ENSG00000183258	protein_coding	6266.31503	1.03197161	0.0725449	14.2252823	6.3846E-46	1.2747E-44
OR52E8	ENSG00000183269	protein_coding	5.07301811	1.48918062	0.59151828	2.51755638	0.01181721	0.0175017

CCDC60	ENSG00000183273	protein_coding	10.5477191	1.72965621	0.22362735	7.734547	1.0377E-14	4.3714E-14
FAM9A	ENSG00000183304	protein_coding	1.9765878	3.39553636	0.41895606	8.10475535	5.2852E-16	2.4201E-15
TMEM121B	ENSG00000183307	protein_coding	68.9708378	1.47439545	0.11662585	12.6420987	1.2371E-36	1.6832E-35
OR2T34	ENSG00000183310	protein_coding	0.50334872	1.06115066	0.45216998	2.34679592	0.01893562	0.02724336
EPHA10	ENSG00000183317	protein_coding	88.8645762	1.34011505	0.28158758	4.75914124	1.9442E-06	4.3291E-06
SPDYE4	ENSG00000183318	protein_coding	1.41469008	1.31080335	0.29813154	4.39672814	1.0989E-05	2.2786E-05
REC114	ENSG00000183324	protein_coding	2.82018106	1.03681808	0.25144059	4.12351115	3.7314E-05	7.3517E-05
GBP6	ENSG00000183347	protein_coding	14.1946082	1.72385934	0.17570115	9.81131522	1.0065E-22	6.803E-22
PMCH	ENSG00000183395	protein_coding	12.8139203	4.8627551	0.33260696	14.6201245	2.0902E-48	4.6535E-47
NPIPA1	ENSG00000183426	protein_coding	220.159573	1.72606115	0.13138588	13.1373414	2.0117E-39	3.0689E-38
GRIN2A	ENSG00000183454	protein_coding	887.417369	1.60787235	0.24743718	6.49810319	8.1339E-11	2.6089E-10
TREX2	ENSG00000183479	protein_coding	182.823784	2.63692459	0.20813765	12.669138	8.7673E-37	1.2008E-35
MEX3B	ENSG00000183496	protein_coding	172.649987	1.17245982	0.11384394	10.2988335	7.1321E-25	5.4069E-24
KLRC4	ENSG00000183542	protein_coding	7.91060669	2.3278298	0.22423499	10.3812067	3.0193E-25	2.3443E-24
C10orf120	ENSG00000183559	protein_coding	0.65529599	1.42618041	0.59949921	2.37895295	0.01736189	0.02512268
GKN2	ENSG00000183607	protein_coding	0.622795	1.43758964	0.39356414	3.65274549	0.00025945	0.00046702
CCR3	ENSG00000183625	protein_coding	9.27341432	1.09650994	0.18707538	5.86132697	4.5918E-09	1.2835E-08
C11orf88	ENSG00000183644	protein_coding	11.1626299	1.61960779	0.14875746	10.8875735	1.3211E-27	1.1618E-26
BMP8A	ENSG00000183682	protein_coding	51.239543	1.4103506	0.14152641	9.96528224	2.1626E-23	1.5128E-22
RFLNB	ENSG00000183688	protein_coding	3122.41582	1.44381172	0.11597242	12.4496123	1.4053E-35	1.8208E-34
NOG	ENSG00000183691	protein_coding	137.279836	2.51359937	0.26983085	9.31546314	1.2142E-20	7.3422E-20
IFNL2	ENSG00000183709	protein_coding	0.58302214	1.73338987	0.44536389	3.89207544	9.939E-05	0.00018719
TRIM52	ENSG00000183718	protein_coding	1149.00249	1.08536229	0.08662959	12.5287709	5.1962E-36	6.8586E-35
FIGLA	ENSG00000183733	protein_coding	1.1052286	2.02248887	0.3375078	5.99242119	2.0674E-09	5.9496E-09
CHEK2	ENSG00000183765	protein_coding	370.434142	1.21311495	0.08725047	13.9038221	6.0047E-44	1.1081E-42

FOXL2	ENSG00000183770	protein_coding	2.95180319	1.55289226	0.26019429	5.96820272	2.3988E-09	6.868E-09
SLC35F3	ENSG00000183780	protein_coding	140.512322	1.2597984	0.22050899	5.71313848	1.1091E-08	2.9996E-08
TUBA8	ENSG00000183785	protein_coding	17.4251126	1.4104771	0.13863339	10.1741517	2.5864E-24	1.9056E-23
NPIPA5	ENSG00000183793	protein_coding	65.7206384	1.27562755	0.18309877	6.96688228	3.2404E-12	1.1492E-11
CCR4	ENSG00000183813	protein_coding	167.934019	1.41483249	0.15810379	8.94875775	3.5951E-19	2.0017E-18
PNMA3	ENSG00000183837	protein_coding	466.206181	1.41614163	0.20722825	6.83372858	8.2735E-12	2.8521E-11
IQGAP3	ENSG00000183856	protein_coding	512.26799	2.47092367	0.14564279	16.9656437	1.4747E-64	5.8229E-63
AC138969.1	ENSG00000183889	protein_coding	28.0736853	1.70167771	0.16020351	10.6219756	2.3552E-26	1.9422E-25
SH2D1A	ENSG00000183918	protein_coding	270.945382	2.46234468	0.16382313	15.030507	4.6344E-51	1.1404E-49
KCNH8	ENSG00000183960	protein_coding	23.4387429	1.27521617	0.24095624	5.29231433	1.2078E-07	2.9966E-07
ADAP2	ENSG00000184060	protein_coding	1430.05733	1.83722897	0.08706944	21.1007327	7.831E-99	1.1149E-96
TRIML1	ENSG00000184108	protein_coding	1.704336	2.04923355	0.34492059	5.94117491	2.8299E-09	8.0499E-09
TSPYL2	ENSG00000184205	protein_coding	2475.13986	1.15832831	0.1106612	10.4673395	1.2202E-25	9.6928E-25
GOLGA6L4	ENSG00000184206	protein_coding	10.1012819	1.69226984	0.19501011	8.6778571	4.033E-18	2.1116E-17
DGAT2L6	ENSG00000184210	protein_coding	1.4063253	1.76641063	0.31660377	5.57924693	2.4156E-08	6.3509E-08
DEFB108B	ENSG00000184276	protein_coding	0.75352864	1.64276118	0.4065688	4.04054909	5.3326E-05	0.00010332
CLECL1	ENSG00000184293	protein_coding	51.3118568	2.29951043	0.15528567	14.808259	1.2955E-49	3.0162E-48
SRPK3	ENSG00000184343	protein_coding	37.8827602	1.07549393	0.17009827	6.32277982	2.569E-10	7.9319E-10
H1-5	ENSG00000184357	protein_coding	2.43048338	2.73030748	0.42695489	6.39483825	1.6072E-10	5.0396E-10
MAP7D2	ENSG00000184368	protein_coding	2047.87897	2.37915468	0.24770283	9.60487482	7.6251E-22	4.9273E-21
CSF1	ENSG00000184371	protein_coding	4232.30251	1.00500803	0.08967249	11.20754	3.7446E-29	3.5739E-28
PLA2G6	ENSG00000184381	protein_coding	836.250268	1.24645461	0.13458524	9.26145097	2.0167E-20	1.2033E-19
A3GALT2	ENSG00000184389	protein_coding	5.68774293	2.2806903	0.23037131	9.90006204	4.1602E-23	2.8665E-22
KCND2	ENSG00000184408	protein_coding	38.6853633	1.36355741	0.2469246	5.52216116	3.3486E-08	8.7033E-08
KNTC1	ENSG00000184445	protein_coding	982.106204	1.18476378	0.08938563	13.2545217	4.2482E-40	6.6396E-39

CCR10	ENSG00000184451	protein_coding	87.8996555	1.68928112	0.13951441	12.1082916	9.5462E-34	1.1319E-32
WDR27	ENSG00000184465	protein_coding	753.369521	1.31489876	0.12698122	10.3550649	3.9692E-25	3.0575E-24
PTP4A3	ENSG00000184489	protein_coding	3185.21117	1.87713779	0.10421008	18.0130152	1.5401E-72	8.1334E-71
TMEM255B	ENSG00000184497	protein_coding	1076.06583	1.50700576	0.10709238	14.0720168	5.6437E-45	1.0829E-43
PROS1	ENSG00000184500	protein_coding	9027.58574	1.12281926	0.1232036	9.11352628	7.9747E-20	4.6128E-19
NUTM1	ENSG00000184507	protein_coding	2.3662751	1.87713801	0.33655963	5.57743076	2.441E-08	6.4142E-08
DHRS7C	ENSG00000184544	protein_coding	1.12569417	1.17980328	0.31758418	3.71493085	0.00020326	0.0003703
SPEM2	ENSG00000184560	protein_coding	3.03941114	3.64565241	0.30533509	11.9398408	7.3363E-33	8.3368E-32
SLITRK6	ENSG00000184564	protein_coding	37.972897	1.40149582	0.32047196	4.3732245	1.2242E-05	2.5261E-05
PIWIL3	ENSG00000184571	protein_coding	2.69357191	1.84689555	0.31426385	5.87689474	4.1803E-09	1.1722E-08
LPAR5	ENSG00000184574	protein_coding	419.565939	2.14864249	0.1222882	17.5703169	4.1584E-69	1.9333E-67
STING1	ENSG00000184584	protein_coding	3962.85482	1.02915539	0.08605276	11.9595864	5.7849E-33	6.6082E-32
C14orf180	ENSG00000184601	protein_coding	168.767861	2.4355162	0.31775899	7.66466503	1.793E-14	7.4214E-14
NELL2	ENSG00000184613	protein_coding	234.114382	1.36628204	0.17037148	8.01942945	1.0624E-15	4.775E-15
ODF4	ENSG00000184650	protein_coding	1.32329383	1.29673187	0.37725906	3.4372451	0.00058766	0.00101734
FOXD4L4	ENSG00000184659	protein_coding	1.07413044	1.36058766	0.30800252	4.41745633	9.9869E-06	2.0794E-05
CDCA2	ENSG00000184661	protein_coding	1171.81235	4.68560384	0.15767483	29.7168795	4.648E-194	9.685E-191
SEPTIN5	ENSG00000184702	protein_coding	1056.81094	1.70245168	0.11258198	15.1218843	1.1617E-51	2.9357E-50
LRRC26	ENSG00000184709	protein_coding	1.68748278	1.64232799	0.45223348	3.63159312	0.00028168	0.0005049
APOBR	ENSG00000184730	protein_coding	713.444492	1.67870072	0.11815514	14.207598	8.2197E-46	1.6354E-44
DDX53	ENSG00000184735	protein_coding	0.68492657	1.58455707	0.46115526	3.43605982	0.00059024	0.00102145
SMIM10	ENSG00000184785	protein_coding	739.211018	1.05235771	0.09012435	11.6767304	1.6762E-31	1.7861E-30
PRR16	ENSG00000184838	protein_coding	186.415143	1.74183829	0.12618637	13.803696	2.4211E-43	4.3559E-42
JAG2	ENSG00000184916	protein_coding	2636.36834	1.65949029	0.1068571	15.5299955	2.1739E-54	6.0324E-53
FMNL1	ENSG00000184922	protein_coding	1945.86794	2.22931348	0.10795284	20.6508091	9.5999E-95	1.1735E-92

OR6A2	ENSG00000184933	protein_coding	0.52380603	1.10986548	0.42173348	2.63167505	0.00849651	0.01284423
RFX6	ENSG00000185002	protein_coding	1.59218056	2.0286029	0.39130376	5.18421515	2.1693E-07	5.267E-07
F8	ENSG00000185010	protein_coding	5454.62304	1.03187636	0.15271607	6.75682898	1.4104E-11	4.7808E-11
LRRC14B	ENSG00000185028	protein_coding	50.635326	3.02419537	0.28264324	10.6996911	1.0212E-26	8.5941E-26
MROH2A	ENSG00000185038	protein_coding	99.9934734	2.5409728	0.28290194	8.98181458	2.6634E-19	1.4933E-18
SPDYE16	ENSG00000185040	protein_coding	0.94837667	2.0307621	0.33365137	6.08647915	1.1542E-09	3.3899E-09
ADSS1	ENSG00000185100	protein_coding	4161.60228	2.44836779	0.16522354	14.8185166	1.1121E-49	2.6011E-48
ANO9	ENSG00000185101	protein_coding	1045.4128	1.67305981	0.17086437	9.7917419	1.2217E-22	8.2228E-22
H2BC13	ENSG00000185130	protein_coding	7.23299992	2.66079824	0.24526962	10.8484624	2.0281E-27	1.7665E-26
MIXL1	ENSG00000185155	protein_coding	13.0597138	1.62928565	0.2489596	6.54437769	5.9744E-11	1.9341E-10
LRRC37B	ENSG00000185158	protein_coding	391.597543	1.03359394	0.06472619	15.9687121	2.111E-57	6.5518E-56
NRBP2	ENSG00000185189	protein_coding	2967.62157	1.47613621	0.12106981	12.1924385	3.4106E-34	4.1318E-33
PRSS57	ENSG00000185198	protein_coding	9.75751803	2.22274339	0.23246109	9.56178693	1.1574E-21	7.3978E-21
TNFAIP2	ENSG00000185215	protein_coding	5388.33796	1.27901347	0.11309935	11.3087605	1.1876E-29	1.1595E-28
IL3RA	ENSG00000185291	protein_coding	1244.33591	1.43617216	0.09797068	14.659203	1.1765E-48	2.6397E-47
SPPL2C	ENSG00000185294	protein_coding	0.93177491	2.32432669	0.3946492	5.88960199	3.8713E-09	1.0883E-08
SCN10A	ENSG00000185313	protein_coding	2.94396686	3.64314309	0.36268597	10.0448967	9.6749E-24	6.8996E-23
TEDC1	ENSG00000185347	protein_coding	288.174348	1.12476254	0.11002841	10.2224734	1.5728E-24	1.1729E-23
MAPK11	ENSG00000185386	protein_coding	947.529508	1.1958546	0.09795799	12.2078317	2.8231E-34	3.4328E-33
KPNA7	ENSG00000185467	protein_coding	9.39445136	3.38018541	0.26071087	12.9652648	1.9256E-38	2.813E-37
KRT6B	ENSG00000185479	protein_coding	7.53774668	3.21350784	0.50559052	6.35594956	2.0714E-10	6.4347E-10
STAC3	ENSG00000185482	protein_coding	237.688864	2.2721097	0.10377941	21.8936472	2.986E-106	5.611E-104
IRF7	ENSG00000185507	protein_coding	1535.68032	1.76046068	0.11711723	15.0316109	4.5579E-51	1.1221E-49
LMNTD2	ENSG00000185522	protein_coding	371.426175	1.9844418	0.14168509	14.006003	1.4324E-44	2.7067E-43
AHNAK2	ENSG00000185567	protein_coding	7246.38606	4.07212454	0.16655127	24.4496756	5.073E-132	2.04E-129

OLFML2A	ENSG00000185585	protein_coding	8597.8716	2.66900898	0.12919529	20.6587167	8.1502E-95	1.0016E-92
INKA1	ENSG00000185614	protein_coding	278.385707	1.33281289	0.11828039	11.2682487	1.8828E-29	1.8224E-28
PDIA2	ENSG00000185615	protein_coding	10.2005555	1.54083171	0.26397276	5.8370861	5.3122E-09	1.4773E-08
P4HB	ENSG00000185624	protein_coding	58536.9235	1.39725254	0.08848163	15.7914427	3.5634E-56	1.0518E-54
NDUFA4L2	ENSG00000185633	protein_coding	84930.1467	5.79271586	0.17390575	33.3095137	2.812E-243	1.841E-239
KRT79	ENSG00000185640	protein_coding	2.00700742	1.76732635	0.41444364	4.26433457	2.005E-05	4.0528E-05
SMIM23	ENSG00000185662	protein_coding	1.9719774	3.1215474	0.36399864	8.57571167	9.8476E-18	5.0281E-17
POU3F1	ENSG00000185668	protein_coding	26.8222925	1.04288096	0.1564528	6.66578662	2.6325E-11	8.759E-11
SNAI3	ENSG00000185669	protein_coding	98.3203666	1.29626486	0.09739779	13.3089761	2.0528E-40	3.2595E-39
LYG2	ENSG00000185674	protein_coding	12.0970735	1.86585651	0.19687723	9.47725894	2.6105E-21	1.6379E-20
PRAME	ENSG00000185686	protein_coding	802.20281	2.78005586	0.29425963	9.44762913	3.4659E-21	2.1575E-20
MYBL1	ENSG00000185697	protein_coding	442.900581	1.75205916	0.11951907	14.6592442	1.1757E-48	2.6394E-47
NRG3	ENSG00000185737	protein_coding	335.071067	1.9429562	0.12269138	15.836127	1.7529E-56	5.228E-55
KCNQ5	ENSG00000185760	protein_coding	54.5538447	1.77683728	0.16800751	10.5759394	3.853E-26	3.1383E-25
IKZF1	ENSG00000185811	protein_coding	1434.32606	2.05986818	0.12734471	16.1755306	7.5048E-59	2.4486E-57
EVI2B	ENSG00000185862	protein_coding	1411.46207	1.93348917	0.12207984	15.8379065	1.704E-56	5.0855E-55
NPIPB4	ENSG00000185864	protein_coding	168.683076	1.98094478	0.13573708	14.593984	3.0675E-48	6.7604E-47
PRSS38	ENSG00000185888	protein_coding	1.50197537	2.35223855	0.51656318	4.55363188	5.2728E-06	1.1271E-05
FFAR3	ENSG00000185897	protein_coding	2.89557542	2.12558814	0.28791248	7.38275783	1.5504E-13	6.0355E-13
TAS2R60	ENSG00000185899	protein_coding	0.58788423	1.24085531	0.4022798	3.08455786	0.00203855	0.00332098
C16orf54	ENSG00000185905	protein_coding	277.770254	1.76364734	0.12783431	13.7963532	2.6807E-43	4.8116E-42
C7orf61	ENSG00000185955	protein_coding	16.662252	1.83675503	0.16493221	11.1364241	8.3401E-29	7.82E-28
FAM186A	ENSG00000185958	protein_coding	37.4290848	1.7662122	0.15573183	11.3413693	8.1854E-30	8.052E-29
SHOX	ENSG00000185960	protein_coding	3.4408904	1.8815017	0.29874814	6.29795292	3.016E-10	9.2697E-10
CCIN	ENSG00000185972	protein_coding	9.98976415	1.05853362	0.19011604	5.56782912	2.5793E-08	6.7654E-08

SLITRK2	ENSG00000185985	protein_coding	324.535799	1.44451222	0.25919208	5.57313418	2.502E-08	6.5719E-08
PLK5	ENSG00000185988	protein_coding	5.36604654	1.78389704	0.39551187	4.51035019	6.4721E-06	1.3721E-05
RASA3	ENSG00000185989	protein_coding	2256.66484	1.38822523	0.07995618	17.3623248	1.5915E-67	6.9613E-66
HTR3E	ENSG00000186038	protein_coding	2.18027884	1.67358946	0.43284973	3.86644452	0.00011043	0.00020697
DLEU7	ENSG00000186047	protein_coding	55.9742097	1.68623648	0.14351641	11.7494332	7.1094E-32	7.7027E-31
KRT73	ENSG00000186049	protein_coding	2.67672137	2.35729837	0.29332745	8.03640562	9.2512E-16	4.1724E-15
CD300LF	ENSG00000186074	protein_coding	294.179067	3.00307094	0.12843763	23.3815502	6.585E-121	1.875E-118
ZPBP2	ENSG00000186075	protein_coding	1.06037262	1.48347571	0.40871765	3.62958561	0.00028388	0.00050865
NBPF6	ENSG00000186086	protein_coding	1.27042227	1.84756572	0.43661156	4.23160052	2.3203E-05	4.665E-05
GSAP	ENSG00000186088	protein_coding	2110.5434	1.18816675	0.08829563	13.4566886	2.8124E-41	4.6325E-40
TAS2R42	ENSG00000186136	protein_coding	1.13495025	1.58074579	0.35926429	4.39995237	1.0827E-05	2.2468E-05
PRR30	ENSG00000186143	protein_coding	1.47571684	2.32912613	0.56360032	4.13258486	3.5871E-05	7.0798E-05
UBL4B	ENSG00000186150	protein_coding	2.95045497	1.59714519	0.26812174	5.9567912	2.5724E-09	7.3434E-09
CCDC84	ENSG00000186166	protein_coding	876.504875	1.73818395	0.12571343	13.8265572	1.7626E-43	3.1949E-42
KIF18B	ENSG00000186185	protein_coding	167.261183	3.21844084	0.16783472	19.1762513	5.8445E-82	4.3283E-80
FFAR4	ENSG00000186188	protein_coding	99.0431227	2.76677466	0.16134004	17.1487164	6.4256E-66	2.6561E-64
BPIFB4	ENSG00000186191	protein_coding	2.25566192	1.16881903	0.45550084	2.5660085	0.01028763	0.01536792
BTLA	ENSG00000186265	protein_coding	66.2800839	1.41821862	0.16210523	8.74875312	2.1572E-18	1.1471E-17
GPAT2	ENSG00000186281	protein_coding	134.509797	1.37631105	0.13865798	9.92594218	3.2106E-23	2.2255E-22
PRELID2	ENSG00000186314	protein_coding	497.89799	2.06656241	0.10095367	20.4704044	3.9533E-93	4.5305E-91
TMEM212	ENSG00000186329	protein_coding	1.25147688	1.05102265	0.28350173	3.7072883	0.00020949	0.00038093
SLC36A3	ENSG00000186334	protein_coding	1.84458172	1.9964237	0.3305708	6.03932263	1.5476E-09	4.4984E-09
THBS2	ENSG00000186340	protein_coding	7779.44885	1.41507791	0.20051649	7.05716474	1.6993E-12	6.1576E-12
ANKRD37	ENSG00000186352	protein_coding	666.067397	1.31571683	0.09838672	13.372911	8.7066E-41	1.3984E-39
CD300E	ENSG00000186407	protein_coding	252.873941	1.08226146	0.14173925	7.63558072	2.248E-14	9.2499E-14

FCAR	ENSG00000186431	protein_coding	24.4736978	1.09432655	0.19420696	5.63484715	1.7521E-08	4.6665E-08
SPATA12	ENSG00000186451	protein_coding	9.55857709	1.14185295	0.19351265	5.90066291	3.6204E-09	1.0209E-08
BTN3A2	ENSG00000186470	protein_coding	6885.20306	1.9836003	0.09850239	20.1375858	3.4579E-90	3.4933E-88
KLK12	ENSG00000186474	protein_coding	1.23941169	2.61721489	0.54404722	4.8106392	1.5045E-06	3.3854E-06
MYT1L	ENSG00000186487	protein_coding	3.77439071	1.06583022	0.27439193	3.88433511	0.00010261	0.00019301
OR9Q1	ENSG00000186509	protein_coding	8.18264896	3.28058694	0.28211717	11.6284553	2.9539E-31	3.1115E-30
ARHGAP30	ENSG00000186517	protein_coding	2072.32868	2.01487464	0.11143236	18.0815927	4.4504E-73	2.403E-71
CYP4F8	ENSG00000186526	protein_coding	1.1040564	1.28260527	0.35210076	3.64272228	0.00026977	0.00048471
FSD2	ENSG00000186628	protein_coding	13.5760088	1.32018307	0.18662649	7.07393168	1.506E-12	5.4788E-12
SPDYE17	ENSG00000186645	protein_coding	0.89521935	1.61644624	0.40598722	3.98151996	6.8476E-05	0.00013102
CARMIL3	ENSG00000186648	protein_coding	156.240153	1.22499694	0.15724328	7.79045645	6.6768E-15	2.8474E-14
PRG2	ENSG00000186652	protein_coding	5.99626021	3.08541983	0.26288513	11.7367607	8.259E-32	8.9313E-31
CCDC73	ENSG00000186714	protein_coding	42.944882	1.68300527	0.13242024	12.7095773	5.2316E-37	7.2258E-36
MPPED1	ENSG00000186732	protein_coding	3.49937095	1.52264447	0.28116053	5.41556976	6.1094E-08	1.5561E-07
ZNF732	ENSG00000186777	protein_coding	12.295471	1.21182582	0.15417214	7.86021253	3.8348E-15	1.6622E-14
CXCR3	ENSG00000186810	protein_coding	325.222834	3.15874931	0.17865118	17.6810993	5.8642E-70	2.8237E-68
LILRB4	ENSG00000186818	protein_coding	1297.62144	3.1086209	0.14565979	21.3416544	4.662E-101	7.293E-99
TNFRSF4	ENSG00000186827	protein_coding	496.057918	3.28884927	0.13756298	23.9079534	2.532E-126	8.533E-124
KRT16	ENSG00000186832	protein_coding	20.4703495	1.29187102	0.33847742	3.81671255	0.00013524	0.00025108
KRT14	ENSG00000186847	protein_coding	33.9065094	2.86448933	0.31884898	8.98384354	2.6147E-19	1.4667E-18
TRABD2A	ENSG00000186854	protein_coding	184.488789	1.65651593	0.1897334	8.73075546	2.5298E-18	1.3399E-17
QRFPR	ENSG00000186867	protein_coding	741.450987	2.91824279	0.16914042	17.2533735	1.0555E-66	4.4969E-65
ERCC6L	ENSG00000186871	protein_coding	56.6716745	1.79414209	0.1437842	12.4780196	9.8404E-36	1.2815E-34
TNFRSF18	ENSG00000186891	protein_coding	84.4414593	2.92666214	0.17065847	17.149235	6.3685E-66	2.6348E-64
FGF3	ENSG00000186895	protein_coding	0.63040857	1.8385592	0.52328645	3.51348519	0.00044227	0.00077631

C1QL4	ENSG00000186897	protein_coding	434.604943	3.03328458	0.27253268	11.1299847	8.9651E-29	8.3837E-28
RTN4RL2	ENSG00000186907	protein_coding	270.511925	1.03351566	0.15355674	6.73051329	1.6907E-11	5.6957E-11
SERPINA11	ENSG00000186910	protein_coding	2.21274346	2.6278088	0.4306696	6.10168165	1.0496E-09	3.0953E-09
ZNF395	ENSG00000186918	protein_coding	18426.0591	2.61689362	0.1023668	25.5638892	3.848E-144	2.125E-141
FAM183A	ENSG00000186973	protein_coding	69.9280144	1.76506877	0.199525	8.84635382	9.0425E-19	4.9242E-18
RHD	ENSG00000187010	protein_coding	49.1029287	1.31773708	0.18725182	7.03724597	1.9608E-12	7.0669E-12
SAMD7	ENSG00000187033	protein_coding	0.74185326	1.81185747	0.3925645	4.61543887	3.9227E-06	8.4908E-06
GPR141	ENSG00000187037	protein_coding	76.764249	2.30039689	0.15242357	15.0921338	1.8245E-51	4.5727E-50
TMPRSS6	ENSG00000187045	protein_coding	50.9879249	2.50437306	0.22355758	11.2023625	3.97E-29	3.7812E-28
TMPRSS11A	ENSG00000187054	protein_coding	3.89450117	3.87860267	0.42469275	9.13272641	6.6796E-20	3.8818E-19
TMEM262	ENSG00000187066	protein_coding	65.7400029	1.55151971	0.11341621	13.6798764	1.3392E-42	2.3342E-41
C3orf70	ENSG00000187068	protein_coding	653.500626	1.49348294	0.11822226	12.6328408	1.3916E-36	1.8874E-35
LILRA5	ENSG00000187116	protein_coding	174.740418	1.24202762	0.13003822	9.55125058	1.2814E-21	8.1732E-21
SLIT1	ENSG00000187122	protein_coding	30.6986889	1.17684755	0.12363948	9.51838033	1.759E-21	1.1137E-20
SPATA21	ENSG00000187144	protein_coding	9.96313034	2.93244004	0.2766881	10.5983598	3.0325E-26	2.4833E-25
KRT12	ENSG00000187242	protein_coding	3.4133594	1.59948416	0.24298473	6.58265295	4.6213E-11	1.5097E-10
FAM9C	ENSG00000187268	protein_coding	9.35956538	2.09096201	0.19519226	10.7123204	8.91E-27	7.5165E-26
CIDEC	ENSG00000187288	protein_coding	39.550439	1.40825834	0.33154644	4.24754476	2.1613E-05	4.3564E-05
FPR3	ENSG00000187474	protein_coding	2031.05805	2.18961025	0.13630923	16.0635508	4.5948E-58	1.4627E-56
H1-6	ENSG00000187475	protein_coding	3.48844909	1.99984727	0.27172151	7.35991508	1.8403E-13	7.128E-13
CDHR4	ENSG00000187492	protein_coding	12.8569195	2.2573613	0.29651843	7.61288711	2.6804E-14	1.0969E-13
COL4A1	ENSG00000187498	protein_coding	75154.3229	1.86085238	0.11671903	15.9430078	3.1864E-57	9.823E-56
GJA4	ENSG00000187513	protein_coding	3426.89592	1.43110522	0.12156444	11.7724004	5.4161E-32	5.9114E-31
PRR27	ENSG00000187533	protein_coding	0.79950187	2.04428107	0.48882413	4.18203798	2.8891E-05	5.7539E-05
FOXD4L3	ENSG00000187559	protein_coding	0.51548185	1.38927366	0.32794166	4.2363439	2.2719E-05	4.5702E-05

DPPA3	ENSG00000187569	protein_coding	0.85860064	2.12129473	0.49399798	4.2941364	1.7537E-05	3.5648E-05
PLEKHN1	ENSG00000187583	protein_coding	174.270499	2.46236846	0.14075086	17.494518	1.5774E-68	7.1879E-67
TET3	ENSG00000187605	protein_coding	1350.68189	1.00630353	0.06635449	15.1655673	5.978E-52	1.5395E-50
ISG15	ENSG00000187608	protein_coding	2005.06912	1.53867714	0.13150489	11.7005315	1.2666E-31	1.3582E-30
RGPD1	ENSG00000187627	protein_coding	3.54471449	1.07196241	0.25498085	4.2040899	2.6213E-05	5.2426E-05
PERM1	ENSG00000187642	protein_coding	404.393187	3.08270148	0.21651518	14.2378074	5.3376E-46	1.0694E-44
SPRY4	ENSG00000187678	protein_coding	6967.23516	1.26194559	0.10226267	12.3402367	5.4994E-35	6.9068E-34
TRPV2	ENSG00000187688	protein_coding	1329.83431	1.95121881	0.08231782	23.7034794	3.32E-124	1.057E-121
EZHIP	ENSG00000187690	protein_coding	5.83047299	1.89729385	0.26365262	7.1961881	6.1919E-13	2.3097E-12
SLC18A3	ENSG00000187714	protein_coding	64.3789207	7.65268682	0.57190054	13.3811498	7.7932E-41	1.2566E-39
DNAJB13	ENSG00000187726	protein_coding	210.514724	3.73127193	0.22659524	16.4666825	6.3668E-61	2.2195E-59
GABRD	ENSG00000187730	protein_coding	849.405824	5.25448286	0.16209548	32.4159734	1.635E-230	7.494E-227
FANCA	ENSG00000187741	protein_coding	333.932161	1.63845804	0.1155328	14.1817568	1.1883E-45	2.3521E-44
OR52B6	ENSG00000187747	protein_coding	1.29152464	1.84562426	0.35257594	5.23468573	1.6527E-07	4.0559E-07
C9orf153	ENSG00000187753	protein_coding	13.149371	1.81728871	0.15150441	11.9949555	3.7762E-33	4.357E-32
LIN28B	ENSG00000187772	protein_coding	0.86873917	2.24443878	0.71625811	3.13356141	0.00172699	0.00283798
CARD9	ENSG00000187796	protein_coding	263.697808	1.49512662	0.11835766	12.632276	1.4016E-36	1.9004E-35
SOWAHD	ENSG00000187808	protein_coding	93.5137101	1.06577085	0.13111828	8.1283161	4.3529E-16	2.0049E-15
C2orf78	ENSG00000187833	protein_coding	0.80173471	1.49987747	0.42515793	3.52781254	0.00041901	0.00073703
PLSCR3	ENSG00000187838	protein_coding	42.5484773	1.5666339	0.11105555	14.1067597	3.4507E-45	6.6886E-44
EIF4EBP1	ENSG00000187840	protein_coding	2422.74501	2.11772973	0.13257407	15.9739366	1.9414E-57	6.0294E-56
TTC24	ENSG00000187862	protein_coding	22.4513718	3.75515426	0.24617645	15.2539132	1.5506E-52	4.0686E-51
SHISA7	ENSG00000187902	protein_coding	4.00204116	1.33798985	0.20050744	6.67301852	2.5059E-11	8.3515E-11
LRRC74B	ENSG00000187905	protein_coding	2.82715805	2.1219341	0.3478801	6.09961326	1.0633E-09	3.134E-09
CLEC17A	ENSG00000187912	protein_coding	27.0060525	1.47277152	0.20094536	7.32921401	2.3151E-13	8.8904E-13

LDLRAD2	ENSG00000187942	protein_coding	230.997699	1.14811284	0.11905601	9.64346815	5.2387E-22	3.4117E-21
KLHL17	ENSG00000187961	protein_coding	323.42176	1.11706801	0.11478628	9.73172028	2.2083E-22	1.4626E-21
PLA2G2C	ENSG00000187980	protein_coding	7.31447898	1.35691822	0.22761918	5.96135278	2.5016E-09	7.1498E-09
TPRG1	ENSG00000188001	protein_coding	156.864949	2.2669068	0.16481485	13.7542633	4.8015E-43	8.5378E-42
RTP5	ENSG00000188011	protein_coding	11.0621019	3.09205279	0.25253118	12.2442415	1.8036E-34	2.2094E-33
S100A3	ENSG00000188015	protein_coding	134.444092	1.05124749	0.13755726	7.64225379	2.1345E-14	8.7954E-14
C19orf67	ENSG00000188032	protein_coding	56.2595993	5.14673599	0.28067093	18.3372606	4.173E-75	2.4276E-73
NRN1L	ENSG00000188038	protein_coding	0.88930553	1.91662603	0.33247455	5.76473012	8.1789E-09	2.2362E-08
ARL4C	ENSG00000188042	protein_coding	5271.46639	1.57005256	0.15308707	10.255945	1.1128E-24	8.3724E-24
TREML4	ENSG00000188056	protein_coding	5.35012619	2.58335896	0.32380278	7.97818655	1.485E-15	6.6213E-15
RAB42	ENSG00000188060	protein_coding	1646.58632	4.86109651	0.20253328	24.0014699	2.684E-127	9.252E-125
PLA2G4E	ENSG00000188089	protein_coding	3.97583238	1.21133468	0.27139478	4.46336763	8.0681E-06	1.6941E-05
FAM25A	ENSG00000188100	protein_coding	0.95311423	1.84596441	0.61730459	2.99036236	0.00278647	0.00447422
DAZ1	ENSG00000188120	protein_coding	0.92769475	2.49591237	1.17431657	2.12541697	0.03355184	0.04656241
MAPK12	ENSG00000188130	protein_coding	972.745835	1.26263286	0.15700719	8.04187941	8.8471E-16	3.9964E-15
NUTM2G	ENSG00000188152	protein_coding	12.6481045	1.35247519	0.15673231	8.6292049	6.178E-18	3.1954E-17
OTOG	ENSG00000188162	protein_coding	22.9248977	2.20361404	0.19943059	11.049529	2.2037E-28	2.0143E-27
FAM166A	ENSG00000188163	protein_coding	8.54109915	1.84216804	0.251303	7.33046583	2.2935E-13	8.8129E-13
AGAP4	ENSG00000188234	protein_coding	93.4557618	1.07834275	0.11290549	9.55084404	1.2864E-21	8.2008E-21
IL17REL	ENSG00000188263	protein_coding	8.85135644	2.67621697	0.23016853	11.6272062	2.9975E-31	3.1552E-30
RUFY4	ENSG00000188282	protein_coding	60.9506104	4.95179388	0.2504281	19.7733154	5.0543E-87	4.543E-85
HES4	ENSG00000188290	protein_coding	531.036271	2.30238896	0.14061597	16.373595	2.9525E-60	1.0108E-58
PEAK3	ENSG00000188305	protein_coding	65.8795868	1.40246069	0.11984148	11.7026314	1.2356E-31	1.3265E-30
BSPH1	ENSG00000188334	protein_coding	0.53848059	1.64552033	0.55918011	2.94273758	0.00325324	0.00518611
ZP3	ENSG00000188372	protein_coding	290.253801	1.54841066	0.14070378	11.0047552	3.625E-28	3.2847E-27

C10orf99	ENSG00000188373	protein_coding	973.123436	7.226589	0.37526877	19.2571021	1.2308E-82	9.372E-81
PPP3R2	ENSG00000188386	protein_coding	1.52003478	1.06410225	0.29892619	3.5597491	0.00037121	0.00065686
PDCD1	ENSG00000188389	protein_coding	286.107285	3.31414922	0.20909838	15.8497123	1.4123E-56	4.2286E-55
TCTEX1D4	ENSG00000188396	protein_coding	11.1817439	1.07333176	0.1573161	6.82277132	8.9301E-12	3.0705E-11
NANOS2	ENSG00000188425	protein_coding	2.48965945	3.08144705	0.50096847	6.15098005	7.7006E-10	2.2965E-09
KRTDAP	ENSG00000188508	protein_coding	4.23513392	1.37350464	0.25286349	5.43180282	5.5788E-08	1.4253E-07
PAQR9	ENSG00000188582	protein_coding	19.8566531	3.6554421	0.26438526	13.8261948	1.7715E-43	3.2097E-42
FAM72B	ENSG00000188610	protein_coding	18.5612579	1.33974101	0.1396787	9.59159156	8.6737E-22	5.5828E-21
IGFL3	ENSG00000188624	protein_coding	0.99775535	2.20759565	0.42542716	5.18912722	2.1128E-07	5.137E-07
GOLGA8M	ENSG00000188626	protein_coding	41.5975909	1.92162528	0.17302574	11.1060079	1.1729E-28	1.0879E-27
ZNF177	ENSG00000188629	protein_coding	1.88150703	1.38423519	0.2578431	5.36851739	7.9387E-08	2.0035E-07
CC2D2B	ENSG00000188649	protein_coding	12.835005	1.58419873	0.17375868	9.11723492	7.7065E-20	4.4651E-19
RNASE9	ENSG00000188655	protein_coding	0.5954214	1.74509455	0.41404893	4.2147061	2.501E-05	5.0133E-05
PARVB	ENSG00000188677	protein_coding	2468.74566	1.08556942	0.11454527	9.47720869	2.6118E-21	1.6385E-20
SLC4A5	ENSG00000188687	protein_coding	74.1289344	1.37510142	0.17862312	7.69833941	1.3785E-14	5.7497E-14
DUPD1	ENSG00000188716	protein_coding	0.37148715	1.08821275	0.3875368	2.80802432	0.00498465	0.0077579
FSIP2	ENSG00000188738	protein_coding	179.239343	1.59244316	0.21305141	7.47445485	7.7525E-14	3.082E-13
PLET1	ENSG00000188771	protein_coding	2.24517074	1.80447493	0.32256533	5.59413796	2.2172E-08	5.848E-08
SKOR1	ENSG00000188779	protein_coding	30.183342	1.19500123	0.11503725	10.3879498	2.8134E-25	2.1881E-24
CATSPER4	ENSG00000188782	protein_coding	2.02433071	2.64383287	0.48791766	5.41860462	6.0066E-08	1.5309E-07
PLA2G2E	ENSG00000188784	protein_coding	0.44538888	1.40466619	0.50149266	2.8009706	0.00509492	0.00792091
TMCO2	ENSG00000188800	protein_coding	1.89057254	1.01319855	0.27655834	3.66359788	0.0002487	0.00044866
ZDHHC11	ENSG00000188818	protein_coding	165.919541	1.40863975	0.17044661	8.26440463	1.4039E-16	6.674E-16
CALHM6	ENSG00000188820	protein_coding	747.827275	2.95926218	0.15803696	18.7251266	3.0894E-78	2.0318E-76
GLRA4	ENSG00000188828	protein_coding	2.28158745	1.96833224	0.27969469	7.03743146	1.9582E-12	7.058E-12

FAM78B	ENSG00000188859	protein_coding	234.298882	1.37622918	0.13815731	9.96131991	2.2505E-23	1.5727E-22
LRRK2	ENSG00000188906	protein_coding	9241.30635	1.04622407	0.12694272	8.24170192	1.6978E-16	8.0252E-16
BSX	ENSG00000188909	protein_coding	0.59880118	1.60667786	0.46280176	3.47163302	0.0005173	0.00090111
SLC15A5	ENSG00000188991	protein_coding	2.74384833	1.4729979	0.29907009	4.92525978	8.4248E-07	1.9425E-06
LIPI	ENSG00000188992	protein_coding	4.80030423	1.36112447	0.29179514	4.66465782	3.0913E-06	6.7564E-06
LRRC66	ENSG00000188993	protein_coding	139.830384	1.93679267	0.14922317	12.9791687	1.6061E-38	2.3553E-37
SBSN	ENSG00000189001	protein_coding	9.87937403	3.37122031	0.43202088	7.8033735	6.0274E-15	2.5791E-14
KIR2DL4	ENSG00000189013	protein_coding	34.6681249	2.39381861	0.16463207	14.5404147	6.7184E-48	1.4562E-46
MAGEB16	ENSG00000189023	protein_coding	0.52860252	1.31002427	0.59866057	2.18825883	0.02865075	0.0401455
VHLL	ENSG00000189030	protein_coding	0.94403657	1.81785274	0.41859699	4.34272773	1.4072E-05	2.8872E-05
RNF222	ENSG00000189051	protein_coding	2.60155932	1.16236595	0.26648873	4.36178271	1.2901E-05	2.6557E-05
FAM111B	ENSG00000189057	protein_coding	238.660357	2.51569886	0.1188567	21.1658143	1.973E-99	2.8445E-97
VSTM1	ENSG00000189068	protein_coding	9.34482278	1.52853802	0.2052489	7.44724083	9.5313E-14	3.7649E-13
PRSS48	ENSG00000189099	protein_coding	1.39917826	1.37434458	0.32671768	4.20652038	2.5933E-05	5.1893E-05
PLAC9	ENSG00000189129	protein_coding	587.659478	1.22429213	0.13783921	8.88203122	6.565E-19	3.605E-18
ZAR1L	ENSG00000189167	protein_coding	0.85162084	1.52276186	0.31499014	4.83431599	1.336E-06	3.0206E-06
KRTAP10-12	ENSG00000189169	protein_coding	0.94723155	2.15818987	0.7132717	3.02576126	0.00248008	0.00400395
KRT77	ENSG00000189182	protein_coding	1.65473285	2.72505893	0.51241673	5.31805226	1.0488E-07	2.6172E-07
DCAF8L2	ENSG00000189186	protein_coding	0.80481881	1.80783484	0.6586749	2.7446542	0.00605747	0.00933913
SPANXN3	ENSG00000189252	protein_coding	0.55425063	1.40315788	0.61137755	2.29507592	0.02172877	0.03101375
DRICH1	ENSG00000189269	protein_coding	26.3743397	1.0177352	0.18943837	5.37238146	7.7704E-08	1.9631E-07
ALKAL2	ENSG00000189292	protein_coding	927.412632	1.65227114	0.20483404	8.06638942	7.2408E-16	3.2877E-15
KIAA0408	ENSG00000189367	protein_coding	10.45962	2.16448593	0.20209411	10.7102868	9.1079E-27	7.6792E-26
TBC1D28	ENSG00000189375	protein_coding	0.53613001	1.1467772	0.35861961	3.19775377	0.00138502	0.00230123
OTUD6A	ENSG00000189401	protein_coding	0.59898641	1.51283195	0.44342284	3.4117141	0.00064556	0.00111264

SH2D5	ENSG00000189410	protein_coding	124.572108	2.46678958	0.15723306	15.6887464	1.8059E-55	5.2262E-54
NCR1	ENSG00000189430	protein_coding	31.1921196	2.61220633	0.15563946	16.7837024	3.2117E-63	1.2088E-61
IL1RAP	ENSG00000196083	protein_coding	1015.44363	1.00865425	0.11546699	8.73543406	2.4272E-18	1.2863E-17
KIAA0895L	ENSG00000196123	protein_coding	1507.68884	2.0420062	0.12273732	16.6372076	3.7469E-62	1.3567E-60
HLA-DRB1	ENSG00000196126	protein_coding	44087.7422	1.61527237	0.10963169	14.7336269	3.9209E-49	8.9689E-48
PLEKHG4	ENSG00000196155	protein_coding	936.208494	2.11628067	0.14394959	14.7015401	6.301E-49	1.4278E-47
KIF19	ENSG00000196169	protein_coding	49.9971275	1.79580479	0.14775498	12.1539374	5.4668E-34	6.5586E-33
CTSE	ENSG00000196188	protein_coding	195.157501	2.82286826	0.27409082	10.2990252	7.1179E-25	5.3986E-24
SIRPB2	ENSG00000196209	protein_coding	348.066197	2.11602951	0.11731002	18.0379263	9.8159E-73	5.2322E-71
OR2T2	ENSG00000196240	protein_coding	0.89030818	1.31423628	0.39893964	3.29432364	0.00098659	0.00166683
BECN2	ENSG00000196289	protein_coding	2.22894111	3.66430368	0.47955171	7.64110226	2.1537E-14	8.8736E-14
ATP2A1	ENSG00000196296	protein_coding	102.237988	2.36369219	0.16967204	13.9309472	4.1087E-44	7.6378E-43
GIMAP5	ENSG00000196329	protein_coding	356.967381	1.33589753	0.11239211	11.8860432	1.3988E-32	1.5666E-31
CGB7	ENSG00000196337	protein_coding	7.45570244	2.3694114	0.22486414	10.5370801	5.828E-26	4.7085E-25
ADH7	ENSG00000196344	protein_coding	1.68849186	2.95927252	0.50325045	5.8803177	4.0948E-09	1.1487E-08
NTNG2	ENSG00000196358	protein_coding	181.338093	1.69009161	0.17285888	9.77729105	1.4093E-22	9.4534E-22
ELAVL3	ENSG00000196361	protein_coding	26.8982061	1.20916435	0.19733113	6.12759043	8.922E-10	2.645E-09
EVL	ENSG00000196405	protein_coding	4537.24056	1.67447075	0.08695841	19.2559965	1.2573E-82	9.5584E-81
PRTN3	ENSG00000196415	protein_coding	15.1459864	2.67409318	0.30846497	8.66903366	4.358E-18	2.2774E-17
C20orf204	ENSG00000196421	protein_coding	62.3887163	2.11794409	0.13378528	15.8309205	1.9041E-56	5.6754E-55
NBPF4	ENSG00000196427	protein_coding	1.65353487	2.34993301	0.42508291	5.52817565	3.2358E-08	8.4193E-08
ASMT	ENSG00000196433	protein_coding	6.04027208	2.05091087	0.20837	9.8426397	7.375E-23	5.0152E-22
NPIPB15	ENSG00000196436	protein_coding	122.198598	1.74493084	0.23082399	7.55957304	4.0439E-14	1.6363E-13
RFX8	ENSG00000196460	protein_coding	81.2822912	3.42187838	0.17607037	19.4347203	3.9255E-84	3.157E-82
FAM72A	ENSG00000196550	protein_coding	22.3369577	1.44715452	0.11370882	12.7268454	4.1945E-37	5.8284E-36

HBG2	ENSG00000196565	protein_coding	16.7611117	2.0153786	0.24994564	8.06326762	7.4282E-16	3.3704E-15
XRCC2	ENSG00000196584	protein_coding	109.39165	1.59615177	0.1197381	13.3303581	1.5416E-40	2.4631E-39
MMP1	ENSG00000196611	protein_coding	417.051119	2.08985447	0.25396592	8.22887763	1.8898E-16	8.8986E-16
UGT2B15	ENSG00000196620	protein_coding	3.93789335	1.05182331	0.31531219	3.33581555	0.0008505	0.00144704
TCF4	ENSG00000196628	protein_coding	7230.55028	1.17569523	0.10780902	10.905351	1.0867E-27	9.6116E-27
HRH1	ENSG00000196639	protein_coding	978.62702	1.01905192	0.11247233	9.0604677	1.2989E-19	7.417E-19
SLC30A10	ENSG00000196660	protein_coding	20.71091	2.93144802	0.39741542	7.37628155	1.6277E-13	6.3266E-13
TLR7	ENSG00000196664	protein_coding	764.779908	2.07738824	0.15201537	13.6656459	1.6285E-42	2.8278E-41
TRPV1	ENSG00000196689	protein_coding	16.1064061	1.36829131	0.17105323	7.99921366	1.2522E-15	5.6077E-15
HLA-DQA1	ENSG00000196735	protein_coding	15062.4607	2.1950662	0.13594029	16.1472822	1.1868E-58	3.842E-57
COL27A1	ENSG00000196739	protein_coding	4215.737	1.74705377	0.12729623	13.7243167	7.261E-43	1.2832E-41
GM2A	ENSG00000196743	protein_coding	6789.6153	1.35635435	0.08136077	16.6708637	2.1349E-62	7.7794E-61
H2AC13	ENSG00000196747	protein_coding	31.8309576	3.93689294	0.26002322	15.1405439	8.7487E-52	2.2268E-50
H2AC11	ENSG00000196787	protein_coding	82.8063151	1.77018347	0.20494677	8.63728415	5.7565E-18	2.9851E-17
CHRNG	ENSG00000196811	protein_coding	3.72440475	2.26098549	0.29151552	7.75596947	8.7671E-15	3.7099E-14
ADA	ENSG00000196839	protein_coding	726.123217	2.22429019	0.13450619	16.5367125	1.9962E-61	7.0555E-60
ARID5A	ENSG00000196843	protein_coding	1813.14771	1.39478118	0.09942147	14.0289734	1.0364E-44	1.9657E-43
H2AC7	ENSG00000196866	protein_coding	14.9799958	1.16539822	0.20989139	5.55238706	2.8179E-08	7.3702E-08
CBWD3	ENSG00000196873	protein_coding	40.1304986	1.05304235	0.11361495	9.26851902	1.8875E-20	1.1285E-19
H2BU1	ENSG00000196890	protein_coding	21.3672108	1.75156141	0.23087966	7.58646924	3.2874E-14	1.3375E-13
TEX43	ENSG00000196900	protein_coding	1.58468136	1.70254045	0.33979602	5.01047784	5.4295E-07	1.2744E-06
ANKRD36B	ENSG00000196912	protein_coding	100.669755	1.35573006	0.13886252	9.76311015	1.6211E-22	1.0825E-21
PDLIM7	ENSG00000196923	protein_coding	2387.81808	1.00473429	0.11106504	9.04636001	1.4781E-19	8.4236E-19
ZNF705A	ENSG00000196946	protein_coding	2.30734353	2.08291333	0.29295603	7.10998628	1.1605E-12	4.254E-12
CASP4	ENSG00000196954	protein_coding	3257.59445	1.24680545	0.06803909	18.3248398	5.2436E-75	3.0465E-73

FUT11	ENSG00000196968	protein_coding	3534.9602	1.54951066	0.06939815	22.3278385	1.983E-110	4.267E-108
ANXA4	ENSG00000196975	protein_coding	41164.2567	1.83977888	0.09336548	19.7051303	1.9484E-86	1.7209E-84
DTHD1	ENSG00000197057	protein_coding	70.1146332	3.22509343	0.20134769	16.0175339	9.6398E-58	3.0329E-56
H4C3	ENSG00000197061	protein_coding	8.62008431	1.32033809	0.26011505	5.07597737	3.8551E-07	9.1637E-07
GAL3ST4	ENSG00000197093	protein_coding	966.559043	2.88098267	0.12332995	23.3599598	1.092E-120	3.089E-118
ADAM32	ENSG00000197140	protein_coding	55.6167669	1.37978694	0.16671484	8.27632926	1.2703E-16	6.0595E-16
H3C12	ENSG00000197153	protein_coding	3.04822094	2.45391674	0.33551451	7.31389156	2.5951E-13	9.9327E-13
MAGEA6	ENSG00000197172	protein_coding	1.17064451	2.10527253	0.63478375	3.31651928	0.00091146	0.00154492
SLC22A4	ENSG00000197208	protein_coding	822.96663	1.0237717	0.13122892	7.80141808	6.1215E-15	2.6177E-14
H4C11	ENSG00000197238	protein_coding	4.83883148	2.22575917	0.23351023	9.53174159	1.5467E-21	9.8187E-21
SLC2A7	ENSG00000197241	protein_coding	7.4923589	2.29058181	0.21188556	10.8104668	3.071E-27	2.6552E-26
SERPINA1	ENSG00000197249	protein_coding	91033.789	1.70022576	0.18334036	9.27360334	1.7996E-20	1.078E-19
TPSB2	ENSG00000197253	protein_coding	518.015005	1.67819064	0.19446675	8.6297047	6.1511E-18	3.1818E-17
C6orf141	ENSG00000197261	protein_coding	74.9969796	2.12341933	0.25478696	8.33409723	7.8092E-17	3.773E-16
IL27	ENSG00000197272	protein_coding	10.8586347	2.44160043	0.18629904	13.1058131	3.0498E-39	4.6247E-38
RAD54B	ENSG00000197275	protein_coding	159.797757	1.07278968	0.12790546	8.38736394	4.9716E-17	2.4328E-16
BLM	ENSG00000197299	protein_coding	146.442823	1.24430149	0.10789964	11.5320266	9.0977E-31	9.3195E-30
LYPD2	ENSG00000197353	protein_coding	4.48278734	1.64637822	0.22727378	7.24403057	4.3554E-13	1.6403E-12
C5AR1	ENSG00000197405	protein_coding	1486.68547	1.36512336	0.11683102	11.6845973	1.528E-31	1.6324E-30
H3C4	ENSG00000197409	protein_coding	31.9800175	1.55756764	0.20308904	7.6693831	1.7283E-14	7.1619E-14
DCHS2	ENSG00000197410	protein_coding	170.051388	1.50319298	0.20140826	7.46341263	8.431E-14	3.3407E-13
FABP12	ENSG00000197416	protein_coding	0.31424105	1.00015029	0.46248191	2.16257168	0.03057414	0.04266901
OPALIN	ENSG00000197430	protein_coding	1.14907677	2.45529837	0.57993024	4.23378229	2.2979E-05	4.621E-05
CYP2F1	ENSG00000197446	protein_coding	1.92606903	2.1111794	0.35646448	5.92255188	3.1698E-09	8.9802E-09
STMN3	ENSG00000197457	protein_coding	4101.75791	2.14010246	0.14355048	14.9083617	2.908E-50	6.9757E-49

SPN	ENSG00000197471	protein_coding	1162.94561	2.11876516	0.13022073	16.270567	1.5967E-59	5.3311E-58
GALP	ENSG00000197487	protein_coding	0.87273117	2.42002789	0.56199496	4.30613809	1.6613E-05	3.3835E-05
SLC28A3	ENSG00000197506	protein_coding	21.9647848	1.41251308	0.21321139	6.62494183	3.4738E-11	1.1456E-10
FAM177B	ENSG00000197520	protein_coding	59.2665836	1.90040837	0.15408574	12.3334473	5.9831E-35	7.504E-34
GZMM	ENSG00000197540	protein_coding	127.82915	2.07370318	0.13622172	15.2229994	2.4886E-52	6.478E-51
HOXA4	ENSG00000197576	protein_coding	549.816893	1.2321964	0.10607992	11.6157366	3.4282E-31	3.5962E-30
DMBX1	ENSG00000197587	protein_coding	5.17356777	3.50522113	0.42590212	8.23010959	1.8704E-16	8.8103E-16
CCDC154	ENSG00000197599	protein_coding	52.2809062	2.29340935	0.19868032	11.5432135	7.9884E-31	8.2088E-30
ZNF841	ENSG00000197608	protein_coding	698.132179	1.07446081	0.09443801	11.3774187	5.418E-30	5.3619E-29
MPEG1	ENSG00000197629	protein_coding	3587.22059	1.29778641	0.12151277	10.6802471	1.2594E-26	1.0556E-25
SERPINB2	ENSG00000197632	protein_coding	5.15508839	1.01069433	0.35504892	2.84663398	0.00441841	0.00692815
SERPINB13	ENSG00000197641	protein_coding	1.38964124	2.59919328	0.45619987	5.69748806	1.2159E-08	3.2796E-08
PDCD1LG2	ENSG00000197646	protein_coding	285.922263	1.08841795	0.12338945	8.82099659	1.1345E-18	6.1362E-18
OR51C1P	ENSG00000197674	protein_coding	0.82850741	2.24863356	0.37621713	5.97695687	2.2734E-09	6.525E-09
NMB	ENSG00000197696	protein_coding	1427.87581	2.56735186	0.16741389	15.3353578	4.4382E-53	1.1822E-51
LHFPL5	ENSG00000197753	protein_coding	6.42216991	3.33991918	0.34524752	9.67398448	3.8893E-22	2.5485E-21
CFD	ENSG00000197766	protein_coding	884.423302	1.2635716	0.16088947	7.85366225	4.0406E-15	1.7484E-14
STPG3	ENSG00000197768	protein_coding	58.7259926	1.74151962	0.22595036	7.70753196	1.2827E-14	5.3627E-14
EME2	ENSG00000197774	protein_coding	736.103899	1.88815275	0.14552732	12.9745583	1.7058E-38	2.4974E-37
CCDC180	ENSG00000197816	protein_coding	101.45329	1.31712347	0.15400437	8.55250708	1.2044E-17	6.1157E-17
CYP2A13	ENSG00000197838	protein_coding	0.90199228	2.19262337	0.48445129	4.52599349	6.0112E-06	1.2784E-05
CYRIA	ENSG00000197872	protein_coding	1123.56168	1.55059763	0.0924113	16.7793081	3.4584E-63	1.2963E-61
UGT2B17	ENSG00000197888	protein_coding	19.995652	1.8262551	0.26845202	6.80291063	1.0253E-11	3.5056E-11
H2BC12	ENSG00000197903	protein_coding	684.628199	1.10689761	0.13722103	8.06653017	7.2324E-16	3.2842E-15
HES5	ENSG00000197921	protein_coding	20.5505967	3.82759424	0.24513133	15.6144634	5.8031E-55	1.6492E-53

ERO1A	ENSG00000197930	protein_coding	7496.52308	1.4789066	0.08814337	16.7784214	3.5104E-63	1.3147E-61
FCHSD1	ENSG00000197948	protein_coding	932.196684	1.2979043	0.10015012	12.9595881	2.0736E-38	3.0224E-37
ELOVL2	ENSG00000197977	protein_coding	289.254783	3.20264703	0.18794814	17.0400569	4.1433E-65	1.6675E-63
GOLGA6L9	ENSG00000197978	protein_coding	114.297212	1.32380059	0.13221055	10.0128214	1.3388E-23	9.4753E-23
CLEC9A	ENSG00000197992	protein_coding	101.289231	1.41995879	0.16068397	8.83696608	9.8353E-19	5.3413E-18
FCGR1B	ENSG00000198019	protein_coding	88.9715272	2.85055308	0.14155405	20.1375595	3.4597E-90	3.4933E-88
TUBA3C	ENSG00000198033	protein_coding	6.34368349	4.69883896	0.46780998	10.0443326	9.7304E-24	6.9381E-23
AVPR1B	ENSG00000198049	protein_coding	205.766483	3.71986702	0.20193539	18.4210751	8.9018E-76	5.3272E-74
SIRPA	ENSG00000198053	protein_coding	12229.3636	1.52553041	0.09643449	15.8193443	2.2886E-56	6.7993E-55
NPIPB13	ENSG00000198064	protein_coding	14.9199433	3.12632313	0.24496799	12.7621702	2.6667E-37	3.7349E-36
AKR1B10	ENSG00000198074	protein_coding	186.446924	1.03234349	0.33786085	3.05552863	0.00224664	0.00364315
CYP2A7	ENSG00000198077	protein_coding	1.61706521	1.84485024	0.33172157	5.56144182	2.6755E-08	7.0094E-08
TMPRSS11F	ENSG00000198092	protein_coding	1.91610474	2.36302164	0.36987068	6.38877792	1.6722E-10	5.2345E-10
CHSY3	ENSG00000198108	protein_coding	312.317966	2.19844018	0.11922927	18.4387621	6.4194E-76	3.8873E-74
MB	ENSG00000198125	protein_coding	28.4456788	1.11275195	0.19841596	5.60817756	2.0447E-08	5.4129E-08
NPIPB6	ENSG00000198156	protein_coding	6.49877197	1.2288402	0.21957771	5.5963796	2.1887E-08	5.7766E-08
RPS6KL1	ENSG00000198208	protein_coding	290.833635	1.16930823	0.13261046	8.81761694	1.1692E-18	6.319E-18
AC092143.1	ENSG00000198211	protein_coding	0.44637114	1.17917039	0.45818832	2.57354965	0.01006612	0.01505615
CSF2RA	ENSG00000198223	protein_coding	728.031883	1.92548147	0.11492969	16.75356	5.3337E-63	1.983E-61
CARD11	ENSG00000198286	protein_coding	998.798989	2.10460597	0.16324685	12.892169	4.9823E-38	7.1799E-37
TMEM239	ENSG00000198326	protein_coding	0.78386157	2.02075953	0.43052951	4.69366092	2.6836E-06	5.8996E-06
MYL4	ENSG00000198336	protein_coding	44.8337504	1.5760483	0.11777801	13.3815162	7.7549E-41	1.2509E-39
NRARP	ENSG00000198435	protein_coding	753.07392	1.6260581	0.12239243	13.2856097	2.8056E-40	4.4273E-39
OR14L1P	ENSG00000198452	protein_coding	0.56874232	1.18108105	0.31418711	3.75916463	0.00017048	0.00031305
RTP2	ENSG00000198471	protein_coding	18.2121524	5.27813556	0.33624077	15.6974884	1.5735E-55	4.5595E-54

HLA-DRB5	ENSG00000198502	protein_coding	14182.4734	1.34920672	0.13674325	9.86671507	5.8034E-23	3.9653E-22
C20orf203	ENSG00000198547	protein_coding	14.0538589	2.95459912	0.24348155	12.1347966	6.9081E-34	8.2554E-33
DDX39B	ENSG00000198563	protein_coding	4150.90479	1.56825396	0.12039284	13.0261402	8.6893E-39	1.287E-37
SH2D1B	ENSG00000198574	protein_coding	84.3297516	1.21552194	0.1230608	9.87741002	5.2164E-23	3.5733E-22
MMP17	ENSG00000198598	protein_coding	87.3722483	2.03110783	0.22013969	9.22644985	2.7975E-20	1.6551E-19
BAZ1A	ENSG00000198604	protein_coding	1996.58957	1.18297804	0.07117323	16.6211083	4.9018E-62	1.7652E-60
RYR2	ENSG00000198626	protein_coding	230.771629	1.57437438	0.23543879	6.6869795	2.2782E-11	7.6148E-11
ANKRD13B	ENSG00000198720	protein_coding	304.881499	1.43802622	0.12080419	11.9037773	1.1311E-32	1.2718E-31
PLXNB3	ENSG00000198753	protein_coding	185.128338	2.01558603	0.19499553	10.3365754	4.8144E-25	3.6875E-24
EPS8L3	ENSG00000198758	protein_coding	281.68541	3.56644569	0.32932395	10.8295972	2.4925E-27	2.1652E-26
RCSD1	ENSG00000198771	protein_coding	1865.67398	1.22398579	0.08790822	13.9234513	4.5633E-44	8.469E-43
MUC2	ENSG00000198788	protein_coding	2.97688212	2.44929999	0.32941427	7.4353184	1.0432E-13	4.1103E-13
ALPK2	ENSG00000198796	protein_coding	4740.86993	1.94007319	0.15339495	12.647569	1.1539E-36	1.5724E-35
BRINP2	ENSG00000198797	protein_coding	9.81034492	1.11791608	0.26915807	4.15338119	3.276E-05	6.491E-05
LRRC10	ENSG00000198812	protein_coding	0.60770383	1.38735655	0.40369807	3.43661926	0.00058902	0.00101959
FOXJ3	ENSG00000198815	protein_coding	9421.40084	1.6332083	0.12277553	13.3023928	2.2418E-40	3.5534E-39
CD247	ENSG00000198821	protein_coding	636.168557	2.57107175	0.1288241	19.9580029	1.2772E-88	1.2146E-86
ARHGAP11A	ENSG00000198826	protein_coding	396.638351	1.38473627	0.10805795	12.8147559	1.3556E-37	1.9228E-36
GJC2	ENSG00000198835	protein_coding	189.461275	1.36007128	0.15295092	8.89220741	5.9907E-19	3.2963E-18
CD3E	ENSG00000198851	protein_coding	1651.3285	2.63661297	0.16269769	16.2055956	4.604E-59	1.5151E-57
GRK5	ENSG00000198873	protein_coding	1584.39417	1.24875159	0.08572926	14.5662238	4.6066E-48	1.007E-46
ITPRIPL1	ENSG00000198885	protein_coding	142.274151	1.77972052	0.09821785	18.1201333	2.2106E-73	1.2064E-71
PRC1	ENSG00000198901	protein_coding	741.510977	1.34525757	0.09317813	14.4374822	3.0062E-47	6.3477E-46
RASGEF1A	ENSG00000198915	protein_coding	526.668721	1.04629381	0.16014067	6.53359197	6.4211E-11	2.0742E-10
CSAG1	ENSG00000198930	protein_coding	7.80843595	3.56168868	0.29181802	12.2051704	2.917E-34	3.5431E-33

TGM2	ENSG00000198959	protein_coding	29419.7917	1.2663744	0.13176218	9.6110611	7.1805E-22	4.6439E-21
INF2	ENSG00000203485	protein_coding	8615.99914	1.28777597	0.09194957	14.0052417	1.4479E-44	2.7347E-43
METTL11B	ENSG00000203740	protein_coding	0.86407708	1.54224364	0.43409228	3.55280133	0.00038115	0.0006736
FCGR3A	ENSG00000203747	protein_coding	8049.29853	3.32135225	0.14265015	23.2832015	6.561E-120	1.812E-117
LORICRIN	ENSG00000203782	protein_coding	0.46918063	1.01213171	0.39524863	2.56074692	0.01044474	0.01559093
SPRR2E	ENSG00000203785	protein_coding	1.13238491	1.8815653	0.71339165	2.63749273	0.00835214	0.01264475
H3C14	ENSG00000203811	protein_coding	0.84423962	1.31381547	0.55869209	2.3515913	0.0186933	0.02691669
PNLIPRP3	ENSG00000203837	protein_coding	2.89622088	1.83977455	0.67344869	2.7318704	0.00629759	0.00968979
SMIM9	ENSG00000203870	protein_coding	3.39514759	3.26878883	0.32757616	9.97871411	1.889E-23	1.3253E-22
C6orf163	ENSG00000203872	protein_coding	44.8413122	1.06675969	0.13805695	7.72695393	1.1015E-14	4.6316E-14
OOEP	ENSG00000203907	protein_coding	16.5569698	1.21242398	0.32686438	3.70925698	0.00020787	0.00037815
KHDC3L	ENSG00000203908	protein_coding	1.08025905	1.95862633	0.38033876	5.14968895	2.6092E-07	6.2965E-07
C1orf146	ENSG00000203910	protein_coding	3.18949501	1.4130265	0.24210188	5.83649527	5.331E-09	1.4821E-08
GLT6D1	ENSG00000204007	protein_coding	0.70909513	2.0845039	0.4954853	4.20699442	2.5879E-05	5.1786E-05
IFIT1B	ENSG00000204010	protein_coding	5.75852142	1.32365875	0.25380865	5.21518375	1.8364E-07	4.4913E-07
LIPN	ENSG00000204020	protein_coding	6.88776244	1.57912728	0.1982107	7.96691244	1.6269E-15	7.2384E-15
LRIT2	ENSG00000204033	protein_coding	7.77145973	4.12323147	0.37414279	11.0204754	3.0445E-28	2.7669E-27
RPA4	ENSG00000204086	protein_coding	7.43245598	2.85239611	0.22523516	12.6640799	9.351E-37	1.2792E-35
CLPSL1	ENSG00000204140	protein_coding	1.50847197	1.66342464	0.51354526	3.23910036	0.00119907	0.00200638
AGAP6	ENSG00000204149	protein_coding	288.176056	1.59373825	0.13507511	11.7989041	3.9543E-32	4.3376E-31
TMEM273	ENSG00000204161	protein_coding	813.16172	1.320735	0.10787038	12.2437225	1.8151E-34	2.223E-33
CXorf65	ENSG00000204165	protein_coding	20.9436692	3.44328076	0.22579685	15.2494631	1.6599E-52	4.3481E-51
AGAP9	ENSG00000204172	protein_coding	236.499509	1.21899978	0.14736991	8.2717004	1.3206E-16	6.2923E-16
NPY4R	ENSG00000204174	protein_coding	3.42722803	2.80784498	0.34989093	8.02491514	1.016E-15	4.5727E-15
AWAT1	ENSG00000204195	protein_coding	1.70553551	1.52746652	0.33321754	4.58399192	4.5618E-06	9.8196E-06

COL11A2	ENSG00000204248	protein_coding	102.564538	1.09885347	0.1531049	7.17712796	7.1191E-13	2.647E-12
HLA-DOA	ENSG00000204252	protein_coding	4817.97941	1.38052639	0.13658392	10.1075324	5.1157E-24	3.7053E-23
HLA-DMA	ENSG00000204257	protein_coding	7807.73509	1.48674406	0.09539216	15.5856005	9.1202E-55	2.5744E-53
COL5A2	ENSG00000204262	protein_coding	6301.63385	2.0572066	0.13536543	15.1974292	3.6777E-52	9.5411E-51
PSMB8	ENSG00000204264	protein_coding	7789.44815	1.62342889	0.07825257	20.7460126	1.3319E-95	1.6913E-93
TAP2	ENSG00000204267	protein_coding	2433.28459	1.13834964	0.07664828	14.8516013	6.7926E-50	1.6023E-48
TMEM235	ENSG00000204278	protein_coding	1.16472645	1.64510238	0.47101146	3.49270136	0.00047816	0.00083627
HLA-DRA	ENSG00000204287	protein_coding	77220.4688	1.71365769	0.11222242	15.2701907	1.2082E-52	3.183E-51
COL15A1	ENSG00000204291	protein_coding	7882.94757	1.40671948	0.14216146	9.89522415	4.3663E-23	3.0067E-22
TMEM225	ENSG00000204300	protein_coding	0.92662469	2.47614751	0.49275884	5.02506964	5.0325E-07	1.1842E-06
NOTCH4	ENSG00000204301	protein_coding	6899.69324	1.56075589	0.11440995	13.641785	2.2596E-42	3.8956E-41
AGER	ENSG00000204305	protein_coding	342.804743	2.07397382	0.1399742	14.8168292	1.1404E-49	2.6645E-48
CD300LD	ENSG00000204345	protein_coding	0.97139405	1.73142373	0.37675599	4.59561031	4.3148E-06	9.3072E-06
BTBD17	ENSG00000204347	protein_coding	0.60457435	1.41358427	0.32580494	4.33874415	1.433E-05	2.9389E-05
LAYN	ENSG00000204381	protein_coding	956.810384	1.24930295	0.10713693	11.6608058	2.0212E-31	2.1458E-30
CARD16	ENSG00000204397	protein_coding	560.006963	1.92772714	0.10109184	19.069067	4.5637E-81	3.3102E-79
MSH5	ENSG00000204410	protein_coding	241.897934	2.17258569	0.151322	14.357368	9.5797E-47	1.9817E-45
LY6G6C	ENSG00000204421	protein_coding	3.04471669	1.80315902	0.2798164	6.4440792	1.163E-10	3.6835E-10
AIF1	ENSG00000204472	protein_coding	2131.87638	1.03997643	0.11182642	9.29991661	1.4056E-20	8.4768E-20
NCR3	ENSG00000204475	protein_coding	46.7580827	1.82610335	0.13604438	13.4228498	4.4429E-41	7.2428E-40
PRAMEF17	ENSG00000204479	protein_coding	0.63401724	1.99867887	0.57128612	3.49856018	0.00046778	0.00081895
LST1	ENSG00000204482	protein_coding	1037.10777	2.24714592	0.11638125	19.3084873	4.5573E-83	3.4935E-81
MICB	ENSG00000204516	protein_coding	351.477681	1.29019104	0.08703476	14.8238597	1.0271E-49	2.4046E-48
HLA-C	ENSG00000204525	protein_coding	103326.721	1.33235694	0.08145362	16.3572469	3.862E-60	1.3163E-58
POU5F1	ENSG00000204531	protein_coding	1816.8709	3.4005418	0.15634374	21.7504182	6.845E-105	1.255E-102

PSORS1C2	ENSG00000204538	protein_coding	22.8036372	3.73106503	0.21966591	16.9851797	1.0573E-64	4.2036E-63
CDSN	ENSG00000204539	protein_coding	4.67320637	2.95434671	0.28135223	10.5005271	8.5899E-26	6.8805E-25
PSORS1C1	ENSG00000204540	protein_coding	358.154517	2.95520928	0.13448225	21.9747155	5.027E-107	9.764E-105
C6orf15	ENSG00000204542	protein_coding	0.79944537	1.18370421	0.40919868	2.8927371	0.00381901	0.00603597
KRTAP5-11	ENSG00000204571	protein_coding	1.25495231	2.11075794	0.35096923	6.01408269	1.8091E-09	5.2315E-09
KRTAP5-10	ENSG00000204572	protein_coding	16.3827277	2.73440746	0.21288025	12.8448151	9.1971E-38	1.3106E-36
LILRB3	ENSG00000204577	protein_coding	205.487965	2.44969051	0.12397001	19.7603471	6.5354E-87	5.8399E-85
HLA-E	ENSG00000204592	protein_coding	78840.5719	1.05288576	0.0651945	16.1499171	1.1371E-58	3.6892E-57
DPRX	ENSG00000204595	protein_coding	0.69852616	2.00649733	0.4596897	4.36489515	1.2718E-05	2.6197E-05
TRIM15	ENSG00000204610	protein_coding	637.965772	1.21239319	0.18957439	6.39534263	1.6019E-10	5.0244E-10
FOXB2	ENSG00000204612	protein_coding	1.16954006	2.68356155	0.45047435	5.95719063	2.5661E-09	7.3264E-09
TRIM31	ENSG00000204616	protein_coding	18.8701004	1.02049095	0.16746708	6.0936809	1.1034E-09	3.2458E-09
DISP3	ENSG00000204624	protein_coding	27.0690087	1.61493633	0.19409281	8.32043348	8.7642E-17	4.2224E-16
RACK1	ENSG00000204628	protein_coding	90066.973	1.20005594	0.0763069	15.7267038	9.9245E-56	2.8941E-54
HLA-G	ENSG00000204632	protein_coding	2220.20637	3.84504325	0.20292011	18.9485571	4.5386E-80	3.1861E-78
HLA-F	ENSG00000204642	protein_coding	8458.29631	2.44095256	0.10256233	23.7996996	3.364E-125	1.101E-122
MOG	ENSG00000204655	protein_coding	9.18066651	3.37925676	0.45985416	7.34854009	2.0038E-13	7.7341E-13
OR2H2	ENSG00000204657	protein_coding	1.28164179	2.03423544	0.36426605	5.5844772	2.344E-08	6.1705E-08
C9orf57	ENSG00000204669	protein_coding	1.42575952	2.28728525	0.41087815	5.56682127	2.5943E-08	6.8019E-08
GABBR1	ENSG00000204681	protein_coding	1146.35773	1.12315166	0.13732972	8.17850409	2.8739E-16	1.3379E-15
OR2H1	ENSG00000204688	protein_coding	2.62303426	3.05162582	0.47415016	6.43599022	1.2267E-10	3.8798E-10
OR11A1	ENSG00000204694	protein_coding	1.31490529	2.48562637	0.46201565	5.37996146	7.4502E-08	1.8853E-07
INSYN2B	ENSG00000204767	protein_coding	146.23921	1.09330156	0.22018799	4.96530969	6.8592E-07	1.5948E-06
FOXD4L5	ENSG00000204779	protein_coding	0.86754992	1.14452362	0.33163159	3.45119002	0.00055812	0.00096886
IGFL2	ENSG00000204866	protein_coding	21.5097425	4.28196553	0.32456859	13.1927908	9.6544E-40	1.4886E-38

GPR20	ENSG00000204882	protein_coding	56.9071691	1.27298625	0.16641512	7.64946282	2.0182E-14	8.3288E-14
KRT25	ENSG00000204897	protein_coding	11.4307444	4.64908594	0.44343628	10.4842255	1.0208E-25	8.1437E-25
PCDHGA1	ENSG00000204956	protein_coding	117.854119	1.33282752	0.15493116	8.60270771	7.7857E-18	4.0007E-17
PCDHA9	ENSG00000204961	protein_coding	12.3089468	1.0146334	0.18040003	5.62435275	1.862E-08	4.9483E-08
MS4A13	ENSG00000204979	protein_coding	0.65437842	1.43623149	0.61110443	2.35022269	0.01876218	0.02700993
PABPN1L	ENSG00000205022	protein_coding	0.96120917	2.02284248	0.35009012	5.77806216	7.5566E-09	2.0706E-08
SLFN12L	ENSG00000205045	protein_coding	138.940584	2.68034139	0.15885169	16.8732317	7.0814E-64	2.7142E-62
SYCE1L	ENSG00000205078	protein_coding	118.928343	1.5787879	0.17478984	9.0324922	1.6781E-19	9.5274E-19
FAM71F2	ENSG00000205085	protein_coding	104.933999	1.26236941	0.16178928	7.80255266	6.0667E-15	2.5955E-14
ACCSL	ENSG00000205126	protein_coding	1.85506462	1.80784268	0.30814267	5.86690152	4.4401E-09	1.2428E-08
C4orf47	ENSG00000205129	protein_coding	373.640355	2.51077465	0.12451462	20.1644963	2.0078E-90	2.0408E-88
ARID3C	ENSG00000205143	protein_coding	12.6128546	3.00357326	0.24057971	12.4847323	9.0447E-36	1.1799E-34
PSMB10	ENSG00000205220	protein_coding	1906.90854	1.37911004	0.0974823	14.1472868	1.941E-45	3.796E-44
AC105052.1	ENSG00000205236	protein_coding	0.41962537	1.02818692	0.32224539	3.19069548	0.00141931	0.00235503
SPDYE2	ENSG00000205238	protein_coding	14.1147679	1.38781955	0.17432058	7.96130617	1.7023E-15	7.5617E-15
PDE7A	ENSG00000205268	protein_coding	2363.58496	1.14083458	0.10043911	11.3584697	6.7313E-30	6.6388E-29
MUC12	ENSG00000205277	protein_coding	151.328439	4.3308127	0.23503024	18.4266189	8.035E-76	4.8211E-74
SAP25	ENSG00000205307	protein_coding	9.85080945	2.35932525	0.22745293	10.3728066	3.297E-25	2.5538E-24
KRT6A	ENSG00000205420	protein_coding	24.1591169	4.31841524	0.47314719	9.12700183	7.0423E-20	4.0878E-19
KRT81	ENSG00000205426	protein_coding	37.404213	3.34293744	0.23579503	14.1773024	1.2662E-45	2.503E-44
EXOC3L4	ENSG00000205436	protein_coding	725.204289	2.03379842	0.15781085	12.8875704	5.2884E-38	7.6067E-37
IZUMO3	ENSG00000205442	protein_coding	0.50491969	1.70194826	0.53162544	3.20140486	0.00136759	0.002274
CPT1B	ENSG00000205560	protein_coding	146.009969	1.84773738	0.17732146	10.4202694	2.0038E-25	1.5721E-24
C17orf107	ENSG00000205710	protein_coding	425.864165	1.12777524	0.12007109	9.39256268	5.8558E-21	3.5983E-20
CRLF2	ENSG00000205755	protein_coding	10.2883454	2.33324474	0.22189237	10.5152097	7.3517E-26	5.9052E-25

ARRDC5	ENSG00000205784	protein_coding	29.1832366	3.76723265	0.2086832	18.0524001	7.5536E-73	4.0404E-71
KLRC2	ENSG00000205809	protein_coding	7.36850969	2.51748887	0.2376722	10.5922733	3.2364E-26	2.6474E-25
KLRC3	ENSG00000205810	protein_coding	2.98075522	2.31832592	0.26043112	8.90187753	5.491E-19	3.0258E-18
C22orf42	ENSG00000205856	protein_coding	1.30242908	2.2233911	0.31117384	7.14517367	8.9882E-13	3.3188E-12
NANOGNB	ENSG00000205857	protein_coding	0.51851467	1.12909177	0.48089129	2.34791479	0.01887884	0.02716763
C1QTNF9B	ENSG00000205863	protein_coding	5.84572614	1.14875971	0.19572985	5.86910835	4.3815E-09	1.2268E-08
KRTAP5-1	ENSG00000205869	protein_coding	8.32536301	1.16346297	0.24107702	4.82610485	1.3923E-06	3.1425E-06
DEFB134	ENSG00000205882	protein_coding	0.4502505	1.18567549	0.48394085	2.45004215	0.01428395	0.02091314
ONECUT3	ENSG00000205922	protein_coding	5.25769443	2.42350892	0.29359687	8.25454613	1.5248E-16	7.2351E-16
OLIG2	ENSG00000205927	protein_coding	33.7147634	2.81370034	0.38516061	7.30526503	2.7672E-13	1.0567E-12
IFITM5	ENSG00000206013	protein_coding	4.81122759	3.80864687	0.41629593	9.14889281	5.752E-20	3.3547E-19
C18orf63	ENSG00000206043	protein_coding	9.02055171	1.93765111	0.23362489	8.29385553	1.0964E-16	5.2516E-16
TMEM211	ENSG00000206069	protein_coding	5.75183876	1.01755222	0.20959125	4.8549366	1.2043E-06	2.7341E-06
ZDHHC11B	ENSG00000206077	protein_coding	265.938561	1.6231401	0.19585954	8.28726578	1.1588E-16	5.5415E-16
HBM	ENSG00000206177	protein_coding	3.99280952	2.20748986	0.42174687	5.23415826	1.6574E-07	4.0672E-07
ANKUB1	ENSG00000206199	protein_coding	4.44397287	1.54218077	0.24596909	6.26981522	3.6148E-10	1.1051E-09
FOXL2NB	ENSG00000206262	protein_coding	3.00044719	3.05834147	0.39406807	7.76094716	8.4297E-15	3.5708E-14
HLA-A	ENSG00000206503	protein_coding	121338.753	1.69065034	0.08334932	20.283912	1.7837E-91	1.8883E-89
CD200R1L	ENSG00000206531	protein_coding	0.70591054	1.77238249	0.32456523	5.4607898	4.7402E-08	1.2173E-07
AC109583.1	ENSG00000206549	protein_coding	33.266955	1.67350978	0.20327715	8.23265067	1.8312E-16	8.6316E-16
TSSK1B	ENSG00000212122	protein_coding	0.60997883	1.22906603	0.57535507	2.13618701	0.03266417	0.04540201
PRR22	ENSG00000212123	protein_coding	124.128871	1.41421543	0.13093687	10.8007423	3.4143E-27	2.9431E-26
TAS2R19	ENSG00000212124	protein_coding	12.7284013	1.59387608	0.18196877	8.75906381	1.9688E-18	1.0502E-17
TAS2R50	ENSG00000212126	protein_coding	1.96622412	1.48702619	0.29015529	5.12493213	2.9764E-07	7.1466E-07
TAS2R13	ENSG00000212128	protein_coding	1.74627965	1.46731604	0.3125849	4.694136	2.6774E-06	5.8867E-06

KRTAP16-1	ENSG00000212657	protein_coding	1.94866162	2.19180544	0.36524464	6.00092436	1.962E-09	5.6576E-09
KRTAP29-1	ENSG00000212658	protein_coding	0.88155146	2.21607621	0.3391708	6.53380603	6.4119E-11	2.0714E-10
CTAGE1	ENSG00000212710	protein_coding	1.64595702	1.13171682	0.26701222	4.23844577	2.2507E-05	4.5296E-05
CEP43	ENSG00000213066	protein_coding	1713.24416	1.35620094	0.10015024	13.541665	8.8754E-42	1.4871E-40
CRYGS	ENSG00000213139	protein_coding	146.826151	2.10213802	0.14343844	14.6553328	1.2454E-48	2.7904E-47
CRIP1	ENSG00000213145	protein_coding	179.450598	1.88646613	0.13920059	13.5521414	7.6952E-42	1.2931E-40
TRIM59	ENSG00000213186	protein_coding	374.146682	1.16292236	0.07806654	14.8965538	3.4702E-50	8.2767E-49
ASIC3	ENSG00000213199	protein_coding	65.4988123	1.99617632	0.16556829	12.0565133	1.7921E-33	2.0952E-32
LTC4S	ENSG00000213316	protein_coding	3.7675551	1.09940847	0.18592916	5.91305025	3.3583E-09	9.4906E-09
QTRT1	ENSG00000213339	protein_coding	2275.30406	1.17082655	0.09719878	12.0456922	2.0435E-33	2.3855E-32
MXD3	ENSG00000213347	protein_coding	268.096446	2.54872503	0.1373865	18.5514954	7.9314E-77	5.0081E-75
HAUS7	ENSG00000213397	protein_coding	347.752854	1.8075304	0.16168858	11.1790854	5.1619E-29	4.889E-28
LCAT	ENSG00000213398	protein_coding	656.769653	1.78440208	0.12867573	13.867433	9.9784E-44	1.826E-42
MAGEA12	ENSG00000213401	protein_coding	3.14505158	2.30899264	0.30796504	7.49758047	6.5007E-14	2.5985E-13
PVRIG	ENSG00000213413	protein_coding	18.1385157	1.58712827	0.16155031	9.82435917	8.8435E-23	5.9925E-22
GPC2	ENSG00000213420	protein_coding	32.5258382	1.97188797	0.15893339	12.4070091	2.3943E-35	3.0719E-34
SIPA1	ENSG00000213445	protein_coding	3124.79375	1.09638129	0.07461733	14.6933876	7.107E-49	1.6089E-47
GBP7	ENSG00000213512	protein_coding	25.7866576	1.15985338	0.21910184	5.29367242	1.1988E-07	2.9754E-07
GPSM3	ENSG00000213654	protein_coding	1821.93076	1.92373923	0.09606072	20.026283	3.2503E-89	3.1769E-87
LAT	ENSG00000213658	protein_coding	61.1132123	3.35733061	0.19033442	17.6391147	1.2338E-69	5.8609E-68
UGT2B11	ENSG00000213759	protein_coding	36.7785777	1.08133862	0.20215352	5.34909626	8.8395E-08	2.2201E-07
DDX47	ENSG00000213782	protein_coding	122.882933	1.62653209	0.11569916	14.0582879	6.8524E-45	1.3094E-43
KLRK1	ENSG00000213809	protein_coding	48.8905799	3.38475069	0.20277002	16.6925596	1.4847E-62	5.4403E-61
UBD	ENSG00000213886	protein_coding	1449.34256	3.16768817	0.188306	16.8220244	1.6831E-63	6.4135E-62
PPM1N	ENSG00000213889	protein_coding	58.1642848	1.13228554	0.14246373	7.94788649	1.8972E-15	8.4045E-15

CEACAM16	ENSG00000213892	protein_coding	2.43114849	1.76284337	0.35107161	5.0213213	5.1317E-07	1.2068E-06
LTB4R	ENSG00000213903	protein_coding	699.624408	2.52168803	0.12089154	20.8590937	1.2604E-96	1.6943E-94
LTB4R2	ENSG00000213906	protein_coding	131.142911	2.18469596	0.12068041	18.1031537	3.0093E-73	1.6364E-71
CSNK1E	ENSG00000213923	protein_coding	5896.52368	1.10220999	0.06435607	17.1267444	9.3757E-66	3.8477E-64
IRF9	ENSG00000213928	protein_coding	369.783178	1.68727064	0.13329634	12.6580417	1.0099E-36	1.3798E-35
HBG1	ENSG00000213934	protein_coding	1.56643463	2.74788923	0.79274385	3.46630152	0.00052767	0.00091823
AP1G2	ENSG00000213983	protein_coding	1542.48858	1.13705163	0.10683991	10.6425737	1.8884E-26	1.5679E-25
ZNF90	ENSG00000213988	protein_coding	229.577048	1.0434498	0.13611151	7.6661395	1.7725E-14	7.3406E-14
TM6SF2	ENSG00000213996	protein_coding	221.46852	1.13234966	0.20582533	5.50150775	3.7656E-08	9.7414E-08
MEF2B	ENSG00000213999	protein_coding	26.8935342	2.01403659	0.14471176	13.9175733	4.9544E-44	9.1801E-43
TTLL3	ENSG00000214021	protein_coding	986.78348	1.58712719	0.14094811	11.260365	2.059E-29	1.9892E-28
TSPAN4	ENSG00000214063	protein_coding	5405.1823	1.19421053	0.08117604	14.7113669	5.4495E-49	1.2374E-47
SMCO1	ENSG00000214097	protein_coding	0.94536774	1.59273418	0.31789305	5.01028308	5.435E-07	1.2756E-06
PRCD	ENSG00000214140	protein_coding	58.2676494	1.04157213	0.11518727	9.04242359	1.5324E-19	8.7217E-19
SH3D21	ENSG00000214193	protein_coding	878.92536	1.45388027	0.11668097	12.4603033	1.229E-35	1.5965E-34
C19orf38	ENSG00000214212	protein_coding	170.272027	1.24516163	0.10875693	11.4490328	2.3778E-30	2.3946E-29
MINDY4B	ENSG00000214237	protein_coding	3.36591423	1.21153544	0.20424853	5.9316728	2.9986E-09	8.5131E-09
ANG	ENSG00000214274	protein_coding	1256.23573	1.57802293	0.12293174	12.8365785	1.023E-37	1.455E-36
SCART1	ENSG00000214279	protein_coding	140.537087	2.22851074	0.17040595	13.0776583	4.4184E-39	6.6517E-38
NEURL1B	ENSG00000214357	protein_coding	3587.09283	1.12635977	0.10357662	10.8746524	1.5223E-27	1.3328E-26
GNAT3	ENSG00000214415	protein_coding	0.51212038	1.33454988	0.50800217	2.62705548	0.00861273	0.01300705
SPINK13	ENSG00000214510	protein_coding	362.754602	4.6370515	0.26904575	17.2351783	1.4461E-66	6.1152E-65
HIGD1C	ENSG00000214511	protein_coding	2.69154403	1.20348421	0.2326207	5.17359031	2.2964E-07	5.5659E-07
ZNF705E	ENSG00000214534	protein_coding	8.29705415	1.15560229	0.15468537	7.47066286	7.9792E-14	3.1691E-13
C10orf105	ENSG00000214688	protein_coding	9.23314514	1.5002826	0.15902723	9.43412422	3.9427E-21	2.4484E-20

MS4A18	ENSG00000214782	protein_coding	0.58284429	1.45494415	0.41605471	3.49700201	0.00047052	0.00082347
MS4A4E	ENSG00000214787	protein_coding	58.4871182	3.13114521	0.17790674	17.5999248	2.4665E-69	1.1538E-67
MTCP1	ENSG00000214827	protein_coding	491.425791	3.24908376	0.12727599	25.5278615	9.673E-144	5.279E-141
SMTNL1	ENSG00000214872	protein_coding	35.065143	2.94156598	0.2089761	14.0760883	5.3278E-45	1.0236E-43
SPATA31D1	ENSG00000214929	protein_coding	0.89036894	2.33453225	0.45307569	5.15263187	2.5686E-07	6.2027E-07
NPIPA8	ENSG00000214940	protein_coding	0.72951586	1.91515786	0.41042013	4.6663351	3.0662E-06	6.7041E-06
GPR33	ENSG00000214943	protein_coding	2.20803644	1.22082794	0.26802993	4.5548194	5.2431E-06	1.1211E-05
TBC1D26	ENSG00000214946	protein_coding	2.91748955	3.22596328	0.33344436	9.67466737	3.8635E-22	2.5322E-21
NPIPA7	ENSG00000214967	protein_coding	0.4165058	1.20643373	0.4905875	2.45916118	0.01392621	0.0204148
GSG1L2	ENSG00000214978	protein_coding	161.218596	9.5628117	0.55359558	17.2740031	7.3838E-67	3.1634E-65
ACSM4	ENSG00000215009	protein_coding	11.392611	1.91864619	0.21194162	9.05271081	1.3946E-19	7.9566E-19
NLRP2B	ENSG00000215174	protein_coding	6.53790905	1.57297533	0.17875646	8.79954379	1.3737E-18	7.3999E-18
MUC5AC	ENSG00000215182	protein_coding	1.93489933	1.52902547	0.34362508	4.44969113	8.5994E-06	1.8004E-05
GOLGA6B	ENSG00000215186	protein_coding	0.99655143	2.08882619	0.3562009	5.86418005	4.5136E-09	1.2624E-08
GOLGA8B	ENSG00000215252	protein_coding	1041.08948	1.99950754	0.17918262	11.1590487	6.468E-29	6.0958E-28
KCNU1	ENSG00000215262	protein_coding	3.29071203	4.09009603	0.57393602	7.1263972	1.0303E-12	3.7869E-12
ZNF705G	ENSG00000215372	protein_coding	1.05297711	2.73245641	0.57486467	4.75321682	2.0021E-06	4.4528E-06
MYL5	ENSG00000215375	protein_coding	683.797755	1.20802456	0.0975218	12.3872254	3.0646E-35	3.8981E-34
SCRT2	ENSG00000215397	protein_coding	0.56846679	1.49710544	0.35279459	4.24356128	2.2E-05	4.4316E-05
NPEPL1	ENSG00000215440	protein_coding	1320.00824	1.49168168	0.1367079	10.9114517	1.0162E-27	9E-27
SKOR2	ENSG00000215474	protein_coding	0.50154614	1.32485587	0.44252621	2.99384725	0.00275484	0.00442576
EFCAB8	ENSG00000215529	protein_coding	10.6296833	2.54088566	0.25360003	10.019264	1.2543E-23	8.894E-23
C20orf202	ENSG00000215595	protein_coding	60.9796097	1.32790031	0.10043702	13.2212238	6.6178E-40	1.0256E-38
FAM72D	ENSG00000215784	protein_coding	9.35511113	1.78100201	0.17099996	10.4152191	2.1131E-25	1.6556E-24
TNFRSF25	ENSG00000215788	protein_coding	496.237933	1.83876529	0.14678607	12.5268376	5.3244E-36	7.0217E-35

RPTN	ENSG00000215853	protein_coding	4.48286577	4.06011461	0.51358294	7.90547018	2.6692E-15	1.1697E-14
C1orf167	ENSG00000215910	protein_coding	5.78642448	1.05526121	0.19861748	5.313033	1.0782E-07	2.6859E-07
TTC34	ENSG00000215912	protein_coding	124.460207	1.33097214	0.13587022	9.79590804	1.1724E-22	7.9035E-22
IFI30	ENSG00000216490	protein_coding	124.648758	2.30710403	0.156427	14.7487581	3.1338E-49	7.1972E-48
SP9	ENSG00000217236	protein_coding	0.67914071	1.67103649	0.70442784	2.37218972	0.01768301	0.0255591
CKLF	ENSG00000217555	protein_coding	366.244021	1.09173899	0.06764342	16.1396191	1.3437E-58	4.3377E-57
PAM16	ENSG00000217930	protein_coding	393.369322	1.03526139	0.0891002	11.6190688	3.2971E-31	3.4626E-30
CELA3B	ENSG00000219073	protein_coding	0.86515149	1.52988672	0.50014976	3.05885723	0.00222183	0.0036047
CATSPERZ	ENSG00000219435	protein_coding	3.0019188	1.81631884	0.28432473	6.38818461	1.6787E-10	5.2531E-10
TAFA5	ENSG00000219438	protein_coding	283.647564	1.36255141	0.16314286	8.35189125	6.7179E-17	3.2601E-16
PPP1R3G	ENSG00000219607	protein_coding	515.164449	2.12567387	0.10851005	19.5896493	1.8949E-85	1.6027E-83
LINGO3	ENSG00000220008	protein_coding	23.7067277	2.49122434	0.1687368	14.7639655	2.5013E-49	5.7619E-48
ZGLP1	ENSG00000220201	protein_coding	46.8626534	1.26810227	0.13362276	9.4901669	2.3066E-21	1.4511E-20
EBF2	ENSG00000221818	protein_coding	788.638386	2.76414539	0.15435419	17.9078097	1.0249E-71	5.2436E-70
PSG3	ENSG00000221826	protein_coding	1.95758155	2.9693352	0.50691131	5.85770158	4.6932E-09	1.3108E-08
MAGEA3	ENSG00000221867	protein_coding	3.71419281	4.0336622	0.62038729	6.50184536	7.9341E-11	2.5473E-10
PSG7	ENSG00000221878	protein_coding	0.76264897	1.93648795	0.52230513	3.70757982	0.00020925	0.00038054
FXYD7	ENSG00000221946	protein_coding	11.1128235	1.70830509	0.16590515	10.2968779	7.2785E-25	5.515E-24
XKR9	ENSG00000221947	protein_coding	85.2955645	1.10697012	0.16582704	6.67544996	2.4647E-11	8.219E-11
APOL6	ENSG00000221963	protein_coding	6793.81434	1.05380557	0.0909142	11.5912103	4.5663E-31	4.7596E-30
FADS3	ENSG00000221968	protein_coding	1818.63531	1.32123585	0.09728489	13.5811003	5.1842E-42	8.7693E-41
CCNL2	ENSG00000221978	protein_coding	5059.49667	1.79205597	0.13342055	13.431634	3.9461E-41	6.4466E-40
TIAF1	ENSG00000221995	protein_coding	165.850852	1.16718073	0.11604063	10.0583803	8.4375E-24	6.0397E-23
BTBD19	ENSG00000222009	protein_coding	673.927664	1.90294918	0.11931169	15.9493945	2.8767E-57	8.8863E-56
DCDC2B	ENSG00000222046	protein_coding	21.5129734	1.83725853	0.1801273	10.1997782	1.9873E-24	1.4757E-23

EBLN1	ENSG00000223601	protein_coding	0.69896977	1.66113439	0.35384141	4.69457323	2.6716E-06	5.8745E-06
ZNF735	ENSG00000223614	protein_coding	0.6096372	1.36399625	0.595333	2.2911484	0.02195483	0.03131496
RFPL4A	ENSG00000223638	protein_coding	4.12067877	1.20717582	0.24440035	4.9393376	7.8388E-07	1.8124E-06
CERS1	ENSG00000223802	protein_coding	26.3548313	1.7595129	0.24700988	7.12324918	1.0541E-12	3.8726E-12
HLA-DPB1	ENSG00000223865	protein_coding	27215.8529	2.05741096	0.11554392	17.8063104	6.3137E-71	3.1631E-69
C4B	ENSG00000224389	protein_coding	4377.34568	1.6902375	0.16374218	10.322554	5.5721E-25	4.2508E-24
NPIPA3	ENSG00000224712	protein_coding	16.1105396	2.45346671	0.24564038	9.98804311	1.7194E-23	1.2091E-22
PPP4R3C	ENSG00000224960	protein_coding	0.52502415	1.49639703	0.6746624	2.21799383	0.02655525	0.03742144
TMEM233	ENSG00000224982	protein_coding	428.673612	2.077944	0.12979943	16.0088837	1.1078E-57	3.4711E-56
PVALEF	ENSG00000225180	protein_coding	0.82084582	1.72003714	0.36281769	4.74077534	2.129E-06	4.723E-06
Z82206.1	ENSG00000225528	protein_coding	9.63705254	1.07445341	0.17680299	6.07712235	1.2236E-09	3.5843E-09
ZNF469	ENSG00000225614	protein_coding	310.245864	2.05553011	0.12162334	16.9007864	4.4395E-64	1.7203E-62
FAM229A	ENSG00000225828	protein_coding	148.978298	1.13230781	0.13583334	8.33600791	7.6841E-17	3.7161E-16
KIF4B	ENSG00000226650	protein_coding	5.55365023	2.39402175	0.24204463	9.89082793	4.5624E-23	3.1389E-22
TAS2R46	ENSG00000226761	protein_coding	1.58708698	1.61266176	0.33981164	4.74575201	2.0773E-06	4.6124E-06
LTA	ENSG00000226979	protein_coding	51.688481	2.63233641	0.17043838	15.4445053	8.2156E-54	2.2471E-52
AKR1B15	ENSG00000227471	protein_coding	7.41027712	1.47046138	0.39920225	3.68349972	0.00023005	0.00041642
LTB	ENSG00000227507	protein_coding	535.844328	1.09510403	0.16386621	6.68291555	2.3423E-11	7.8228E-11
RD3L	ENSG00000227729	protein_coding	0.56217505	1.29229495	0.57760268	2.23734236	0.02526397	0.03570825
ORM2	ENSG00000228278	protein_coding	10.0770103	3.43525834	0.3413261	10.0644467	7.9332E-24	5.685E-23
C2orf92	ENSG00000228486	protein_coding	156.281733	2.04204125	0.14018395	14.5668695	4.5633E-48	9.9897E-47
SAPCD1	ENSG00000228727	protein_coding	46.9456865	2.97110624	0.21278837	13.9627283	2.6316E-44	4.924E-43
PGA4	ENSG00000229183	protein_coding	1.17532989	2.37566496	0.43429323	5.47018648	4.4956E-08	1.156E-07
RFPL4AL1	ENSG00000229292	protein_coding	1.05665987	1.3961367	0.34287079	4.07190327	4.6631E-05	9.0934E-05
ORM1	ENSG00000229314	protein_coding	27.105561	3.41628443	0.39632222	8.61996702	6.6973E-18	3.4562E-17

SFTA3	ENSG00000229415	protein_coding	1.50402378	1.06074108	0.35986218	2.94763149	0.00320219	0.00510845
PATL2	ENSG00000229474	protein_coding	168.631252	2.83719127	0.1458691	19.4502554	2.8998E-84	2.3528E-82
PRAC2	ENSG00000229637	protein_coding	3.32543158	3.91041747	0.52395441	7.46327809	8.4396E-14	3.3438E-13
PGA3	ENSG00000229859	protein_coding	3.39702042	1.64680076	0.36547077	4.50597125	6.607E-06	1.3993E-05
TEX53	ENSG00000230054	protein_coding	1.83857872	1.53990386	0.26983231	5.70689213	1.1506E-08	3.1082E-08
CCDC192	ENSG00000230561	protein_coding	19.9079251	1.18992694	0.11621975	10.2385953	1.3316E-24	9.9771E-24
TEX48	ENSG00000230601	protein_coding	0.89701281	2.12349126	0.3945815	5.38162906	7.3815E-08	1.8683E-07
PLSCR5	ENSG00000231213	protein_coding	0.47052492	1.23834116	0.48626607	2.5466329	0.01087678	0.0161926
HLA-DPA1	ENSG00000231389	protein_coding	36348.2009	1.83654514	0.11976729	15.3342793	4.5125E-53	1.2013E-51
CYP21A2	ENSG00000231852	protein_coding	101.293155	2.49869477	0.19878496	12.5698381	3.0936E-36	4.1248E-35
PSG1	ENSG00000231924	protein_coding	2.87439196	2.82848373	0.40318782	7.01530058	2.2946E-12	8.2259E-12
TAPBP	ENSG00000231925	protein_coding	22658.9621	1.59712616	0.07334327	21.7760427	3.915E-105	7.207E-103
DYTN	ENSG00000232125	protein_coding	0.7656262	1.62891494	0.39235582	4.15162683	3.3012E-05	6.5386E-05
ASCL5	ENSG00000232237	protein_coding	12.0995944	1.82166889	0.17884872	10.1855294	2.301E-24	1.7016E-23
AJM1	ENSG00000232434	protein_coding	421.160852	2.06422986	0.11422872	18.0710232	5.3905E-73	2.9037E-71
HLA-DQB2	ENSG00000232629	protein_coding	1797.27953	2.80072613	0.17846071	15.693797	1.6677E-55	4.8295E-54
GOLGA8N	ENSG00000232653	protein_coding	139.194603	1.15003352	0.12972073	8.86545668	7.6191E-19	4.1669E-18
NPIPA9	ENSG00000233024	protein_coding	4.71161687	1.81573678	0.21425726	8.47456374	2.3596E-17	1.1788E-16
PHGR1	ENSG00000233041	protein_coding	0.98771651	2.34185154	0.36285433	6.45397157	1.0896E-10	3.4577E-10
PIRT	ENSG00000233670	protein_coding	1.59553437	1.33774745	0.34049339	3.92885003	8.5353E-05	0.00016185
H2BC15	ENSG00000233822	protein_coding	125.927949	1.10850032	0.09500395	11.6679389	1.8587E-31	1.9756E-30
RPS28	ENSG00000233927	protein_coding	13126.3096	1.00746036	0.09894011	10.1825269	2.3732E-24	1.7535E-23
PAGE2	ENSG00000234068	protein_coding	1.63570476	3.09497744	0.48653337	6.36128506	2.0007E-10	6.2239E-10
C16orf82	ENSG00000234186	protein_coding	0.69941937	1.8950868	0.46306815	4.09245768	4.2683E-05	8.358E-05
CCDC188	ENSG00000234409	protein_coding	38.7038807	1.72713341	0.14752488	11.7074045	1.168E-31	1.2557E-30

C5orf58	ENSG00000234511	protein_coding	28.6990828	2.93308831	0.17182787	17.069922	2.4853E-65	1.0082E-63
HLA-B	ENSG00000234745	protein_coding	176919.795	1.92345303	0.08378362	22.9573861	1.243E-116	3.081E-114
IQCM	ENSG00000234828	protein_coding	1.54265603	2.00414255	0.43922391	4.56291767	5.0448E-06	1.0805E-05
APOC2	ENSG00000234906	protein_coding	24.7691282	2.02126483	0.20195575	10.0084538	1.3992E-23	9.8908E-23
AL158151.3	ENSG00000235007	protein_coding	1.43626898	1.51116039	0.27913756	5.4136763	6.1744E-08	1.5717E-07
RAMACL	ENSG00000235272	protein_coding	7.65212149	1.15690403	0.3156151	3.66555346	0.0002468	0.00044534
NFAM1	ENSG00000235568	protein_coding	883.104165	2.10373365	0.1057681	19.8900584	4.9617E-88	4.6326E-86
SLFN14	ENSG00000236320	protein_coding	5.88919525	2.55891778	0.24080099	10.6266915	2.2392E-26	1.8495E-25
PPIAL4G	ENSG00000236334	protein_coding	1.23694878	1.81770248	0.32570711	5.5807884	2.3943E-08	6.297E-08
SLC35G4	ENSG00000236396	protein_coding	0.7080349	2.13071702	0.58770392	3.62549394	0.00028841	0.00051624
AL354761.1	ENSG00000236543	protein_coding	0.70186321	1.98633008	0.80172054	2.47758412	0.01322752	0.01945029
CTAGE9	ENSG00000236761	protein_coding	40.6711333	4.07395655	0.2356051	17.291462	5.4551E-67	2.3458E-65
C3orf84	ENSG00000236980	protein_coding	1.14926359	2.3079615	0.38445735	6.00316661	1.9351E-09	5.5828E-09
C4orf51	ENSG00000237136	protein_coding	7.92445749	1.34297059	0.25041319	5.36301853	8.1843E-08	2.0628E-07
PATE4	ENSG00000237353	protein_coding	1.21007076	1.90949569	0.41388186	4.61362499	3.9571E-06	8.5633E-06
BHMG1	ENSG00000237452	protein_coding	3.47004658	1.83290646	0.37961167	4.82837232	1.3765E-06	3.1082E-06
HLA-DQA2	ENSG00000237541	protein_coding	3119.41562	2.45007184	0.22090359	11.0911362	1.3852E-28	1.2794E-27
KIFC1	ENSG00000237649	protein_coding	316.803945	1.95018283	0.12235916	15.9381839	3.4421E-57	1.059E-55
OR2I1P	ENSG00000237988	protein_coding	1726.85668	3.81493788	0.21495505	17.747608	1.7985E-70	8.7802E-69
ETDA	ENSG00000238210	protein_coding	0.89315475	2.3314206	0.31281732	7.4529781	9.1256E-14	3.6088E-13
PAGE2B	ENSG00000238269	protein_coding	1.42013157	2.38282165	0.33033514	7.21334591	5.4593E-13	2.0425E-12
ALKBH6	ENSG00000239382	protein_coding	154.2441	1.68617822	0.11179615	15.0826139	2.1076E-51	5.2736E-50
OR1J4	ENSG00000239590	protein_coding	0.71544767	1.84310503	0.45680892	4.03473961	5.4663E-05	0.00010577
STPG4	ENSG00000239605	protein_coding	4.99457998	1.79992576	0.23473183	7.66800885	1.7469E-14	7.2377E-14
PRR20G	ENSG00000239620	protein_coding	1.22255308	2.91988799	0.42271084	6.90753034	4.9316E-12	1.7268E-11

MEIKIN	ENSG00000239642	protein_coding	1.70256406	1.55794219	0.27886928	5.58663975	2.3151E-08	6.0973E-08
CDRT4	ENSG00000239704	protein_coding	35.6003175	1.5023875	0.14876224	10.0992534	5.5664E-24	4.0222E-23
APOBEC3G	ENSG00000239713	protein_coding	1611.76296	2.32419328	0.1135234	20.4732534	3.7288E-93	4.2947E-91
TLR9	ENSG00000239732	protein_coding	3.53289734	1.51794107	0.18970475	8.00159754	1.2282E-15	5.5045E-15
DEFA3	ENSG00000239839	protein_coding	3.55049226	1.88510841	0.40462997	4.65884528	3.1799E-06	6.942E-06
LILRA4	ENSG00000239961	protein_coding	77.9781672	2.51393424	0.19422535	12.9433892	2.5608E-38	3.7231E-37
LILRA2	ENSG00000239998	protein_coding	267.916082	1.88737349	0.11998085	15.7306221	9.329E-56	2.7222E-54
AMY2B	ENSG00000240038	protein_coding	550.86328	1.75128687	0.15841416	11.055116	2.0707E-28	1.8958E-27
LY6G5B	ENSG00000240053	protein_coding	145.978775	2.35374311	0.16056025	14.6595631	1.1702E-48	2.6284E-47
PSMB9	ENSG00000240065	protein_coding	3992.63675	2.21818368	0.1040473	21.3189936	7.567E-101	1.1719E-98
PCDHGC3	ENSG00000240184	protein_coding	1788.82381	1.10171574	0.1099973	10.0158436	1.2985E-23	9.1986E-23
UGT1A5	ENSG00000240224	protein_coding	1.99919485	2.78480711	0.37829395	7.36149	1.8187E-13	7.0468E-13
ACAD11	ENSG00000240303	protein_coding	495.825105	1.11985465	0.15118098	7.40737812	1.2882E-13	5.0434E-13
KIR3DL2	ENSG00000240403	protein_coding	8.55856819	2.36506899	0.1995286	11.8532829	2.0692E-32	2.3018E-31
TNFRSF13B	ENSG00000240505	protein_coding	17.1597451	1.03117522	0.23909659	4.31279767	1.612E-05	3.2866E-05
PNMA2	ENSG00000240694	protein_coding	4385.58278	2.81848143	0.14865653	18.959688	3.6732E-80	2.5865E-78
LRRD1	ENSG00000240720	protein_coding	5.36630396	1.67894063	0.20606039	8.14780872	3.7058E-16	1.7132E-15
PCDHGC5	ENSG00000240764	protein_coding	37.0820132	2.15333785	0.16440021	13.0981454	3.374E-39	5.1079E-38
MIF	ENSG00000240972	protein_coding	3111.61602	1.13374216	0.11842142	9.57379269	1.0305E-21	6.6118E-21
HLA-DOB	ENSG00000241106	protein_coding	280.883559	2.16664853	0.17339951	12.4951251	7.9371E-36	1.0375E-34
RPL36A	ENSG00000241343	protein_coding	3879.66739	1.26458198	0.09688749	13.0520668	6.1847E-39	9.2349E-38
EGFL8	ENSG00000241404	protein_coding	103.815076	1.87252345	0.1652063	11.3344556	8.8581E-30	8.697E-29
AC244197.3	ENSG00000241489	protein_coding	71.9608427	1.9187406	0.16299506	11.7717713	5.4567E-32	5.9543E-31
UGT1A1	ENSG00000241635	protein_coding	21.8831094	2.22621015	0.22346292	9.9623246	2.2279E-23	1.5573E-22
PLEKHO2	ENSG00000241839	protein_coding	3862.76132	1.02924175	0.07405175	13.8989523	6.4275E-44	1.1833E-42

KIR3DL3	ENSG00000242019	protein_coding	0.62472241	1.96278829	0.46602649	4.2117526	2.534E-05	5.076E-05
HYPK	ENSG00000242028	protein_coding	146.489979	1.38450054	0.12006791	11.5309789	9.2091E-31	9.4294E-30
MTFP1	ENSG00000242114	protein_coding	769.82017	1.63583698	0.10380675	15.7584842	6.0056E-56	1.7614E-54
PSG2	ENSG00000242221	protein_coding	1.12074556	2.62324605	0.47806484	5.48721806	4.0831E-08	1.0534E-07
BGLAP	ENSG00000242252	protein_coding	38.7334506	2.30127715	0.17325167	13.2828568	2.9107E-40	4.5916E-39
UGT1A8	ENSG00000242366	protein_coding	28.3248813	2.78072526	0.36318228	7.65655543	1.9099E-14	7.8938E-14
PCDHGC4	ENSG00000242419	protein_coding	26.7053773	1.85282174	0.15674906	11.8203049	3.0657E-32	3.388E-31
UGT1A10	ENSG00000242515	protein_coding	51.2611847	3.91191339	0.41325425	9.46611781	2.9044E-21	1.8179E-20
HLA-DMB	ENSG00000242574	protein_coding	4711.31044	1.08289383	0.10344335	10.4684722	1.2057E-25	9.5809E-25
STRC	ENSG00000242866	protein_coding	8.49842825	2.77531476	0.26807251	10.3528509	4.0621E-25	3.1254E-24
ERVW-1	ENSG00000242950	protein_coding	19.7811486	1.18577888	0.17893142	6.62700219	3.4257E-11	1.1308E-10
EIF4EBP3	ENSG00000243056	protein_coding	615.104224	1.27695414	0.10162565	12.5652735	3.2774E-36	4.3661E-35
PSG11	ENSG00000243130	protein_coding	1.21093868	2.19991469	0.50746051	4.33514457	1.4566E-05	2.9847E-05
UGT1A3	ENSG00000243135	protein_coding	26.5417745	4.2919162	0.29551834	14.5233496	8.6193E-48	1.8568E-46
KRTAP10-11	ENSG00000243489	protein_coding	0.83145642	1.82620913	0.63699838	2.8668976	0.00414517	0.00651977
WFDC6	ENSG00000243543	protein_coding	0.7338305	1.49011524	0.53303262	2.79554232	0.00518127	0.00804998
IL10RB	ENSG00000243646	protein_coding	2690.08323	1.02287912	0.0592358	17.2679223	8.2043E-67	3.5051E-65
CFB	ENSG00000243649	protein_coding	1410.15659	1.33912508	0.14644674	9.14410958	6.0123E-20	3.5029E-19
AC006254.1	ENSG00000243696	protein_coding	62.9764406	1.74766513	0.18499993	9.44684235	3.4921E-21	2.1732E-20
PLA2G4B	ENSG00000243708	protein_coding	30.1443159	1.83838863	0.1799509	10.2160567	1.6804E-24	1.2513E-23
LEFTY1	ENSG00000243709	protein_coding	84.8216299	1.03084261	0.28924946	3.56385314	0.00036545	0.00064702
NPIPB5	ENSG00000243716	protein_coding	263.46225	2.93353815	0.19118376	15.3440761	3.8804E-53	1.0366E-51
KIR2DL3	ENSG00000243772	protein_coding	11.6756083	2.56158144	0.18470912	13.8681917	9.8735E-44	1.8075E-42
JMJD7	ENSG00000243789	protein_coding	9.19522683	1.03262716	0.14831216	6.96252546	3.3423E-12	1.1837E-11
APOBEC3D	ENSG00000243811	protein_coding	394.996873	2.01044552	0.10795517	18.6229665	2.0929E-77	1.3381E-75

IFITM10	ENSG00000244242	protein_coding	715.052181	1.39436656	0.15949459	8.74240665	2.282E-18	1.212E-17
AL645922.1	ENSG00000244255	protein_coding	1.15225372	1.75894741	0.33169878	5.30284556	1.1401E-07	2.8352E-07
KRTAP5-7	ENSG00000244411	protein_coding	3.47169864	1.16443474	0.24852722	4.68534075	2.7949E-06	6.1344E-06
UGT1A4	ENSG00000244474	protein_coding	4.07168974	3.18672503	0.37705246	8.45167541	2.8715E-17	1.4274E-16
LILRA6	ENSG00000244482	protein_coding	125.047972	2.50302734	0.13540865	18.4849892	2.7275E-76	1.6828E-74
APOBEC3C	ENSG00000244509	protein_coding	3644.07213	2.09395298	0.11259391	18.5973916	3.3733E-77	2.1477E-75
PTCHD4	ENSG00000244694	protein_coding	296.361753	1.11633799	0.161957	6.8928049	5.4703E-12	1.9085E-11
C4A	ENSG00000244731	protein_coding	4635.80016	1.68899419	0.16864124	10.0153095	1.3055E-23	9.247E-23
CRYBB2	ENSG00000244752	protein_coding	15.1095432	1.77421587	0.16852548	10.527879	6.4266E-26	5.1748E-25
CEBPA	ENSG00000245848	protein_coding	603.57169	1.46777468	0.1369203	10.7199205	8.2074E-27	6.9403E-26
UBAP1L	ENSG00000246922	protein_coding	315.90157	2.16345887	0.14260579	15.1709045	5.5112E-52	1.4209E-50
INSL3	ENSG00000248099	protein_coding	22.5186815	1.45042307	0.18814258	7.70916963	1.2664E-14	5.2968E-14
POU5F2	ENSG00000248483	protein_coding	9.51160677	2.6425549	0.25432637	10.3904085	2.7418E-25	2.1346E-24
STIMATE-MUSTN1	ENSG00000248592	protein_coding	24.8180576	1.04364922	0.14961354	6.97563336	3.045E-12	1.0824E-11
ACTN3	ENSG00000248746	protein_coding	6.67994529	1.53670114	0.20768321	7.39925541	1.3695E-13	5.3498E-13
AC004997.1	ENSG00000248751	protein_coding	7.9756449	1.10701805	0.16504652	6.7073091	1.9825E-11	6.6494E-11
TNFSF12-TNFSF13	ENSG00000248871	protein_coding	5.41444423	1.02754507	0.16525324	6.21800245	5.0352E-10	1.5227E-09
AL159163.1	ENSG00000249141	protein_coding	13.8173894	2.23908932	0.16076635	13.9275995	4.3059E-44	7.9978E-43
ATP5MGL	ENSG00000249222	protein_coding	3.5719455	1.30450038	0.24394358	5.34754957	8.9153E-08	2.2384E-07
NAIP	ENSG00000249437	protein_coding	103.37371	1.36206179	0.1476187	9.22689222	2.786E-20	1.649E-19
ECSCR	ENSG00000249751	protein_coding	589.368758	1.26767713	0.11120457	11.3995054	4.205E-30	4.1787E-29
TERB1	ENSG00000249961	protein_coding	4.11830772	1.63109611	0.2199479	7.4158294	1.2087E-13	4.7433E-13
YJEFN3	ENSG00000250067	protein_coding	182.642523	2.08683308	0.18197996	11.4673783	1.924E-30	1.9457E-29

TRIM75P	ENSG00000250374	protein_coding	2.18146821	2.14018472	0.31143524	6.87200573	6.3305E-12	2.2008E-11
CDK3	ENSG00000250506	protein_coding	32.8164825	2.39955875	0.17055037	14.0695018	5.848E-45	1.1203E-43
REELD1	ENSG00000250673	protein_coding	12.2173372	2.34184809	0.21563165	10.8604098	1.7795E-27	1.5535E-26
AC010255.3	ENSG00000250803	protein_coding	1.07216812	1.82457404	0.35501939	5.1393645	2.7567E-07	6.6398E-07
SHANK3	ENSG00000251322	protein_coding	4740.08389	1.21262422	0.10788266	11.2402146	2.5875E-29	2.4914E-28
AC005324.3	ENSG00000251537	protein_coding	3.90536522	2.32442381	0.26788255	8.67702574	4.0625E-18	2.1264E-17
PTX4	ENSG00000251692	protein_coding	4.98289772	2.16378674	0.23026576	9.39691044	5.6189E-21	3.4565E-20
TMEM200B	ENSG00000253304	protein_coding	786.329237	1.17768671	0.1190135	9.89540472	4.3584E-23	3.0017E-22
SERPINE3	ENSG00000253309	protein_coding	18.7196396	1.41156386	0.1368242	10.3166241	5.9271E-25	4.5103E-24
PCDHGA5	ENSG00000253485	protein_coding	128.822087	1.02450158	0.16768785	6.1095755	9.9896E-10	2.9502E-09
PRSS51	ENSG00000253649	protein_coding	29.0199019	1.89996103	0.1759627	10.7975217	3.5362E-27	3.0465E-26
ETV3L	ENSG00000253831	protein_coding	2.74008424	2.77658188	0.34754517	7.9891251	1.359E-15	6.079E-15
PCDHGB2	ENSG00000253910	protein_coding	324.541077	1.62110837	0.19968741	8.11823011	4.7303E-16	2.1734E-15
PCDHGB7	ENSG00000254122	protein_coding	1019.96522	1.26136844	0.1811545	6.9629427	3.3324E-12	1.1804E-11
NPIPB11	ENSG00000254206	protein_coding	77.3756561	2.33912145	0.18280787	12.7955183	1.7369E-37	2.4521E-36
CHKB-CPT1B	ENSG00000254413	protein_coding	63.8970391	1.71474802	0.17009768	10.0809607	6.7067E-24	4.8219E-23
SIGLEC14	ENSG00000254415	protein_coding	239.198783	1.61251896	0.15177163	10.6246405	2.2889E-26	1.8895E-25
PBOV1	ENSG00000254440	protein_coding	1.51029465	1.55534088	0.36332314	4.28087483	1.8616E-05	3.775E-05
SIGLEC12	ENSG00000254521	protein_coding	44.5311457	2.59224997	0.23204816	11.171172	5.6431E-29	5.3315E-28
PABPC4L	ENSG00000254535	protein_coding	564.911548	1.27355434	0.1197661	10.6336794	2.0775E-26	1.719E-25
AL360181.3	ENSG00000254536	protein_coding	1.30882267	1.55788436	0.28025012	5.55890693	2.7147E-08	7.1099E-08
ARMS2	ENSG00000254636	protein_coding	4.73386211	1.49455056	0.19401823	7.70314489	1.3276E-14	5.5446E-14
RTL1	ENSG00000254656	protein_coding	9.18127403	4.34570224	0.60229404	7.21525025	5.3835E-13	2.0151E-12
IGLL5	ENSG00000254709	protein_coding	1535.23827	2.18625758	0.27924982	7.8290385	4.9162E-15	2.1167E-14
AP001931.1	ENSG00000254732	protein_coding	16.3495671	1.24955936	0.15888442	7.86458056	3.7034E-15	1.6061E-14

EEF1G	ENSG00000254772	protein_coding	25.4960131	1.03841375	0.14356839	7.23288556	4.7284E-13	1.7775E-12
AP000781.2	ENSG00000254979	protein_coding	16.6025481	3.09250275	0.18093755	17.0915474	1.7156E-65	6.9969E-64
STX16-NPEPL1	ENSG00000254995	protein_coding	65.8760334	1.98903384	0.17582507	11.3125726	1.1371E-29	1.1111E-28
SAA2-SAA4	ENSG00000255071	protein_coding	81.3686441	4.25126045	0.51237162	8.2972208	1.0658E-16	5.1088E-16
AC005324.4	ENSG00000255104	protein_coding	1.88498011	3.09419532	0.33046045	9.36328474	7.7296E-21	4.7187E-20
GLYATL1B	ENSG00000255151	protein_coding	1.31496917	2.30069669	0.3301544	6.96854772	3.2023E-12	1.1364E-11
CARD17	ENSG00000255221	protein_coding	4.66995371	3.05174798	0.2720808	11.2163296	3.3905E-29	3.2455E-28
AL096711.2	ENSG00000255330	protein_coding	24.8539293	1.61275369	0.15817587	10.1959525	2.0671E-24	1.534E-23
AL133352.1	ENSG00000255339	protein_coding	7.1675348	1.82103349	0.18316009	9.94230493	2.7245E-23	1.8958E-22
TAS2R43	ENSG00000255374	protein_coding	2.58325113	1.34483118	0.29145409	4.61421274	3.9459E-06	8.5403E-06
EBLN2	ENSG00000255423	protein_coding	39.8108987	1.2453635	0.13624281	9.14076483	6.2012E-20	3.6098E-19
AC135050.2	ENSG00000255439	protein_coding	5.86552963	1.37539921	0.16645612	8.26283331	1.4225E-16	6.7597E-16
NPIPB8	ENSG00000255524	protein_coding	1.42388659	1.87639633	0.32337176	5.80259807	6.5295E-09	1.8007E-08
LY6G6E	ENSG00000255552	protein_coding	0.54675101	1.32042739	0.34899	3.78356795	0.0001546	0.00028524
OR10G2	ENSG00000255582	protein_coding	0.58370029	1.79899843	0.5014661	3.58747763	0.00033389	0.00059348
RAB44	ENSG00000255587	protein_coding	13.8550765	1.70179853	0.14682988	11.5902738	4.6165E-31	4.8108E-30
AC068775.1	ENSG00000255641	protein_coding	0.52414301	1.39512522	0.33849576	4.12154415	3.7634E-05	7.4125E-05
AP002373.1	ENSG00000255663	protein_coding	2.12609177	1.50541015	0.2197394	6.85088864	7.3393E-12	2.5403E-11
AC011462.1	ENSG00000255730	protein_coding	8.79939579	2.77649195	0.19366902	14.336273	1.2984E-46	2.6703E-45
KLRC4-KLRK1	ENSG00000255819	protein_coding	14.980232	2.29129197	0.19773005	11.5879804	4.7418E-31	4.9369E-30
TIFAB	ENSG00000255833	protein_coding	42.3323976	2.42431415	0.19211913	12.6188065	1.6632E-36	2.2498E-35
TAS2R20	ENSG00000255837	protein_coding	27.7036891	1.98122255	0.15042909	13.170475	1.2978E-39	1.997E-38
CYP2A6	ENSG00000255974	protein_coding	6.26885815	1.35101641	0.20292299	6.65777895	2.78E-11	9.2312E-11
TAS2R30	ENSG00000256188	protein_coding	2.47489883	1.55545773	0.27693001	5.61679005	1.9454E-08	5.1569E-08
SMIM3	ENSG00000256235	protein_coding	4044.32205	1.15313122	0.16411005	7.02657275	2.1167E-12	7.6103E-12

TAS2R31	ENSG00000256436	protein_coding	12.6863654	1.31313491	0.18519271	7.09064025	1.3349E-12	4.8726E-12
AP003419.1	ENSG00000256514	protein_coding	0.94701503	1.59175383	0.3780344	4.21060579	2.5469E-05	5.1005E-05
POLG2	ENSG00000256525	protein_coding	513.603475	1.09503912	0.07858537	13.9343885	3.9154E-44	7.2814E-43
OR13A1	ENSG00000256574	protein_coding	7.54829551	1.83659912	0.27821075	6.60146704	4.0711E-11	1.3346E-10
CLEC12B	ENSG00000256660	protein_coding	13.4177908	3.73797685	0.24920093	14.9998514	7.3584E-51	1.8029E-49
PGA5	ENSG00000256713	protein_coding	68.757743	2.33848723	0.25051088	9.33487305	1.0111E-20	6.1351E-20
STH	ENSG00000256762	protein_coding	2.2774895	3.15669213	0.35540429	8.88197533	6.5683E-19	3.6064E-18
KLRF2	ENSG00000256797	protein_coding	4.33313424	3.39629345	0.36798541	9.22942416	2.7209E-20	1.6115E-19
SLC5A8	ENSG00000256870	protein_coding	2096.11362	1.50320609	0.26920113	5.58395171	2.3511E-08	6.1881E-08
AC069503.2	ENSG00000256950	protein_coding	9.92093361	1.89990885	0.19561997	9.71224387	2.6738E-22	1.7654E-21
KHDC1L	ENSG00000256980	protein_coding	1.2120793	2.15504321	0.35341234	6.09781539	1.0753E-09	3.1674E-09
GPR142	ENSG00000257008	protein_coding	1.55040943	1.96369901	0.29420995	6.67448199	2.4811E-11	8.2722E-11
HP	ENSG00000257017	protein_coding	1896.75383	5.69826895	0.39572644	14.3995154	5.2108E-47	1.0897E-45
TAS2R38	ENSG00000257138	protein_coding	0.65111011	1.78351781	0.53431533	3.33794992	0.00084399	0.00143656
AL928654.3	ENSG00000257341	protein_coding	3.45289261	1.67601435	0.24202659	6.92491831	4.3623E-12	1.533E-11
AL157935.2	ENSG00000257524	protein_coding	24.8253232	2.14959431	0.16950181	12.6818369	7.4564E-37	1.0225E-35
MGAM2	ENSG00000257743	protein_coding	81.8732925	2.06215199	0.19573382	10.5354917	5.9273E-26	4.787E-25
OR9A4	ENSG00000258083	protein_coding	0.87045512	2.09910692	0.3948424	5.31631591	1.0589E-07	2.6402E-07
PRSS58	ENSG00000258223	protein_coding	0.49454378	1.52493873	0.45468759	3.35381645	0.00079705	0.00135984
CLEC5A	ENSG00000258227	protein_coding	303.040412	2.04717148	0.15777559	12.9752101	1.6913E-38	2.4771E-37
AC009779.3	ENSG00000258311	protein_coding	206.090571	2.00607289	0.11387043	17.6171539	1.8193E-69	8.5978E-68
C17orf49	ENSG00000258315	protein_coding	87.2172582	1.00066913	0.08404754	11.9059897	1.1015E-32	1.2391E-31
RTEL1	ENSG00000258366	protein_coding	86.4509152	1.13824018	0.09522751	11.9528502	6.2736E-33	7.1557E-32
PPT2-EGFL8	ENSG00000258388	protein_coding	66.6871901	1.18215012	0.1158085	10.207801	1.8296E-24	1.3607E-23
AC012651.1	ENSG00000258461	protein_coding	128.621238	1.72453109	0.1592903	10.826341	2.5827E-27	2.2423E-26

BCL2L2-PABPN1	ENSG00000258643	protein_coding	13.747882	1.01200527	0.13447711	7.52548325	5.2526E-14	2.1116E-13
C20orf141	ENSG00000258713	protein_coding	1.54607798	2.58359359	0.49467231	5.22283854	1.762E-07	4.3161E-07
AL121594.1	ENSG00000258790	protein_coding	9.97212863	2.01500288	0.19774903	10.1896981	2.2045E-24	1.6326E-23
RNASE4	ENSG00000258818	protein_coding	289.987558	1.01252576	0.12508796	8.09451	5.7495E-16	2.627E-15
MC1R	ENSG00000258839	protein_coding	236.337491	1.58271752	0.13130261	12.0539681	1.8483E-33	2.1604E-32
AC007040.2	ENSG00000258881	protein_coding	8.85798975	1.24026318	0.21676973	5.7215699	1.0554E-08	2.8602E-08
TUBB3	ENSG00000258947	protein_coding	61.311291	1.59245529	0.22737405	7.00368094	2.4932E-12	8.9185E-12
TMEM179	ENSG00000258986	protein_coding	227.401141	3.9893224	0.27840649	14.3291287	1.4391E-46	2.9544E-45
AL163636.2	ENSG00000259171	protein_coding	37.3119072	1.14526678	0.1332218	8.59669179	8.2047E-18	4.2099E-17
SLC35G6	ENSG00000259224	protein_coding	3.59092371	1.13607641	0.23280604	4.87992662	1.0613E-06	2.4215E-06
ZHX1-C8orf76	ENSG00000259305	protein_coding	32.0684487	1.03913593	0.10126931	10.2611136	1.0548E-24	7.9439E-24
AC087632.2	ENSG00000259316	protein_coding	4.06335824	1.0790967	0.17506933	6.1638249	7.1008E-10	2.1228E-09
GH1	ENSG00000259384	protein_coding	1.89906188	1.75226737	0.27144281	6.45538325	1.0795E-10	3.4278E-10
AL136295.5	ENSG00000259529	protein_coding	30.2947093	1.64157123	0.14775291	11.1102465	1.1185E-28	1.0402E-27
BLID	ENSG00000259571	protein_coding	0.82103504	1.91761112	0.45696516	4.1964055	2.7118E-05	5.4146E-05
AC136428.1	ENSG00000259680	protein_coding	5.2387025	1.40876294	0.3640782	3.86939653	0.00010911	0.00020463
AC107871.1	ENSG00000260007	protein_coding	0.67750902	1.46289377	0.30444526	4.80511266	1.5466E-06	3.476E-06
AC020636.2	ENSG00000260234	protein_coding	0.81729851	2.2391728	0.36743708	6.09403059	1.101E-09	3.2393E-09
AC093525.2	ENSG00000260272	protein_coding	7.4751514	1.21917862	0.1823741	6.68504252	2.3086E-11	7.7128E-11
SCX	ENSG00000260428	protein_coding	29.4943321	1.02147839	0.17093357	5.97587918	2.2885E-09	6.5666E-09
KCNJ18	ENSG00000260458	protein_coding	1.57995359	1.45759456	0.3770127	3.86616834	0.00011056	0.00020719
AC009690.1	ENSG00000260729	protein_coding	24.7169163	1.14044542	0.1720746	6.62762203	3.4114E-11	1.1262E-10
AL049634.2	ENSG00000260861	protein_coding	1.49103126	2.92188951	0.31347194	9.32105595	1.1519E-20	6.9753E-20
SULT1A3	ENSG00000261052	protein_coding	4.16727044	2.40106046	0.26070909	9.20973033	3.2695E-20	1.9264E-19
CLEC19A	ENSG00000261210	protein_coding	0.70062669	1.15974893	0.50311274	2.3051472	0.02115833	0.03024193

MUC22	ENSG00000261272	protein_coding	5.39692752	2.63859993	0.30633361	8.61348484	7.0873E-18	3.6516E-17
AC010325.1	ENSG00000261341	protein_coding	2.75769514	2.15168724	0.27395323	7.85421374	4.0229E-15	1.7412E-14
PECAM1	ENSG00000261371	protein_coding	37507.2482	1.22195149	0.10128347	12.0646688	1.6231E-33	1.904E-32
TEN1-CDK3	ENSG00000261408	protein_coding	90.1061909	2.00308186	0.13813169	14.5012474	1.1897E-47	2.5532E-46
TMEM249	ENSG00000261587	protein_coding	4.72561976	2.37669515	0.29115928	8.16286934	3.2716E-16	1.5175E-15
TPBGL	ENSG00000261594	protein_coding	87.4725725	2.34345691	0.21125014	11.0932799	1.3524E-28	1.2504E-27
GOLGA6L7	ENSG00000261649	protein_coding	18.5760217	3.11379236	0.35187896	8.84904398	8.8272E-19	4.8104E-18
HPR	ENSG00000261701	protein_coding	25.125943	1.38216419	0.38538469	3.58645329	0.00033521	0.00059566
AL031708.1	ENSG00000261732	protein_coding	15.1598056	2.22379903	0.19283511	11.5321273	9.087E-31	9.3106E-30
GOLGA8S	ENSG00000261739	protein_coding	5.85725278	1.53330387	0.20343601	7.5370327	4.8079E-14	1.9367E-13
MIA	ENSG00000261857	protein_coding	4.21324288	1.25688426	0.28404153	4.42500177	9.6441E-06	2.0115E-05
AC040162.1	ENSG00000261884	protein_coding	154.158123	2.10300521	0.13055414	16.1082993	2.2308E-58	7.1611E-57
LINC00514	ENSG00000262152	protein_coding	17.2043853	1.90404529	0.16990836	11.206307	3.7971E-29	3.6225E-28
CORO7	ENSG00000262246	protein_coding	785.023466	1.54309949	0.09084375	16.986304	1.0372E-64	4.1274E-63
MMP12	ENSG00000262406	protein_coding	43.1520399	2.12524171	0.33240315	6.39356673	1.6206E-10	5.08E-10
CCER2	ENSG00000262484	protein_coding	24.8490858	2.62484488	0.21440863	12.2422541	1.8483E-34	2.263E-33
C19orf84	ENSG00000262874	protein_coding	12.0531202	1.98915037	0.17024751	11.6838736	1.5411E-31	1.6456E-30
DPEP2NB	ENSG00000263201	protein_coding	1.11117734	2.18688089	0.41292144	5.29611854	1.1829E-07	2.9375E-07
AC119396.1	ENSG00000263264	protein_coding	25.53923	1.14656334	0.13659368	8.39397064	4.6999E-17	2.3033E-16
FAM72C	ENSG00000263513	protein_coding	6.68638826	2.35961508	0.20397893	11.5679354	5.9907E-31	6.1991E-30
MSMB	ENSG00000263639	protein_coding	3.0040711	2.41157773	0.46487793	5.18755047	2.1308E-07	5.1785E-07
LINC00672	ENSG00000263874	protein_coding	66.6278689	1.15495503	0.11032875	10.4683054	1.2079E-25	9.5961E-25
RHEX	ENSG00000263961	protein_coding	3206.94047	1.96756345	0.1615825	12.1768352	4.13E-34	4.9848E-33
AKR1C8P	ENSG00000264006	protein_coding	12.6673154	1.63951903	0.25007541	6.55609845	5.5234E-11	1.7928E-10
AC055811.2	ENSG00000264187	protein_coding	5.78529503	1.61265793	0.17833171	9.04302419	1.524E-19	8.6802E-19

ANXA8L1	ENSG00000264230	protein_coding	10.7198304	1.93234288	0.24476476	7.89469395	2.9103E-15	1.2711E-14
MYH4	ENSG00000264424	protein_coding	6.36734888	1.02084316	0.37110141	2.75084689	0.00594414	0.0091715
NPY4R2	ENSG00000264717	protein_coding	1.32927517	2.38205637	0.40857129	5.83020993	5.5358E-09	1.5361E-08
AC134669.1	ENSG00000265118	protein_coding	5.06789606	1.69136156	0.19264894	8.77950079	1.642E-18	8.8037E-18
ANXA8	ENSG00000265190	protein_coding	8.10105807	1.85799588	0.39306298	4.72696732	2.279E-06	5.0418E-06
AC074143.1	ENSG00000265690	protein_coding	0.27982287	1.07007185	0.45654876	2.34382818	0.01908696	0.02744473
FSBP	ENSG00000265817	protein_coding	0.58545641	1.0255777	0.32709652	3.13539779	0.00171621	0.00282103
AC004805.1	ENSG00000266076	protein_coding	1.21854563	1.4228463	0.27864961	5.10622029	3.2867E-07	7.8606E-07
RASSF5	ENSG00000266094	protein_coding	1680.3297	1.69438582	0.1069639	15.8407266	1.6293E-56	4.8656E-55
AC098850.3	ENSG00000266302	protein_coding	19.9351672	1.64414176	0.31966947	5.14325552	2.7002E-07	6.5106E-07
NBPF15	ENSG00000266338	protein_coding	848.998873	1.23329367	0.08201148	15.0380606	4.1349E-51	1.0218E-49
MYO15B	ENSG00000266714	protein_coding	7406.21383	1.60421713	0.13473257	11.9066769	1.0924E-32	1.2295E-31
AC015688.4	ENSG00000266728	protein_coding	1.24962222	1.86772928	0.27454376	6.80302931	1.0244E-11	3.5029E-11
FXYD1	ENSG00000266964	protein_coding	231.195787	1.83046957	0.23192923	7.89236253	2.9652E-15	1.2942E-14
AD000671.2	ENSG00000267120	protein_coding	4.53064346	1.82359998	0.19557443	9.32432725	1.1169E-20	6.768E-20
AC007998.2	ENSG00000267140	protein_coding	2.12569659	1.06374841	0.20419128	5.20956835	1.8928E-07	4.6239E-07
ATF7-NPFF	ENSG00000267281	protein_coding	103.33389	2.30689695	0.153277	15.0505092	3.4259E-51	8.5073E-50
AC011511.4	ENSG00000267303	protein_coding	0.67943407	1.1320498	0.30228374	3.74499073	0.0001804	0.00033056
APOC4	ENSG00000267467	protein_coding	0.72141388	1.57602617	0.4771473	3.30301808	0.0009565	0.00161791
LIN37	ENSG00000267796	protein_coding	206.133177	1.00025088	0.07055622	14.1766496	1.278E-45	2.5253E-44
CCDC177	ENSG00000267909	protein_coding	4.80633345	2.47467727	0.35484716	6.9739243	3.0822E-12	1.095E-11
ERFL	ENSG00000268041	protein_coding	8.19201376	2.8880624	0.22147689	13.0400171	7.2441E-39	1.0764E-37
GABRQ	ENSG00000268089	protein_coding	157.998239	1.47017084	0.18765628	7.8343814	4.7116E-15	2.0313E-14
AC090004.1	ENSG00000268279	protein_coding	20.9357538	1.98350956	0.15515863	12.7837529	2.0208E-37	2.8442E-36
NFILZ	ENSG00000268480	protein_coding	0.55908916	1.78490741	0.5708149	3.12694605	0.00176632	0.00289903

AC003002.2	ENSG00000268533	protein_coding	5.49093416	1.46606313	0.19944398	7.35075145	1.971E-13	7.6136E-13
ZNF723	ENSG00000268696	protein_coding	0.99995813	1.01638024	0.47093396	2.15822242	0.03091054	0.04310307
AC008878.3	ENSG00000268861	protein_coding	2.24981743	1.26865817	0.22932045	5.53225061	3.1615E-08	8.2344E-08
FBXO17	ENSG00000269190	protein_coding	6482.42524	1.53036557	0.09469306	16.1613273	9.4505E-59	3.0681E-57
SPIB	ENSG00000269404	protein_coding	64.354162	1.31746829	0.2063539	6.38450877	1.7195E-10	5.3767E-10
AC011455.2	ENSG00000269547	protein_coding	3.74170337	2.83878614	0.27900222	10.1747798	2.5698E-24	1.8942E-23
CT45A10	ENSG00000269586	protein_coding	0.88344992	2.07698814	0.81129024	2.56010492	0.01046406	0.01561671
CCDC194	ENSG00000269720	protein_coding	7.0048058	3.17712874	0.27422951	11.5856559	4.8722E-31	5.0703E-30
AC011473.4	ENSG00000269741	protein_coding	0.69282554	1.57202235	0.72920414	2.15580558	0.03109885	0.04334192
ARHGAP19-SLIT1	ENSG00000269891	protein_coding	0.72269918	1.46936761	0.35645882	4.12212437	3.7539E-05	7.3948E-05
AL365273.2	ENSG00000270099	protein_coding	0.9323456	1.1649727	0.29319147	3.97341942	7.0848E-05	0.00013536
TSNAX-DISC1	ENSG00000270106	protein_coding	1.57965892	1.11616654	0.22708533	4.91518553	8.8698E-07	2.0403E-06
PPIAL4H	ENSG00000270339	protein_coding	1.26691029	2.05312637	0.34946917	5.87498567	4.2288E-09	1.1855E-08
HEATR9	ENSG00000270379	protein_coding	12.3849374	3.43883935	0.24959782	13.7775214	3.4802E-43	6.2148E-42
CTAGE6	ENSG00000271321	protein_coding	2.9973549	2.98108337	0.3006899	9.91414519	3.6134E-23	2.4968E-22
CCL5	ENSG00000271503	protein_coding	4069.25378	3.22018254	0.16493783	19.523614	6.9168E-85	5.7545E-83
MILR1	ENSG00000271605	protein_coding	280.094351	1.97657346	0.11641463	16.9787378	1.18E-64	4.6791E-63
AC233992.2	ENSG00000271698	protein_coding	3.93691534	2.44336845	0.29577021	8.26103633	1.4441E-16	6.8616E-16
AL353588.1	ENSG00000272442	protein_coding	12.0437791	1.65796914	0.15848381	10.4614418	1.2986E-25	1.0296E-24
PCDHB16	ENSG00000272674	protein_coding	876.391187	1.02440918	0.1535361	6.67210608	2.5216E-11	8.4012E-11
AC073610.2	ENSG00000272822	protein_coding	6.44162434	1.82331408	0.17010851	10.7185354	8.3312E-27	7.0411E-26
ATP6V1FNB	ENSG00000272899	protein_coding	335.462708	1.24630062	0.18981046	6.56602693	5.1675E-11	1.6811E-10
AC022400.5	ENSG00000272916	protein_coding	11.8251897	1.3445195	0.15575789	8.63211155	6.023E-18	3.1183E-17
RBAK-RBAKDN	ENSG00000272968	protein_coding	4.33055846	1.03692805	0.15402007	6.73242185	1.6686E-11	5.6244E-11
LYPD4	ENSG00000273111	protein_coding	2.62566023	3.77321689	0.49619839	7.60425061	2.8656E-14	1.1703E-13

TMEM271	ENSG00000273238	protein_coding	0.7861331	2.36576617	0.57381958	4.12283973	3.7423E-05	7.3722E-05
TM4SF19-TCTEX1D2	ENSG00000273331	protein_coding	1.50580551	2.37185862	0.32471225	7.30449377	2.7831E-13	1.0626E-12
TBC1D3K	ENSG00000273513	protein_coding	0.50999679	1.4246586	0.5460269	2.60913631	0.00907711	0.01366559
AGBL1	ENSG00000273540	protein_coding	2.15893918	2.86642971	0.58807686	4.87424328	1.0923E-06	2.4903E-06
H4C12	ENSG00000273542	protein_coding	5.02921558	1.37020948	0.22852923	5.99577342	2.0252E-09	5.834E-09
EPOP	ENSG00000273604	protein_coding	197.38994	1.14194377	0.12124712	9.4183169	4.5838E-21	2.8345E-20
H2BC14	ENSG00000273703	protein_coding	1.80345181	3.34353521	0.45544808	7.34119946	2.1169E-13	8.1553E-13
CEACAM20	ENSG00000273777	protein_coding	4.48930966	3.07847559	0.36046704	8.54024147	1.3394E-17	6.7861E-17
NOL12	ENSG00000273899	protein_coding	239.635797	1.06775515	0.09523544	11.2117422	3.5709E-29	3.4153E-28
H3C8	ENSG00000273983	protein_coding	24.0650004	1.94233116	0.24011225	8.08926321	6.0027E-16	2.7388E-15
GGTLC3	ENSG00000274252	protein_coding	2.62376991	1.00140698	0.40557303	2.46911629	0.01354472	0.01988992
H2BC6	ENSG00000274290	protein_coding	15.6518122	1.5363599	0.20904532	7.3494105	1.9908E-13	7.6865E-13
AL136531.2	ENSG00000274322	protein_coding	19.2540806	1.24497276	0.14878274	8.36772314	5.8742E-17	2.8586E-16
TMEM269	ENSG00000274386	protein_coding	5.4111475	1.69984858	0.19659045	8.64664899	5.3034E-18	2.7567E-17
TBC1D3D	ENSG00000274419	protein_coding	4.0433186	2.97659424	0.60652484	4.90762132	9.2188E-07	2.1165E-06
TBC1D3L	ENSG00000274512	protein_coding	37.0433051	2.23023415	0.21793888	10.2333012	1.4064E-24	1.0521E-23
RIMBP3B	ENSG00000274600	protein_coding	1.35273294	1.82214654	0.27340583	6.66462212	2.6535E-11	8.8258E-11
TBC1D3	ENSG00000274611	protein_coding	0.6713231	1.67749242	0.7179724	2.33643022	0.01946883	0.02797188
H4C6	ENSG00000274618	protein_coding	1.46655321	2.63014986	0.60307728	4.36121534	1.2934E-05	2.6623E-05
H2BC17	ENSG00000274641	protein_coding	4.77962604	2.14616353	0.25534769	8.40486772	4.2834E-17	2.1041E-16
H3C6	ENSG00000274750	protein_coding	45.9502408	1.16002074	0.15500743	7.4836458	7.2289E-14	2.8805E-13
TBC1D3B	ENSG00000274808	protein_coding	5.60533674	2.28621693	0.229245	9.97281043	2.0047E-23	1.4045E-22
NPHP3-ACAD11	ENSG00000274810	protein_coding	4.37288908	1.65589315	0.19483718	8.49885614	1.9147E-17	9.6135E-17
AL139260.3	ENSG00000274944	protein_coding	7.39288935	1.17430068	0.15622399	7.51677543	5.6144E-14	2.2529E-13

H2AC12	ENSG00000274997	protein_coding	1.10052062	2.11336158	0.37299452	5.66593203	1.4623E-08	3.9209E-08
CCL4	ENSG00000275302	protein_coding	909.4265	2.64194808	0.14875116	17.7608573	1.4205E-70	7.0018E-69
H3C11	ENSG00000275379	protein_coding	0.90320978	2.30309125	0.59424852	3.87563649	0.00010635	0.00019969
CCL18	ENSG00000275385	protein_coding	893.218879	5.67630469	0.28431594	19.9647785	1.1152E-88	1.0628E-86
C17orf98	ENSG00000275489	protein_coding	3.08384161	1.51822183	0.3204029	4.73847721	2.1533E-06	4.775E-06
GRIFIN	ENSG00000275572	protein_coding	1.85318036	1.93408753	0.27955173	6.91853183	4.5635E-12	1.6006E-11
H2BC9	ENSG00000275713	protein_coding	12.35021	2.13726845	0.23042449	9.27535283	1.7703E-20	1.0611E-19
RIMBP3	ENSG00000275793	protein_coding	15.7830849	1.34178995	0.13309281	10.0816108	6.6625E-24	4.7931E-23
U2AF1L5	ENSG00000275895	protein_coding	16.7812511	1.3470658	0.24284522	5.54701393	2.9059E-08	7.589E-08
PRSS2	ENSG00000275896	protein_coding	22.8754199	3.04027591	0.31554652	9.6349532	5.6918E-22	3.7004E-21
UHRF1	ENSG00000276043	protein_coding	263.358623	2.75530581	0.13927392	19.7833577	4.1417E-87	3.7301E-85
CCL4L2	ENSG00000276070	protein_coding	440.616554	2.54908611	0.18948394	13.4527818	2.965E-41	4.8769E-40
CCL3L3	ENSG00000276085	protein_coding	199.256124	1.69408012	0.20123488	8.41842179	3.8159E-17	1.8805E-16
H4C9	ENSG00000276180	protein_coding	302.464533	1.1419842	0.13717364	8.32509942	8.4258E-17	4.0636E-16
PIK3R6	ENSG00000276231	protein_coding	264.038434	3.37154751	0.15392382	21.9040004	2.379E-106	4.516E-104
H2AC14	ENSG00000276368	protein_coding	1.61243906	2.67071414	0.43760139	6.10307514	1.0405E-09	3.0696E-09
H2BC3	ENSG00000276410	protein_coding	0.94401535	1.47414026	0.43217624	3.41097013	0.00064732	0.00111552
RAB7B	ENSG00000276600	protein_coding	342.045356	2.04942916	0.1143111	17.9285227	7.0632E-72	3.6339E-70
PADI6	ENSG00000276747	protein_coding	2.26330572	2.82655978	0.35850901	7.88420845	3.1654E-15	1.3787E-14
H2AC16	ENSG00000276903	protein_coding	1.09493174	2.11870815	0.45127771	4.69490983	2.6672E-06	5.8654E-06
GSTT4	ENSG00000276950	protein_coding	1.5911375	1.17339922	0.31308168	3.74790121	0.00017832	0.00032688
H4C5	ENSG00000276966	protein_coding	17.6140832	2.00795728	0.23558651	8.52322685	1.5517E-17	7.8295E-17
H2AC8	ENSG00000277075	protein_coding	88.8338673	1.64832115	0.20937664	7.87251692	3.4758E-15	1.5107E-14
H4C4	ENSG00000277157	protein_coding	5.94596827	1.31476375	0.29775635	4.41556921	1.0074E-05	2.0968E-05
H2BC7	ENSG00000277224	protein_coding	12.0966074	2.78248028	0.30788887	9.03728761	1.6061E-19	9.1311E-19

MARCKS	ENSG00000277443	protein_coding	11168.1343	1.09165064	0.07996222	13.65208	1.962E-42	3.3901E-41
PKD1L3	ENSG00000277481	protein_coding	13.7414417	1.42695728	0.17455097	8.17501795	2.9582E-16	1.3758E-15
CCL3	ENSG00000277632	protein_coding	444.755428	1.82248674	0.16594786	10.9822852	4.6501E-28	4.1863E-27
H3C7	ENSG00000277775	protein_coding	1.50648531	2.51609509	0.41392215	6.07866744	1.2119E-09	3.5522E-09
SENP3-EIF4A1	ENSG00000277957	protein_coding	4.54133269	1.73280328	0.20028417	8.65172376	5.0727E-18	2.638E-17
RDM1	ENSG00000278023	protein_coding	20.5856426	2.01097141	0.16641768	12.0838807	1.2851E-33	1.5163E-32
H2AC4	ENSG00000278463	protein_coding	1.2367316	1.92672196	0.41266357	4.66898973	3.0268E-06	6.6221E-06
H2BC10	ENSG00000278588	protein_coding	2.22991652	2.26229727	0.44510738	5.08258764	3.7233E-07	8.8646E-07
TBC1D3E	ENSG00000278599	protein_coding	2.99174374	2.28751437	0.4256993	5.37354511	7.7203E-08	1.9509E-07
H4C1	ENSG00000278637	protein_coding	1.18070835	1.78981903	0.43480333	4.11638758	3.8486E-05	7.5721E-05
GOLGA6L10	ENSG00000278662	protein_coding	25.6508145	1.4631807	0.16447664	8.89597865	5.7907E-19	3.1882E-18
H2AC17	ENSG00000278677	protein_coding	11.7170483	2.72018154	0.23236836	11.7063335	1.1828E-31	1.2708E-30
IQCA1L	ENSG00000278685	protein_coding	1.23716867	2.57934009	0.37057301	6.96040994	3.3928E-12	1.2012E-11
H4C2	ENSG00000278705	protein_coding	4.33639993	1.09347804	0.3128302	3.49543626	0.00047329	0.00082815
H3C10	ENSG00000278828	protein_coding	177.912042	2.57594047	0.19601148	13.1417836	1.897E-39	2.8987E-38
OR2T11	ENSG00000279301	protein_coding	0.66519684	1.27733841	0.44776736	2.85268313	0.00433518	0.00680439
AC244517.10	ENSG00000279983	protein_coding	0.84639348	1.96785128	0.34045945	5.77998723	7.4706E-09	2.0482E-08
OR12D2	ENSG00000280236	protein_coding	0.82630086	2.2632145	0.80409766	2.81460152	0.00488377	0.0076115
AC068946.1	ENSG00000280537	protein_coding	42.9510451	1.44677578	0.17589417	8.225263	1.9476E-16	9.1589E-16
AC005154.5	ENSG00000281039	protein_coding	2.35514342	1.99347405	0.29094458	6.85173119	7.2962E-12	2.526E-11
TMEM272	ENSG00000281106	protein_coding	10.4587836	1.13360678	0.16437086	6.89664079	5.3247E-12	1.8591E-11
AC120114.4	ENSG00000281348	protein_coding	4.5790513	1.31214197	0.1501584	8.73838525	2.3647E-18	1.2543E-17
AC006978.2	ENSG00000281593	protein_coding	4.03743678	1.19302307	0.20202124	5.90543396	3.5172E-09	9.9232E-09
