# Supplementary material for: Identification and Validation of a Novel Ferroptotic Prognostic Genes-Based Signature of Clear Cell Renal Cell Carcinoma
Source: Cancers (Basel). 2022 Sep 27;14(19):4690. doi: 10.3390/cancers14194690 (PMC9562262; doi:10.3390/cancers14194690)
Supplement: Supplementary file 1 [file cancers-14-04690-s001.zip › Table S5 List of 41 DEGs.pdf]

Table S5 List of 41 DEGs.

Gene name
DPEP1
NOX4
TYRO3
MIOX
VDR
MT1G
CDO1
GCH1
GLS2
GABARAPL1
LCN2
PROM2
AQP3
ACSF2
GLRX5
ALOX5
CP
SCD
TIMP1
SLC39A14
PPP1R13L
TGFB1
CAV1
EZH2
CA9
IFNG
CDCA3
KIF20A
IDO1
HILPDA
EGFR
PLIN2
CDKN2A
SLC7A11
GJA1
SLC16A1
CYGB
CYBB
RRM2
NUPR1
P4HB
