# Supplementary material for: Identification and Validation of a Novel Ferroptotic Prognostic Genes-Based Signature of Clear Cell Renal Cell Carcinoma
Source: Cancers (Basel). 2022 Sep 27;14(19):4690. doi: 10.3390/cancers14194690 (PMC9562262; doi:10.3390/cancers14194690)
Supplement: Supplementary file 1 [file cancers-14-04690-s001.zip › Table S6 List of 207 TPGs.pdf]

Table S6 List of 207 TPGs.

Gene name
IREB2
ATP5MC3
CS
EMC2
CYBB
NOX4
VDAC2
PIK3CA
FLT3
SCP2
LPCAT3
NRAS
KRAS
TF
TFR2
SLC38A1
SLC1A5
GLS2
GOT1
HMOX1
ATG5
ATG7
NCOA4
ALOX12B
ALOXE3
PHKG2
ACO1
ATG3
ATG4D
BECN1
GABARAPL2
GABARAPL1
SNX4
MAPK3
MAPK1
ZEB1
DPP4
CDKN2A
PEBP1
SOCS1
MAPK8

MAPK9
CHAC1
MAPK14
PRKAA2
PRKAA1
ELAVL1
BAP1
ACVR1B
TGFBR1
EPAS1
ANO6
HMGB1
TNFAIP3
TLR4
MIOX
TAZ
MTDH
SIRT1
CD82
CYB5R1
PTEN
IL6
USP7
AQP8
PEX10
AGPAT3
PEX12
CHP1
GPAT4
BRPF1
OSBPL9
INTS2
AEBP2
AGPS
CDCA3
PEX2
TIMM9
DCAF7
AMN
PEX3
MTCH1
ACADSB
MAP3K11

GSK3B
BRD7
MFN2
TSC1
SIRT3
DLD
WWTR1
LGMN
MYCN
SMG9
PPARG
SNX5
MICU1
QSOX1
CLTRN
KLF2
FOXO4
YTHDC2
SLC39A7
TRIM46
ACSL1
KDM5A
TRIM21
DPEP1
GSTZ1
GJA1
PGRMC1
CIRBP
TRIM26
NDRG1
LIFR
EGR1
CPEB1
TIMP1
KDM6B
METTL14
MIB1
CCDC6
SLC40A1
NFE2L2
AKR1C1
AKR1C2
AKR1C3

RB1
GCLC
NQO1
MT1G
SRC
MTOR
NFS1
CDKN1A
ENPP2
FH
CISD2
CBS
ACSL3
NF2
JUN
CA9
PLIN2
LAMP2
PROM2
CHMP5
CHMP6
RRM2
NR4A1
SREBF2
BCAT2
PLA2G6
ATF2
ACOT1
ALDH3A2
CDH1
NEDD4L
BRD2
BRD3
DECR1
GLRX5
NCOA3
PANX2
TFAP2A
ARF6
PPP1R13L
TFAM
KDM3B
AHCY

PPARA
NOS2
RELA
NEDD4
AR
MTF1
USP35
PARP4
PARP6
PARP11
PARP15
PDSS2
CREB1
CREB3
BEX1
ASAH2
SIRT6
KIF20A
VCP
KDM4A
MPC1
SOX2
SIRT2
MEF2C
EZH2
ADAMTS13
ENO3
LCN2
MARCHF5
TRIB2
DHODH
PDK4
CISD3
MS4A15
FURIN
GALNT14
KLHDC3
